# Transcriptomics and Comparative Analysis of Three Antarctic Notothenioid Fishes

**DOI:** 10.1371/journal.pone.0043762

**Published:** 2012-08-16

**Authors:** Seung Chul Shin, Su Jin Kim, Jong Kyu Lee, Do Hwan Ahn, Min Gyu Kim, Hyoungseok Lee, Jungeun Lee, Bum-Keun Kim, Hyun Park

**Affiliations:** 1 Korea Polar Research Institute, Yeonsu-gu, Incheon, South Korea; 2 College of Life Sciences and Biotechnology, Korea University, Seongbuk-gu, Seoul, South Korea; 3 University of Science & Technology, Yuseong-gu, Daejeon, South Korea; 4 Korea Food Research Institute, Bundang-gu, Sungnam, South Korea; University of North Carolina at Charlotte, United States of America

## Abstract

For the past 10 to 13 million years, Antarctic notothenioid fish have undergone extraordinary periods of evolution and have adapted to a cold and highly oxygenated Antarctic marine environment. While these species are considered an attractive model with which to study physiology and evolutionary adaptation, they are poorly characterized at the molecular level, and sequence information is lacking. The transcriptomes of the Antarctic fishes *Notothenia coriiceps*, *Chaenocephalus aceratus*, and *Pleuragramma antarcticum* were obtained by 454 FLX Titanium sequencing of a normalized cDNA library. More than 1,900,000 reads were assembled in a total of 71,539 contigs. Overall, 40% of the contigs were annotated based on similarity to known protein or nucleotide sequences, and more than 50% of the predicted transcripts were validated as full-length or putative full-length cDNAs. These three Antarctic fishes shared 663 genes expressed in the brain and 1,557 genes expressed in the liver. In addition, these cold-adapted fish expressed more Ub-conjugated proteins compared to temperate fish; Ub-conjugated proteins are involved in maintaining proteins in their native state in the cold and thermally stable Antarctic environments. Our transcriptome analysis of Antarctic notothenioid fish provides an archive for future studies in molecular mechanisms of fundamental genetic questions, and can be used in evolution studies comparing other fish.

## Introduction

Antarctic fish have undergone extraordinary evolutionary episodes since the onset of widespread glaciation in Antarctica approximately 34 million years ago, when the Southern Ocean cooled to the freezing point of seawater (−1.9°C) [1]. The Antarctic fish fauna are dominated by the perciform suborder Notothenioidei, which represents 77% of the species diversity and 91% of the biomass. There are currently 322 recognized species of Antarctic fishes, and a total of 132 notothenioid species are known [2]. Notothenioids have survived in the subzero waters of the continental shelf and may have experienced a unique type of adaptive radiation known as species flock [3], [4]. Notothenioid fishes possess a wide range of unique adaptations to the extreme Antarctic environment, such as antifreeze glycoproteins, loss of heat shock response [5], and lack of hemoglobin [6], [7]. The Antarctic notothenioid antifreeze glycopeptides are derived from a related pancreatic trypsinogen-like protease [8], [9], and they represent key evolutionary adaptations to life in subzero ice-laden water. Previous research on Antarctic notothenioid fishes has shown that these cold-adapted species lack a common cellular defense mechanism called the heat shock response, which involves the highly conserved and coordinated induction of a family of heat shock proteins in response to elevated temperatures [5], [10], [11]. Other important phenotypic features are the loss of erythrocytes and hemoglobin and the variable patterns of myoglobin expression in muscle tissues of white-blooded channichthyids [12], [13]. The notothenioids have undergone resistant and compensatory adaptations to the extreme Antarctic marine environment as well as regressive evolutionary changes. Thus, they are considered an attractive model species for evolutionary and physiological studies [4], [14].

Chen et al. [11] reported expressed sequence tag (EST) sequencing from Antarctic notothenioid *Dissostichus mawsoni* tissues and compared them to tissues of temperate/tropical teleost fish. They identified 177 notothenioid protein families that were overexpressed, which suggests that these protein families are upregulated by low temperatures. Further analysis of these upregulated genes indicated substantial expansion by gene duplication of 118 gene families involved in metabolic processes such as protein biosynthesis, folding and degradation, and lipid metabolism. This suggests that gene duplication may function as an adaptive strategy for organisms under freezing conditions. Detrich et al. [15] determined the genomic sizes of 11 notothenioid species including perches, notothes, dragonfish, and icefish, which have variable genome sizes ranging from 0.90 to 1.83 pg, and found that the evolution of phylogenetically derived notothenioid families was accompanied by genome expansion. The icefish (channichthyids), which are considered the most phylogenetically derived group within the notothenioids, have the largest genomes. Evolution in chronic cold and stable temperature conditions have resulted in these species lacking any erythrocytes or hemoglobin genes [6], [16], [17], [18] and in variable patterns of myoglobin expression in muscle tissues in cold, well-oxygenated seawater [12], [13].

**Figure 1:**
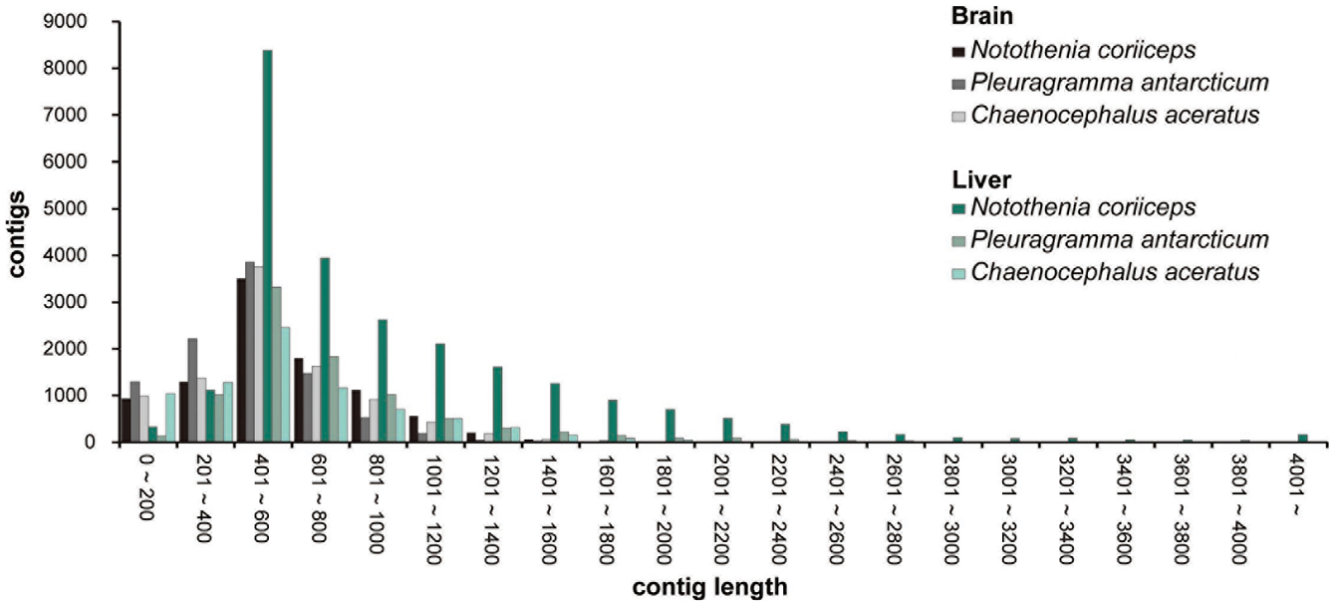
Contig distribution of three notothenioid fish transcriptome sequences.

**Table 1:** Statistics for pyrosequencing of the three notothenioid species.

Species	*N. coriiceps*		*P. antarcticum*		*C. aceratus*	
Tissue	Liver	Brain	Liver	Brain	Liver	Brain
Total no. of reads	835,611	194,267	179,515	293,042	220,146	195,902
Total size (bases)	234,713,842	58,080,538	67,023,904	86,741,806	74,665,023	62,949,666
Average length (bases)	281	299	373	296	339	321
Total no. of contigs	24,836	9,532	10,271	9,671	7,815	9,414
Total no. of reads within contigs	617,734	93,296	156,123	110,825	105,997	84,670
Total size within contigs (bases)	176,382,863	29,571,950	58,290,242	34,842,269	35,355,477	26,981,344
Average length of contigs (bases)	966	582	701	488	604	502
Significant BLAST hits (e≤10^−10^)	10,271	2,782	5,325	3,657	3,700	2,989

Although ESTs represent only a subset of the entire eukaryotic genome, their sequencing is helpful for investigating the transcriptome rather than the genome of an organism. It also allows one to focus on the genome sections with high levels of functional information, avoiding introns and intragenic regions that can complicate data analysis [19]. Next-generation sequencing technologies, such as 454 pyrosequencing, offer novel and rapid approaches for genome-wide characterization and profiling of mRNAs, small RNAs, transcription factor regions, chromatin structure, DNA methylation patterns, and metagenomics [20]. Pyrosequencing of ESTs provides an efficient way to generate sequence data for non-model organisms in the form of transcriptome sequencing, and can be used to characterize gene expression and identify novel genes [21], [22], [23]. The availability of complete genome sequences and large sets of ESTs from several fish species have stimulated the development of efficient and informative techniques for large-scale and genome-wide analysis of gene expression and comparative genomics.

**Figure 2:**
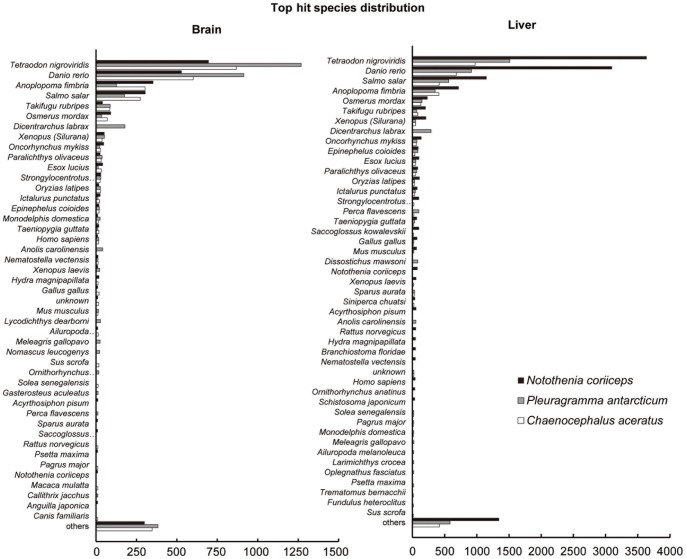
Species distribution of three notothenioid fishes based on BLAST hits from nr sequence database.

**Table 2:** Summary of full-length cDNA in the three notothenioids species.

	*N. coriiceps*		*P. antarcticum*		*C. aceratus*	
	Liver	Brain	Liver	Brain	Liver	Brain
No. of full-length	13,850	4,811	4,745	3,205	3,372	4,389
No. of putative full-length	572	161	357	254	230	172
No. of non-full-length	10,414	4,560	5,169	6,212	4,213	4,853
No. of total sequences	24,836	9,532	1,0271	9,671	7,815	9,414

We herein describe the transcriptomes of three Antarctic notothenioid fishes, *Notothenia coriiceps*, *Chaenocephalus aceratus*, and *Pleuragramma antarcticum*, by 454 FLX Titanium sequencing. These three notothenioid fishes are characterized by distinct biological and ecological traits, although, like other members of this suborder, all of them lack a gas bladder. *N. coriiceps* (family Nototheniidae) retained an ancestral notothenioid benthic habitus. Instead, *P. antarcticum* (family Nototheniidae) and *C. aceratus* (family Channicthyidae) were subjected to trophic evolution towards a pelagic lifestyle, involving an important suite of adaptations [24]. Among them *C. aceratus* is a benthopelagic species while *P. antarcticum* is a true pelagic species living all stages of its development within the water column [25]. We herein report the generation of more than 71,539 contigs from these three Antarctic fish species. Forty percent of the contigs (28,724 BLAST hits of the 71,539 contigs) could be annotated based on similarity to known protein or nucleotide sequences. Our work represents an ongoing genome project studying *N. coriiceps* (NCBI Genome Project ID #66471), and this initial study identified EST sequences expressed in two tissues (liver and brain) of *N. coriiceps* and in two tissues of *C. aceratus* and *P. antarcticum* for comparative analyses. This represents the first report of publicly available pyrosequencing data for Antarctic fish and provides an important comparative resource for studies of physiology and evolutionary adaptation in fish biology.

**Table 3:** Summary of microsatellite marker identification in the three notothenioid species.

	*N. coriiceps*		*P. antarcticum*		*C. aceratus*	
	Brain	Liver	Brain	Liver	Brain	Liver
Total no. of unique sequences	9,532	24,836	9,671	10,271	9,414	7,815
Microsatellites identified	267	1,350	299	816	274	201
Dinucleotide	94	441	103	500	88	57
Trinucleotide	146	821	181	277	170	126
Tetranucleotide	27	79	9	25	15	14
Pentanucleotide	0	3	4	12	1	3
Hexanucleotide	0	6	2	2	0	1
No. of unique sequences containing microsatellites	250	1,178	281	708	259	192
No. of unique sequences containing microsatellites with sufficient flanking sequences for PCR primer design	183	961	203	387	181	135

## Materials and Methods

### Ethics Statement

This study including sample collection and experimental research conducted on these animals was according to the law on activities and environmental protection to Antarctic approved by the Minister of Foreign Affairs and Trade of the Republic of Korea.

### Sample collection

*N. coriiceps* (length 35 cm), *C. aceratus* (length 32 cm), and *P. antarcticum* (length 13 cm) were collected in the Antarctic Peninsula (62°14'S, 58°47'W) from December 2009 to January 2010. Benthic nearshore specimens of *N. coriiceps* and *C. aceratus* were obtained using the hook-and-line method from depths of 20 to 30 m. Cryopelagic specimens of *P. antarcticum* were caught in traps. After capture, these fish were maintained in flow-through aquaria at ambient seawater temperatures (−1.5°C) for 48 h before sacrifice. Brain and liver tissues of each specimen were dissected, immediately frozen in liquid nitrogen, and stored at −80°C until use.

**Table 4:** Functional annotation of proteins encoded in the transcriptomes of the three notothenioid fish based on gene ontology (GO).

	No. of sequences (%)					
	*N. coriiceps*		*P. antarcticum*		*C. aceratus*	
	Liver	Brain	Liver	Brain	Liver	Brain
**Biological process**						
Biological adhesion	357(1.12)	56(0.91)	105(0.70)	205(1.99)	70(0.76)	205(1.99)
Biological regulation	3,460(10.90)	675(10.92)	1,621(10.83)	1,238(12.04)	1,024(11.05)	1,238(12.04)
Cell proliferation	599(1.89)	96(1.55)	240(1.60)		159(1.72)	
Cellular component biogenesis	937(2.95)	195(3.16)	378(2.53)	294(2.86)	240(2.59)	294(2.86)
Cellular component organization	1,983(6.25)	348(5.63)	760(5.08)	722(7.02)	446(4.81)	722(7.02)
Cellular process	6,242(19.67)	1,442(23.34)	3,154(21.08)	2,093(20.35)	2,015(21.75)	2,093(20.35)
Death	775(2.44)	121(1.96)	358(2.39)	256(2.49)	210(2.27)	256(2.49)
Developmental process	2,143(6.75)	339(5.49)	826(5.52)	833(8.10)	520(5.61)	833(8.10)
Growth	285(0.90)	52(0.84)	148(0.99)		87(0.94)	
Immune system process	634(2.00)	75(1.21)	265(1.77)		169(1.82)	
Localization	2,071(6.53)	426(6.89)	1,051(7.02)	868(8.44)	676(7.30)	868(8.44)
Locomotion	380(1.20)	49(0.79)	126(0.84)		86(0.93)	
Metabolic process	4,765(15.01)	1,195(19.34)	2,758(18.43)	1,415(13.76)	1,714(18.50)	1,415(13.76)
Multicellular organismal process	2,546(8.02)	391(6.33)	994(6.64)	989(9.62)	614(6.63)	989(9.62)
Multiorganism process	391(1.23)	74(1.20)	232(1.55)		132(1.42)	
Pigmentation	48(0.15)		26(0.17)			
Reproduction	448(1.41)	70(1.13)	200(1.34)		131(1.41)	
Response to stimulus	1,883(5.93)	312(5.05)	1,012(6.76)	572(5.56)	554(5.98)	572(5.56)
Rhythmic process	66(0.21)		28(0.19)		18(0.19)	
Signaling	1,725(5.44)	263(4.26)	682(4.56)	801(7.79)	401(4.33)	801(7.79)
**Cellular component**						
Cell	7,192(42.22)	1,679(41.65)	3,564(41.68)	2,414(44.69)	2,321(42.32)	1,220(43.31)
Extracellular region	569(3.34)	102(2.53)	391(4.57)	157(2.91)	231(4.21)	82(2.91)
Macromolecular complex	2,311(13.57)	666(16.52)	1,159(13.56)	778(14.40)	772(14.08)	428(15.19)
Membrane-enclosed lumen	1,559(9.15)	312(7.74)	768(8.98)	375(6.94)	445(8.11)	184(6.53)
Organelle	5,256(30.86)	1,235(30.64)	2,625(30.70)	1,482(27.43)	1,682(30.67)	812(28.82)
Synapse	147(0.86)	37(0.92)	43(0.50)	196(3.63)	33(0.60)	91(3.23)
**Molecular function**						
Antioxidant activity	51(0.39)	18(0.61)	28(0.42)		17(0.39)	14(0.60)
Binding	6,566(49.91)	1,460(49.58)	3,127(46.58)	2,065(49.20)	2,087(48.00)	1,132(48.67)
Catalytic activity	3,527(26.81)	828(28.12)	2,122(31.61)	996(23.73)	1,311(30.15)	591(25.41)
Channel regulator activity	23(0.17)					13(0.56)
Electron carrier activity	111(0.84)	38(1.29)	106(1.58)		50(1.15)	14(0.60)
Enzyme regulator activity	422(3.21)	82(2.78)	253(3.77)	182(4.34)	140(3.22)	78(3.35)
Molecular transducer activity	583(4.43)	108(3.67)	252(3.75)	317(7.55)	168(3.86)	127(5.46)
Structural molecule activity	571(4.34)	144(4.89)	201(2.99)	137(3.26)	167(3.84)	104(4.47)
Transcription regulator activity	664(5.05)	119(4.04)	261(3.89)	184(4.38)	167(3.84)	90(3.87)
Translation regulator activity	21(0.16)		15(0.22)		11(0.25)	
Transporter activity	617(4.69)	148(5.03)	348(5.18)	316(7.53)	230(5.29)	163(7.01)

### cDNA preparation and sequencing

Total RNA was isolated by homogenization of each sample in a TRIzol (Invitrogen, Carlsbad, CA)/chloroform mixture, followed by processing using an RNeasy mini kit (Qiagen, Chatsworth, CA) for DNAse treatment and cleaning. RNA quality and quantity were analyzed using an Agilent 2100 Bioanalyzer (Agilent Technologies, Palo Alto, CA) and Nanodrop ND1000 (NanoDrop Technologies, Wilmington, DE), respectively. First- and second-strand cDNA were synthesized from 200 ng mRNA using a SuperScript Double-Stranded cDNA Synthesis Kit (Invitrogen) with 100 mM random hexamer primers (Macrogen, Seoul, Korea). Double-stranded cDNA was purified with a QIAquick MinElute PCR purification column (Qiagen). The cDNA library was normalized according to the protocol described in the Trimmer Direct Kit (Evrogen, Moscow, Russia). Briefly, 300 ng cDNA was denatured at 95°C for 5 min and allowed to renature at 68°C for 5 h in the hybridization buffer included with the kit (50 mM HEPES, pH 7.5, and 0.5 M NaCl). After incubation, the reaction mixture was treated with 1 ml 4-fold-diluted duplex-specific nuclease. Then normalized cDNA was amplified using PCR Advantage II polymerase (Clontech, Palo Alto, CA). After library construction, the samples were quantified using a Qubit fluorometer (Invitrogen), and average fragment sizes were determined by analyzing 1 ml samples on a bioanalyzer (Agilent) using a DNA 7500 chip. Approximately 10 mg cDNA from each of the six samples was used for sequencing on a GS-FLX Titanium platform (454 Life Sciences, Branford, CT) at the DNA Link Inc. facility (Seoul, Korea) according to the manufacturer's protocol.

**Figure 3:**
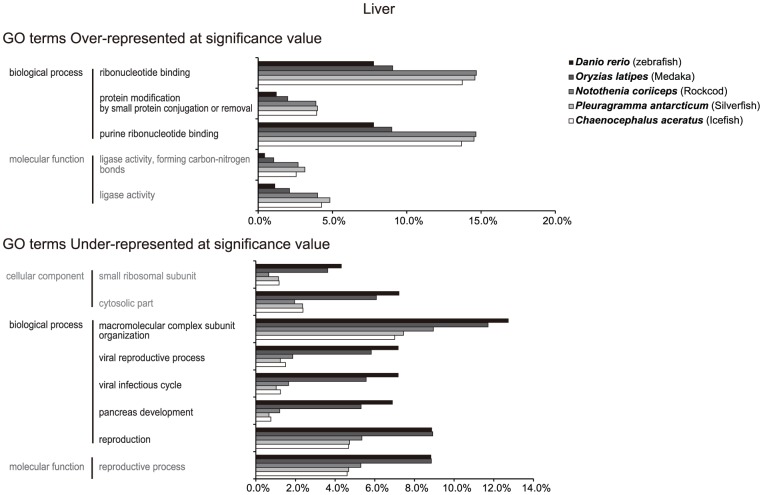
Gene ontology (GO) enrichment analysis of five fish liver transcriptomes: three notothenioid fish, zebra fish, and medaka.

**Figure 4:**
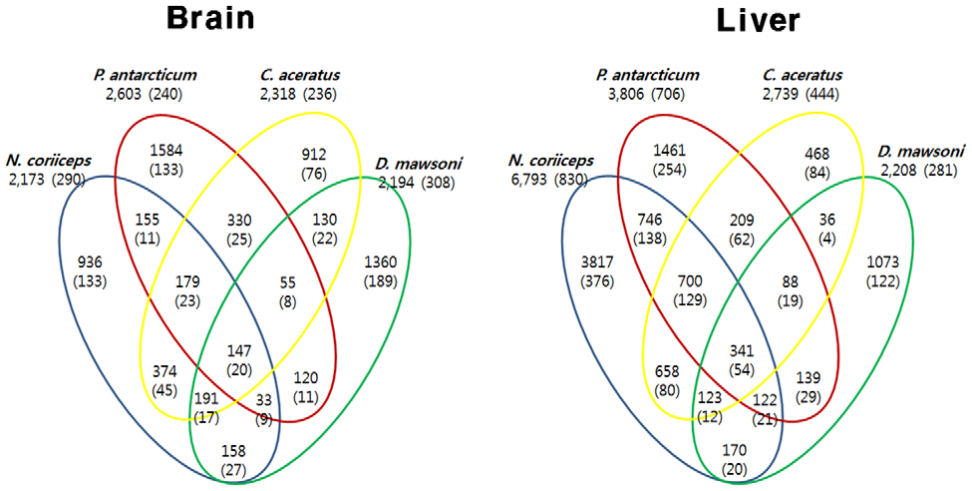
Comparison of shared and unique genes identified in four notothenioid fishes. Numbers in parentheses represent the total number of enzymes in metabolic pathway analysis.

### Bioinformatic analysis

The raw 454 sequence files in sff format were processed and assembled using Newbler. The resulting contigs were subjected to a BLASTx search against the non-redundant protein database (nr) with an e-value threshold of 10^−3^ and HSP length cutoff of 33. The gene ontology (GO) terms were assigned to each unique gene based on the GO terms annotated to the corresponding homologs in the UniProt database. GO mapping and annotation were performed with an annotation cutoff of 10^−10^. Enrichment analysis was performed using Fisher's exact test. All analyses were performed using the BLAST2GO program [26]. Identification of metabolic genes was accomplished by MetaFishNet computation [27]. All cDNA sequences of *D. mawsoni* were retrieved from the National Center for Biotechnology Information (NCBI). Putative full-length cDNAs were identified by comparing full-length genes and start signals in the UniProt and nr databases to those of ORF prediction using the software Full-Lengther [28] with a cutoff e-value of 1E^−5^. Once the start codon (ATG) and poly(A) tail had been identified, the sequence was considered a full-length cDNA. The unique sequences of each teleost fish tissue were used to search for microsatellite markers using msatcommander (http://code.google.com/p/msatcommander/) with a repeat threshold of eight dinucleotide repeats or five tri-, tetra-, penta-, and hexanucleotide repeats. The unique genes and homologous genes of the three Antarctic notothenioids were identified using BLASTX against the NCBI Refseq protein and Ensembl databases (*Tetraodon nigroviridis*, zebrafish *Danio rerio*, and Atlantic salmon *Salmo salar*) with an e-value threshold of 10^−10^.

**Figure 5:**
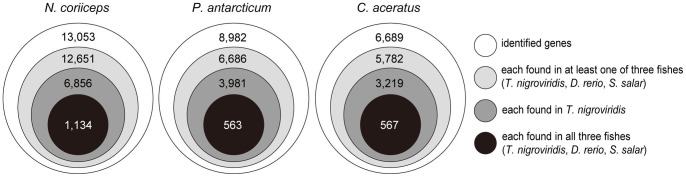
Conservation of three notothenioid fish genes with other species. Number of notothenioid fish homologous genes annotated in GO analysis.

## Results and Discussion

### Sequence assembly

Each liver and brain cDNA library of *C. aceratus* and *P. antarcticum* and the brain cDNA library of *N. coriiceps* were subjected to a one-quarter plate run, and the liver cDNA library of *N. coriiceps* was subjected to a one-plate run with the 454 GS-FLX Titanium platform. After removing low-quality regions, adaptors, and all possible contaminants, we obtained a total of 1,918,483 high-quality reads containing 584,174,779 bases from all of six libraries with average read length 318 bases, and sequencing depth of each library were 10∼15X (Table 1). A de novo assembly was performed for each of the six samples independently. The cleaned read data were entered into Newbler for assembly; the size-selected reads were assembled into 7,815 and 9,414 contigs from the liver and brain of *C. aceratus*, 10,271 and 9,671 contigs from the liver and brain of *P. antarcticum*, and 24,836 and 9,532 contigs from the liver and brain of *N. coriiceps*, respectively. The contigs ranged in size from 152 to 4,012 bp with an average size of 604 bp in the liver, and from 239 to 2,951 bp with an average size of 502 bp in the brain of *C. aceratus*. In *P. antarcticum*, they ranged from 102 to 7,900 bp with an average size of 701 bp in the liver, and from 100 to 2,520 bp with an average size of 488 bp in the brain. In *N. coriiceps*, they ranged from 371 to 6,171 bp with an average size of 966 bp in the liver, and from 238 to 2,457 bp with an average size of 582 bp in the brain. In total, 523 contigs were greater than 3 kb in length, and 2,179 contigs were composed of more than 300 reads, with the largest contig being 12,625 bp composed of 1,108 sequences, which this contig was annotated to titin. The size distribution of the reads is shown in Figure 1. All high-quality reads have been deposited in the NCBI and can be accessed in the Short Read Archive (SRA) under the accession number SRP007644. Table 1 presents a summary of the sequencing and assembly results, and all transcriptome information of the three fishes is accessible at http://antagen.polar.re.kr. Although the singletons potentially contained useful sequences with low levels of expression, they included short reads, and a small number of redundant sequences and singleton reads were excluded from further analysis. The genome sizes of two of these notothenioids (*N. coriiceps*, C = 1.13 pg and *C. aceratus*, C = 1.73 pg) were recently described [15], but the percentages of the transcribed genomes remain unknown. Thus, it is difficult to predict the depth of coverage of the Antarctic fish transcriptome by our *de novo* assembled sequences.

A total of 28,724 contigs had a significant BLASTx hit at a cutoff value of <1E^−10^ in the nr protein database: 6,689 of 17,229 contigs (38.8%) from *C. aceratus*, 8,982 of 19,942 contigs (45.0%) from *P. antarcticum*, and 13,053 of 34,368 contigs (38.0%) from *N. coriiceps*, respectively. To obtain an overall view of the transcriptome, these commonly expressed sequences in each tissue of the three fish (with an associated database match) represented a varied mix of functional groups (Table S1). However, in terms of sequence completeness, an estimate of the fraction of full-length sequences in the transcriptome was obtained. A sequence was considered full-length when it included the complete 5′ and 3′ sequences of the mRNA. We used the software Full-Lengther [28], and 36% to 52% of predicted transcripts were validated as full-length or putative full-length in each tissue of the three fish species (Table 2). Among these, 3,170 (41%) from the liver and 2,484 (26%) from the brain of *C. aceratus*, 10,188 (41%) from the liver and 2,209 (23%) from the brain of *N. coriiceps*, and 4,757 (46%) from the liver and 3,012 (31%) from the brain of *P. antarcticum* had significant BLAST matches. As expected, the majority of the sequences (81.2%) showed matches with teleost fish, with eukaryotes accounting for 89.0% of positive hits. Among the fish, the pufferfish *Tetraodon nigroviridis* showed the highest percentage of hits (24.6%), and the zebrafish *Danio rerio* represented approximately 13.6% of all hits (Fig. 2).

A total of 3,207 microsatellites were identified from 71,539 unique sequences from six libraries, including di-, tri-, tetra-, penta-, and hexanucleotide repeats (Table 3). Previous observations were reported that 454 pyrosequencing in transcriptomic studies were shown to be an excellent method for large scale prediction of molecular markers for future genetic linkage in non-model organisms [29], [30]. Therefore, given that these microsatellite predicted from transcriptomic sequences, they are likely linked to protein-coding genes, might have substantial physiological implications.

### Gene ontology

The transcripts of the six libraries were assigned GO terms based on BLAST matches (Table 4). GO assignments were divided into molecular function, biological process, and cellular components. Predicted proteins assigned to biological process were mainly associated with cellular processes (20%–23%), metabolic processes (14%–19%), and biological regulation processes (11%–12%). Those assigned to molecular function were mainly linked to the binding of ATP, zinc ions, and protein (47%–50%); catalytic activities of enzymes (25%–32%); and transporter activity (5%–8%). Finally, those assigned to cellular components included intracellular locations (42%–43%), organelles (29%–31%), and macromolecular complexes (14%–17%).

GO enrichment analysis was performed on the three notothenioid fish, and these were compared to the transcriptome database of zebrafish and medaka (*Oryzias latipes*) (NCBI library IC, 14,410 for liver and 1,522 for brain of zebrafish; 17,414 for liver and 8,625 for brain of medaka) because tissue-specific transcriptomes of these two fishes are well known in public databases (Fig. 3). Five GO terms were significantly overexpressed in the liver of Antarctic fish relative to the temperature/tropical fish: ribonucleotide binding, protein modification by small protein conjugation or removal, purine nucleotide binding in the biological process category, and ligase activity in the molecular function category. Eight terms were underrepresented in the liver, including the small ribosomal subunit and cytosolic parts in the cellular component category; macromolecular complex subunit organization, viral reproductive process, viral infectious cycle, pancreas development, and reproduction in the biological process category; and reproductive process in the molecular function category. Of the overrepresented molecular function terms, ligase activity terms were primarily composed of ubiquitin (Ub)-conjugated protein (75 of 339 genes in *N. coriiceps*, 27 of 120 genes in *C. aceratus*, and 45 of 203 genes in *P. antarcticum*). The Ub-proteasome pathway is a cytosolic protein-degradation pathway of misfolded or damaged proteins that takes place two distinct and successive steps. The first step involves tagging of the misfolded or damaged protein by multiple Ub molecules and degradation of the tagged protein by the 26S proteasome complex [31], [32], [33]. Transcriptomic analysis of another Antarctic notothenioid fish, *D. mawsoni*, also revealed high levels of Ub-conjugated proteins compared to temperate/tropical teleosts [11]. Antarctic fish may have unusually high levels of misfolded or damaged proteins because low temperatures may affect the rate of protein folding [34]. Previous studies have shown that Antarctic notothenioid fish lack a common cellular defense mechanism, such as the heat shock response [5], [10], [35]. These cold-adapted fish require an alternative cellular protein homeostasis mechanism to ensure proper cell functioning. These findings suggest that increased levels of Ub-conjugated proteins in Antarctic fish may be involved in maintaining proteins in their native state in the cold and thermally stable Antarctic environments.

### Comparative analysis among four notothenioid species

A total of 28,724 genes were identified from the three notothenioid species based on a BLAST search. Previously, Chen et al. characterized ESTs of *D. mawsoni* [11]. Therefore, we compared expressed transcriptomes of the liver and brain among these four notothenioid species to cross matching using tBLASTn. A total of 331 genes expressed in the liver and a total of 191 genes expressed in the brain were shared among the four species (e-value, 1E^−10^) (Fig. 4). In the three fishes focused on in this research, a total of 663 genes expressed in the brain and a total of 1,557 genes in the liver were shared (e-value, 1E^−10^). The summary of shared genes and identified genes among all species is shown in Table S2 and Table S3.

Li et al. [27] reported the construction of a genome-wide fish metabolic network model to identify and compare the metabolic pathway. They categorized 115 metabolic pathways from 5 fish genomes (*D. rerio, O. latipes, Takifugu rubripes, T. nigroviridis*, and *Gymnopilus aculeatus*) to create a list of all fish metabolic genes via gene ontology. And they identified the corresponding enzymes using either orthologous relationships to human genes or similarity to consensus enzyme sequences from this metabolic gene list. We analyzed all cDNA sequences from the four notothenioid fishes and 88 metabolic pathways were assigned; that is, no enzymes in 27 of these metabolic pathways were found mainly lipid related pathway, such as glycosphingolipid biosynthesis, mono-unsaturated fatty acid betaoxidation, omega-3 fatty acid metabolism, and sphingolipid metabolism, compared with the fish metabolic genes to other temperate/tropical teleosts (Table S4). In contrast, we have noticed that the enzyme in electron transport chain were more mapping than that in temperate/tropical fishes, that suggests greater demands for these functions in the cold Antarctic environment.

To assess the evolutionary conservation of the genes, the number of genes with homologs in *Tetraodon*, zebrafish, and Atlantic salmon (*Salmo salar*), which were the primary BLAST results, were compared (Fig. 5). A total of 3,605 genes (402 in *N. coriiceps*, 2296 in *P. antarcticum* and 907 in *C. aceratus*. 12.6% of the total number of unique notothenioid fish genes) were found. Among these genes, 1,134 (8.7%) from *N. coriiceps*, 563(6.3%) from *P. antarcticum* and 567 (8.5%) from *C. aceratus* were commonly found in all three species (*Tetraodon*, zebrafish, and Atlantic salmon).

The 15 known species of icefish and white-blooded fish, including *C. aceratus*, all lack the hemoglobin gene [1], [36]. This phenotype preceded the evolutionary radiation of the icefish. Di Prisco et al. [6] showed that the *C. aceratus* genome has transcriptionally inactive truncated variants of α1-globin-related DNA and lacks β-globin genes. They found that the *C. aceratus* transcriptome contained only cytoglobin for oxygen transport and/or oxygen-binding machinery (Table S5). Cytoglobin is one of four types of globin (hemoglobin, myoglobin, neuroglobin, and cytoglobin), which differ in structure, tissue distribution, and likely function, but mainly serve to transport oxygen in the circulatory system [37]. To determine the molecular phylogenetic position of *C. aceratus* cytoglobin, a phylogenetic tree was constructed using the neighbor-joining method from a distance matrix, calculated with MEGA4 [38]. Cytoglobin was grouped with the fish cytoglobin cluster (Figure S1). There have been no previous reports of cytoglobin sequences from other icefish species. The cytoglobin of *C. aceratus* showed the highest level of identity (72%) to that of *O. latipes* based on amino acid similarity (Figure S2). The mechanism of the compensatory physiological and circulatory adaptations that resulted in replacement of the lost hemoglobin and myoglobin functions remains unknown. Recently, Cheng et al. [39] hypothesized that neuroglobin may play a role in oxygen transport because this gene is widely found in icefish despite the fact that this fish has generally lost hemoglobin and myoglobin. The observation that at least one icefish have retained the cytoglobin gene is intriguing, and the function of the cytoglobin gene should be further explored to address the evolutionary development and alternative physiology of losing globin genes.

## Conclusions

We generated and assembled the transcriptomes of three Antarctic notothenioid fish species. We generated more than 71,539 contigs, identified more than 28,724 unique genes expressed in the brain and liver of the three Antarctic fish, and identified more than 3,200 gene-associated microsatellites. The Antarctic fish transcriptome, the analyzed by high-throughput 454 sequencing, can be functionally characterized for a wide range of molecules encoded in the transcriptomes of members of the notothenioid. Comparative sequencing of the three notothenioid fish transcriptomes also provided information on the variation in evolution and speciation of species that live at permanently cold temperatures. We are currently performing whole-genome sequencing of *N. coriiceps*. Comparison between genome and transcriptome sequences will allow for a better understanding of gene structure and organization in molecular mechanisms of fundamental genetic questions and furthermore provide a comprehensive view into evolution studies to environmental challenges during climate changes.

## Supporting Information

Figure S1 **Phylogenetic analysis of the icefish (** ***Chaenocephalus aceratus*** **) cytoglobin compared to other species.**
(PDF)

Figure S2 **Alignment of the amino acid sequence of cytoglobin with other known fish cytoglobins.**
(PDF)

Table S1 **Top 30 commonly expressed sequences with associated BLAST matches in the three notothenioid fishes.**
(PDF)

Table S2 **Number of shared genes and number of enzymes (parentheses) involved in the metabolic pathway of each species.**
(PDF)

Table S3 **Analysis of species-specific statistics of metabolic pathway.**
(PDF)

Table S4 **List of enzymes in metabolic pathways identified in four notothenioid fishes.**
(PDF)

Table S5 **Putatively identified globin genes in the three notothenioid fishes.**
(PDF)
