# Supplementary material for: Transcriptomics and Comparative Analysis of Three Antarctic Notothenioid Fishes
Source: PLoS One. 2012 Aug 16;7(8):e43762. doi: 10.1371/journal.pone.0043762 (PMC3420891; doi:10.1371/journal.pone.0043762)
Supplement: Figure S1 — Phylogenetic analysis of the icefish ( Chaenocephalus aceratus ) cytoglobin compared to other species. (PDF) [file pone.0043762.s001.pdf]

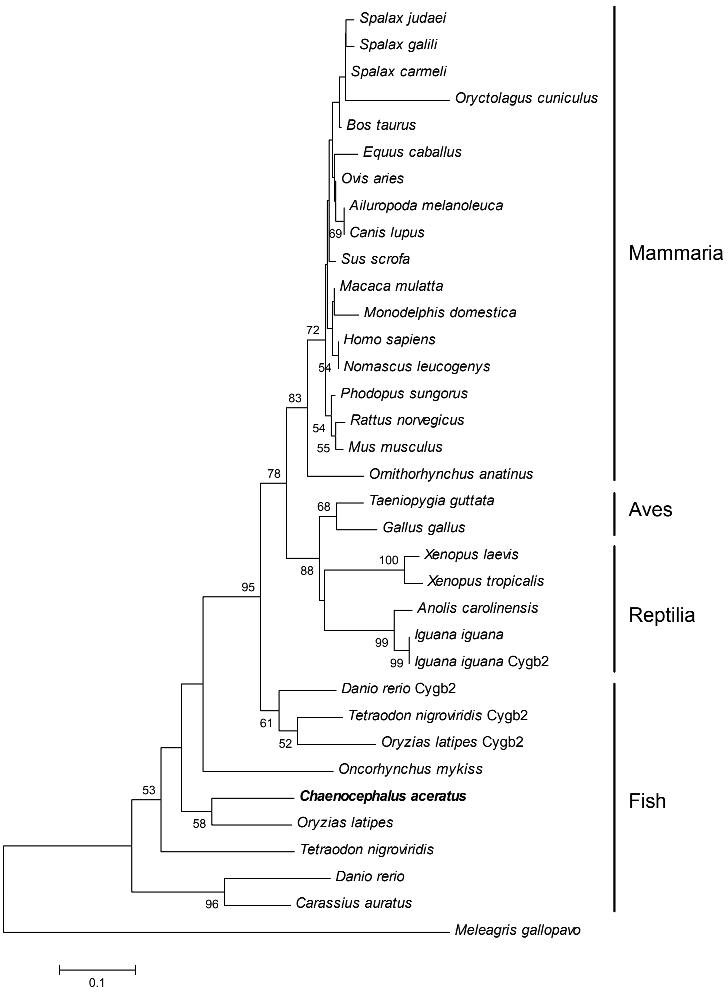


Figure S1. Phylogenetic analysis of the icefish (*Chaenocephalus aceratus*) cytoglobin compared to other species. Numbers at each branch indicate the percentage of the times a node was supported in 1000 bootstrap pseudoreplications by the neighbor-joining method in MEGA4. GenBank accession numbers for the sequences are as follows: *Oryzias latipes*, NP_001098237; *Tetraodon nigroviridis*, CAG25613; *Oncorhynchus mykiss*, NP_001117862; *Taeniopygia guttata*, XP_002195443; *Bos taurus*, NP_001193649; *Gallus gallus*, NP_001008789; *Ovis aries*, NP_001159664; *Sus scrofa*, XP_003131227; *Macaca mulatta*, XP_001104888; *Spalax carmeli*, CAM32305; *Spalax judaei*, CAL91964; *Ailuropoda melanoleuca*, XP_002919913; *Meleagris gallopavo*, XP_003211469; *Homo sapiens*, NP_599030; *Phodopus sungorus*, CAX11509; *Tetraodon nigroviridis* Cygb2, CAG25614; *Nomascus leucogenys*, XP_003279161; *Monodelphis domestica*, XP_001377108; *Rattus norvegicus*, NP_570100; *Spalax galili*, CAL91963; *Iguana iguana*, ABN71516; *Oryzias latipes* Cygb2, NP_001098238; *Mus musculus*, NP_084482; *Iguana iguana* Cygb2, ABN71517; *Canis lupus*, NP_001071055; *Xenopus laevis*, NP_001087751; *Xenopus tropicalis*, NP_001006870; *Anolis carolinensis*, XP_003217236; *Danio rerio*, NP_694484; *Danio rerio* Cygb2, XP_001923665; *Equus caballus*, XP_001491634; *Ornithorhynchus anatinus*, XP_001507856; *Carassius auratus*, CAP69823; *Oryctolagus cuniculus*, XP_002722974.
