# Supplementary material for: Transcriptomics and Comparative Analysis of Three Antarctic Notothenioid Fishes
Source: PLoS One. 2012 Aug 16;7(8):e43762. doi: 10.1371/journal.pone.0043762 (PMC3420891; doi:10.1371/journal.pone.0043762)
Supplement: Figure S2 — Alignment of the amino acid sequence of cytoglobin with other known fish cytoglobins. (PDF) [file pone.0043762.s002.pdf]

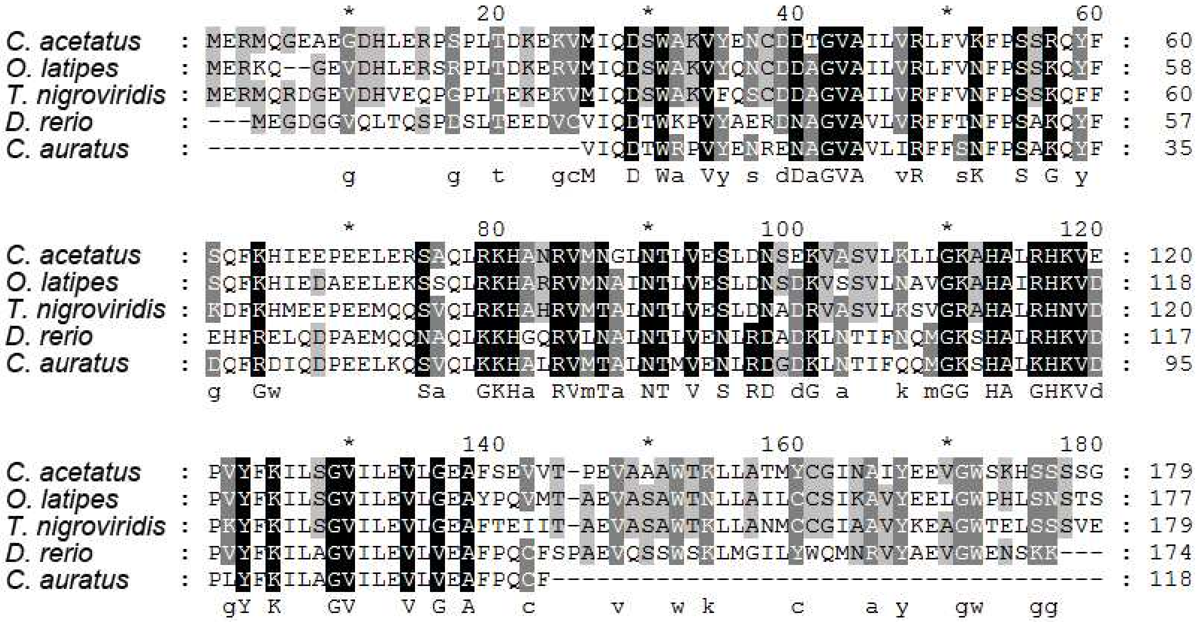

Figure S2. Alignment of the amino acid sequence of cytoglobin with other known fish cytoglobins. Sequences were aligned in MEGA4 using CLUSTALW with default settings, and refined manually. Identical amino acids in all sequences are highlighted in dark gray and conserved residues are highlighted in light gray. The GenBank accession numbers of the cytoglobin are as in Figure S1.
