# Supplementary material for: Transcriptomics and Comparative Analysis of Three Antarctic Notothenioid Fishes
Source: PLoS One. 2012 Aug 16;7(8):e43762. doi: 10.1371/journal.pone.0043762 (PMC3420891; doi:10.1371/journal.pone.0043762)
Supplement: Table S1 — Top 30 commonly expressed sequences with associated BLAST matches in the three notothenioid fishes. (PDF) [file pone.0043762.s003.pdf]

Table S1. Top 30 commonly expressed sequences with associated BLAST matches in the three notothenioid fishes

Contig ID	Length (bp)	No. of Reads	Sequence Description	E-Value	Biological process
Notothenia coriiceps					
**Liver**					
NCL contig01112	2889	6075	nebulin-related anchoring protein isoform 2	0	
NCL contig03200	3429	4804	synaptopodin 2	3.1E-77	
NCL contig01639	5275	4427	myomesin 185kda	0	musPAL contraction
NCL contig01281	2579	4179	reverse transcriptase-like protein	9.8E-41	
NCL contig01633	5799	4058	myomesin 185kda	0	musPAL contraction
NCL contig01380	2213	3463	transposase	2E-146	DNA-mediated transposition
NCL contig01110	5804	3321	myomesin 2	0	musPAL contraction
NCL contig03496	4483	2810	b-cell translocation gene anti-proliferative	1.4E-77	regulation of transcription
NCL contig01286	1103	2761	s100-a1	5.4E-20	
NCL contig01670	3895	2655	myomesin 1 isoform 1	0	musPAL contraction
NCL contig04433	1017	2183	musPAL eblind-like protein 1-like	1E-99	regulation of alternative nuPAL ear mRNA splicing
NCL contig01970	1400	2181	triosephosphate isomerase	5E-130	fatty acid biosynthetic process
NCL contig02983	3443	2125	ubiquitin specific peptidase 7	0	regulation of protein stability
NCL contig02213	2691	1886	calpain small subunit 1	3E-101	positive regulation of cell proliferation
NCL contig03886	999	1868	3 -5 exonuPAL ease with to crispr-associated protein	6.2E-30	DNA replication
NCL contig02010	3326	1626	hypothetical protein	2.1E-92	nuPAL acid binding
NCL contig02284	3326	1588	ahnak nuPAL eoprotein	6E-152	nervous system development
NCL contig03490	1260	1518	e3 ubiquitin-protein ligase march7	8.3E-07	
NCL contig03967	2642	1313	melanoma cell adhesion molecule	4.6E-56	
NCL contig02611	3711	1294	calpastatin	8.8E-70	transcription
NCL contig02341	583	1266	histone PAL uster h2bc-like	8.3E-43	nuPAL eosome assembly
NCL contig04364	1391	1198	family with sequence similarity 62 (c2 domain containing) member a	4.1E-74	
NCL contig03606	6171	1192	nfix protein	0	DNA replication
NCL contig03548	2793	1185	zinc finger protein-like	0	
NCL contig02041	1073	1121	viral a-type iNCL usion protein	2.1E-13	regulation of cellular process
NCL contig04436	927	1119	musPAL eblind-like 1 isoform 4	2.7E-96	regulation of alternative nuPAL ear mRNA splicing
NCL contig00533	4138	1104	harbinger transposase derived 1	5E-21	
NCL contig00684	2671	1087	Gag-Pol polyprotein	9E-109	
NCL contig00682	2649	1086	retrotransposon ty3-gypsy subPAL ass	2.5E-63	
NCL contig00685	2672	1085	retrotransposon-like family member (retr-1)-like	4E-114	macromolecule metabolic process

**Brain**					
NCB contig09189	1003	261	endonuPAL ease-reverse transcriptase	2.6E-47	RNA-dependent DNA replication
NCB contig06129	623	168	midkine	1.7E-42	cell migration
NCB contig09777	440	144	nadh dehydrogenase	1.8E-22	
NCB contig07911	786	133	splicing factor proline glutamine rich (polypyrimidine tract binding protein associated)	1.6E-24	RNA splicing
NCB contig08524	416	111	histone PAL uster h2bc-like	6.8E-43	nuPAL eosome assembly
NCB contig04308	872	93	reverse transcriptase-like protein	9.6E-37	RNA-dependent DNA replication
NCB contig04190	671	88	endonuPAL ease-reverse transcriptase	1.3E-09	RNA-dependent DNA replication
NCB contig05464	746	86	ribosomal protein l4	1.7E-94	translational elongation
NCB contig00238	1193	71	eukaryotic translation elongation factor 2	1E-177	translational elongation
NCB contig08443	1338	70	transposase	6E-144	DNA-mediated transposition
NCB contig07558	763	67	60s ribosomal protein l5	2E-107	translation
NCB contig09507	480	64	cytochrome c oxidase subunit vic	1.3E-28	
NCB contig05034	1339	62	eukaryotic translation initiation factor 4 2	0	regulation of translational initiation
NCB contig07044	1025	61	glutamine synthetase	5.1E-46	cell proliferation
NCB contig04691	841	54	proteasome (macropain) 26s non- 3	1E-118	regulation of protein catabolic process
NCB contig09703	1201	53	nuPAL ease harbi1-like	5.9E-23	
NCB contig02221	2457	52	kh domain rna signal transduction associated 1	3.1E-36	cell cyPAL e process
NCB contig08690	341	50	nuPAL ear nf-kappab activating protein	1.8E-22	
NCB contig08282	238	49	tubulin alpha-1c chain-like	9.9E-21	protein polymerization
NCB contig00606	801	47	gamma-secretase subunit pen-2	4.2E-49	positive regulation of catalytic activity
NCB contig05446	801	47	PAL audin 6	4.3E-53	calcium-independent cell-cell adhesion
NCB contig09369	515	46	high mobility group-t protein	1.7E-34	P:DNA metabolic process; F:DNA binding
NCB contig01954	1706	45	a chain structures of glycogen phosphorylase-inhibitor complexes and the implications for structure-based drug design	4E-103	glycogen catabolic process
NCB contig04962	996	45	fk506 binding protein 25kda	4E-106	protein folding
NCB contig07578	777	45	transcription elongation factor b polypeptide 1	7.3E-59	regulation of transcription from RNA polymerase II promoter
NCB contig01668	523	43	ribosomal protein s16	4.8E-75	rRNA processing
NCB contig05495	802	43	coagulation factor x	2.3E-31	
NCB contig09174	505	43	general transcription factor ii-i repeat domain-containing protein partial	3.3E-43	
NCB contig03177	972	42	upf0585 protein c16orf13 homolog	5.7E-92	
NCB contig01794	1447	39	dystonin	9.5E-51	axonogenesis
Pleuragramma antarctivum					
**Liver**					
PAL 1Contig167	1750	5499	14 kda apolipoprotein	1.3E-55	lipid transport
PAL 2Contig5	7900	4458	apolipoprotein b	0	lipid transport
PAL 3Contig8	2111	2822	betaine--homocysteine s-methyltransferase 1	0	protein amino acid methylation
PAL 1Contig445	2259	2120	fibrinogen gamma chain	2E-171	platelet activation
PAL 4Contig1	970	2064	mid1-interacting protein 1	6.6E-49	

PAL 2Contig10	1615	1896	apolipoprotein a-i	3.4E-81	lipid transport
PAL 1Contig917	2601	1825	fibrinogen beta chain	0	platelet activation
PAL 1Contig811	3666	1812	methionine adenosyltransferase alpha	0	auxin biosynthetic process
PAL 7Contig2	2016	1431	cytochrome c oxidase subunit ii	7E-107	transport
PAL 1Contig954	1342	1310	apolipoprotein c-ii	1.5E-18	lipid transport
PAL 6Contig7	5820	1195	fatty acid synthase	0	fatty acid biosynthetic process
PAL 8Contig2	2651	1149	s-adenosylhomocysteine hydrolase	0	response to nutrient
PAL 1Contig490	680	1123	apolipoprotein c-i	2E-07	lipoprotein metabolic process
PAL 11Contig2	2002	1086	cytochrome c oxidase subunit i	0	electron transport chain
PAL 9Contig4	2607	1034	alanine-glyoxylate aminotransferase	0	glyoxylate metabolic process
PAL 1Contig166	3024	941	serpina1 protein	6E-172	
PAL 13Contig2	951	925	type-iv antifreeze protein	5.1E-48	response to freezing
PAL 14Contig2	3071	854	acyl- desaturase	8E-151	fatty acid biosynthetic process
PAL 1Contig57	1708	828	apolipoprotein e	9E-105	lipid transport
PAL 10Contig2	1517	827	fructose-bisphosphate aldolase b	0	response to stress
PAL 1Contig1041	4723	823	inter-alpha inhibitor h2	0	hyaluronan metabolic process
PAL 15Contig3	2717	810	selenoprotein p precursor	8E-59	growth
PAL 17Contig1	966	764	cytochrome c oxidase subunit iii	3E-111	mitochondrial electron transport
PAL 21Contig2	2657	671	transferrin	0	iron ion transport
PAL 1Contig1014	2286	668	uridine phosphorylase 2	2E-158	nuPAL eotide catabolic process
PAL 5Contig5	1615	662	protein ambp precursor	3E-139	transport
PAL 18Contig2	2347	657	fetuin b	2E-116	
PAL 23Contig1	1991	639	elongation factor 1 alpha	0	translational elongation
PAL 2Contig11	835	636	apolipoprotein a-i	2.7E-36	lipid transport
PAL 1Contig919	4849	628	complement component c3	0	innate immune response
**Brain**					
PAB contig08212	215	1989	histone h2b 5-like	2.5E-12	nuPAL eosome assembly
PAB contig04362	487	695	cd81 antigen	2.9E-55	
PAB contig09320	126	656	histone h2b	6.8E-10	nuPAL eosome assembly
PAB contig02243	607	635	h g transactivated protein 2 isoform 1	0.00057	
PAB contig00011	1776	386	ubiquitin associated protein 2-like isoform 3	1E-164	
PAB contig08668	179	291	CR1-3 [Lycodichthys dearborni]	2.5E-14	
PAB contig00027	1541	218	influenza virus ns1a binding protein	3E-153	response to virus
PAB contig00052	1367	217	heterogeneous nuPAL ear ribonuPAL eoprotein	2.3E-06	RNA processing
PAB contig00800	808	217	ependymin-1 precursor	6.6E-91	cell-matrix adhesion
PAB contig00014	1734	201	cadherin h-cadherin	0	regulation of endocytosis
PAB contig00138	1136	200	protein piccolo	3.7E-18	synaptic vesiPAL exocytosis
PAB contig01956	630	184	reticulon 3	6.5E-78	
PAB contig00007	1850	181	contactin a	0	metabolic process
PAB contig06356	387	181	nadh dehydrogenase	1.9E-20	respiratory electron transport chain
PAB contig00012	1770	177	xk-related protein 5	1.2E-93	

PAB	contig01870	646	163	reverse transcriptase-like protein	2E-48	RNA-dependent DNA replication
PAB	contig00035	1496	161	kinesin heavy chain isoform 5c-like	6.3E-08	
PAB	contig00206	1065	161	spastic ataxia of charlevoix-saguenay	2.6E-96	protein folding
PAB	contig04056	498	161	protocadherin 2 alpha b 6	8.2E-32	homophilic cell adhesion
PAB	contig01422	699	138	seizure protein 6 homolog	1.3E-22	
PAB	contig01581	671	130	wsb1 protein	9.6E-49	
PAB	contig00001	2520	127	cadherin-related neuronal receptor c02	0	homophilic cell adhesion
PAB	contig08067	226	127	protein	2.2E-08	
PAB	contig07727	256	123	hypothetical protein DAPPUDRAFT_305258 [Daphnia pulex]	0.00044	
PAB	contig01939	636	119	upf0585 protein c16orf13 homolog	4.7E-47	
PAB	contig04226	491	109	enterin neuropeptide	1.3E-16	
PAB	contig07460	287	109	histone h2b 1 2-like	5.2E-24	nuPAL eosome assembly
PAB	contig00986	768	102	chk1 checkpoint-like protein	5.1E-29	protein amino acid phosphorylation
PAB	contig05316	442	101	histone-lysine n-methyltransferase nsd3	7.6E-49	cell growth
PAB	contig00281	1014	97	sra stem-loop-interacting rna-binding mitochondrial precursor	1E-39	regulation of transcription

Chaenocephalus aceratus

**Liver**					
CAL contig03951	1166	1094	cathepsin e	4E-124	proteolysis
CAL contig07229	1101	751	vitellogenin c	2E-113	lipid transport
CAL contig07523	3707	707	vitellogenin b	0	lipid transport
CAL contig04500	1007	644	peptidylprolyl isomerase b (cyPAL ophilin b)	1.1E-98	protein folding
CAL contig07707	628	602	whey acidic protein precursor	1.1E-36	
CAL contig03571	4012	582	apolipoprotein b	0	system development
CAL contig03904	1377	542	vitellogenin	0	lipid transport
CAL contig04203	632	505	14 kda apolipoprotein	1.2E-41	
CAL contig07680	152	430	tributyltin binding protein type 2	1.9E-11	response to toxin
CAL contig04426	1145	409	aquaporin 12	1E-103	
CAL contig03917	1698	332	hypothetical protein Pcal_1868 [Pyrobaculum calidifontis JCM 11548]	2.2E-27	
CAL contig04343	913	329	isoform cra_a	1E-127	response to organic cyPAL ic substance
CAL contig07820	367	315	nattectin precursor	3.1E-35	induction of bacterial agglutination
CAL contig03605	490	300	chk1 checkpoint-like protein	1E-30	
CAL contig03986	549	288	complement component q subcomponent-like 4 like	1.1E-22	defense response to bacterium
CAL contig03805	848	287	choriogenin h	6.8E-56	
CAL contig00296	1474	256	serpina1 protein	1E-128	
CAL contig02144	1233	245	ribosomal protein l4	5E-174	translational elongation
CAL contig06335	1099	228	cell death activator cide-b	3.4E-75	apoptosis
CAL contig00002	660	215	CDH-1D	9.1E-64	
CAL contig07979	359	214	choriogenin h	1.6E-34	
CAL contig08352	214	212	tributyltin binding protein type 2	2E-10	response to toxin
CAL contig04235	1042	201	glutathione peroxidase 3	5E-65	oxidation reduction
CAL contig04346	949	199	beta-2-microglobulin precursor	7.1E-38	immune response

CAL contig07731	465	195	choriogenin l	4.9E-60	
CAL contig00144	1625	189	reticulon 1a	1.2E-80	neuron differentiation
CAL contig04005	1125	189	kininogen 1	1.6E-83	regulation of biological quality
CAL contig04525	1143	189	60s acidic ribosomal protein p0	2E-135	embryonic development
CAL contig00444	2871	172	elongation factor 2	0	response to chemical stimulus
CAL contig04326	1437	161	gdp dissociation inhibitor 2	0	vesiPAL e-mediated transport
**Brain**					
CAB contig04503	1140	578	protein s100-b	8.5E-24	cell proliferation
CAB contig04534	694	462	ependymin-1 precursor	1.1E-38	cell-matrix adhesion
CAB contig04086	1747	375	putative senescence-associated protein	6.8E-55	
CAB contig00003	628	346	CDH-1D	8.1E-64	
CAB contig09365	527	341	chk1 checkpoint homolog	1.3E-19	auxin biosynthetic process
CAB contig05348	1660	180	novel protein vertebrate piccolo (presynaptic cytomatrix protein)	3.3E-20	
CAB contig09236	1688	155	cytochrome c oxidase subunit ii	6E-104	respiratory electron transport chain
CAB contig04832	2951	146	nadh dehydrogenase subunit 4	0	mitochondrial electron transport
CAB contig00031	1631	133	cytochrome c oxidase subunit i	0	respiratory electron transport chain
CAB contig00593	1105	132	male-specific protein	5.7E-78	
CAB contig04329	2291	117	endonuPAL ease-reverse transcriptase	1.7E-72	RNA-dependent DNA replication
CAB contig00033	1438	104	creatine testis isozyme	0	
CAB contig00050	1254	102	ribosomal protein l4	5E-174	translational elongation
CAB contig00098	1269	102	apolipoprotein a-i	9.4E-75	lipid transport
CAB contig09783	239	90	reverse trancriptase	1.1E-11	RNA-dependent DNA replication
CAB contig03761	604	87	transcription elongation factor b polypeptide 1	4.2E-59	ubiquitin-dependent protein catabolic process
CAB contig06436	968	84	beta-2-microglobulin precursor	1.6E-37	immune response
CAB contig00437	1462	77	apolipoprotein e	9E-107	lipid transport
CAB contig09116	774	73	pleiotrophic factor-alpha-2 precursor	1.2E-42	cell migration
CAB contig04978	2268	72	novel protein vertebrate cadherin h-cadherin	2E-121	regulation of endocytosis
CAB contig09534	307	71	histone h2b	6.9E-43	nuPAL eosome assembly
CAB contig04413	1707	70	alpha 1b	0	microtubule-based movement
CAB contig05585	1018	68	influenza virus ns1a-binding protein homolog a	1.7E-89	response to virus
CAB contig00713	1083	67	60s ribosomal protein l5	5E-156	translation
CAB contig05313	1075	65	phospholemman precursor	1.2E-37	ion transport
CAB contig08999	359	65	nadh dehydrogenase	1.2E-23	
CAB contig00032	1148	61	cytochrome b	0	respiratory electron transport chain
CAB contig04906	1266	60	beta-actin	0	auxin biosynthetic process
CAB contig04723	1061	58	scavenger receptor PAL ass member 2	7.9E-90	cell adhesion
CAB contig00103	1973	56	visinin-like 1	2E-106	calcium ion binding
