# Supplementary material for: Transcriptomics and Comparative Analysis of Three Antarctic Notothenioid Fishes
Source: PLoS One. 2012 Aug 16;7(8):e43762. doi: 10.1371/journal.pone.0043762 (PMC3420891; doi:10.1371/journal.pone.0043762)
Supplement: Table S2 — Number of shared genes and number of enzymes (parentheses) involved in the metabolic pathway of each species. (PDF) [file pone.0043762.s004.pdf]

Table S2. Number of shared genes, and number of enzymes (parentheses) involved in the metabolic pathway between each species

	Brain					Liver				
	No. of genes	*N. coriiceps*	*C. aceratus*	*P. antarcticum*	*D. mawsoni*	No. of genes	*N. coriiceps*	*C. aceratus*	*P. antarcticum*	*D. mawsoni*
No. of enzymes involved metabolic pathway		(290)	(236)	(240)	(308)		(830)	(444)	(706)	(281)
N. coriiceps	2173	-	(105)	(63)	(78)	6793	-	(275)	(342)	(107)
C. aceratus	2318	891	-	(76)	(67)	2739	1822	-	(264)	(89)
P. antarcticum	2603	514	711	-	(48)	3806	1909	1338	-	(123)
D. mawsoni	2194	529	523	355	-	2208	756	588	690	-
