# Supplementary material for: Transcriptomics and Comparative Analysis of Three Antarctic Notothenioid Fishes
Source: PLoS One. 2012 Aug 16;7(8):e43762. doi: 10.1371/journal.pone.0043762 (PMC3420891; doi:10.1371/journal.pone.0043762)
Supplement: Table S3 — Analysis of species-specific statistics of metabolic pathway. (PDF) [file pone.0043762.s005.pdf]

Table S3. Analysis of specie-specifics statistics of metabolic pathway

	Liver				Brain								
**mfn_pathway**	**NC***	**PA***	**CA***	**DM***	**NC**	**PA**	**CA**	**DM**	**Zebrafish**	**Stickleback**	**Medaka**	**Takifugu**	**Tetraodon**
1- and 2-Methylnaphthalene degradation	1	1							3	2	3	3	2
3-Chloroacrylic acid degradation	1	1	2	2	1		3	5	2	1	2	2	1
3-oxo-10R-octadecatrienoate beta-oxidation	5	8	3	6	5				4	4	4	4	4
Alanine and Aspartate Metabolism	8	11	9	3	4		2	7	7	9	7	10	9
Alkaloid biosynthesis II	4	2	3	1		2	1	1	3	3	2	3	3
Aminosugars metabolism	23	15	11	3	5	2	3	4	19	18	18	19	19
Androgen and estrogen biosynthesis and metabolism	36	31	17	14	12	10	7	12	12	18	19	16	18
Arachidonic acid metabolism	24	13	12	12	11	3	8	8	19	19	21	17	19
Arginine and Proline Metabolism	13	12	5	3	2	3	2	2	11	12	11	12	11
Ascorbate (Vitamin C) and Aldarate Metabolism									1	1	1	1	1
Aspartate and asparagine metabolism	24	18	16	12	7	3	8	10	26	36	33	39	33
Atrazine degradation									0	0	1	1	1
Benzoate degradation via CoA ligation	4	4	3	3	2	1			3	4	3	3	3
Beta-Alanine metabolism	7	9	6	1	2	1	4		6	6	6	6	6
Bile acid biosynthesis	5	12	6	3	1	1	1	2	14	14	16	16	15
Biopterin metabolism	6	3	2	1	5			1	5	5	4	4	3
Blood Group Biosynthesis	1	3				5	3	1	2	3	2	3	2
Butanoate metabolism	2	5	1	2	1	1	2	4	11	11	12	12	11
C21-steroid hormone biosynthesis and metabolism			1		1				10	15	16	17	15
C5-Branched dibasic acid metabolism	1								2	2	2	1	2
Carbon fixation	4	3		3	2	3	5	5	3	3	3	3	3
Carnitine shuttle	3	3	2			1		2	0	1	1	1	1
Chondroitin sulfate degradation	6	1	2		1				1	2	2	2	3
CoA Catabolism									0	0	0	0	0
D4&E4-neuroprostanes formation									0	0	0	0	0
De novo fatty acid biosynthesis	7	10	3	1	3	3	1	4	8	11	10	9	9
Dimethyl-branched-chain fatty acid mitochondrial beta-oxidation									4	4	4	4	4
Di-unsaturated fatty acid beta-oxidation	6	13	4		3	3	5		6	6	6	6	6
Dynorphin metabolism									2	2	2	1	1
Electron transport chain	57	54	43	16	45	31	34	14	5	5	5	5	5
Fatty acid activation									2	2	2	2	2
Fatty Acid Metabolism	5	5	2	2		1	2	1	11	11	11	11	10
Fatty acid oxidation									2	2	2	2	2
Fatty acid oxidation, peroxisome	1	1	1	1					1	1	1	1	1
Fructose and mannose metabolism	13	16	11	4	4	2	4	5	13	13	13	12	13
Galactose metabolism	4	5	1	2	2	1	2	5	9	10	10	10	10
Geraniol degradation									1	1	1	1	1
Glutamate metabolism	2	1				1		2	10	11	11	12	10
Glutathione Metabolism	2				1				3	6	5	6	6
Glycerolipid metabolism									1	1	1	1	1
Glycerophospholipid metabolism	38	28	15	8	6	16	9	11	33	43	42	43	43
Glycine, serine, alanine and threonine metabolism	33	32	25	21	13	6	9	22	21	30	29	29	25
Glycolysis and Gluconeogenesis	18	18	10	5	7	3	4	13	28	28	27	28	26
Glycosphingolipid biosynthesis - ganglioseries	9	11	5	3	2	2	2	4	7	13	14	15	16
Glycosphingolipid biosynthesis -	1	1	3	1			1	4	5	5	5	7	6

globoseries													
Glycosphingolipid biosynthesis - lactoseries									2	3	3	2	1
Glycosphingolipid biosynthesis - neolactoseries	1			1					4	8	8	7	6
Glycosphingolipid metabolism	9	9	4	5	2	4	3		16	21	21	22	23
Glycosylphosphatidylinositol(GPI)-anchor biosynthesis									1	1	1	1	1
Glyoxylate and Dicarboxylate Metabolism		1							2	2	2	2	2
Heparan sulfate biosynthesis	2	1							1	1	1	1	1
Heparan sulfate degradation	2					1			1	3	3	3	4
Hexose phosphorylation	2	2	1		1		1		3	4	5	4	5
Histidine metabolism	9	10	6	7	3	2		3	9	10	11	10	11
Hyaluronan Metabolism									2	3	3	3	3
Keratan sulfate biosynthesis	1			1		1	1		2	2	1	3	2
Keratan sulfate degradation	1	2				1			7	7	7	7	7
Leukotriene metabolism	1								15	14	14	14	13
Limonene and pinene degradation									4	4	4	4	4
Linoleate metabolism		1			1			1	7	8	8	7	7
Lipoate metabolism	5	5	5	2	3	1		2	4	3	2	3	2
Lysine metabolism	94	79	41	26	25	49	23	30	14	15	14	16	13
Methionine and cysteine metabolism	24	25	18	16	7	3	8	15	26	32	31	30	32
Mono-unsaturated fatty acid beta-oxidation									4	4	4	4	4
N-Glycan biosynthesis	26	16	18	6	11	8	7	11	8	11	11	11	12
N-Glycan Degradation		1			1				7	7	7	7	7
Nitrogen metabolism	3	1		2	1	1	2	1	3	3	3	3	3
Nucleotide Sugar Metabolism	1								2	1	2	1	2
O-Glycan biosynthesis	2	1				1	1	1	2	3	3	3	4
Omega-3 fatty acid metabolism									7	7	7	6	7
Omega-6 fatty acid metabolism	1								9	9	9	9	9
Parathio degradation									2	1	2	1	1
Pentose and Glucuronate Interconversions		1							1	1	2	1	0
Pentose phosphate pathway	8	12	10	2	4	3	3	5	10	13	12	12	13
Phosphatidylinositol phosphate metabolism	30	11	5	3	3	13	6	12	11	21	22	20	21
Phytanic acid peroxisomal oxidation	1	2	1					1	5	5	5	5	5
Polyunsaturated fatty acid biosynthesis	1	2	1						4	5	5	4	4
Porphyrin metabolism	8	2	2	2	2		2	1	10	9	10	9	10
Propanoate metabolism	2	1				1			8	8	8	8	8
Prostaglandin formation from arachidonate	10	6	5	1	1	4	2	6	8	8	10	8	10
Prostaglandin formation from dihomo gama-linoleic acid									3	2	2	2	3
Proteoglycan biosynthesis	4	1		1				3	4	15	14	15	15
Purine metabolism	70	36	32	27	25	12	20	17	33	41	38	41	42
Putative anti-Inflammatory metabolites formation from EPA									5	6	6	6	5
Pyrimidine metabolism	15	14	5	5	4	2	2	5	30	32	31	30	30
Pyruvate Metabolism	1	2			1				4	5	5	6	5
R Group Synthesis									0	0	0	0	0
ROS Detoxification	2	2	2	2	3	1	2	1	2	2	2	2	2
Saturated fatty acids beta-oxidation		1							9	9	10	10	10
Selenoamino acid metabolism	1	2	1	1				1	8	9	9	9	10
Sialic acid metabolism									12	14	12	15	14
Sphingolipid metabolism									1	1	1	2	1
Squalene and cholesterol biosynthesis	9	14	7	4	6	2	2	4	13	14	14	14	13

Starch and Sucrose Metabolism	9	4		1	1		1		5	6	8	8	8
TCA cycle	16	15	9	5	7	9	8	9	13	14	15	14	15
Trihydroxycoprostanoyl-CoA beta-oxidation									4	4	4	4	4
Tryptophan metabolism	13	13	11	2	6	7	4	2	18	21	22	23	23
Tyrosine metabolism	9	11	8	3	4	1	4	4	31	37	36	35	34
Ubiquinone Biosynthesis				1					1	1	0	1	1
Urea cycle/amino group metabolism	8	2	3		2		1	2	20	25	24	24	25
Valine, leucine and isoleucine degradation	13	14	6	5	7	2	3	6	21	21	22	22	22
Vitamin A (retinol) metabolism	2	5	2	2		1	1	2	6	6	6	7	6
Vitamin B1 (thiamin) metabolism	13	9	5	6	4	2	2	7	5	6	5	4	4
Vitamin B12 (cyanocobalamin) metabolism	1				1				0	1	0	1	1
Vitamin B2 (riboflavin) metabolism	5	4	5	1	2	2	3	2	3	3	4	3	4
Vitamin B3 (nicotinate and nicotinamide) metabolism	8	6	2			1	1	3	11	9	11	12	11
Vitamin B5 - CoA biosynthesis from pantothenate	3	3	2	1	1		1	1	4	3	3	3	3
Vitamin B6 (pyridoxine) metabolism	1			1				1	2	1	0	1	1
Vitamin B9 (folate) metabolism	9	7	4	6	3				9	12	12	8	11
Vitamin D									0	0	0	0	0
Vitamin D3 (cholecalciferol) metabolism									0	1	1	2	1
Vitamin E metabolism									7	6	6	6	6
Vitamin H (biotin) metabolism									2	2	1	1	0
Vitamin K metabolism									0	1	0	1	1
Xenobiotics metabolism									7	6	8	8	6
**Total**	**830**	**706**	**445**	**281**	**290**	**240**	**236**	**308**	**850**	**987**	**983**	**991**	**976**

* NC, *N. coriiceps*; PA, *P. antarcticum*; CA, *C. aceratus*; DM, *D. mawsoni.*
