# Supplementary material for: Transcriptomics and Comparative Analysis of Three Antarctic Notothenioid Fishes
Source: PLoS One. 2012 Aug 16;7(8):e43762. doi: 10.1371/journal.pone.0043762 (PMC3420891; doi:10.1371/journal.pone.0043762)
Supplement: Table S4 — List of enzymes in metabolic pathways identified in four notothenioid fishes. (PDF) [file pone.0043762.s006.pdf]

Table S4. List of enzymes in metabolic pathways identified in four notothenioid fishes

| | | liver | | | | brain | | | |
|---|---|---|---|---|---|---|---|---|---|
| Sequence Description | No. contigs | NC* | PA* | CA* | DM* | NC | PA | CA | DM |
| zona pellucida sperm-binding protein 3-like | 490 | | | | o | | | | |
| apolipoprotein a-i | 425 | | o | o | o | o | o | o | o |
| novel protein | 350 | o | o | o | o | o | o | o | o |
| orf2-encoded protein | 339 | o | o | o | o | o | o | o | o |
| reverse transcriptase-like protein | 181 | o | o | o | o | o | o | o | o |
| complement component c3 | 136 | o | o | o | o | | | | o |
| apolipoprotein a-i precursor | 124 | | | | o | | | | o |
| fibrinogen beta chain precursor | 121 | | o | | o | | | | o |
| nadh dehydrogenase | 118 | o | o | o | o | o | o | o | o |
| cathepsin z precursor | 114 | | o | | o | o | | | |
| 14 kda apolipoprotein | 110 | | o | o | o | o | o | o | o |
| retrotransposable element tf2 155 kda protein type 1-like | 109 | o | | o | o | o | o | o | |
| reverse transcriptase ribonuclease h methyltransferase-like | 96 | o | | o | | o | | o | |
| ependymin-1 precursor | 95 | | | | o | o | o | o | o |
| member ras oncogene family | 95 | o | o | o | o | o | o | o | o |
| apolipoprotein e | 88 | o | o | | o | | | o | o |
| male-specific protein | 84 | o | o | o | o | o | o | o | o |
| retrotransposon-like family member (retr-1)-like | 82 | o | | o | o | o | o | o | |
| reverse transcriptase | 76 | o | o | o | o | o | o | o | o |
| creatine testis isozyme | 68 | | | o | o | | o | o | o |
| choriogenin l | 66 | | o | o | o | | | | o |
| gag-pol poly | 63 | o | | o | | o | | o | |
| 39s ribosomal protein mitochondrial precursor | 62 | o | o | o | o | o | o | o | o |
| general transcription factor ii-i repeat domain-containing protein 2-like | 62 | o | o | o | o | o | o | o | o |
| endonuclease-reverse transcriptase | 59 | o | o | o | o | o | o | o | |
| novel protein bloodthirsty | 59 | o | o | o | o | o | | | o |
| protein s100-b-like | 59 | | | | o | | o | | o |
| fish virus induced trim protein | 56 | o | o | o | | o | o | o | |
| af442732_2lambda-recombinase-like protein | 55 | o | | o | o | o | o | o | o |
| complement c1q-like protein 4 precursor | 55 | o | o | o | o | | o | | o |
| glyceraldehyde-3-phosphate dehydrogenase | 55 | o | o | o | o | o | o | o | o |
| multiple banded antigen | 54 | | | | o | | | | |
| uncharacterized protein | 54 | | o | | o | | o | | o |
| family with sequence similarity member a | 53 | o | o | o | o | o | o | o | o |
| iron-sulfur cluster assembly enzyme mitochondrial precursor | 48 | o | o | o | o | o | | o | o |
| conserved plasmodium protein | 47 | | | | o | | | | o |
| f-type lectin | 47 | | o | o | o | | | | |
| ubiquitin c | 47 | | | | o | | | o | o |
| coatomer subunit epsilon | 46 | o | o | o | o | o | o | o | o |
| gag-pol polyprotein | 46 | o | o | o | o | o | o | o | |
| 60s ribosomal protein l5 | 44 | o | o | o | o | o | o | o | o |
| transferrin | 44 | o | o | o | o | o | | o | o |
| pol-like protein | 43 | o | | o | o | o | o | o | |
| polyprotein-like | 42 | o | o | o | o | o | o | o | o |
| zona pellucida glycoprotein | 42 | o | | o | o | o | | | |
| adp atp translocase 2 | 41 | o | o | o | o | o | o | o | o |
| atp synthase subunit mitochondrial precursor | 41 | o | o | o | o | o | o | o | o |
| choriogenin h | 41 | o | | o | o | | | | |
| fibrinogen gamma chain-like | 40 | | | | o | | | | |
| gap-pol poly | 40 | o | | o | | o | o | | |

| | | | | | | | | | |
|---|---|---|---|---|---|---|---|---|---|
| guanine nucleotide-binding protein subunit beta-2-like 1 | 40 | o | o | o | o | o | | o | o |
| line-1 reverse transcriptase homolog | 40 | o | o | o | | o | o | o | |
| myosin heavy chain | 40 | o | o | | o | | o | o | |
| tubulin beta-2c chain | 40 | | o | o | o | | o | o | o |
| beta-actin | 39 | | o | o | o | | o | o | o |
| fibrinogen alpha chain | 39 | | o | o | o | | o | | o |
| elongation factor 2 | 38 | o | o | o | o | o | o | o | o |
| lactose-binding lectin l-2-like | 38 | | | | o | | | | |
| mannose-binding lectin-associated serine protease-3b | 38 | | o | | o | | | | |
| myomesin 185kda | 38 | o | | | | | | | |
| family with sequence similarity member b | 37 | o | o | o | | o | o | o | o |
| calmodulin | 36 | o | o | o | o | | | o | o |
| na+ k+ alpha 1 polypeptide | 36 | o | | | | o | o | o | o |
| glutamine synthetase | 35 | o | o | o | o | o | o | o | o |
| warm temperature acclimation protein 65 kda-2 | 35 | | o | | o | | | | o |
| atp synthase subunit mitochondrial | 34 | o | o | o | o | o | o | o | o |
| bile salt-activated lipase | 34 | | o | | o | | | | o |
| serpina1 protein | 34 | | o | o | o | | | o | |
| translationally-controlled tumor protein | 34 | o | o | o | o | o | | o | o |
| phospholipid hydroperoxide glutathione peroxidase | 33 | o | | o | o | o | | | o |
| zinc finger protein | 33 | o | o | o | o | o | o | o | |
| apolipoprotein b | 32 | | o | o | o | | o | | |
| endonuclease reverse transcriptase | 32 | o | o | o | o | o | o | o | o |
| ribosyldihydronicotinamide dehydrogenase | 32 | | | | o | | | | |
| antihemorragic factor chlp-b | 31 | | | | o | | | | o |
| cytochrome c oxidase subunit mitochondrial precursor | 31 | o | o | o | o | o | o | o | o |
| middle subunit | 31 | o | o | o | o | o | | o | o |
| stathmin | 31 | o | | | o | | | o | o |
| transposase | 31 | o | | o | o | o | o | | |
| enzymatic poly | 30 | | | | o | | o | | |
| fam111a protein | 30 | o | | | | | | | |
| heat shock protein 90 | 30 | o | o | o | o | o | o | o | o |
| egg envelope protein | 29 | | | | o | | | | |
| ribosomal protein l4 | 29 | o | o | o | o | o | o | o | o |
| type alpha 1 | 29 | o | | o | | | | o | |
| 60s ribosomal protein l7 | 28 | o | o | o | o | o | o | o | o |
| atp synthase lipid-binding mitochondrial precursor | 28 | o | o | o | o | o | o | o | o |
| copia-type -like | 28 | o | | o | | o | | o | |
| histone | 28 | o | o | o | o | o | o | o | o |
| mgc86501 protein | 28 | | | | o | | | | |
| serpin peptidase clade a (alpha-1 antitrypsin) member 7 | 28 | | o | | o | | | | |
| af442732_1 gag protein | 27 | o | | o | o | o | | o | o |
| cofilin-2 | 27 | o | o | o | o | | o | o | o |
| leucine-rich repeat neuronal protein 1-like | 27 | | | | | | | | o |
| uba and wwe domain containing 1 | 26 | o | o | o | o | o | o | o | |
| atp synthase f0 subunit 6 | 25 | | | | o | o | o | | o |
| clusterin precursor | 25 | | | | o | | | | o |
| elongation factor 1 alpha | 25 | | o | | o | | o | | o |
| heat shock protein 70 | 25 | o | o | o | o | o | | | o |
| lipocalin precursor | 25 | o | o | o | | o | o | o | o |
| nlr pyrin domain containing 1-like | 25 | o | | o | | o | | o | |
| ornithine decarboxylase antizyme 1 | 25 | o | o | o | o | o | o | o | o |
| superoxide dismutase | 25 | o | o | o | o | o | o | o | |

| | | | | | | | | | |
|---|---|---|---|---|---|---|---|---|---|
| AF503912_1polyprotein [Danio rerio] | 24 | o | o | o | | | o | o | |
| calpastatin | 24 | o | | | | | | | |
| dynein light chain cytoplasmic | 24 | o | o | o | o | o | o | o | o |
| heat shock protein 90 beta | 24 | | | o | o | o | o | o | o |
| nattectin precursor | 24 | o | o | o | | | | | |
| zinc finger | 24 | o | | o | o | o | | o | |
| 4-hydroxyphenylpyruvate dioxygenase | 23 | | o | o | o | | | | |
| death-associated 1-a | 23 | o | o | o | o | o | | o | o |
| h-2 class ii histocompatibility antigen gamma chain | 23 | o | o | o | o | o | o | o | o |
| integral membrane protein 2b | 23 | o | o | o | o | o | o | o | o |
| poly a binding cytoplasmic 1 b | 23 | o | o | | o | | o | | o |
| prostaglandin e synthase 3 | 23 | o | o | o | o | | | o | o |
| protein tyrosine receptor d | 23 | o | o | | | o | o | o | |
| ribosomal protein l6 | 23 | o | o | o | o | o | | o | o |
| basic transcription factor 3 | 22 | o | o | o | o | o | o | o | o |
| cd9 antigen | 22 | o | o | o | o | o | o | o | o |
| epididymal secretory protein e1 precursor | 22 | o | o | o | o | o | | o | o |
| fructose-bisphosphate aldolase c | 22 | | | | | o | o | o | o |
| reticulon 1 | 22 | | | | | | o | o | o |
| zinc mym domain containing 1 | 22 | o | | o | | o | | o | |
| 60s acidic ribosomal protein p0 | 21 | o | o | o | o | o | | o | o |
| beta tubulin | 21 | | | | o | | | | o |
| complement c4 | 21 | o | o | o | o | | o | | |
| elongation factor 1-delta | 21 | o | o | o | o | o | o | o | o |
| fetuin b | 21 | | o | o | o | | | | |
| glycogen muscle form | 21 | o | o | | | | o | o | o |
| isocitrate dehydrogenase | 21 | o | o | o | o | o | o | o | o |
| leucine-rich repeat neuronal protein partial | 21 | | | | | | | | o |
| mid1-interacting protein 1 | 21 | o | o | o | o | | o | o | o |
| myosin binding protein slow type | 21 | o | | | | | | | |
| p2x purinoceptor 7 | 21 | o | | o | | o | | o | |
| phosphoglycerate mutase 1 | 21 | o | o | o | o | o | o | o | o |
| ret finger | 21 | o | | o | | | | | |
| triosephosphate isomerase | 21 | o | o | o | | o | | o | o |
| ubiquitin-conjugating enzyme e2 d2 | 21 | o | o | o | o | | o | o | o |
| 40s ribosomal protein s3a | 20 | o | o | o | o | | o | o | o |
| c1q-like protein | 20 | | o | | | | | | |
| cellular nucleic acid-binding protein | 20 | o | o | o | o | | | o | o |
| complement factor h-like | 20 | | | | o | | | | |
| fatty acid-binding heart | 20 | o | o | o | o | o | o | o | o |
| gtpase imap family member 7 | 20 | o | | o | | o | | o | |
| heat shock cognate 70 | 20 | o | o | o | o | o | | o | o |
| myosin light polypeptide 6 | 20 | o | o | o | o | o | o | o | o |
| nadh dehydrogenase iron-sulfur protein mitochondrial precursor | 20 | o | o | o | o | o | o | o | o |
| pol polyprotein | 20 | o | | o | | o | | o | |
| protein ambp precursor | 20 | | o | o | o | | | | o |
| sumo-conjugating enzyme ubc9 | 20 | o | o | o | o | o | o | o | |
| uridine phosphorylase 2 | 20 | | o | o | o | | | | o |
| alcohol dehydrogenase class-3 | 19 | o | o | o | | o | o | o | o |
| apolipoprotein c-i precursor | 19 | | | | o | | | | o |
| atp-dependent rna helicase ddx39 | 19 | o | o | o | o | o | o | | o |
| beta-synuclein | 19 | | | | | | | | o |
| cathepsin d | 19 | o | o | o | o | o | | o | o |
| cold-inducible rna-binding protein | 19 | o | o | o | o | | | | o |
| egg envelope component zpax | 19 | | | o | o | | | | |
| elongation factor 1-alpha | 19 | o | | o | o | o | | o | o |
| furry homolog | 19 | o | o | | | | o | | |

| | | | | | | | | | |
|---|---|---|---|---|---|---|---|---|---|
| myosin regulatory light chain smooth muscle isoform | 19 | o | o | o | o | o | o | o | o |
| proactivator polypeptide precursor | 19 | o | o | o | o | o | o | o | o |
| profilin-2 | 19 | o | o | o | o | o | o | o | |
| t-complex protein 1 subunit epsilon | 19 | | | | o | | | o | o |
| transposable element tcb1 transposase | 19 | o | | o | o | o | | o | |
| alpha tubulin | 18 | | | | o | o | | | o |
| arginine vasotocin preprohormone | 18 | | | | | | o | | o |
| coatomer subunit zeta-1 | 18 | o | | o | o | o | | o | o |
| cytochrome b-c1 complex subunit mitochondrial precursor | 18 | o | o | o | | o | o | o | o |
| cytochrome family subfamily polypeptide 1 | 18 | o | o | o | | o | o | | |
| eno1 protein | 18 | | | | o | | | | o |
| fibronectin 1b | 18 | o | o | o | o | o | o | o | |
| glutathione s-transferase | 18 | o | o | o | o | o | | o | o |
| nucleic acid binding protein | 18 | o | o | o | o | o | o | o | |
| pentraxin | 18 | o | o | o | o | o | | o | o |
| probable glutamate receptor-like | 18 | | | | | | o | | o |
| protein z-dependent protease inhibitor-like | 18 | | | | o | | | | o |
| titin | 18 | o | | | o | | o | | |
| tmsb4x protein | 18 | o | o | | | o | | o | o |
| 40s ribosomal protein s4 | 17 | o | o | o | o | o | o | o | o |
| ankyrin node of ranvier (ankyrin g) | 17 | o | | | | o | o | o | |
| cerebellin-1 precursor | 17 | | | | | | | o | o |
| cytochrome c | 17 | o | o | o | o | o | | | o |
| electron transfer flavoprotein subunit mitochondrial precursor | 17 | o | o | o | o | o | o | o | o |
| glutathione s-transferase kappa 1 | 17 | o | o | o | | o | o | | |
| heavy subunit | 17 | o | o | o | o | o | o | o | o |
| high mobility group protein b2 | 17 | o | o | o | o | | o | | o |
| microsomal glutathione s-transferase 3 | 17 | o | o | o | | o | | o | o |
| nadh dehydrogenase 1 beta subcomplex subunit mitochondrial precursor | 17 | o | o | o | | o | o | o | |
| peptidyl-prolyl cis-trans isomerase h | 17 | o | | o | o | o | | | o |
| peroxiredoxin 6 | 17 | o | o | o | o | o | | o | o |
| scan domain containing 3 | 17 | o | o | o | | o | o | | |
| tubulin alpha-1c chain- partial | 17 | | | | o | | | | o |
| type 1 collagen alpha 2 | 17 | o | o | o | | o | | o | |
| zgc:161969 protein | 17 | o | o | o | | | o | o | o |
| 40s ribosomal protein sa | 16 | | | | o | | o | o | o |
| 60s ribosomal protein l19 | 16 | o | o | | o | o | o | o | o |
| 60s ribosomal protein l35 | 16 | o | o | o | o | o | o | o | |
| alanine aminotransferase 2-like | 16 | o | o | o | o | o | o | o | o |
| chromobox protein homolog 3 | 16 | o | o | o | o | o | o | o | o |
| cytoplasmic dynein 1 heavy chain 1 | 16 | o | | | | o | o | o | o |
| eukaryotic translation initiation factor 4 2 | 16 | o | o | o | | o | o | o | o |
| fatty acid-binding brain | 16 | o | | | | o | o | o | o |
| fgg protein | 16 | | o | | o | | | | |
| fibrinogen gamma chain precursor | 16 | | | | o | | | | o |
| fk506-binding protein 1a | 16 | o | | o | o | o | | o | o |
| fructose-bisphosphate aldolase b | 16 | | o | o | o | | | | |
| immunoglobulin mu heavy chain | 16 | o | | | | | | | |
| lactate dehydrogenase-a | 16 | o | o | o | o | | | o | o |
| mhc class ii antigen beta chain | 16 | o | | o | | | o | | o |
| nascent polypeptide-associated complex subunit alpha | 16 | o | o | o | o | o | o | o | o |
| proteasome activator complex subunit 1 | 16 | o | o | o | o | o | | o | o |
| scan domain-containing protein 3-like | 16 | o | o | o | | o | o | o | |
| tubulin beta chain variant 1 | 16 | | | | o | | | | o |
| vacuolar proton pump subunit e 1 | 16 | o | o | o | o | o | | | o |

| | | | | | | | | | |
|---|---|---|---|---|---|---|---|---|---|
| zgc:171352 protein | 16 | | o | o | o | | | | |
| 14-3-3 protein beta alpha | 15 | o | o | o | o | o | | o | o |
| 60s acidic ribosomal protein p2 | 15 | o | o | o | | o | o | o | |
| apolipoprotein c-ii | 15 | | o | o | o | o | o | | o |
| beta-2 microglobulin | 15 | | | | o | | | | o |
| beta-microseminoprotein precursor | 15 | | o | o | o | | o | | |
| complement component c9 | 15 | | o | o | o | | | | |
| complement factor h | 15 | | o | o | o | o | | | |
| copine iii | 15 | o | o | o | | | | o | o |
| cytochrome c oxidase subunit 4 isoform mitochondrial precursor | 15 | o | o | o | o | o | o | o | o |
| glycine cleavage system h mitochondrial precursor | 15 | o | o | o | o | o | | o | o |
| inter-alpha-trypsin inhibitor heavy chain h3-like | 15 | | o | | o | | | | o |
| low density lipoprotein receptor-related protein 1 | 15 | o | | | | | o | o | |
| malate mitochondrial precursor | 15 | o | o | o | | o | o | o | o |
| microfibril-associated glycoprotein 4-like | 15 | | o | | | | o | | |
| microtubule-associated protein 1a | 15 | | | | | o | o | o | |
| myelin basic protein | 15 | o | | | | o | o | o | o |
| ras homolog enriched in brain | 15 | o | o | o | | o | o | | o |
| ribosomal protein s12 | 15 | o | o | o | o | o | | o | |
| sjchgc03009 protein | 15 | o | o | o | | | o | o | o |
| succinate dehydrogenase iron-sulfur mitochondrial precursor | 15 | o | o | o | o | o | o | o | o |
| thioredoxin | 15 | o | o | o | o | o | | o | o |
| tpa_inf: rtn1 | 15 | | | | o | | | o | o |
| transgelin | 15 | o | | o | o | o | | o | o |
| uncharacterized protein kiaa1586 | 15 | o | | | | o | | o | |
| zinc finger bed domain-containing protein 1 | 15 | o | | | o | o | | o | o |
| alpha-2-hs-glycoprotein precursor | 14 | | | | o | | | | |
| basic leucine zipper and w2 domains 1 | 14 | o | o | o | o | o | | | |
| beta-2-microglobulin precursor | 14 | o | o | o | | o | | o | |
| betaine--homocysteine s-methyltransferase 1 | 14 | o | o | | o | | | | o |
| chaperonin containing subunit 6a (zeta 1) | 14 | o | o | | o | o | o | | o |
| cystatin precursor | 14 | o | o | o | o | o | o | o | o |
| dynein light chain tctex-type 3 | 14 | o | | o | | o | | | |
| eukaryotic translation initiation factor 5 | 14 | o | o | o | o | o | o | o | o |
| family with sequence similarity member a1 | 14 | o | o | o | | o | o | | |
| inter-alpha inhibitor h3 | 14 | o | o | o | | | o | | |
| malate cytoplasmic | 14 | o | o | o | o | | | o | o |
| nadh dehydrogenase subunit 5 | 14 | o | o | o | | o | o | o | o |
| nuclear protein 1 | 14 | o | o | o | o | o | | o | o |
| nuclear receptor subfamily group member 2 | 14 | o | o | o | | | o | o | |
| peroxiredoxin 5 | 14 | o | o | o | | o | o | o | o |
| ras-related protein rab-7a | 14 | o | o | o | | o | | o | o |
| retrovirus -like | 14 | o | | | | o | | o | |
| rnase k | 14 | o | o | o | | o | o | o | o |
| sbcfr-1 protein | 14 | | o | | o | | | | |
| serine incorporator 1 | 14 | o | o | o | | | o | o | o |
| solute carrier family member 3 | 14 | o | o | o | | | o | o | |
| transcription factor btf3 homolog 4 | 14 | o | o | o | o | o | o | o | |
| transmembrane protein 111 | 14 | o | o | o | o | o | | o | o |
| tributyltin binding protein type 1 | 14 | | | | o | | | | |
| ubiquitin specific peptidase 7 (herpes virus-associated) | 14 | o | | | | o | | o | |
| unconventional prefoldin rpb5 interactor-like | 14 | | | | o | | | | |
| vacuolar atp synthase 16 kda proteolipid subunit | 14 | o | o | o | o | o | | | o |

| | | | | | | | | | |
|---|---|---|---|---|---|---|---|---|---|
| annexin a5 | 13 | o | o | o | o | o | o | | o |
| atp synthase-coupling factor mitochondrial precursor | 13 | o | o | o | o | o | | o | o |
| baculoviral iap repeat-containing 6 | 13 | o | o | | | | o | | |
| c1 inhibitor | 13 | | o | o | o | | | | |
| ccr4-not transcription subunit 1 | 13 | o | o | o | | | o | o | |
| cd81 antigen | 13 | o | o | o | | o | o | o | |
| complement factor h-related 1 | 13 | | o | o | o | | | | |
| cytochrome c oxidase polypeptide viia-liver mitochondrial precursor | 13 | o | o | o | | o | | o | |
| dna-damage-inducible transcript 4 | 13 | o | o | o | | | | | o |
| eh domain binding protein 1 | 13 | o | | | | | o | | |
| enhancer of rudimentary homolog | 13 | o | o | | o | o | | o | o |
| eukaryotic translation initiation factor 3 subunit g | 13 | o | o | o | o | o | o | o | o |
| gag-pol fusion polyprotein | 13 | | | o | | o | o | o | |
| glutathione peroxidase 4b | 13 | o | o | | o | o | | o | o |
| glutathione s-transferase theta-1 | 13 | o | o | o | o | o | | | o |
| imap family member 4-like | 13 | o | | o | | | | | |
| interferon-induced very large gtpase 1-like | 13 | o | | o | | | | | |
| isotocin precursor | 13 | | | | | o | o | o | o |
| leukocyte elastase inhibitor | 13 | o | o | o | | o | | | |
| mannose-binding protein c precursor | 13 | o | | | | o | | | |
| methionine adenosyltransferase alpha | 13 | o | o | o | | | o | o | |
| nadh dehydrogenase subunit 4 | 13 | o | o | o | | o | o | o | o |
| nicotinamide riboside kinase 2 | 13 | o | o | o | o | o | | o | o |
| nuclease-sensitive element-binding protein 1 | 13 | o | o | | o | | o | o | o |
| pituitary tumor-transforming gene 1 protein-interacting protein precursor | 13 | o | o | o | o | o | | o | o |
| proteasome subunit alpha type-3 | 13 | o | o | | o | o | o | | o |
| proteasome subunit alpha type-7 | 13 | o | o | o | o | o | | o | o |
| ribosomal protein l7a | 13 | o | o | o | o | o | o | o | o |
| ribosomal protein l8 | 13 | | o | o | o | o | | o | o |
| ribosomal protein s9 | 13 | o | o | o | o | o | o | o | o |
| serine threonine-protein phosphatase pp1-beta catalytic subunit | 13 | o | o | o | o | o | o | o | o |
| splicing factor 3b subunit 1 | 13 | o | o | o | o | o | o | o | o |
| tumor suppressor candidate 2 | 13 | o | o | o | o | | | o | o |
| 28s ribosomal protein mitochondrial precursor | 12 | o | o | | o | o | | o | |
| 40s ribosomal protein s24 | 12 | o | o | o | o | o | o | | |
| 40s ribosomal protein s27 | 12 | o | o | o | o | o | | o | |
| actin-related protein 2 3 complex subunit 4 | 12 | o | o | o | o | o | o | | o |
| ankyrin erythrocytic | 12 | o | | | | o | o | | |
| beta-2-glycoprotein 1-like | 12 | | o | | o | | o | | |
| calpain small subunit 1 | 12 | o | o | o | o | o | | o | o |
| casein kinase ii subunit alpha | 12 | | o | | | o | o | o | o |
| cathepsin h | 12 | o | | o | o | o | | o | o |
| ceruloplasmin | 12 | | o | o | o | | | | |
| chaperonin containing subunit 2 | 12 | o | o | o | | o | | o | o |
| complement factor h precursor | 12 | | | | o | | | | |
| cyclin g1 | 12 | o | o | o | o | o | | o | o |
| cytochrome b-c1 complex subunit 10 | 12 | o | o | o | | o | o | o | |
| cytochrome c oxidase polypeptide viii-mitochondrial precursor | 12 | o | o | o | | o | o | o | |
| cytochrome c oxidase subunit via polypeptide 1 | 12 | o | | o | o | o | o | o | |
| envelope polyprotein | 12 | o | | | o | o | | o | |
| eukaryotic translation elongation factor 1 gamma | 12 | o | o | o | o | o | | o | o |

| | | | | | | | | | |
|---|---|---|---|---|---|---|---|---|---|
| fam18b | 12 | o | o | o | o | o | | o | o |
| gamma-aminobutyric acid receptor-associated protein | 12 | o | o | o | o | | | o | o |
| glycyl-trna synthetase | 12 | o | o | o | o | | | o | o |
| growth hormone | 12 | | | | o | | | | |
| high-mobility group box 1 | 12 | | | o | o | o | | o | o |
| keratin 18 | 12 | o | o | o | | o | | | o |
| kiaa1586 protein | 12 | o | | | | | | | |
| lyr motif-containing protein 5 | 12 | o | o | o | | o | o | o | o |
| nad h dehydrogenase | 12 | | | | o | | | | |
| oligosaccharyltransferase complex subunit ostc | 12 | o | o | o | o | o | o | o | o |
| pleiotrophin precursor | 12 | o | | | | | o | o | o |
| prolow-density lipoprotein receptor-related protein 1 | 12 | o | o | | | | o | o | |
| proteasome subunit alpha type-4 | 12 | o | o | o | o | o | | o | o |
| protein s100-b | 12 | o | | | | | | o | o |
| pseudouridine rsu | 12 | | | | o | | | | |
| rna-binding protein 8a | 12 | o | o | o | o | o | | o | o |
| ryanodine receptor 3 | 12 | o | | | | o | o | | |
| small ubiquitin-related modifier 2 precursor | 12 | o | | o | | o | | o | o |
| spen transcriptional regulator | 12 | o | o | o | o | o | o | o | o |
| splicing arginine serine-rich 11 | 12 | o | o | o | | o | | o | |
| transcription elongation factor b polypeptide 2 | 12 | o | o | o | o | o | | o | o |
| transmembrane protein 30a | 12 | o | | o | | | o | o | o |
| u6 snrna-associated sm-like protein lsm6 | 12 | o | o | | o | o | o | o | o |
| ubiquitin a-52 residue ribosomal protein fusion product 1 | 12 | o | o | o | o | o | | | o |
| ubiquitin protein ligase e3 component n-recognin 4 | 12 | o | o | | | | o | | |
| ubiquitin-conjugating enzyme e2 d4 | 12 | o | o | o | | o | | o | |
| vacuolar protein sorting-associated protein 11 homolog | 12 | o | o | o | o | | | o | o |
| voltage-dependent anion channel 2 | 12 | o | o | o | | o | | o | o |
| x-box binding protein 1 | 12 | o | o | o | | | | | o |
| 26s protease regulatory subunit 7 | 11 | o | o | o | o | | o | | o |
| 26s proteasome non-atpase regulatory subunit 4 | 11 | o | o | o | o | o | o | | o |
| 60s acidic ribosomal protein p1 | 11 | o | o | o | o | | | | o |
| 60s ribosomal protein l3 | 11 | o | | | o | | | o | o |
| alpha-2-macroglobulin-like protein 1-like | 11 | | o | | o | | | | |
| catechol-o-methyltransferase domain-containing protein 1 | 11 | o | o | o | o | o | | o | |
| coatomer subunit beta | 11 | o | o | o | | | o | o | o |
| coiled-coil domain-containing protein 56 | 11 | o | o | o | o | o | | o | |
| complement factor b | 11 | | o | o | o | | | | |
| complement factor d precursor | 11 | o | o | | | | | | |
| complement factor properdin | 11 | | o | o | o | | | | |
| cyclic amp-dependent transcription factor atf-4 | 11 | o | o | o | | o | o | o | o |
| cytochrome c oxidase subunit vib isoform 1 | 11 | o | o | o | | o | o | o | o |
| cytoplasmic dynein 1 heavy chain 1-like | 11 | | o | o | | o | o | o | o |
| deah (asp-glu-ala-his) box polypeptide 15 | 11 | o | o | o | | o | o | o | |
| diablo mitochondrial precursor | 11 | o | o | o | o | o | | o | o |
| dna-binding protein inhibitor id-1 | 11 | o | o | o | o | o | | o | o |
| elongation factor 1-gamma | 11 | | | | o | | o | | o |
| ethanolamine kinase 1 | 11 | o | o | o | | | o | o | |
| eukaryotic translation initiation factor 4 gamma 1 | 11 | o | o | o | o | o | o | o | |

| | | | | | | | | | |
|---|---|---|---|---|---|---|---|---|---|
| gamma-secretase subunit pen-2 | 11 | o | o | o | o | o | | o | o |
| glycogen debranching enzyme | 11 | o | | | | | | | |
| glyoxylate reductase hydroxypyruvate reductase | 11 | o | o | o | o | | | | o |
| gtf2i repeat domain containing 2 | 11 | o | | | | | o | o | |
| guanine nucleotide binding protein (g protein) gamma 5 | 11 | o | o | o | | o | o | o | |
| heterogeneous nuclear ribonucleoprotein k | 11 | o | o | o | o | o | o | o | |
| heterogeneous nuclear ribonucleoprotein l | 11 | o | o | o | | o | o | o | o |
| hexokinase 1 | 11 | o | o | | | | | | o |
| hypothetical loc792613 | 11 | o | o | o | o | o | o | o | |
| immunoglobulin m heavy chain secreted form | 11 | o | | o | | o | | | |
| integral membrane protein 1 | 11 | o | o | o | o | o | | o | o |
| inter-alpha-trypsin inhibitor heavy chain h3 | 11 | | o | | o | | | | |
| member of ras oncogene family | 11 | o | o | o | | o | | | o |
| myeloid leukemia factor 1 | 11 | o | | | | | | | |
| nadh dehydrogenase subunit 2 | 11 | | o | o | o | o | o | o | o |
| novel nacht domain containing protein | 11 | o | | o | | o | | o | |
| probable glutamate receptor precursor | 11 | | | | | o | o | o | o |
| protein ambp-like | 11 | | | | o | | | | |
| retinoic acid receptor responder protein 3 | 11 | o | o | o | o | o | | o | o |
| retinol binding protein cellular | 11 | o | o | o | | o | | o | |
| riken cdna isoform cra_a | 11 | o | | o | | o | | o | |
| saccharopine dehydrogenase | 11 | o | o | | | o | | | |
| scavenger receptor class member 2 | 11 | o | o | | | | o | o | |
| secreted protein | 11 | | | | o | | | | |
| serine threonine-protein phosphatase 2a catalytic subunit beta isoform | 11 | o | o | o | | o | | o | o |
| serum lectin isoform 3 | 11 | o | o | o | | | | | |
| sodium potassium-transporting atpase subunit beta-233 | 11 | o | o | | | o | o | o | o |
| sorcin | 11 | o | | o | o | o | | o | o |
| sorting nexin 14 | 11 | o | o | | | o | o | o | o |
| spastic ataxia of charlevoix-saguenay | 11 | o | | o | | | o | o | |
| speg complex locus | 11 | o | | | | | o | o | |
| s-phase kinase-associated protein 1 | 11 | o | o | o | o | o | o | o | o |
| succinate dehydrogenase | 11 | o | o | | o | o | o | o | o |
| tubulin alpha-4a chain | 11 | o | o | | o | o | | o | o |
| tubulin beta-1 chain | 11 | o | o | o | | o | | | o |
| ubiquinol-cytochrome c reductase core protein ii | 11 | o | o | | | o | | | o |
| ubiquitin-conjugating enzyme e2 a | 11 | o | o | o | | o | o | o | o |
| ubiquitin-conjugating enzyme e2 g1 | 11 | o | o | o | o | o | | o | |
| ubiquitin-conjugating enzyme e2 k | 11 | o | o | o | | o | o | o | |
| ubiquitin-conjugating enzyme e2 variant 2 | 11 | o | o | o | o | | o | o | o |
| udp-glucuronosyltransferase 2a1-like | 11 | o | | o | | | | o | |
| vitellogenin | 11 | | | o | | | | | |
| 26s proteasome non-atpase regulatory subunit 8 | 10 | o | o | o | o | o | o | o | o |
| 40s ribosomal protein s11 | 10 | o | o | o | o | o | | o | o |
| 40s ribosomal protein s19 | 10 | | o | | o | | o | o | o |
| 60s ribosomal protein l10a | 10 | o | o | o | o | o | o | o | |
| 60s ribosomal protein l32 | 10 | o | o | o | o | o | | o | o |
| acyl- -binding domain-containing protein 7 | 10 | o | o | o | | o | o | o | o |
| acyl-coenzyme a c-4 to c-12 straight chain | 10 | o | o | o | o | | | o | o |
| alanine-glyoxylate aminotransferase | 10 | | o | o | o | | | | o |
| alpha globin | 10 | o | o | | o | o | | | o |
| atpase mitochondrial precursor | 10 | o | o | o | | o | o | o | o |
| bccip homolog | 10 | o | o | o | o | o | | o | o |
| bleomycin hydrolase | 10 | o | o | o | o | o | | o | o |

| | | | | | | | | | |
|---|---|---|---|---|---|---|---|---|---|
| calcium voltage- l alpha 1s subunit | 10 | o | | | | | | | |
| carnitine acetyltransferase | 10 | o | o | o | | | | o | o |
| coagulation factor vii | 10 | | o | o | o | | | | o |
| complement component 1 q subcomponent-binding mitochondrial precursor | 10 | o | o | o | o | | | o | |
| cytochrome b | 10 | o | o | o | o | o | o | o | o |
| cytochrome c oxidase subunit i | 10 | o | o | o | | | o | o | |
| dead (asp-glu-ala-asp) box polypeptide 1 | 10 | o | o | o | | o | | o | |
| deleted in malignant brain tumors 1 | 10 | | o | o | o | | | | |
| diacylglycerol o-acyltransferase 2 | 10 | o | o | o | o | | o | | |
| diamine acetyltransferase 1 | 10 | o | o | o | | o | | o | |
| eukaryotic translation initiation factor 3 subunit i | 10 | o | o | o | o | o | o | | o |
| family with sequence similarity member c | 10 | o | o | o | o | | o | | |
| fk506 binding protein 25kda | 10 | o | o | o | | o | o | o | o |
| fk506-binding protein 3 | 10 | | | | o | | | | o |
| gamma-interferon-inducible lysosomal thiol reductase precursor | 10 | o | | o | o | o | | o | o |
| glutaryl-coenzyme a dehydrogenase | 10 | o | o | o | o | | | | |
| gtp-binding protein sar1b | 10 | o | o | o | | | o | | |
| h chain thrombin inhibitor complex | 10 | | | | o | | | | o |
| h+ v1 subunit h | 10 | o | o | o | | o | | o | o |
| high mobility group protein b1 | 10 | o | | | o | | | o | o |
| histone deacetylase complex subunit sap18 | 10 | o | o | o | o | | | o | |
| hydroxyacyl-coenzyme a mitochondrial precursor | 10 | o | o | o | o | o | | o | o |
| isoamyl acetate-hydrolyzing esterase 1 homolog | 10 | o | o | o | o | o | | o | |
| kinesin family member 1a | 10 | | o | | | | o | o | |
| lipoprotein lipase | 10 | o | o | o | o | | | o | |
| major facilitator superfamily domain containing 2 | 10 | o | o | o | | | o | | o |
| microtubule-associated protein 1b | 10 | o | | | | o | o | o | o |
| mitochondrial precursor | 10 | o | | | o | o | | o | o |
| mki67 fha domain-interacting nucleolar phospho | 10 | o | o | o | o | o | | o | o |
| mrna turnover protein 4 homolog | 10 | | o | o | o | o | | o | o |
| na+ k+ alpha 3a polypeptide | 10 | o | | | | o | o | o | |
| nedd8 precursor | 10 | o | o | o | | o | | o | o |
| non-erythrocytic 1 | 10 | o | | | | | o | o | |
| nucleotide-binding oligomerization domain containing 2-like | 10 | o | | | | o | | o | |
| peptidyl-prolyl cis-trans isomerase-like | 10 | | | | o | | | | o |
| peptidyl-prolyl cis-trans mitochondrial precursor | 10 | o | o | o | o | o | o | o | |
| phosphoglucose isomerase | 10 | o | o | o | | | | o | o |
| phospholemman precursor | 10 | o | o | o | | o | | o | o |
| probable e3 ubiquitin-protein ligase mycbp2 | 10 | o | o | o | | o | o | o | o |
| proliferating cell nuclear antigen | 10 | o | o | | o | o | | o | |
| protein lsm12 homolog | 10 | o | o | o | | o | | o | |
| protein phosphatase catalytic subunit | 10 | o | o | o | | o | | o | o |
| protein rer1 | 10 | o | o | o | o | o | o | o | o |
| proteolipid protein 1b | 10 | o | | | o | | o | o | o |
| purine nucleoside phosphorylase | 10 | o | o | o | o | | | | |
| retinoid x receptor beta | 10 | o | o | o | | | o | o | |
| retrotransposon-derived protein peg10-like | 10 | o | | o | | o | | o | |
| secretogranin-2 precursor | 10 | | | | | o | o | o | o |
| selenoprotein t | 10 | o | o | o | | o | | o | |
| serine threonine-protein phosphatase pp1-gamma catalytic subunit | 10 | o | o | | | | o | o | |

| | | | | | | | | | |
|---|---|---|---|---|---|---|---|---|---|
| signal recognition particle 9 kda protein | 10 | o | o | o | o | o | | o | o |
| splicing factor proline glutamine rich (polypyrimidine tract binding protein associated) | 10 | o | o | o | | o | | o | |
| succinate dehydrogenase assembly factor mitochondrial precursor | 10 | o | o | o | | o | | o | o |
| survival of motor neuron-related-splicing factor 30 | 10 | o | o | o | o | o | | o | o |
| tetraspanin 7 | 10 | o | o | | | o | o | o | o |
| ubiquitin carboxyl-terminal hydrolase isozyme l1 | 10 | o | | | o | | | o | o |
| ubiquitin-activating enzyme e1 | 10 | o | | o | | | o | o | |
| ubiquitin-conjugating enzyme e2 l3 | 10 | o | o | o | o | o | o | o | o |
| vitellogenin b | 10 | | | o | | | | | |
| vitronectin precursor | 10 | | o | o | o | | | | o |
| xin actin-binding repeat containing 2 | 10 | o | | | | | | | |
| zinc finger bed domain-containing protein 5 | 10 | o | | o | | o | | | |
| 14-3-3 protein beta alpha-2 | 9 | | | o | o | o | o | o | |
| 1-acylglycerol-3-phosphate o-acyltransferase 4 (lysophosphatidic acid delta) | 9 | o | o | o | | | | | |
| 40s ribosomal protein s23 | 9 | o | o | o | o | o | o | o | |
| 40s ribosomal protein s8 | 9 | | | | o | | | | o |
| 4f2 cell-surface antigen heavy chain | 9 | o | o | | | o | o | o | o |
| 60s ribosomal protein l10 | 9 | o | o | o | o | o | o | o | |
| 60s ribosomal protein l12 | 9 | o | o | o | o | o | | o | |
| 60s ribosomal protein l17 | 9 | o | | o | o | o | | o | o |
| 6-phosphofructokinase type c | 9 | | o | | | | o | | o |
| a chain low resolution structures of bovine mitochondrial f1-atpase during controlled dehydration: hydration state | 9 | o | | o | | o | o | o | |
| actin | 9 | o | o | | o | o | | | |
| adenosine kinase | 9 | o | o | o | o | o | | | |
| adp-ribosylation factor 1 | 9 | o | o | o | o | | | o | |
| apolipoprotein c-i-like | 9 | | | | o | | | | o |
| apoptosis-associated speck-like protein containing a card | 9 | o | | o | o | | | | |
| b-cell receptor-associated protein 31 | 9 | o | o | o | o | o | | | |
| bcl2 adenovirus e1b 19 kda protein-interacting protein 3 | 9 | o | o | o | | o | | o | o |
| biotinidase precursor | 9 | | o | o | o | | | | |
| cathepsin b | 9 | o | o | o | o | o | | o | |
| cathepsin f | 9 | | o | | | | o | | o |
| cathepsin l | 9 | o | o | o | | o | o | o | |
| cation transport regulator-like protein 1 | 9 | o | o | o | o | o | | o | o |
| cd59 glycoprotein precursor | 9 | o | | o | | o | | o | o |
| chaperonin containing subunit 5 | 9 | o | o | o | o | o | o | o | |
| clusterin | 9 | o | | | | o | o | o | |
| coiled-coil-helix-coiled-coil-helix domain containing 2 variant 1 | 9 | o | o | | | o | o | o | o |
| conserved helix-loop-helix ubiquitous kinase | 9 | o | o | | | | o | | |
| cop9 signalosome complex subunit 6 | 9 | o | o | o | o | o | | o | o |
| copper transport protein atox1 | 9 | o | o | o | o | o | | o | o |
| cyclin b1 | 9 | o | o | | o | | | | |
| cytoplasmic linker associated protein 2 | 9 | o | o | | | | o | o | o |
| cytoskeletal calmodulin and titin-interacting | 9 | o | | | | | o | | o |
| dehydrogenase reductase sdr family member 12 | 9 | | o | | | | o | o | o |
| double-stranded rna-binding protein staufen homolog 1 | 9 | o | o | | | | o | | o |
| e3 ubiquitin-protein ligase march7 | 9 | o | | o | o | | o | o | |

| | | | | | | | | | |
|---|---|---|---|---|---|---|---|---|---|
| ependymin precursor | 9 | o | o | o | | o | | o | o |
| f1f0-type atp synthase subunit g | 9 | o | | o | | o | | o | |
| fibrinogen beta chain | 9 | | o | o | o | | | | |
| fibrinogen gamma chain | 9 | | o | o | o | | | | o |
| fibulin 2 | 9 | o | | | | | | | |
| fructose- -bisphosphatase 1 | 9 | | o | o | o | | | | o |
| g1 to s phase transition 1 | 9 | o | o | o | | o | | | o |
| general transcription factor ii-i repeat domain-containing protein 2a-like | 9 | o | | o | | | o | | |
| glycogenin 1 | 9 | o | o | | o | | | | |
| golgi snap receptor complex member 1 | 9 | o | o | o | o | o | | o | o |
| growth hormone inducible transmembrane protein | 9 | o | o | o | | o | o | o | |
| histone cluster h1b-like | 9 | o | | | | o | | o | |
| histone cluster h2bc-like | 9 | o | | | | o | | | |
| importin subunit alpha-2 | 9 | o | o | | o | | o | | o |
| integral membrane protein 2c | 9 | o | o | o | | o | o | o | o |
| isochorismatase domain-containing protein mitochondrial precursor | 9 | o | o | o | o | o | o | o | o |
| isocitrate dehydrogenase 3 (nad+) alpha | 9 | o | o | o | | o | o | o | o |
| jagunal homolog 1 | 9 | o | o | o | o | o | | o | |
| loc400590 protein | 9 | o | o | | o | o | | o | o |
| low molecular weight phosphotyrosine protein phosphatase | 9 | o | | o | | o | o | o | |
| lower subunit | 9 | | o | o | o | o | | o | |
| lysosomal protective | 9 | | | | o | | | | o |
| lysyl-trna synthetase | 9 | o | o | o | | o | o | | |
| manganese superoxide dismutase | 9 | o | o | o | | o | | o | o |
| methionine aminopeptidase 1 | 9 | o | | o | o | | o | o | |
| mitochondrial fission 1 protein | 9 | o | o | o | o | o | o | o | o |
| mitochondrial import receptor subunit tom40 homolog | 9 | o | o | | o | o | | o | o |
| mitogen-activated protein kinase 14a | 9 | o | o | | o | | o | o | |
| myosin phosphatase rho interacting protein | 9 | o | | | | | o | o | o |
| nad-dependent deacetylase sirtuin-5 | 9 | o | o | | | | o | | o |
| nfix protein | 9 | o | | o | | o | | o | |
| nop56 protein | 9 | | | | o | | | | o |
| novel protein dna polymerases | 9 | o | | o | | o | | o | |
| nuclear migration protein nudc | 9 | o | | o | o | o | | o | o |
| nuclease harbi1-like | 9 | o | | o | o | o | | | |
| o-linked n-acetylglucosamine transferase (udp-n-acetylglucosamine:polypeptide-n-acetylglucosaminyl transferase) | 9 | o | o | o | | | o | o | |
| orf1-encoded protein | 9 | o | | | | | o | o | |
| phosphoribosylaminoimidazole phosphoribosylaminoimidazole succinocarboxamide synthetase | 9 | o | o | o | o | | | o | o |
| pl-5283 protein | 9 | o | o | o | | o | | o | |
| plexin a1 | 9 | o | | | | o | o | o | |
| pre-mrna-splicing factor syf2 | 9 | o | | o | o | o | o | o | o |
| probable signal peptidase complex subunit 2 | 9 | o | o | o | o | o | | o | |
| proteasome maturation protein | 9 | o | o | o | o | o | o | o | o |
| proteasome subunit beta type-7 precursor | 9 | o | o | o | | o | | o | o |
| reticulon 3 | 9 | o | o | o | | o | o | o | |
| retinoblastoma binding protein 4 | 9 | o | | | o | o | | o | |
| ribosomal protein l13a | 9 | o | o | o | o | o | | o | o |
| ribosomal protein l39 | 9 | o | | o | o | o | | o | o |
| ribosomal protein s26 | 9 | o | o | | o | o | | o | o |
| ribosomal protein s7 | 9 | o | o | o | o | o | | o | o |
| ribosome maturation protein sbds | 9 | o | o | o | | | | o | o |

| | | | | | | | | | |
|---|---|---|---|---|---|---|---|---|---|
| ring finger protein 121 | 9 | o | o | o | o | o | | o | o |
| rwd domain-containing protein 1 | 9 | o | | o | o | | o | o | o |
| s100-a1 | 9 | o | o | o | | o | | o | |
| sb:cb283 protein | 9 | o | o | | o | o | o | o | o |
| sialic-acid binding protein-4 | 9 | | | | | o | o | o | |
| signal recognition particle 19 kda protein | 9 | o | o | o | o | o | | o | o |
| small nuclear ribonucleoprotein e | 9 | o | o | | | o | o | o | o |
| small ubiquitin-related modifier 3 precursor | 9 | o | o | o | o | o | o | o | |
| solute carrier family member 1 | 9 | o | o | | | o | | o | o |
| something about silencing protein 10 | 9 | o | o | o | o | o | o | | o |
| spectrin alpha brain | 9 | o | o | | | o | o | o | o |
| stearoyl- desaturase | 9 | o | o | o | | o | o | o | o |
| sterol o-acyltransferase 1 | 9 | | o | o | o | | | | |
| subfamily member 3 | 9 | o | o | o | | o | | | |
| subfamily member 9 | 9 | o | o | o | o | o | o | | |
| synaptosome-associated protein 25a | 9 | | | | o | o | o | o | o |
| tax1 (human t-cell leukemia virus type i) binding protein 3 | 9 | o | o | | | | | | |
| t-cell receptor beta chain ana 11 | 9 | o | o | | | o | o | | o |
| t-complex protein 1 subunit delta | 9 | o | o | | o | o | o | | o |
| t-complex protein 1 subunit theta | 9 | o | o | o | o | o | | | o |
| tetraspanin 3 | 9 | o | o | o | o | o | | o | |
| tetraspanin-9 | 9 | o | o | o | | o | o | o | o |
| thioredoxin domain-containing protein 17 | 9 | o | o | | | o | o | o | o |
| tns1 protein | 9 | o | | | o | o | o | | |
| transcription initiation factor tfiid subunit 10 | 9 | o | o | o | o | o | | | o |
| transcription initiation factor tfiid subunit 12 | 9 | o | o | o | o | o | | o | o |
| translocon-associated protein subunit alpha precursor | 9 | o | o | o | | | | | |
| translocon-associated protein subunit delta precursor | 9 | o | o | o | | o | | o | o |
| transmembrane protein 50a | 9 | o | o | o | o | o | | o | o |
| transport protein sec61 subunit alpha | 9 | o | o | o | | | | o | o |
| triosephosphate isomerase b | 9 | | o | | o | o | | | o |
| tripartite motif-containing protein 39 | 9 | o | | o | | | | o | o |
| tropomyosin 4 | 9 | o | o | o | | o | o | o | |
| tyrosine aminotransferase | 9 | | o | o | o | | | | |
| ubiquitin | 9 | o | o | | o | | | o | o |
| ubiquitin fusion degradation protein 1 homolog | 9 | o | o | o | o | o | | o | o |
| ubiquitin thioesterase otub1 | 9 | o | o | o | o | o | | o | |
| ubiquitin-conjugating enzyme e2 e1 | 9 | o | o | o | | o | o | o | |
| ubiquitin-conjugating enzyme e2 g2 | 9 | o | o | o | o | | o | | o |
| ubiquitin-like modifier-activating enzyme 5 | 9 | o | o | o | | o | | | |
| ubiquitin-like protein 4a | 9 | o | o | o | o | o | | | o |
| ww domain containing e3 ubiquitin protein ligase 1 | 9 | o | | o | | | o | o | |
| zinc finger protein 214-like | 9 | o | | o | | | | o | |
| zpc domain containing protein 5 precursor | 9 | | | | o | | | | |
| 14 kda phosphohistidine phosphatase | 8 | o | | o | | o | | o | o |
| 14-3-3 protein epsilon | 8 | | | | | | | | o |
| 52 kda ro protein | 8 | o | | o | o | | | | |
| 5-aminolevulinate mitochondrial precursor | 8 | o | o | | o | o | o | | o |
| 60s ribosomal protein l37a | 8 | o | o | o | o | o | | | |
| acetyl-coenzyme a carboxylase alpha | 8 | o | o | o | | o | | o | |
| activated rna polymerase ii transcriptional coactivator p15 | 8 | o | o | | o | o | | o | o |
| acyl- synthetase long-chain family member 1 | 8 | o | o | o | | | | o | |
| adp-ribosylation factor 6 | 8 | o | o | o | o | | | | o |
| ae binding protein 1 | 8 | o | o | o | | o | | | |

| | | | | | | | | | |
|---|---|---|---|---|---|---|---|---|---|
| akirin 2 | 8 | o | | o | o | o | o | o | |
| aldose reductase | 8 | o | o | o | | o | | o | |
| alpha-1-microglobulin bikunin precursor | 8 | | o | o | o | | | | |
| alpha-kinase 3 | 8 | o | | | | | | | |
| alpha-n-acetylgalactosaminidase | 8 | o | o | o | o | | | o | |
| and pleckstrin domain protein 1 (chondrocyte-derived) | 8 | o | | | | | o | | |
| ankyrin repeat and socs box-containing 5 | 8 | o | | | | o | o | | |
| annexin a11 | 8 | o | o | o | | o | | | |
| annexin a13 | 8 | o | o | | | | o | | o |
| annexin a3 | 8 | o | | | o | | | | |
| apolipoprotein c-i | 8 | | o | | o | | | o | |
| atp-binding cassette sub-family f member 2 | 8 | | o | o | o | o | | o | o |
| beta-2-glycoprotein 1 precursor | 8 | o | o | o | | | | | |
| bile salt-activated lipase-like | 8 | | | | o | | | | o |
| biliverdin reductase a | 8 | o | | | o | o | | o | o |
| butyrophilin-like 8-like | 8 | o | | | | | | | |
| c3orf68 homolog | 8 | o | o | o | o | o | | o | o |
| calcium binding atopy-related autoantigen 1 | 8 | o | o | o | | o | | | o |
| calreticulin | 8 | o | o | o | | | | o | o |
| calsyntenin-3 precursor | 8 | | | | | | | | o |
| capping protein (actin filament) muscle z-alpha 1 | 8 | o | o | | | o | o | | o |
| carbonic anhydrase 4-like | 8 | | | | o | | | | |
| cd63 antigen | 8 | o | o | o | | o | o | | o |
| centrosomal protein of 95 kda-like | 8 | | | | | | | | o |
| chaperonin containing subunit 7 | 8 | o | o | | | | o | o | o |
| chromosome 1 open reading frame 151 | 8 | o | o | o | | o | | o | |
| clathrin heavy chain 1 | 8 | o | | o | | | o | o | |
| c-myc-binding protein | 8 | o | o | o | | o | o | o | |
| coagulation factor v | 8 | | o | o | o | | | | o |
| complement component 7 | 8 | o | o | o | | | | o | |
| c-type lectin domain family 4 member e | 8 | o | o | o | | | | o | |
| cysteine sulfinic acid decarboxylase | 8 | | | | o | | | | |
| cysteine-rich pdz-binding protein | 8 | o | o | o | | o | | o | o |
| cysteine-rich with egf-like domains 2 | 8 | o | o | o | o | | | | |
| dedicator of cytokinesis 3 | 8 | | | | | | o | o | |
| dimethylaniline monooxygenase | 8 | | | | o | | | | o |
| dna-binding protein inhibitor id-2 | 8 | o | | o | | o | o | o | o |
| dual specificity phosphatase 1 | 8 | o | | | | | | | |
| enoyl coenzyme a hydratase peroxisomal | 8 | o | o | o | o | o | | | o |
| ethylmalonic encephalopathy 1 | 8 | o | o | o | o | o | | o | o |
| eukaryotic translation initiation factor 2 subunit 3 | 8 | o | o | o | | | | o | o |
| eukaryotic translation initiation factor 3 subunit c | 8 | o | o | o | o | o | | | o |
| eukaryotic translation initiation factor 3 subunit d-like | 8 | | | | o | | o | | o |
| eukaryotic translation initiation factor 4e | 8 | o | o | o | o | o | o | | |
| exocyst complex component 3 | 8 | o | | o | | | | | o |
| extracellular matrix protein 1 | 8 | o | o | o | o | | | | |
| fatty acid desaturase domain member 6 | 8 | o | o | | o | o | o | o | o |
| fibronectin 1 | 8 | o | | o | o | | | o | |
| gamma-aminobutyric acid receptor-associated 1 | 8 | o | o | o | | o | | o | o |
| general transcription factor ii-i repeat domain-containing protein 2a | 8 | o | | o | | o | | o | |
| glycerol kinase | 8 | | o | | o | | o | | |
| herpud family member 2 | 8 | o | | o | | | | o | o |

| | | | | | | | | | |
|---|---|---|---|---|---|---|---|---|---|
| high affinity immunoglobulin epsilon receptor subunit gamma precursor | 8 | o | o | o | | o | | o | o |
| high mobility group-t protein | 8 | o | o | o | | o | | o | |
| histamine n-methyltransferase | 8 | o | o | o | o | o | o | | |
| homeodomain interacting protein kinase 3 | 8 | o | | | | | o | | |
| hypoxanthine-guanine phosphoribosyltransferase | 8 | o | | o | o | o | | o | |
| immediate early response 3 interacting protein 1 | 8 | o | o | o | | o | | | |
| insulin-degrading enzyme | 8 | o | o | | | | | | |
| integrin beta-1 precursor | 8 | o | o | o | o | o | o | | |
| interferon-inducible protein gig2-like | 8 | o | | o | | o | | o | |
| interferon-related developmental regulator 1 | 8 | o | o | o | | | | o | o |
| isopentenyl-diphosphate delta-isomerase 1 | 8 | o | | o | o | o | | | o |
| krueppel-like factor 6 | 8 | o | | | | | o | | |
| line-1 type transposase domain-containing protein 1-like | 8 | o | | | | | o | o | o |
| lwamide neuropeptides | 8 | | | | o | | | | |
| lysosomal protective protein | 8 | o | | o | o | o | | o | o |
| malignant t cell amplified sequence 1 | 8 | o | o | o | o | o | | o | |
| mimecan precursor | 8 | o | | | | | | o | o |
| mitochondrial ribosomal protein l24 | 8 | o | o | o | | o | | o | o |
| mitochondrial ribosomal protein l3 | 8 | o | o | o | o | o | | o | |
| mitochondrial ribosomal protein l54 | 8 | o | o | o | | o | | o | |
| mitogen-activated protein kinase 8 | 8 | o | o | | | | o | o | |
| myelin basic | 8 | | | | | | o | | o |
| myocyte enhancer factor 2a | 8 | o | | | | | | | |
| myomesin 1 | 8 | o | | | | | | | |
| myomesin 1 isoform 1 | 8 | o | | o | | | | | |
| myomesin 2 | 8 | o | | | | | | | |
| myotrophin | 8 | o | o | | | | o | o | o |
| nadh dehydrogenase 1 alpha subcomplex subunit 13 | 8 | o | o | o | | o | | o | |
| nedd4 family-interacting protein 1 | 8 | o | o | o | | | o | o | o |
| notochord-related protein | 8 | o | | o | | | o | | |
| novel protein containing multiple scavenger receptor cysteine-rich domains vertebrate deleted in malignant brain tumors 1 | 8 | o | | | | | | | |
| novel protein vertebrate interferon-induced protein 44 | 8 | o | | | | o | | o | |
| novel ubiquitin-protein ligase | 8 | o | | | | | | | |
| nuclear transport factor 2 | 8 | o | o | o | o | o | | o | |
| oxoglutarate (alpha-ketoglutarate) dehydrogenase | 8 | o | o | | | | o | | |
| palmitoyl-protein thioesterase 1 precursor | 8 | o | o | o | o | o | | o | o |
| parvalbumin | 8 | o | | | | o | o | o | |
| peptidyl-prolyl cis-trans isomerase | 8 | o | | o | | o | o | o | o |
| phosphatidylinositol transfer protein beta isoform | 8 | o | | o | | o | | o | o |
| phosphatidylinositol-binding clathrin assembly protein | 8 | o | o | | | | | | |
| phosphoribosyl pyrophosphate synthetase-associated protein 1 | 8 | o | o | o | o | o | | | o |
| phosphoserine phosphatase | 8 | o | o | o | | o | | o | o |
| plasminogen | 8 | | o | o | o | | | | o |
| plectin isoform 1hij | 8 | o | | | | | o | | |
| polo-like kinase 2 | 8 | o | | o | | | o | | |
| polyketide synthase | 8 | o | | | | | | | |
| pra1 family protein 3 | 8 | o | o | o | | | | o | o |
| probable ergosterol biosynthetic protein 28 | 8 | o | | | o | o | | o | o |

| | | | | | | | | | |
|---|---|---|---|---|---|---|---|---|---|
| probable ribosome biogenesis protein rlp24 | 8 | o | | o | o | o | | o | o |
| programmed cell death 8 (apoptosis-inducing factor) | 8 | o | o | o | | o | o | o | |
| prohibitin 2 | 8 | o | o | o | | o | | o | o |
| proteasome ( macropain) 26s 6 | 8 | o | o | o | | o | o | o | o |
| proteasome subunit beta type-1-a | 8 | o | o | o | | o | o | o | |
| protein bassoon-like | 8 | | | | | | o | | |
| protein lin-7 homolog b | 8 | | | | o | o | | o | o |
| pyruvate dehydrogenase beta | 8 | o | o | | | | | o | o |
| qil1 | 8 | o | o | o | | o | | o | o |
| receptor expression-enhancing protein 5 | 8 | o | o | o | o | o | | o | |
| replication protein a 70 kda dna-binding subunit | 8 | o | | | o | o | | o | o |
| rho gtpase activating protein 1 | 8 | o | | o | | o | o | o | |
| rho guanine nucleotide exchange factor 5 | 8 | | | o | o | | | o | o |
| ribophorin ii | 8 | o | o | o | | o | | | o |
| ribosomal protein l18 | 8 | o | o | | o | o | | o | o |
| ribosomal protein l19 | 8 | o | o | o | | o | o | o | o |
| ribosomal protein l23 | 8 | o | o | o | o | o | | | o |
| ribosomal protein l23a | 8 | o | o | o | o | o | | o | |
| ribosomal protein l27 | 8 | o | o | o | o | o | | o | |
| ribosomal protein s10 | 8 | o | o | o | o | o | o | | o |
| ribosomal protein s16 | 8 | o | o | o | o | o | o | o | |
| ribosomal protein s27a | 8 | o | o | o | o | o | | o | |
| ribosomal protein s8 | 8 | o | o | o | o | o | | o | |
| rna-binding protein pno1 | 8 | o | o | o | | o | | o | o |
| rna-directed dna polymerase from mobile element jockey-like | 8 | o | o | | o | | o | o | |
| s-adenosylhomocysteine hydrolase-like 1 | 8 | o | | | | | o | o | o |
| serine threonine-protein kinase 6 | 8 | o | o | | o | | | | |
| serpin peptidase clade a (alpha-1 antitrypsin) member 10 | 8 | o | o | o | | o | | | o |
| sine oculis-binding protein homolog | 8 | o | | o | o | | o | o | |
| solute carrier family member 4 | 8 | o | o | o | | | o | | |
| sparc precursor | 8 | o | o | o | | o | o | o | |
| splicing arginine serine-rich 3 | 8 | o | o | o | o | o | | o | |
| splicing factor 3b subunit 5 | 8 | o | o | o | o | o | | o | o |
| subfamily member 11 | 8 | o | o | o | | | | o | o |
| succinyl- ligase | 8 | | o | o | o | | o | o | o |
| synaptopodin 2 | 8 | o | | | | | | | |
| tax1-binding protein 1 homolog | 8 | o | | o | o | o | o | o | |
| thrombospondin 2 | 8 | o | | | | | | | |
| thymidylate kinase | 8 | o | o | o | | o | | | o |
| thyroid hormone receptor associated protein 3 | 8 | o | o | | | | o | | |
| tpa: endonuclease-reverse transcriptase | 8 | o | | | | | | | |
| transcription factor iiia | 8 | | | o | o | | | | |
| transducer of 1 | 8 | o | o | o | o | | | | |
| transforming acidic coiled coil 2 | 8 | o | | | | | o | | |
| translocon-associated protein subunit gamma | 8 | o | o | o | o | o | | o | |
| transmembrane 4 l6 family member 4 | 8 | o | o | o | o | o | | | |
| transmembrane 9 superfamily member 3 | 8 | o | o | o | | | | o | o |
| transmembrane protein 106b | 8 | o | o | o | o | | | o | |
| tubulin-specific chaperone d | 8 | | o | | o | o | | o | o |
| ubiquinol-cytochrome c rieske iron-sulfur polypeptide 1 | 8 | o | o | o | | o | | o | |
| ubiquitin carboxyl-terminal hydrolase 7-like | 8 | | o | | | | o | | o |
| ubiquitin conjugation factor e4 b | 8 | o | o | o | o | o | o | | o |
| ubiquitin protein ligase e3 component n-recognin 7 | 8 | o | | o | o | o | | | o |

| | | | | | | | | | |
|---|---|---|---|---|---|---|---|---|---|
| upf0608 protein c19orf42-like | 8 | o | | o | | o | o | o | o |
| urate oxidase | 8 | | o | o | o | | | | |
| vacuolar protein sorting-associated protein 29 | 8 | o | o | o | | o | o | o | o |
| viral a-type inclusion protein | 8 | o | | | o | | | | |
| vitronectin | 8 | | o | o | | | | | |
| von hippel-lindau binding protein 1 | 8 | o | o | | o | o | | o | o |
| whey acidic protein precursor | 8 | o | o | o | | o | | o | o |
| zgc:162565 protein | 8 | | | | | | | o | o |
| zgc:171445 protein | 8 | o | o | o | | | | | |
| zgc:174680 protein | 8 | o | | o | | o | o | | |
| zinc binding alcohol domain containing 2 | 8 | o | o | o | | o | | | o |
| zinc finger mym-type protein 1 | 8 | o | | o | | | | o | |
| zpax protein | 8 | | | | o | | | | |
| 28s ribosomal protein mitochondrial-like | 7 | | o | | o | | | | o |
| 40s ribosomal protein s2 | 7 | | o | | o | | o | | o |
| 60s ribosomal protein l27a | 7 | o | | o | o | o | | o | o |
| 60s ribosomal protein l9 | 7 | o | o | o | o | | | o | |
| abhydrolase domain containing 12 | 7 | o | o | | | o | | o | o |
| acetyl- mitochondrial precursor | 7 | o | o | o | o | o | | | |
| acetylserotonin o-methyltransferase-like | 7 | o | o | o | | | | | |
| adp-ribosylation factor 5 | 7 | o | o | o | | | | | |
| aldehyde dehydrogenase 4 member a1 | 7 | o | o | | o | | | | o |
| alpha-2-macroglobulin | 7 | | o | | o | | | | o |
| aminoacylase 1 | 7 | o | o | | o | o | | | o |
| amyloid beta a4 protein | 7 | o | | o | | | o | o | |
| ankyrin family a (rfxank-like) 2 | 7 | o | | | | o | | o | |
| ankyrin repeat domain 13c | 7 | o | o | o | | o | | | |
| ankyrin repeat domain-containing protein 13c | 7 | | | | | | | | o |
| ap-2 complex subunit mu-1 | 7 | o | | o | | | o | o | o |
| apoptosis-associated tyrosine kinase | 7 | o | | | | | o | o | |
| arrestin domain containing 3 | 7 | o | | | | | | o | |
| ash1 ( or homeotic)-like | 7 | o | | | | | | o | |
| aspartate cytoplasmic | 7 | o | o | | | o | | | o |
| aspartyl-trna synthetase | 7 | o | o | o | | | | o | |
| atp h+ mitochondrial f1 beta polypeptide | 7 | o | o | o | | | o | o | |
| atpase h+ transporting v1 subunit g isoform 1 | 7 | | o | o | | | | o | o |
| atp-binding sub-family a member 1 | 7 | o | o | | | o | | o | |
| atp-dependent clp protease atp-binding subunit clpx- mitochondrial-like | 7 | o | o | | | | o | o | |
| beta-galactoside-binding lectin | 7 | o | o | o | | o | | | o |
| bystin-like | 7 | o | o | | o | o | | o | |
| c10orf104 homolog | 7 | o | o | o | | o | | o | o |
| c12orf31 homolog | 7 | o | o | o | o | o | | o | o |
| c1orf74 homolog | 7 | o | o | o | o | o | | o | |
| c-1-tetrahydrofolate cytoplasmic | 7 | | o | | o | | | | |
| calcitonin gene-related peptide-receptor component protein | 7 | o | | o | | o | o | o | |
| capping protein (actin filament) muscle z-alpha 2 | 7 | o | o | o | | | | | o |
| cathepsin a | 7 | o | o | o | o | o | | | |
| c-c motif chemokine 25 precursor | 7 | o | o | o | o | o | | | o |
| cdp-diacylglycerol--serine o-phosphatidyltransferase | 7 | o | o | | | o | o | o | |
| cell cycle associated protein 1 | 7 | o | o | | | o | o | o | o |
| cell division control protein 42 homolog precursor | 7 | o | o | o | | | o | o | |
| cell division cycle protein 20 homolog | 7 | | o | | o | | | | |
| cellular apoptosis susceptibility protein | 7 | o | o | | | | | | |
| cellular repressor of e1a-stimulated genes 2 | 7 | o | o | o | o | o | | o | o |
| claudin-like protein zf-a89 | 7 | | | | o | | | | |

| | | | | | | | | | |
|---|---|---|---|---|---|---|---|---|---|
| cmp-sialic acid transporter | 7 | o | o | | o | o | | | |
| coagulation factor ii precursor | 7 | | | | o | | | | |
| coiled-coil domain-containing protein 72 | 7 | o | | o | o | o | o | o | |
| cold shock domain-containing protein e1 | 7 | o | o | o | | | o | | |
| collagen alpha-1 chain | 7 | o | o | | | | o | | |
| collagen type i alpha 1 | 7 | o | o | o | | | o | | |
| complement c5 | 7 | | o | o | o | | | | |
| cop9 constitutive photomorphogenic homolog subunit 8 | 7 | o | o | o | | o | | o | o |
| cop9 signalosome complex subunit 5 | 7 | o | o | o | | o | | o | |
| CR1-3 [Lycodichthys dearborni] | 7 | | o | | o | | o | | |
| creb atf bzip transcription factor | 7 | o | o | o | | o | | | |
| crystallin j1a | 7 | o | o | o | o | | | | o |
| cysteine and glycine-rich protein 1 | 7 | o | | o | | o | | | o |
| cytochrome c oxidase copper chaperone | 7 | o | o | | o | o | | o | |
| cytochrome p450 | 7 | o | o | | o | | | | |
| cytochrome p450 1a | 7 | o | | | o | | | | |
| developmentally regulated gtp binding protein 1 | 7 | o | o | o | o | o | | o | |
| dihydrolipoamide s-succinyltransferase (e2 component of 2-oxo-glutarate complex) | 7 | o | o | o | | | | | o |
| dmx-like 2 | 7 | | | | | | o | o | |
| dystonin | 7 | o | | | | o | o | o | o |
| egg membrane protein | 7 | | | o | o | | | | |
| elongation factor-1 alpha | 7 | | | | | | | | o |
| enolase 1 isoform b | 7 | | | | o | | | | o |
| enolase alpha non-neuron | 7 | o | o | | | | o | o | o |
| epidermis-type lipoxygenase 3-like | 7 | | | | o | | | | |
| es1 protein mitochondrial | 7 | o | | o | | o | | o | |
| eukaryotic translation initiation factor 3 subunit e | 7 | o | o | o | | o | | o | o |
| eukaryotic translation initiation factor 3 subunit m | 7 | o | o | o | | o | | o | o |
| eukaryotic translation initiation factor x-chromosomal | 7 | o | o | o | o | o | | o | o |
| extended synaptotagmin-1 | 7 | o | | | | | o | | o |
| extracellular superoxide dismutase | 7 | o | | | o | o | | | |
| f-box only protein 2 | 7 | | | | | | | o | o |
| filamin a interacting protein 1 | 7 | o | | | | | | | |
| fk506-binding protein 11 precursor | 7 | o | o | o | | o | | | o |
| fk506-binding protein 2 precursor | 7 | o | o | o | o | o | | | o |
| flotillin 1 | 7 | o | o | o | | | | | o |
| fun14 domain containing 1 | 7 | o | | o | | o | | o | o |
| gaba receptor-associated 2 | 7 | o | o | o | | o | | o | o |
| gap junction protein | 7 | | o | o | | | | | |
| general transcription factor ii-i repeat domain-containing protein 2 | 7 | o | o | | | | | | |
| glioma tumor suppressor candidate region gene 2 | 7 | | | o | o | | | | |
| glutamate | 7 | | o | | o | o | o | | o |
| glutaredoxin 3 | 7 | o | o | o | | o | | o | o |
| glutathione peroxidase 3 | 7 | o | o | o | | o | o | o | |
| glycoprotein m6b | 7 | o | | | | o | o | | o |
| guanine nucleotide-binding 3 | 7 | o | o | | o | | | | o |
| guanylate kinase | 7 | o | | o | o | o | | o | |
| harbinger transposase derived 1 | 7 | o | | | | | | | |
| heat shock protein hsp 90-alpha | 7 | o | | | | o | o | | o |
| helentron 4 helitron-like transposon replicase helicase endonuclease partial | 7 | | | | | | o | | |
| high choriolytic enzyme 2-like | 7 | | | | o | | | | |

| | | | | | | | | | |
|---|---|---|---|---|---|---|---|---|---|
| high density lipoprotein binding protein | 7 | o | o | o | | | | | |
| histone h2a | 7 | o | | o | o | | | | |
| histone h2b | 7 | o | | o | | | o | o | |
| immunoglobulin light chain | 7 | o | | | | | | | o |
| importin 7 | 7 | o | o | o | | o | | | |
| kelch domain containing 1 | 7 | o | o | o | | | | | |
| kinesin family member 1b | 7 | o | | | | | o | o | |
| lactate dehydrogenase b | 7 | | | | | o | o | | o |
| lactoylglutathione lyase | 7 | o | | | o | o | o | o | o |
| lecithin-cholesterol acyltransferase | 7 | o | o | | | | | o | |
| leucine rich repeat containing 2 | 7 | o | | | | | | | |
| leydig cell tumor 10 kda protein homolog | 7 | o | o | o | o | o | | o | o |
| lim domains containing 1 | 7 | o | o | o | | | | o | |
| membrane-associated ring finger 6 | 7 | o | o | | | | o | | |
| metaxin 2 | 7 | o | | o | | | | o | o |
| methylmalonate-semialdehyde dehydrogenase | 7 | o | o | | o | o | | | |
| microfibrillar-associated protein 1 | 7 | o | o | | o | | | | o |
| microspherule protein 1 | 7 | o | | o | o | o | o | o | |
| mitochondrial atp synthase gamma chain | 7 | o | o | o | | o | o | o | |
| mitochondrial import receptor subunit tom5 homolog | 7 | o | o | o | | o | o | o | |
| mitogen-activated protein kinase 3 | 7 | o | | | | o | o | o | |
| mitogen-activated protein-binding protein-interacting protein | 7 | o | o | o | o | o | | o | o |
| mx protein | 7 | o | | o | o | | | | |
| myelin proteolipid protein | 7 | | | | | | | | o |
| myeloma overexpressed 2 | 7 | o | | o | | o | o | o | |
| myomesin (m-protein) 165kda | 7 | o | | | | | | | |
| myst histone acetyltransferase 1 | 7 | o | o | o | o | o | | | |
| nadh dehydrogenase 1 alpha subcomplex subunit 4 | 7 | o | | o | o | o | | o | |
| nadh dehydrogenase subunit 1 | 7 | o | o | o | | o | o | o | o |
| nadh-cytochrome b5 reductase 3 | 7 | | o | o | | | | o | o |
| nadh-ubiquinone oxidoreductase 75 kda mitochondrial precursor | 7 | o | o | | o | o | | | |
| neutral alpha-glucosidase ab-like | 7 | o | o | o | | | o | o | |
| nf-kappa-b inhibitor alpha | 7 | o | o | o | | o | | o | |
| nhp2-like protein 1 | 7 | o | | o | o | o | o | o | |
| nicotinamide nucleotide transhydrogenase | 7 | o | o | | | | o | | |
| novel immune type receptor | 7 | o | | | o | | | o | |
| nuclear protein hcc-1 | 7 | o | o | o | o | o | | o | |
| nuclease harbi1 | 7 | o | | | | | | o | |
| nucleolar protein 120kda | 7 | o | o | | | o | o | | |
| orf2 | 7 | o | | | | | o | o | |
| pancreatic progenitor cell differentiation and proliferation factor | 7 | o | o | o | | o | | o | |
| peroxiredoxin 1 | 7 | o | o | o | | o | o | o | |
| phosphatidylinositol 4-kinase alpha | 7 | | o | o | | | o | | |
| phosphatidylinositol n-acetylglucosaminyltransferase subunit h | 7 | o | o | o | o | | | o | |
| phospholipase beta 1 (phosphoinositide-specific) | 7 | o | | | | | o | o | |
| phosphoserine aminotransferase 1 | 7 | o | | o | | o | | o | |
| pol protein | 7 | | | o | o | | o | o | |
| pre-mrna branch site protein p14 | 7 | o | o | o | o | o | o | o | |
| pre-mrna-processing-splicing factor 8 | 7 | o | o | | o | o | o | | |
| pre-mrna-splicing factor 18 | 7 | o | o | o | o | o | | | |
| probable atp-dependent rna helicase ddx5-like | 7 | | | | | | | | o |
| probable dimethyladenosine transferase | 7 | o | o | o | o | o | | o | |
| procollagen c-endopeptidase enhancer | 7 | o | o | o | o | o | | o | |

| | | | | | | | | | |
|---|---|---|---|---|---|---|---|---|---|
| proteasome activator complex subunit 3 | 7 | o | o | | o | | | | |
| proteasome subunit alpha type-2 | 7 | o | o | o | | | o | o | o |
| proteasome subunit alpha type-6 | 7 | o | o | o | | o | o | o | |
| proteasome subunit beta type-3 | 7 | o | o | o | o | o | | o | |
| protein bassoon | 7 | | | | | | o | o | |
| protein kinase c and casein kinase substrate in neurons 3 | 7 | o | | | | o | | o | |
| protein phosphatase regulatory subunit delta isoform | 7 | o | o | o | | | o | | |
| protein tyrosine phosphatase type member 2 | 7 | o | o | o | | | o | | o |
| protein tyrosine phosphatase-like a domain containing 1 | 7 | o | | o | o | o | | o | |
| protein yippee-like 5-like | 7 | | | | | | | | o |
| proteoglycan 4 | 7 | o | o | o | o | | | | |
| quaking protein | 7 | o | | | | | o | o | |
| ras-related c3 botulinum toxin substrate 1 (rho small gtp binding protein rac1) | 7 | o | o | o | | | | | o |
| rho family gtpase 1 | 7 | | | o | | o | o | o | |
| ribosomal p0 | 7 | | | | o | | | | o |
| ribosomal protein l13 | 7 | o | o | o | o | o | | o | |
| ribosome binding protein 1 homolog 180kda | 7 | o | | o | | o | o | o | |
| ring finger protein 185 | 7 | o | o | o | o | o | | | o |
| rna binding motif protein 24 | 7 | o | | | | | | | |
| rna binding motif protein 39 | 7 | o | o | o | | | | o | |
| rna binding motif protein 5 | 7 | o | o | o | | o | | | |
| rna-binding protein 5 | 7 | o | o | o | | | o | | o |
| ryanodine receptor 1b | 7 | o | | | | | | | |
| saps domain member 2 | 7 | o | | | | | o | o | |
| selenoprotein p precursor | 7 | o | o | | o | | | | o |
| selenoprotein u | 7 | o | o | o | | o | | | o |
| serine threonine-protein phosphatase 4 catalytic subunit | 7 | o | o | o | o | o | | | o |
| s-formylglutathione hydrolase | 7 | o | o | o | | | | o | o |
| sh3 domain-binding glutamic acid-rich-like protein | 7 | o | o | | | o | | o | o |
| sh3-domain grb2-like endophilin b2 | 7 | o | | | o | o | | | o |
| sideroflexin 2 | 7 | o | o | o | o | o | | | o |
| single stranded dna binding protein 3 | 7 | o | | | | | o | o | o |
| slu7 protein | 7 | o | | o | o | | o | | o |
| sodium channel subunit beta-1 precursor | 7 | o | | o | | o | | | o |
| solute carrier family 6 (neurotransmitter gaba) member 1 | 7 | | | | | | o | o | |
| src kinase-associated phosphoprotein 2 | 7 | o | | | o | | | | |
| stress-induced-phosphoprotein 1 | 7 | o | o | | | o | | | o |
| sushi domain-containing protein 2-like | 7 | | | | o | | | | |
| synaptic 2 | 7 | o | o | o | o | o | | | |
| synaptic vesicle glycoprotein 2a | 7 | | | | | | o | o | |
| syntaxin binding protein 1 | 7 | | | | | o | o | o | |
| t cell receptor alpha chain | 7 | o | | | | | | | |
| t lymphocyte 1 antigen-like protein | 7 | o | | | | o | o | o | o |
| tenascin c | 7 | o | | o | | | | | |
| tetratricopeptide repeat protein 35 | 7 | o | o | o | | o | o | | o |
| thioredoxin domain-containing protein 9 | 7 | o | o | o | | o | | o | o |
| toll-like receptor 3 | 7 | o | o | o | o | | | | |
| trafficking protein particle complex subunit 6b | 7 | o | o | | | o | o | o | |
| transcription initiation factor iib | 7 | o | o | o | o | o | | | |
| translocase of outer mitochondrial membrane 20 homolog | 7 | o | o | o | | o | | | |
| transmembrane and coiled-coil domains 1 | 7 | o | o | o | o | o | | o | |

| | | | | | | | | | |
|---|---|---|---|---|---|---|---|---|---|
| transmembrane protein 85 | 7 | o | o | o | | o | o | o | |
| transmembrane protein 93 | 7 | o | | o | | o | | o | o |
| tyrosine 3-monooxygenase tryptophan 5-monooxygenase activation epsilon polypeptide | 7 | o | o | o | | | o | o | |
| u2 small nuclear ribonucleoprotein b | 7 | o | | o | o | o | o | o | o |
| u3 small nucleolar ribonucleoprotein protein imp4 | 7 | o | | o | o | o | | o | |
| ubiquitin domain-containing protein ubfd1 | 7 | o | o | o | | o | o | o | |
| ubiquitin protein ligase e3a | 7 | o | o | | | | o | | |
| ubiquitin-conjugating enzyme e2n | 7 | o | | o | | o | o | o | |
| udp-glucose 4-epimerase | 7 | o | o | o | o | | | | o |
| ufm1-specific protease 2 | 7 | o | o | o | o | o | | | |
| uncharacterized transposon-derived | 7 | | o | | | | o | | |
| upf0472 protein c16orf72 homolog | 7 | o | o | o | o | | | o | |
| upf0585 protein c16orf13 homolog | 7 | o | | | | o | o | o | o |
| vacuolar atpase assembly integral membrane protein vma21 | 7 | o | o | o | | o | | o | o |
| vacuolar fusion protein ccz1 homolog | 7 | o | o | o | | o | | | o |
| vacuolar protein sorting-associated protein 26a | 7 | o | o | o | o | o | | o | |
| valosin containing protein | 7 | o | o | o | | | | | o |
| vascular endothelial growth factor | 7 | o | | | | o | o | o | o |
| von willebrand factor type egf and pentraxin domain-containing protein 1 | 7 | | | | o | | | | |
| v-type proton atpase subunit brain isoform-like | 7 | | | | | | | | o |
| weakly inward rectifying potassium channel | 7 | o | | | | | o | | |
| zinc c2h2 type family protein | 7 | o | o | o | | | o | o | |
| zinc finger protein 183 | 7 | o | | o | o | o | | o | |
| zinc finger protein 259 | 7 | o | o | o | | o | | o | o |
| zinc finger protein 462 | 7 | o | | | o | | o | o | |
| zinc finger protein 521 | 7 | o | | | | o | o | o | |
| zinc finger protein 706 | 7 | o | o | o | | o | | o | |
| zinc finger protein 865 | 7 | o | | | | | o | o | |
| zinc finger rna binding protein | 7 | o | o | o | | | o | o | |
| zinc imprinted 3 | 7 | o | | | | | | | |
| 14-3-3 protein zeta | 6 | o | o | o | | | o | o | |
| 17-beta-hydroxysteroid dehydrogenase 14 | 6 | o | o | o | | | | | |
| 26s proteasome non-atpase regulatory subunit 10 | 6 | o | | o | | o | | o | |
| 26s proteasome non-atpase regulatory subunit 14 | 6 | o | o | o | | | | o | o |
| 26s proteasome non-atpase regulatory subunit 6 | 6 | | o | | | o | | o | o |
| 3 (2 ) -bisphosphate nucleotidase 1 | 6 | o | o | o | o | o | | o | |
| 40s ribosomal protein s14 | 6 | | o | | o | | | | o |
| 40s ribosomal protein s25 | 6 | o | o | o | o | | o | o | |
| 60s ribosomal export protein nmd3 | 6 | o | o | | o | o | | | |
| 60s ribosomal protein l14 | 6 | o | | o | o | o | | o | |
| 60s ribosomal protein l31 | 6 | o | o | o | | o | o | o | |
| actin-binding homolog 2 | 6 | o | | | | | | | |
| acyl- -binding protein | 6 | | o | o | o | | o | o | o |
| acyl carrier mitochondrial precursor | 6 | o | o | o | | o | o | o | |
| acyl-coenzyme a short branched chain | 6 | o | o | o | | o | o | o | |
| adaptor-related protein complex mu 1 subunit | 6 | o | o | o | | | | o | |
| adaptor-related protein complex sigma 1 subunit | 6 | o | o | o | | o | | o | |
| adducin 3 | 6 | o | | | | | | o | |
| adenine phosphoribosyltransferase | 6 | o | | o | o | o | | o | |

| | | | | | | | | | |
|---|---|---|---|---|---|---|---|---|---|
| adp-ribosylation factor-like protein 8b | 6 | o | | o | o | | o | o | |
| adp-sugar pyrophosphatase | 6 | o | o | o | o | o | | | |
| aflatoxin b1 aldehyde reductase member 2 | 6 | o | o | o | o | | | | |
| ahnak nucleoprotein | 6 | o | | o | | o | | | |
| ahsg protein | 6 | o | o | o | o | | | | |
| aldehyde dehydrogenase family 9 member a1-a | 6 | o | | o | o | | | o | o |
| alpha-2 macroglobulin-like protein | 6 | | o | o | | | | | |
| alpha-2-macroglobulin-like 1 | 6 | o | o | o | | o | | o | |
| alpha-type globin | 6 | o | o | | | o | o | | |
| ambp protein precursor | 6 | | | | o | | | | o |
| apoptosis inhibitor 5 | 6 | | o | | o | | | | o |
| aquaporin 1 | 6 | o | | | | o | | | o |
| arf-gap with sh3 ank repeat and ph domain-containing protein 1 | 6 | o | | | | o | o | o | o |
| asparaginyl-trna cytoplasmic | 6 | o | o | o | | | | o | |
| ataxin-7-like protein 3 | 6 | | | o | o | o | o | o | |
| autophagy-related protein 101 | 6 | o | o | o | | o | | o | |
| b-cell cll lymphoma 7b | 6 | o | | o | | o | | o | o |
| bet1 homolog | 6 | o | o | o | | o | | o | |
| bifunctional aminoacyl-trna synthetase | 6 | o | o | | | o | | o | |
| bifunctional purine biosynthesis protein purh | 6 | | o | | o | o | | | o |
| brix domain-containing protein 1 | 6 | o | o | o | o | o | | o | |
| bromodomain containing 2 | 6 | o | o | o | | | o | o | |
| bsd domain containing 1 | 6 | o | o | o | | | | | o |
| butyrophilin-like 3 | 6 | o | | | | | | | |
| c12orf62 homolog | 6 | o | o | o | | o | o | o | |
| calcineurin subunit b type 1 | 6 | o | | | | | o | o | |
| calcium and integrin-binding protein 1 | 6 | o | o | o | | o | | | o |
| calretinin | 6 | | | | | o | | o | o |
| camp responsive element binding protein 1 | 6 | o | | o | | | | o | |
| canopy 2 homolog | 6 | o | o | o | | o | | | |
| carbonic anhydrase | 6 | o | o | | | o | | o | o |
| carboxyl ester lipase | 6 | o | o | | o | | | | |
| carboxymethylenebutenolidase homolog | 6 | o | o | o | | o | | | |
| carboxy-terminal domain rna polymerase ii polypeptide a small phosphatase 1 | 6 | o | | o | | | | o | |
| carnitine palmitoyltransferase 1a | 6 | o | o | | | | | | o |
| casein kinase alpha 1 | 6 | o | o | | | | | | o |
| cd63-like protein | 6 | o | | | | | | | |
| cdgsh iron sulfur domain-containing protein 1 | 6 | o | o | o | | o | | o | |
| cell division cycle 42 (gtp binding 25kda) | 6 | o | o | | | | o | o | o |
| cell division cycle protein 23 homolog | 6 | o | | | o | | | | o |
| cell growth-regulating nucleolar protein | 6 | o | | o | o | | o | o | |
| centromere kinetochore protein zw10 homolog | 6 | o | o | o | o | | | | o |
| charged multivesicular body protein 5 | 6 | o | o | o | o | o | o | | |
| chloride intracellular channel 4 | 6 | o | | | | | o | o | |
| chromosome 19 open reading frame 10 | 6 | o | | o | | | | | |
| chromosome 20 open reading frame 30 | 6 | o | o | o | | o | o | o | |
| chromosome 7 open reading frame 59 | 6 | o | o | o | | o | o | o | |
| chromosome 8 open reading frame 40 | 6 | o | o | o | | o | | o | |
| cl012 protein | 6 | o | | | | o | | | o |
| coagulation factor x | 6 | o | o | | o | o | | | |
| coatomer subunit alpha | 6 | | o | o | | o | | o | o |
| coiled-coil domain-containing protein 124 | 6 | o | o | o | | o | | o | o |
| complement factor i | 6 | | o | o | o | | | | |
| connector enhancer of kinase suppressor of ras 2 | 6 | | | | | | o | o | |
| coxsackie virus and adenovirus receptor | 6 | o | | o | | | | | |

| | | | | | | | | | |
|---|---|---|---|---|---|---|---|---|---|
| cullin 3 | 6 | o | | o | | o | | | o |
| cxxc finger 5 | 6 | o | | o | o | o | o | o | |
| cypher zasp splice variant 3 alpha | 6 | o | | | | | | | |
| cystathionine beta-synthase | 6 | o | o | o | | | | o | o |
| cysteine-rich protein 1 | 6 | o | | o | | o | | o | |
| cytochrome b5 | 6 | o | o | o | | | | o | |
| cytochrome b-c1 complex subunit 9 | 6 | o | o | o | o | o | | o | |
| cytochrome c oxidase polypeptide mitochondrial precursor | 6 | o | o | | | o | o | | |
| cytochrome c oxidase subunit iii | 6 | o | o | o | | o | o | | o |
| cytochrome c oxidase subunit vic | 6 | o | o | o | | o | o | o | |
| cytochrome c-1 | 6 | o | o | | o | | o | | o |
| cytochrome p450 3a | 6 | | o | o | | o | | | |
| cytoskeleton associated protein 5 | 6 | o | | | | | | o | |
| dcn1-like protein 1 | 6 | o | o | | o | | | o | o |
| dead (asp-glu-ala-asp) box polypeptide 21 | 6 | o | o | o | o | | | | |
| dead (asp-glu-ala-asp) box polypeptide 27 | 6 | | o | o | o | | o | | |
| dead (asp-glu-ala-asp) box polypeptide 6 | 6 | o | o | | | o | o | o | |
| dehydrogenase reductase sdr family member 1 | 6 | o | o | | | o | | o | o |
| deoxyribonuclease i | 6 | o | o | | o | o | o | | |
| dermal papilla-derived protein 6 homolog | 6 | o | | | o | | | | |
| diamine acetyltransferase 2 | 6 | o | o | o | | | | o | |
| dihydrolipoyllysine-residue succinyltransferase component of 2-oxoglutarate dehydrogenase mitochondrial | 6 | o | o | | | | | o | |
| dna-(apurinic or apyrimidinic site) lyase | 6 | o | | | | o | o | o | |
| dna-directed rna polymerases and iii subunit rpabc2 | 6 | o | o | o | | o | | o | o |
| dolichyl-diphosphooligosaccharide-protein glycosyltransferase | 6 | o | o | o | | | o | | |
| double-strand-break repair protein rad21 homolog | 6 | o | o | | o | | o | | o |
| down syndrome cell adhesion molecule | 6 | o | | | | o | o | | |
| dpy30 domain containing 1 | 6 | o | | | | | | | |
| dynactin subunit 2 | 6 | o | o | o | o | | | o | o |
| dynamin 2 | 6 | o | | o | | | o | o | |
| ectonucleotide pyrophosphatase phosphodiesterase 2 | 6 | | o | o | o | | o | o | |
| elongation factor 1-beta | 6 | o | o | o | o | o | | | |
| elongation of very long chain fatty acids protein 1 | 6 | o | o | o | o | | | o | |
| endoplasmin precursor | 6 | | o | o | o | o | | o | |
| endou protein | 6 | o | | o | | o | | | |
| enoyl- mitochondrial precursor | 6 | o | o | | o | o | | | o |
| es1 protein mitochondrial-like | 6 | | o | | o | | | | |
| eukaryotic translation elongation factor 2 | 6 | o | | | o | o | o | o | |
| eukaryotic translation initiation factor 1b | 6 | o | o | o | | o | | o | o |
| eukaryotic translation initiation factor 3 subunit 3 | 6 | | o | o | | o | o | o | o |
| eukaryotic translation initiation factor 3 subunit a | 6 | o | | o | o | | o | | |
| eukaryotic translation initiation factor 3 subunit b | 6 | o | o | o | | o | | | o |
| eukaryotic translation initiation factor 3 subunit d | 6 | o | o | o | | o | o | | |
| eukaryotic translation initiation factor 4e type 3 | 6 | o | o | o | | o | | | |
| eukaryotic translation initiation factor 5a | 6 | o | o | o | | | | | o |
| fact complex subunit ssrp1 | 6 | | o | | o | o | o | o | |
| fam32a-like | 6 | o | o | o | | o | | o | |

| | | | | | | | | | |
|---|---|---|---|---|---|---|---|---|---|
| fbxo11 protein | 6 | o | o | | o | | o | | |
| fintrim family protein | 6 | o | | o | | o | | o | |
| flavin containing monooxygenase 5 | 6 | o | o | o | | | | | |
| four and a half lim domains 2 | 6 | o | | | | | | | |
| g patch domain-containing protein 8 | 6 | o | | o | | o | o | o | |
| g protein pathway suppressor 1 | 6 | | o | | o | o | | | |
| g2 m phase-specific e3 ubiquitin-protein ligase | 6 | o | | | | o | | | |
| galectin-3-binding protein precursor | 6 | o | | o | o | | | o | o |
| gamma-aminobutyric acid receptor subunit alpha-1-like | 6 | | | | | | o | | o |
| gf20795 | 6 | o | | | | o | | o | |
| glioblastoma amplified sequence | 6 | o | o | | | | | | o |
| glutamate ampa 2a | 6 | | | | | | o | o | |
| glutamate dehydrogenase | 6 | o | o | o | | | | | o |
| glutathione s-transferase mu 3 | 6 | o | o | o | | o | o | | o |
| glutathione transferase omega-1 | 6 | o | o | o | | o | | o | |
| growth arrest and dna-damage-inducible protein gadd45 beta | 6 | | o | | o | | | | |
| gtp binding protein 4 | 6 | o | o | o | | | | | |
| gtp-binding nuclear protein ran | 6 | | | | o | o | | | o |
| guanidinoacetate n-methyltransferase | 6 | o | o | o | o | o | o | | |
| h+ lysosomal accessory protein 1 | 6 | o | o | | | o | | | o |
| heat shock factor-binding protein 1 | 6 | o | o | o | | o | o | o | |
| heat shock protein 4 | 6 | | o | o | o | o | | o | |
| heat shock protein 67b2 | 6 | o | o | o | | o | | o | |
| hepatocyte growth factor-regulated tyrosine kinase substrate | 6 | o | o | | o | | o | | |
| hepcidin | 6 | o | o | o | o | o | | | |
| heterogeneous nuclear ribonucleoprotein g | 6 | o | o | | o | | o | o | |
| heterogeneous nuclear ribonucleoprotein l-like | 6 | o | | | | | o | o | o |
| histidine triad nucleotide binding protein 2 | 6 | o | | o | o | o | | | |
| histidine triad nucleotide-binding protein 2 | 6 | o | o | o | | | | o | |
| histone deacetylase 1 | 6 | o | o | | | | | o | o |
| hla-b associated transcript 3 | 6 | o | | | | | o | o | o |
| host cell factor 1 | 6 | o | | o | | | | o | |
| hrc protein | 6 | o | | | | | | | |
| hydroxysteroid (17-beta) dehydrogenase 8 | 6 | o | o | | o | o | | | o |
| immunoglobulin light chain precursor | 6 | o | | o | | o | o | | |
| inorganic pyrophosphatase | 6 | o | o | o | | o | | o | |
| inositol oxygenase | 6 | | o | | o | | | | |
| insulin receptor substrate 2 | 6 | o | o | o | | | o | | |
| interleukin 2 gamma (severe combined immunodeficiency) | 6 | o | | | | | | | |
| isocitrate dehydrogenase 2 (nadp+) mitochondrial | 6 | o | o | o | | | o | | o |
| kda mitochondrial proteolipid-like | 6 | o | o | o | o | o | | | |
| keratinocyte associated protein 2 | 6 | o | o | o | | o | o | o | |
| latexin | 6 | o | o | o | o | o | | | |
| linker histone h1m | 6 | | | | o | | | | |
| loc559122 protein | 6 | | | | o | | | | |
| macrophage migration inhibitory factor | 6 | o | o | o | | o | o | o | |
| mannan-binding lectin serine protease 1 | 6 | o | o | o | | | | o | |
| mannose c type 1 | 6 | o | | o | | | | | |
| map microtubule affinity-regulating kinase 3-like | 6 | | o | | o | o | | o | |
| melanoma cell adhesion molecule | 6 | o | | | | | | | |
| methionine aminopeptidase 2 | 6 | | | | | | o | | o |
| mgc81154 protein | 6 | o | | o | | | | o | |

| | | | | | | | | | |
|---|---|---|---|---|---|---|---|---|---|
| microtubule associated calponin and lim domain containing 3 | 6 | o | o | | | | o | o | |
| mitochondrial carrier homolog 2 | 6 | o | o | o | | | | | o |
| mitochondrial elongation factor g | 6 | o | o | | | | | o | |
| mitochondrial import inner membrane translocase subunit tim13 | 6 | o | o | o | | o | | | |
| mitochondrial import inner membrane translocase subunit tim17-a | 6 | o | o | o | | o | | | o |
| mitochondrial import inner membrane translocase subunit tim9 | 6 | o | o | o | | o | o | o | |
| mitochondrial import receptor subunit tom7 homolog | 6 | o | o | o | | o | o | o | |
| mitochondrial leucine-rich ppr motif-containing protein | 6 | o | | | | | o | o | o |
| mitochondrial ribosomal protein l15 | 6 | o | o | o | | o | | o | |
| mitochondrial ribosomal protein l19 | 6 | o | o | o | | o | | | |
| mitochondrial ribosomal protein s18a | 6 | o | o | o | | o | | o | |
| mitochondrial ribosomal protein s7 | 6 | o | o | | o | o | o | o | |
| mitogen-activated protein kinase kinase kinase 7 | 6 | o | | | | | o | | |
| mlx interacting protein | 6 | o | o | o | | | | o | |
| motile sperm domain containing 2 | 6 | o | o | o | | | | o | |
| muscle-related coiled-coil protein | 6 | o | | | | | | | |
| myomesin- partial | 6 | o | | | | | | | |
| myotubularin related protein 4 | 6 | | o | | | | o | | |
| na-k-cl cotransporter | 6 | | | | o | | | | o |
| nck-associated protein 1 | 6 | o | o | | | | | o | |
| neighbor of cox4 | 6 | o | o | o | | | | | |
| neuron navigator 1 | 6 | o | | | | o | o | o | |
| nipped-b homolog | 6 | o | o | | | o | | | |
| nktr protein | 6 | o | | o | | | o | o | |
| n-myristoyltransferase 1 | 6 | o | o | o | | | o | o | |
| novel gene | 6 | o | | | | | | | |
| novel protein vertebrate complement component 3 | 6 | | o | o | o | | | | |
| novel protein vertebrate muscle derived protein | 6 | o | | | | | | | |
| novel protein vertebrate pleckstrin homology-like family member 1 | 6 | o | | | | | o | | |
| novel protein vertebrate stabilin family | 6 | | | | | | | | o |
| n-terminal acetyltransferase complex ard1 subunit homolog a | 6 | o | o | o | o | o | | | o |
| nuclear autoantigenic sperm protein | 6 | | | | o | | | | |
| nuclear factor 1 x-type | 6 | o | | o | | | o | | |
| nucleosome assembly protein 1-like 1 | 6 | o | o | o | o | o | | | |
| ocia domain-containing protein 1 | 6 | o | o | o | o | o | | o | |
| orm1-like protein 2 | 6 | o | | o | o | o | | o | o |
| peptide methionine sulfoxide reductase | 6 | o | o | o | | o | | | o |
| peptidyl arginine type iv | 6 | o | | | | | | | |
| peptidylprolyl isomerase like | 6 | o | o | | | o | | o | o |
| peptidylprolyl isomerase -like 4 | 6 | o | o | | o | o | | | |
| peripheral myelin protein 22 | 6 | o | o | o | | | | | |
| peroxisomal membrane protein 22kda | 6 | o | o | o | | o | | | |
| phenylalanyl-trna synthetase alpha chain | 6 | o | o | o | | | | o | o |
| phosducin-like protein | 6 | o | | o | | o | | | o |
| phosducin-like protein 3 | 6 | o | o | o | | o | | o | |
| phosphate carrier mitochondrial precursor | 6 | o | o | o | | o | | | |
| phosphatidic acid phosphatase type 2b | 6 | o | | | | | o | o | |
| phosphatidic acid phosphatase type 2c | 6 | o | | | | o | | o | o |
| phosphoglycerate kinase 1 | 6 | o | o | | o | | | | o |

| | | | | | | | | | |
|---|---|---|---|---|---|---|---|---|---|
| phospholipase a-2-activating protein | 6 | o | | | | | o | | o |
| phospholysine phosphohistidine inorganic pyrophosphate phosphatase | 6 | o | | | | o | | o | o |
| phosphopantothenoylcysteine decarboxylase | 6 | o | | o | | o | | o | |
| pleiotrophic factor-alpha-2 precursor | 6 | | | | | o | o | o | o |
| plexin b2 | 6 | o | o | | | | o | | |
| poly -binding-splicing factor puf60 | 6 | | o | o | | o | | o | o |
| polyprotein [Atlantic salmon swim bladder sarcoma virus] | 6 | o | | | | o | | o | |
| ppe family protein | 6 | | | o | o | | | o | o |
| prefoldin subunit 2 | 6 | o | o | o | | | o | o | o |
| prefoldin subunit 5 | 6 | o | o | o | o | o | | o | |
| prefoldin subunit 6 | 6 | o | | o | | o | o | o | |
| probable carboxypeptidase pm20d1 precursor | 6 | o | o | | | o | | o | |
| probable protein brick1 | 6 | o | o | o | o | o | | o | |
| programmed cell death protein 5 | 6 | o | | o | o | o | o | o | |
| prohibitin | 6 | o | o | | o | | | | o |
| proteasome ( macropain) 26s non- 13 | 6 | o | o | o | | o | | o | o |
| proteasome ( macropain) 26s non- 2 | 6 | o | o | | | o | | o | |
| proteasome assembly chaperone 4-like | 6 | o | o | o | o | o | | | |
| proteasome subunit alpha type-5 | 6 | o | o | o | | o | | o | o |
| proteasome subunit beta type-4 precursor | 6 | o | o | o | | o | | | o |
| proteasome subunit beta type-5 precursor | 6 | o | o | | | o | | | o |
| protein bud31 homolog | 6 | o | o | o | o | o | | o | |
| protein ccsmst1 | 6 | o | o | o | | o | | o | |
| protein fam168a-like | 6 | o | | | | o | o | o | |
| protein isoform 2 | 6 | o | | o | | o | | o | |
| protein kinase c and casein kinase substrate in neurons 2 | 6 | o | o | | | | | o | |
| protein kinase c inhibitor aswz variant 5 | 6 | o | o | o | | o | o | o | |
| protein kinase c substrate 80k-h | 6 | o | | o | | o | | o | |
| protein phosphatase 1 regulatory subunit 3a | 6 | o | | | | | | | |
| protein-cysteine n-palmitoyltransferase hhat-like protein | 6 | o | | | | | | o | |
| protocadherin beta-16-like | 6 | | | | | | o | | |
| pyruvate dehydrogenase isoenzyme 2 | 6 | o | o | | | | o | | o |
| rab-like protein 3 | 6 | o | o | o | | o | | o | |
| ral guanine nucleotide dissociation stimulator-like 1 | 6 | o | | | | | | o | |
| ras-related protein rab-6b | 6 | o | | | | o | o | o | o |
| rcg47273-like | 6 | o | o | o | | o | o | o | |
| regulator of microtubule dynamics protein 1 | 6 | o | o | o | | o | | | |
| related ras viral (r-ras) oncogene homolog 2 | 6 | o | | o | | o | o | o | |
| repressor of rna polymerase iii transcription maf1 homolog | 6 | o | o | o | | | | | o |
| response gene to complement 32 protein | 6 | o | | o | | | | o | |
| retinol dehydrogenase 12 | 6 | o | | o | o | o | | | o |
| retinol dehydrogenase 3 | 6 | | o | | o | | | o | |
| rho gtpase-activating protein 32 | 6 | | | | | | o | | |
| ribonucleoside-diphosphate reductase large subunit | 6 | o | o | | o | o | | | |
| ribosomal protein l11 | 6 | o | o | o | | o | | o | o |
| ribosomal protein l15 | 6 | o | o | o | | o | | | o |
| ribosomal protein l22 | 6 | o | o | o | | o | | o | |
| ribosomal protein l35a | 6 | o | o | o | o | o | | | o |
| ribosomal protein l7-like 1 | 6 | o | o | o | o | o | | o | |
| ribosomal protein s17 | 6 | o | o | o | | o | | o | |
| ribosomal protein sa | 6 | o | o | o | | o | o | | o |
| ribulose-phosphate 3-epimerase | 6 | o | o | o | | o | | o | o |
| ring finger 1 | 6 | o | | | | | o | o | |

| | | | | | | | | | |
|---|---|---|---|---|---|---|---|---|---|
| ring finger protein 10 | 6 | o | o | o | | | | | |
| ring-box protein 1 | 6 | o | o | o | o | o | | o | |
| rna binding motif protein 25 | 6 | o | o | o | | o | | o | o |
| rna binding motif protein 38 | 6 | o | | | | | | | |
| rna binding protein with multiple splicing 2 | 6 | o | | o | | o | o | o | |
| rna polymerase ii subunit a c-terminal domain phosphatase ssu72 | 6 | o | o | | | o | o | o | o |
| run and fyve domain containing 3 | 6 | | | | | o | | o | o |
| sarcolemma associated protein | 6 | o | | | | | | o | o |
| sec14-like 1 ( cerevisiae) | 6 | o | o | | o | | | | |
| secretagogin | 6 | o | | | o | o | | o | o |
| selenoprotein 1 | 6 | o | o | o | | o | | o | |
| selenoprotein 2a | 6 | o | o | o | | | o | o | |
| senescence-associated protein | 6 | | o | o | | o | o | | |
| serine hydrolase-like 2 | 6 | o | o | o | | | | | o |
| serine threonine-protein kinase pim-2-like | 6 | | o | | o | | o | | |
| short coiled-coil protein | 6 | o | | | o | | | o | o |
| signal peptidase complex catalytic subunit sec11a | 6 | o | o | o | | o | | o | o |
| signal peptide peptidase-like 2a | 6 | o | | o | | o | | | |
| signal recognition particle receptor subunit beta | 6 | o | o | o | o | o | | | |
| small acidic protein | 6 | o | o | | o | o | | | o |
| small nuclear ribonucleoprotein polypeptide a | 6 | o | o | o | | o | | o | |
| solute carrier family 43 member 3 | 6 | o | o | o | | | | | |
| spartin | 6 | | o | o | o | | | o | |
| splicing factor 3a subunit 3 | 6 | o | | | o | | | | o |
| split hand foot malformation type 1 | 6 | o | o | o | | o | o | o | |
| sra stem-loop-interacting rna-binding mitochondrial precursor | 6 | o | o | o | | o | o | o | |
| srp72 protein | 6 | o | o | o | o | o | | | |
| sterol carrier protein 2 | 6 | o | o | | | o | | o | |
| sterol-c4-methyl oxidase-like | 6 | o | o | o | o | o | | o | |
| subfamily member 1 | 6 | o | o | o | | | | o | |
| subfamily member 12 | 6 | o | o | o | | | | | o |
| subfamily member 4 | 6 | o | | o | | | | | |
| t-cell receptor type 1 | 6 | o | | | | | | | |
| thap domain-containing protein 9 | 6 | o | | o | | o | | | |
| thioredoxin-dependent peroxide mitochondrial precursor | 6 | | o | o | o | o | | | |
| thioredoxin-like 1 | 6 | o | o | o | | o | | o | |
| threonyl-trna cytoplasmic | 6 | | o | | o | | | | o |
| trafficking protein particle complex subunit 3 | 6 | o | o | o | | o | | o | o |
| trans- -dihydrobenzene- -diol dehydrogenase | 6 | o | | | o | o | | | |
| transcobalamin-2 precursor | 6 | | o | | o | | | o | o |
| transcription elongation factor 1 homolog | 6 | o | | | | o | | o | |
| transcription elongation factor a 1 | 6 | o | o | o | | o | | o | |
| transcription elongation factor b polypeptide 1 | 6 | o | o | o | | o | o | o | |
| transcription initiation factor tfiid subunit 11 | 6 | o | o | o | o | o | | | |
| transforming protein precursor | 6 | o | o | o | | | o | o | |
| transketolase | 6 | | o | | o | o | o | o | |
| translocase of outer mitochondrial membrane 70 homolog a | 6 | o | o | | | o | o | o | |
| translocation protein sec63 homolog | 6 | o | o | o | | | o | | |
| translocator protein | 6 | o | o | o | o | o | | o | |
| transmembrane emp24 domain-containing protein 9 precursor | 6 | o | o | o | | | | | |
| transmembrane protein 14c | 6 | o | o | | | o | o | o | |
| transmembrane protein 35 | 6 | | | | o | o | o | o | o |

| | | | | | | | | | |
|---|---|---|---|---|---|---|---|---|---|
| transmembrane protein 87b | 6 | o | o | o | | | o | | |
| triadin | 6 | o | | | | | | | |
| trna selenocysteine 1-associated protein 1 | 6 | o | o | o | | o | o | | |
| tubulin beta-2b chain-like | 6 | o | | | | | o | o | |
| tubulin-specific chaperone a | 6 | o | | o | o | o | | | |
| u6 snrna-associated sm-like protein lsm5 | 6 | o | o | o | | o | o | o | |
| ubiquinol-cytochrome c reductase core protein i | 6 | o | o | o | | | o | | |
| ubiquitin carboxyl-terminal hydrolase l5 | 6 | o | o | o | o | o | | o | |
| ubiquitin-associated protein 2 | 6 | o | o | | | o | o | | o |
| ubiquitin-conjugating enzyme e2r 2 | 6 | o | o | o | | | | | |
| ubiquitin-fold modifier 1 | 6 | o | o | o | | o | o | o | |
| up-regulated during skeletal muscle growth protein 5-like | 6 | o | | o | | o | | o | |
| vacuolar protein sorting-associated protein 41 homolog | 6 | o | o | | o | o | | o | |
| vacuolar proton pump subunit d | 6 | | | o | | | | o | o |
| v-crk sarcoma virus ct10 oncogene homolog -like | 6 | o | | o | | | o | o | |
| very long-chain acyl- synthetase | 6 | o | o | o | | | | | o |
| v-ki-ras2 kirsten rat sarcoma viral oncogene homolog | 6 | o | | | | o | | o | o |
| voltage-dependent anion channel 1 | 6 | o | o | | o | | | | o |
| voltage-dependent anion channel 3 | 6 | o | o | o | | | | o | o |
| voltage-dependent anion-selective channel protein 2 | 6 | | o | | o | | | | o |
| wash complex subunit strumpellin | 6 | o | o | | | | o | o | |
| wd repeat-containing protein 82 | 6 | o | o | | | o | | o | o |
| ww domain binding protein 2 | 6 | | o | o | | | | | o |
| yip1 domain member 1 | 6 | o | o | o | | | | | |
| yy1 transcription factor | 6 | o | | o | | o | o | o | |
| zinc finger and btb domain containing 8 opposite strand | 6 | o | o | | | o | | o | o |
| zinc finger protein 452 | 6 | o | | | | o | | o | |
| zinc finger protein 568 | 6 | o | | | | | | | |
| zinc finger ran-binding domain-containing protein 2 | 6 | o | | | | o | o | | |
| zona pellucida sperm-binding protein 4-like | 6 | | | | o | | | | |
| 15 kda selenoprotein precursor | 5 | o | o | o | | | | o | |
| 15-hydroxyprostaglandin dehydrogenase | 5 | o | o | o | o | | | | o |
| 1-phosphatidylinositol-3-phosphate 5-kinase | 5 | o | | o | | | | | o |
| 26s protease regulatory subunit 6a | 5 | o | o | o | | | | | o |
| 28s ribosomal protein mitochondrial | 5 | | o | | o | | | | o |
| 2-hydroxyacylsphingosine 1-beta-galactosyltransferase | 5 | o | | | | | o | o | o |
| 39s ribosomal protein mitochondrial-like | 5 | | | | o | | | | o |
| 3-hydroxyanthranilate -dioxygenase | 5 | | o | o | | | o | | |
| 3-hydroxybutyrate dehydrogenase type 2 | 5 | o | o | o | | o | | | |
| 3-ketoacyl- mitochondrial | 5 | o | o | o | o | | | o | |
| 3-oxo-5-beta-steroid 4-dehydrogenase | 5 | | o | o | | | | | |
| 3-oxoacid transferase 1 | 5 | o | | | | | | | o |
| 3-phosphoinositide dependent protein kinase-1 | 5 | o | o | | | | o | o | |
| 40s ribosomal protein s13 | 5 | | | | o | | | | |
| 40s ribosomal protein s15a | 5 | o | o | o | | o | | o | |
| 40s ribosomal protein s7 | 5 | | | | o | | | | o |
| 4-aminobutyrate aminotransferase | 5 | o | o | o | | | o | | |
| 5-formyltetrahydrofolate cyclo-ligase | 5 | o | o | o | o | | | | |
| 6-phosphofructokinasemuscle type | 5 | o | | | | | | o | |
| 60 kda lysophospholipase | 5 | o | | o | | | | o | |

| | | | | | | | | | |
|---|---|---|---|---|---|---|---|---|---|
| 60s ribosomal protein l11 | 5 | | | | o | | | | |
| 60s ribosomal protein l30 | 5 | o | o | o | | o | | o | |
| 60s ribosomal protein l36 | 5 | o | o | o | | o | | | |
| 60s ribosomal protein l7a | 5 | o | | | o | | | | o |
| 60s ribosome subunit biogenesis protein nip7 homolog | 5 | o | | o | o | o | | | |
| 6-phosphofructo-2-kinase fructose- -biphosphatase 1 | 5 | o | o | | | | | | |
| 6-pyruvoyl tetrahydrobiopterin synthase | 5 | o | o | | | o | | | |
| acetoacetyl- synthetase | 5 | | o | | o | | | o | o |
| actin-related protein 2 3 complex subunit 3 | 5 | o | o | o | | o | | o | |
| activating transcription factor 4 | 5 | o | o | | | o | o | | o |
| activator of 90 kda heat shock protein atpase homolog 1 | 5 | o | o | | o | | | | o |
| active bcr-related gene | 5 | o | | | | | o | o | |
| acyl- dehydrogenase | 5 | o | o | o | | o | | | |
| acyl- desaturase | 5 | o | o | | | | o | | o |
| adenomatosis polyposis coli 2 | 5 | | | | | o | o | o | |
| adenylosuccinate synthetase isozyme 2 | 5 | | o | o | o | | | | o |
| alanine--glyoxylate aminotransferase 2-like 1 | 5 | | o | o | | | | | |
| alcohol dehydrogenase | 5 | | o | o | o | | | | o |
| aldehyde dehydrogenase 2 family | 5 | o | o | o | | o | | | |
| alpha isoform cra_b | 5 | | | | | | | | o |
| alpha-enolase | 5 | o | | o | | | o | o | |
| aminopeptidase-like 1 | 5 | o | o | o | o | | | | |
| ancient ubiquitous protein 1 | 5 | o | o | o | o | | | | o |
| angiotensinogen precursor | 5 | | o | o | o | | | o | |
| ankyrin neuronal | 5 | o | | | | | o | o | |
| ankyrin repeat domain 11 | 5 | o | | | | | o | | |
| annexin a1 | 5 | o | | o | | o | | o | o |
| annexin a2 | 5 | o | | o | | o | | o | |
| antizyme inhibitor 1 | 5 | o | o | | | | o | | |
| apolipoprotein b-100 | 5 | | o | | | | o | | |
| apoptosis-related protein 3 precursor | 5 | o | o | o | | | | o | |
| arsa arsenite atp- homolog 1 | 5 | o | o | o | | | | o | |
| arsenite methyltransferase | 5 | o | o | | o | o | | | |
| asparagine-linked glycosylation 9 homolog ( alpha- -mannosyltransferase) | 5 | o | o | o | | o | | | |
| atp gtp binding protein 1 | 5 | o | | | | | o | o | |
| atp synthase subunit mitochondrial-like | 5 | | | | | | | | o |
| atp-binding cassette sub-family e member 1 | 5 | | | o | | | | | o |
| atp-binding sub-family e member 1 | 5 | o | o | o | | | | | |
| atp-dependent rna helicase ddx1 | 5 | | | | o | o | | | o |
| b chain reactivity modulation of human branched-chain alpha- ketoacid dehydrogenase by an internal molecular switch | 5 | o | | o | | | | o | o |
| bactericidal permeability-increasing protein | 5 | o | | o | | | | o | |
| baculoviral iap repeat-containing 2 | 5 | | o | o | o | | | | o |
| barrier-to-autointegration factor | 5 | o | o | | | | o | o | |
| basic leucine zipper and w2 domain-containing protein 1-like | 5 | | | | o | | | | o |
| bc003940 protein | 5 | o | | o | | o | o | o | |
| b-cell receptor-associated protein 29 | 5 | o | o | | | | | | |
| b-cell translocation gene 4 | 5 | o | | | o | | | | |
| b-cell translocation gene anti-proliferative | 5 | o | | | | | o | o | |
| beta lysosomal-like | 5 | o | | o | | o | o | o | |
| beta-galactosidase-1-like protein 2-like | 5 | | | | | | | | o |
| beta-microseminoprotein | 5 | | | | o | | | | |
| beta-sarcoglycan | 5 | o | o | o | | o | | | |
| brain protein 44 | 5 | o | o | o | | | | o | o |

| | | | | | | | | | |
|---|---|---|---|---|---|---|---|---|---|
| brain protein 44-like protein | 5 | o | o | o | | o | | o | |
| breast carcinoma amplified sequence 2 | 5 | o | o | o | | o | | o | |
| btb domain containing 1 | 5 | o | o | o | | | | o | |
| c15orf24 homolog precursor | 5 | o | o | o | | o | | o | |
| c19orf60 homolog | 5 | o | o | o | | o | o | | |
| calponin acidic | 5 | o | o | | o | o | | | |
| camp-regulated phosphoprotein 19 | 5 | o | | o | o | o | o | | |
| carboxypeptidase d | 5 | o | | o | | | | o | |
| carboxy-terminal domain rna polymerase ii polypeptide a small phosphatase 2 | 5 | o | o | o | | | | | |
| carnitine o-palmitoyltransferase liver isoform | 5 | o | o | | | | o | | |
| cartilage acidic protein 2 | 5 | o | | | o | o | | | |
| casein kinase alpha 1 polypeptide | 5 | o | o | | | | o | o | |
| casein kinase beta polypeptide | 5 | o | o | o | | o | | o | |
| cathepsin c | 5 | o | | o | o | | | | |
| caveolin 2 | 5 | o | o | | | | | | |
| caytaxin | 5 | o | | | | | | | o |
| ccr4-not transcription subunit 6-like | 5 | o | o | | | | | | |
| cd209 antigen-like protein a | 5 | o | o | o | | | | | |
| cd300 molecule-like family member d-like | 5 | o | | | | | | o | |
| cdna sequence bc003266 | 5 | o | | o | o | o | | o | |
| charged multivesicular body protein 2a | 5 | o | o | o | | | | | o |
| chk1 checkpoint-like protein | 5 | | o | o | | | o | o | |
| choline kinase isoform cra_b | 5 | o | | | o | | | | o |
| choline transporter-like protein 2 | 5 | o | o | | o | | | | |
| chromosome 12 open reading frame 51 | 5 | o | | | | | o | | |
| chromosome 16 open reading frame 57 | 5 | o | o | o | | | | | |
| chromosome 2 open reading frame 7 | 5 | o | o | o | | o | | | |
| chromosome 9 open reading frame 78 | 5 | o | o | o | | o | | | |
| chromosome 9 open reading frame 85 | 5 | o | | o | | o | | o | |
| claudin 19 | 5 | o | | o | | | o | o | o |
| cleft lip and palate associated transmembrane protein 1 | 5 | | o | | | | o | o | o |
| coagulation factor ix | 5 | | o | o | | | | | |
| coatomer protein subunit beta 2 (beta prime) | 5 | o | o | o | | o | | | |
| coenzyme q4 homolog | 5 | o | o | | | | o | | |
| coiled-coil domain-containing protein 115 | 5 | o | | o | o | | o | | |
| coiled-coil-helix-coiled-coil-helix domain containing 5 | 5 | o | o | | | o | o | o | |
| comm domain-containing protein 9 | 5 | o | | o | | o | | o | o |
| complement c4-like | 5 | | | | o | | | | |
| cop9 constitutive photomorphogenic homolog subunit 2 | 5 | o | | o | | o | | o | |
| c-src tyrosine kinase | 5 | o | | o | | | | | |
| c-type lectin | 5 | o | o | | | | | o | |
| cug triplet rna binding protein 2 | 5 | o | o | | | | | o | o |
| cullin 4b | 5 | o | o | o | | | | | |
| cwc15 homolog | 5 | | o | | o | | | | o |
| cxxc finger 1 (phd domain) | 5 | | o | | o | o | | | o |
| cyclin a2 | 5 | o | | | o | | | | |
| cyclin b2 | 5 | o | | | o | | | | |
| cyclophilin a | 5 | | o | o | | | o | | |
| cystathionine gamma-lyase | 5 | o | o | o | | | | o | |
| cystatin-b | 5 | o | o | | | o | o | o | |
| cytidine deaminase | 5 | o | o | o | | | | | |
| cytochrome b-c1 complex subunit 7 | 5 | o | o | | | o | o | o | |
| cytochrome b-c1 complex subunit 8 | 5 | o | o | | | o | o | o | |
| cytochrome c oxidase subunit ii | 5 | | o | o | | o | o | o | |
| cytochrome c subunit viic-like | 5 | o | o | o | | o | | o | |
| cytochrome p450 cyp2n | 5 | | o | o | | | | | |

| | | | | | | | | | |
|---|---|---|---|---|---|---|---|---|---|
| cytosolic sulfotransferase 3 | 5 | o | o | | o | o | | | |
| dci protein | 5 | o | o | o | | o | | | |
| dcn1-like protein 5 | 5 | o | o | | o | o | | | |
| ddb1- and cul4-associated factor 13 | 5 | o | o | o | | | | o | o |
| death-associated protein 1 | 5 | | o | o | o | | | o | o |
| defender against cell death 1 | 5 | o | o | o | | o | | o | |
| dehydrogenase reductase sdr family member 13 precursor | 5 | o | o | o | | | | | |
| delta and notch-like epidermal growth factor-related receptor-like | 5 | | | | | | | | o |
| density-regulated protein | 5 | o | | o | | o | | o | |
| deoxyribonuclease gamma precursor | 5 | o | o | | o | | | | |
| dihydrofolate reductase | 5 | | o | o | | o | | | |
| dihydrolipoamide branched chain transacylase e2 | 5 | o | o | | | | | | o |
| dimethylglycine dehydrogenase | 5 | | o | o | | | | | |
| dipeptidyl-peptidase 9 | 5 | o | o | o | | | | | |
| dmx-like 1 | 5 | o | | | | | o | | |
| dna photolyase | 5 | o | o | | | o | o | | |
| dna polymerase alpha subunit | 5 | o | o | o | | o | | | |
| dna replication licensing factor mcm4 | 5 | o | | | o | | | | |
| dna-directed rna polymerases and iii subunit rpabc4-like | 5 | o | | o | | o | o | o | |
| dolichol phosphate-mannose biosynthesis regulatory protein | 5 | o | o | o | | | | o | |
| dymeclin | 5 | o | | | | | | o | o |
| dynactin subunit 3 | 5 | o | o | o | | | | o | |
| e3 ubiquitin-protein ligase trim21-like | 5 | | | | | | o | | o |
| e3 ubiquitin-protein ligase ubr5 | 5 | o | o | | | | | | |
| early growth response 1 | 5 | o | | | o | | o | | |
| ectonucleoside triphosphate diphosphohydrolase 4 | 5 | o | o | o | | | | | o |
| electron-transfer- beta polypeptide | 5 | o | o | o | | | | | o |
| enhancer of yellow 2 transcription factor homolog | 5 | o | | | | o | | o | |
| enolase 3-2 | 5 | o | o | o | | o | | | |
| enolase-phosphatase e1 | 5 | o | o | o | | | | | |
| epithelial membrane protein 2 | 5 | o | o | | | o | | | |
| estradiol 17-beta-dehydrogenase 12-b | 5 | | o | o | | | | | o |
| eukaryotic initiation factor 4a-iii | 5 | o | o | | o | | | | |
| eukaryotic translation initiation factor 2 subunit 3-like | 5 | | | | o | | | | o |
| eukaryotic translation initiation factor 3 subunit h | 5 | o | | o | o | | | | |
| eukaryotic translation initiation factor 4e binding protein 1 | 5 | o | o | o | | o | | o | |
| eukaryotic translation initiation factor subunit 4 67kda | 5 | o | o | o | | | | | |
| eukaryotic translation initiation factor subunit 5 47kda | 5 | o | o | o | o | o | | | |
| exocyst complex component 6b | 5 | o | | | | | o | | |
| exosome complex exonuclease rrp4 | 5 | o | | | o | o | | | |
| exosome complex exonuclease rrp42 | 5 | o | | | o | o | o | | |
| exported repetitive protein | 5 | | | | o | | o | | |
| extracellular matrix protein 1-like | 5 | | o | | o | | | o | |
| far upstream element binding protein 1 | 5 | o | | | | | | o | |
| fasciculation and elongation protein zeta-1 | 5 | | | | | o | o | o | o |
| fatty acid synthase | 5 | | o | | | | | | |
| fatty acyl- reductase 1 | 5 | o | | | | | o | o | |
| fatty aldehyde dehydrogenase | 5 | o | o | o | | | | | |

| | | | | | | | | | |
|---|---|---|---|---|---|---|---|---|---|
| f-box only protein 31 | 5 | | o | | | | | o | o |
| f-box protein 9 | 5 | o | o | o | | o | | o | |
| fbp32ii precursor | 5 | o | | o | | o | | o | |
| fgfr1 oncogene partner | 5 | | | | o | | | | |
| fibrinogen gamma isoform cra_a | 5 | | o | | o | | o | | |
| fibroblast growth factor intracellular binding protein | 5 | o | | o | | o | o | | o |
| filamin a-interacting protein 1-like | 5 | o | | | | | o | o | |
| g patch domain containing 8 | 5 | o | o | | | | o | o | |
| gamma-enolase-like isoform 2 | 5 | | | | | | | | o |
| gamma-glutamyl hydrolase | 5 | o | o | o | o | o | | | |
| general transcription factor iih subunit 2 | 5 | o | | | | o | | | o |
| general transcription factor iih subunit 5 | 5 | o | o | o | | o | o | | |
| gethr pentapeptide repeat (5 copies) family | 5 | | | | o | | | | |
| glucagon family neuropeptides precursor | 5 | | | | | o | | o | o |
| glucocorticoid receptor | 5 | o | | | | | o | | o |
| glutamate metabotropic 3 | 5 | | | | | | o | o | |
| glutaminyl-trna synthetase | 5 | o | | o | o | | | | |
| glutathione peroxidase 1 | 5 | o | o | o | | o | | o | |
| glutathione peroxidase 4 | 5 | o | o | o | o | | | | |
| glycerol-3-phosphate dehydrogenase | 5 | o | o | o | | | | | |
| glycerophosphodiester phosphodiesterase domain containing 1 | 5 | | o | | | o | o | o | |
| glycine dehydrogenase | 5 | o | o | | | | | | |
| glycine receptor subunit beta | 5 | | | | | o | | | o |
| glycogenin-1 | 5 | | | o | o | o | o | | |
| glyoxalase domain-containing protein 4 | 5 | o | o | | | o | | o | |
| granulins precursor | 5 | o | o | o | | o | | | |
| growth factor receptor-bound protein 2 | 5 | o | | o | | | | | |
| growth hormone-inducible transmembrane protein | 5 | | | | o | | | | o |
| gtp cyclohydrolase 1 feedback regulatory protein | 5 | o | o | o | | o | | | o |
| guanine nucleotide binding 2 | 5 | o | | o | | o | o | | |
| guanine nucleotide binding protein (g protein) q polypeptide | 5 | o | | | | o | o | | |
| guanine nucleotide-binding protein g subunit alpha-2 | 5 | o | o | o | | | | | o |
| h aca ribonucleoprotein complex subunit 3 | 5 | o | o | o | | o | o | | |
| h+ lysosomal v1 subunit a | 5 | o | | | | | | o | |
| haptoglobin [Oreochromis mossambicus] | 5 | | | | o | | | | |
| hbs1-like protein | 5 | o | o | o | | o | | | |
| heat shock 70kda protein 4 | 5 | o | o | | | o | | | |
| heat shock protein 90 alpha | 5 | | | | o | | | | o |
| hect domain containing 1 | 5 | o | o | | | | o | o | |
| hepatitis b virus x-interacting protein | 5 | o | o | o | | o | | o | |
| heterogeneous nuclear ribonucleoprotein r | 5 | o | | | o | | o | | |
| hig1 domain family member 2a | 5 | o | o | o | | o | | o | |
| histidine ammonia-lyase | 5 | | o | o | | | | | |
| histone h2b 1 2-like | 5 | | o | | | | o | | o |
| hmg-box transcription factor 1 | 5 | o | o | | | | | | |
| homer homolog 1 | 5 | o | | | | | | | |
| huntingtin interacting protein k | 5 | o | o | | | o | o | o | |
| hydroxypyruvate isomerase | 5 | | o | | o | | | | |
| hypothetical loc729991 protein | 5 | o | o | | | o | o | | |
| ictacalcin | 5 | o | | o | | o | | | |
| immunoglobulin binding protein 1 | 5 | o | | o | o | | | | o |
| inner centromere protein | 5 | o | o | | o | o | | | |
| inositol hexaphosphate kinase 2 | 5 | o | o | | | o | | | o |
| inositol-3-phosphate synthase a | 5 | | | | | o | | | o |

| | | | | | | | | | |
|---|---|---|---|---|---|---|---|---|---|
| insulin induced gene 1 | 5 | o | o | o | | | | | |
| insulin-like growth factor binding protein 2 | 5 | | o | o | o | | | | |
| integrin alpha fg-gap repeat containing 1 | 5 | o | o | o | | o | | | |
| interferon inducible mx protein | 5 | o | | o | o | | | | |
| interferon regulatory factor 8 | 5 | o | | | | | | | o |
| interferon-induced protein 44-like | 5 | | | | | | | | o |
| interferon-inducible protein gig1-like | 5 | o | | o | | | | | |
| kelch-like ech-associated protein 1 | 5 | o | o | o | | | | o | o |
| keratin 8 | 5 | o | | o | o | o | | | o |
| kh domain rna signal transduction associated 1 | 5 | o | o | | | o | | o | o |
| lag1 ceramide synthase 2 | 5 | o | o | | | | o | | |
| legumain | 5 | o | o | | | o | | o | |
| leptin receptor gene-related protein | 5 | o | | o | | o | o | o | |
| leukocyte immune-type receptor | 5 | o | | o | | o | | | |
| limbic system-associated membrane protein | 5 | o | | | | o | o | o | |
| lipopolysaccharide-induced tumor necrosis factor-alpha factor homolog | 5 | o | | | o | | o | | o |
| lmbr1 domain containing 2 | 5 | o | o | o | | | o | | |
| loc495244 protein | 5 | o | | | | o | | | |
| loc563247 protein | 5 | o | o | o | | o | o | | |
| loc798124 protein | 5 | o | | | | o | | o | |
| low density lipoprotein receptor-related protein associated protein 1 | 5 | o | o | o | | o | | o | |
| low quality protein: titin-like | 5 | o | | | | | | | |
| lsm domain-containing protein 1 | 5 | o | o | o | | | o | o | |
| lyr motif-containing protein 4 | 5 | o | | o | o | o | | o | |
| lysophospholipase ii | 5 | o | o | o | | | o | | |
| lysosomal-associated protein transmembrane 4 alpha | 5 | o | o | | | o | o | | o |
| mago-nashi proliferation-associated | 5 | o | | o | | o | o | o | |
| malic enzyme nadp(+)- mitochondrial | 5 | o | o | | | | | | |
| mannose-binding lectin-associated serine protease-3a | 5 | | o | o | o | | | | |
| mannose-p-dolichol utilization defect 1 | 5 | o | o | o | | o | o | | |
| mediator complex subunit 22 | 5 | o | o | o | | | | | o |
| mediator of rna polymerase ii transcription subunit 9 | 5 | o | o | o | | o | o | | |
| membrane magnesium transporter 1-like | 5 | | | | o | | | | o |
| methylcrotonoyl-coenzyme a carboxylase 2 | 5 | o | o | o | | o | | | |
| methylmalonic aciduria (cobalamin deficiency) cbld with homocystinuria | 5 | o | o | | | o | | | |
| mhc class i alpha antigen | 5 | o | | o | o | | | | |
| microsomal glutathione s-transferase 1 | 5 | o | | o | o | o | o | | |
| microtubule-associated protein 1 light chain 3 beta | 5 | | o | o | | o | o | o | |
| mitochondrial 28s ribosomal protein s36 | 5 | | o | o | | | o | o | o |
| mitochondrial import receptor subunit tom20 homolog | 5 | | | | o | | | | o |
| mitochondrial import receptor subunit tom22 homolog | 5 | o | o | | o | o | | | |
| mitochondrial ribosomal protein l1 | 5 | o | | o | o | o | | o | |
| mitochondrial ribosomal protein l10 | 5 | o | o | o | o | | | | |
| mitochondrial ribosomal protein l12 | 5 | o | o | o | | o | | o | |
| mitochondrial ribosomal protein l40 | 5 | o | | o | o | o | | o | |
| mitochondrial ribosomal protein l53 | 5 | o | o | | | o | o | o | |
| mitochondrial ribosomal protein s15 | 5 | o | | o | | o | o | o | |
| mitochondrial ribosomal protein s17 | 5 | o | o | | | o | o | | |
| mitochondrial ribosomal protein s18b | 5 | o | | o | | o | o | | |
| mitogen-activated protein kinase 6 | 5 | o | o | | | | | o | |

| | | | | | | | | | |
|---|---|---|---|---|---|---|---|---|---|
| mitogen-activated protein kinase kinase 1-interacting protein 1 | 5 | o | o | o | | o | | o | |
| moesin | 5 | o | | o | | o | | o | |
| mps one binder kinase activator-like 1a | 5 | o | | | | | o | o | |
| mpv17 protein | 5 | o | o | o | o | | | | |
| mrna cap guanine-n7 methyltransferase | 5 | o | o | | | | | o | o |
| musashi homolog 1 | 5 | o | | | | o | o | o | |
| myelin-oligodendrocyte glycoprotein precursor | 5 | o | | | | | | | |
| myeloid lymphoid or mixed-lineage leukemia (trithorax drosophila) translocated 4 | 5 | o | | | | | o | o | |
| myocyte enhancer factor 2d | 5 | o | | | | | o | | |
| myosin light chain kinase member 4 | 5 | o | | | | | | | |
| myozenin 2 | 5 | o | | | | | | | |
| n-acylethanolamine-hydrolyzing acid amidase | 5 | o | o | | | | | o | o |
| nadh dehydrogenase 1 alpha subcomplex subunit 11 | 5 | o | o | o | | | | o | |
| nadh dehydrogenase 1 alpha subcomplex subunit 5 | 5 | | o | o | | o | o | o | |
| nadh dehydrogenase 1 alpha subcomplex subunit mitochondrial precursor | 5 | o | o | o | | o | o | | |
| nadh dehydrogenase 1 beta subcomplex subunit 10 | 5 | o | o | o | | o | | o | |
| nadh dehydrogenase 1 beta subcomplex subunit 4 | 5 | o | o | o | | o | | | |
| nadh dehydrogenase 1 beta subcomplex subunit 6 | 5 | o | o | o | | o | | o | |
| nadph--cytochrome p450 reductase | 5 | o | o | o | | | o | | |
| n-alpha-acetyltransferase auxiliary subunit | 5 | o | | | | o | | o | |
| nanor b-like | 5 | o | | | | | | o | |
| natural killer-tumor recognition sequence | 5 | o | | o | | | | o | o |
| nebulin-related anchoring protein isoform 2 | 5 | o | | | | | | | |
| neuropeptide y | 5 | | | | | | | o | o |
| nidogen 2 | 5 | o | | | | | | | |
| noelin precursor | 5 | | | | | | o | o | o |
| non-specific lipid-transfer protein | 5 | o | o | o | o | o | | | |
| nop58 protein | 5 | o | o | o | | o | | o | |
| novel krab box and zinc c2h2 type domain containing protein | 5 | o | | | | o | | | |
| novel protein (zgc:136474) | 5 | o | o | | | o | | o | |
| novel protein cytochrome family subfamily j | 5 | | o | | o | | | | o |
| novel protein vertebrate piccolo (presynaptic cytomatrix protein) | 5 | | | | | | o | o | |
| nuclear autoantigenic sperm protein (histone-binding) | 5 | o | | | o | | | | o |
| nuclear receptor coactivator 6 | 5 | o | o | | | | o | o | |
| nucleolar pre-ribosomal-associated protein 1 | 5 | | | | o | | | | |
| nucleolar protein 16 | 5 | o | o | o | | o | | o | |
| nucleolar protein 5a | 5 | o | o | o | | o | | | |
| nucleolin | 5 | o | o | | | | | | o |
| nucleoplasmin-like protein no29 | 5 | | | o | o | | | o | |
| nucleoside diphosphate kinase | 5 | | o | o | | o | | | o |
| nucleoside diphosphate kinase 7 | 5 | o | | | | o | | | |
| nudix (nucleoside diphosphate linked moiety x)-type motif 3 | 5 | o | | o | | | o | o | |
| orm1-like protein 1 | 5 | o | | o | o | o | | | o |
| ornithine decarboxylase antizyme | 5 | o | | o | o | | | | o |
| p2y purinoceptor 12 | 5 | o | | o | | o | | o | o |
| peroxiredoxin 1 variant 2 | 5 | | | o | | o | | o | o |
| peroxiredoxin 4 | 5 | o | o | o | | o | | o | |

| | | | | | | | | | |
|---|---|---|---|---|---|---|---|---|---|
| phosphoglycerate dehydrogenase | 5 | o | | o | | o | | | o |
| phosphoglycolate phosphatase | 5 | o | o | | o | o | | | |
| phospholipase a1 member a | 5 | | o | o | | | | o | |
| phospholipid transfer protein | 5 | o | o | o | | o | | | |
| plasma retinol-binding protein 1 | 5 | o | o | o | o | | | | |
| pleckstrin homology-like family member 1 | 5 | o | | | | o | | | o |
| plexin d1 | 5 | o | | o | | | | o | |
| poly(adp-ribose) glycohydrolase | 5 | o | | | | | | | o |
| polycomb group ring finger protein 1 | 5 | | | | o | | | | |
| potassium voltage-gated shaker-related member 3 | 5 | o | | | | | | | |
| prefoldin subunit 4 | 5 | o | | o | o | o | | o | |
| pre-mrna-processing factor 19 | 5 | o | o | | o | | | | |
| pre-rrna-processing protein tsr1 homolog | 5 | o | | | o | o | | | o |
| probable cation-transporting atpase 13a3-like | 5 | o | | o | | | o | | |
| probable e3 ubiquitin-protein ligase rnf144a-a | 5 | o | | o | o | | | | |
| probable palmitoyltransferase zdhhc6 | 5 | o | o | o | | | | o | |
| probable thiopurine s-methyltransferase | 5 | o | | o | | | | o | |
| procollagen- 2-oxoglutarate 5-dioxygenase 2 | 5 | o | | | | | o | | |
| programmed death ligand 1 | 5 | o | | | | | | | |
| proteasome ( macropain) 26s 1 | 5 | o | o | o | o | | | | o |
| proteasome ( macropain) 26s non- 12 | 5 | o | o | o | | o | | o | |
| proteasome ( macropain) 26s non- 3 | 5 | | o | o | | o | o | o | |
| proteasome ( macropain) activator subunit 4 | 5 | o | o | | | | | | |
| proteasome ( macropain) alpha 1 | 5 | o | | o | | o | | o | |
| proteasome activator complex subunit 2 | 5 | o | o | o | | o | | o | |
| proteasome assembly chaperone 2 | 5 | o | | | o | | | | o |
| proteasome beta type 10 | 5 | o | | o | | o | | o | o |
| proteasome beta type 8 | 5 | o | | o | | o | | o | o |
| proteasome subunit beta type-2 | 5 | o | | o | o | o | | o | |
| protein cwc15 homolog | 5 | o | o | o | | o | | o | |
| protein disulfide isomerase family member 3 | 5 | o | o | o | | o | | | |
| protein fam185a-like | 5 | o | | | | o | | | o |
| protein impact | 5 | o | o | o | | o | | | |
| protein- interferon-inducible double stranded rna dependent repressor of (p58 repressor) | 5 | o | o | | | | | | |
| protein kinase c binding protein 1 | 5 | o | | | | | | | |
| protein kish-a precursor | 5 | o | | o | | o | | o | |
| protein lyric | 5 | o | o | o | | | | o | |
| protein mab-21-like 1 | 5 | o | | | | o | | o | o |
| protein phosphatase 1 regulatory subunit 7 | 5 | o | o | o | | o | | o | |
| protein phosphatase methylesterase 1 | 5 | o | o | o | | | o | | |
| protein piccolo | 5 | | | | | | o | | |
| protein regulator of cytokinesis 1 | 5 | o | o | | o | | | | |
| protein yif1a | 5 | o | o | o | | | | | |
| protocadherin 1 gamma 22 | 5 | o | | | | | o | o | |
| protocadherin 2 alpha b 6 | 5 | o | | | | o | o | o | |
| protocadherin 9 | 5 | | | | | | o | o | |
| pterin-4-alpha-carbinolamine dehydratase | 5 | o | o | o | | o | | o | |
| pyrroline-5-carboxylate reductase member 2 | 5 | o | o | o | | | | | |
| pyruvate dehydrogenase alpha 1 | 5 | o | o | o | | | | | |
| pyruvate dehydrogenase component x | 5 | o | o | | | o | o | | |
| rap1 gtpase-gdp dissociation stimulator 1 isoform 1 | 5 | o | | | | | o | | |
| ras gtpase-activating-like protein iqgap1 | 5 | o | | o | | | | | |
| ras-related gtp binding a | 5 | o | o | o | o | | | o | |
| ras-related protein rab-11b | 5 | o | o | o | | | | | o |
| reactive oxygen species modulator 1 | 5 | o | o | o | | o | | | |
| regulation of nuclear pre-mrna domain containing 2 | 5 | o | | | | o | o | o | |

| | | | | | | | | | |
|---|---|---|---|---|---|---|---|---|---|
| reticulon 4 interacting protein 1 | 5 | o | o | | | o | | | |
| retrotransposon ty3-gypsy subclass | 5 | o | | | | | | o | |
| riboflavin kinase | 5 | | o | o | o | | | o | |
| ribonuclease h2 subunit b | 5 | o | | o | o | | | | |
| ribonuclease like 2 | 5 | | o | o | o | | | | o |
| ribonuclease uk114 | 5 | | o | o | | | | | o |
| ribosomal protein l12 | 5 | | o | | o | | o | | o |
| ribosomal protein l21 | 5 | o | o | o | | o | o | | |
| ribosomal protein l22-like 1 | 5 | o | o | | o | | | o | |
| ribosomal protein l24 | 5 | o | o | o | o | o | | | |
| ribosomal protein l26 | 5 | o | o | o | | o | | o | |
| ribosomal protein l28 | 5 | o | | o | | o | o | o | |
| ribosomal protein l3 | 5 | o | o | o | | o | | | |
| ribosomal protein l34 | 5 | o | o | o | | o | | o | |
| ribosomal protein l38 | 5 | o | o | | o | | | | o |
| ribosomal protein s13 | 5 | o | o | o | | o | | o | |
| ribosomal protein s15 | 5 | o | o | o | o | o | | | |
| ribosomal protein s18 | 5 | o | | o | o | o | | o | |
| ribosomal protein s20 | 5 | o | o | o | | o | | o | |
| ribosomal protein s27-like | 5 | o | o | o | | o | | o | |
| ribosomal protein s6 | 5 | o | o | o | | | | o | o |
| ribosome biogenesis protein brx1 homolog | 5 | o | o | o | | | | | o |
| ribosome biogenesis protein nsa2 homolog | 5 | o | o | o | | o | | o | |
| rilp-like protein 1 | 5 | o | o | | | | | | |
| ring finger protein 145 | 5 | o | | o | | | o | o | |
| ring finger protein 167 | 5 | o | | o | | o | | o | |
| ring finger protein 213 | 5 | o | | o | | | | | o |
| ring finger protein 213-like | 5 | o | | | | | o | | o |
| ring-box protein 2 | 5 | o | | o | o | o | | o | |
| rrna-processing protein fcf1 homolog | 5 | o | o | o | | o | o | | |
| rwd domain containing 4a | 5 | o | | o | | o | o | o | |
| ryanodine receptor skeletal muscle | 5 | o | | | o | | | | |
| sec23-interacting protein | 5 | | o | o | | o | | | o |
| selenoprotein m precursor | 5 | o | | o | | o | | | |
| sentrin-specific protease 8 | 5 | o | | o | o | | | | |
| serglycin precursor | 5 | o | o | o | | o | | o | |
| serine hydroxymethyltransferase 2 | 5 | o | o | o | | | | | |
| serine threonine kinase 19 | 5 | o | o | o | | o | | | |
| serine threonine kinase receptor associated protein | 5 | o | o | o | | o | | | |
| serine threonine-protein phosphatase 2a 56 kda regulatory subunit gamma isoform | 5 | o | | o | | | | o | |
| serologically defined colon cancer antigen 1 homolog | 5 | o | o | o | | | | o | |
| serum glucocorticoid regulated kinase 1 | 5 | o | | o | | | o | o | |
| serum paraoxonase arylesterase 2 | 5 | | o | | o | | | | |
| sh3 domain and tetratricopeptide repeats 2 | 5 | o | | o | | | | | |
| sideroflexin 4 | 5 | o | o | | o | | | o | |
| signal peptidase complex subunit 1 | 5 | o | o | o | | o | | o | |
| signal peptide peptidase 3 | 5 | o | | | | | o | o | |
| signal peptide peptidase-like 2b | 5 | o | o | | | | o | | |
| sil1 protein | 5 | o | | | | o | | | |
| sjchgc04881 protein | 5 | o | | | | | | | |
| slow myosin heavy chain 1 | 5 | o | | | | | | | |
| small g protein signaling modulator 3 | 5 | o | o | | | | o | | |
| small nuclear ribonucleoprotein polypeptide g | 5 | o | o | o | | o | | o | |
| small nuclear ribonucleoprotein sm d2 | 5 | o | o | o | o | | | o | |
| snapc5 protein | 5 | o | | o | | o | o | o | |
| sodium- and chloride-dependent taurine transporter | 5 | o | o | o | | | o | o | |

| | | | | | | | | | |
|---|---|---|---|---|---|---|---|---|---|
| sodium-coupled neutral amino acid transporter 2 | 5 | | | o | | | o | o | o |
| solute carrier family 25 member 3 | 5 | | o | | | | o | o | o |
| solute carrier family 35 (udp-glucuronic acid udp-n-acetylgalactosamine dual transporter) member d1 | 5 | o | o | o | | | | | |
| solute carrier family 37 (glucose-6-phosphate transporter) member 4 | 5 | o | o | o | | o | | | |
| solute carrier family member 2 | 5 | o | o | | o | | | | |
| solute carrier family member isoform cra_a | 5 | o | | | | | o | o | |
| solute carrier family sodium bicarbonate member 7 | 5 | o | | | | | o | o | |
| sorting nexin 12 | 5 | o | o | o | | o | | | o |
| sorting nexin 5 | 5 | o | o | o | | | o | | |
| sox3 | 5 | | | | o | o | o | o | |
| spermine oxidase | 5 | | | o | | | | o | o |
| splicing arginine serine-rich 5 | 5 | | o | | | | | o | o |
| sterile alpha motif domain-containing protein 9-like | 5 | o | | o | | | | | o |
| structural maintenance of chromosomes protein 1a | 5 | o | o | o | | | | o | |
| subfamily member 13 | 5 | o | o | o | | | | | |
| succinate dehydrogenase cytochrome b560 mitochondrial precursor | 5 | o | o | | | o | o | o | |
| surfeit 6 | 5 | o | o | o | | | | o | |
| swi snf matrix actin dependent regulator of subfamily member 2 | 5 | o | | | | | o | o | |
| sympk protein | 5 | o | | | | o | | | |
| synaptotagmin-like 1 | 5 | | | | | o | | o | o |
| syndecan-2-a precursor | 5 | o | o | o | | | o | | |
| taf9 rna polymerase tata box binding protein - associated factor | 5 | o | | o | | o | | o | |
| tartrate-resistant acid phosphatase type 5 precursor | 5 | | | | o | | | | |
| t-complex protein 1 subunit gamma | 5 | o | o | | | | | | o |
| tetratricopeptide repeat domain 4 | 5 | o | | o | o | o | | | |
| tetratricopeptide repeat protein 38 | 5 | o | o | o | | o | | | |
| thimet oligopeptidase | 5 | o | o | | | | | | |
| thioredoxin domain containing 5 | 5 | o | o | | | | o | | |
| tho complex subunit 7 homolog | 5 | o | | o | o | | | o | |
| threonyl-trna synthetase | 5 | o | o | o | | o | | | |
| thymosin beta | 5 | o | | o | o | | | o | |
| thyroid hormone receptor interactor 12 isoform 2 | 5 | o | | o | | | o | | |
| thyroid transcription factor 1-associated protein 26 homolog | 5 | o | | o | o | | | | |
| tm2 domain containing 1 | 5 | o | o | o | | o | | o | |
| tmem9 domain member b | 5 | o | o | o | | | o | o | |
| tnf receptor-associated protein 1 | 5 | o | o | | | o | | | |
| transaldolase | 5 | o | o | o | | o | | | o |
| transcription factor | 5 | o | | o | | | | | |
| transcription factor ap-1 | 5 | o | | | o | | | | o |
| transcription factor e2-alpha | 5 | o | | o | | | o | o | |
| transcriptional adapter 3 | 5 | | | | o | o | | | o |
| transcriptional repressor protein yy1-like | 5 | | | | o | | | | o |
| translation initiation factor eif-2b subunit epsilon | 5 | o | o | o | | o | | | |
| translocation protein sec62 | 5 | o | | o | | o | o | o | |
| transmembrane emp24 domain-containing protein 10 precursor | 5 | o | o | o | | | | o | o |

| | | | | | | | | | |
|---|---|---|---|---|---|---|---|---|---|
| transmembrane emp24 domain-containing protein 3 precursor | 5 | o | o | o | o | | | | |
| transmembrane emp24 protein transport domain containing 5 | 5 | o | o | o | | | | o | |
| transmembrane protein 120a | 5 | o | | o | | | | o | |
| transmembrane protein 128 | 5 | o | | o | | o | | | o |
| transmembrane protein 141 | 5 | o | o | | o | o | o | | |
| transmembrane protein 144 | 5 | | | | o | | | | |
| transmembrane protein 18 | 5 | o | o | o | | o | | o | |
| transmembrane protein 199 | 5 | o | | o | | o | | o | |
| transmembrane protein 49 | 5 | o | o | | | | o | | |
| transmembrane protein 59 | 5 | o | o | o | | o | | o | |
| transmembrane protein 59 precursor | 5 | | | | o | | o | o | o |
| transmembrane protein 63b | 5 | o | | | | | o | o | |
| transmembrane protein 87a | 5 | o | o | | | | | | |
| transmembrane protein c10orf57 homolog | 5 | o | o | o | | o | | o | |
| transportin 2 (importin karyopherin beta 2b) | 5 | o | | | | | o | | |
| transposon tx1 uncharacterized 149 kda | 5 | | | o | o | o | o | | |
| tributyltin binding protein type 2 | 5 | | | o | o | | | | |
| tripartite motif protein 21-like | 5 | o | | o | | | | | |
| tripeptidyl peptidase i | 5 | o | o | o | | | | | |
| tuberous sclerosis 2 | 5 | o | o | | | o | | | o |
| tubulin alpha-1c chain | 5 | o | | | o | | | | o |
| tubulin alpha-1c chain-like | 5 | | o | | | o | | | o |
| tubulin-specific chaperone c | 5 | o | | | o | | | | |
| tumor protein d52-like 2 | 5 | o | | | | o | o | | |
| tumor suppressor candidate 3 | 5 | o | | o | | o | | o | |
| tumor-associated calcium signal transducer 2 precursor | 5 | o | | | o | | | | |
| type alpha 3 | 5 | o | | o | | o | | | |
| type i keratin s8 | 5 | o | o | o | o | | o | | |
| type ii | 5 | o | o | | | o | | | |
| tyrosyl-trna synthetase | 5 | o | o | o | | o | | | o |
| u1 small nuclear ribonucleoprotein 70 kda | 5 | o | o | | | o | | | o |
| u3 small nucleolar ribonucleoprotein protein imp3 | 5 | o | | o | o | o | | o | |
| u4 tri-snrnp-associated protein 1 | 5 | | | | o | | | | |
| u6 snrna-associated sm-like protein lsm1 | 5 | o | o | | o | o | | | |
| ubiquitin carboxyl-terminal hydrolase 10 | 5 | o | o | | | o | o | | |
| ubiquitin carboxyl-terminal hydrolase 5 | 5 | o | | | o | o | o | | |
| ubiquitin protein ligase e3 component n-recognin 3 | 5 | o | | | | | | o | |
| ubiquitin specific peptidase like 1 | 5 | o | o | | | o | o | | |
| ubiquitin-conjugating enzyme e2l 3 | 5 | o | | o | | o | | o | |
| ubiquitin-conjugating enzyme e2q family member 2 | 5 | o | o | | | | o | | |
| ubiquitin-like 3 | 5 | o | o | | | | o | o | |
| ubiquitin-like protein 5 | 5 | o | o | | | | o | o | |
| ubtf protein | 5 | o | | o | | o | o | o | |
| udp- c:betagal beta- -n-acetylglucosaminyltransferase 5 | 5 | o | | | o | | | | |
| udp-glucose pyrophosphorylase 2 | 5 | o | o | o | | | | | |
| upf0466 protein mitochondrial-like | 5 | o | o | o | | o | o | | |
| upf0600 protein c5orf51-like | 5 | o | o | o | | o | | | |
| upstream binding transcription rna polymerase i | 5 | o | | | | | o | o | |
| uracil phosphoribosyltransferase | 5 | o | | o | | | o | o | |
| uracil-dna glycosylase | 5 | o | | o | | o | | o | |
| uv excision repair protein rad23 homolog b | 5 | o | o | | | o | o | o | |

| | | | | | | | | | |
|---|---|---|---|---|---|---|---|---|---|
| vacuolar protein sorting 13 homolog a ( cerevisiae) | 5 | o | o | o | | | | | |
| vesicle-fusing atpase | 5 | o | | | | | o | | o |
| vesicle-trafficking protein sec22b-b | 5 | o | o | o | | | | | |
| voltage-dependent anion-selective channel protein 1 | 5 | | | | | | | | o |
| voltage-gated sodium channel | 5 | o | | | | | | | |
| von willebrand factor d and egf domain-containing | 5 | o | | | | | | | |
| vps10 domain receptor protein sorcs 3-like | 5 | o | | | | o | | | |
| v-type proton atpase 116 kda subunit a isoform 2 | 5 | o | | | | o | o | | |
| v-type proton atpase subunit d | 5 | o | o | | | o | | | o |
| v-type proton atpase subunit e 1 | 5 | o | o | o | | | o | o | |
| wd repeat- and fyve domain-containing protein 4 | 5 | | o | | o | | o | | |
| wd repeat phosphoinositide interacting 1 | 5 | o | o | o | | o | | | |
| y box binding protein 1 | 5 | | | o | o | | | | o |
| yeats domain containing 4 | 5 | o | | o | o | o | o | | |
| yip1 domain member 4 | 5 | o | o | o | | o | o | | |
| yippee-like 5 | 5 | o | o | | | o | o | o | |
| zinc cchc domain containing 17 | 5 | o | o | | | o | o | o | |
| zinc finger cchc domain-containing protein 4 | 5 | | | | o | | | | |
| zinc finger protein 292 | 5 | o | | | o | | | | o |
| zinc finger protein 572 | 5 | o | | o | | | o | | |
| zinc finger protein 576 | 5 | o | | | o | | | | o |
| zinc finger protein 622 | 5 | o | o | | | o | | | o |
| zinc finger protein 646 | 5 | o | | | | | o | o | |
| zinc finger protein 821 | 5 | o | | | | | | | |
| 10 kda heat shock mitochondrial | 4 | o | o | | | o | | o | |
| 11-beta-hydroxysteroid dehydrogenase type 3 | 4 | o | o | o | | | | | |
| 1-acylglycerol-3-phosphate o-acyltransferase 3 | 4 | o | o | | | | o | o | |
| 24-dehydrocholesterol reductase | 4 | o | o | o | | o | | | |
| 26s proteasome non-atpase regulatory subunit 3 | 4 | o | o | | | | | | o |
| 2-hydroxyacyl- lyase 1 | 4 | o | o | o | | | | | |
| 39s ribosomal protein mitochondrial | 4 | o | | | o | | | o | |
| 3-hydroxy-3-methylglutaryl-coenzyme a reductase | 4 | | o | | o | | | | |
| 40s ribosomal protein s20 | 4 | | | | o | | | | |
| 5 -amp-activated protein kinase catalytic subunit alpha-1 | 4 | o | | o | | | | o | |
| 5 -nucleotidase domain containing 2 | 4 | o | | | | o | | o | |
| 5a11 basigin-2 | 4 | o | o | o | | | | | |
| 60s ribosomal protein l22 | 4 | o | o | | | | o | | o |
| 6-phosphofructo-2-kinase fructose- -biphosphatase 4 | 4 | o | | | | | | o | o |
| a chain crystal spectroscopic and catalytic properties of cobalt copper nickel and mercury derivatives of the zinc endopeptidase a correlation of structure and proteolytic activity | 4 | | | | o | | | | |
| acetyl- carboxylase 1 | 4 | o | o | | | | | | |
| acetyl- carboxylase 2 | 4 | o | o | | | | | | o |
| aconitate mitochondrial | 4 | o | | o | | | o | | |
| actin beta subunit | 4 | | | | | | | | o |
| actin binding 2a | 4 | | | o | | | | | |
| actin related protein 2 3 subunit 41kda | 4 | o | o | o | | | | | |

| | | | | | | | | | |
|---|---|---|---|---|---|---|---|---|---|
| actin-related protein 2 3 complex subunit 1b | 4 | o | | o | o | o | | | |
| activating signal cointegrator 1 complex subunit 3 | 4 | o | o | o | | | | | |
| activating transcription factor 6 | 4 | o | | o | | | o | | |
| activating transcription factor 7-interacting protein 1 | 4 | o | | | o | o | | | |
| acyl- dehydrogenase family member mitochondrial | 4 | | o | | o | o | | | |
| acyl- synthetase bubblegum family member 2 | 4 | o | o | | | | | | |
| acyl- synthetase short-chain family member 2 | 4 | o | o | | | | | | |
| acyl- thioesterase 9 | 4 | o | | | | | | | o |
| acyl-coenzyme a very long chain | 4 | o | o | o | | | | | |
| acyl-protein thioesterase 1 | 4 | o | o | o | o | | | | |
| adam metallopeptidase with thrombospondin type 1 13 | 4 | | o | o | | | | | |
| adaptor-related protein complex beta 1 subunit | 4 | o | o | | | | o | | |
| adaptor-related protein complex gamma 1 subunit isoform 2 | 4 | o | | o | | | o | | |
| adenylate cyclase activating polypeptide 1 receptor type i | 4 | | | | | | o | o | |
| adenylyl cyclase-associated protein 1 | 4 | o | o | o | | | | o | |
| adp-ribosylation factor 3 | 4 | o | | | | | | o | |
| adp-ribosylation factor-like 2 | 4 | o | | o | | o | | o | |
| adp-ribosylation factor-like protein 4a | 4 | o | | o | | o | | o | |
| af442732_3reverse transcriptase ribonuclease h methyltransferase | 4 | | | o | | o | | o | |
| alanyl-trna synthetase | 4 | o | | o | | | | | o |
| aldehyde dehydrogenase 18 member a1 | 4 | o | o | o | | o | | | |
| aldehyde dehydrogenase 7 member a1 | 4 | o | o | o | o | | | | |
| aldehyde dehydrogenase 9 member a1 | 4 | | o | | | | | | |
| alkylated dna repair protein alkb homolog 1 | 4 | o | o | | | o | | | |
| alpha 2-macroglobulin 4 | 4 | | o | o | | | | | |
| alpha- and gamma-adaptin-binding protein p34 | 4 | o | o | o | | | | | |
| alpha-mannosyl-glycoprotein 2-beta-n-acetylglucosaminyltransferase | 4 | | o | o | | | | | o |
| alpha-2-antiplasmin precursor | 4 | | o | o | o | | | | |
| amino-terminal enhancer of split | 4 | o | o | | | | | o | |
| amphiphysin | 4 | o | | | | o | | | o |
| amplified in osteosarcoma | 4 | o | o | o | | | | | |
| amylo- 6- 4-alpha-glucanotransferase isoform 1 | 4 | o | | | | | | | |
| angiopoietin-like 2 | 4 | o | | | | | | | |
| angiopoietin-related protein 4 | 4 | | o | o | | | o | | |
| angiotensin converting enzyme | 4 | o | | | | | | o | o |
| ankyrin repeat and ibr domain-containing protein 1 | 4 | o | | | | o | | o | |
| ankyrin repeat domain-containing protein 46 | 4 | o | o | | | o | | | o |
| ankyrin unc44 | 4 | o | | | | | | | |
| annexin a4 | 4 | o | o | o | | o | | | |
| anthrax toxin receptor 1 | 4 | o | o | | | | | | |
| ap-1 complex subunit mu-2 | 4 | | o | | o | | | | |
| ap-1 complex subunit sigma-2 | 4 | o | | o | | | | o | o |
| ap-3 complex subunit delta-1 | 4 | | | o | | o | o | o | |
| ap-4 complex subunit sigma-1-like | 4 | | o | | | | | | o |
| apobec1 complementation factor | 4 | | o | | | | | | |
| apolipoprotein a-iv | 4 | | o | o | | | | | |
| apolipoprotein b-100-like | 4 | | | | o | | | | |
| apolipoprotein d | 4 | o | o | o | o | | | | |

| | | | | | | | | | |
|---|---|---|---|---|---|---|---|---|---|
| apolipoprotein o-like | 4 | o | o | o | | | | | |
| aquaporin-4 | 4 | o | | | | | | | |
| arf-gap with ank repeat and ph domain-containing protein 1-like | 4 | | | | | | | | o |
| arp1 actin-related protein 1 homolog centractin beta | 4 | o | o | | | | | o | |
| aryl hydrocarbon receptor interacting protein | 4 | o | o | | | o | | o | |
| arylsulfatase a | 4 | o | | o | | | | | o |
| aspartate mitochondrial precursor | 4 | o | | o | | o | | | o |
| aspartoacylase | 4 | o | o | o | | | | | o |
| atp citrate lyase | 4 | o | o | | | | | | o |
| atp h+ mitochondrial f1 delta subunit | 4 | o | o | | | o | | | o |
| atp synthase subunit 6 | 4 | | | | o | | | | o |
| atp1a3 protein | 4 | | | | | | | | o |
| atp-binding cassette sub-family f member 1 | 4 | o | o | o | | o | | | |
| atp-binding sub-family b (mdr tap) member 8 | 4 | o | o | | | | | | |
| atp-binding sub-family c (cftr mrp) member 2 | 4 | o | o | o | | | | | |
| atp-dependent rna helicase ddx54 | 4 | o | o | | o | | | | o |
| atp-dependent rna helicase ddx55 | 4 | o | | | | | | | o |
| atp-sensitive inward rectifier potassium channel 10 | 4 | | | | | | o | | o |
| b-cell cll lymphoma 9-like | 4 | o | | | | | | | |
| bcl2-associated x protein | 4 | o | o | | o | | | | o |
| beta (43kda dystrophin-associated glycoprotein) | 4 | | | | | | | o | o |
| beta tubulin 3 | 4 | | | | | | | | o |
| beta-lactamase-like protein 2 | 4 | o | o | o | | | | | o |
| bile acid receptor | 4 | | o | o | | | | | |
| biogenesis of lysosome-related organelles complex 1 subunit 1 | 4 | o | | o | | o | | o | |
| bola-like protein 1 | 4 | o | o | | | o | o | | |
| bone morphogenetic protein 1 | 4 | o | o | o | | | o | | |
| brain-specific angiogenesis inhibitor 1 | 4 | o | | | | | o | | |
| branched chain keto acid dehydrogenase beta polypeptide | 4 | o | o | | | o | | | |
| brefeldin a-inhibited guanine nucleotide-exchange protein 1 | 4 | | o | o | o | | | o | |
| bromodomain containing 4 | 4 | o | | | | | o | | |
| btb domain containing 6 | 4 | | | | | o | | | o |
| btbd2 protein | 4 | o | o | o | | o | | | |
| c12orf51 protein | 4 | o | | | | | o | | |
| c16orf14 homolog | 4 | o | | o | | o | | | |
| c18orf32 homolog | 4 | o | o | o | | | | o | |
| c19orf12 homolog | 4 | o | o | | | | | | |
| c1galt1-specific chaperone 1 | 4 | o | | o | | | | | o |
| c1orf50 homolog | 4 | o | o | | | | o | o | |
| c21orf51 homolog | 4 | o | | | | o | | o | |
| c7orf24 homolog | 4 | o | o | | | o | o | | |
| calcineurin binding protein 1 | 4 | o | | | | o | o | o | |
| calcium binding protein p22 | 4 | o | | o | | o | | | |
| calcium calmodulin-dependent protein kinase ( kinase) ii gamma 1 | 4 | o | | | | | o | o | |
| calcium calmodulin-dependent protein kinase ii inhibitor 2 | 4 | | | | | o | o | o | o |
| calcium voltage- gamma subunit 1 | 4 | o | | | | | | | |
| calcium-binding protein 39 | 4 | o | | | | o | | | |
| calmodulin regulated spectrin-associated protein 1-like 1 | 4 | o | | | | | o | | |
| calpain (m ii) large subunit a | 4 | o | | | | o | o | | |
| calsyntenin 1 | 4 | o | | | | | o | o | |

| | | | | | | | | | |
|---|---|---|---|---|---|---|---|---|---|
| camp-dependent protein kinase inhibitor gamma | 4 | o | o | | | | | | o |
| capping protein (actin filament) muscle z- beta | 4 | | o | o | | o | | | o |
| carbohydrate kinase domain-containing | 4 | | | | o | o | | | o |
| carboxypeptidase a2 | 4 | o | o | | | | | | |
| carboxypeptidase b2 | 4 | | o | o | | | | | |
| carboxypeptidase e | 4 | o | | | | | | | o |
| carboxypeptidase polypeptide 1 | 4 | o | o | o | | | | o | |
| cardiomyopathy associated 5 | 4 | o | | | | | | | |
| cartilage associated protein | 4 | | | o | o | | | o | o |
| casein kinase gamma 2 | 4 | o | o | o | | | | | o |
| casein kinase ii subunit beta | 4 | | | | o | | | | o |
| caspace-like protein | 4 | o | | | | | | | |
| castor homolog zinc finger | 4 | o | | | | | | | |
| catechol o-methyltransferase | 4 | o | o | o | | o | | | |
| catenin beta-1 | 4 | o | o | | | | | | o |
| cathepsin b precursor | 4 | | o | o | o | | | | o |
| ccaat enhancer-binding protein delta | 4 | o | o | o | | | | | |
| cd029 protein | 4 | o | | | | | | | |
| cd209 antigen-like protein d | 4 | | o | o | | | | | o |
| cd48 molecule | 4 | o | | | | | | | |
| cdk5 regulatory subunit associated protein 3 | 4 | o | o | o | | | | | |
| cdkn2a interacting protein n-terminal like | 4 | o | o | o | | | | o | |
| cell death activator cide-b | 4 | | o | o | | | | | o |
| cell differentiation protein rcd1 homolog | 4 | o | | o | o | | | o | |
| cell division cycle 123 homolog ( cerevisiae) | 4 | o | o | o | | | | | |
| centrin 3 | 4 | o | o | | | o | o | | |
| charged multivesicular body protein 3 | 4 | o | | | | o | | o | |
| charged multivesicular body protein 6 | 4 | o | | o | | | | o | |
| cholinergic alpha 7-like | 4 | o | | | | | o | | |
| chondroitin sulfate proteoglycan 2 | 4 | o | | | | | o | | |
| chondroitin sulfate proteoglycan 5-like | 4 | | | | | | | | o |
| chromobox protein homolog 1 | 4 | | | | | o | o | o | |
| chromobox protein homolog 5 | 4 | o | o | o | | | | o | |
| chromosome 1 open reading frame 123 | 4 | o | | o | | | o | o | |
| chromosome 1 open reading frame 91 | 4 | o | | o | | o | | | |
| chromosome 14 open reading frame 153 | 4 | o | | o | | o | | o | |
| chromosome 2 open reading frame 43 | 4 | o | | o | | o | | o | |
| chromosome 6 open reading frame 106 | 4 | o | o | o | | | | | |
| chromosome 6 open reading frame 125 | 4 | o | o | | o | o | | | |
| chromosome 6 open reading frame 62 | 4 | o | o | | | o | | o | |
| chromosome 9 open reading frame 16 | 4 | o | | o | | o | | o | |
| chromosome 9 open reading frame 25 | 4 | o | | | | | o | | |
| chromosome x open reading frame 26 | 4 | o | | o | | o | | o | |
| chronic lymphocytic leukemia deletion region gene 6 protein homolog | 4 | o | | o | | o | | | |
| c-jun-amino-terminal kinase-interacting protein 1-like | 4 | | | | | | o | o | |
| clathrin light polypeptide | 4 | o | o | o | | o | | | |
| claudin 6 | 4 | o | | | o | o | | o | |
| coagulation factor ii | 4 | | o | o | | | | | |
| coatomer subunit gamma-2 | 4 | | o | | | | | | o |
| cobl-like 1 | 4 | o | o | | | | o | | |
| coenzyme q6 monooxygenase ( cerevisiae) | 4 | o | o | | | | o | | |
| cofactor required for sp1 transcriptional subunit 3 | 4 | | | | | | o | o | |
| coiled-coil domain containing 109b | 4 | o | | | | | | | |
| coiled-coil domain containing 25 | 4 | o | | o | | o | o | | |
| coiled-coil domain containing 86 | 4 | o | o | o | | o | | | |

| | | | | | | | | | |
|---|---|---|---|---|---|---|---|---|---|
| coiled-coil domain-containing protein 43 | 4 | o | o | o | o | | | | |
| coiled-coil-helix-coiled-coil-helix domain containing 3 | 4 | o | | o | | | | | o |
| coiled-coil-helix-coiled-coil-helix domain-containing protein 6 | 4 | o | o | | | o | | | |
| collagen alpha-1 chain-like | 4 | o | | | o | | o | | o |
| complement component 6 | 4 | o | o | o | | | | | |
| complement component c8 beta chain | 4 | | | | o | | | | o |
| connexin 43 | 4 | o | | | | o | o | o | |
| cop9 signalosome complex subunit 3 | 4 | o | | | o | | | | |
| copia protein | 4 | o | | | | o | | | |
| copine viii | 4 | | | | | | o | o | |
| cornifelin homolog b | 4 | o | o | o | | | | o | |
| cox16 cytochrome c oxidase assembly homolog | 4 | o | o | o | | o | | | |
| ctd (carboxy-terminal rna polymerase polypeptide a) small phosphatase like 2 | 4 | o | | | | | o | | |
| cub and sushi multiple domains 1 | 4 | | | | | | o | | |
| cullin 2 | 4 | o | o | o | | | | | |
| cyclin i | 4 | o | o | | | | o | | |
| cyclin-dependent kinase 2-associated protein 1 | 4 | o | | o | | o | | o | |
| cyclin-dependent kinase 2-associated protein 2 | 4 | o | | o | | o | o | | |
| cyclin-dependent kinase 4 | 4 | o | | | | | | | o |
| cyclin-dependent kinase 5 | 4 | | | o | | | o | o | |
| cyclin-dependent kinase 6 | 4 | o | | o | | | | | |
| cyclin-dependent kinase 8 | 4 | o | o | | | | | | |
| cyclin-dependent kinase 9 | 4 | | o | | o | | | | |
| cysteine- angiogenic 61 | 4 | o | | | | | | | |
| cysteine dioxygenase type 1 | 4 | o | o | o | | | | | |
| cysteine protease atg4b | 4 | o | | o | | | | | o |
| cysteinyl-trna synthetase mitochondrial | 4 | o | | | | | | | |
| cystinosin | 4 | o | o | o | | | | | |
| cytochrome c oxidase subunit viib | 4 | | o | o | | | | o | o |
| cytochrome family subfamily polypeptide 2 | 4 | | o | o | | | o | | |
| cytosolic fe-s cluster assembly factor nubp2 | 4 | o | | o | | o | o | | |
| cytosolic non-specific dipeptidase | 4 | | | o | | o | | o | o |
| d-dopachrome tautomerase | 4 | o | o | o | | | | o | |
| dead (asp-glu-ala-asp) box polypeptide 56 | 4 | o | | o | o | | | o | |
| deah (asp-glu-ala-his) box polypeptide 38 | 4 | o | o | | | | | | |
| death inducer-obliterator 1 | 4 | o | | | | | o | | |
| death-associated protein kinase 2 | 4 | o | o | o | | | | | |
| decorin | 4 | o | | o | | o | | o | |
| dedicator of cytokinesis 11 | 4 | o | | | | | o | | |
| dedicator of cytokinesis 4 | 4 | o | | | | | o | | |
| dehydrodolichyl diphosphate synthase | 4 | o | | o | o | o | | | |
| dehydrogenase e1 and transketolase domain containing 1 | 4 | o | o | | | | | | |
| dehydrogenase reductase sdr family member 4 | 4 | o | o | o | o | | | | |
| dendritic cell immunoactivating receptor | 4 | | | | | o | | | o |
| deoxyhypusine synthase | 4 | o | | | | | | o | o |
| deoxyribose-phosphate aldolase | 4 | | o | | o | | | | |
| diacylglycerol alpha | 4 | | | | | | o | o | |
| dihydrolipoamide dehydrogenase | 4 | o | o | | | | | | |
| di-n-acetylchitobiase | 4 | | o | o | | | | o | |
| dna damage-binding protein 1 | 4 | o | o | | | | | | o |
| dna excision repair protein ercc-6-like | 4 | | o | | o | | | | |
| dna replication complex gins protein psf2 | 4 | o | | | o | o | | | |
| dna-binding protein inhibitor id-3 | 4 | o | o | o | o | | | | |

| | | | | | | | | | |
|---|---|---|---|---|---|---|---|---|---|
| dna-directed rna polymerase ii subunit rpb7 | 4 | o | o | | | o | | o | |
| dnaj homolog subfamily c member 15 | 4 | o | | | o | o | | o | |
| dolichol-phosphate mannosyltransferase subunit 3 | 4 | o | o | o | | o | | | |
| dolichyl-phosphate mannosyltransferase polypeptide catalytic subunit | 4 | o | | o | | o | | | o |
| dst protein | 4 | | | | | | | | o |
| dual specificity mitogen-activated protein kinase kinase 4 | 4 | o | o | | | o | | | |
| dynactin subunit 4 | 4 | o | | | | | o | o | |
| dynactin subunit 6 | 4 | o | | o | | o | o | | |
| dynamin 1-like | 4 | o | | | | o | o | o | |
| dystroglycan preproprotein | 4 | o | | | | | o | o | |
| e2f transcription factor 4 | 4 | | o | o | o | | | | |
| e2f-associated phosphoprotein | 4 | o | | | | o | o | o | |
| e3 sumo-protein ligase 2 | 4 | o | | | | | o | | |
| e3 sumo-protein ligase nse2 | 4 | o | | o | | o | | o | |
| e3 ubiquitin-protein ligase bre1a | 4 | | o | | | | o | | o |
| e3 ubiquitin-protein ligase siah2 | 4 | | | | o | | | | o |
| ecto-adp-ribosyltransferase 5-like | 4 | o | | o | | | | | |
| ectonucleoside triphosphate diphosphohydrolase 1 | 4 | o | o | o | | | | | o |
| egf-like repeat and discoidin i-like domain-containing protein 3-like | 4 | | | | | o | | o | o |
| eh domain-containing protein 3 | 4 | o | | o | o | | | | |
| eif4e binding protein | 4 | o | o | o | o | | | | |
| elastase 2a | 4 | o | o | o | | | | | |
| elmo ced-12 domain containing 2 | 4 | o | o | | | o | | o | |
| elongation factor 1-alpha 1 | 4 | | | | o | | o | | |
| endothelial pas domain protein 1 | 4 | o | | | | | | o | |
| endothelin receptor type a | 4 | o | | | o | | | | |
| engulfment and cell motility protein 1 | 4 | o | | | | | o | | o |
| ependymin-2 precursor | 4 | | | | o | | | | o |
| epidermal growth factor receptor | 4 | o | | o | | | | | |
| epithelial membrane protein 3 | 4 | o | | o | | o | | | |
| epoxide hydrolase 1 | 4 | o | o | | | o | | | |
| epoxide hydrolase microsomal | 4 | | o | | | | | o | o |
| erbb2 interacting protein | 4 | o | | | | | | o | |
| ester hydrolase c11orf54 homolog | 4 | o | o | | | o | | o | |
| eukaryotic elongation factor-2 kinase | 4 | o | | | | | | | |
| eukaryotic initiation factor 4a-i | 4 | | | | o | | | | o |
| eukaryotic translation initiation factor 3 subunit j | 4 | | o | o | | o | o | | |
| eukaryotic translation initiation factor 3 subunit k | 4 | o | o | o | | | | o | |
| eukaryotic translation initiation factor 3 subunit l | 4 | o | o | o | o | | | | |
| eukaryotic translation initiation factor 3 subunit l-like | 4 | | | | o | | | | |
| eukaryotic translation initiation factor 6 | 4 | | o | | o | | | | o |
| eukaryotic translation initiation factor isoform 1 | 4 | o | o | o | | | o | | |
| eukaryotic translation initiation factor isoform 1b | 4 | | | o | | o | | o | |
| eukaryotic translation termination factor 1 | 4 | | o | o | | o | | | |
| exocyst complex component 2 | 4 | o | | o | | o | | o | |
| exocyst complex component 7 | 4 | o | | | | o | | | |
| exosome component 10 | 4 | o | | | | o | | | o |
| exostosin 1 | 4 | o | | | o | o | | | |
| extended synaptotagmin-1-like | 4 | o | | | | | | | o |

| | | | | | | | | | |
|---|---|---|---|---|---|---|---|---|---|
| fas apoptotic inhibitory molecule 2 | 4 | | | | | | o | o | o |
| f-box only protein 6 | 4 | o | o | | | | o | | o |
| fibrillin 1 | 4 | o | o | | | | | | |
| fibrinogen gamma polypeptide | 4 | | o | | o | | | | |
| fibrinogen-like 1 | 4 | o | | | | | | | |
| fibroblast growth factor-binding protein 2-like | 4 | | | | | | | | o |
| fibronectin type-iii domain-containing protein unq728 pro1410 homolog precursor | 4 | o | | | | o | | o | o |
| filamin gamma isoform 1 | 4 | o | | | | | | | |
| fk506 binding protein 10 | 4 | o | o | | | | | | |
| folylpolyglutamate synthase | 4 | o | o | | | | | | |
| formin binding protein 4 | 4 | | o | | | | o | o | |
| fshd region gene 1 | 4 | o | o | o | | o | | | |
| fumarate hydratase | 4 | o | o | o | | | | | |
| fxyd domain containing ion transport regulator 8 | 4 | o | | | | | | | |
| fyn-binding protein | 4 | | | | o | | | | o |
| g kinase anchoring protein 1 | 4 | o | | o | o | | | | |
| g protein beta subunit-like | 4 | o | | | | o | | | |
| g2 m-phase specific e3 ubiquitin ligase-like | 4 | o | | o | | | | | |
| gag-pol protein | 4 | o | | | | o | | | |
| gag-pro-pol polyprotein | 4 | | o | o | | | | | |
| galactose-1-phosphate uridylyltransferase | 4 | o | o | | | | | | o |
| galectin-related protein | 4 | o | | o | | | | | o |
| gastric cancer antigen zg14 homolog | 4 | o | | o | | o | | o | |
| gastrula zinc finger protein | 4 | o | | | | | | o | |
| gcn1 general control of amino-acid synthesis 1-like 1 | 4 | o | o | o | | | o | | |
| gdp dissociation inhibitor 2 | 4 | o | | o | | o | | o | |
| general transcription factor ii-i repeat domain-containing protein partial | 4 | o | | | | o | | | |
| GK25437 [Drosophila willistoni] | 4 | o | | | | | | | |
| glucocorticoid receptor dna-binding factor 1 | 4 | o | o | | | o | | | |
| glutamate metabotropic 2 | 4 | | | | | o | | | o |
| glutamate-cysteine catalytic subunit | 4 | o | o | | | | o | | |
| glutaredoxin-1 | 4 | o | o | | | o | o | | |
| glutathione reductase | 4 | o | o | | | | | | |
| glycoprotein beta polypeptide | 4 | o | | | | | | | |
| golgi gamma adaptin ear arf binding protein 1 | 4 | o | | | | o | | | |
| golgi golgin subfamily 7 | 4 | o | | | | | o | o | |
| golgi to er traffic protein 4 homolog | 4 | o | | o | o | | | | |
| gpn-loop gtpase 3 | 4 | | o | | | o | | | o |
| granulocyte colony-stimulating factor receptor-like | 4 | | | | o | | | | |
| g-rich sequence factor 1 | 4 | | | o | o | o | | | o |
| grip and coiled-coil domain-containing protein 1 | 4 | | | | | | | | o |
| growth and transformation-dependent protein | 4 | o | | o | | o | | o | |
| growth arrest and dna damage-inducible protein gadd45 gamma | 4 | o | o | o | | | | | |
| growth factor receptor-bound protein 14 | 4 | o | | | | | | | |
| growth inhibition and differentiation-related protein 88 homolog | 4 | | o | | o | | | | o |
| guanine nucleotide binding 3 | 4 | o | | o | | o | | o | |
| guanine nucleotide binding protein (g protein) alpha 13 | 4 | o | o | o | | | | o | |
| guanine nucleotide binding protein (g protein) beta polypeptide 1 | 4 | o | | | | | o | o | |
| guanine nucleotide binding protein (g protein) gamma 13 | 4 | o | | | | o | o | o | |

| | | | | | | | | | |
|---|---|---|---|---|---|---|---|---|---|
| h+ lysosomal accessory protein 2 | 4 | o | o | | | | | o | o |
| h+ lysosomal v1 subunit f | 4 | o | o | | o | | | | o |
| haloacid dehalogenase-like hydrolase domain containing 3 | 4 | o | | o | | | | | |
| hat family dimerisation | 4 | o | | | | | | | |
| heat shock 70kda protein 14 | 4 | o | | o | | | | | |
| heat-responsive protein 12 | 4 | o | o | | | o | | o | |
| heavy chain cardiac beta | 4 | o | | | | | | | |
| heavy polypeptide 200kda-like isoform 1 | 4 | o | | | | | | | |
| heavy polypeptide a | 4 | | o | | | | o | | |
| hect (homologous to the e6-ap carboxyl terminus) domain and rcc1 -like domain 1 | 4 | o | o | | | | | | |
| hematological and neurological expressed 1 | 4 | | | | | o | o | | o |
| heme transporter hrg1 | 4 | o | o | | | | o | | |
| hepatocyte nuclear factor 4-alpha | 4 | | o | o | | | | | |
| hig1 domain family member 1a | 4 | o | o | o | | | | o | |
| high affinity choline transporter 1-like | 4 | | o | | o | | | | o |
| high affinity immunoglobulin gamma fc receptor i precursor | 4 | o | | | | | | | |
| hira interacting protein 5 | 4 | o | | o | | o | | o | |
| hira-interacting protein 3 | 4 | | o | | o | | | | |
| histone h5a | 4 | o | o | o | | | | | |
| hiv-1 tat interactive protein 30kda | 4 | o | o | o | | o | | | |
| hobo-like transposase | 4 | o | | | | o | o | | |
| homeodomain transcription factor 2 | 4 | o | | o | | | o | | |
| homolog subfamily c member 12 | 4 | o | | o | | o | | o | |
| homolog subfamily c member 8 | 4 | o | | o | o | o | | | |
| hormone-sensitive lipase | 4 | o | o | | o | | | | |
| hsp90 co-chaperone cdc37 | 4 | o | | o | | o | | o | |
| human immunodeficiency virus type i enhancer binding protein 2 | 4 | o | | | | | o | | |
| human immunodeficiency virus type i enhancer binding protein 3 | 4 | o | | | | | o | o | |
| hyaluronan binding protein 2 | 4 | | o | | o | | | | |
| hydroxyacyl-coenzyme a dehydrogenase 3-ketoacyl-coenzyme a thiolase enoyl-coenzyme a hydratase (trifunctional protein) alpha subunit | 4 | o | o | o | | o | | | |
| im:6912380 protein | 4 | | | | | | o | o | |
| importin subunit alpha-4 | 4 | o | o | | | | | | |
| inhibitor of growth member 1 | 4 | o | | o | o | o | | | |
| inositol polyphosphate-5- 40kda | 4 | o | o | | | | | o | |
| insulin-like growth factor binding acid labile subunit | 4 | | o | | o | | | | |
| integrator complex subunit 7 | 4 | o | | | o | | o | | |
| interferon regulatory factor 1 | 4 | o | o | o | | | | | o |
| interferon-induced 17 kda protein precursor | 4 | o | | o | | | | | |
| intraflagellar transport protein 20 homolog | 4 | o | | o | o | o | | | |
| iq motif and wd repeats 1 | 4 | o | | o | | | o | o | |
| iron-sulfur assembly mitochondrial precursor | 4 | o | o | | | o | o | | |
| iron-sulfur cluster assembly 1 homolog ( cerevisiae) | 4 | o | | | | o | o | o | |
| jeltraxin precursor | 4 | | o | | o | o | | o | |
| jumonji domain containing 2c | 4 | o | | o | | | o | o | |
| kcnab2 protein | 4 | o | | | | | | | |
| kda protein | 4 | o | | o | | o | | o | |
| kdel (lys-asp-glu-leu) endoplasmic reticulum protein retention receptor 2 | 4 | o | o | o | | | | | |
| kiaa2032 protein | 4 | o | | o | | o | | o | |
| kinesin family member partial | 4 | o | | | o | o | | | |

| | | | | | | | | | |
|---|---|---|---|---|---|---|---|---|---|
| kruppel-like factor 11 | 4 | o | o | | | | o | | |
| lambda-crystallin homolog | 4 | o | o | | | | | o | |
| laminin subunit beta-1 | 4 | o | | | | o | o | | |
| large homolog 5 | 4 | o | | o | | | | o | o |
| large proline-rich protein bat3 | 4 | | o | o | | | | | o |
| ldlr chaperone mesd | 4 | o | o | o | | | | o | |
| lemur tyrosine kinase 2 | 4 | o | | o | | o | | | |
| leucine-rich glioma-inactivated protein 1 precursor | 4 | | | | | | | o | o |
| leucine-rich repeats and calponin homology domain containing 1 | 4 | o | | | | | o | | |
| leucine-zipper-like transcription regulator 1 | 4 | o | | | | o | o | o | |
| leucine-zipper-like transcriptional regulator 1 | 4 | | o | o | | | | | o |
| leucyl-trna cytoplasmic | 4 | o | | o | o | | | o | |
| lim and senescent cell antigen-like-containing domain protein 1 | 4 | o | o | | | | | | o |
| lim domain 7 | 4 | o | o | | | o | | | |
| lim domain binding 3 | 4 | o | | | | | | | |
| lin-52 homolog | 4 | o | o | | | o | | o | |
| lish domain and heat repeat-containing protein kiaa1468-like | 4 | o | o | | | | o | o | |
| loc100127275 protein | 4 | o | | | | | | | |
| loc398864 protein | 4 | o | | o | | o | | | |
| loc407663 protein | 4 | o | | | | o | | | o |
| loc553397 protein | 4 | o | | | | o | | o | |
| loc553430 protein | 4 | o | | o | | o | | o | |
| loc734098 protein | 4 | | | | | | | o | o |
| lon peptidase n-terminal domain and ring finger 1 | 4 | o | | | | | o | o | |
| low quality protein: desmoplakin-like | 4 | o | o | o | | | | | |
| lpxtg-motif cell wall anchor domain protein | 4 | o | | | o | o | | | |
| lyr motif-containing protein 7 | 4 | o | o | | | o | | o | |
| lyric protein | 4 | | o | o | | o | | | |
| lysophosphatidylcholine acyltransferase 2 | 4 | o | o | | | | | | |
| lysosomal-associated membrane protein 1 | 4 | o | | | | o | | o | |
| lysosomal-associated membrane protein 2 | 4 | o | | | | | o | | o |
| macrophage erythroblast attacher | 4 | o | o | | o | | | o | |
| malate dehydrogenase | 4 | o | o | o | | | | | |
| maltase- intestinal | 4 | | o | | o | | o | | |
| mannose- 1 | 4 | o | o | o | | | | | |
| map-kinase activating death domain | 4 | o | | | | | o | | |
| matrix-remodelling associated 7 | 4 | o | | o | | | | | |
| max protein | 4 | o | | o | | | o | | |
| mediator complex subunit 13 | 4 | o | | | | | o | o | |
| mediator of rna polymerase ii transcription subunit 11 | 4 | o | | o | | o | | | |
| membrane palmitoylated 6 (maguk p55 subfamily member 6) | 4 | o | o | o | | | | | |
| mercaptopyruvate sulfurtransferase | 4 | o | o | o | | | | | |
| mesoderm specific transcript | 4 | o | o | | | | | | o |
| metalloprotease c21orf57 | 4 | o | | o | | o | o | | |
| metalloproteinase inhibitor 2 precursor | 4 | o | | o | | | | | |
| methyl- -binding domain protein 3 | 4 | o | o | | | | o | | |
| methylosome subunit picln | 4 | o | o | | | | | | o |
| mgc82104 protein | 4 | o | | | o | | | | |
| mhc class i antigen | 4 | o | o | | o | | | | |
| microtubule-associated protein 2 | 4 | | | | | | o | o | o |
| midline 1 | 4 | o | | | | | o | | |
| mitochondrial atp h+ transporting f1 complex beta subunit | 4 | | | | o | | | | o |

| | | | | | | | | | |
|---|---|---|---|---|---|---|---|---|---|
| mitochondrial endonuclease g | 4 | o | o | | | | | | o |
| mitochondrial enolase superfamily member 1 | 4 | o | o | | | | | | |
| mitochondrial import inner membrane translocase subunit tim23 | 4 | | | | o | o | | | |
| mitochondrial inner membrane protease atp23 homolog | 4 | o | o | | o | o | | | |
| mitochondrial inner membrane protein oxa1l precursor | 4 | o | o | o | | | | | |
| mitochondrial nadh dehydrogenase ubiquinone flavoprotein 1 | 4 | o | o | o | | | | | |
| mitochondrial ribosomal protein 63 | 4 | o | o | | | o | o | | |
| mitochondrial ribosomal protein l17 | 4 | o | o | | | o | o | | |
| mitochondrial ribosomal protein l37 | 4 | o | | o | | o | | o | |
| mitochondrial ribosomal protein s14 | 4 | o | | o | | o | | o | |
| mitochondrial ribosomal protein s25 | 4 | o | o | o | | o | | | |
| mitochondrial ribosomal protein s31 | 4 | o | o | | | o | | | |
| mitogen-activated protein kinase 1 | 4 | | | | o | | o | o | |
| mitogen-activated protein kinase 13 | 4 | o | | o | | o | | o | |
| mitogen-activated protein kinase kinase kinase kinase 5 | 4 | o | | | | | | | |
| mortality factor 4-like protein 1 | 4 | | | o | o | o | | o | |
| mps one binder kinase activator-like 2a | 4 | | o | o | o | o | | | |
| mps one binder kinase activator-like 2-like | 4 | o | o | o | o | | | | |
| mps one binder kinase activator-like 3 | 4 | o | o | o | | | | o | |
| mterf domain containing 1 | 4 | o | o | o | | | | | |
| muscleblind-like 1 isoform 1 | 4 | o | | | | | | | |
| muscleblind-like 2 | 4 | o | | | | o | | o | o |
| myc-induced nuclear antigen-like | 4 | | | | o | | | | o |
| myelin protein zero | 4 | o | | | | | o | o | |
| myeloid lymphoid or mixed-lineage leukemia 2 | 4 | o | | o | | o | | | |
| myeloid-associated differentiation marker | 4 | o | o | | | | | | |
| myocyte enhancer factor 2c protein | 4 | o | | | | | | | |
| myo-inositol oxygenase | 4 | | o | o | o | | | | |
| myomesin member 3 | 4 | o | | | | | | | |
| myosin binding protein cardiac | 4 | o | | | o | | | | |
| myosin light chain smooth muscle | 4 | o | o | | o | | | | |
| myosin-binding protein fast-type | 4 | o | | | | | | | |
| n-acetylglucosamine kinase | 4 | o | o | o | | | | | |
| n-acetyltransferase 5 | 4 | o | o | | o | | | | o |
| n-acetyltransferase 9 | 4 | o | | | | o | | | o |
| n-acetyltransferase nat13 | 4 | | o | o | o | | | | o |
| n-acylsphingosine amidohydrolase (acid ceramidase) 1 | 4 | o | | o | | o | | o | |
| nadh dehydrogenase 1 alpha assembly factor 2 | 4 | o | | | | o | | o | o |
| nadh dehydrogenase 1 alpha subcomplex subunit 6 | 4 | o | | o | | o | o | | |
| nadh dehydrogenase 1 subunit c2 | 4 | o | o | o | | | | | |
| nadh dehydrogenase subunit 6 | 4 | o | | o | | | o | o | |
| nadh-ubiquinone oxidoreductase variant 1 | 4 | o | o | | | o | o | | |
| nap homolog 2 | 4 | o | o | | | | | o | o |
| nardilysin precursor | 4 | | o | o | | o | | | |
| n-arginine dibasic nrd convertase 1 | 4 | o | o | o | | | o | | |
| natterin-like protein | 4 | o | | o | | | | | |
| n-deacetylase n-sulfotransferase (heparan glucosaminyl) 1 | 4 | o | o | | | | | o | |
| nei endonuclease viii-like 1 ( coli) | 4 | o | | | | o | | o | |
| neurocalcin-delta | 4 | o | | | | | o | o | |
| neurogranin | 4 | | | | | | | o | o |

| | | | | | | | | | |
|---|---|---|---|---|---|---|---|---|---|
| neuronal acetylcholine receptor subunit alpha-7-like | 4 | | | | | | o | o | o |
| neuron-specific protein family member 1 | 4 | | | | | | | | o |
| neuropathy target esterase | 4 | | o | | | | o | | |
| nfkb activating | 4 | o | o | o | | o | | | |
| nidogen 1 | 4 | o | | | | | | | |
| nipped-b-like protein | 4 | | | | o | o | o | | o |
| n-myc downstream regulated gene 3 | 4 | o | | | | | o | o | |
| n-myristoyltransferase 2 | 4 | | o | o | o | | | | o |
| non-lysosomal glucosylceramidase | 4 | | o | | o | | | | o |
| non-specific cytotoxic cell receptor protein 1 homolog | 4 | o | o | o | | o | | | |
| non-structural maintenance of chromosomes element 1 homolog | 4 | o | o | | | | | o | |
| novel protein glutaminase | 4 | o | | | | | | | o |
| novel protein titin | 4 | o | | | | | | | |
| novel protein vertebrate ankyrin neuronal ( zgc:101738) | 4 | o | | | | | o | o | |
| novel protein vertebrate nebulin | 4 | o | | | | | | | |
| novel protein vertebrate sorbin and sh3 domain containing family | 4 | o | | o | | o | o | | |
| novel serine protease protein | 4 | o | | | | o | o | | |
| novel synuclein domain containing protein (zgc:110133) | 4 | | | | | | o | | o |
| nuclear factor erythroid 2-related factor 2 | 4 | o | o | | | o | | | |
| nuclear factor of activated t-cells 5 | 4 | o | | | | | o | | |
| nuclear nucleic acid-binding protein c1d | 4 | o | | o | o | | | | |
| nuclear receptor subfamily 4 group a member 1 | 4 | o | | | | | | | |
| nuclear transcription factor alpha | 4 | o | | o | | | | | o |
| nuclear transcription factor y subunit gamma | 4 | | o | o | | | | o | |
| nucleolar protein 16-like | 4 | | | | o | | | | o |
| nucleolysin tiar | 4 | o | o | | | | | o | |
| nucleoporin 54kda | 4 | o | o | o | | | | o | |
| nucleoporin nup37 | 4 | o | o | | | o | o | | |
| nucleosome assembly protein 1-like 4 | 4 | o | o | | o | | | | |
| obg-like atpase 1 | 4 | o | o | | o | | | | |
| organic solute transporter subunit alpha | 4 | o | o | | o | | | | |
| ornithine aminotransferase | 4 | o | o | o | | | | | |
| orphan 1 | 4 | o | o | | | o | | | |
| otu domain-containing protein 6b | 4 | o | o | o | | | | | |
| p2y purinoceptor 11 | 4 | o | o | o | | | | | |
| paladin | 4 | o | | | | o | | o | |
| pantophysin | 4 | o | o | o | | | | | |
| pantothenate kinase 2 (hallervorden-spatz syndrome) | 4 | o | | | | | | | |
| pantothenate kinase 4 | 4 | o | o | | | o | | | |
| paraoxonase 2 | 4 | o | | | o | o | | | o |
| pc4 and sfrs1 interacting protein 1 | 4 | o | | | | | | o | |
| pctaire protein kinase 2 | 4 | o | o | | | | o | o | |
| pdz and lim domain 5 | 4 | o | | | | | o | | |
| peptidoglycan recognition protein | 4 | | o | o | o | | | | |
| peptidyl-prolyl cis-trans isomerase fkbp2-like | 4 | | | | o | | | | o |
| peptidyl-prolyl cis-trans isomerase fkbp4 | 4 | o | | | | | | | o |
| peptidyl-prolyl cis-trans isomerase nima-interacting 1 | 4 | o | | o | | o | | o | |
| peptidyl-prolyl cis-trans isomerase-like 2 | 4 | o | | o | | | | | |
| peptidylprolyl isomerase domain and wd repeat containing 1 | 4 | o | o | | | o | o | | |

| | | | | | | | | | |
|---|---|---|---|---|---|---|---|---|---|
| periodic tryptophan protein 1 homolog | 4 | o | o | | | | | | o |
| peroxiredoxin-1 | 4 | | | | o | | | | o |
| peroxisomal acyl-coenzyme a oxidase 3 | 4 | o | o | | | | | | |
| peroxisomal membrane protein 4 | 4 | o | o | | | o | | o | |
| peroxisomal trans-2-enoyl- reductase | 4 | o | o | o | | | | | |
| pescadillo homolog | 4 | | o | o | | | | | o |
| phenylalanine hydroxylase | 4 | | o | o | o | | | | |
| phosphatase and actin regulator 4 | 4 | o | | | | | | | |
| phosphatidylinositol n-acetylglucosaminyltransferase subunit y | 4 | o | o | o | | o | | | |
| phosphatidylinositol-glycan biosynthesis class x protein precursor | 4 | o | | o | | o | o | | |
| phosphodiesterase 4d interacting protein | 4 | o | | | | | | | |
| phosphoenolpyruvate carboxykinase | 4 | | o | o | | | | | o |
| phosphoethanolamine n-methyltransferase | 4 | | o | o | | | | | |
| phospholipase gamma 1 | 4 | o | | | | | o | | |
| phospholipase group xiib | 4 | | o | o | o | | | | |
| phosphomannomutase 1 | 4 | | o | o | | | | | o |
| phosphoribosyl pyrophosphate synthetase 1 | 4 | o | o | o | | | | | |
| phytanoyl- 2-hydroxylase | 4 | o | o | o | o | | | | |
| piggybac transposable element derived 4 | 4 | o | | | | | o | | |
| pituitary tumor-transforming 1 interacting protein | 4 | o | | o | o | o | | | |
| plasma membrane calcium-transporting atpase 1 | 4 | o | | | | | o | o | |
| plasma membrane calcium-transporting atpase 2 | 4 | | | | | | o | o | |
| platelet-activating factor acetylhydrolase ib subunit alpha | 4 | | o | | | | o | | |
| platelet-activating factor acetylhydrolase ib subunit gamma | 4 | o | | | | | | o | o |
| platelet-derived growth factor receptor beta | 4 | o | | o | | | | o | |
| pleckstrin homology domain-containing family b member 2 | 4 | o | | | | | o | o | |
| plexin a4 | 4 | o | | | | | o | | |
| poly (adp-ribose) glycohydrolase | 4 | o | o | | | | | o | |
| poly (adp-ribose) polymerase member 6 | 4 | o | | | | | o | o | |
| poly binding cytoplasmic 1 | 4 | | o | o | | o | | o | |
| poly -binding protein 2 | 4 | o | o | | | | | o | |
| poly binding protein interacting protein 1 | 4 | o | o | o | | | | | |
| polyadenylate-binding protein 1 | 4 | | o | | | | o | | o |
| polymerase i and transcript release factor | 4 | o | | | | | | | |
| polymeric immunoglobulin receptor | 4 | o | o | o | | | | | |
| ppig protein | 4 | o | | o | | o | | o | |
| PREDICTED: lambda-recombinase-like protein-like [Danio rerio] | 4 | o | | o | | | | o | |
| prefoldin subunit 1 | 4 | o | o | | | o | | | o |
| pre-mrna-processing factor 17 | 4 | o | | o | | o | | | |
| prenyl diphosphate subunit 2 | 4 | o | | o | | | | | |
| prestin | 4 | o | o | | | | | | |
| probable atp-dependent rna helicase ddx41 | 4 | o | | | o | | | | |
| probable e3 ubiquitin-protein ligase herc1-like | 4 | o | o | | | | o | | |
| probable lysosomal cobalamin transporter | 4 | o | | o | | | | o | |
| probable trna pseudouridine synthase 2 | 4 | o | o | o | | o | | | |
| probable u3 small nucleolar rna-associated protein 11 | 4 | o | o | o | | o | | | |
| programmed cell death protein 4 | 4 | | o | | | o | | | o |
| prolactin regulatory element-binding protein | 4 | | o | | | | | | o |
| proliferation-associated protein 2g4 | 4 | | o | o | | | o | | |
| proline-rich nuclear receptor coactivator 2 | 4 | o | o | o | | | o | | |

| | | | | | | | | | |
|---|---|---|---|---|---|---|---|---|---|
| propionyl coenzyme a beta polypeptide | 4 | o | o | | | o | | | |
| propionyl-coenzyme a alpha polypeptide | 4 | o | o | | | | | | |
| proprotein convertase subtilisin kexin type 5 | 4 | | o | o | | | | | |
| proteasome ( macropain) 26s 4 | 4 | o | o | o | | | | | |
| proteasome ( macropain) 26s non- 11 | 4 | o | o | | | o | | | |
| proteasome ( macropain) 26s non- 6 | 4 | o | o | o | | o | | | |
| proteasome assembly chaperone 1 | 4 | o | | | o | | | o | |
| proteasome assembly chaperone 3 | 4 | o | | o | | o | | | o |
| proteasome subunit beta type-6 precursor | 4 | | o | | | o | | o | |
| protein amp- gamma 3 non-catalytic subunit | 4 | o | | | | | | | |
| protein atp1b4-like | 4 | | | | | | | | o |
| protein c10 | 4 | | | o | | o | o | o | |
| protein c20orf11 homolog | 4 | o | o | o | | | | | |
| protein disulfide isomerase family member 3 precursor | 4 | | | | o | | | | o |
| protein disulfide isomerase family member 4 | 4 | o | o | o | o | | | | |
| protein dj-1 | 4 | o | | o | | | | | o |
| protein dpcd | 4 | o | | o | o | | | o | |
| protein fam46a-like | 4 | | | | o | | | | |
| protein itfg3 | 4 | | o | | o | | | | o |
| protein ltv1 homolog | 4 | o | o | o | | | | | |
| protein nlrc3-like | 4 | | o | | | | | | o |
| protein phosphatase 1h (pp2c domain containing) | 4 | o | | | | | o | o | |
| protein phosphatase 1k (pp2c domain containing) | 4 | o | o | o | | | | | |
| protein phosphatase 1l | 4 | o | o | | | | o | | |
| protein phosphatase regulatory subunit 12b | 4 | o | | | | | | | |
| protein phosphatase regulatory subunit 14a | 4 | o | | | | o | | o | o |
| protein piccolo isoform 1 | 4 | | | | | | o | o | |
| protein s | 4 | o | o | o | | | | | |
| protein s100-z-like | 4 | | | | o | | | | |
| protein sda1 homolog | 4 | o | o | o | | o | | | |
| protein strawberry notch homolog 1 | 4 | o | | o | | o | | o | |
| protein tyrosine phosphatase-like (proline instead of catalytic arginine) member b | 4 | | o | o | o | | o | | |
| protein tyrosine receptor u | 4 | | | | | o | o | | |
| protein yipf5 | 4 | o | o | o | | | | | |
| protein-glutamine gamma-glutamyltransferase 2 | 4 | o | o | o | | | | | |
| prothrombin precursor | 4 | | | | o | | | | o |
| protocadherin 1 | 4 | | | | | | o | o | |
| protocadherin 1 gamma 31 | 4 | | | | | o | o | o | |
| protocadherin 2 gamma 29 | 4 | o | | | | | o | o | |
| protocadherin 2a3-like | 4 | | | | | | | o | |
| protocadherin-10-like | 4 | | | | | | o | | |
| px domain-containing protein c6orf145 homolog | 4 | | o | o | o | | | | |
| pyrroline-5-carboxylate reductase 1 | 4 | | o | o | | | | | o |
| pyruvate carboxylase | 4 | o | o | o | | | | | |
| pyruvate kinase | 4 | o | | | | | o | | o |
| rab acceptor 1 | 4 | o | o | o | | | | | o |
| rab gdp dissociation inhibitor beta | 4 | | | | | | o | o | o |
| rab interacting factor | 4 | o | | | | o | o | | |
| ran binding protein 9 | 4 | o | o | o | | | | | |
| rapunzel 4-like | 4 | o | | o | | o | | o | |
| ras homolog gene member c | 4 | o | | o | | o | | | |
| ras homolog gene member u | 4 | o | o | | | | | | |
| ras-related protein rab-8a | 4 | o | o | o | | | | o | |
| ras-related protein rap-1b precursor | 4 | o | o | o | | | | | |

| | | | | | | | | | |
|---|---|---|---|---|---|---|---|---|---|
| rb1-inducible coiled-coil 1 | 4 | o | | | | | | | |
| receptor tyrosine kinase | 4 | o | o | | o | | | | |
| receptor-transporting protein 3 | 4 | o | | o | | | | | |
| regeneration associated muscle protease | 4 | o | | | | | | | |
| regulator of g-protein signaling 7-binding protein | 4 | | | | | o | o | o | |
| renin receptor isoform 3 | 4 | | | | o | | | | o |
| reticulocalbin ef-hand calcium binding domain | 4 | o | o | o | | | | | |
| reticulon 1a | 4 | o | o | o | | | | | |
| retinoblastoma binding protein 6 | 4 | o | o | o | | | | | |
| retinoblastoma-like 1 | 4 | o | | | | | o | | |
| rho gdp dissociation inhibitor alpha | 4 | o | | | | | o | | |
| rho gtpase activating protein 21 | 4 | o | | | | o | o | o | |
| rho gtpase-activating protein | 4 | o | | | | | o | o | |
| rhoa activator c11orf59 homolog | 4 | o | o | | | | | o | |
| ribokinase | 4 | o | o | o | | | | | o |
| ribosomal protein l18a | 4 | o | | o | | o | | o | |
| ribosomal protein l36a-like | 4 | o | | o | | o | | o | |
| ribosomal protein l37 | 4 | o | o | o | | | o | | |
| ribosomal protein s14 | 4 | o | | o | | o | o | | |
| ribosomal protein s2 | 4 | o | | o | | | | o | |
| ribosomal protein s21 | 4 | o | o | | o | o | | | |
| ribosomal protein s3 | 4 | o | o | o | | o | | | |
| ribosomal protein s5 | 4 | o | | o | | o | | o | |
| ribosomal protein s6 polypeptide 1 | 4 | o | | | | | o | | |
| ribosomal rna processing 12 homolog ( cerevisiae) | 4 | o | o | o | | | | | |
| riia domain-containing protein | 4 | | o | | | o | | o | |
| riken cdna 6330578e17 variant 1 | 4 | o | | o | | o | | o | |
| ring finger protein 103 | 4 | o | o | | | | | | |
| ring finger protein 115 | 4 | o | o | o | | | | | |
| ring finger protein 170 | 4 | o | | o | | o | | | |
| rna binding motif protein 10 | 4 | o | | | o | | | | o |
| rna binding single stranded interacting protein 3-like | 4 | o | | | | | | o | |
| rna-binding protein with multiple splicing | 4 | | | o | | o | o | o | |
| ryanodine receptor | 4 | o | | | | | o | | |
| s2 ribosomal protein | 4 | | | | o | o | | | |
| s-adenosylhomocysteine hydrolase | 4 | o | o | | | | | | o |
| s-adenosylmethionine decarboxylase | 4 | | | | | | o | o | |
| sb:cb37 protein | 4 | o | o | o | | | | | |
| scan domain containing partial | 4 | o | o | | | o | o | | |
| sec23b ( cerevisiae) | 4 | o | o | | | | | | |
| secernin 3 | 4 | o | o | o | o | | | | |
| secretogranin iii | 4 | | | | | o | | o | o |
| secretory carrier membrane protein 1 | 4 | o | | | | o | o | | |
| selenoprotein i | 4 | o | o | o | | | | o | |
| selenoprotein k | 4 | o | o | o | | o | | | |
| selenoprotein s | 4 | | o | o | o | | | | |
| sema transmembrane domain and cytoplasmic 6d | 4 | | | | o | | o | o | |
| sentrin-specific protease 7 | 4 | | o | | | | | | o |
| septin 7 | 4 | o | o | | | | | | |
| septin 9 | 4 | o | o | | | | o | o | |
| septin-7 isoform 2 | 4 | o | o | | | | o | | o |
| sequestosome 1 | 4 | o | o | | | | | | |
| serine arginine repetitive matrix 1 | 4 | o | | o | | o | | | o |
| serine arginine-rich protein specific kinase 1 | 4 | o | | | | o | | | |
| serine carboxypeptidase 1 precursor protein | 4 | o | | | | o | | | |
| serine cytosolic | 4 | | | | o | o | | | o |

| | | | | | | | | | |
|---|---|---|---|---|---|---|---|---|---|
| serine hydroxymethyltransferase 1 | 4 | o | o | o | | | | | |
| serine incorporator 1 precursor | 4 | | | | | | | | o |
| serine protease 23 precursor | 4 | o | | o | | | | | o |
| serine threonine kinase 24 (ste20 yeast) | 4 | o | | o | | | | o | |
| serine threonine tyrosine-interacting protein | 4 | o | | | | o | | | |
| serine threonine-protein kinase pim-3 | 4 | o | o | o | | | | | |
| serine threonine-protein kinase pim-3-like | 4 | o | | | | o | | o | |
| serine threonine-protein phosphatase 2a catalytic subunit alpha isoform | 4 | o | | | | | | o | o |
| serine threonine-protein phosphatase 2b catalytic subunit alpha isoform | 4 | o | | | | | o | o | |
| sestrin 1 | 4 | o | | o | | | o | | |
| set and mynd domain-containing protein 1 | 4 | o | | | | | | | |
| seven transmembrane helix receptor | 4 | o | | | | o | o | | |
| sh3 and cysteine-rich domain-containing protein 3 | 4 | o | | | | | | | |
| sh3-domain kinase binding protein 1 | 4 | o | | | | | | | |
| short transient receptor potential channel 1 | 4 | | | | | o | | o | o |
| signal peptidase complex subunit 3 | 4 | | o | o | | | | | |
| signal transducer and activator of transcription 3 | 4 | o | | o | | | | o | |
| single-stranded dna-binding mitochondrial precursor | 4 | | | | o | o | | | |
| sjogren syndrome scleroderma autoantigen 1 | 4 | o | o | | o | o | | | |
| slit and ntrk-like member 2 | 4 | | | o | | | o | o | |
| slit homolog 1 | 4 | o | | | | | o | o | |
| slow troponin t 1 | 4 | o | | | | | | | |
| small nuclear ribonucleoprotein g | 4 | | | | o | | | | |
| sodium calcium exchanger 1 | 4 | o | | | | o | o | o | |
| sodium channel protein type 8 subunit alpha isoform 2 | 4 | | | | | | o | o | |
| solute carrier family 1 (glial high affinity glutamate transporter) member 2 | 4 | | | | | o | o | o | |
| solute carrier family 1 (glial high affinity glutamate transporter) member 3 | 4 | o | | o | | | | | o |
| solute carrier family 23 member 2-like | 4 | | o | o | | | | | |
| solute carrier family 25 (mitochondrial aralar) member 12 | 4 | o | | | | | | o | o |
| solute carrier family 25 (mitochondrial carrier peroxisomal membrane 34kda) member 17 | 4 | o | o | o | | | | o | |
| solute carrier family 25 (mitochondrial carrier: glutamate) member 22 | 4 | o | | o | | | | o | |
| solute carrier family 25 member 40 | 4 | o | o | o | | | | | o |
| solute carrier family 25 member 42 | 4 | o | | | | | | | o |
| solute carrier family 27 (fatty acid transporter) member 1 | 4 | o | | | | o | | o | |
| solute carrier family 27 (fatty acid transporter) member 4 | 4 | | | | | | | o | |
| solute carrier family 5 (choline transporter) member 7 | 4 | o | | | | | o | o | |
| solute carrier family member 33 | 4 | o | o | | | | | | |
| solute carrier family member 34 | 4 | o | o | | | | | | |
| solute carrier family member 43 | 4 | o | | | | | o | | |
| solute carrier family member 4-like | 4 | o | | o | | o | | | |
| solute carrier organic anion transporter family member 1c1-like | 4 | | o | | o | | | | |
| sorting nexin 3 | 4 | o | o | | | | o | | |
| sphingosine-1-phosphate phosphatase 1 | 4 | o | o | o | o | | | | |
| spindlin protein c | 4 | o | | | o | o | o | | |
| splicing arginine serine-rich 8 | 4 | o | o | | | o | | | |

| | | | | | | | | | |
|---|---|---|---|---|---|---|---|---|---|
| staphylococcal nuclease domain containing 1 | 4 | o | | o | | o | | | |
| sterile alpha motif domain containing 9-like | 4 | o | | o | | o | | o | |
| sterol carrier protein-2 | 4 | | | | o | | | | o |
| stomatin -like 1 | 4 | o | o | o | | o | | | |
| storkhead box 2 | 4 | o | | | | o | o | | |
| stromal interaction molecule 1 | 4 | o | o | o | | | o | | |
| subfamily member 2 | 4 | o | o | | | | | | |
| subfamily member 5 | 4 | o | | o | | | o | | |
| succinate- gdp- alpha subunit | 4 | o | o | | | | o | o | |
| suppression of tumorigenicity 13 (colon carcinoma) (hsp70 interacting protein) | 4 | o | | | o | o | | o | |
| suppressor of fused homolog | 4 | o | | o | | | o | o | |
| sushi domain containing 2 | 4 | | o | o | | | | | |
| svil protein | 4 | o | | | o | | | | |
| swi snf matrix actin dependent regulator of subfamily member 5 | 4 | o | | | | | | o | |
| synaptogyrin 2 | 4 | o | | o | | o | | | |
| synaptopodin 2-like | 4 | o | | | | | | | |
| synaptosomal-associated protein 25 | 4 | | | | o | | | o | o |
| synaptotagmin ii | 4 | | | | | | o | o | |
| syntaxin 12 | 4 | o | | | | o | | | o |
| syntaxin 5 | 4 | o | o | o | | | | | |
| syntaxin-binding protein 3 | 4 | o | o | | | | | | o |
| taf3 protein | 4 | o | | o | | o | | | |
| taurine transporter | 4 | o | | | | o | | o | |
| t-complex 1 | 4 | o | o | o | | | | o | |
| t-complex protein 1 subunit beta | 4 | | o | | o | | | | o |
| t-complex protein 11-like protein 2 | 4 | | o | o | o | | | | o |
| tescalcin | 4 | o | | | | o | | | o |
| testis enhanced gene transcript (bax inhibitor 1) | 4 | o | o | o | | | | o | |
| tetraspanin 17 | 4 | o | | | | | | | |
| tetraspanin 8 | 4 | o | o | | o | | | | o |
| tetratricopeptide repeat protein 9c | 4 | o | o | o | | o | | | |
| thap domain apoptosis associated protein 2-like | 4 | o | | | | | | | |
| thap domain-containing protein 4 | 4 | o | | o | | | | o | o |
| thioredoxin-like protein 4a | 4 | o | | o | | o | o | | |
| thiosulfate sulfurtransferase kat | 4 | o | o | o | | o | | | |
| tho complex 2 | 4 | o | o | | | | o | o | |
| tho complex 3 | 4 | | o | | o | | | | |
| thyroid hormone receptor interactor isoform cra_a | 4 | o | o | | | | o | | o |
| tissue specific transplantation antigen p35b | 4 | o | | o | | | o | o | |
| tkt protein | 4 | o | | | o | | | o | o |
| torsin family member b (torsin b) | 4 | o | o | | | o | | | o |
| trafficking protein particle complex 4 | 4 | o | | o | | o | | o | |
| transcription elongation factor a 3 | 4 | o | | o | | | | | |
| transcription factor cp2-like protein 1 | 4 | | o | | | | | | |
| transcription factor dp-1 | 4 | o | o | | | | | | o |
| transcription factor iib | 4 | o | o | o | | o | | | |
| transcription factor mitochondrial | 4 | o | o | | | | | | |
| transcription initiation factor tfiid subunit 7 | 4 | o | o | o | | o | | | |
| translation initiation factor eif-2b subunit beta | 4 | | | o | o | | | | o |
| translational activator gcn1 | 4 | | | | o | | o | | o |
| translocase of inner mitochondrial membrane 44 homolog | 4 | o | | | | | | | |
| translocase of inner mitochondrial membrane 8 homolog b | 4 | o | o | o | | o | | | |
| transmembrane 9 superfamily member 2 | 4 | o | o | o | | | | | o |

| | | | | | | | | | |
|---|---|---|---|---|---|---|---|---|---|
| transmembrane channel-like protein 7 | 4 | o | o | | o | | | | o |
| transmembrane protein 11 | 4 | o | o | | | | o | | |
| transmembrane protein 129 | 4 | o | | o | | | | | |
| transmembrane protein 131 | 4 | o | o | | | | | | |
| transmembrane protein 134 | 4 | o | | o | | o | | o | |
| transmembrane protein 147 | 4 | o | o | o | | o | | | |
| transmembrane protein 165 | 4 | o | o | o | | | | o | |
| transmembrane protein 177 | 4 | o | o | | | | | | |
| transmembrane protein 188 | 4 | o | o | o | | o | | | |
| transmembrane protein 198 | 4 | o | | | | o | | | o |
| transmembrane protein 205 | 4 | o | | | o | | | | |
| transmembrane protein 208 | 4 | o | | o | | o | | o | |
| transmembrane protein 32 | 4 | o | | o | | o | | o | |
| transmembrane protein 41a | 4 | o | o | | | | | | |
| transmembrane protein 53 | 4 | o | o | o | | o | | | |
| transmembrane protein 82 | 4 | | o | | | | o | | |
| transmembrane protein 90b-like | 4 | o | | o | | o | | o | |
| transport protein sec61 subunit gamma | 4 | o | | o | | o | | o | |
| trimeric intracellular cation channel type a | 4 | o | | | | | | | |
| trm112-like protein | 4 | o | o | o | | o | | | |
| trna (guanine-n -)-methyltransferase | 4 | | | o | o | | | | |
| trna selenocysteine 1-associated protein 1-like | 4 | | | o | o | | | | o |
| troponin t | 4 | o | | | | | | | |
| tryptophan-rich protein | 4 | o | o | o | | o | | | |
| tubulin alpha-1a chain | 4 | | | | | | | | o |
| tubulin-specific chaperone e | 4 | | | o | | | o | | o |
| tumor necrosis factor receptor superfamily member 14 precursor | 4 | o | o | o | | | | | |
| tumor protein d52 | 4 | o | | o | | | | o | |
| twinfilin-1 | 4 | | o | | | | o | | o |
| type-iv antifreeze protein | 4 | | o | | o | | | | |
| tyrosine recombinase-like | 4 | o | | o | | | o | o | |
| tyrosylprotein sulfotransferase 2 | 4 | | o | | | | | | o |
| u4 small nuclear ribonucleoprotein 27 kda protein | 4 | o | o | o | | o | | | |
| ubiquilin 4 | 4 | o | o | | | | | | o |
| ubiquinone biosynthesis protein coq7 homolog | 4 | o | o | | | o | | | |
| ubiquinone biosynthesis protein mitochondrial precursor | 4 | o | o | | | o | | | |
| ubiquitin specific peptidase 54 | 4 | o | | | | | | o | |
| ubiquitin thioesterase otub2 | 4 | o | o | | | | o | | o |
| ubiquitin-conjugating enzyme e2 e2 | 4 | o | o | | | | o | o | |
| ubiquitin-protein ligase e3b | 4 | o | o | | | | o | o | |
| udp glucuronosyltransferase 5 family polypeptide b4 | 4 | | o | | | | | | |
| udp-glucose dehydrogenase | 4 | o | o | | | o | | | |
| udp-n-acetylglucosamine--dolichyl-phosphate n-acetylglucosaminephosphotransferase | 4 | o | o | o | | | | | |
| uncharacterized peroxidase-related enzyme | 4 | o | o | o | | | | o | |
| uncharacterized protein c10orf71-like | 4 | o | | | | | | | |
| uncharacterized protein kiaa1107-like | 4 | | o | | | | o | | |
| uncharacterized protein kiaa1704-like | 4 | o | o | o | | | | o | |
| uncharacterized protein loc535121 isoform 4 | 4 | | | | | | | | o |
| uncharacterized protein mitochondrial-like | 4 | | o | | o | | | | |
| upf0139 membrane protein c19orf56 homolog | 4 | o | o | o | | o | | | |
| upf0480 protein c15orf24 homolog | 4 | | | | | | | | o |
| upf0493 protein kiaa1632-like | 4 | o | | | | | | | |
| upf0510 protein inm02-like | 4 | | | | o | | | | o |
| upf0697 protein c8orf40 homolog | 4 | | | | o | | | | o |

| | | | | | | | | | |
|---|---|---|---|---|---|---|---|---|---|
| uridine-cytidine kinase 1-like 1 | 4 | o | | | | | o | | |
| urocanase domain containing 1 | 4 | | o | o | | | | | |
| vacuolar protein sorting 35 | 4 | o | o | o | | | | | |
| vacuolar protein sorting-associated protein 18 homolog | 4 | | o | | | | | | o |
| vacuolar protein sorting-associated protein 28 homolog | 4 | o | o | o | | | | o | |
| vacuolar protein-sorting-associated protein 25 | 4 | o | | o | o | o | | | |
| vacuolar protein-sorting-associated protein 36 | 4 | | o | o | | o | | | |
| valyl-trna synthetase | 4 | o | | o | | | o | | |
| v-erb-a erythroblastic leukemia viral oncogene homolog 4 | 4 | o | o | | | | o | | |
| vesicle-associated membrane protein-associated protein a | 4 | o | o | o | | | o | | |
| vimentin | 4 | | | | | o | | o | o |
| vinculin isoform 1 | 4 | o | | | | | | | |
| vitamin k epoxide reductase subunit 1-like 1 | 4 | o | | o | | | o | o | |
| voltage-dependent l-type ca channel alpha 1 subunit | 4 | o | | | | | | | |
| von willebrand factor a domain containing 5a-like | 4 | o | | o | | o | | | |
| von willebrand factor a domain-containing protein 5a-like | 4 | | o | | | | | | o |
| v-type proton atpase subunit c 1 | 4 | | | | | o | | | o |
| v-type proton atpase subunit s1-like | 4 | | | | o | | o | | o |
| wd repeat domain 43 | 4 | o | o | o | | | | | |
| wd repeat domain 5 | 4 | o | o | | | | o | o | |
| zgc:158463 protein | 4 | o | | o | | o | | o | |
| zinc finger homeobox protein 3-like | 4 | | | | | | o | | |
| zinc finger mym-type protein 1-like | 4 | | | | o | | o | | |
| zinc finger protein 106 homolog | 4 | o | | | | | | | |
| zinc finger protein 277 | 4 | o | | o | | o | o | | |
| zinc finger protein 593 | 4 | o | o | o | | | | o | |
| zinc finger protein c3h type-like 1 | 4 | o | o | o | | | | | |
| zinc finger protein mym-type | 4 | o | | | | o | o | | |
| zinc finger protein zic 1 | 4 | | | | | | | o | o |
| zp3b protein | 4 | o | | | o | | | | |
| zpc domain containing protein 1 | 4 | | | | o | | | | |
| 10-formyltetrahydrofolate dehydrogenase | 3 | o | o | | | | | | |
| 2 -cyclic-nucleotide 3 -phosphodiesterase-like | 3 | | | | | | | | o |
| 26s protease regulatory subunit 6b | 3 | | o | | | | o | | o |
| 26s proteasome non-atpase regulatory subunit 7 | 3 | | o | | o | o | | | |
| 2-acylglycerol o-acyltransferase 1 | 3 | o | | o | | | | | |
| 2-acylglycerol o-acyltransferase 2 | 3 | | | | o | | | | |
| 2-c-methyl-d-erythritol 4-phosphate cytidylyltransferase-like protein | 3 | o | | | | o | | | |
| 2-oxoisovalerate dehydrogenase subunit mitochondrial-like | 3 | | | | o | | | | o |
| 2-peptidylprolyl isomerase a | 3 | | | o | o | | | | o |
| 3 -5 exoribonuclease csl4 homolog | 3 | o | | o | | | | o | |
| 3 -phosphoadenosine 5 -phosphosulfate synthase 2 | 3 | o | | o | | | | | o |
| 3-hydroxyisobutyrate dehydrogenase | 3 | o | o | | | | | | |
| 3-mercaptopyruvate sulfurtransferase | 3 | | o | | | | | | o |
| 40s ribosomal protein s15 | 3 | | | | o | | | o | o |
| 40s ribosomal protein s17 | 3 | | o | | o | | | | o |
| 40s ribosomal protein s30 | 3 | | | o | | o | | o | |
| 40s ribosomal protein s5 | 3 | | o | | o | | | | |
| 40s ribosomal protein s6 | 3 | | | | o | | | | o |

| | | | | | | | | | |
|---|---|---|---|---|---|---|---|---|---|
| 5 -nucleotidase domain containing 3 | 3 | o | | | | | | | |
| 55 kda erythrocyte membrane protein | 3 | o | o | o | | | | | |
| 5-aminolevulinate mitochondrial | 3 | | | | o | | | | o |
| 5-hydroxyisourate hydrolase | 3 | | o | o | | | | | |
| 60s ribosomal protein l13a | 3 | | | | o | | | | o |
| 60s ribosomal protein l18a | 3 | | o | | | | o | | o |
| 60s ribosomal protein l28 | 3 | | | | o | | | | o |
| 60s ribosomal protein l4-a | 3 | | | | o | | | | |
| 78 kda glucose-regulated protein precursor | 3 | o | | o | | | o | | |
| 7-dehydrocholesterol reductase | 3 | o | | | | | | | o |
| a chain structures of glycogen phosphorylase-inhibitor complexes and the implications for structure-based drug design | 3 | o | o | | | o | | | |
| a kinase anchor protein 1 | 3 | o | | | | | o | o | |
| abhydrolase domain containing 10 | 3 | o | o | o | | | | | |
| abhydrolase domain containing 14a | 3 | o | | o | | o | | | |
| abhydrolase domain containing 2 | 3 | o | | | | | o | | |
| abl interactor 1 | 3 | | | | | o | o | | |
| absent in melanoma 1 | 3 | | o | o | | o | | | |
| ac9 transposase | 3 | o | | | | | o | o | |
| acetylcholine receptor gamma subunit | 3 | o | | | | | | | |
| acid ceramidase-like | 3 | | | | o | | | | o |
| acidic coiled-coil containing protein 3 | 3 | o | o | | | | o | | |
| aconitase mitochondrial | 3 | o | | | | | o | | |
| actin binding 1c | 3 | | o | | | | o | o | |
| actin-binding lim protein 2-like | 3 | o | | | | | o | | |
| actinin alpha 2 | 3 | o | | | | | | o | |
| actin-related protein 2 3 complex subunit 2 | 3 | o | | | | | | | o |
| actin-related protein 3 | 3 | o | o | | | | | | |
| activating signal cointegrator 1 complex subunit 1 | 3 | | | o | | | | | o |
| acyl- synthetase family member mitochondrial precursor | 3 | o | o | o | | | | | |
| acyl- synthetase long-chain family member 3 | 3 | | | o | | | o | o | |
| acyl- thioesterase 11 | 3 | o | o | | | | | | |
| acylglycerol kinase | 3 | o | | | | o | | o | |
| adenosine deaminase-like protein | 3 | o | | o | | | o | | |
| adenosine monophosphate deaminase 1 (isoform m) | 3 | o | | | | | | | |
| adenosylhomocysteinase 3 | 3 | o | o | | | | | | |
| adenylate cyclase type 9 | 3 | o | | | | | o | o | |
| adenylate kinase 1 | 3 | o | | | | o | | o | |
| adenylate kinase 2 | 3 | | o | o | | o | | | |
| adiponectin receptor 1 | 3 | o | o | o | | | | | |
| adipose triglyceride lipase | 3 | | o | | | | | | |
| adp-ribosylation factor 4 | 3 | | | o | | o | | | o |
| adp-ribosylation factor gtpase activating protein 1 | 3 | o | | o | | | o | | |
| adp-ribosylation factor gtpase activating protein 3 | 3 | o | o | o | | | | | |
| adp-ribosylation factor interacting protein 1 | 3 | o | | o | | | | | |
| a-kinase anchor protein sphkap | 3 | | | | | | o | | o |
| akt-interacting protein | 3 | o | o | | | | | | |
| alanine racemase domain protein | 3 | o | | o | | o | | | |
| alanine-glyoxylate aminotransferase 2-like 2 | 3 | o | | o | | | | | |
| alcohol dehydrogenase 5 (class iii) chi polypeptide | 3 | o | o | | | | | | |
| aldh2 | 3 | | | o | o | | | | |
| aldo-keto reductase | 3 | o | | | | | | | o |
| alkaline ceramidase 2 | 3 | o | | | | | | | |

| | | | | | | | | | |
|---|---|---|---|---|---|---|---|---|---|
| allantoinase | 3 | | o | o | o | | | | |
| allograft inflammatory factor 1-like | 3 | o | | | o | | | | |
| all-trans-retinol -reductase-like | 3 | | | | o | | | | o |
| alpha-parvin | 3 | o | o | | | o | | | |
| alpha 2 ( congenital muscular dystrophy) | 3 | o | | | | | o | | |
| alpha-sialyltransferase st3gal iii | 3 | o | o | | | | | | |
| alpha-aminoadipic semialdehyde dehydrogenase | 3 | | o | | o | o | | | |
| alpha-fetoprotein enhancer-binding protein (at motif-binding factor) (at-binding transcription factor 1) | 3 | o | | | | | | o | |
| alpha-l- tissue | 3 | o | o | | | o | | | |
| aminoacyl trna synthase complex-interacting multifunctional protein 1 | 3 | o | o | o | | | | | |
| aminoacyl trna synthetase complex-interacting multifunctional protein 2 | 3 | o | | o | | | o | | |
| aminoadipate-semialdehyde synthase | 3 | o | o | o | | | | | |
| aminoglycoside phosphotransferase domain-containing protein 1 | 3 | o | | | o | | o | | |
| aminopeptidase n | 3 | o | | | | | o | | |
| aminophospholipid transporter class type member 1-like | 3 | | | o | | | | o | |
| amyloid beta precursor protein (cytoplasmic tail) binding protein 2 | 3 | o | | | | | | | |
| amyloid beta precursor-like protein 1 | 3 | o | | | | | o | o | |
| amyloid beta precursor-like protein 2 | 3 | | | | | o | o | o | |
| anamorsin | 3 | | o | o | | | | | o |
| anaphase promoting complex subunit 10 | 3 | o | o | | | o | | | |
| anaphase promoting complex subunit 11 | 3 | | | o | | o | | | |
| anaphase-promoting complex subunit 13 | 3 | o | o | | | o | | | |
| angio-associated migratory cell protein | 3 | o | o | | | | | | o |
| ankyrin repeat and btb domain containing 1 | 3 | o | | o | | o | | | |
| ankyrin repeat domain 27 (vps9 domain) | 3 | o | | | | | o | | |
| ankyrin repeat domain 39 | 3 | o | | | | | | | |
| annexin a6 | 3 | o | o | | | | | | |
| anthrax toxin receptor 2 | 3 | o | o | | | | | | |
| ap-1 complex subunit beta-1 | 3 | o | o | | | | | | |
| ap-1 complex subunit beta-1 isoform 2 | 3 | | | o | | | | o | o |
| ap2-associated protein kinase 1-like | 3 | | | | | | o | o | |
| apolipoprotein b-like protein | 3 | | o | | o | | | | |
| apolipoprotein d-like | 3 | | | | o | | | | |
| apolipoprotein f | 3 | | o | | | | | | |
| apoptosis-inducing factor mitochondrial | 3 | o | o | | | | | | |
| apurinic apyrimidinic endonuclease 2 | 3 | o | o | | | | | | |
| arginine- mutated in early stage tumors | 3 | o | o | o | | | | | |
| arginine serine-rich coiled-coil protein 2 | 3 | o | o | | | | | | o |
| arginine-rich mutated in early stage tumors | 3 | o | | o | | | | | |
| argininosuccinate lyase | 3 | | | | o | o | | | o |
| argininosuccinate synthase | 3 | o | o | | | | | o | |
| argininosuccinate synthetase 1 | 3 | | | | | o | | | o |
| arginyl trna anticodon binding | 3 | o | | | | | | | |
| arylamine n- pineal gland isozyme nat-10 | 3 | o | | o | | | | | |
| asb12 protein | 3 | o | | o | | | | | o |
| aspartate beta-hydroxylase | 3 | o | o | | | | | | |
| astacin-like metalloprotease | 3 | | | | o | | | | |
| astrotactin 1 | 3 | o | | | | | o | | |
| ataxin-1 ubiquitin-like interacting protein | 3 | o | o | | | | | | o |
| atp synthase mitochondrial f1 complex assembly factor 2 | 3 | o | o | | | o | | | |
| atp synthase subunit s-like | 3 | o | | o | o | | | | |

| | | | | | | | | | |
|---|---|---|---|---|---|---|---|---|---|
| atp-binding cassette sub-family a member 1-like | 3 | | o | | | | o | | |
| atp-binding cassette sub-family g member 8 | 3 | | o | o | | | | | |
| atp-binding sub-family f member 1 | 3 | o | o | | | | o | | |
| atp-binding sub-family f member 2 | 3 | o | o | | | | o | | |
| atp-dependent rna helicase ddx19b | 3 | | | o | o | | | | |
| atp-dependent rna helicase ddx51 | 3 | | | | | | o | | o |
| aurora kinase a-interacting protein | 3 | o | o | | o | | | | |
| autophagy related 16 like 2-like | 3 | o | | | | | | | |
| autophagy-related protein 2 homolog a | 3 | | | | | | o | | |
| basigin (ok blood group) | 3 | | | o | | | o | o | |
| bat2 domain containing 1 | 3 | o | | | | | o | | |
| bax inhibitor 1 | 3 | | | | o | | | | o |
| bbsome-interacting protein of 10 kda-like | 3 | o | | o | | o | | | |
| bcl2 adenovirus e1b 19kda interacting protein 2 | 3 | o | o | o | | | | | |
| bcl2-like 14 (apoptosis facilitator) | 3 | o | o | o | | | | | |
| beclin 1 | 3 | o | | o | | o | | | |
| beta-ala-his dipeptidase | 3 | o | | | | | | o | o |
| beta-catenin-like protein 1 | 3 | o | o | | | | | | o |
| beta-glucuronidase precursor | 3 | o | o | o | | | | | |
| betaine-homocysteine methyltransferase | 3 | | o | o | | | o | | |
| beta-site app-cleaving enzyme 1 | 3 | o | | | | | o | | |
| beta-thymosin domain repeat protein csp24kda_v1 | 3 | | | | o | | | | |
| bh3 interacting domain death agonist | 3 | o | | | o | | | o | |
| biogenesis of lysosome-related organelles complex-1 subunit 3 | 3 | o | | o | | o | | | |
| bloom syndrome helicase | 3 | o | o | | | | | o | |
| br serine threonine-protein kinase | 3 | | | | o | | | | |
| brain-specific angiogenesis inhibitor 3 | 3 | | | | | o | o | o | |
| branched chain keto acid dehydrogenase alpha polypeptide | 3 | o | o | | | | | | |
| branched chain ketoacid dehydrogenase kinase | 3 | o | o | | | | | | |
| brevican | 3 | o | | | | | o | o | |
| bromodomain adjacent to zinc finger 1b | 3 | o | | | | | | | |
| bromodomain containing 9 | 3 | o | o | | | | | | |
| bromodomain-containing protein 2 precursor | 3 | | | | | o | o | | |
| btb poz domain-containing protein kctd6-like | 3 | | | | o | | | | |
| btg3 associated nuclear protein | 3 | o | o | | | | | | |
| btla protein | 3 | o | | | | | | | |
| c21orf59 homolog ( sapiens) like | 3 | o | | o | | | | | |
| c2orf76 protein | 3 | o | o | | | o | | | |
| c5orf44 protein | 3 | o | | o | | o | | | |
| c6orf64 homolog | 3 | o | | o | | o | | | |
| ca2+-dependent secretion activator-like | 3 | | | | | o | | o | |
| cadherin-related neuronal receptor c02 | 3 | o | | | | | o | o | |
| calcineurin b | 3 | | | | | o | | o | |
| calcium binding protein 39-like | 3 | o | | | | | o | o | |
| calcium release-activated calcium channel protein 1 | 3 | o | | | | | | o | |
| calcium voltage- alpha 2 delta subunit 1 | 3 | o | | | | | | | |
| calcium voltage- p q alpha 1a isoform cra_a | 3 | | | | | | o | o | |
| calcium-binding mitochondrial carrier protein s -1 | 3 | o | | | | | | | o |
| calcium-binding protein 1-like | 3 | | | | o | | | | o |
| calcyclin binding protein | 3 | o | | o | | | | o | |
| calmodulin binding transcription activator 1 | 3 | | | | | | o | o | |
| camp-dependent protein kinase catalytic subunit alpha | 3 | o | o | | | | | | o |

| | | | | | | | | | |
|---|---|---|---|---|---|---|---|---|---|
| camp-dependent protein kinase type i-alpha regulatory subunit | 3 | o | o | | | | | | |
| cancer associated nucleoprotein | 3 | o | | | | | | | |
| cannabinoid receptor 1 | 3 | o | | | | | o | | |
| caprin2 protein | 3 | | | | o | | | | |
| cardiac troponin i | 3 | o | | | | | | | |
| cardiolipin synthase 1 | 3 | o | o | | | | | | |
| carnitine palmitoyltransferase ii | 3 | o | o | o | | | | | |
| cartilage intermediate layer nucleotide pyrophosphohydrolase | 3 | o | | | | o | | | |
| cas1 domain containing 1 | 3 | | | o | | | o | o | |
| casein kinase alpha prime polypeptide | 3 | o | o | | | | | o | |
| caspase b | 3 | o | o | | o | | | | |
| caspase-3 precursor | 3 | o | o | | | | | | |
| catalase | 3 | o | o | | o | | | | |
| catenin alpha-1 | 3 | o | | | | | | | |
| cathepsin k | 3 | o | | | | o | | o | |
| cathepsin s precursor | 3 | | | | o | | | | |
| cathepsin z | 3 | o | | o | | | | o | |
| cbp p300-interacting transactivator 2 | 3 | | | | | o | o | | o |
| ccr4-not transcription complex subunit 1-like | 3 | | o | | | | o | | |
| ccr4-not transcription subunit 6 | 3 | o | | | | | o | | |
| cdc28 protein kinase regulatory subunit 1b | 3 | o | o | | | | | | |
| cdc5 cell division cycle 5-like ( pombe) | 3 | o | o | | | | | | |
| cdc-like kinase 2 | 3 | o | | | | | | | |
| cdgsh iron sulfur domain 3 | 3 | o | o | o | | | | | |
| cell cycle control protein 50a | 3 | | o | | | | o | | |
| cell cycle regulator mat89bb homolog | 3 | o | o | | | | | | |
| cell division control protein 2 homolog | 3 | o | o | | | | | | o |
| cell division cycle 42 | 3 | o | | o | | | | o | |
| cell division cycle paf1 rna polymerase ii complex homolog ( cerevisiae) | 3 | o | o | o | | | | | |
| cell division cycle protein 16 homolog | 3 | o | | | o | | | | o |
| cell surface protein precursor | 3 | | | | o | | | | o |
| cellular retinoic acid-binding protein 1 | 3 | o | | | | o | | o | |
| cerebellin 2 | 3 | | | | | o | o | o | |
| cg32095 cg32095-pa | 3 | o | | | | o | | o | |
| chaperonin containing subunit 8 | 3 | o | o | | | | | | o |
| charged multivesicular body protein 4c | 3 | | | | o | o | | | |
| chemokine ccl-c11b | 3 | o | | | | o | o | | |
| choline kinase alpha | 3 | | o | o | | | | | o |
| cholinergic beta 1 | 3 | o | | | | | | | |
| chondroitin sulfate proteoglycan 4 | 3 | o | | | | | | | |
| chromatin modification-related protein meaf6 | 3 | o | | | | o | | | o |
| chromatin modifying protein 4b | 3 | o | | o | | o | | | |
| chromosome 1 open reading frame 128 | 3 | o | | | | o | | o | |
| chromosome 1 open reading frame 43 | 3 | o | | | | | | o | |
| chromosome 16 open reading frame isoform cra_a | 3 | o | | o | | | | | |
| chromosome 19 open reading frame 43 | 3 | o | | o | | o | | | |
| chromosome 3 open reading frame isoform cra_b | 3 | | | | | | | o | o |
| chromosome 6 open reading frame 57 | 3 | o | o | | | o | | | |
| chromosome 7 open reading frame 30 | 3 | o | | o | | | | o | |
| chromosome 7 open reading frame 38 | 3 | o | | | | | | o | |
| chymotrypsinogen 2 | 3 | | o | | | | | | |
| citrate synthase | 3 | o | o | | | | | | |
| claudin 4 | 3 | | | | o | | o | | |
| cleavage and polyadenylation specificity factor subunit 4-like isoform 1 | 3 | | | | o | | | | |

| | | | | | | | | | |
|---|---|---|---|---|---|---|---|---|---|
| cleft lip and palate transmembrane protein 1 homolog | 3 | | | | o | | | | o |
| cleft lip and palate transmembrane protein 1-like protein | 3 | o | o | o | | | | | |
| coagulation factor x precursor | 3 | | | | | | | | o |
| coagulation factor xi precursor | 3 | | o | | o | | | | |
| coatomer subunit delta | 3 | o | | o | | o | | | |
| coenzyme a synthase | 3 | o | o | o | | | | | |
| cofilin 2 | 3 | o | o | | | | | | |
| coiled-coil domain containing 106 | 3 | o | | | | | | | |
| coiled-coil domain containing 19 | 3 | | o | | | | | o | |
| coiled-coil domain containing 23 | 3 | o | | o | | | | o | |
| coiled-coil domain containing 28a | 3 | o | | o | | o | | | |
| coiled-coil domain containing 32 | 3 | o | o | | | | | o | |
| coiled-coil domain containing 47 | 3 | o | o | o | | | | | |
| coiled-coil domain containing 53 | 3 | o | o | | | | | | |
| coiled-coil domain containing 94 | 3 | o | o | | | | | | |
| coiled-coil domain-containing protein 12 | 3 | o | o | | | o | | | |
| coiled-coil domain-containing protein 152-partial | 3 | | | | o | | | | o |
| coiled-coil domain-containing protein 22 | 3 | o | o | | | o | | | |
| coiled-coil domain-containing protein 93 | 3 | o | o | | | o | | | |
| coiled-coil-helix-coiled-coil-helix domain containing 1 | 3 | | o | o | | o | | | |
| coiled-coil-helix-coiled-coil-helix domain-containing protein 7 | 3 | o | | o | | | | o | |
| coiled-coil-helix-coiled-coil-helix domain-containing protein 8 | 3 | o | | | o | o | | | |
| coiled-coil-helix-coiled-coil-helix domain-containing protein mitochondrial | 3 | | | | o | | | o | o |
| collagen alpha-6 chain-like | 3 | o | | | | | | | |
| collagenase 3 precursor | 3 | o | o | o | | | | | |
| comm domain-containing protein 3 | 3 | o | | o | | | | o | |
| comm domain-containing protein 8 | 3 | | | | o | | | | o |
| complement component 4a (rodgers blood group) | 3 | o | | | o | | | | |
| complement component beta polypeptide | 3 | | o | o | | | | | |
| complement component c7 | 3 | o | o | | | o | | | |
| complement factor h-related 2 | 3 | | o | | | | | | |
| component of oligomeric golgi complex 6 | 3 | o | | o | | o | | | |
| condensin complex subunit 1 | 3 | | | | o | o | | | |
| connector enhancer of kinase suppressor of ras 1 | 3 | o | o | | | | | | |
| cop9 signalosome complex subunit 1 | 3 | o | o | o | | | | | |
| cop9 signalosome complex subunit 4 | 3 | o | | o | | | | | |
| cop9 signalosome complex subunit 7a | 3 | | o | | | | o | | |
| copine family member ix | 3 | | | | | | o | | |
| copine i | 3 | o | | | | | o | | |
| cornichon homolog | 3 | | o | | | | o | o | |
| cr054 protein | 3 | o | | | | | o | | |
| ctd small phosphatase-like protein | 3 | o | | | | | | | |
| c-type lysozyme | 3 | | | | o | | | | |
| c-type superfamily member 14 | 3 | | o | o | | | | | |
| cub and sushi domain-containing protein 3 isoform 3 | 3 | o | | | | | o | | |
| cub and sushi multiple domains 3 | 3 | | | | | | o | | |
| cugbp elav-like family member 4-like | 3 | | | | | | o | o | |
| cullin 1 | 3 | o | o | | | | | | |
| cullin 1a | 3 | | o | o | | | | o | |
| cullin 3 isoform 1 | 3 | o | | o | | | | o | |

| | | | | | | | | | |
|---|---|---|---|---|---|---|---|---|---|
| c-x-c motif chemokine 14 precursor | 3 | | | | | | | | o |
| cyclic amp-dependent transcription factor atf-5 | 3 | o | | | | | | o | |
| cyclin-dependent kinase 10 | 3 | o | | | | | | o | |
| cyclin-dependent kinase 7 | 3 | o | o | o | | | | | |
| cyclin-h | 3 | | | o | | o | | o | |
| cysteine dioxygenase | 3 | | o | | o | | | | |
| cysteine-rich hydrophobic domain 2 | 3 | o | o | | | | | | |
| cysteinyl-trna synthetase | 3 | o | | o | | | | | |
| cytochrome b reductase 1 | 3 | o | | | | | o | o | |
| cytochrome c oxidase subunit mitochondrial-like | 3 | | | | o | | | | o |
| cytochrome c oxidase subunit va | 3 | | | | o | | | | |
| cytochrome c oxidase subunit viia polypeptide 2 like | 3 | o | | o | | | | o | |
| cytochrome c oxidase subunit viia-related mitochondrial precursor | 3 | o | o | | | o | | | |
| cytochrome p450 27c1-like | 3 | | | | o | | | | o |
| cytoglobin | 3 | | | o | o | | | o | |
| cytohesin 1 | 3 | o | o | | | | | | |
| cytoplasmic aconitate hydratase | 3 | o | o | | | | | | |
| cytoplasmic dynein 1 intermediate chain 2 | 3 | o | o | | | | | | |
| cytoplasmic polyadenylation element binding protein 4 | 3 | o | | | | | o | | |
| cytosol aminopeptidase | 3 | | o | o | | o | | | |
| cytosolic carboxypeptidase 4 | 3 | o | | | | | | | |
| cytosolic fe-s cluster assembly factor narfl | 3 | o | o | | | | | | o |
| d-amino-acid oxidase | 3 | o | o | o | | | | | |
| daz associated protein 2 | 3 | | o | | | | o | o | |
| ddhd domain containing 2 | 3 | o | | | | | o | | |
| ddx5 protein | 3 | o | o | | | | o | | |
| dead (asp-glu-ala-asp) box polypeptide 18 | 3 | o | o | o | | | | | |
| dead (asp-glu-ala-asp) box polypeptide 23 | 3 | | o | o | | o | | | |
| dead (asp-glu-ala-asp) box polypeptide 49 | 3 | o | | | | o | o | | |
| dead-box protein | 3 | o | | | | | | o | |
| deah (asp-glu-ala-his) box polypeptide 37 | 3 | o | | o | | | | | |
| deah (asp-glu-ala-his) box polypeptide 8 | 3 | o | o | | | | | | |
| dehydrogenase reductase (sdr family) member 7c | 3 | o | | | | | | | |
| dehydrogenase reductase sdr family member 11 precursor | 3 | o | o | | | | | o | |
| deleted in bladder cancer 1 | 3 | | | | | o | | o | |
| deleted in liver cancer 1 | 3 | o | | | | | o | | |
| delta-6 fatty acyl desaturase | 3 | | o | | | o | | | o |
| deltex 3-like | 3 | o | | o | | | | | |
| denn madd domain containing 2d | 3 | o | o | | | | | | |
| dentin sialophosphoprotein precursor | 3 | o | | | | o | o | | |
| deoxynucleotidyltransferase terminal-interacting protein 2 | 3 | | | | o | | | | |
| deoxyribonuclease ii beta | 3 | o | | o | | | | | |
| det1- and ddb1-associated protein 1 | 3 | o | o | o | | | | | |
| developmentally regulated gtp binding protein 2 | 3 | o | | o | | | | | |
| developmentally-regulated gtp-binding protein 1 | 3 | | | | o | | | | |
| differentially expressed in fdcp 8 homolog | 3 | o | | | | | | | o |
| dihydropyrimidinase-related protein 3 | 3 | o | | | | | | | |
| dihydropyrimidine dehydrogenase | 3 | | o | o | | | | | |
| di-n-acetylchitobiase precursor | 3 | o | | | o | | | | |
| diphthamide biosynthesis protein 2 | 3 | o | | | | | | | |

| | | | | | | | | | |
|---|---|---|---|---|---|---|---|---|---|
| disabled homolog 2 interacting protein | 3 | | | | | | o | | |
| discoidin domain receptor member 2 | 3 | o | | | | | | | |
| disintegrin metalloproteinase | 3 | o | | o | | | | | |
| dna ligase 3 | 3 | | o | | | | | | o |
| dna polymerase beta | 3 | o | o | | | | | o | |
| dna polymerase delta subunit 3 | 3 | o | | | o | o | | | |
| dna replication licensing factor mcm3 | 3 | o | | | o | | | | |
| dna-binding protein | 3 | | o | | o | | | | |
| dna-damage inducible protein 2 | 3 | o | | o | | | o | | |
| dna-damage-inducible transcript 3 | 3 | o | o | | | | | | |
| dna-damage-inducible transcript 4-like | 3 | | | | o | | | | o |
| dna-directed rna polymerase iii subunit rpc10 | 3 | o | | o | o | | | | |
| dna-directed rna polymerase iii subunit rpc4 | 3 | o | o | | | | | | |
| dna-directed rna polymerase subunit rpabc1 | 3 | o | | o | | o | | | |
| dna-directed rna polymerases and iii subunit rpabc3 | 3 | o | | o | | o | | | |
| dna-directed rna polymerases i and iii subunit rpac2 | 3 | o | o | | o | | | | |
| dnase domain containing 3 | 3 | o | | | | o | | | |
| dolichyl-diphosphooligosaccharide--protein glycosyltransferase 48 kda subunit precursor | 3 | o | | | o | o | | | |
| dolichyl-diphosphooligosaccharide--protein glycosyltransferase subunit 1 precursor | 3 | | | | o | | | | o |
| dolichyl-diphosphooligosaccharide--protein glycosyltransferase subunit 4 | 3 | o | | o | | o | | | |
| domain containing 2 | 3 | o | | | o | | | | |
| doublecortin domain-containing protein 2 | 3 | | | | o | | | | |
| down syndrome critical region protein 3 | 3 | o | | o | | | | o | |
| drebrin-like protein | 3 | o | o | o | | | | | |
| dual specificity phosphatase 26 | 3 | o | | | | | o | | |
| dual specificity phosphatase and pro isomerase domain containing 1 | 3 | o | | | | | | | |
| dual specificity protein phosphatase cdc14a | 3 | o | | | | o | | | |
| dual-specificity tyrosine- -phosphorylation regulated kinase 1a | 3 | o | | | | | o | o | |
| dynactin subunit 1 | 3 | o | o | | | | | | |
| dynein heavy chain | 3 | o | | | o | | o | | |
| dynein light chain roadblock-type 1 | 3 | | o | o | | | | o | |
| dystonin isoform 2 | 3 | | o | o | | | o | | |
| dystrobrevin binding protein 1 | 3 | o | | | | | | o | |
| e2f transcription factor 3 | 3 | o | | | | | | | |
| e3 ubiquitin isg15 ligase trim25-like | 3 | o | o | o | | | | | |
| e3 ubiquitin-protein ligase listerin | 3 | | | | | | | | o |
| e3 ubiquitin-protein ligase march2 | 3 | o | o | | | | | | |
| ectonucleotide pyrophosphatase phosphodiesterase family member 3 | 3 | | o | | o | | | | |
| eef1a2 binding | 3 | o | | | | | | | |
| ef-hand calcium binding protein | 3 | | | o | | o | o | | |
| ef-hand domain member a1 | 3 | o | o | | | o | | | |
| egl nine homolog 1 | 3 | o | | o | | o | | | |
| eh domain-binding protein 1-like protein 1 | 3 | o | | | | | | | |
| eh domain-binding protein 1-like protein 1-like | 3 | o | | | | | | | |
| eh-domain containing 2 | 3 | o | o | | | | | | |
| elastase 1 precursor | 3 | | o | | | | | | |
| elastase 2 like | 3 | o | o | | | | o | | |
| elastin a | 3 | o | | | | | | | |
| elav-like protein 3-like | 3 | | | | | | o | o | |
| electrogenic sodium bicarbonate cotransporter 1 | 3 | | | | | o | | o | o |

| | | | | | | | | | |
|---|---|---|---|---|---|---|---|---|---|
| electron-transfer- alpha polypeptide | 3 | | | | o | | | | o |
| elegans protein partially confirmed by transcript evidence | 3 | o | | | | | | | |
| elongation factor 1-alpha 1-like isoform 3 | 3 | | | | | o | o | o | |
| elongation factor tu gtp binding domain containing 2 | 3 | o | | o | | o | | | |
| elongation rna polymerase 2 | 3 | o | | o | | | | | |
| ena vasp-like protein | 3 | o | | | | | o | | o |
| endothelin b receptor-like protein 2 precursor | 3 | | | | | | o | o | |
| ensangp00000010363 | 3 | | | | | o | o | o | |
| eorge syndrome critical region gene 2 | 3 | o | | o | | | o | | |
| eorge syndrome critical region gene 8 | 3 | | o | | | | | | o |
| epsilon-sarcoglycan isoform 3 | 3 | o | | | | o | o | | |
| er lipid raft associated 1 | 3 | o | o | | | | | | |
| erythrocyte band 7 integral membrane protein | 3 | o | o | o | | | | | |
| estrogen receptor binding site 9 | 3 | o | | | o | | | | o |
| eukaryotic initiation factor 4a-ii | 3 | | | | o | o | | | |
| eukaryotic peptide chain release factor subunit 1 | 3 | o | | o | | | | | |
| eukaryotic translation elongation factor 1 alpha oocyte form | 3 | | | | o | | | | |
| eukaryotic translation initiation factor 2 alpha kinase 4 | 3 | o | o | | | | | | |
| eukaryotic translation initiation factor 2-alpha kinase 3 | 3 | o | | | | | | | |
| eukaryotic translation initiation factor 5a-1 | 3 | o | o | | | | | o | |
| eukaryotic translation initiation factor subunit 1 alpha | 3 | o | | o | | | | | |
| exocyst complex component 1 isoform 1 | 3 | o | o | | | | | o | |
| exosome component 8 | 3 | o | | o | o | | | | |
| exportin 1 (crm1 yeast) | 3 | o | | | | | | | |
| eya1 protein | 3 | o | | | | | | | |
| eyes absent homolog 1 | 3 | o | | | | | | | |
| fat tumor suppressor homolog 1 | 3 | | o | | | | o | | |
| f-box protein 2 | 3 | o | | | | o | | | o |
| fgfr1 oncogene partner 2 | 3 | o | o | | | | | o | |
| fibroblast growth factor receptor 1 iiic vt-isoform | 3 | o | o | o | | | | | |
| fibroblast growth factor receptor 2 | 3 | | o | | | | o | | |
| fibronectin type iii domain containing 1 | 3 | o | | | | | | | |
| fin bud initiation factor homolog | 3 | o | | o | | o | | | |
| fk506 binding protein 12-rapamycin associated protein 1 | 3 | o | | | | o | o | | |
| fk506 binding protein 22 kda | 3 | o | | o | | | | | |
| fk506-binding protein | 3 | | o | | o | | | | o |
| fkbp9 protein | 3 | o | | | o | | | | |
| flj00022 protein | 3 | | o | o | | | o | | |
| fn1 protein | 3 | | | | o | | o | | |
| foie gras | 3 | o | | o | | | | | |
| frataxin | 3 | o | | | | o | | o | |
| friend leukemia integration 1 transcription factor | 3 | o | | | | | | | o |
| fumarylacetoacetate hydrolase | 3 | | o | | | | | | |
| fumarylacetoacetate hydrolase domain-containing protein 1 | 3 | o | o | o | | | | | |
| g protein pathway suppressor 2 | 3 | | o | | | | | | |
| g protein-coupled receptor 137b | 3 | o | | | | | o | | |
| g protein-coupled receptor 146 | 3 | o | | | | | o | o | |
| g protein-coupled receptor 155 | 3 | | o | | | | | | |
| g protein-coupled receptor 89a | 3 | o | | o | | o | | | |

| | | | | | | | | | |
|---|---|---|---|---|---|---|---|---|---|
| gag-like protein | 3 | o | | o | | | o | | |
| galactoside- 3 binding protein | 3 | | o | | | o | | | |
| galactoside- soluble isoform cra_a | 3 | | | | | | | | o |
| galectin-9 | 3 | o | | o | | | | | o |
| gametogenetin binding protein 2 | 3 | o | o | | | | | | |
| gamma 1 | 3 | o | | | | | | o | |
| gamma-aminobutyric acid receptor subunit beta-3-like | 3 | | | | | | o | | |
| gamma-soluble nsf attachment protein | 3 | | | | | | | | o |
| ganab protein | 3 | | o | | | | o | o | |
| gap junction beta-1 protein | 3 | | | | | | o | | o |
| gats-like protein 3 | 3 | o | | | | | | | o |
| gc-rich sequence dna-binding factor 1 | 3 | | | | o | | | | o |
| gdp-mannose -dehydratase | 3 | o | | | | o | | | |
| gdp-mannose pyrophosphorylase isoform cra_a | 3 | | o | o | | o | | | |
| glia maturation factor beta | 3 | o | | o | | | | o | |
| glia-derived nexin precursor | 3 | o | | | o | | | | o |
| glial fibrillary acidic protein | 3 | o | | | | | o | o | |
| glioma tumor suppressor candidate region gene 2 protein | 3 | o | o | | | o | | | |
| gliomedin | 3 | o | | | | | | | o |
| glucocorticoid induced transcript 1 | 3 | | | o | | o | | | |
| glucokinase | 3 | | o | o | | | | | |
| glucosamine-6-phosphate deaminase 1 | 3 | o | | | | o | | o | |
| glucosamine-phosphate n-acetyltransferase 1 | 3 | o | o | o | | | | | |
| glucose-6-phosphatase | 3 | | o | | o | | | | |
| glutamate ampa 3 | 3 | | | | | | | o | |
| glutamate ampa 4 | 3 | | | | | | o | o | |
| glutamate delta 1 | 3 | | | | | | o | o | |
| glutamate n-methyl d-aspartate-associated protein 1 (glutamate binding) | 3 | o | | | | | | o | |
| glutamate receptor u1 precursor | 3 | | | | | o | o | o | |
| glutamate-rich wd repeat containing 1 | 3 | o | | o | o | | | | |
| glutamyl-prolyl-trna synthetase | 3 | o | | o | | | | | |
| glutaredoxin 2 | 3 | o | | | | | o | o | |
| glutaredoxin-related protein 5 | 3 | o | o | o | | | | | |
| glycogen synthase 2 | 3 | | o | | | | | | |
| glycolipid transfer protein | 3 | o | o | o | | | | | |
| glycylpeptide n-tetradecanoyltransferase 1 | 3 | o | | | o | | o | | |
| gmp synthase | 3 | o | | | | | | o | |
| gnl3 protein | 3 | | o | | o | | | | |
| golgi apparatus protein 1 | 3 | | | o | | | o | o | |
| golgi snap receptor complex member 2 | 3 | o | | o | | o | | | |
| gpi mannosyltransferase 3 | 3 | o | o | | o | | | | |
| gpn-loop gtpase 1 | 3 | o | | | | | | | o |
| gpn-loop gtpase 2 | 3 | o | | o | | o | | | |
| grancalcin | 3 | o | | | | | | | o |
| grb2-associated binding protein 1 | 3 | o | | | | | | o | |
| g-rich rna sequence binding factor 1 | 3 | o | o | | | | | o | |
| grnb protein | 3 | o | | o | | | | o | |
| group xiia secretory phospholipase a2 precursor | 3 | o | | o | | | o | | |
| growth arrest and dna-damage- alpha | 3 | o | | o | | | | | |
| growth hormone-regulated tbc protein 1-like | 3 | | | | o | | | | |
| growth inhibition and differentiation related protein 86 | 3 | o | | o | | | | o | |
| gtp-binding protein 8 | 3 | o | | o | | | | o | |
| gtp-binding protein guf1 homolog | 3 | o | o | | | | | | |

| | | | | | | | | | |
|---|---|---|---|---|---|---|---|---|---|
| guanine nucleotide binding protein (g protein) alpha activating activity olfactory type | 3 | o | | | | | o | o | |
| guanine nucleotide-binding 3-like | 3 | | | | o | | | | o |
| guanine nucleotide-binding protein g g g subunit gamma-2-like | 3 | | | | o | | | | o |
| guanine nucleotide-binding protein g g g subunit gamma-3-like | 3 | | | | | | | | o |
| guanine nucleotide-binding protein g subunit alpha-1-like | 3 | | o | | | | | | |
| guanine nucleotide-binding protein g subunit alpha-like | 3 | | | | o | | | | o |
| guanosine monophosphate reductase 2 | 3 | | | o | | o | o | | |
| h+ lysosomal v0 subunit a2 | 3 | o | | | | | | o | |
| h+ lysosomal v0 subunit d1 | 3 | o | o | | | | | | |
| h+ lysosomal v1 subunit b2 | 3 | o | o | | | | o | | |
| h+ lysosomal v1 subunit c1 | 3 | o | o | | | | | | |
| haptoglobin precursor | 3 | | | | o | | | | |
| haus augmin-like complex subunit 1-like | 3 | | | | o | | | | |
| haus augmin-like complex subunit 6-like | 3 | | | | o | | | | |
| heat shock 70 kda protein 14 | 3 | | | | o | o | | | |
| heat shock cognate 70 kda protein | 3 | | | | o | | o | | |
| heat shock protein 47 | 3 | o | | | | o | | | |
| heat shock protein beta-1 | 3 | | o | | | o | | | o |
| heavy chain non-muscle | 3 | o | o | | | | | | |
| helicase mov-10 | 3 | | | o | o | | | o | |
| helicase with zinc finger | 3 | | | o | | | o | o | |
| helicase with zinc finger domain | 3 | o | | o | | | | | |
| heme oxygenase 1 | 3 | o | o | o | | | | | |
| hemoglobin beta chain | 3 | o | | | | o | o | | |
| hemoglobin subunit beta-2 | 3 | o | o | | | o | | | |
| hemolytic toxin | 3 | o | | | | | | | |
| heparan sulfate glucosamine 3-o-sulfotransferase 1-like | 3 | | | | | | o | | o |
| heparan-alpha-glucosaminide n-acetyltransferase-like | 3 | o | o | | | | | | |
| heparin cofactor ii | 3 | | o | o | o | | | | |
| hepatic leukemia factor- partial | 3 | o | | o | | o | | | |
| hepatocyte growth factor-like protein | 3 | | o | o | | | | | |
| hepatocyte nuclear factor 1-alpha | 3 | | o | o | | | | | |
| hepatoma-derived growth factor-related protein 2 | 3 | o | o | | | | | | |
| hephaestin-like 1 | 3 | o | | | | | o | | |
| hermansky-pudlak syndrome 5 | 3 | | o | | o | | | | |
| heterogeneous nuclear ribonucleoprotein a b | 3 | | | | | | | | o |
| heterogeneous nuclear ribonucleoprotein m | 3 | o | o | | | | | | o |
| heterogeneous nuclear ribonucleoprotein q | 3 | o | | | | o | o | | |
| hexosaminidase a | 3 | o | | o | | | | | |
| high choriolytic enzyme 1 precursor | 3 | o | o | | | | | | |
| high-mobility group 20b | 3 | o | | o | | o | | | |
| histocompatibility 13 | 3 | | o | o | o | | | | |
| histone h2a-like | 3 | | o | | o | | | | o |
| histone-lysine n-methyltransferase ezh1-like | 3 | o | | | | | o | o | |
| homeobox protein 9aa | 3 | o | | | | | | | |
| homeodomain interacting protein kinase 1 | 3 | | | | | | o | | |
| homolog 3 ( coli) | 3 | o | | | | | | | |
| hsp70-binding protein 1 | 3 | o | | | | | | | o |
| hspc020 homolog | 3 | o | | o | | | | o | |
| huntingtin | 3 | o | | | | | o | | |
| hyaluronan and proteoglycan link protein 1 precursor | 3 | o | | | | | | | o |

| | | | | | | | | | |
|---|---|---|---|---|---|---|---|---|---|
| hydroxyacylglutathione hydrolase | 3 | o | o | o | | | | | |
| hydroxyacylglutathione mitochondrial-like | 3 | | | | o | | | | |
| hydroxy-delta-5-steroid 3 beta- and steroid delta-isomerase 7 | 3 | o | o | o | | | | | |
| hydroxysteroid (17-beta) dehydrogenase 4 | 3 | | o | o | | | | | |
| hydroxysteroid dehydrogenase-like protein 2 | 3 | o | o | | | | | | |
| hypoxanthine phosphoribosyltransferase 1 | 3 | o | | | | o | | | |
| immediate early response 2 | 3 | o | o | | o | | | | |
| immediate early response 3-interacting protein 1-like | 3 | | | | o | | | | o |
| immune- lectin-like receptor 3 | 3 | o | | | | | | | o |
| immunoglobulin member 3 | 3 | | | o | | | o | o | |
| immunoglobulin member 9b | 3 | | | o | | | o | | |
| importin 4 | 3 | o | | o | | | o | | |
| influenza virus ns1a binding protein | 3 | o | | | | | o | | |
| inhibitor of dna binding dominant negative helix-loop-helix protein | 3 | | o | | | o | o | | |
| inosine triphosphate pyrophosphatase | 3 | o | | | | o | | o | |
| inositol -pentakisphosphate 2-kinase | 3 | o | | o | | | o | | |
| inositol polyphosphate 5-phosphatase ocrl-1 | 3 | o | | | | | | o | |
| inositol-3-phosphate synthase 1 | 3 | o | | | | | | | o |
| inositol-3-phosphate synthase 1-b-like | 3 | | | | | | | | o |
| insulin-induced gene 2 protein | 3 | o | | | | | | | o |
| insulin-like growth factor binding protein 1 | 3 | | o | o | | | | | |
| integral membrane protein 2a | 3 | | | o | | | | | o |
| integrator complex subunit 8 | 3 | o | | | | o | | | |
| inter-alpha inhibitor h4 | 3 | | o | | | | | | |
| interferon induced with helicase c domain 1 | 3 | | | o | | | | | |
| interferon regulatory factor 6 | 3 | | o | o | | | | | |
| interferon-induced helicase c domain-containing protein 1 | 3 | o | | | o | | | | |
| interferon-induced protein 35 | 3 | o | o | o | | | | | |
| interleukin 8 | 3 | o | | o | | | | | |
| intracellular hyaluronan-binding protein 4 | 3 | | | | o | | | | |
| iron zinc purple acid phosphatase-like | 3 | o | | o | | | | o | |
| iron-responsive element binding protein 2 | 3 | o | o | o | | | | | |
| iron-responsive element-binding protein 2 | 3 | | | | o | | | | |
| isoleucyl-trna synthetase | 3 | o | | o | o | | | | |
| isovaleryl coenzyme a dehydrogenase | 3 | o | o | | | o | | | |
| janus kinase 2 | 3 | o | | | | | o | | |
| jumonji domain containing 2a | 3 | o | | | | | o | | |
| jumonji domain containing 4 | 3 | o | | o | | | | o | |
| junction plakoglobin | 3 | o | o | o | | | | | |
| junctional adhesion molecule 3 | 3 | o | | | | | | | |
| kaptin (actin binding protein) | 3 | o | o | | | | | | |
| kdel (lys-asp-glu-leu) endoplasmic reticulum protein retention receptor 3 | 3 | o | | | | | | | o |
| kiaa0232 gene product | 3 | o | | | | | | | |
| kiaa0907 protein | 3 | o | o | | | | o | | |
| kiaa1872 protein | 3 | | | | | o | | o | |
| kinase d-interacting substrate of 220 kda | 3 | | | | o | | o | o | |
| kinesin family member 3a | 3 | o | | | | | | o | |
| kininogen 1 | 3 | | o | o | | | | | |
| kruppel-like factor 12 | 3 | | o | | | | o | o | |
| ks5 protein | 3 | | o | o | | | | | |
| kynureninase (l-kynurenine hydrolase) | 3 | | o | o | | | | | |
| kynurenine 3-monooxygenase | 3 | o | o | o | | | | | |
| kynurenine--oxoglutarate transaminase 3 | 3 | o | o | | o | | | | |
| kynurenine--oxoglutarate transaminase 3-like | 3 | | | | o | o | | | |
| l antigen member 3 | 3 | o | o | | | o | | | |

| | | | | | | | | | |
|---|---|---|---|---|---|---|---|---|---|
| l1 cell adhesion molecule | 3 | o | | | | | o | o | |
| la ribonucleoprotein domain member 1 | 3 | o | | o | | | | | |
| lactation elevated 1 | 3 | o | | | | | o | o | |
| l-aspartate dehydrogenase | 3 | | o | o | | | | | o |
| latrophilin 2 | 3 | | | | | | o | | |
| leiomodin 2 | 3 | o | | | | | | | |
| leiomodin 3 | 3 | o | | | | | | | |
| letm1 domain-containing protein 1 | 3 | o | o | | | | | | |
| leucine carboxyl methyltransferase 1 | 3 | o | | | | o | | | |
| leucine rich repeat containing 15 | 3 | | o | o | | o | | | |
| leucine rich repeat containing 4b | 3 | | | | | | o | | |
| leucine rich repeat containing 8 member d | 3 | o | o | | | | | | |
| leucine-rich alpha-2-glycoprotein 1 | 3 | | o | o | | | | | |
| leucine-rich repeat-containing protein 14-like | 3 | o | | | | | | | |
| leucine-rich repeat-containing protein 3b-like | 3 | | | | o | | | | o |
| leucine-rich repeat-containing protein 40 | 3 | | | o | | | | | o |
| leukocyte surface antigen cd53 | 3 | o | | o | | o | | | |
| lim and senescent cell antigen-like domains 1 | 3 | | o | | | | | | o |
| lim and sh3 protein 1 | 3 | o | | o | | | o | | |
| lim domain containing isoform cra_a | 3 | o | | | | o | o | | |
| lim homeobox protein lhx8 | 3 | | | | o | | | | |
| lipid phosphate phosphohydrolase 1 | 3 | o | | | | | | o | |
| lipoic acid synthetase | 3 | o | o | o | | | | | |
| liver-type fatty acid-binding protein | 3 | | o | | | | | | |
| loc100003370 protein | 3 | | | | o | | | | |
| loc398481 protein | 3 | | | o | | | o | o | |
| loc446287 protein | 3 | | o | o | | | | | |
| loc496093 protein | 3 | o | o | o | | | | | |
| loc548392 protein | 3 | o | o | o | | | | | |
| loc553275 protein | 3 | o | | o | | o | | | |
| loc553334 protein | 3 | | o | | | o | | | o |
| loc562123 protein | 3 | o | | | | o | | | |
| loc569167 protein | 3 | o | | | | o | | o | |
| loc795096 protein | 3 | o | | | | | | | |
| long-chain-fatty-acid-- ligase acsbg2 | 3 | o | o | | | | | | |
| low affinity cationic amino acid transporter 2-like | 3 | o | | | o | | | | |
| low density lipoprotein receptor | 3 | | o | o | | | | | |
| low density lipoprotein receptor adaptor protein 1 | 3 | o | o | | | | | | |
| low quality protein: zinc finger homeobox protein 4-like | 3 | o | | | | | o | | |
| lrr and pyd domains-containing protein 3-like | 3 | | | | | | o | | o |
| l-threonine dehydrogenase | 3 | o | o | o | | | | | |
| lupus la protein homolog b | 3 | o | o | | | | | | |
| lymphocyte antigen 75 | 3 | o | | | | | | | |
| lymphoid-specific | 3 | o | | | | | | | |
| lysine-specific demethylase 3b | 3 | | o | | | | o | | |
| lysine-specific demethylase no66 | 3 | o | | | | | | o | |
| lysosomal membrane glycoprotein 2 | 3 | o | | o | | | | o | |
| lysosomal-associated transmembrane protein 4b | 3 | | | | | o | o | o | |
| macro domain containing 2 | 3 | | | | | | o | o | |
| mad2 mitotic arrest deficient-like 2 | 3 | o | o | | | o | | | |
| mads box transcription enhancer factor polypeptide c (myocyte enhancer factor 2c) | 3 | o | | | | | | | |
| major facilitator superfamily domain containing 5 | 3 | o | | | | o | o | | |
| major facilitator superfamily domain-containing protein 10 | 3 | o | | | | | | o | |

| | | | | | | | | | |
|---|---|---|---|---|---|---|---|---|---|
| major histocompatibility class i receptor | 3 | | | o | | | | o | o |
| maleylacetoacetate isomerase | 3 | o | o | o | | | | | |
| mannose c type 2 | 3 | o | | | | | o | | |
| mannose-6-phosphate isomerase | 3 | | | | | | o | o | |
| mannose-6-phosphate receptor binding protein 1 | 3 | o | o | o | | | | | |
| mannosyl-oligosaccharide glucosidase | 3 | o | o | o | | | | | |
| map kinase-interacting serine threonine kinase 2 | 3 | o | o | | | | | | |
| map7 domain containing 1-like | 3 | o | | | | | | | |
| math (meprin-associated traf homology) domain containing family member (math-33)-like | 3 | o | | o | | | | | |
| matrix gla protein | 3 | o | o | | | | | o | |
| matrix metalloproteinase 2 | 3 | o | | | | | o | | |
| matrix metalloproteinase-2 | 3 | | | o | | | | o | |
| mediator of rna polymerase ii transcription subunit 10 | 3 | o | | | | | o | o | |
| mediator of rna polymerase ii transcription subunit 29 | 3 | | o | | o | | | | |
| mediator of rna polymerase ii transcription subunit 7 | 3 | o | | o | | | | | |
| megakaryoblastic leukemia 1 | 3 | o | | | | | | o | |
| membrane protein | 3 | | | | o | | | o | |
| membrane-spanning 4- subfamily member | 3 | o | | o | | | | | |
| membrane-type matrix metalloproteinase | 3 | o | | o | | | o | | |
| meq protein | 3 | | | | | | | | o |
| methionine sulfoxide reductase b3 | 3 | o | o | o | | | | | |
| methionyl aminopeptidase 2 | 3 | o | | o | | | | o | |
| methylenetetrahydrofolate dehydrogenase (nadp+ dependent) methenyltetrahydrofolate cyclohydrolase | 3 | o | | o | | o | | | |
| methylmalonyl coenzyme a mutase | 3 | o | | | | | o | | |
| methyltransferase like 7a | 3 | o | o | | | | | | o |
| methyltransferase-like protein 10 | 3 | o | | o | | | | | |
| methyltransferase-like protein 5 | 3 | o | | | | o | | | |
| mgc82112 protein | 3 | | | | | | | | o |
| mhc class ii antigen alpha chain | 3 | | | o | | | | | |
| microtubule associated serine threonine kinase 2 | 3 | | | | | | o | | |
| microtubule-actin crosslinking factor 1 | 3 | o | | | | | o | o | |
| microtubule-associated protein rp eb family member 3 | 3 | o | | | | o | | o | |
| microtubule-associated rp eb member 1 | 3 | o | | | | o | | o | |
| microtubule-associated rp eb member 3 | 3 | o | | o | | | | | |
| midline 2 | 3 | o | | | | | | | |
| mitochondrial 28s ribosomal protein s33 | 3 | o | o | o | | | | | |
| mitochondrial 2-oxoglutarate malate carrier protein | 3 | o | | o | | | | | |
| mitochondrial 39s ribosomal protein l27 | 3 | o | | o | | o | | | |
| mitochondrial antiviral signaling | 3 | o | | | | | o | | |
| mitochondrial carnitine acylcarnitine carrier protein cacl | 3 | o | o | | | | | | |
| mitochondrial import inner membrane translocase subunit tim8 a | 3 | o | | o | | o | | | |
| mitochondrial nadh-ubiquinone oxidoreductase 75 kda subunit | 3 | o | o | | | | | | |
| mitochondrial ribosomal protein l2 | 3 | o | o | o | | | | | |
| mitochondrial ribosomal protein l20 | 3 | o | o | | | | o | | |
| mitochondrial ribosomal protein l28 | 3 | o | | | | | | | o |

| | | | | | | | | | |
|---|---|---|---|---|---|---|---|---|---|
| mitochondrial ribosomal protein l32 | 3 | o | o | | | o | | | |
| mitochondrial ribosomal protein l45 | 3 | o | | o | | | | | |
| mitochondrial ribosomal protein l48 | 3 | o | | | o | o | | | |
| mitochondrial ribosomal protein s10 | 3 | o | | | | o | o | | |
| mitochondrial ribosomal protein s16 | 3 | o | | o | | | | o | |
| mitochondrial ribosomal protein s2 | 3 | o | o | | | | | | o |
| mitochondrial ribosomal protein s21 | 3 | o | | | | o | o | | |
| mitochondrial ribosomal protein s23 | 3 | o | o | | | o | | | |
| mitochondrial ribosomal protein s24 | 3 | o | | o | | o | | | |
| mitochondrial ribosomal protein s36 | 3 | o | | | | o | | | |
| mitochondrial ribosome recycling factor | 3 | o | | | o | | | o | |
| mitochondrial trna pseudouridine synthase a | 3 | o | o | | | | | | o |
| mitogen-activated protein kinase 12 | 3 | o | | o | | | | | |
| mitogen-activated protein kinase 14 | 3 | o | | | o | | | | |
| mitogen-activated protein kinase 9 | 3 | o | | | | | o | o | |
| mitogen-activated protein kinase kinase kinase 14 | 3 | o | | | | | | | |
| mitogen-activated protein kinase kinase kinase 5 | 3 | o | | | | | o | | |
| mitogen-activated protein kinase kinase kinase 8 | 3 | o | o | | o | | | | |
| mitogen-activated protein kinase kinase kinase kinase 4 | 3 | o | | | | | | | |
| mitogen-activated protein kinase-activated protein kinase 2 | 3 | o | o | | | | | | |
| mitotic-spindle organizing protein 1-like | 3 | | | | o | | o | | |
| mmp37-like mitochondrial precursor | 3 | o | o | | | | | | |
| monocarboxylate transporter 9 | 3 | o | | | | | o | | |
| morc family cw-type zinc finger 3 | 3 | o | | o | | | | | |
| mortality factor 4 like 1 | 3 | o | | | o | | | | |
| mosc domain-containing protein mitochondrial precursor | 3 | o | o | | | o | | | |
| mothers against decapentaplegic homolog 2 | 3 | o | o | | | | | o | |
| m-phase phosphoprotein 8 | 3 | o | | | | o | o | | |
| multidrug and toxin extrusion protein 1 | 3 | | o | | | o | o | | |
| multiple ankyrin repeats single kh domain protein | 3 | o | | o | | o | | | |
| multiple inositol polyphosphate phosphatase 1 | 3 | | o | o | | | | | |
| multiple pdz domain protein | 3 | o | o | | | | | | |
| myeloid cell leukemia sequence 1 | 3 | o | o | o | | | | | |
| myosin 18a | 3 | o | | | | | | | |
| myosin binding protein h | 3 | o | | | | | | | |
| myosin ic | 3 | o | | | | o | | | |
| myosin phosphatase-rho interacting protein | 3 | o | | | | | | o | |
| myosin va | 3 | o | | | o | | o | | |
| myozenin 1 | 3 | o | | | | | | | |
| n-acetylglucosamine-1-phosphate gamma subunit | 3 | o | o | o | | | | | |
| n-acetyltransferase 10 | 3 | | | | o | o | | | |
| nad kinase | 3 | | o | | | | | | o |
| nad-dependent epimerase dehydratase | 3 | o | | | | | | | o |
| nadh dehydrogenase 1 alpha 3 | 3 | o | | | | o | | o | |
| nadh dehydrogenase 1 alpha 39kda | 3 | o | | o | | | | o | |
| nadh dehydrogenase 1 beta subcomplex subunit 1 | 3 | o | | | | o | | | |
| nardilysin (n-arginine dibasic convertase) | 3 | o | o | | | | | | |
| nck-associated protein 5-like | 3 | o | | | | | o | | |
| nebulin-related anchoring protein | 3 | o | o | | | | | | |
| nedd8-activating enzyme e1 catalytic subunit | 3 | o | | | | o | | o | |

| | | | | | | | | | |
|---|---|---|---|---|---|---|---|---|---|
| netrin receptor unc5c-like | 3 | | | | | | o | | |
| neural cell adhesion molecule l1-like protein | 3 | | o | | | | o | | o |
| neuroblast differentiation-associated protein ahnak-like | 3 | | o | | | | | | o |
| neuroendocrine protein 7b2-like | 3 | | | | | | | | o |
| neuroepithelial cell transforming gene 1 | 3 | o | o | | | | | o | |
| neurofilament medium polypeptide | 3 | | | | | | o | | o |
| neurogenic differentiation 2 | 3 | | | | | o | | o | |
| neuroligin 1 | 3 | | | | | | o | | |
| neuropilin 1a | 3 | o | | | | | o | | |
| neuroplastin precursor | 3 | | | | | | | | o |
| neuroserpin precursor | 3 | | | | | | o | o | |
| neutrophil cytosolic factor 2 | 3 | o | | o | | | | o | |
| nfu1 iron-sulfur cluster scaffold homolog | 3 | o | o | | | o | | | |
| nicotinamide mononucleotide adenylyltransferase 1 | 3 | o | o | o | | | | | |
| ninjurin 1 | 3 | | | | | o | | o | o |
| nipsnap1 protein | 3 | | o | o | o | | | | |
| nitric oxide synthase-interacting protein | 3 | o | | o | | | | | |
| nitrilase member 2 | 3 | o | o | | | | | | |
| n-myc downstream regulated gene 1 | 3 | o | o | | | | | | o |
| novel carboxylesterase domain containing protein | 3 | o | o | | | o | | | |
| novel immune-type receptor | 3 | o | | | o | | | | |
| novel protein (zgc:153928) | 3 | o | | | | | | | |
| novel protein (zgc:77651) | 3 | | o | o | | | | | |
| novel protein (zgc:85741) | 3 | | o | | | | o | | |
| novel protein biglycan-like protein 3 ( zgc:77123) | 3 | | o | | | | o | o | |
| novel protein containing 10 heat domains | 3 | o | | | | | o | | |
| novel protein human and mouse sema transmembrane domain and cytoplasmic 6d | 3 | o | | | | | o | | |
| novel protein human ankyrin repeat and sterile alpha motif domain containing 1a | 3 | | | | | | o | o | |
| novel protein ovary-specific acidic protein | 3 | o | | | | | | | o |
| novel protein vertebrate hla-b associated transcript 2 | 3 | o | | | | | o | | |
| novel protein vertebrate plectin intermediate filament binding protein 500kda | 3 | o | | | | | | | |
| nuclear envelope pore membrane protein pom 121-like | 3 | o | | | | | | | |
| nuclear nf-kappab activating protein | 3 | | | | | o | o | o | |
| nuclear receptor subfamily group member 1 | 3 | o | o | o | | | | | |
| nuclear rna export factor 1 | 3 | o | | | | o | | o | |
| nucleobindin 2 | 3 | | o | | | | | | |
| nucleolar complex associated 4 homolog ( cerevisiae) | 3 | o | o | o | | | | | |
| nucleolar complex protein 4 homolog | 3 | | | | o | | | | o |
| nucleolar protein 12 | 3 | o | | | | | | | o |
| nucleolar protein 14 | 3 | o | o | o | | | | | |
| nucleolar protein with mif4g domain 1 | 3 | | | | | o | | | |
| nucleoporin nup43 | 3 | o | | | o | | | | |
| nucleoporin p54 | 3 | | | | o | | | | |
| nudix (nucleoside diphosphate linked moiety x)-type motif 1 | 3 | o | | o | | o | | | |
| obscurin-like 1 | 3 | o | | | | | | | |
| oculocerebrorenal syndrome of lowe | 3 | o | | | | o | o | | |
| odd oz ten-m homolog 3 | 3 | o | o | | | | o | | |
| olfactomedin 2 | 3 | | | | o | o | | | |
| olfactomedin-like 2b | 3 | o | | | | o | | | o |

| | | | | | | | | | |
|---|---|---|---|---|---|---|---|---|---|
| oligodendrocyte lineage transcription factor 2 | 3 | | | | | | | | o |
| opposite strand transcription unit to stag3 | 3 | o | | | | o | | o | |
| optic atrophy 3 | 3 | o | | o | | o | | | |
| optineurin | 3 | o | o | | | o | | | |
| ornithine carbamoyltransferase | 3 | | | | o | | | o | |
| ornithine decarboxylase | 3 | o | o | | | | | | |
| orphan sodium- and chloride-dependent neurotransmitter transporter ntt73 | 3 | o | | | | | o | o | |
| osteoblast specific factor | 3 | o | | | | | | | |
| osteoclast-stimulating factor 1 | 3 | | o | | | o | | o | |
| otu domain containing 3 | 3 | o | o | | | o | | | |
| outcome predictor in acute leukemia 1-like | 3 | o | | | | | | | |
| oxidoreductase glyr1 | 3 | o | | | | | | o | |
| oxysterol-binding protein 3 | 3 | o | | | o | | | | |
| oxysterols receptor lxr-alpha | 3 | | o | | | | | | o |
| p150 | 3 | o | | | | | o | o | |
| p21-activated kinase 3 | 3 | o | | | | | o | | |
| pdz and lim domain 1 | 3 | | o | | o | | | o | |
| pdz and lim domain 2 | 3 | | o | o | o | | | | |
| pdz and lim domain protein 1 | 3 | o | o | | o | | | | |
| pdz domain containing 8 | 3 | o | | | | | o | | |
| pentatricopeptide repeat-containing protein mitochondrial precursor | 3 | o | o | | | | | | |
| peptidase d | 3 | o | o | | | | | | o |
| peptidyl-prolyl cis-trans isomerase cwc27 homolog | 3 | o | | | | | | o | |
| peptidyl-prolyl cis-trans isomerase d | 3 | | | | | | | | o |
| peptidyl-prolyl cis-trans isomerase fkbp14-like | 3 | | | | o | | | | |
| peptidyl-prolyl cis-trans isomerase fkbp1b | 3 | o | | | | | | | o |
| peptidylprolyl isomerase b (cyclophilin b) | 3 | o | | o | | | | o | |
| peptidylprolyl isomerase -like 3 | 3 | o | o | o | | | | | |
| perforin 1 (pore forming protein) | 3 | o | | o | | | | | |
| peroxisomal acyl-coenzyme a oxidase 1 | 3 | o | | | | | | | |
| peroxisomal -trans-enoyl- isomerase | 3 | o | o | o | | | | | |
| peroxisome proliferator-activated receptor beta | 3 | | o | | | | | | o |
| per-pentamer repeat gene | 3 | | | | o | | | | |
| pg1 homology to homo sapiens | 3 | o | | | | | | o | |
| pg1 protein | 3 | | | o | | | | o | |
| phosphatase and actin regulator 2 | 3 | o | | o | | | | | |
| phosphatidylethanolamine-binding protein variant 1 | 3 | o | | o | | | | o | |
| phosphatidylinositol 3-kinase regulatory subunit alpha | 3 | o | | | | | | | |
| phosphatidylinositol 3-kinase regulatory subunit alpha-like | 3 | o | o | | | | | | |
| phosphatidylinositol- -trisphosphate 3-phosphatase and dual-specificity protein phosphatase pten | 3 | | | | | | o | | o |
| phosphatidylinositol- -trisphosphate 5-phosphatase 1 | 3 | o | | | o | | | | |
| phosphatidylinositol-binding clathrin assembly | 3 | | o | | | | o | | |
| phosphatidylinositol-glycan biosynthesis class f protein | 3 | | o | o | o | | | | |
| phosphoacetylglucosamine mutase | 3 | o | | o | | o | | | |
| phosphoglucomutase 1 | 3 | o | o | | | | | o | |
| phosphoglucomutase 2 | 3 | o | o | o | | | | | |
| phospholipase a1 member a precursor | 3 | | | | o | | | | o |

| | | | | | | | | | |
|---|---|---|---|---|---|---|---|---|---|
| phospholipase b-like 2-like | 3 | | o | o | | o | | | |
| phospholipase d3 | 3 | o | o | o | | | | | |
| phospholipase group pancreas | 3 | | o | | | | | | |
| phosphopantothenate--cysteine ligase | 3 | | o | | o | | | | |
| phosphorylase b kinase gamma catalytic skeletal muscle isoform | 3 | | o | o | | o | | | |
| phosphorylase beta | 3 | o | o | | | | | | |
| phosphotriesterase-related protein | 3 | o | o | o | | | | | |
| pin2-interacting protein x1 | 3 | o | | | | | o | o | |
| pla2g15 protein | 3 | o | o | | | | | | |
| placenta growth factor-like | 3 | | | | | | | | o |
| plasma glutamate carboxypeptidase | 3 | o | o | o | | | | | |
| plasminogen precursor | 3 | | | | o | | | | |
| plasticity related gene 1 | 3 | | | | | | o | | |
| plastin 3 (t isoform) | 3 | o | | | | | o | | |
| pleckstrin homology domain family m (with run domain) member 2 | 3 | o | | | | | o | | |
| pleckstrin homology domain-containing family j member 1 | 3 | o | | | o | | | o | |
| plethodontid modulating factor | 3 | | o | | | | | | |
| podocan | 3 | o | | o | | | | | |
| polo-like kinase 3 | 3 | o | | | | | o | | |
| poly (adp-ribose) polymerase member 12 | 3 | o | | | | o | | | |
| polycystic kidney disease 1-like 2 | 3 | o | o | | | | | | |
| polymerase (dna directed) delta regulatory subunit | 3 | o | | | o | | o | | |
| polymerase (dna-directed) delta interacting protein 2 | 3 | o | o | | | | o | | |
| polymerase i polypeptide 53kda | 3 | o | | o | | | | | |
| polymerase ii (dna directed) polypeptide | 3 | o | o | | | o | | | |
| polymerase ii (dna directed) polypeptide d | 3 | o | | | | o | | o | |
| polypyrimidine tract binding protein 2 | 3 | o | | | | | o | | |
| polypyrimidine tract-binding protein 1 | 3 | o | | o | | | | o | |
| potassium channel tetramerisation domain containing | 3 | | | | | | | | o |
| potassium voltage-gated subfamily member 1 | 3 | | | o | | o | | | o |
| pou domain class 5 transcription factor 1 | 3 | | | | o | | | | |
| pq loop repeat containing 3 | 3 | o | | | | o | | o | |
| pr domain containing with znf domain | 3 | o | | | o | | o | | |
| PREDICTED: ReO_6-like [Danio rerio] | 3 | o | | o | | | | o | |
| PREDICTED: similar to predicted protein [Equus caballus] | 3 | | | | | o | | | |
| PREDICTED: similar to predicted protein, partial [Hydra magnipapillata] | 3 | o | | | o | o | | | |
| preli domain containing 1 | 3 | o | o | o | | | | | |
| pre-mrna-splicing factor atp-dependent rna helicase dhx15-like | 3 | | | | | | | | o |
| prenyl diphosphate subunit 1 | 3 | o | | o | | o | | | |
| pre-rrna-processing protein tsr2 homolog | 3 | o | | | | o | | | o |
| proactivator polypeptide | 3 | o | | | | | | | o |
| probable arylformamidase | 3 | o | o | | | | | | |
| probable asparaginyl-trna mitochondrial | 3 | o | | o | | | | | |
| probable atp-dependent rna helicase dhx58 | 3 | o | | o | | | | o | |
| probable bax inhibitor 1 | 3 | | | | | | | | o |
| probable histone acetyltransferase myst1 | 3 | | | | o | | o | | |
| probable methylthioribulose-1-phosphate dehydratase | 3 | o | | o | o | | | | |
| probable methyltransferase-like protein 15-like | 3 | | | | o | | | | |
| probable proline dehydrogenase 2 | 3 | | o | | o | | | | |

| | | | | | | | | | |
|---|---|---|---|---|---|---|---|---|---|
| probable rna-directed dna polymerase from transposon bs-like | 3 | | o | | | | o | | o |
| programmed cell death 10 | 3 | o | o | o | | | | | |
| programmed cell death 4 | 3 | o | o | o | | | | | |
| programmed cell death protein 6 | 3 | o | | o | | | | o | |
| proliferation-associated 2g4 | 3 | o | | | | | | | |
| proline synthetase co-transcribed bacterial homolog protein | 3 | | o | | | o | | | o |
| proline-rich protein partial | 3 | o | | | | | | | o |
| prolyl 4- beta polypeptide | 3 | o | | o | | | | | |
| prominin 1 | 3 | o | | | | | | o | |
| proprotein convertase subtilisin kexin type 2 | 3 | | | | | o | o | o | |
| prosaposin | 3 | o | | o | | | | | |
| prospero homeobox protein 1 | 3 | | o | | | | o | | |
| prostaglandin e synthase 2 | 3 | o | | | | o | | o | |
| prostaglandin e synthase 2-like | 3 | | | | o | | | | o |
| protachykinin 1 precursor | 3 | | | | | o | | o | o |
| proteasomal ubiquitin receptor adrm1 | 3 | o | o | | | | o | | |
| proteasome ( macropain) 26s 5 | 3 | o | o | | | o | | | |
| proteasome ( macropain) 26s non- 7 | 3 | o | | o | | | | o | |
| proteasome subunit alpha type-1 | 3 | | o | | | o | o | | |
| proteasome subunit beta type-9 precursor | 3 | o | | o | | | | | |
| protein arginine methyltransferase 1 | 3 | o | o | | | | | o | |
| protein arginine n-methyltransferase 7 | 3 | o | o | | | o | | | |
| protein ariadne-1 homolog | 3 | o | o | | | | | | |
| protein btg3 | 3 | | | | o | | | | |
| protein cdv3 homolog | 3 | o | | | | | o | | o |
| protein churchill | 3 | o | o | | | o | | | |
| protein fam131a-like | 3 | | | | | | o | | o |
| protein fam171a2-like | 3 | | | | | | o | o | |
| protein fam5c-like | 3 | | | | | | | | o |
| protein fam84b-like | 3 | | | | o | | | | |
| protein kiaa0664-like | 3 | | | | | | o | o | o |
| protein kinase c beta type | 3 | | | | | | o | o | |
| protein lin-7 homolog c | 3 | o | o | o | | | | | |
| protein lsm14 homolog a | 3 | o | | | | | | o | |
| protein mak16 homolog | 3 | o | | o | o | | | | |
| protein ndrg2 | 3 | o | | | | | o | o | |
| protein phosphatase 1 regulatory subunit 1c | 3 | o | | | | o | | | o |
| protein phosphatase 1a (formerly 2c) magnesium- alpha isoform | 3 | o | | | | | | o | |
| protein phosphatase 2 (formerly 2a) regulatory subunit beta isoform | 3 | o | | | | | o | o | |
| protein phosphatase ptc7 homolog | 3 | o | | | | | | | |
| protein phosphatase regulatory subunit 3c | 3 | o | | o | | | | | |
| protein phosphatase regulatory subunit 3d | 3 | o | | | | | | | |
| protein sec13 homolog | 3 | o | o | | o | | | | |
| protein spire homolog 1-like | 3 | | | | | | | | o |
| protein tbrg4 | 3 | | | | o | | | | o |
| protein transport protein sec16b | 3 | o | | | | | | | |
| protein transport protein sec31a | 3 | | | | | | o | | |
| protein transport protein sec61 subunit gamma-like | 3 | | o | | | | o | | o |
| protein tweety homolog 3 | 3 | | | | | | o | o | |
| protein tyrosine non-receptor type 21 | 3 | o | o | o | | | | | |
| protein tyrosine non-receptor type 9 | 3 | o | o | | | | | | |
| protein tyrosine phosphatase e | 3 | o | | | | | o | | o |
| protein tyrosine receptor n polypeptide 2 | 3 | | | | | | o | o | |
| protein yipf3 | 3 | o | o | | | | | o | |

| | | | | | | | | | |
|---|---|---|---|---|---|---|---|---|---|
| protein-l-isoaspartate(d-aspartate) o-methyltransferase | 3 | o | | | | | | | |
| protocadherin 18 | 3 | o | | | | | o | | |
| protocadherin fat 3-like | 3 | | | | | | o | | |
| proto-oncogene serine threonine-protein kinase pim-1-like | 3 | o | | | | o | | | |
| psmd1 protein | 3 | o | | o | | | | | |
| pten induced kinase 1 | 3 | | | | | | | | o |
| purine nucleoside phosphorylase-like | 3 | | | | o | | | | o |
| purinergic receptor ligand-gated ion 1 | 3 | | | o | o | | | | |
| purinergic receptor ligand-gated ion 5 | 3 | o | o | | | | | | |
| pyrophosphatase 1 | 3 | o | | | | o | | o | |
| rab5-interacting protein | 3 | o | o | o | | | | | |
| radixin | 3 | o | | | | | | | |
| ran binding protein 1 | 3 | o | | | | | | o | |
| ran binding protein 3 | 3 | o | | | | | | | |
| rap guanine nucleotide exchange factor 4 | 3 | | | | | | o | | |
| ras association domain-containing protein 1 | 3 | o | o | | | | | | |
| ras homolog gene member a | 3 | o | o | | | | | | o |
| ras-related protein rab-10 | 3 | o | o | | | o | | | |
| ras-related protein rab-2a | 3 | o | o | | | | o | | |
| ras-related protein rab-9a | 3 | | | | | o | | o | o |
| receptor activity modifying protein 1 | 3 | o | | | | | | | |
| receptor-type tyrosine-protein phosphatase n2-like | 3 | | | | o | | | | o |
| receptor-type tyrosine-protein phosphatase s | 3 | | | | | | o | | |
| receptor-type tyrosine-protein phosphatase zeta | 3 | | | | | | o | | |
| receptor-type tyrosine-protein phosphatase-like n | 3 | | | | | | o | | o |
| reelin isoform 1 | 3 | | | | | | o | o | |
| regulating synaptic membrane exocytosis protein 1-like | 3 | | | | | | o | | |
| regulator of g-protein signaling 20 | 3 | o | | | | o | | | o |
| replicase helicase endonuclease-like | 3 | | | o | | | | o | |
| replication protein 14kda | 3 | o | | | | o | o | | |
| reproduction regulator 2 | 3 | o | | | | o | | | |
| reticulon 2 | 3 | o | | | | | o | | |
| retinal dehydrogenase 2 | 3 | o | | | | | o | | |
| retinal g protein coupled receptor | 3 | | o | | | o | | | |
| retinaldehyde binding protein 1 | 3 | o | | | | o | | o | |
| retinitis pigmentosa 9 protein homolog | 3 | o | o | | o | | | | |
| retinoid x receptor alpha | 3 | o | | | | | o | | o |
| retinol dehydrogenase 14 | 3 | o | | | o | | | | o |
| rho gtpase activating protein 12 | 3 | o | | | | | o | | |
| rho gtpase activating protein 5 | 3 | o | | | | | | | |
| rho gtpase-activating protein 12 | 3 | o | | | | | o | | |
| rhoptry protein | 3 | | | | | | | | o |
| rho-related btb domain-containing protein 2 | 3 | o | | | | o | | o | |
| ribonuclease h1 | 3 | o | | | | | | | o |
| ribonuclease p 21kda subunit | 3 | o | | o | | | | o | |
| ribonuclease t2 | 3 | o | o | | | | | | |
| ribophorin i | 3 | o | o | | | o | | | |
| ribose-phosphate pyrophosphokinase 1 | 3 | | | o | | | o | o | |
| ribosomal protein s19 | 3 | o | | o | | o | | | |
| ribosomal protein s28 | 3 | o | o | | | o | | | |
| ribosome production factor 1 | 3 | o | o | o | | | | | |
| ribosome-releasing factor mitochondrial | 3 | | o | | o | | | | |
| riken cdna 2810453i06 gene | 3 | o | o | o | | | | | |
| ring finger protein 114 | 3 | o | | | o | o | | | |

| | | | | | | | | | |
|---|---|---|---|---|---|---|---|---|---|
| ring finger protein 122 | 3 | o | | | | | | o | |
| ring finger protein 175 | 3 | o | o | o | | | | | |
| ring finger protein 24 | 3 | o | | | | | | | |
| ring finger protein 31 | 3 | | o | | | | o | | |
| rna 3 -terminal phosphate cyclase-like protein | 3 | o | | | | o | | | |
| rna binding motif protein 17 | 3 | o | | o | | o | | | |
| rna binding single stranded interacting protein 1 | 3 | o | | | | | | o | |
| rna methyltransferase like 1 | 3 | o | | | o | | | | |
| rna polymerase-associated protein ctr9 homolog | 3 | o | o | | | | o | | |
| rna terminal phosphate cyclase-like 1 | 3 | | o | | | | | | o |
| rna-binding protein 12 | 3 | | | o | | o | | o | |
| rna-binding protein 39 | 3 | o | o | | | o | | | |
| rwd domain-containing protein 4-like | 3 | | | | o | | | | o |
| s100 calcium binding protein a14 | 3 | o | o | | o | | | | |
| s100 calcium binding protein a16 | 3 | o | | | | o | o | | |
| s-adenosylmethionine synthase isoform type-2 | 3 | o | o | o | | | | | |
| saps domain member 3 | 3 | o | | o | | | | o | |
| sarcoma amplified sequence | 3 | o | o | o | | | | | |
| sarcoplasmic endoplasmic reticulum calcium atpase 2 | 3 | o | | | | o | o | | |
| scrn2_danre ame: full=secernin-2 | 3 | o | | | | | | | |
| sec1 family domain-containing protein 1 | 3 | | o | o | o | | | | |
| sec1 family domain-containing protein 1-like | 3 | | | o | | o | | | |
| sec61 beta subunit | 3 | o | o | o | | | | | |
| secretory carrier membrane protein 4 | 3 | o | o | | | | | | |
| seizure protein 6 homolog | 3 | | | | | | o | | |
| selenium binding protein 1 | 3 | | o | | o | | | | |
| sept2 protein | 3 | o | o | | | | | | |
| septin 8 | 3 | o | | | | o | | | |
| septin 9b | 3 | o | o | | | | | | |
| serine threonine-protein kinase lmtk1-like | 3 | | | | | | o | | |
| serine threonine-protein kinase pak 2 | 3 | o | o | | | | | | o |
| serine threonine-protein kinase plk1 | 3 | o | o | | o | | | | |
| serine threonine-protein kinase sgk3 | 3 | o | | | o | | | | |
| serine threonine-protein kinase sik2 | 3 | o | | | | | o | | |
| serine threonine-protein kinase ulk1 | 3 | o | o | | | | | | |
| serine threonine-protein phosphatase 2a activator | 3 | | o | o | | | | o | |
| serine--pyruvate aminotransferase | 3 | | o | | | | | | |
| serpine1 mrna binding protein 1 | 3 | o | | | o | | | | o |
| serrate rna effector molecule homolog | 3 | o | o | | | | | | o |
| seryl-trna synthetase | 3 | | o | o | | | | o | |
| set and mynd domain-containing protein 5 | 3 | o | | | o | | | | |
| set domain containing 3 | 3 | o | o | | | | | | |
| sex hormone-binding globulin | 3 | | o | | | | | | |
| sf3b1 protein | 3 | | | o | | | o | o | |
| shc (src homology 2 domain containing) transforming protein 1 | 3 | o | | | | | | o | |
| short chain dehydrogenase reductase family member 5 | 3 | | o | o | | | | | |
| sialic acid acetylesterase | 3 | o | o | o | | | | | |
| sialic acid synthase | 3 | o | o | | | | | | o |
| signal cub egf-like 1 | 3 | | | | | o | o | o | |
| signal recognition particle 54 kda protein | 3 | o | o | | | | | | |
| signal recognition particle receptor subunit alpha | 3 | o | o | o | | | | | |

| | | | | | | | | | |
|---|---|---|---|---|---|---|---|---|---|
| signal transducer and activator of transcription 1 | 3 | o | | o | | | | | |
| sin3 histone deacetylase corepressor complex component sds3 | 3 | o | | | | o | | | |
| single-stranded dna-binding mitochondrial | 3 | o | | o | | | | o | |
| small muscle x-linked | 3 | o | | | | o | | | |
| small nuclear ribonucleoprotein f | 3 | o | o | | | o | | | |
| small nuclear ribonucleoprotein sm d3 | 3 | o | o | | | o | | | |
| small nuclear rna activating polypeptide 43kda | 3 | o | o | | | | | | |
| small subunit processome homolog | 3 | o | o | | | | | o | |
| smc5 partial | 3 | o | | | | | | | |
| smoothelin isoform 2 | 3 | o | | | | | o | | |
| smoothelin-like protein 2 | 3 | o | | | | | | | |
| snrnp200 protein | 3 | o | | | o | | | | o |
| snw domain containing 1 | 3 | | | o | | | | | |
| sodium- and chloride-dependent neurotransmitter transporter | 3 | o | | | | o | | o | |
| sodium bicarbonate transporter-like protein 11 | 3 | | | | | | o | | o |
| sodium voltage- type beta | 3 | o | | | | | | o | |
| solute carrier family 12 member 9-like | 3 | | | | o | o | | | o |
| solute carrier family 2 (facilitated glucose transporter) member 1 | 3 | o | | | | | o | o | |
| solute carrier family 24 (sodium potassium calcium exchanger) member 3 | 3 | | | | | | o | | |
| solute carrier family 24 (sodium potassium calcium exchanger) member 6 | 3 | o | o | | | | | | |
| solute carrier family 25 (mitochondrial carrier oxoglutarate carrier) member 11 | 3 | o | o | | | | | | |
| solute carrier family 25 (mitochondrial oxodicarboxylate carrier) member 21 | 3 | o | o | | | | | | |
| solute carrier family 30 (zinc transporter) member 4 | 3 | o | o | | | | | | |
| solute carrier family 35 member b1 | 3 | o | o | | | o | | | |
| solute carrier family 39 (metal ion transporter) member 11 | 3 | | | o | | | | | |
| solute carrier family 6 (neurotransmitter gaba) member 13 | 3 | o | | | | o | | | |
| solute carrier family member 17 | 3 | o | | | | | o | o | |
| solute carrier family member 29 | 3 | o | o | | | | | | |
| solute carrier organic anion transporter member 5a1 | 3 | o | | o | | | | o | |
| sorcin-like isoform 2 | 3 | | o | | | | | | o |
| sorting and assembly machinery component 50 homolog | 3 | | | | | o | | | o |
| sorting and assembly machinery component 50 homolog ( cerevisiae) | 3 | o | | | | o | | o | |
| sorting nexin-10 | 3 | o | | | o | | | | |
| sorting nexin-14 | 3 | | | o | | | | o | o |
| sorting nexin-15 | 3 | o | o | | | o | | | |
| sorting nexin-1-like | 3 | o | | o | | | | o | |
| sorting nexin-6-like | 3 | o | o | | | | | | |
| source of immunodominant mhc-associated peptides | 3 | | | o | | o | | | |
| spastin | 3 | o | | o | | o | | | |
| sperm-activating peptide i precursor | 3 | | | | o | | | | |
| spermidine synthase | 3 | o | o | | | | | o | |
| spg7 protein | 3 | o | o | | o | | | | |
| sphingosine 1-phosphate receptor 2 | 3 | o | | o | | | o | | |

| | | | | | | | | | |
|---|---|---|---|---|---|---|---|---|---|
| splicing arginine serine-rich 6 | 3 | o | o | | | | | | |
| splicing factor 3a subunit 1 | 3 | o | o | o | | | | | |
| splicing factor 3b subunit 3 | 3 | o | o | | | | o | | |
| splicing factor 4 | 3 | o | | | | o | | | |
| splicing factor subunit 155kda | 3 | o | o | | | | o | | |
| spondin extracellular matrix protein | 3 | o | | | | | o | o | |
| spry domain containing 4 | 3 | o | | o | | | | | |
| spry domain-containing socs box protein 3 | 3 | o | | | | o | o | | |
| st3 beta-galactoside alpha- -sialyltransferase 4 | 3 | o | | o | | | | | |
| stard3 n-terminal like | 3 | o | | | | o | | o | |
| stathmin 1 oncoprotein 18 variant 8 | 3 | o | | | | o | | | |
| stathmin-like 2 | 3 | | | | | o | | | o |
| sterol regulatory element binding transcription factor 1 | 3 | o | | | | | o | o | |
| stomatin-like protein 2 | 3 | | o | | | | | | o |
| stromal cell-derived factor 2-like 1 | 3 | | o | o | | | | | |
| structural maintenance of chromosomes 1a | 3 | o | | | | o | o | | |
| structural maintenance of chromosomes protein 4 | 3 | o | | | | | o | | |
| structural maintenance of chromosomes protein 5 | 3 | o | | | o | | | | |
| structural maintenance of chromosomes protein 6 | 3 | o | | | o | | | | |
| subfamily member 19 | 3 | o | o | | | | | o | |
| subfamily member 3b | 3 | o | | | | | | | |
| subfamily member 7 | 3 | o | o | | | | | | |
| succinate dehydrogenase assembly factor mitochondrial-like | 3 | o | | | o | | | | |
| sulfide quinone reductase-like | 3 | o | o | | | | | o | |
| superkiller viralicidic activity 2-like 2 | 3 | o | | o | | | | | |
| supervillin | 3 | o | | | | | | | |
| suppressor of g2 allele of skp1 homolog | 3 | o | | o | | | o | | |
| suppressor of swi4 1 homolog | 3 | o | o | o | | | | | |
| surfeit locus protein 4 | 3 | o | | o | | o | | | |
| swi snf matrix actin dependent regulator of subfamily member 1 | 3 | o | | | | | | | o |
| swi snf matrix actin dependent regulator of subfamily member 3 | 3 | o | | | | o | | o | |
| synaptogyrin 3 | 3 | | | | | | | o | o |
| synaptojanin-2-binding protein | 3 | o | | o | o | | | | |
| synaptosomal-associated protein 23 | 3 | o | o | | | | | | |
| synaptosomal-associated protein 29 | 3 | o | o | o | | | | | |
| syndecan binding protein | 3 | o | o | | | | | o | |
| synovial apoptosis inhibitor synoviolin | 3 | o | | o | | | | | |
| syntaxin 18 | 3 | o | | o | | | | | |
| syntaxin 1b | 3 | | | | | | o | o | |
| syntaxin 8 | 3 | o | o | | | | | | o |
| talin 1 | 3 | o | | | | | | | |
| tar dna-binding protein 43 | 3 | o | | | | | | o | |
| taxilin beta | 3 | o | | | | | | | |
| tbc1 domain member 13 | 3 | o | | o | | | | | |
| tbc1 domain member 15 | 3 | | o | | | o | | | |
| tc1-like transporase | 3 | | | o | | | o | o | |
| t-cell immunoglobulin and mucin domain containing 4 | 3 | o | | | | o | o | | |
| t-cell lymphoma invasion and metastasis 1 | 3 | | | | | | o | | |
| t-cell receptor beta chain | 3 | o | | | | | | o | o |
| t-complex polypeptide 1 | 3 | | | o | | | | o | o |
| tetraspanin 13 | 3 | | o | o | | | | | o |
| tetraspanin 4 | 3 | o | | | | | | o | |

| | | | | | | | | | |
|---|---|---|---|---|---|---|---|---|---|
| tetraspanin-3-like isoform 2 | 3 | | | | | | | | o |
| tetratricopeptide repeat domain 17 | 3 | o | | o | | | o | | |
| thiopurine s-methyltransferase | 3 | | o | o | | o | | | |
| thioredoxin domain-containing protein 12 precursor | 3 | | | | o | | | | o |
| thioredoxin domain-containing protein 15 | 3 | o | | o | | | | o | |
| thioredoxin-related transmembrane protein 2 | 3 | o | | | | | o | | |
| thiosulfate sulfurtransferase | 3 | o | | o | | | | | |
| tho complex subunit 4 | 3 | | o | | o | | | | o |
| tho complex subunit 5 homolog | 3 | | o | o | | o | | | |
| threonine synthase-like 1 | 3 | o | o | | | | | | |
| thrombospondin 1 | 3 | o | | | | | | | |
| thromboxane-a synthase | 3 | o | | | | | o | | |
| thymidine cytosolic | 3 | | o | | o | | o | | |
| tia1 cytotoxic granule-associated rna binding protein | 3 | o | | | | | o | | |
| tight junction protein 2 (zona occludens 2) | 3 | | o | | | | o | | |
| tissue factor pathway inhibitor 2 precursor | 3 | o | | o | o | | | | |
| tnfaip3-interacting protein 1 | 3 | o | o | | | | | | o |
| topoisomerase i | 3 | o | | | o | | | | o |
| tox high mobility group box family member 2 | 3 | o | | | | | o | | |
| tpa: calcitonin receptor | 3 | | | | | | o | | |
| tpa_exp: replicase helicase endonuclease | 3 | o | | | | | o | o | |
| traf2 and nck interacting kinase | 3 | o | | | | | | o | |
| trafficking protein particle complex subunit 2 | 3 | o | | | | o | | | |
| trafficking protein particle complex subunit 2-like protein | 3 | o | | | | o | | o | |
| transcription elongation factor spt4 | 3 | o | | | | o | | o | |
| transcription factor 20 | 3 | | o | | | o | | | o |
| transcription factor 25 (basic helix-loop-helix) | 3 | o | o | | | | | | |
| transcription factor sox-8 | 3 | | | | | | o | o | |
| transcription initiation factor iia subunit 2 | 3 | o | | | | | | | o |
| transcription initiation factor tfiid subunit 13 | 3 | o | | o | | o | | | |
| transcriptional activator protein pur-beta | 3 | o | | | | | o | o | |
| transcriptional adapter 3-like | 3 | | | | o | | | | o |
| transcriptional enhancer factor tef-5-like | 3 | o | | | | | | | |
| transcriptional regulator atrx-like | 3 | | | | o | | o | | o |
| transducin -like 2 | 3 | o | | o | | | | | |
| transferase c1orf69 mitochondrial-like | 3 | o | | o | | o | | | |
| transforming acidic coiled coil 1b isoform b | 3 | | | | | | o | | o |
| transforming growth beta receptor iii | 3 | o | | | | | | | |
| transketolase-like 2 | 3 | o | o | | o | | | | |
| translation initiation factor eif-2b subunit alpha | 3 | o | | o | | | | o | |
| translocase of inner mitochondrial membrane 10 homolog | 3 | o | o | | | o | | | |
| translocase of inner mitochondrial membrane 23 homolog | 3 | o | o | o | | | | | |
| translocation associated membrane protein 2 | 3 | o | o | o | | | | | |
| transmembrane 4 l6 family member 5 | 3 | | | | o | | | | |
| transmembrane 9 superfamily member 4 precursor | 3 | | o | o | | | | o | |
| transmembrane and coiled-coil domain-containing protein c6orf129 homolog | 3 | o | | o | | | | o | |
| transmembrane anterior posterior transformation 1-like | 3 | | o | o | | | | o | |
| transmembrane anterior posterior transformation protein 1 homolog | 3 | o | | o | | | | | |
| transmembrane bax inhibitor motif containing 4 | 3 | o | o | | | | | o | |

| | | | | | | | | | |
|---|---|---|---|---|---|---|---|---|---|
| transmembrane emp24 domain trafficking protein 2 | 3 | o | | o | | | o | | |
| transmembrane phosphatase with tensin homology | 3 | o | | | | | | o | |
| transmembrane protease serine 9-like | 3 | | | | o | | | | o |
| transmembrane protein 14a | 3 | o | | o | | | | o | |
| transmembrane protein 167b | 3 | o | | o | o | | | | |
| transmembrane protein 181 | 3 | o | | o | | | | o | |
| transmembrane protein 184b | 3 | | | | | | o | o | |
| transmembrane protein 220 | 3 | o | | o | | | | o | |
| transmembrane protein 222 | 3 | | o | o | | o | | | |
| transmembrane protein 33 | 3 | o | o | | | | | | o |
| transmembrane protein 38b | 3 | o | o | o | | | | | |
| transmembrane protein 41b | 3 | o | o | | | | | | |
| transmembrane protein 47 | 3 | o | o | o | | | | | |
| transmembrane protein 69 | 3 | o | | | | o | | | |
| transmembrane protein loc124446 | 3 | o | o | o | | | | | |
| transportin 3 | 3 | o | o | | | | | | o |
| transposable element p transposase | 3 | o | | | | o | | | |
| transposable element tc1 transposase | 3 | o | | | | | | | |
| tributyltin binding protein type 1b | 3 | | | | o | | | | |
| tricarboxylate transport mitochondrial-like | 3 | | | | o | | | | o |
| tripartite motif-containing 25 | 3 | o | o | | | | | | |
| tripartite motif-containing 63 | 3 | o | | | | | | | |
| triple functional domain (ptprf interacting) | 3 | | | | | | o | o | |
| trk-fused gene | 3 | o | | o | | | o | | |
| trna (cytosine-5-)-methyltransferase nsun2 | 3 | o | | | | | | | |
| trna selenocysteine 1 associated protein 1 | 3 | o | | o | | | | o | |
| tropomodulin 4 | 3 | o | o | | | | | | |
| tropomyosin alpha-4 chain | 3 | o | o | | | | | o | |
| tropomyosin-1 alpha chain | 3 | o | | o | | o | | | |
| trypsin-1 precursor | 3 | | o | | | | | | |
| trypsinogen 3 | 3 | | o | o | | | | | |
| trypsinogen-like protease 1 | 3 | | o | | o | | | | |
| tryptophan -dioxygenase | 3 | | o | o | o | | | | |
| tryptophanyl-trna synthetase | 3 | o | | o | | o | | | |
| tsc22 domain member 3 | 3 | o | o | o | | | | | |
| tubb5 protein | 3 | | | | | | | | o |
| tubulin folding cofactor b | 3 | o | | o | | | | | |
| tubulin polyglutamylase complex subunit 1 | 3 | | | | | | | | o |
| tubulin polyglutamylase complex subunit 2 | 3 | | | | o | | | | o |
| tumor necrosis alpha-induced protein 8-like protein 2 | 3 | o | | | | o | | | |
| tumor rejection antigen 1 | 3 | o | o | | o | | | | |
| tumor suppressor candidate 5 homolog | 3 | | o | | | | | | o |
| type alpha isoform cra_b | 3 | o | | | | | | | |
| tyrosinase | 3 | o | | | | | | | |
| tyrosine phosphatase type iva 2 | 3 | | | | o | o | | o | |
| tyrosine-protein kinase 223-like | 3 | o | o | | | | o | | |
| tyrosine-protein phosphatase non-receptor type 21-like | 3 | | | o | | | o | | o |
| tyrosylprotein sulfotransferase 1 | 3 | o | | o | | | | | |
| u2 snrnp-associated surp motif-containing protein | 3 | | | | | | | | o |
| u2-associated sr140 protein | 3 | o | o | | | o | | | |
| u5 small nuclear ribonucleoprotein 40 kda protein | 3 | o | | o | | | | | o |
| u6 snrna-associated sm-like protein lsm3-like | 3 | | | | | | | | o |
| u6 snrna-associated sm-like protein lsm8 | 3 | o | o | | | | o | | |
| ubiquitin carboxyl-terminal hydrolase 16 | 3 | o | o | o | | | | | |

| | | | | | | | | | |
|---|---|---|---|---|---|---|---|---|---|
| ubiquitin carboxyl-terminal hydrolase 7 | 3 | o | o | | | | o | | |
| ubiquitin carboxyl-terminal hydrolase isozyme l3 | 3 | o | | | | o | | o | |
| ubiquitin protein ligase e3 component n-recognin 1 | 3 | o | o | o | | | | | |
| ubiquitin specific peptidase 25 | 3 | o | o | | | | | | |
| ubiquitin specific peptidase 34 | 3 | o | | | | | o | o | |
| ubiquitin specific peptidase 8 | 3 | o | o | o | | | | | |
| ubiquitin-associated and sh3 domain-containing protein b | 3 | o | | o | | | | o | |
| ubiquitin-conjugating enzyme e2 j1 | 3 | o | | | | | o | | |
| ubiquitin-conjugating enzyme e2 variant 1 | 3 | | o | | | o | | | |
| ubiquitin-conjugating enzyme e2t | 3 | o | | o | | o | | | |
| ubiquitin-like protein 1 | 3 | | | o | | | | o | |
| ubiquitin-related modifier 1 homolog | 3 | o | | | | o | | | o |
| ubx domain protein 4 | 3 | o | | | | o | | | |
| ubx domain-containing protein 11-like | 3 | | | | | | | | o |
| ubx domain-containing protein 7 | 3 | o | | o | | | | | |
| udp-n-acetyl-alpha-d-galactosamine:polypeptide n-acetylgalactosaminyltransferase-like 4 | 3 | o | | | | | o | | |
| udp-n-acetylglucosamine--peptide n-acetylglucosaminyltransferase 110 kda subunit | 3 | o | | | | | o | | o |
| ufm1-specific protease 1 | 3 | o | | o | | o | | | |
| uhrf1-binding protein 1-like | 3 | o | | | o | | o | | |
| ump-cmp kinase | 3 | o | | | | o | o | | |
| unc-45 homolog b ( elegans) | 3 | o | | | | | | | |
| unc-50 homolog | 3 | o | o | o | | | | | |
| uncharacterized protein c11orf87 homolog | 3 | | | | | | | | o |
| uncharacterized protein c9orf85 homolog | 3 | | | | o | | | | o |
| uncharacterized protein dkfzp762i1415-like | 3 | o | | o | | o | | | |
| unconventional prefoldin rpb5 interactor | 3 | | o | o | | | | | |
| unhealthy ribosome biogenesis protein 2 homolog | 3 | o | o | | | | | | o |
| unkempt homolog -like | 3 | o | | | | | o | o | |
| unknown [Sparus aurata] | 3 | | o | | | | | | |
| unnamed protein product [Homo sapiens] | 3 | | o | | | o | o | | |
| unnamed protein product [Mus musculus] | 3 | o | | | | | o | o | o |
| unq655 pro1286 precursor | 3 | o | o | | | o | | | |
| upf0293 protein c16orf42-like | 3 | o | o | o | | | | | |
| upf0308 protein c9orf21 homolog | 3 | o | | | | o | | | |
| upf0368 protein cxorf26-like | 3 | | | | o | | | | o |
| upf0414 transmembrane protein c20orf30 homolog | 3 | o | | o | | o | | | |
| upf0488 protein c8orf33 homolog | 3 | o | | o | | o | | | |
| upf0534 protein c4orf43-like | 3 | o | | o | o | | | | |
| upf0595 protein c22orf40-like | 3 | o | | o | | | | o | |
| upf0609 protein c4orf27 homolog | 3 | | | | o | | | | o |
| upf0663 transmembrane protein c17orf28-like | 3 | | | | | | o | | o |
| upf0739 protein c1orf74 homolog | 3 | | | | o | | | | o |
| up-regulated during skeletal muscle growth protein 5 | 3 | | o | | | | o | | |
| uracil phosphoribosyltransferase homolog | 3 | | o | | | o | | o | |
| uridine phosphorylase 1 | 3 | o | o | | | o | | | |
| uroporphyrinogen iii synthase | 3 | o | | | | | | o | |
| usp39 protein | 3 | o | | o | | | | | |
| uv excision repair protein rad23 homolog b-like | 3 | o | o | | | | o | | |
| vacuolar protein sorting 4b | 3 | o | | | | | | | |

| | | | | | | | | | |
|---|---|---|---|---|---|---|---|---|---|
| vacuolar protein sorting-associated protein 33a | 3 | | | | o | o | | o | |
| vacuolar protein sorting-associated protein 37a | 3 | o | o | | | | o | | |
| vacuolar protein sorting-associated protein 4b | 3 | | o | o | | | | | |
| vacuolar protein sorting-associated protein 72 homolog | 3 | | o | | o | | | | |
| vacuolar-sorting protein snf8 | 3 | o | | | o | | | | o |
| v-akt murine thymoma viral oncogene homolog 2 | 3 | o | | | | | | o | |
| vascular endothelial growth factor receptor 2 | 3 | o | o | | | | | | |
| vasohibin 1 | 3 | o | o | | | | o | | |
| vesicle-associated membrane associated protein b and c | 3 | o | | o | | | | | |
| vesicle-associated membrane protein 3 | 3 | o | o | | | | o | | |
| vesicle-associated membrane protein 7 | 3 | o | | o | | | | o | |
| visinin-like 1 | 3 | | | | | o | o | o | |
| vitellogenic-like | 3 | o | o | | | o | | | |
| vitellogenin c | 3 | | | o | o | | | | |
| voltage-dependent calcium channel gamma-8 subunit | 3 | | | | | | o | | |
| voltage-dependent l-type calcium channel subunit alpha-1d | 3 | | | | o | | | o | |
| voltage-gated sodium channel type iv alpha subunit | 3 | o | | | | | o | | |
| von willebrand factor | 3 | o | o | | | | | | |
| v-type proton atpase catalytic subunit a-like | 3 | | | | o | | | | o |
| was protein member 2 | 3 | o | o | | o | | | | |
| was protein member 3 | 3 | o | | o | | | o | | |
| wd repeat domain 1 | 3 | o | | | | o | | | o |
| wd repeat domain 45 | 3 | o | o | | | | o | | |
| wd repeat domain 46 | 3 | o | | | | | | | |
| wd repeat domain phosphoinositide-interacting protein 3 | 3 | o | | | | o | | o | |
| wdfy family member 4 | 3 | o | o | | | | | | |
| with fg repeats 1 | 3 | | o | | | | o | | |
| wnk lysine deficient protein kinase 2 | 3 | | | | | o | o | | |
| ww domain binding protein 1 | 3 | o | | | | | o | | |
| ww domain-binding protein 2-like | 3 | | | | | | | | o |
| xbp1 protein | 3 | | | | | | | | o |
| xeplin variant 2 | 3 | o | | o | | | | | |
| xeroderma complementation group a | 3 | o | | | | | | | |
| y+l amino acid transporter 1 | 3 | | o | o | o | | | | |
| yeast mon (monensin-resistant) homolog family member (mon-2)-like | 3 | o | | o | | o | | | |
| yippee-like 1 | 3 | | o | | | | | | o |
| yme1-like 1 ( cerevisiae) | 3 | o | o | | | | | o | |
| yth domain member 2 | 3 | o | | | | o | | | |
| zbed1 protein | 3 | o | | | | | | o | |
| zeta (quinone reductase)-like 1 | 3 | | o | | | | | | o |
| zgc:112265 protein | 3 | | o | | | | | | |
| zinc bed-type containing 1 | 3 | o | | | | o | o | | |
| zinc bed-type containing 4 | 3 | o | | o | | | | | |
| zinc c4h2 domain containing | 3 | o | | | | | | | o |
| zinc finger and btb domain containing 33 | 3 | o | | o | | | | o | |
| zinc finger bed domain-containing protein 1-like | 3 | o | | | o | | | | |
| zinc finger cchc domain-containing protein 10 | 3 | o | | o | o | | | | |
| zinc finger e-box-binding homeobox 2 | 3 | | | | | | | | o |
| zinc finger hit domain-containing protein 2 | 3 | o | | | o | | | | |

| | | | | | | | | | |
|---|---|---|---|---|---|---|---|---|---|
| zinc finger homeobox 3-like | 3 | o | | | | | | | |
| zinc finger homeobox protein 4 | 3 | o | | | | | o | | |
| zinc finger matrin-type protein 5 | 3 | o | | o | | o | | | |
| zinc finger protein 131 | 3 | o | o | | | o | | | |
| zinc mym domain containing partial | 3 | o | | | | | | | |
| zinc mym-type 4 | 3 | o | | | | | o | | |
| zinc mym-type 6 | 3 | o | | | o | o | | | |
| zinc transporter | 3 | | o | o | | | | | o |
| zona pellucida protein d | 3 | | | | o | o | | | |
| zpa domain containing protein precursor | 3 | | | | o | | | | |
| zygote arrest 1 | 3 | | | | o | | | | |
| 1300001i01rik protein | 2 | o | | | | | o | | |
| 14-3-3 protein | 2 | | | | o | | | | o |
| 14-3-3 protein gamma-2 | 2 | o | | | | | | | |
| 14-3-3-like protein | 2 | | o | | | | | | o |
| 1a1l1_takru ame: full=1-aminocyclopropane-1-carboxylate synthase-like protein 1 short=acc synthase-like protein 1 | 2 | | o | | o | | | | |
| 1-acylglycerol-3-phosphate o-acyltransferase 6 (lysophosphatidic acid zeta) | 2 | | o | | | o | | | |
| 1-acyl-sn-glycerol-3-phosphate acyltransferase gamma | 2 | | o | | | | | o | |
| 1-aminocyclopropane-1-carboxylate synthase-like protein 1-like | 2 | | | | o | | | | |
| 1-phosphatidylinositol- -bisphosphate phosphodiesterase delta-4 | 2 | o | | | | | | | o |
| 2 -cyclic nucleotide 3 phosphodiesterase | 2 | | o | | | | | | |
| 2310022m17rik protein | 2 | o | | | | o | | | |
| 26s protease regulatory subunit 4 | 2 | | | | | o | | o | |
| 26s proteasome non-atpase regulatory subunit 12 | 2 | | | | o | | | | o |
| 26s proteasome non-atpase regulatory subunit 3-like | 2 | | | | | | | | o |
| 26s proteasome non-atpase regulatory subunit 5-like | 2 | | | | | | | | o |
| 26s proteasome non-atpase regulatory subunit 7-like | 2 | | | | o | | | | |
| 26s proteasome non-atpase regulatory subunit 9 | 2 | | | o | | | | o | |
| 2-amino-3-carboxymuconate-6-semialdehyde decarboxylase | 2 | | o | o | | | | | |
| 2-hydroxyacylsphingosine 1-beta-galactosyltransferase precursor | 2 | o | | o | | | | | |
| 2-oxo-4-hydroxy-4-carboxy-5-ureidoimidazoline decarboxylase | 2 | | o | o | | | | | |
| 2-oxoglutarate and iron-dependent oxygenase domain-containing protein 1 | 2 | o | | | | | | | |
| 3-hydroxyacyl- dehydrogenase type-2 | 2 | | o | | | | | | o |
| 3-keto-steroid reductase-like | 2 | | o | | o | | | | |
| 40s ribosomal protein s16 | 2 | | | | o | | | | o |
| 40s ribosomal protein s3 | 2 | | | | | | o | o | |
| 4f2 cell-surface antigen heavy chain-like | 2 | | | | o | | | | |
| 5 - cytosolic ii | 2 | o | | | | | | | |
| 5 - cytosolic iii | 2 | | | o | | | | o | |
| 5-aminolevulinate erythroid- mitochondrial precursor | 2 | o | | | | | | | |
| 5-amp-activated protein kinase subunit beta-1 | 2 | | o | o | | | | | |
| 5-azacytidine induced 1 | 2 | o | | | | o | | | |
| 5ht3 receptor | 2 | o | | | | | | | |
| 60s ribosomal protein l13 | 2 | | o | | o | | | | |

| | | | | | | | | | |
|---|---|---|---|---|---|---|---|---|---|
| 60s ribosomal protein l23a | 2 | | | | o | | | | |
| 60s ribosomal protein l26 | 2 | | | | o | | | | |
| 60s ribosomal protein l36a-like | 2 | | | | o | | | | o |
| 60s ribosomal protein l6 | 2 | | | | o | | | | o |
| 60s ribosomal protein l8 | 2 | | | | o | | | | o |
| 6-phosphofructokinase type c-like | 2 | | | | | o | | o | |
| 6-phosphogluconolactonase-like | 2 | | | | | | | | o |
| a kinase anchor protein 11 | 2 | o | | | | | o | | |
| a kinase anchor protein 12 | 2 | o | | | | | | o | |
| a kinase anchor protein 2 | 2 | o | | o | | | | | |
| a kinase anchor protein 6 | 2 | o | | | | | | | |
| aars protein | 2 | | | | | | | | o |
| abc-transporter subfamily-b type 3 | 2 | o | | o | | | | | |
| abhydrolase domain containing 6 | 2 | | o | | | | | | |
| abhydrolase domain containing 7 | 2 | | | | | | o | | |
| abhydrolase domain-containing protein 2-like | 2 | | | | | | o | | |
| abhydrolase domain-containing protein fam108c1 | 2 | | | o | | | | | |
| abl interactor 2 | 2 | o | o | | | | | | |
| abl-interactor 1 | 2 | o | | | | o | | | |
| ac transposable element derived partial | 2 | o | | | | | | | |
| ac transposable element-derived protein 4 | 2 | | | | | o | o | | |
| acetolactate synthase-like protein | 2 | | o | | | | | | |
| acetyl- carboxylase | 2 | | o | o | | | | | |
| acetyl- carboxylase alpha | 2 | | | | | | o | | |
| acetyl- cytosolic | 2 | o | o | | | | | | |
| acetylcholine receptor subunit alpha precursor | 2 | o | | | | | | | |
| acid phosphatase lysosomal | 2 | o | | | | | | | |
| acid trehalase-like protein 1-like | 2 | | | | | | | | o |
| acidic (leucine-rich) nuclear phosphoprotein 32 member a | 2 | o | | | | | | o | |
| acidic (leucine-rich) nuclear phosphoprotein 32 member b | 2 | o | | | | o | | | |
| acidic coiled-coil containing protein 1 | 2 | | | | | | o | | |
| acidic coiled-coil containing protein 2 | 2 | | | | | | o | | |
| acidic leucine-rich nuclear phosphoprotein 32 family member b | 2 | | o | | | | | o | |
| acidic repeat-containing | 2 | | | | o | | | | |
| actin associated protein | 2 | o | | | o | | | | |
| actin binding 1a | 2 | | | o | | o | | | |
| actin binding 1b | 2 | o | | | | | | | |
| actin binding protein 6 | 2 | o | | | | | | | o |
| actin filament-associated protein 1-like 1 | 2 | o | | o | | | | | |
| actin-related protein 10 | 2 | | o | | | | | | |
| actin-related protein 2 | 2 | o | | | | | | | o |
| actin-related protein 6 | 2 | o | o | | | | | | |
| activated leukocyte cell adhesion molecule | 2 | | | | | o | o | | |
| activating transcription factor 3 | 2 | o | | | | | | | |
| activating transcription factor 4 (tax-responsive enhancer element b67) | 2 | | | o | | | | | o |
| activating transcription factor 7-interacting protein 1-like | 2 | | o | | | | | | |
| activator of basal transcription 1 | 2 | o | | | | o | | | |
| activity-dependent neuroprotector homeobox protein | 2 | | | | o | | o | | |
| acyl- :lysophosphatidylglycerol acyltransferase 1 | 2 | | | | | | o | o | |
| acyl- synthetase long-chain family member 4 | 2 | | o | | | | | | |
| acyl- synthetase long-chain family member 6 | 2 | | o | | | | o | | |

| | | | | | | | | | |
|---|---|---|---|---|---|---|---|---|---|
| acylamino-acid-releasing enzyme | 2 | o | o | | | | | | |
| acyl-coenzyme a dehydrogenase member 9 | 2 | o | o | | | | | | |
| acyl-coenzyme a long chain | 2 | o | o | | | | | | |
| acyl-coenzyme a oxidase isoform cra_b | 2 | o | | o | | | | | |
| acyl-coenzyme a oxidase palmitoyl | 2 | | o | o | | | | | |
| acylglycerol mitochondrial precursor | 2 | | o | o | | | | | |
| adam metallopeptidase with thrombospondin type 1 1 | 2 | o | | | | | | | |
| adam metallopeptidase with thrombospondin type 1 15 | 2 | o | | | | | | | |
| adaptor-related protein complex alpha 1 subunit | 2 | | o | | | | o | | |
| adaptor-related protein complex mu 2 subunit | 2 | | o | | | | o | | |
| adenosine 3 -phospho 5 -phosphosulfate transporter 2 | 2 | | | | o | | | | |
| adenosine deaminase | 2 | o | | | | | | o | |
| adenosine kinase a | 2 | | o | o | | | | | |
| adenosylmethionine decarboxylase 1 | 2 | o | | | | | o | | |
| adenylate cyclase-associated 2 | 2 | o | | | | | | | |
| adenylosuccinate lyase | 2 | o | | | | | | | |
| adenylosuccinate synthetase isozyme 1 | 2 | o | | | | | | | |
| adhesion molecule with ig like domain 1 | 2 | | | | | | o | | |
| adiponectin receptor 2 | 2 | | o | | | | | | |
| adnp homeobox protein 2-like | 2 | | | | o | | o | | |
| adp-dependent glucokinase | 2 | | o | | | | | | |
| adp-ribosylation factor guanine nucleotide-exchange factor 2 (brefeldin a-inhibited) | 2 | o | | o | | | | | |
| adp-ribosylation factor interacting protein 2 | 2 | | | | | | o | o | |
| adp-ribosylation factor-like 1 | 2 | o | | o | | | | | |
| adp-ribosylation factor-like 14 | 2 | o | o | | | | | | |
| adp-ribosylation factor-like 16 | 2 | o | | | | o | | | |
| adp-ribosylation factor-like 3 | 2 | o | o | | | | | | |
| adp-ribosylation factor-like 5a | 2 | o | | | | | | | |
| adp-ribosylation factor-like 6 interacting protein 1 | 2 | o | | | | | | o | |
| adp-ribosylation factor-like 9 | 2 | o | | | | | | | |
| adp-ribosylation factor-like protein 2 | 2 | | | | o | | | | |
| adp-ribosylation factor-like protein 6-interacting protein 4 | 2 | o | | | | o | | | |
| adp-ribosylation-like factor 6 interacting protein 6 | 2 | o | | | | o | | | |
| adrenomedullin 1 | 2 | o | | o | | | | | |
| af363273_1 toxin-1 | 2 | | o | | | | | | |
| agouti-related protein | 2 | | | | | o | | | o |
| agrin | 2 | | | | | | o | | |
| a-kinase anchor protein 11-like | 2 | | | | | | o | | |
| akt interacting protein | 2 | o | | | | | | | |
| alanine-glyoxylate aminotransferase 2 | 2 | | o | o | | | | | |
| aldehyde dehydrogenase 1 member l2 | 2 | o | o | | | | | | |
| aldo keto reductase | 2 | | | | | | | | o |
| aldo-keto reductase family 1 member b10-like | 2 | | o | | | | | | o |
| alkaline phosphatase | 2 | | | | | o | | | o |
| alkylation repair homolog 1 ( coli) | 2 | | | o | | | | o | |
| alkylation repair homolog 3 | 2 | o | | | | o | | | |
| alkylation repair homolog 4 ( coli) | 2 | o | o | | | | | | |
| allantoicase | 2 | | o | | | | | | |
| allograft inflammatory factor 1 | 2 | | | o | | | | o | |
| alpha-mannosyl-glycoprotein 4-beta-n-acetylglucosaminyltransferase c | 2 | | o | o | | | | | |
| alpha-sialyltransferase st6 c v | 2 | | | | | | | o | o |

| | | | | | | | | | |
|---|---|---|---|---|---|---|---|---|---|
| alpha-sialyltransferase st8sia vi | 2 | o | o | | | | | | |
| alpha-fucosyltransferase | 2 | o | | | | | o | | |
| alpha-2-hs-glycoprotein | 2 | | o | | | | | | |
| alpha-2-macroglobulin-like isoform 1 | 2 | | o | | | | | | |
| alpha-aspartyl dipeptidase | 2 | o | | o | | | | | |
| alpha-kinase 2 | 2 | o | | | | | | | |
| alpha-l- plasma | 2 | | o | | | | o | | |
| alpha-methylacyl- racemase | 2 | o | o | | | | | | |
| alpha-soluble nsf attachment protein | 2 | | | | | o | o | | |
| amine oxidase (flavin containing) domain 1 | 2 | o | | | | | | | |
| aminophospholipid transporter- class type member 2 | 2 | | | | | | o | | |
| amp deaminase 2 | 2 | | | | | | o | | o |
| amp-activated protein kinase gamma2 subunit-like | 2 | o | | | | | | | |
| amp-activated protein non-catalytic gamma-3 subunit | 2 | o | | | | | | | |
| amyloid beta a4 precursor protein- family member 2 isoform 4 | 2 | o | | | | | | | |
| amyloid beta precursor protein b | 2 | o | | | | o | | | |
| amyloid beta precursor protein- family member 1 | 2 | o | | | | | o | | |
| amyloid-like protein 2 | 2 | o | | | | | | o | |
| amyloid-like protein 2-like | 2 | | | | | | o | | o |
| an1-type zinc finger protein 2b | 2 | o | | o | | | | | |
| an1-type zinc finger protein 5 | 2 | o | | | | | | | |
| anaphase-promoting complex subunit 11 | 2 | o | | | o | | | | |
| angiogenic factor vg5q | 2 | | o | | | o | | | |
| angiogenic factor with g patch and fha domains 1 | 2 | o | | | | | | o | |
| angiopoietin-like 1 | 2 | o | | | | | | | |
| angiopoietin-like 3 | 2 | | o | | | | | | |
| angiopoietin-like 4 | 2 | o | | | o | | | | |
| ankyrin repeat and fyve domain containing 1 | 2 | | o | o | | | | | |
| ankyrin repeat and fyve domain-containing protein 1 | 2 | | | | | | | | o |
| ankyrin repeat and kh domain-containing protein 1 | 2 | | o | | | | | | |
| ankyrin repeat and mynd domain-containing protein 2 | 2 | o | | | | | | | |
| ankyrin repeat and sam domain-containing protein 3 | 2 | | o | o | | | | | |
| ankyrin repeat and socs box-containing 10-like | 2 | o | | | | | | | |
| ankyrin repeat and socs box-containing 12 | 2 | o | | | | | | | |
| ankyrin repeat and socs box-containing 2 | 2 | o | | | | | | | |
| ankyrin repeat domain 10 | 2 | o | | | | | | | |
| ankyrin repeat domain 12 | 2 | | | | o | | o | | |
| ankyrin repeat domain 12 isoform 2 | 2 | o | | | | | o | | |
| ankyrin repeat domain 29 | 2 | | | | | o | | o | |
| ankyrin repeat domain-containing protein 50-like | 2 | | | | o | | | | |
| ankyrin repeat-containing cofactor-1 | 2 | | | | | | o | o | |
| anoctamin 5 | 2 | o | | | | | | | |
| anoctamin calcium activated chloride channel | 2 | o | | | | o | | | |
| anoctamin-3 isoform 1 | 2 | o | | | | | o | | |
| antigen precursor | 2 | | o | | | o | | | |
| antigenic determinant of reca protein homolog | 2 | | o | o | | | | | |
| antithrombin iii | 2 | | o | | | | | | |
| ap-1 complex subunit mu-1 | 2 | | | o | | | | | o |

| | | | | | | | | | |
|---|---|---|---|---|---|---|---|---|---|
| ap-1 complex subunit sigma-3 | 2 | | o | | | | | | |
| ap-2 complex subunit beta | 2 | | | | | | o | | |
| ap-3 complex subunit sigma-2 | 2 | | | | | | | o | o |
| ap-4 complex subunit mu-1 | 2 | | o | | | o | | | |
| apolipoprotein a1 binding protein | 2 | o | | | | o | | | |
| apolipoprotein h (beta-2-glycoprotein i) | 2 | | | o | | | | | |
| apolipoprotein m | 2 | | o | o | | | | | |
| apolipoprotein o | 2 | o | o | | | | | | |
| apoptosis-enhancing nuclease-like | 2 | | | | o | | | | |
| aquaporin 12 | 2 | | o | o | | | | | |
| aquaporin 9 | 2 | | o | | | | | | |
| arachidonate 12-lipoxygenase | 2 | | | | o | | | | |
| arc cg6741- partial | 2 | o | | | | | | | |
| arginine serine-rich protein pnisr-like | 2 | | | | o | | | | |
| ariadne homolog 2 | 2 | o | | | | | | | |
| arm-1 protein variant 1 | 2 | | | | o | | | | o |
| armadillo repeat containing 6 | 2 | o | o | | | | | | |
| armadillo repeat-containing protein 1 | 2 | o | | | o | | | | |
| armadillo repeat-containing protein 8 | 2 | | | | | | o | | |
| arp1 actin-related protein 1 homolog centractin alpha | 2 | | | o | | | | o | |
| arp2 actin-related protein 2 homolog | 2 | o | | | | | | | o |
| arp3 actin-related protein 3 homolog | 2 | o | | | | | | | |
| aryl hydrocarbon receptor 2 | 2 | o | | | | | o | | |
| aryl hydrocarbon receptor nuclear translocator | 2 | | | | o | | | | |
| arylsulfatase a-like | 2 | | o | | | | | | o |
| arylsulfatase d | 2 | | o | | | | | o | |
| asc-type amino acid transporter 1 | 2 | o | | | | | | o | |
| asialoglycoprotein receptor 1 | 2 | | o | | o | | | | |
| asparagine synthetase | 2 | o | | o | | | | | |
| asparagine synthetase domain-containing protein 1 | 2 | o | | | | o | | | |
| asparagine-linked glycosylation 3 homolog ( alpha- -mannosyltransferase) | 2 | o | | o | | | | | |
| asparagine-linked glycosylation protein 11 homolog | 2 | | o | | | | | | |
| aspartate cytoplasmic-like | 2 | | | | | | | | o |
| aspartoacylase 3 | 2 | o | | o | | | | | |
| aspartyl-trna cytoplasmic | 2 | | | | o | | | | o |
| at rich interactive domain 2 ( rfx-like) | 2 | o | | | | | o | | |
| ataxin 1 | 2 | o | | | | | o | | |
| ataxin 7-like 3 | 2 | o | | | | | | | |
| atg10 autophagy related 10 homolog ( cerevisiae) | 2 | o | | | | | | | |
| atg12 autophagy related 12 homolog ( cerevisiae) | 2 | o | | | | o | | | |
| atg2 autophagy related 2 homolog a | 2 | o | o | | | | | | |
| atp synthase mitochondrial f1 complex assembly factor 1 | 2 | o | | | | o | | | |
| atp synthase mitochondrial f1 complex assembly factor 1-like | 2 | | | | | | | | o |
| atp2b1a protein | 2 | | | o | | o | | | |
| atpase aaa domain containing 4 | 2 | o | | o | | | | | |
| atpase type 13a1 | 2 | o | o | | | | | | |
| atp-binding cassette sub-family a member 12 | 2 | | | | | | | | o |
| atp-binding cassette sub-family g member 2 | 2 | | o | | | | | | |
| atp-binding sub-family c (cftr mrp) member 12 | 2 | | o | | | | | | |
| atp-binding sub-family f member 3 | 2 | | o | | | | | | |

| | | | | | | | | | |
|---|---|---|---|---|---|---|---|---|---|
| atp-citrate synthase | 2 | | o | | | | | | |
| atp-dependent dna helicase pif1 | 2 | | | | o | | | | |
| atp-dependent metalloprotease yme1l1 | 2 | | o | | | | | | |
| atp-dependent rna helicase ddx42 | 2 | | | o | | | | o | |
| atp-dependent rna helicase dhx30 | 2 | | o | | | | | | o |
| atp-sensitive inward rectifier potassium channel 12-like | 2 | | | | | | | | o |
| at-rich interactive domain-containing protein 2 | 2 | | | | | | o | | |
| au rna binding protein enoyl-coenzyme a hydratase | 2 | | o | | | o | | | |
| aurora kinase b | 2 | o | | | o | | | | |
| autocrine motility factor receptor | 2 | | o | | | | | | |
| autophagy-related protein 2 homolog b | 2 | o | | | | | o | | |
| autophagy-related protein 3 | 2 | o | o | | | | | | |
| autotransporter-associated beta strand repeat protein | 2 | o | | | | | | | |
| b chain crystallographic and kinetic studies of human mitochondrial acetoacetyl-coa thiolase : the importance of potassium and chloride for its structure and function | 2 | | | | | | | | o |
| b chain structural basis for formation and hydrolysis of calcium messenger cyclic adp-ribose by human cd38 | 2 | o | | | | o | | | |
| b2 bradykinin receptor | 2 | o | | | | | | | |
| b9 domain-containing protein 2 | 2 | | | o | | | | | |
| baculoviral iap repeat-containing protein 5 | 2 | o | | | o | | | | |
| band -like protein 3 | 2 | | | | | | o | | |
| band -like protein 3-like | 2 | | o | | o | | | | |
| band -like protein 4b-like | 2 | | | | | | | | o |
| bardet-biedl syndrome 2 protein | 2 | | | | o | | | | |
| basal cell adhesion molecule | 2 | | | o | | | o | | |
| basement membrane-specific heparan sulfate proteoglycan core | 2 | | | | o | | | | |
| basic leucine zipper transcriptional factor atf-like 3 | 2 | o | | | | | | | |
| bc016423 protein | 2 | o | | o | | | | | |
| b-cell cll lymphoma 7 protein family member b | 2 | | | o | | | | | o |
| b-cell lymphoma 6 protein | 2 | o | o | | | | | | |
| b-cell receptor cd22-like | 2 | | o | | | o | | | |
| b-cell translocation gene 3 | 2 | o | | o | | | | | |
| b-cell translocation protein 1 | 2 | | | o | | | | o | |
| bcl2 adenovirus e1b interacting protein 3-like | 2 | o | o | | | | | | |
| bcl6 co-repressor-like 1 | 2 | o | | | | | | o | |
| bdnf nt-3 growth factors receptor | 2 | | | | | | o | | |
| bernardinelli-seip congenital lipodystrophy 2 homolog isoform cra_a | 2 | o | | | | | | | |
| beta-glucuronyltransferase 1 (glucuronosyltransferase p) isoform cra_a | 2 | o | | | | | | | |
| beta lysosomal | 2 | | o | | | | | | |
| beta-adrenergic receptor kinase 2 | 2 | o | | | | | o | | |
| beta-galactoside alpha -sialyltransferase st6gal i | 2 | | | | | | o | | |
| beta-hexosaminidase subunit alpha | 2 | o | | | | o | | | |
| beta-ureidopropionase | 2 | | o | | | | | | o |
| bicaudal d homolog 1 | 2 | o | | | | | | o | |
| bifunctional 3 -phosphoadenosine 5 -phosphosulfate synthase 1 | 2 | o | | | | | | | o |
| bifunctional methylenetetrahydrofolate dehydrogenase mitochondrial precursor | 2 | o | | | o | | | | |

| | | | | | | | | | |
|---|---|---|---|---|---|---|---|---|---|
| bifunctional udp-n-acetylglucosamine 2-epimerase n-acetylmannosamine kinase | 2 | o | | o | | | | | |
| bile salt export pump | 2 | | o | | | | | | |
| biliary glycoprotein | 2 | o | | | o | | | | |
| bisphosphoglycerate mutase | 2 | o | | | | | | | |
| bmp-2 inducible kinase | 2 | o | o | | | | | | |
| bone morphogenetic protein 15 | 2 | | | | o | | | | |
| brain and reproductive organ-expressed (tnfrsf1a modulator) | 2 | o | | | | | | | |
| brca1-a complex subunit bre | 2 | | o | | | o | | | |
| brca1-a complex subunit merit40 | 2 | o | | | | o | | | |
| breast cancer metastasis-suppressor 1-like | 2 | | o | | | | | o | |
| brefeldin resistant arf-gef 2b | 2 | | | o | | | | o | |
| bridging integrator 3 | 2 | o | | | | | | | |
| bromodomain adjacent to zinc finger 2b | 2 | | | | | | | o | o |
| bromodomain and phd finger 3 | 2 | o | | | | | | | |
| bromodomain containing 3 | 2 | o | | | | | | | |
| bsg protein | 2 | | | | o | | | | |
| btb poz domain-containing protein 10 | 2 | o | | | | | | | o |
| btb poz domain-containing protein kctd6 | 2 | o | | o | | | | | |
| butyrate response factor 1 | 2 | o | o | | | | | | |
| c11orf46 homolog | 2 | o | | o | | | | | |
| c12orf65 homolog | 2 | o | | o | | | | | |
| c13orf22 homolog ( sapiens) like | 2 | | | | | | | o | o |
| c1orf31 | 2 | o | | | | o | | | |
| c1r c1s subunit of ca2+-dependent complex precursor | 2 | | | | o | | | | |
| c219-reactive peptide | 2 | o | | | | | | | |
| c6orf115 | 2 | | o | | | | o | | |
| c6orf130 homolog | 2 | | | | | | o | | |
| c8orf4 homolog | 2 | | o | | | | | o | |
| ca++ cardiac fast twitch 1 | 2 | o | | | | | | | |
| ca++ cardiac slow twitch 2a | 2 | o | | | | | | o | |
| caax alpha | 2 | o | | | | | | | o |
| caax prenyl protease 1 homolog | 2 | | | | | | o | o | |
| cadherin 5 | 2 | o | | | | | | | |
| cadherin type 1 preproprotein | 2 | | | | | | o | o | |
| calcineurin b homologous protein 2 | 2 | o | | | | | | | |
| calcineurin subunit b type 1-like | 2 | | | | | | o | | |
| calcitonin receptor | 2 | | | | | | o | | |
| calcium activated nucleotidase 1 | 2 | | o | o | | | | | |
| calcium activated nucleotidase isoform cra_b | 2 | o | | | | | | | |
| calcium binding and coiled-coil domain 2 | 2 | o | | | | | | | |
| calcium calmodulin-dependent protein kinase ( kinase) ii gamma | 2 | o | | | | | | | |
| calcium calmodulin-dependent serine protein kinase (maguk family) | 2 | | | | | | o | o | |
| calcium voltage- gamma subunit 6 | 2 | o | | | | | | | |
| calcium voltage- l alpha 1c subunit | 2 | | | | | o | | o | |
| calcium voltage- l alpha 1d subunit | 2 | | | | | | o | o | |
| calcium-binding mitochondrial carrier protein aralar1 | 2 | o | | | | | | | o |
| calmodulin binding protein 3 | 2 | | o | | | | | | |
| calmodulin regulated spectrin-associated protein 1 | 2 | o | | | | | o | | |
| calmodulin-like | 2 | | | | | | o | | |
| calmodulin-regulated spectrin-associated protein 3 | 2 | | | | | | o | | |
| calpain 5 | 2 | o | o | | | | | | |
| calpain-1 catalytic subunit | 2 | | o | | | | | | o |

| | | | | | | | | | |
|---|---|---|---|---|---|---|---|---|---|
| calpain-2 catalytic subunit | 2 | | | | o | | | | |
| calpain-like protein | 2 | | | | o | | | | |
| calponin 2 | 2 | o | | o | | | | | |
| calponin smooth muscle | 2 | o | | | | | o | | |
| calreticulin precursor | 2 | o | | | o | | | | |
| calsyntenin 3 | 2 | | | | | | | o | |
| calumenin isoform 1 | 2 | o | o | | | | | | |
| camp responsive element binding protein 3-like 1 | 2 | o | | | | | o | | |
| camp responsive element binding protein 3-like 3 | 2 | | o | o | | | | | |
| camp-dependent protein kinase type i-beta regulatory subunit-like | 2 | | | | | | | | o |
| camp-dependent protein kinase type ii-alpha regulatory subunit | 2 | | o | | | | | | |
| camsap1l1 protein | 2 | | | | | o | o | | |
| canopy 4 homolog | 2 | o | | | o | | | | |
| capz-interacting protein | 2 | | | | o | | | | |
| carbohydrate sulfotransferase 15 | 2 | o | | | | | | | o |
| carbohydrate sulfotransferase 2 | 2 | o | | | | o | | | |
| carbonic anhydrase mitochondrial | 2 | o | | | | | o | | |
| carboxyl ester lipase (bile salt-stimulated lipase) | 2 | | o | o | | | | | |
| carboxypeptidase a1 | 2 | o | o | | | | | | |
| carboxypeptidase b | 2 | | o | | | | | | |
| carboxypeptidase b2-like | 2 | | | | o | | | | |
| carboxypeptidase polypeptide 2 | 2 | | o | | | | | | |
| carboxypeptidase z | 2 | o | | | | | | | |
| cardiomyopathy associated 1 | 2 | o | | | | | | | |
| -carotene 9 -oxygenase-like | 2 | | | | o | | | | o |
| carotene- -monooxygenase | 2 | o | | | | | | | |
| cartilage intermediate layer protein 2-like | 2 | o | | | | | | | |
| cas hef-like protein 1 | 2 | o | | | | | | | |
| casein kinase delta | 2 | o | o | | | | | | |
| caseinolytic atp- proteolytic subunit homolog ( coli) | 2 | | o | | | | o | | |
| cat eye syndrome chromosome candidate 5 homolog | 2 | | o | | | | | | |
| catenin (cadherin-associated protein) alpha 2 | 2 | | | | | | o | | o |
| catenin (cadherin-associated protein) alpha-like 1 | 2 | | o | o | | | | | |
| catenin (cadherin-associated protein) delta 2 (neural plakophilin-related arm-repeat protein) | 2 | | | | | | o | | |
| catenin alpha-1-like | 2 | | o | | | | o | | |
| cathepsin s | 2 | o | | | | o | | | |
| cation diffusion facilitator family transporter | 2 | | o | | o | | | | |
| cation transport regulator homolog 2 ( coli) | 2 | o | o | | | | | | |
| cation-independent mannose-6-phosphate receptor | 2 | o | | | | | | | |
| cbf1 interacting corepressor | 2 | o | | o | | | | | |
| cc chemokine | 2 | o | | | | | | | |
| c-c motif chemokine 4 precursor | 2 | | | o | | | | o | |
| ccar1 protein | 2 | o | | | | | | | |
| ccr4-not transcription complex subunit 1 | 2 | o | | | | | o | | |
| ccr4-not transcription complex subunit 7 | 2 | o | o | | | | | | |
| ccr4-not transcription subunit 2 | 2 | o | | | | | | | |
| ccr4-not transcription subunit 8 | 2 | o | | o | | | | | |
| cd151 antigen | 2 | o | | | | | | | |
| cd2 antigen cytoplasmic tail-binding protein 2 | 2 | o | | | | o | | | |

| | | | | | | | | | |
|---|---|---|---|---|---|---|---|---|---|
| cd209 antigen-like protein e | 2 | | | | | | o | | o |
| cd226 antigen precursor | 2 | | | | o | | | | o |
| cd3 gamma delta | 2 | o | | | o | | | | |
| cd302 antigen precursor | 2 | o | | o | | | | | |
| cd36 antigen | 2 | | o | | | | o | | |
| cd46 complement regulatory | 2 | o | | | | | | | |
| cd59 glyco | 2 | | | | o | | | | o |
| cd82 antigen | 2 | o | | | | | | | o |
| cell adhesion molecule 1 | 2 | | | | | | o | o | |
| cell adhesion molecule 1-like | 2 | | | | o | | | | |
| cell adhesion molecule 2 | 2 | | | | | | o | o | |
| cell adhesion molecule 3 isoform 1 | 2 | o | | | | o | | | |
| cell cycle progression protein 1-like | 2 | | | | | | | | o |
| cell division cycle 20 homolog ( cerevisiae) | 2 | o | | | o | | | | |
| cell division cycle 34 | 2 | o | | | | | | | |
| cell division cycle 40 homolog | 2 | o | | | | o | | | |
| cell division cycle 40 homolog ( cerevisiae) | 2 | o | o | | | | | | |
| cell division cycle associated 7 | 2 | o | | | | o | | | |
| cell division cycle associated 8 | 2 | | o | | o | | | | |
| cell division cycle protein 123 homolog | 2 | o | | | o | | | | |
| cell division cycle protein 27 homolog | 2 | o | | | | o | | | |
| cell division protein kinase 4 | 2 | o | | o | | | | | |
| cellular repressor of e1a-stimulated genes | 2 | | | o | | | | o | |
| centrin 2 | 2 | | | | | | | o | o |
| centromere protein h | 2 | | | | o | | | | |
| centrosomal protein 170kda | 2 | o | | | | | o | | |
| centrosomal protein of 164 kda | 2 | | | | o | | | | |
| centrosomal protein of 192 kda-like | 2 | o | | | | | | o | |
| centrosomal protein of 55 kda | 2 | | | | o | | | | |
| cerebellar degeneration-related protein 2-like | 2 | | | | | | | | o |
| cerebellin 1 precursor | 2 | | | | | o | | o | |
| ceroid- neuronal late variant | 2 | o | | | | | | | |
| cfh protein | 2 | | | o | | | | | |
| c-fos protein | 2 | o | | | | | o | | |
| cgmp-dependent 3 -cyclic phosphodiesterase | 2 | | | | | | o | | |
| chaf1a protein | 2 | | | | o | o | | | |
| chaperone activity of bc1 complex-mitochondrial | 2 | o | | | | | | | |
| chaperone activity of bc1 complex-mitochondrial precursor | 2 | o | o | | | | | | |
| chaperone activity of bc1 complex-mitochondrial-like | 2 | | o | | | | | | o |
| charged multivesicular body protein 1b | 2 | o | | o | | | | | |
| charged multivesicular body protein 4b | 2 | o | o | | | | | | |
| checkpoint protein hus1 | 2 | o | o | | | | | | |
| chemokine (c-x-c motif) ligand 6 | 2 | | | o | | o | | | |
| chemokine-like receptor 1 | 2 | o | | | | | | | |
| chemokine-like receptor 1-like | 2 | | | | o | | | | o |
| chimerin 1 | 2 | | | | | o | | o | |
| chloride channel 3 | 2 | | o | | | | o | | |
| chloride channel protein 2-like | 2 | | | | | | o | | |
| chloride intracellular channel 5 | 2 | o | o | | | | | | |
| chloride intracellular channel protein 2 | 2 | o | | o | | | | | |
| chm protein | 2 | o | | | | | | | |
| chmp member 7 | 2 | o | | | | | | o | |
| cholecystokinin | 2 | | | | | o | | o | |
| cholesterol 24-hydroxylase-like | 2 | | o | | | | | | |
| cholesteryl ester transfer plasma | 2 | | o | | o | | | | |
| cholesteryl ester transfer protein | 2 | | o | o | | | | | |
| choline ethanolamine phosphotransferase 1 | 2 | o | o | | | | | | |

| | | | | | | | | | |
|---|---|---|---|---|---|---|---|---|---|
| choline ethanolaminephosphotransferase 1 | 2 | | | | | | | | o |
| chondroitin sulfate proteoglycan 5 | 2 | | | | | | o | o | |
| choriogenin h minor | 2 | | | | o | | | | |
| chromatin accessibility complex 1 | 2 | o | | o | | | | | |
| chromodomain helicase dna binding protein 2 | 2 | o | | | | | | | |
| chromodomain helicase dna binding protein 4 | 2 | | o | | | | o | | |
| chromodomain helicase dna binding protein 5 | 2 | | | | | | o | | |
| chromodomain helicase dna binding protein 8 | 2 | | | | | o | | | |
| chromosome 1 open reading frame 57 | 2 | o | | o | | | | | |
| chromosome 1 open reading frame 9 | 2 | o | o | | | | | | |
| chromosome 10 open reading frame 65 | 2 | | o | | | | | | |
| chromosome 12 open reading frame 23 | 2 | | o | o | | | | | |
| chromosome 14 open reading frame 133 | 2 | o | | | | | | | |
| chromosome 15 open reading frame 29 | 2 | o | | | | | | | |
| chromosome 15 open reading frame 43 | 2 | | o | | | | | o | |
| chromosome 16 open reading frame 7 | 2 | o | o | | | | | | |
| chromosome 17 open reading frame 37 | 2 | o | | o | | | | | |
| chromosome 18 open reading frame 45 | 2 | o | o | | | | | | |
| chromosome 19 open reading frame 29 | 2 | | o | | | o | | | |
| chromosome 19 open reading frame 50 | 2 | o | | | | o | | | |
| chromosome 19 open reading frame 52 | 2 | o | | o | | | | | |
| chromosome 20 open reading frame 54 | 2 | o | | | | | | | |
| chromosome 21 open reading frame 2 | 2 | o | | | | o | | | |
| chromosome 5 open reading frame 15 | 2 | o | | o | | | | | |
| chromosome 5 open reading frame 24 | 2 | o | | | | | | o | |
| chromosome 6 open reading frame 130 | 2 | o | | | | o | | | |
| chromosome 9 open reading frame 114 | 2 | o | o | | | | | | |
| chromosome 9 open reading frame 3 | 2 | o | | | | | | | |
| chromosome transmission fidelity protein 8 homolog | 2 | o | | | o | | | | |
| churchill protein | 2 | | | | | | | o | |
| ciliary rootlet coiled- rootletin | 2 | | | | | o | | | o |
| c-jun protein | 2 | o | o | | | | | | |
| class e basic helix-loop-helix protein 40 | 2 | o | | | | | | | |
| class member 1 | 2 | | o | | | o | | | |
| class member 2 | 2 | | o | | | | | | |
| class type 11c | 2 | o | | | | | o | | |
| class type 9a | 2 | o | o | | | | | | |
| clathrin coat assembly protein ap180 | 2 | | | | | | o | | o |
| clathrin heavy chain 1-like | 2 | | | | | | o | | o |
| clathrin interactor 1 | 2 | o | | o | | | | | |
| clathrin light chain b | 2 | o | | | | | o | | |
| claudin 7 | 2 | | | | o | | | | |
| claudin domain containing 1 | 2 | o | | | | | | | o |
| claudin gene 1 | 2 | | | | o | | | | |
| claudin-like protein zf4a22 | 2 | | | | o | | | | |
| cleavage and polyadenylation specificity factor subunit 5 | 2 | o | | o | | | | | |
| cleavage stimulation factor subunit 3 | 2 | o | | | | | | | o |
| clrn2 protein | 2 | o | | | | | | | |
| cmp-n-acetylneuraminate-beta-galactosamide-alpha- -sialyltransferase 4 isoform 1 | 2 | | o | | | | | | |
| cmp-n-acetylneuraminate-beta-galactosamide-alpha- -sialyltransferase 4-like | 2 | | | | | | | | o |
| c-myc promoter-binding protein | 2 | | | | | | o | | |
| coactivator-associated arginine methyltransferase 1 | 2 | o | | | | | | | |
| coagulation factor ix precursor | 2 | | | | o | | | | o |
| coagulation factor viii | 2 | o | o | | | | | | |
| coagulation factor x-like | 2 | | | | o | | | | |

| | | | | | | | | | |
|---|---|---|---|---|---|---|---|---|---|
| coatomer protein subunit gamma 2 | 2 | | | o | | | | o | |
| coatomer subunit beta -like | 2 | | | | | | o | | o |
| coatomer subunit epsilon-like | 2 | | | | | | | | o |
| cocaine- and amphetamine-regulated transcript protein | 2 | o | | | | | | | |
| coenzyme q10 homolog b ( cerevisiae) | 2 | o | o | | | | | | |
| coenzyme q2 prenyltransferase | 2 | o | | | | | | | |
| coenzyme q-binding protein coq10 homolog mitochondrial-like | 2 | | | | | | o | | |
| cohesin loading complex subunit scc4 homolog | 2 | o | o | | | | | | |
| coiled-coil domain containing 137 | 2 | o | | | | | | | |
| coiled-coil domain containing 44 | 2 | o | | | | | | | |
| coiled-coil domain containing 45 | 2 | o | o | | | | | | |
| coiled-coil domain containing 55 | 2 | o | | o | | | | | |
| coiled-coil domain-containing protein 104 | 2 | | | | | | | | o |
| coiled-coil domain-containing protein 106 | 2 | | | | | o | | | o |
| coiled-coil domain-containing protein 124-like | 2 | | | | o | | | | o |
| coiled-coil domain-containing protein 23 | 2 | | o | | | | | | o |
| coiled-coil domain-containing protein 40-like | 2 | | | | | | | | o |
| coiled-coil domain-containing protein 43-like | 2 | | | | o | | | | |
| coiled-coil domain-containing protein 58 | 2 | o | o | | | | | | |
| coiled-coil domain-containing protein 75 | 2 | o | | | | | | o | |
| coiled-coil-helix-coiled-coil-helix domain-containing protein mitochondrial-like | 2 | | | | o | | | | o |
| collagen alpha-2 chain-like | 2 | o | | | | | | | o |
| collagen alpha-3 chain | 2 | o | | | | | | | |
| collagen alpha-4 chain- partial | 2 | o | | | | | | | |
| collagen triple helix repeat containing 1 | 2 | o | | | | | | | |
| collagen type i alpha 2 | 2 | | | o | | | | | o |
| collagen type iv alpha-3-binding | 2 | o | o | | | | | | |
| collagen-like triple helix repeat protein | 2 | | | | o | | | | o |
| collectin sub-family member 12 | 2 | o | | | | | | | |
| comm domain containing 4 | 2 | o | | | | o | | | |
| comm domain-containing protein 5 | 2 | o | | o | | | | | |
| comm domain-containing protein 6 | 2 | o | | | | | | | o |
| complement c1q-like protein 2-like | 2 | | o | | | | | | |
| complement c1s subcomponent | 2 | o | | o | | | | | |
| complement component alpha polypeptide | 2 | | o | o | | | | | |
| complement component c4 | 2 | | | | o | | | o | |
| complement component c7 precursor | 2 | | o | o | | | | | |
| complement component gamma polypeptide | 2 | | o | o | | | | | |
| complement factor b precursor | 2 | | | | o | | | | |
| complement factor d | 2 | o | | o | | | | | |
| complement factor h1 protein | 2 | | | | | o | | o | |
| complement factor h-related 4 | 2 | | o | | o | | | | |
| complement factor partial | 2 | | | | o | | | | |
| complement regulatory plasma protein | 2 | | o | | | | | | |
| complexin 1 | 2 | | | | | | | | o |
| component of oligomeric golgi complex 5 | 2 | o | | | | o | | | |
| condensin complex subunit 3 | 2 | | o | | o | | | | |
| conserved domain protein | 2 | o | | | | | | | o |
| conserved hypothetical protein [Ixodes scapularis] | 2 | | o | | | o | | | |
| conserved hypothetical protein [Perkinsus marinus ATCC 50983] | 2 | | | | | | | | o |
| conserved oligomeric golgi complex subunit 7 | 2 | | | | | | | | o |
| contactin 6 | 2 | o | | | | | o | | |
| contactin a | 2 | | | | | | o | | |
| contactin associated 2 | 2 | | | | | | o | | |

| | | | | | | | | | |
|---|---|---|---|---|---|---|---|---|---|
| contactin-1 precursor | 2 | | | | | | o | o | |
| contactin-associated protein 1-like | 2 | | | | | | o | | |
| cop9 constitutive photomorphogenic homolog subunit 4 | 2 | | o | | | o | | | |
| cop9 signalosome complex subunit 2 | 2 | | | | o | | | | |
| copia ltr rider | 2 | | | o | | | | | |
| copine v | 2 | | | | | | o | | |
| corin variant1-like | 2 | | | | | | o | o | |
| coronin 6 | 2 | o | | | | | | | |
| coronin 6 clipin e type c isoform 1 | 2 | o | | | | o | | | |
| coronin 7 | 2 | o | | | | | | | |
| cox16-like protein c14orf112 mitochondrial precursor | 2 | | | | o | | | o | |
| creatine kinase b-type | 2 | | | | o | | | | o |
| creatine mitochondrial 2 | 2 | o | | | | | | | |
| creb-binding protein | 2 | o | | | | | o | | |
| cse1 chromosome segregation 1-like | 2 | | o | | | | o | | |
| csp and trap-related protein | 2 | | | | o | | | | o |
| csrp2 binding protein | 2 | o | | | | o | | | |
| c-terminal binding protein 1 | 2 | o | | | | | o | | |
| ctp synthase | 2 | o | | | | | o | | |
| ctp synthase 1 | 2 | o | o | | | | | | |
| c-type lectin domain family 10 member a-like | 2 | | | | o | | | | |
| c-type lectin domain family 4 member m | 2 | | o | | | | | | |
| c-type lectin domain family member b | 2 | o | | | | o | | | |
| c-type natriuretic peptide 1 precursor | 2 | | | | | | | o | o |
| cullin 4a | 2 | | | | | o | o | | |
| cullin 5 | 2 | o | | | | | | | |
| cullin-associated nedd8-dissociated protein 1 | 2 | | o | | | | o | | |
| cyclic amp-dependent transcription factor atf-6 alpha-like protein | 2 | o | | | | | | | |
| cyclin e2 | 2 | o | | | | | | | |
| cyclin f | 2 | | | o | | | | | |
| cyclin g associated kinase | 2 | | | | | | o | o | |
| cyclin h | 2 | o | | | | o | | | |
| cyclin l1 | 2 | o | o | | | | | | |
| cyclin t2 | 2 | o | o | | | | | | |
| cyclin y-like 1 | 2 | | | o | | o | | | |
| cyclin-dependent kinase 11b | 2 | | o | | | | o | | |
| cyclin-dependent kinase inhibitor 1b | 2 | | o | | | | | | |
| cyclooxygenase 1 | 2 | o | | | | | | | |
| cystatin b | 2 | | | o | | | | | o |
| cysteine mitochondrial | 2 | o | | | | o | | | |
| cysteinyl-trna cytoplasmic | 2 | o | | | | | | | |
| cystine glutamate transporter | 2 | | | | | o | | | |
| cytidylate kinase | 2 | | o | | | | | | o |
| cytochrome b- alpha polypeptide | 2 | o | | o | | | | | |
| cytochrome b5 domain containing 2 | 2 | o | | | | o | | | |
| cytochrome c oxidase assembly protein cox19 | 2 | o | o | | | | | | |
| cytochrome p450 2k1-like | 2 | | o | | o | | | | |
| cytokine induced apoptosis inhibitor 1 | 2 | o | | | | o | | | |
| cytokine receptor family member b13 | 2 | o | | | | | | | |
| cytokine-inducible sh2-containing protein | 2 | o | | o | | | | | |
| cytoplasmic fmr1-interacting protein 2 | 2 | | | | | | o | | |
| cytoplasmic fmr1-interacting protein 2-like | 2 | | | | | | o | | |
| cytoskeleton-associated protein 5 | 2 | | | | | | o | o | |
| cytosolic 5 -nucleotidase 3 | 2 | o | | o | | | | | |
| cytosolic iron-sulfur protein assembly 1 homolog ( cerevisiae) | 2 | o | | o | | | | | |
| cytosolic nonspecific dipeptidase | 2 | | o | | | | | o | |

| | | | | | | | | | |
|---|---|---|---|---|---|---|---|---|---|
| d chain crystal structure of the pyruvate dehydrogenase component of human pyruvate dehydrogenase complex | 2 | | | | | | | | o |
| d-2-hydroxyglutarate dehydrogenase | 2 | o | o | | | | | | |
| d-3-phosphoglycerate dehydrogenase | 2 | | | | | | | o | o |
| dachsous 1 | 2 | | | | | | o | | |
| damage-regulated autophagy modulator | 2 | o | | o | | | | | |
| dclk2 protein | 2 | | | | | | | o | |
| dead (asp-glu-ala-asp) box polypeptide 17 | 2 | o | | | | | o | | |
| dead (asp-glu-ala-asp) box polypeptide 46 | 2 | o | | o | | | | | |
| dead (asp-glu-ala-asp) box polypeptide 5 | 2 | | o | | o | | | | |
| deah (asp-glu-ala-his) box polypeptide 33 | 2 | | | | o | | | | |
| deah (asp-glu-ala-his) box polypeptide 35 | 2 | o | | | | | | | |
| dec-205 cd205 | 2 | o | | | | | | | |
| decaprenyl-diphosphate synthase subunit 1 | 2 | | | | o | | | | o |
| dedicator of cytokinesis 1 | 2 | | o | | | | | | |
| dedicator of cytokinesis 2 | 2 | o | | | | | | | |
| dedicator of cytokinesis protein 9 | 2 | o | | | | | o | | |
| dedicator of cytokinesis protein 9-like | 2 | o | | | | | o | | |
| degenerative spermatocyte homolog lipid desaturase | 2 | | | o | o | | | | |
| dehydrogenase reductase (sdr family) member 7 | 2 | o | | | | o | | | |
| dehydrogenase reductase sdr family member 11-like | 2 | | | | o | | | | o |
| dehydrogenase reductase sdr family member 12-like | 2 | | | | | | | | o |
| dek oncogene (dna binding) | 2 | o | | | | o | | | |
| denn domain-containing protein 5b-like | 2 | o | o | | | | | | |
| denn madd domain containing 1a | 2 | o | | | | | o | | |
| denn madd domain containing 5a | 2 | | | | | o | | o | |
| dennd4a protein | 2 | o | | | | | | | |
| deoxyribonuclease tatdn3-like | 2 | o | | | | | | | |
| deoxyuridine triphosphatase | 2 | o | o | | | | | | |
| der1-like domain member 1 | 2 | o | o | | | | | | |
| der1-like domain member 2 | 2 | o | o | | | | | | |
| desmin | 2 | o | | | | | | | |
| destrin | 2 | o | | | | o | | | |
| dexamethasone-induced 1 | 2 | | | | | o | | o | |
| diacylglycerol delta 130kda isoform 1 | 2 | o | | | | | | | |
| diaphanous 2 | 2 | | | | | | o | o | |
| diaphanous homolog 2 | 2 | o | | | | | o | | |
| diaphanous homolog 3 | 2 | o | | | o | | | | |
| differentially expressed in fdcp 6 homolog | 2 | | o | | | | | | |
| differentially regulated trout protein 1 | 2 | | o | o | | | | | |
| digestive organ expansion factor homolog | 2 | o | | | | | | | o |
| dihydrolipoamide s-acetyltransferase (e2 component of pyruvate dehydrogenase complex) | 2 | o | o | | | | | | |
| dihydroorotate dehydrogenase | 2 | o | o | | | | | | |
| -dihydroxyvitamin d 24- mitochondrial | 2 | | o | | | | | | |
| dipeptidyl peptidase 3 | 2 | | o | | | | | | |
| disabled homolog mitogen-responsive phosphoprotein | 2 | o | o | | | | | | |
| discoidin domain receptor tyrosine kinase 2 | 2 | o | | | | | | | |
| disks large homolog 2-like | 2 | | | | | | | o | |
| disks large-associated protein 4 | 2 | | | | | | o | | |
| dj-1 protein | 2 | | | | | o | | | o |
| dmx-like 1 isoform 1 | 2 | | | | | | o | | |
| dmx-like protein 2-like | 2 | | | | | | o | | |

| | | | | | | | | | |
|---|---|---|---|---|---|---|---|---|---|
| dna polymerase delta subunit 4 | 2 | o | | o | | | | | |
| dna repair protein xrcc1 | 2 | | | | o | | | o | |
| dna repair protein xrcc4 | 2 | o | | | | o | | | |
| dna replication inhibitor | 2 | o | | | | | | | |
| dna replication licensing factor mcm5 | 2 | | | | o | | | | |
| dna replication licensing factor mcm7 | 2 | o | | | o | | | | |
| dna replication licensing factor mcm7-like | 2 | | | | o | | | | |
| dna translocase | 2 | | | | o | | | | |
| dna-binding protein rfx7-like | 2 | | | | | | o | | |
| dna-binding protein rfxank | 2 | o | | | | | | | |
| dna-directed rna polymerase i subunit rpa12 | 2 | o | | | | | | o | |
| dna-directed rna polymerase ii subunit rpb3 | 2 | | o | | | | | | o |
| dna-directed rna polymerase ii subunit rpb9 | 2 | o | | | | | | | |
| dna-directed rna polymerase iii subunit rpc7 | 2 | | | | o | | | | |
| dna-directed rna polymerases i and iii subunit rpac1 | 2 | | | o | | | | | o |
| dnaj homolog subfamily a member 2-like | 2 | | | | | | | | o |
| dnaj homolog subfamily a member 4 | 2 | | | | o | | | | |
| dnaj homolog subfamily c member 18 | 2 | | | | o | | | | o |
| dnaj homolog subfamily c member 2 | 2 | | | | o | | o | | |
| dock9 protein | 2 | o | | | | | o | | |
| dolichyl-diphosphooligosaccharide--protein glycosyltransferase subunit 1-like | 2 | | | | | | | | o |
| dol-p-glc:glc man c -pp-dol alpha- -glucosyltransferase-like | 2 | | | | o | | | | o |
| dom-3 homolog z ( elegans) | 2 | o | | o | | | | | |
| domain containing 3 | 2 | o | o | | | | | | |
| domain protein dehydratase | 2 | o | | | | | | | o |
| double-stranded rna activated protein kinase | 2 | | o | | | o | | | |
| dpy-19-like 4 ( elegans) | 2 | o | | | | | | | |
| dr1-associated corepressor | 2 | o | o | | | | | | |
| dsc2l protein | 2 | | o | | | | | | o |
| dspa protein | 2 | | | | | | | | o |
| dtw domain containing 1 | 2 | o | | | | | | o | |
| dual specificity mitogen-activated protein kinase kinase 6 | 2 | o | o | | | | | | |
| dual specificity phosphatase 12 | 2 | o | o | | | | | | |
| dual specificity phosphatase 5 | 2 | o | | | | | o | | |
| dual specificity protein kinase clk4 | 2 | | o | | | | | | |
| dual specificity protein phosphatase 7 | 2 | o | | | | | | | o |
| dutp pyrophosphatase | 2 | | | | | o | | | |
| dynactin 5 | 2 | o | | | | o | | | |
| dynamin 1 | 2 | | | | | | o | | |
| dystroglycan 1 (dystrophin-associated glycoprotein 1) | 2 | o | | | | | o | | |
| e2a-1 transcription factor | 2 | o | | | | | o | | |
| e3 ubiquitin isg15 ligase trim25 | 2 | | o | o | | | | | |
| e3 ubiquitin-protein ligase itchy homolog | 2 | | | | | | o | | |
| e3 ubiquitin-protein ligase mib2 | 2 | | o | | | | | | |
| e3 ubiquitin-protein ligase pdzrn3 | 2 | | | | | | | | o |
| e3 ubiquitin-protein ligase ubr7-like | 2 | | | | o | | | | |
| early b-cell factor 1 | 2 | o | | | | | | | |
| ebna1 binding protein 2 | 2 | | o | o | | | | | |
| ectoderm-neural cortex protein 1 | 2 | | | | | | o | | |
| ectodysplasin splice variant-9 exons | 2 | o | | | o | | | | |
| ectonucleoside triphosphate diphosphohydrolase 2 | 2 | | o | o | | | | | |
| ectonucleotide pyrophosphatase phosphodiesterase 4 ( function) | 2 | | | | | | o | | |

| | | | | | | | | | |
|---|---|---|---|---|---|---|---|---|---|
| ectonucleotide pyrophosphatase phosphodiesterase 5 ( function) | 2 | o | | | | | | | |
| eef1a1 protein | 2 | | | | | | | | o |
| ef-hand domain member a2 | 2 | o | | | | | | | |
| ef-hand domain-containing family member a2 | 2 | | o | | | | o | | |
| ef-hand domain-containing protein d2 | 2 | o | | o | | | | | |
| ef-hand domain-containing protein kiaa0494-like | 2 | o | o | | | | | | |
| egf- fibronectin type iii and laminin g domains | 2 | | | o | | | | | |
| egf-like domain 7 | 2 | | | o | | o | | | |
| eh domain-binding protein 1 isoform 3 | 2 | | | | | | o | o | |
| elastase pancreatic | 2 | | o | | | | | | |
| elastin microfibril interfacer 2 | 2 | o | o | | | | | | |
| electron-transferring-flavoprotein dehydrogenase | 2 | | o | | | | | | |
| ell associated factor 2 | 2 | o | | | | | | o | |
| elmo ced-12 domain containing 1 | 2 | | | | | | o | | |
| elmo domain-containing protein 1 | 2 | o | | | | | o | | |
| elmo domain-containing protein 2 | 2 | | o | o | | | | | |
| elongation factor 1 alpha isoform 42sp50 | 2 | | | | o | | | | |
| elongation factor 1-alpha 1-like | 2 | | | | | | o | | o |
| elongation factor rna polymerase ii | 2 | o | | | | | | o | |
| elongation factor-2 kinase | 2 | o | | | | o | | | |
| elongation of very long chain fatty acids protein 4 | 2 | o | | | | | | o | |
| elongation of very long chain fatty acids protein 6 | 2 | o | o | | | | | | |
| embryonic muscle myosin heavy chain | 2 | o | | | | | | | |
| endo-alpha | 2 | | o | | | o | | | |
| endonuclease domain-containing 1 | 2 | | o | | | | | | |
| endonuclease reverse partial | 2 | | | | | | o | o | |
| endonuclease-reverse transcriptase -e01 | 2 | | | | | | | | o |
| endopeptidase type m3 | 2 | o | | | | | | | |
| endoplasmic reticulum lectin 1 isoform 3 | 2 | | | o | | | | o | |
| endoplasmic reticulum-golgi intermediate compartment 1 | 2 | o | | | | | | | |
| endoplasmic reticulum-golgi intermediate compartment protein 2 | 2 | o | | | | | | | |
| endoribonuclease dicer | 2 | o | | | | | | | |
| endosulfine alpha | 2 | o | | | | | | o | |
| endothelial differentiation-related factor 1 | 2 | o | o | | | | | | |
| endothelial differentiation-related factor 1 variant 1 | 2 | | | o | | | | o | |
| endothelial lipase | 2 | o | o | | | | | | |
| endothelin-converting enzyme 1 | 2 | o | o | | | | | | |
| enolase 2 | 2 | | | | | o | | | o |
| enoyl coenzyme a hydratase domain containing 3 | 2 | | | o | | | | | o |
| ependymin | 2 | | | | | | | o | o |
| eph receptor b2 | 2 | o | | | | | o | | |
| ephrin-b1 | 2 | o | | | | | o | | |
| epithelial cell adhesion molecule | 2 | o | | | | | | | |
| epithelial stromal interaction 1 | 2 | o | o | | | | | | |
| epsilon 1 | 2 | o | | | | | | | |
| equilibrative nucleoside transporter 1 | 2 | o | | o | | | | | |
| equilibrative nucleoside transporter 3-like | 2 | o | o | | | | | | |
| er degradation-enhancing alpha-mannosidase-like 3-like | 2 | | o | | | | | | o |
| er lipid raft associated 2 | 2 | | o | | | | | | o |
| er lumen protein retaining receptor 2 | 2 | | o | | | | | | o |

| | | | | | | | | | |
|---|---|---|---|---|---|---|---|---|---|
| ergic and golgi 2 | 2 | o | | | | | | | |
| ero1-like beta ( cerevisiae) | 2 | | | o | | | | | |
| erythroblast membrane-associated | 2 | o | | | | | | o | |
| erythrocyte membrane protein band -like 2 | 2 | | | | | | o | o | |
| estrogen receptor alpha | 2 | | | o | | | | | |
| estrogen-regulated protein | 2 | o | | | | o | | | |
| euchromatic histone-lysine n-methyltransferase 2 | 2 | o | | | | | | o | |
| eukaryotic translation elongation factor 1 epsilon 1 | 2 | o | o | | | | | | |
| eukaryotic translation initiation factor 1 | 2 | o | | | | | o | | |
| eukaryotic translation initiation factor 2 subunit 2 | 2 | | | | | | | | o |
| eukaryotic translation initiation factor 2-alpha kinase 1 | 2 | | | | o | | | | |
| eukaryotic translation initiation factor 2-alpha kinase 2 | 2 | | | o | | | | | |
| eukaryotic translation initiation factor 3 subunit 3 gamma | 2 | o | | | | | | | |
| eukaryotic translation initiation factor 4 gamma 2-like | 2 | | | | | | | | o |
| eukaryotic translation initiation factor 4b | 2 | | o | | | o | | | |
| eukaryotic translation initiation factor 4e binding protein 2 | 2 | o | | | | | o | | |
| eukaryotic translation initiation factor 4e nuclear import factor 1 | 2 | o | | | | | | | |
| eukaryotic translation initiation factor 4e type 2 | 2 | | | | o | | | | |
| eukaryotic translation initiation factor 4e-1a-binding protein | 2 | o | | | | o | | | |
| eukaryotic translation initiation factor gamma 1 | 2 | o | | | | | | | |
| eukaryotic translation initiation factor isoform 2 | 2 | o | | | | | o | | |
| eukaryotic translation initiation factor subunit 10 | 2 | | o | | | | | | o |
| eukaryotic translation initiation factor subunit 3 gamma | 2 | o | | o | | | | | |
| excision repair cross-complementing rodent repair deficiency complementation group 1 | 2 | o | | | | | | | |
| exocyst complex component 5 | 2 | o | o | | | | | | |
| exosome complex exonuclease rrp41 | 2 | o | | o | | | | | |
| exosome component 6 | 2 | | o | | o | | | | |
| exostoses 2 | 2 | o | o | | | | | | |
| exostosin-2 | 2 | | | | | | | o | o |
| exostosin-like 2 | 2 | o | o | | | | | | |
| exportin 6 | 2 | | o | | | | | o | |
| exportin crm1 homolog | 2 | o | o | | | | | | |
| extracellular matrix protein 1 precursor | 2 | | | | | | | | o |
| f10 protein | 2 | | o | o | | | | | |
| factor in the germline alpha | 2 | | | | o | | | | |
| factor viii intron 22 protein | 2 | | o | | | | | | |
| fam54b protein | 2 | o | | | | | | o | |
| family with sequence similarity 120c | 2 | o | | | | | o | | |
| family with sequence similarity 62 (c2 domain containing) member a | 2 | o | | | | | | | |
| family with sequence similarity member a-like | 2 | o | | | | | | | |
| far upstream element binding protein 3 | 2 | o | o | | | | | | |
| fas associated factor family member 2 | 2 | | | o | | o | | | |
| fas-associated factor 2 | 2 | | o | | | | o | | |

| | | | | | | | | | |
|---|---|---|---|---|---|---|---|---|---|
| fast kinase domain-containing protein 3 | 2 | o | | | | | | | |
| fast kinase domains 2 | 2 | o | o | | | | | | |
| fat storage-inducing transmembrane protein 1 | 2 | o | | | | | | | |
| fatty acid-binding intestinal | 2 | o | o | | | | | | |
| f-box and wd-40 domain protein 11 | 2 | o | | | | | | | |
| f-box only protein 6b | 2 | | | | | o | | | o |
| f-box protein 33 | 2 | | | | | | o | | |
| f-box wd repeat-containing protein 7 isoform 1 | 2 | o | | | | | | | |
| ferm domain containing 4b | 2 | o | | o | | | | | |
| ferredoxin 1 | 2 | o | | | | o | | | |
| ferritin high chain | 2 | | | | o | | | | o |
| fetuin-B [Perca flavescens] | 2 | | o | o | | | | | |
| fibrillarin | 2 | o | o | | | | | | |
| fibrinogen and fibronectin | 2 | | | | | | | | o |
| fibrinogen-like 2 | 2 | o | | o | | | | | |
| fibroblast growth factor 12 | 2 | | | | | | o | o | |
| fibroblast growth factor 13 | 2 | o | | | | | | | |
| fibroblast growth factor receptor 1 | 2 | | o | | | | o | | |
| fibronectin type iii and spry domain containing 2 | 2 | o | | | | | | | |
| fibronectin type iii domain-containing protein 7-like | 2 | | | | | | | | o |
| fk506 binding protein 4 | 2 | o | o | | | | | | |
| fk506 binding protein 5 | 2 | | o | | | | o | | |
| fk506 binding protein 65 kda | 2 | | | | | o | | o | |
| flap endonuclease 1-b | 2 | o | o | | | | | | |
| flavin reductase | 2 | o | o | | | | | | |
| flotillin 2 | 2 | o | o | | | | | | |
| folliculin interacting protein 1 | 2 | o | | | | | | | |
| follistatin-like 1 | 2 | o | o | | | | | | |
| forkhead box a2 | 2 | | o | | | | | | |
| forkhead box k2 | 2 | o | | | | | | o | |
| forkhead box p4 isoform 1 | 2 | | | | | | o | | |
| formiminotransferase cyclodeaminase | 2 | | o | | | | | | |
| four and a half lim domains 3 | 2 | o | | | | o | | | |
| four and a half lim domains protein 1 | 2 | o | | | | | | | |
| fras1 related extracellular matrix protein 2 | 2 | o | | | | | | | |
| fructose- -bisphosphatase | 2 | o | o | | | | | | |
| fumarylacetoacetate hydrolase domain containing 2a | 2 | o | o | | | | | | |
| fxyd domain-containing ion transport regulator 6 precursor | 2 | | | | | | o | o | |
| fyve-finger-containing rab5 effector protein rabenosyn-5 | 2 | o | o | | | | | | |
| g patch domain and kow motifs | 2 | o | o | | | | | | |
| g protein-coupled receptor 142 | 2 | | | | | | o | | |
| g protein-coupled receptor 177 | 2 | o | | | | | | o | |
| g protein-coupled receptor 98 | 2 | o | | | | o | | | |
| ga-binding protein alpha chain | 2 | | | | | | | | o |
| gag-pol precursor polyprotein | 2 | | o | | | | | o | |
| galactocerebrosidase precursor | 2 | o | o | | | | | | |
| galactosamine (n-acetyl)-6-sulfate sulfatase | 2 | o | | | | | | | |
| galactoside- 2 | 2 | | | o | | o | | | |
| galactoside- 8 (galectin 8) | 2 | o | o | | | | | | |
| galactosylceramide sulfotransferase | 2 | o | | | | o | | | |
| galectin 3 | 2 | o | | | | | | | |
| gamma polypeptide | 2 | | | | o | | | | |
| gamma2-synuclein [Takifugu rubripes] | 2 | o | | | | | | | |
| gamma-aminobutyric acid a gamma 1 | 2 | | | | | | o | o | |

| | | | | | | | | | |
|---|---|---|---|---|---|---|---|---|---|
| gamma-aminobutyric acid b receptor 2 | 2 | | | | | | o | o | |
| gamma-aminobutyric acid receptor subunit alpha-1 precursor | 2 | | | | | o | | o | |
| gamma-aminobutyric acid receptor subunit rho-3 | 2 | o | | | | | | | |
| gamma-glutamyl carboxylase | 2 | | o | o | | | | | |
| gamma-glutamyl cyclotransferase | 2 | | | o | | | | o | |
| gamma-glutamyltransferase 5 | 2 | o | | | | | | | |
| gamma-glutamyltransferase 7 | 2 | | | | | | | o | o |
| gamma-secretase subunit aph-1b | 2 | | o | | | | o | | |
| ganglioside sialidase | 2 | | | | | o | | o | |
| gap junction gamma-1 protein | 2 | o | o | | | | | | |
| gastrula zinc finger | 2 | | | | o | | | | |
| gata zinc finger domain containing 2a | 2 | o | | | | | o | | |
| gdp-mannose pyrophosphorylase a | 2 | o | | | | | | | |
| gem-associated protein 7 | 2 | o | | | | | | o | |
| general transcription factor 3c polypeptide 3 | 2 | | o | | | | | | |
| general transcription factor 3c polypeptide 6 | 2 | o | | o | | | | | |
| general transcription factor iif subunit 1 | 2 | | | o | | o | | | |
| general transcription factor polypeptide 30kda | 2 | o | o | | | | | | |
| general transcription factor polypeptide 74kda | 2 | o | | | | | | o | |
| general transcription factor polypeptide beta 34kda | 2 | | | | | | | o | o |
| genetic suppressor element 1 | 2 | o | | | | | | o | |
| ggnbp2 protein | 2 | o | o | | | | | | |
| gla-rich protein | 2 | o | | | | | | | |
| gli pathogenesis-related 2 | 2 | o | | | | | | o | |
| glucagon receptor | 2 | | o | | | o | | | |
| glucokinase regulatory protein | 2 | | o | o | | | | | |
| glucosamine (n-acetyl)-6-sulfatase | 2 | o | | | | | | | |
| glucosamine-6-phosphate isomerase 1 | 2 | | o | | | | | | o |
| glucose-6-phosphate translocase | 2 | | | | o | | | | o |
| glucosylceramidase isoform 2 | 2 | | | o | | | | | o |
| glutamate ampa | 2 | | | | | | | | o |
| glutamate metabotropic 1 | 2 | | | | | | o | o | |
| glutamate n-methyl d-aspartate 2d | 2 | | | o | | | o | | |
| glutamine rich 2-like isoform 1 | 2 | | | | o | | | | |
| glutamine-rich protein 1 | 2 | o | | | | | | | o |
| glutaminyl-peptide cyclotransferase | 2 | | o | o | | | | | |
| glutaminyl-trna synthetase-like | 2 | | o | | o | | | | |
| glutaredoxin-like protein ydr286c homolog | 2 | o | | | | o | | | |
| glutathione peroxidase 2 | 2 | o | | | | o | | | |
| glutathione peroxidase 6 precursor | 2 | | | | o | | | | |
| glutathione peroxidase 7 | 2 | o | | | | o | | | |
| glutathione s-transferase a | 2 | | | | | | | | o |
| glyceraldehyde-3-phosphate liver | 2 | o | | | | | o | | |
| glycerate kinase | 2 | | o | | | | | | |
| glycerol-3-phosphate acyltransferase mitochondrial | 2 | | o | | | | o | | |
| glycine beta | 2 | o | | | | | o | | |
| glycine c-acetyltransferase (2-amino-3-ketobutyrate-coenzyme a ligase) | 2 | o | o | | | | | | |
| glycine n-acyltransferase-like protein 3 | 2 | o | | o | | | | | |
| glycine n-methyltransferase | 2 | | o | | | | | | |
| glycogen | 2 | o | | | o | | | | |
| glycogen phosphorylase | 2 | | | | o | | | | |
| glycogen synthase kinase-3 beta | 2 | | | | | | o | | |
| glycoprotein endo-alpha- -mannosidase | 2 | | o | | o | | | | |

| | | | | | | | | | |
|---|---|---|---|---|---|---|---|---|---|
| glycoprotein m6a | 2 | o | | | | | o | | |
| glycoprotein-n-acetylgalactosamine 3-beta-galactosyltransferase 1 | 2 | | | | | | | | o |
| glyoxalase domain-containing protein 5 | 2 | | | o | | | | o | |
| gnas complex locus | 2 | o | | | | | | | |
| golgi golgin subfamily 4 | 2 | | | | | o | | | |
| golgi ph regulator | 2 | o | | | o | | | | |
| golgi reassembly-stacking protein 1-like | 2 | | | | o | | | | o |
| golgi-specific brefeldin a-resistance guanine nucleotide exchange factor 1 | 2 | o | | o | | | | | |
| gon-4-like protein | 2 | | | | | | | | o |
| gpi-anchored adp-ribosyltransferase | 2 | o | | | | | | | |
| gpn-loop gtpase 2-like | 2 | | | | | | | | o |
| gram domain-containing protein 1b | 2 | | | | | | o | o | |
| granzyme f | 2 | o | o | | | | | | |
| grb10 interacting gyf protein 2 protein | 2 | o | o | | | | | | |
| growth arrest and dna damage-inducible proteins-interacting protein 1 | 2 | | | | | | | | o |
| growth arrest and dna-damage-inducible protein gadd45 gamma | 2 | | | | | o | o | | |
| growth arrest-specific 6 | 2 | o | o | | | | | | |
| growth hormone receptor | 2 | o | | | | | | | |
| growth hormone receptor type ii | 2 | o | o | | | | | | |
| grpe protein homolog mitochondrial-like | 2 | | | | o | | | | |
| gsk3-beta interaction protein | 2 | o | | o | | | | | |
| gtp cyclohydrolase 1 | 2 | | o | | o | | | | |
| gtpase activating protein (sh3 domain) binding protein 2 | 2 | | o | | | | | o | |
| gtpase activating protein and vps9 domains 1 | 2 | o | | | | | o | | |
| gtpase activating rap domain-like 1 | 2 | | | | | o | | o | |
| gtp-gdp dissociation stimulator 1 | 2 | o | | | | | | o | |
| guanine nucleotide binding alpha inhibiting 1 | 2 | | o | o | | | | | |
| guanine nucleotide binding protein (g protein) alpha activating activity polypeptide o | 2 | | | | | o | o | | |
| guanine nucleotide binding protein (g protein) alpha inhibiting activity polypeptide 3 | 2 | o | | | | | | o | |
| guanine nucleotide binding protein (g protein) beta polypeptide 2-like 1 | 2 | | | | | | | | o |
| guanine nucleotide binding protein (g protein) gamma 10 | 2 | o | | | | | | o | |
| guanine nucleotide exchange factor dbs | 2 | | | | | | o | | |
| guanine nucleotide exchange factor dbs-like | 2 | | | | | | | | o |
| guanine nucleotide-binding protein g subunit alpha-1 | 2 | o | | | | | | | |
| guanine nucleotide-binding protein gi gs go subunit gamma-5 precursor | 2 | | | | | o | o | | |
| guanine nucleotide-binding protein gi gs gt subunit beta-1 | 2 | | | | | o | | o | |
| guanine nucleotide-binding protein subunit beta-5 | 2 | | | | | | | | o |
| guanosine monophosphate reductase | 2 | o | | | | | | o | |
| h aca ribonucleoprotein complex subunit 2-like | 2 | | o | | o | | | | |
| h aca ribonucleoprotein complex subunit 2-like protein | 2 | o | | | o | | | | |
| h g transactivated protein 2 isoform 1 | 2 | | | | | o | o | | |
| h+ lysosomal 56 v1 subunit b2 | 2 | o | | o | | | | | |
| h+ lysosomal v0 subunit b | 2 | o | o | | | | | | |
| h+ transporting atp synthase beta subunit | 2 | | | | | | | o | o |
| h1 histone | 2 | | | | o | | o | | |

| | | | | | | | | | |
|---|---|---|---|---|---|---|---|---|---|
| h2a histone member v | 2 | | o | | | o | | | |
| h2a histone member y | 2 | o | | | | o | | | |
| haloacid dehalogenase-like hydrolase domain containing 2 | 2 | o | o | | | | | | |
| hat family dimerisation domain containing protein | 2 | o | | | | | | | |
| hCG2045189 [Homo sapiens] | 2 | | | | | o | | o | |
| heart of glass | 2 | o | | | | | | | |
| heat shock 27kda protein 1 | 2 | o | | | | | | | o |
| heat shock 70 kda protein 12a | 2 | | | | | | o | o | |
| heat shock 70 kda protein 4l | 2 | | | | | | | o | o |
| heat shock 70 kda protein 4-like | 2 | | | | o | | | | |
| heat shock 70kda protein 5 | 2 | | | o | | | | | |
| heat shock cognate 71 kda | 2 | | o | | | | o | | |
| heat shock protein alpha-like | 2 | | | | o | | | | |
| heat shock protein beta member 1 | 2 | o | | o | | | | | |
| heavy chain 7b | 2 | o | | | | | | | |
| heavy polypeptide 200kda-like isoform 2 | 2 | o | | | | | | | |
| heavy polypeptide non-muscle | 2 | | o | | | | | o | |
| hect domain and rld 2 | 2 | | | | | | o | o | |
| hect domain and rld 4 isoform 1 | 2 | o | | o | | | | | |
| hect domain containing 3 | 2 | o | o | | | | | | |
| hedgehog interacting 1 | 2 | | | o | | o | | | |
| helicase arip4 | 2 | o | | | | | | | |
| hematopoietic signal peptide-containing precursor | 2 | | o | | | o | | | |
| heme-binding protein 1 | 2 | o | | | | | | | o |
| hemimethylated dna binding domain-containing protein | 2 | | o | o | | | | | |
| heparan sulfate proteoglycan 2 | 2 | | o | | | o | | | |
| heparin-binding growth factor 1 | 2 | o | | | | | | | o |
| hepatocellular carcinoma-associated antigen isoform cra_a | 2 | | o | o | | | | | |
| hepatocyte growth factor | 2 | o | o | | | | | | |
| hepatocyte growth factor activator | 2 | | o | | | | | | |
| hepatoma-derived growth related protein 2 | 2 | o | | | | | | | |
| heterogeneous nuclear ribonucleoprotein a0 | 2 | o | o | | | | | | |
| heterogeneous nuclear ribonucleoprotein a0-like | 2 | | | | o | | | | o |
| heterogeneous nuclear ribonucleoprotein u-like protein 1 | 2 | o | o | | | | | | |
| hexokinase 2 | 2 | o | o | | | | | | |
| hexose-6-phosphate dehydrogenase (glucose 1-dehydrogenase) | 2 | | o | o | | | | | |
| high mobility group nucleosomal binding domain 3 | 2 | | | o | | | | o | |
| high-mobility group protein 2-like 1 | 2 | o | | | | o | | | |
| hippocalcin-like 1 | 2 | o | | | | | | o | |
| histidine decarboxylase | 2 | | o | o | | | | | |
| histidine kinase- dna gyrase b- and hsp90-like domain containing protein | 2 | o | | | | o | | | |
| histidyl-trna synthetase | 2 | o | | | | o | | | |
| histone acetyltransferase 1 | 2 | o | | | | | | | o |
| histone chaperone asf1a | 2 | o | | | | | | | |
| histone deacetylase 9 protein | 2 | o | | | | | | | |
| histone deacetylase 9-like | 2 | | | | | | o | o | |
| histone deacetylase family protein | 2 | o | | | | | | | |
| histone -like | 2 | | | | | | | o | o |
| histone-lysine n-methyltransferase mll3 | 2 | | | | | | o | | |
| homeodomain-only protein | 2 | o | | | | o | | | |

| | | | | | | | | | |
|---|---|---|---|---|---|---|---|---|---|
| homogentisate -dioxygenase | 2 | | o | | o | | | | |
| homologue of sarcophaga proteinase | 2 | | o | o | | | | | |
| host cell factor c1 | 2 | | | | | o | o | | |
| hsp70 protein | 2 | | | | o | | | o | |
| hsp90aa1 protein | 2 | | | | o | | | | o |
| hyaluronan and proteoglycan link protein 1 | 2 | | | | | o | | o | |
| hyaluronan-binding protein 2 | 2 | | | | o | | | | |
| hydroxyacid oxidase 1 | 2 | | o | | | | | | |
| hydroxymethylglutaryl- mitochondrial precursor | 2 | | o | o | | | | | |
| hydroxysteroid dehydrogenase like 1 | 2 | | o | | | | o | | |
| im:6894757 protein | 2 | o | | | | | | | |
| immediate early response 5-like | 2 | o | | | | | | | |
| immunoglobulin member 9 | 2 | | | o | | | | | |
| immunoglobulin member isoform cra_a | 2 | o | | | | | o | | |
| immunoglobulin mu binding protein 2 | 2 | o | | | | | | | |
| importin 11 | 2 | o | | | | | o | | |
| importin 5 | 2 | | | o | | o | | | |
| importin subunit alpha-3 | 2 | o | | | | | | | |
| inactive dual specificity phosphatase 27 | 2 | o | | | | | | | |
| inactive serine protease 35 precursor | 2 | o | | o | | | | | |
| influenza virus ns1a-binding protein homolog a | 2 | | | o | | | | o | |
| ing defective family member (mlt-10)- partial | 2 | | | | o | | | | |
| inhibitor of bruton tyrosine kinase | 2 | | | | | | | | o |
| inhibitor of growth protein 4 | 2 | o | | o | | | | | |
| ino80 complex subunit d | 2 | o | o | | | | | | |
| inosine-5 -monophosphate dehydrogenase 2 | 2 | | | | | | | | o |
| inositol -1(or 4)-monophosphatase 1 | 2 | o | | | | | | | o |
| inositol monophosphatase 1-like | 2 | | | | | | | | o |
| insulin receptor partial | 2 | | | | o | | | | |
| insulin receptor substrate 1-like | 2 | o | | | | | | | |
| integral membrane protein 2c-like | 2 | | | | | | | | o |
| integral membrane protein gpr137b-like | 2 | o | | | | | | | o |
| integrator complex subunit 3 | 2 | o | o | | | | | | |
| integrator complex subunit 9 | 2 | | | | o | | o | | |
| integrin alpha-5 | 2 | o | | | | | | | |
| integrin-linked protein kinase | 2 | o | | o | | | | | |
| intelectin 2 | 2 | | o | | | | | | |
| interacting protein 2 | 2 | o | | | | o | | | |
| interferon regulatory factor 2-binding protein 2-a | 2 | | o | | | | | | o |
| interferon regulatory factor 2-binding protein 2-b | 2 | o | | | | o | | | |
| interferon regulatory factor 3 | 2 | o | | o | | | | | |
| interferon-induced protein with tetratricopeptide repeats 1 | 2 | | | | o | | | | |
| interleukin 13 alpha 2 | 2 | o | | | | | | | |
| interleukin 18 | 2 | o | | | | | | | |
| interleukin 6 receptor | 2 | | o | | | | | | |
| interleukin 6 signal transducer ( oncostatin m receptor) | 2 | | o | | | | o | | |
| interleukin-17N [Takifugu rubripes] | 2 | o | | | | | o | | |
| interleukin-6 receptor subunit alpha precursor | 2 | o | o | | | | | | |
| intersectin 1 (sh3 domain protein) | 2 | o | | | | | o | | |
| intraflagellar transport 52 homolog | 2 | o | | | | | | | |
| intraflagellar transport protein 43 homolog | 2 | | | | o | | | | o |
| intron-binding protein aquarius | 2 | | | | | | o | | o |
| iodotyrosine deiodinase | 2 | | o | | | | | | |
| iq motif and sec7 domain-containing protein 1 | 2 | o | | | | | o | | |

| | | | | | | | | | |
|---|---|---|---|---|---|---|---|---|---|
| iq motif and sec7 domain-containing protein 3 | 2 | | | | o | | o | | |
| islet amyloid polypeptide | 2 | o | o | | | | | | |
| isocitrate dehydrogenase 3 (nad+) gamma | 2 | o | o | | | | | | |
| ist1 homolog | 2 | o | | | | | | | o |
| josephin-2 | 2 | o | | o | | | | | |
| jumonji domain containing 1c | 2 | o | | | | | | | |
| jumonji domain containing 2b | 2 | | | o | | | | o | |
| junctophilin 1 | 2 | o | | | | | | | |
| k0090 protein | 2 | o | | | | | o | | |
| kaiso-like zinc finger protein | 2 | | | | | | o | o | |
| karyopherin alpha 3 | 2 | o | | | | | | | |
| katanin p60 atpase-containing subunit a-like 1 | 2 | | | | | | | | o |
| k-cl cotransporter | 2 | o | | | | o | | | |
| kelch domain containing 3 | 2 | o | | | | | o | | |
| kelch repeat and btb domain containing 10 | 2 | o | | | | | | | |
| kelch repeat and btb domain containing 5 | 2 | o | | | | | | | |
| kelch-like 13 | 2 | o | | | | | o | | |
| kelch-like 23 | 2 | o | | | | | | | |
| kelch-like protein 30 | 2 | o | | | | | | | |
| keratin-like protein krt222 | 2 | o | | | | | | | |
| keratinocyte growth factor | 2 | | | o | | | | | |
| ketohexokinase | 2 | o | | | | | | | |
| kh domain- rna- signal transduction-associated protein 1 | 2 | | | | | | o | | o |
| kiaa0310 protein | 2 | o | | | | | | | |
| kiaa0513 protein | 2 | | | | | o | | | |
| kiaa0528 protein | 2 | o | o | | | | | | |
| kiaa0614 protein | 2 | | o | | | | o | | |
| kiaa0649 protein | 2 | o | | o | | | | | |
| kiaa0913 protein | 2 | o | o | | | | | | |
| kiaa1143 homolog | 2 | o | | o | | | | | |
| kiaa1250 protein | 2 | | | | | | o | | |
| kiaa2005 protein | 2 | o | | | | | | | |
| kif1-binding protein | 2 | | | | | o | o | | |
| kinectin 1 | 2 | | o | | | | | o | |
| kinesin family member 15 | 2 | o | | | | | o | | |
| kinesin family member 2c | 2 | o | o | | | | | | |
| kinesin family member 5c | 2 | | | | | | o | o | |
| kinesin heavy chain isoform 5c | 2 | | | | | | | | o |
| kinesin light chain 1 | 2 | | | o | | | | | o |
| kinesin-1 heavy chain | 2 | | o | | | | o | | |
| kinesin-associated protein 3 | 2 | | | | | | o | | o |
| kinesin-like protein | 2 | o | | | | | | | o |
| kinesin-like protein kif14 | 2 | | | | o | | | | |
| kinesin-like protein kif1a-like | 2 | | | | | | o | | |
| kinesin-like protein kif22 | 2 | | o | | | | o | | |
| kinesin-like protein kif2a isoform 1 | 2 | | | | | o | | | o |
| kinesin-like protein surhe | 2 | | | | o | | | | |
| kininogen-1 isoform 2 | 2 | | | | o | | | | |
| kininogen-1 precursor | 2 | | o | | o | | | | |
| kn motif and ankyrin repeat domain-containing protein 1 | 2 | | o | | o | | | | |
| krr1 small subunit processome component homolog | 2 | | | | | o | | | o |
| kruppel-like factor 2 | 2 | o | | | | | | | |
| kti12 homolog | 2 | o | | | | | o | | |
| la ribonucleoprotein domain member 4 protein | 2 | | | | | | o | o | |
| lamina-associated polypeptide isoforms beta delta epsilon gamma-like | 2 | | | | o | | | | |
| lantibiotic synthetase component c-like 1 | 2 | | o | | | o | | | |

| | | | | | | | | | |
|---|---|---|---|---|---|---|---|---|---|
| la-related protein 4 | 2 | o | | o | | | | | |
| la-related protein 7 | 2 | o | | | | | o | | |
| large subunit gtpase 1 homolog | 2 | o | | | o | | | | |
| large subunit ribosomal protein 23 | 2 | | | | o | | | | o |
| las1-like ( cerevisiae) | 2 | o | | | | o | | | |
| latent transforming growth factor beta binding protein 3 | 2 | o | | | | | | | |
| lathosterol oxidase | 2 | o | | | | | | | |
| latrophilin 3 | 2 | | | | | | o | | |
| latrophilin-2-like isoform 2 | 2 | | | | | | o | o | |
| lchn protein | 2 | | | | | o | | o | |
| ldb1a protein | 2 | o | | | | | | | |
| ldlr chaperone mesd-like | 2 | | | | o | | | | o |
| lecithin retinol acyltransferase-like | 2 | | o | | | | | | o |
| leucine aminopeptidase 3 | 2 | o | | | | | | | |
| leucine- glioma inactivated 1a | 2 | | | | | o | o | | |
| leucine rich repeat (in flii) interacting protein 1 | 2 | o | | | | | | | |
| leucine rich repeat and fibronectin type iii domain containing 5 | 2 | | | | | | o | | |
| leucine rich repeat containing 4 | 2 | | | | | | o | | o |
| leucine rich repeat containing 7 | 2 | | | | | | o | | |
| leucine rich repeat neuronal 1 | 2 | | | | | o | | | |
| leucine rich repeat neuronal 3 | 2 | o | | | | | o | | |
| leucine rich repeat neuronal isoform cra_b | 2 | | | | o | o | | | |
| leucine zipper and ctnnbip1 domain containing | 2 | o | | | | | | | |
| leucine zipper transcription factor-like 1 | 2 | o | | | | o | | | |
| leucine-rich alpha-2-glycoprotein | 2 | | o | | o | | | | |
| leucine-rich repeat and immunoglobulin-like domain-containing nogo receptor-interacting protein 3-like | 2 | | | | | | o | o | |
| leucine-rich repeat transmembrane neuronal protein 1-like | 2 | o | | | | o | | | |
| leucine-rich repeat-containing g-protein coupled receptor 6 | 2 | | | | | | | | o |
| leucine-rich repeat-containing protein 33 precursor | 2 | o | o | | | | | | |
| leucine-rich repeat-containing protein 69 | 2 | | | | o | | | | |
| leucine-rich repeat-containing protein 7-like | 2 | | | | | | o | o | |
| leucine-rich tropomodulin and proline-rich containing protein | 2 | | | | | | o | o | |
| leucyl cystinyl aminopeptidase | 2 | o | | | | | o | | |
| leucyl-trna synthetase | 2 | | o | | | | | | |
| leukocyte cell-derived chemotaxin 2 precursor | 2 | | o | o | | | | | |
| leukocyte receptor cluster member 8 | 2 | o | | | | | o | | |
| leukocyte surface protein precursor | 2 | o | | | | | | | |
| leukotriene a-4 hydrolase | 2 | o | | | | | | | |
| ligand dependent nuclear receptor corepressor-like | 2 | o | | | | | o | | |
| ligatin | 2 | o | | | | | | | |
| light chain alkali embryonic | 2 | o | | | | | | | |
| lim and calponin homology domains-containing protein 1 | 2 | o | o | | | | | | |
| lim and sh3 domain protein 1 | 2 | | | | | o | | o | |
| lim domain and actin-binding protein 1-like | 2 | | | | o | | | | o |
| lim domain kinase 1 | 2 | o | | | | | o | | |
| limb and neural patterns | 2 | o | | o | | | | | |
| lin-37 homolog | 2 | o | | | | | | o | |
| lipase lysosomal cholesterol esterase | 2 | | o | o | | | | | |

| | | | | | | | | | |
|---|---|---|---|---|---|---|---|---|---|
| lipase maturation factor 2 | 2 | o | o | | | | | | |
| lipin 1 | 2 | o | | | | | | | |
| lipin 2 | 2 | o | | | | | o | | |
| lipopolysaccharide-responsive and beige-like anchor protein | 2 | | | | | | | | o |
| lissencephaly-1 homolog | 2 | | | | | | o | | |
| lissencephaly-1 homolog a | 2 | | | | | | | o | |
| liver carboxylesterase 2-like | 2 | | o | o | | | | | |
| liver-expressed antimicrobial peptide 2 | 2 | | o | o | | | | | |
| lman2l protein | 2 | o | | | | | | | |
| loc100002393 protein | 2 | | | | | o | o | | |
| loc100137634 protein | 2 | o | o | | | | | | |
| loc361985 protein | 2 | o | | o | | | | | |
| loc398446 protein | 2 | o | | | | o | | | |
| loc443676 protein | 2 | o | | | | | | | |
| loc445837 protein | 2 | | | | o | | | | |
| loc553425 protein | 2 | | | | | o | o | | |
| loc556764 protein | 2 | | o | | | | | | |
| loc565172 protein | 2 | | | | | | | | o |
| loc568230 protein | 2 | o | | | | o | | | |
| loc733342 protein | 2 | o | o | | | | | | |
| loc792676 protein | 2 | o | | | | o | | | |
| loc798996 protein | 2 | | | | o | | | | |
| lon peptidase n-terminal domain and ring finger 2 | 2 | o | o | | | | | | |
| long-chain-fatty-acid-- ligase 1 | 2 | | o | | | | | o | |
| low density lipo protein 1b (deleted in tumors) | 2 | | | | | | o | | o |
| low quality protein: glucoside xylosyltransferase 1-like | 2 | | | | o | | | | o |
| low quality protein: gon-4-like | 2 | | | | | | | | o |
| low quality protein: lysine-specific demethylase 5b-like | 2 | o | | | | | o | | |
| low quality protein: neurogenic locus notch homolog protein 3-like | 2 | o | | | | | | | |
| low quality protein: xin actin-binding repeat-containing protein 2-like | 2 | o | | | | | | | |
| lrr and pyd domains-containing protein 12-like | 2 | | o | | | | o | | |
| lrr and pyd domains-containing protein 14-like | 2 | | o | | | | | | o |
| lsm2 u6 small nuclear rna associated ( cerevisiae) | 2 | o | | | | o | | | |
| lsm4 u6 small nuclear rna associated ( cerevisiae) | 2 | | | | o | | | | o |
| lsm7 u6 small nuclear rna associated ( cerevisiae) | 2 | | o | | | o | | | |
| ltv1 homolog ( cerevisiae) | 2 | o | | | | o | | | |
| luc7-like protein 3 | 2 | o | | o | | | | | |
| lumican | 2 | o | o | | | | | | |
| ly6 plaur domain containing 1 | 2 | | | | | o | | o | |
| lymphocyte g0 g1 switch protein 2 | 2 | o | | | | o | | | |
| lyr motif-containing protein 2 | 2 | o | o | | | | | | |
| lysm and peptidoglycan-binding domain-containing protein 3 | 2 | | o | | o | | | | |
| lysophosphatidic acid receptor 4 | 2 | | | | | | | | o |
| lysophosphatidylcholine acyltransferase 3 | 2 | o | | | | | | | |
| lysosomal protective protein precursor | 2 | | | | o | | | | |
| lysozyme c ii precursor | 2 | | o | o | | | | | |
| lysozyme g | 2 | o | | | | | | | |

| | | | | | | | | | |
|---|---|---|---|---|---|---|---|---|---|
| lysyl oxidase | 2 | o | o | | | | | | |
| lysyl oxidase-like 2 | 2 | o | | | | | | | |
| lysyl oxidase-like 3 | 2 | o | | | | | | | |
| macrophage mannose receptor 1-like | 2 | | | | o | | | | |
| mad2 mitotic arrest deficient-like 1 | 2 | o | o | | | | | | |
| magnesium transporter 1 | 2 | o | | o | | | | | |
| major facilitator superfamily domain containing 11 | 2 | | o | o | | | | | |
| major facilitator superfamily domain-containing protein 6 | 2 | o | | | | | o | | |
| malate synthase | 2 | | o | o | | | | | |
| malic enzyme nad(+)- mitochondrial | 2 | o | | | | | | | |
| mam domain containing glycosylphosphatidylinositol anchor 1 | 2 | | | | | | o | o | |
| mammalian ependymin-related protein 1 precursor | 2 | o | | | | | o | | |
| mammalian ependymin-related protein 1-like | 2 | | | | | | | | o |
| mannan-binding lectin serine protease 2 | 2 | | o | | | | | o | |
| mannose receptor c1-like protein | 2 | o | | | | | | | |
| mannose-1-phosphate guanyltransferase beta | 2 | | | o | o | | | | |
| mannose-specific lectin precursor | 2 | o | | | | | o | | |
| mannosyl (alpha- -)-glycoprotein beta- -n-acetylglucosaminyltransferase | 2 | o | o | | | | | | |
| mannosyl-oligosaccharide glucosidase-like | 2 | | | | o | | | | |
| map kinase-activated protein kinase 5 | 2 | o | | | | | | | |
| map kinase-activating death domain | 2 | | | | | | o | | |
| map7 domain containing 1 | 2 | | o | | | | o | | |
| mapk activating protein | 2 | o | | | | | | | |
| mapk mak mrk overlapping kinase | 2 | | | | | | | o | o |
| maspardin-like isoform 1 | 2 | | | | o | | | | |
| matrin 3 | 2 | o | | o | | | | | |
| matrin 3-like | 2 | o | | | | | o | | |
| matrix-remodeling-associated protein 8 | 2 | o | | | | | | | |
| mediator of rna polymerase ii transcription subunit 17 | 2 | o | | | | | | | o |
| mediator of rna polymerase ii transcription subunit 18 | 2 | | | o | o | | | | |
| mediator of rna polymerase ii transcription subunit 20 | 2 | | | | | o | | o | |
| mediator of rna polymerase ii transcription subunit 25 | 2 | o | | | | | | o | |
| mediator of rna polymerase ii transcription subunit 26 | 2 | o | | | | | | | |
| melanocortin 5 receptor | 2 | | | | | | o | | |
| membrane frizzled-related protein | 2 | | o | | o | | | | |
| membrane metallo-endopeptidase-like 1 | 2 | | | | | o | o | | |
| membrane protein fam174b precursor | 2 | o | o | | | | | | |
| membrane-associated progesterone receptor component 1 | 2 | | o | | | | | | o |
| membrane-spanning 4-domains subfamily a member 15 | 2 | o | | | | | | | |
| membrane-spanning 4-domains subfamily a member 4d | 2 | o | | | | o | | | |
| mesenchymal stem cell protein dscd75 precursor | 2 | o | | o | | | | | |
| met proto-oncogene precursor | 2 | | | o | | | | | |
| metabotropic glutamate receptor 8-like | 2 | | | | | | o | | |
| metallo-beta-lactamase superfamily | 2 | o | | | | | | | |
| metalloreductase steap4 | 2 | o | o | | | | | | |
| metastasis suppressor protein 1 | 2 | o | | | | | | o | |

| | | | | | | | | | |
|---|---|---|---|---|---|---|---|---|---|
| metaxin 1 | 2 | o | o | | | | | | |
| metaxin 3 | 2 | | | | | | o | o | |
| methenyltetrahydrofolate synthetase domain containing | 2 | o | o | | | | | | |
| methenyltetrahydrofolate synthetase domain-containing protein | 2 | o | | o | | | | | |
| methionine sulfoxide reductase b2 | 2 | | o | | | | | | o |
| methylenetetrahydrofolate dehydrogenase (nadp+ dependent) methenyltetrahydrofolate formyltetrahydrofolate synthetase | 2 | | o | | | | | | |
| methylmalonic aciduria (cobalamin deficiency) cblb type | 2 | o | | | | o | | | |
| methylmalonyl epimerase | 2 | o | o | | | | | | |
| methylosome protein 50 | 2 | o | | | | | | | o |
| methyltransferase kiaa1456-like | 2 | | | | o | | | | o |
| methyltransferase like 11b | 2 | o | | | | | | | |
| methyltransferase like 12 | 2 | o | | | | o | | | |
| methyltransferase-like protein 13 | 2 | o | | | | | | | |
| methyltransferase-like protein 2 | 2 | | o | o | | | | | |
| methyltransferase-like protein 7a-like | 2 | | | | o | | | | o |
| mflj00348 protein | 2 | o | | | | o | | | |
| mgc83858 protein | 2 | o | | | | | | | |
| mhd domain-containing death-inducing protein | 2 | o | | | | | | | |
| microfibril-associated glycoprotein 4 precursor | 2 | | o | o | | | | | |
| microfibrillar-associated protein 2 | 2 | o | | | | | | | |
| microfibrillar-associated protein 3-like | 2 | | | o | o | | | | |
| microfibrillar-associated protein 4-like | 2 | | | o | | | | | |
| microsomal triglyceride transfer protein | 2 | | o | | | | | | |
| microsomal triglyceride transfer protein large subunit | 2 | | | o | | | | | |
| microtubule-associated protein 1a-like | 2 | | | | | | | o | |
| microtubule-associated protein rp eb family member 1 | 2 | | | | | | o | | o |
| microtubule-associated protein rp eb family member 3-like | 2 | | | | | | o | | o |
| mit domain-containing protein 1-like | 2 | | | | | | | | o |
| mitochondrial 28s ribosomal protein s25 | 2 | | o | | | o | | | |
| mitochondrial carnitine acylcarnitine carrier protein | 2 | o | | | | o | | | |
| mitochondrial carrier triple repeat 1 | 2 | | | o | | | | o | |
| mitochondrial fission factor-like | 2 | o | | | | | | | o |
| mitochondrial folate transporter carrier | 2 | o | o | | | | | | |
| mitochondrial gtpase 1 homolog ( cerevisiae) | 2 | | o | o | | | | | |
| mitochondrial gtpase 1 precursor | 2 | o | | | | | | | |
| mitochondrial h+-transporting atp synthase f1 complex alpha subunit 1 | 2 | | | | o | | | | o |
| mitochondrial import inner membrane translocase subunit tim16 | 2 | | o | | | o | | | |
| mitochondrial import inner membrane translocase subunit tim9 b | 2 | o | o | | | | | | |
| mitochondrial import receptor subunit tom34 | 2 | o | | | | | o | | |
| mitochondrial intermembrane space import and assembly protein 40 | 2 | | o | | o | | | | |
| mitochondrial lon protease-like protein | 2 | | | o | | | o | | |
| mitochondrial ribosomal protein l11 | 2 | o | o | | | | | | |
| mitochondrial ribosomal protein l13 | 2 | o | | o | | | | | |
| mitochondrial ribosomal protein l18 | 2 | | o | | | o | | | |
| mitochondrial ribosomal protein l22 | 2 | | | | o | | | | |

| | | | | | | | | | |
|---|---|---|---|---|---|---|---|---|---|
| mitochondrial ribosomal protein l35 | 2 | | o | o | | | | | |
| mitochondrial ribosomal protein l44 | 2 | | o | | o | | | | |
| mitochondrial ribosomal protein s26 | 2 | o | o | | | | | | |
| mitochondrial ribosomal protein s27 | 2 | o | o | | | | | | |
| mitochondrial ribosomal protein s5 | 2 | o | o | | | | | | |
| mitochondrial thiamine pyrophosphate carrier | 2 | | | | | | | | o |
| mitochondrial trans-2-enoyl- reductase | 2 | o | | | | | | | |
| mitochondrial transcription termination factor | 2 | o | | | | | | | |
| mitochondrial uncoupling protein 2 | 2 | o | | | | | | | |
| mitochondrial-processing peptidase subunit beta-like | 2 | | | | | | o | | o |
| mitofusin 2 | 2 | o | | | | | o | | |
| mitogen-activated protein kinase 10 | 2 | | | | | | o | o | |
| mitogen-activated protein kinase 8 interacting protein 2 | 2 | | | | | | o | o | |
| mitogen-activated protein kinase kinase 1 | 2 | | | | | o | o | | |
| mitogen-activated protein kinase kinase 4 | 2 | | | o | | | | o | |
| mitogen-activated protein kinase kinase kinase kinase 3 | 2 | o | | | | | | | |
| mitogen-activated protein kinase-activated protein kinase 3 | 2 | o | | | | | | | |
| mitotic-spindle organizing protein 1 | 2 | o | | | | | | o | |
| mixed-lineage leukemia-like protein | 2 | o | | | | | o | | |
| mll1 mll complex subunit c17orf49-like protein | 2 | o | o | | | | | | |
| mon2 protein | 2 | o | | | | | | o | |
| monocyte to macrophage differentiation-associated 2 | 2 | | o | | | | | | |
| mothers against decapentaplegic homolog 3 | 2 | o | | | | | o | | |
| mothers against decapentaplegic homolog 4 | 2 | | | | | | o | | |
| mps one binder kinase activator-like 1b | 2 | o | | | | | | | |
| mps one binder kinase activator-like 2c | 2 | o | | | | | | | o |
| mrna export factor | 2 | o | | | | | | o | |
| msx2-interacting protein | 2 | | | | o | | o | | |
| mterf domain-containing protein mitochondrial | 2 | | | | o | | | | o |
| mterf domain-containing protein mitochondrial precursor | 2 | o | | | | o | | | |
| mucolipin 2 | 2 | | o | | | | | | |
| multifunctional protein ade2 | 2 | | o | | | o | | | |
| multiple egf-like-domains 8 | 2 | | | | | | o | | |
| multiple inositol polyphosphate histidine 1 | 2 | | o | | | | | | |
| multiple myeloma tumor-associated protein 2 homolog | 2 | o | | | o | | | | |
| multisynthetase complex auxiliary component p38 | 2 | o | | o | | | | | |
| muscleblind-like protein 1-like | 2 | o | | | | | | | |
| muted protein | 2 | o | | | | o | | | |
| myelin and lymphocyte protein | 2 | o | | o | | | | | |
| myeloid cell leukemia sequence 1b | 2 | | | | | o | | o | |
| myeloid-associated differentiation marker-like protein 2 | 2 | o | | | | | | | |
| myeloma-overexpressed gene 2 protein homolog | 2 | | | | | | | | o |
| myo-inositol 1-phosphate synthase a1 | 2 | o | | | | | | | o |
| myopalladin | 2 | o | | | | | | | |
| myosin ie | 2 | o | | | | | o | | |
| myosin light chain 2 | 2 | o | | | | | | | |
| myosin light chain 3 | 2 | o | | | | | | | |
| myosin light chain kinase | 2 | o | | | | | | | |

| | | | | | | | | | |
|---|---|---|---|---|---|---|---|---|---|
| myosin regulatory light chain ventricular cardiac muscle isoform | 2 | o | | | | | | | |
| myosin x | 2 | | | | | | o | | |
| myosin xviii-like 1 | 2 | o | | | | | | | |
| myosin-ib isoform 1 | 2 | | o | | | | o | | |
| n -(beta-n-acetylglucosaminyl)-l-asparaginase-like | 2 | | | | o | | | | |
| na+ k+ alpha 1b polypeptide | 2 | | | | | | o | o | |
| n-acetylgalactosaminyltransferase 7 | 2 | o | | | | | | | |
| n-acetylglucosamine-6-phosphate deacetylase | 2 | o | o | | | | | | |
| n-acetylglucosaminyl-phosphatidylinositol de-n-acetylase | 2 | o | | o | | | | | |
| n-acetylneuraminate lyase | 2 | o | | | | o | | | |
| n-acetylneuraminic acid synthase | 2 | | | | | o | | o | |
| n-acetyltransferase esco1 | 2 | | o | o | | | | | |
| nadh dehydrogenase subunit 3 | 2 | | o | | | o | | | |
| nag-5 protein | 2 | | | | | | o | o | |
| n-alpha-acetyltransferase catalytic subunit | 2 | o | | | | | o | | |
| nasal embryonic lhrh factor | 2 | | | | | o | | o | |
| nbeal1 protein | 2 | o | | | | | | | |
| necdin-like 2 | 2 | o | | | | | | | |
| nedd4 family interacting protein 2 | 2 | | | o | | | | o | |
| nedd4 family-interacting protein 2 | 2 | | o | | | | o | | |
| nedd8 activating enzyme e1 subunit 1 | 2 | o | | o | | | | | |
| nedd8-conjugating enzyme ube2f | 2 | o | | | | | | o | |
| nefa-interacting nuclear protein nip30 | 2 | o | | | | | o | | |
| nef-associated protein 1 | 2 | o | | o | | | | | |
| nef-associated protein 1-like | 2 | o | | | | o | | | |
| neighbor of brca1 gene 1 | 2 | | o | | | | | | |
| n-ethylmaleimide-sensitive factor attachment alpha | 2 | | o | o | | | | | |
| n-ethylmaleimide-sensitive factor attachment beta | 2 | o | | | | | | o | |
| neugrin | 2 | o | | o | | | | | |
| neural cell surface protein f3 | 2 | | | | | | | | o |
| neural precursor cell developmentally down-regulated 4-like | 2 | | o | | | | o | | |
| neural proliferation differentiation and control protein 1 precursor | 2 | o | | | | o | | | |
| neuralized-like protein 2 | 2 | o | | | | | | | |
| neurensin 1 | 2 | | | | | | o | o | |
| neurexin 3b | 2 | | | | | | o | o | |
| neurobeachin | 2 | | | | | | o | | o |
| neuroblastoma-amplified sequence | 2 | | | | | | | | o |
| neurocan core protein precursor | 2 | | | | | | | o | |
| neuroendocrine convertase 2 precursor | 2 | | | | | o | | o | |
| neuroendocrine protein 7b2 | 2 | | | | | | o | | o |
| neurofascin homolog | 2 | o | | | | | | o | |
| neurofibromin isoform 2 | 2 | o | | | | | o | | |
| neurofilament heavy polypeptide | 2 | | | | | | | o | o |
| neurofilament light polypeptide | 2 | | | | | | o | | o |
| neurogenic locus notch homolog protein 3 precursor | 2 | | | | | | o | o | |
| neuroligin 3b | 2 | | | | | | o | | |
| neuronal pentraxin 1 | 2 | o | | | | | | o | |
| neuronal pentraxin-1-like | 2 | | | | | | | | o |
| neuron-specific protein family member 2-like | 2 | | | | | | | | o |
| neuropilin 2b | 2 | o | | | | | o | | |
| neurotrophic tyrosine type 2 | 2 | | | | | | o | | |
| neutral ceramidase | 2 | | o | | | | | | |

| | | | | | | | | | |
|---|---|---|---|---|---|---|---|---|---|
| neutrophil cytosol factor 2-like | 2 | | | | o | | | | |
| nexilin | 2 | o | | | | | | | |
| next to brca1 gene 1 protein | 2 | | o | | | | | | o |
| nf-kappa-b inhibitor epsilon | 2 | o | | | o | | | | |
| nhs-like 2 | 2 | | | | | o | | o | |
| nhs-like protein 2-like | 2 | | | | | | o | | |
| nicolin 1 | 2 | o | | | | o | | | |
| nicotinamide mononucleotide adenylyltransferase 2 | 2 | | | | | | o | o | |
| nicotinamide phosphoribosyltransferase | 2 | o | | o | | | | | |
| nif3-like protein 1 | 2 | o | o | | | | | | |
| nima (never in mitosis gene a)-related kinase 7 | 2 | | | o | | | | | |
| ninjurin 2 | 2 | | o | | | o | | | |
| nipsnap homolog 3a | 2 | o | o | | | | | | |
| n-myc (and stat) interactor | 2 | o | | | | | | | o |
| nodal modulator 2 | 2 | o | | | | | | | |
| non-metastatic cells protein expressed in | 2 | | o | o | | | | | |
| non-pou domain-containing octamer-binding protein | 2 | | | | o | | | | o |
| normal mucosa of esophagus-specific gene 1 protein | 2 | o | | | | | | | o |
| norrin-like | 2 | | | | o | | | | |
| notch gene homolog 1 | 2 | | | | | | o | o | |
| notchless homolog 1 | 2 | o | | | | | | | o |
| novel immune type receptor-like | 2 | o | | o | | | | | |
| novel nacht domain containing | 2 | | | o | | | | | |
| novel protein (wu:fb14b05) | 2 | | o | | | o | | | |
| novel protein (zgc:112490) | 2 | o | | o | | | | | |
| novel protein (zgc:158450) | 2 | | | | | | o | o | |
| novel protein (zgc:56258) | 2 | o | o | | | | | | |
| novel protein ca++ plasma membrane (zgc:92885) | 2 | | | | | | o | o | |
| novel protein containing tubulin-binding domains (zgc:103474) | 2 | | | | | | o | | |
| novel protein human large homolog-associated protein 2 | 2 | | | | | | o | | |
| novel protein kiaa1310 ( zgc:109953) | 2 | | o | o | | | | | |
| novel protein myosin ib | 2 | o | | | | | | | o |
| novel protein piccolo (presynaptic cytomatrix protein) | 2 | o | | | | | | o | |
| novel protein sine oculis binding protein homolog | 2 | o | | | | | | | |
| novel protein vertebrate ankyrin repeat domain 15 | 2 | o | | o | | | | | |
| novel protein vertebrate bruno-like rna binding protein family | 2 | | | | | | o | o | |
| novel protein vertebrate cub and sushi multiple domain containing protein family | 2 | | | | | | o | | |
| novel protein vertebrate dab2 interacting protein | 2 | | | | | | o | | |
| novel protein vertebrate gamma-aminobutyric acid b 2 | 2 | | o | | | | o | | |
| novel protein vertebrate granzyme family | 2 | o | | | | | | | |
| novel protein vertebrate greb1 protein | 2 | o | | o | | | | | |
| novel protein vertebrate lim and calponin homology domains 1 | 2 | o | | | | | | | |
| novel protein vertebrate map microtubule affinity-regulating kinase 3 ( zgc:153725) | 2 | o | | | | | | | o |
| novel protein vertebrate periphilin 1 | 2 | | o | | | | | | o |

| | | | | | | | | | |
|---|---|---|---|---|---|---|---|---|---|
| novel protein vertebrate protein phosphatase 2 (formerly 2a) regulatory subunit b family | 2 | o | | | | | | | |
| novel protein vertebrate ubiquitin associated protein 2-like | 2 | o | | o | | | | | |
| novel protein vertebrate udp-glycosyltransferase family | 2 | | o | | | | | | |
| novel protein vertebrate yip1 interacting factor homolog b ( cerevisiae) ( zgc:103562) | 2 | | o | | | | o | | |
| novel protein with a scavenger receptor cysteine-rich domain | 2 | | | | | | | | o |
| novel protein with zona pellucida-like domain | 2 | o | | | o | | | | |
| novel rho gtpase activation protein | 2 | o | | | | | | o | |
| novel tetraspanin family protein | 2 | | | | | o | | | o |
| nt-3 growth factor receptor-like isoform 2 | 2 | | | | | | | o | |
| ntd5 protein | 2 | o | | | | o | | | |
| ntf2-related export protein 2 | 2 | | | | o | o | | | |
| nuak snf1-like 2 | 2 | | o | | | | | | |
| nuclear autoantigenic sperm | 2 | | | | o | | | | o |
| nuclear distribution gene c homolog ( nidulans) | 2 | | | | o | | | | o |
| nuclear distribution protein nude-like 1-like | 2 | o | | | | | o | | |
| nuclear factor 1 x-type-like | 2 | | | o | | | o | | |
| nuclear factor i isoform cra_c | 2 | | | o | | | o | | |
| nuclear factor i x-like | 2 | | | o | | | | o | |
| nuclear factor interleukin-3-regulated protein | 2 | o | o | | | | | | |
| nuclear factor of kappa light polypeptide gene enhancer in b-cells zeta | 2 | | o | | | | | | |
| nuclear factor related to kappa-b-binding protein | 2 | o | | | | | | o | |
| nuclear prelamin a recognition factor | 2 | o | | | | | o | | |
| nuclear receptor binding protein | 2 | | | | | o | o | | |
| nuclear receptor coactivator 1 | 2 | o | | | | | o | | |
| nuclear receptor coactivator 4 | 2 | o | o | | | | | | |
| nuclear receptor coactivator 5 | 2 | o | o | | | | | | |
| nuclear receptor coactivator 7-like | 2 | | | | | | o | | |
| nuclear receptor co-repressor 1 | 2 | o | | | | | o | | |
| nuclear receptor subfamily 0 group b member 2 | 2 | | o | | | | | | |
| nuclear receptor-binding protein 2 | 2 | | | | | | o | | |
| nuclear transcription factor y subunit beta | 2 | o | | o | | | | | |
| nuclear transcription x-box binding 1 | 2 | o | | | | | | o | |
| nucleobindin-1 precursor | 2 | | | | | | | | o |
| nucleolar and coiled-body phosphoprotein 1 | 2 | o | | | | | o | | |
| nucleolar complex protein 2 homolog | 2 | o | | | | | o | | |
| nucleolar gtp-binding protein 1 | 2 | | | | | | | | o |
| nucleolar gtp-binding protein 2 | 2 | | | | | o | o | | |
| nucleolar pre-rrna processing homolog ( cerevisiae) | 2 | o | | | | o | | | |
| nucleolar protein 11-like | 2 | o | o | | | | | | |
| nucleolar protein 7-like | 2 | | | | o | | | | |
| nucleophosmin | 2 | | | | o | | | | |
| nucleophosmin 3 | 2 | o | | | | o | | | |
| nucleophosmin-like isoform 1 | 2 | o | | | | | | | |
| nucleoporin 155kda | 2 | o | | | | | | | |
| nucleoporin 62kda | 2 | | o | | | | | o | |
| nucleoporin seh1 | 2 | o | | | | | | | o |
| nucleoprotein tpr-like | 2 | o | | | | | | | |
| nucleoside diphosphate kinase b | 2 | o | | | | | | | |
| nucleoside diphosphate mitochondrial | 2 | | | | o | | | | |

| | | | | | | | | | |
|---|---|---|---|---|---|---|---|---|---|
| nucleoside diphosphate-linked moiety x motif mitochondrial-like | 2 | o | | | | | | | |
| nudix (nucleoside diphosphate linked moiety x)-type motif 15 | 2 | o | | | | o | | | |
| ocia domain-containing protein 2 | 2 | o | o | | | | | | |
| olfactomedin 4 | 2 | o | | | | | | | o |
| olfactomedin-like protein 2b precursor | 2 | | | | | | o | o | |
| olfactory receptor 226-like | 2 | o | | | | | | | |
| oligosaccharyl transferase | 2 | o | | | | | | | |
| opioid growth factor receptor | 2 | o | | | | | | | |
| orf1ab polyprotein | 2 | | | o | | | o | | |
| organic solute transporter subunit alpha-like | 2 | | | | o | | | | |
| ornithine decarboxylase antizyme 2 | 2 | o | | | | | | o | |
| osteoclast stimulating factor 1 | 2 | o | | o | | | | | |
| otoraplin | 2 | | | | | o | | o | |
| oxidation resistance 1 | 2 | | | | | | o | o | |
| oxidation resistance protein 1 | 2 | | | | | o | o | | |
| oxidative-stress responsive 1 | 2 | o | | | | | o | | |
| oxidoreductase htatip2 | 2 | | | | o | | | | o |
| oxysterol binding 2 | 2 | | o | | | | | | |
| oxytocin receptor | 2 | o | | | | | | o | |
| p140 gene | 2 | | | | | | o | o | |
| p21-activated protein kinase-interacting protein 1-like | 2 | | | | o | | | | |
| p300 cbp-associated factor | 2 | o | | | | | | o | |
| p4hb protein | 2 | o | | o | | | | | |
| p53 and dna damage-regulated protein 1 | 2 | o | | | | o | | | |
| pa2g4 protein | 2 | | | | | o | | | |
| paired amphipathic helix protein sin3a isoform 1 | 2 | o | | | | | | | |
| pallidin | 2 | o | | o | | | | | |
| pancreatic alpha-amylase | 2 | o | o | | | | | | |
| pancreatic progenitor cell differentiation and proliferation factor b | 2 | | o | | o | | | | |
| pantothenate kinase 1 | 2 | | o | | | | | o | |
| parkinson disease 7 domain-containing protein 1 precursor | 2 | o | | | | o | | | |
| parp1-binding protein | 2 | | | | o | | | | |
| parvalbumin alpha | 2 | | | | | | o | | o |
| patatin-like phospholipase domain-containing protein 4 | 2 | | o | | | | | | o |
| pbx knotted 1 homeobox 1 | 2 | o | | o | | | | | |
| pci domain-containing protein 2 | 2 | o | | | | o | | | |
| pde4dip protein | 2 | o | | | | | | | |
| pdgfa associated protein 1 | 2 | o | | | | | | | |
| pdz and lim domain 4 | 2 | o | | o | | | | | |
| pdz and lim domain 5 isoform 1 | 2 | o | | | | | | | |
| pdz and lim domain 7 | 2 | o | | | | | | | |
| pdz and lim domain protein 3 | 2 | o | | | | | | | |
| pdz binding kinase | 2 | o | | | | | | | |
| pecanex-like 2 | 2 | | | | | | o | | |
| pentatricopeptide repeat-containing protein 2 | 2 | | | | o | | | | |
| peptidase (mitochondrial processing) alpha | 2 | o | o | | | | | | |
| peptidase (mitochondrial processing) beta | 2 | o | | | | | | | |
| peptidase astacin | 2 | | | | o | | | | |
| peptidase inhibitor 16 | 2 | o | | | | | o | | |
| peptidase inhibitor r3hdml-like | 2 | | | | o | | | | |
| peptidase m23 | 2 | o | | | | | | | |
| peptide-n4-n-acetyl-beta-d-glucosaminylasparagine amidase f precursor | 2 | | | | o | | | | |

| | | | | | | | | | |
|---|---|---|---|---|---|---|---|---|---|
| peptidoglycan recognition protein ii | 2 | | o | | o | | | | |
| peptidyl-prolyl cis-trans isomerase e | 2 | o | | | | | | | o |
| peptidyl-prolyl cis-trans isomerase nima-interacting 4 | 2 | o | | | | o | | | |
| peptidylprolyl isomerase -like 2 | 2 | | o | | | o | | | |
| peptidylprolyl isomerase -like 5 | 2 | o | | | | | | | o |
| peptidyl-trna hydrolase mitochondrial precursor | 2 | o | | o | | | | | |
| periphilin 1 | 2 | o | | | | | | | |
| Periphilin-1 [Salmo salar] | 2 | o | | o | | | | | |
| peroxisomal biogenesis factor 11 gamma | 2 | | o | | | | | | |
| peroxisomal biogenesis factor 19 | 2 | o | o | | | | | | |
| perq amino acid-rich with gyf domain-containing protein 2 | 2 | | o | o | | | | | |
| pescadillo homolog containing brct domain | 2 | o | | | | o | | | |
| pest proteolytic signal containing nuclear protein | 2 | o | | o | | | | | |
| ph domain and leucine rich repeat protein phosphatase | 2 | o | | | | | | o | |
| phd finger protein 3 | 2 | o | | | | | | | |
| phd finger protein 5a | 2 | o | | | | o | | | |
| phd finger protein 6 | 2 | o | | | | | | | |
| phenazine biosynthesis-like domain-containing protein 2 | 2 | | o | o | | | | | |
| phldb3 protein | 2 | | o | o | | | | | |
| phosphatase and actin regulator 1 | 2 | | | | | | o | | o |
| phosphatase and actin regulator 3 | 2 | | | | | | o | o | |
| phosphate cytidylyltransferase ethanolamine | 2 | o | o | | | | | | |
| phosphatidylcholine transfer protein | 2 | o | | | | o | | | |
| phosphatidylinositol 3-kinase regulatory subunit gamma | 2 | o | | | | | | | |
| phosphatidylinositol 4-kinase type ii | 2 | | | o | | | | | o |
| phosphatidylinositol n-acetylglucosaminyltransferase subunit p | 2 | | | | o | | | | |
| phosphatidylinositol-5-phosphate 4- type beta | 2 | o | | | | | | o | |
| phosphodiesterase 10a | 2 | | o | | | | | | |
| phosphofurin acidic cluster sorting protein 2 | 2 | | | | | | o | | |
| phosphoglucomutase 3 | 2 | | o | o | | | | | |
| phosphoglucomutase 5 | 2 | o | | | | | | | |
| phosphogluconate dehydrogenase | 2 | o | o | | | | | | |
| phosphoglucose isomerase-2 | 2 | o | | | | | | | o |
| phosphoglycerate mutase 2 | 2 | o | | | | | | | |
| phosphoglycerate mutase family member 5 | 2 | o | o | | | | | | |
| phosphoinositide-3- class 3 | 2 | o | o | | | | | | |
| phospholipase a2-activating protein | 2 | | o | | | | | | |
| phospholipase beta 1 | 2 | o | | | | | | o | |
| phospholipase c-like 2 | 2 | o | | | | | | o | |
| phospholipase group ib | 2 | o | o | | | | | | |
| phospholipase group iii | 2 | | | o | | | o | | |
| phosphomannomutase 2 | 2 | o | o | | | | | | |
| phosphoprotein enriched in astrocytes 15 | 2 | | | | | | o | o | |
| phytoene synthase-like precursor | 2 | o | o | | | | | | |
| pif harbinger-like protein | 2 | o | | o | | | | | |
| piggybac transposase uribo1 | 2 | o | | | | o | | | |
| pim-3 oncogene | 2 | | | o | | | o | | |
| pirin | 2 | | | | o | | | | |
| piwi-like protein 1 | 2 | | | | o | | | | |
| plakophilin-4 isoform 2 | 2 | | | | | | | | o |
| plasma kallikrein | 2 | | | o | o | | | | |

| | | | | | | | | | |
|---|---|---|---|---|---|---|---|---|---|
| plasmodium falciparum trophozoite antigen r45-like protein | 2 | | | o | | | | o | |
| platelet derived growth factor receptor alpha | 2 | o | | | | | | | |
| platelet glycoprotein 4 | 2 | o | o | | | | | | |
| pleckstrin | 2 | o | | | | o | | | |
| pleckstrin homology domain containing family a member 8 | 2 | o | | | | | | | o |
| pleckstrin homology domain family a member 6 | 2 | | o | o | | | | | |
| pleckstrin homology domain family g (with ef domain) member 1 | 2 | o | | | | | | | |
| pleckstrin homology domain-containing family a member 8 | 2 | o | | | | | | o | |
| plectin intermediate filament binding protein isoform cra_b | 2 | | | | | | o | o | |
| plectin isoform 1 | 2 | o | | | | | | | |
| pleiotropic regulator 1 | 2 | o | | o | | | | | |
| pol partial | 2 | | | o | | | | | |
| poliovirus receptor-related protein 1-like | 2 | | o | | | | o | | |
| polo-like kinase 1 | 2 | o | o | | | | | | |
| poly (adp-ribose) polymerase member 1 | 2 | | | | o | o | | | |
| poly (adp-ribose) polymerase member 14 | 2 | o | | o | | | | | |
| poly (adp-ribose) polymerase member 15 | 2 | o | | | | | | | |
| poly (adp-ribose) polymerase member 4 | 2 | o | | | | | | | |
| poly binding cytoplasmic 4 (inducible form) | 2 | o | | | o | | | | |
| poly binding protein 2 | 2 | o | | | | | o | | |
| poly binding protein 3 | 2 | | o | | | | o | | |
| poly -binding protein 3-like | 2 | | | | | | o | | |
| poly polymerase gamma | 2 | o | | o | | | | | |
| poly -specific endoribonuclease | 2 | o | o | | | | | | |
| polyadenylate-binding protein 2 | 2 | o | | | | | o | | |
| polyadenylate-binding protein-interacting protein 2b | 2 | o | o | | | | | | |
| polyamine-modulated factor 1 | 2 | | | | o | | | | |
| polybromo 1 | 2 | | o | | | | o | | |
| polybromo 1 isoform 2 | 2 | o | | | | o | | | |
| polycomb group ring finger 6 | 2 | o | | | | | | | |
| polymerase ii (dna directed) polypeptide 33kda | 2 | o | | o | | | | | |
| polymerase iii (dna directed) polypeptide c | 2 | | o | o | | | | | |
| polypeptide 1 | 2 | o | | | | o | | | |
| polypeptide n-acetylgalactosaminyltransferase 6 | 2 | | | | o | | | | |
| polyprotein | 2 | o | | o | | | | | |
| polypyrimidine tract binding protein 1 | 2 | o | | | | o | | | |
| polyubiquitin | 2 | | | | | | | o | o |
| polyubiquitin with 3 ub domains | 2 | o | | | o | | | | |
| potassium channel subfamily t member 1-like | 2 | | | | | | o | o | |
| potassium channel subunit | 2 | o | | | | | o | | |
| potassium channel tetramerisation domain containing 14 | 2 | o | | o | | | | | |
| potassium channel tetramerisation domain containing 5 | 2 | o | | | | | | | |
| potassium channel tetramerisation domain containing 9 | 2 | o | | | | | | | |
| potassium inwardly-rectifying subfamily member 12 | 2 | o | | | | | | | |
| potassium inwardly-rectifying subfamily member 3 | 2 | | | | | | o | o | |
| potassium subfamily member 1 | 2 | | | | | | o | o | |

| | | | | | | | | | |
|---|---|---|---|---|---|---|---|---|---|
| potassium subfamily member 2 | 2 | | | | | | o | | |
| pou domain protein class v transcription factor 1 | 2 | | | | o | | | | |
| ppia protein | 2 | | | | o | | | o | |
| ppm2c protein | 2 | o | | | | | | | |
| PREDICTED: klotho-like [Oreochromis niloticus] | 2 | | | | o | | | | |
| PREDICTED: predicted protein-like [Danio rerio] | 2 | o | | | | | | | |
| PREDICTED: similar to transposase [Strongylocentrotus purpuratus] | 2 | o | | | | | o | | |
| PREDICTED: wu:fl04e06 [Danio rerio] | 2 | o | | | | o | | | |
| pregnancy specific beta-1-glycoprotein 1-like | 2 | o | | | | | | o | |
| pre-mrna-processing factor 39 | 2 | | o | | | | | | o |
| pre-mrna-processing factor 40 homolog a | 2 | o | | o | | | | | |
| pre-mrna-splicing factor 38a | 2 | | o | | o | | | | |
| pre-mrna-splicing factor atp-dependent rna helicase prp16 | 2 | | o | | | | o | | |
| pre-mrna-splicing factor cwc22 homolog | 2 | o | | | | | o | | |
| pre-mrna-splicing factor slu7 | 2 | | o | o | | | | | |
| pre-mrna-splicing factor syf1 | 2 | o | | | | | | | o |
| presenilin rhomboid-like | 2 | o | o | | | | | | |
| prion protein 2 | 2 | o | | | | | | | |
| probable atp-dependent rna helicase ddx27 | 2 | | | | | | o | | o |
| probable atp-dependent rna helicase ddx5 | 2 | o | o | | | | | | |
| probable atp-dependent rna helicase ddx56 | 2 | | | | o | | | | o |
| probable atp-dependent rna helicase dhx58-partial | 2 | o | | | | | | | |
| probable bifunctional methylenetetrahydrofolate dehydrogenase cyclohydrolase 2 | 2 | | | | o | | | | |
| probable c- | 2 | o | | | | | | | |
| probable carboxypeptidase x1-like | 2 | | | | | | | | o |
| probable e3 ubiquitin-protein ligase herc1 | 2 | | | o | | | | | o |
| probable exonuclease mut-7 homolog | 2 | | | | | o | | | o |
| probable hydrolase pnkd-like | 2 | | | | | | | | o |
| probable proline racemase | 2 | o | | o | | | | | |
| probable rna-binding protein 19 | 2 | o | | | | | | | |
| probable rrna-processing protein ebp2 | 2 | o | | o | | | | | |
| probable saccharopine dehydrogenase | 2 | | o | | | | | | |
| probable serine carboxypeptidase cpvl precursor | 2 | | | o | | | | | o |
| probable u3 small nucleolar rna-associated protein 11-like | 2 | | | | o | | | | |
| probable ubiquitin carboxyl-terminal hydrolase faf-x | 2 | o | | | | | | | |
| probable udp-sugar transporter protein slc35a4 | 2 | o | o | | | | | | |
| procollagen c-endopeptidase enhancer 2 | 2 | o | | | | | | o | |
| prodh protein | 2 | o | | | | o | | | |
| prodynorphin precursor | 2 | | | | | | o | | o |
| programmed cell death protein 7 | 2 | | o | | | | o | | |
| progressive ankylosis protein | 2 | o | | | | | | | o |
| progressive ankylosis protein homolog | 2 | | | | | | | o | o |
| proline dehydrogenase 1 | 2 | o | o | | | | | | |
| proline mitochondrial-like | 2 | | o | | | | | | |
| proline-rich protein 12 | 2 | | | | | | o | | |
| proline-rich transmembrane protein 4 | 2 | o | | | | | o | | |
| prolyl 4-hydroxylase subunit alpha-1 precursor | 2 | o | | | | | | | |

| | | | | | | | | | |
|---|---|---|---|---|---|---|---|---|---|
| prolylcarboxypeptidase (angiotensinase c) | 2 | o | | o | | | | | |
| prominin- splice variant | 2 | | o | | | | o | | |
| pro-neuregulin- membrane-bound isoform | 2 | | | | | | o | | |
| prostacyclin synthase | 2 | o | | | | | | | |
| proteasome ( macropain) 26s non- 5 | 2 | o | o | | | | | | |
| proteasome ( macropain) 26s non- 9 | 2 | o | | | | o | | | |
| proteasome activator complex subunit 4 | 2 | o | | | | | | | |
| proteasome subunit beta type-5 | 2 | | | o | | | | | o |
| proteasome-associated protein ecm29 homolog | 2 | o | o | | | | | | |
| protein amp- alpha 2 catalytic subunit | 2 | o | | | | | o | | |
| protein bat2-like 2 | 2 | o | | | | | | | |
| protein camp- beta | 2 | o | | | | | | | |
| protein casc4 | 2 | o | | | | | o | | |
| protein containing duf1446 | 2 | o | o | | | | | | |
| protein deltex-1 | 2 | | | | | | o | o | |
| protein deltex-1-like | 2 | | | | | | o | | |
| protein disulfide isomerase associated 4 | 2 | | | o | | o | | | |
| protein disulfide isomerase family member 6 | 2 | | o | o | | | | | |
| protein disulfide-isomerase | 2 | o | | o | | | | | |
| protein dopey-2 | 2 | | | | | o | | | o |
| protein dpy-30 homolog | 2 | | | | o | o | | | |
| protein fam164a-like | 2 | | | | | | | | o |
| protein fam168b-like | 2 | | | | | | o | | o |
| protein fam46a-like isoform 2 | 2 | | | o | | | | | |
| protein fam49b-like | 2 | | | | | | | | o |
| protein fem-1 homolog c | 2 | o | | | | | | | |
| protein furry homolog-like | 2 | | | | | | o | | |
| protein homolog mitochondrial precursor | 2 | | o | | | | | o | |
| protein kinase c delta type | 2 | o | o | | | | | | |
| protein kinase c-binding protein nell2 isoform 2 | 2 | | | | | | o | | |
| protein kinase domain cytoplasmic homolog | 2 | o | | | | | | | |
| protein kinase raf 1 | 2 | o | | o | | | | | |
| protein kinase-like protein | 2 | | | | o | | | | |
| protein lsm14 homolog b-like | 2 | | | | o | | | | |
| protein lyric-like | 2 | | | | o | | | | |
| protein mdm4 | 2 | | o | | | | o | | |
| protein mef2bnb-like | 2 | | | | o | | | | |
| protein memo1 | 2 | o | o | | | | | | |
| protein mida mitochondrial-like | 2 | | | | o | | | | |
| protein mon2 homolog | 2 | | | | | | | | o |
| protein muted homolog | 2 | o | | o | | | | | |
| protein njmu-r1-like | 2 | | | | o | | o | | |
| protein noxp20-like | 2 | | | o | | | | | |
| protein n-terminal glutamine amidohydrolase | 2 | o | | | | o | | | |
| protein os-9 | 2 | o | | | | o | | | |
| protein pelota homolog | 2 | | o | | | | | | |
| protein phosphatase 1 (formerly 2c)-like | 2 | o | | | | | | | |
| protein phosphatase 1 regulatory subunit 12a | 2 | o | o | | | | | | |
| protein phosphatase 1b | 2 | o | | | | | o | | |
| protein phosphatase 1g (formerly 2c) magnesium- gamma isoform | 2 | o | o | | | | | | |
| protein phosphatase 2 (formerly 2a) catalytic alpha isoform | 2 | o | | o | | | | | |
| protein phosphatase catalytic beta isoform | 2 | | | | | | | o | |
| protein phosphatase regulatory subunit 14b | 2 | o | | | | | | | |
| protein phosphatase regulatory subunit 1-like | 2 | o | | | | | | | |
| protein phosphatase regulatory subunit b alpha isoform | 2 | o | | | | | | | |

| | | | | | | | | | |
|---|---|---|---|---|---|---|---|---|---|
| protein phosphatase type 2c alpha 2 | 2 | | | o | | | o | | |
| protein piccolo isoform 2 | 2 | | | | | | o | o | |
| protein piccolo-like | 2 | | | | | | | o | |
| protein polybromo-1 | 2 | | | | | | | | o |
| protein quaking-b | 2 | o | | | | | | o | |
| protein red | 2 | | o | | | | | | o |
| protein red-like | 2 | | o | | | | o | | |
| protein rrp5 homolog | 2 | | o | | o | | | | |
| protein sco1 mitochondrial-like | 2 | | | | o | | | o | |
| protein sel-1 homolog 1 | 2 | | | | | | | | o |
| protein set-like | 2 | | | | | | o | o | |
| protein sidekick-2 | 2 | o | | | | | | | o |
| protein tmed8 | 2 | o | | | | | | | |
| protein transport protein sec61 subunit alpha isoform 2 | 2 | o | | | | | | | |
| protein tssc1 | 2 | | | | | | o | | o |
| protein tyrosine phosphatase type member 1 | 2 | | o | | | | | | o |
| protein tyrosine phosphatase type member 3 | 2 | o | | | | | | o | |
| protein tyrosine receptor c | 2 | o | | | | | o | | |
| protein tyrosine receptor f | 2 | | | | | | o | | |
| protein tyrosine receptor f polypeptide interacting protein alpha 3 | 2 | | | | | | o | | |
| protein tyrosine receptor isoform cra_b | 2 | o | | | | | | o | |
| protein tyrosine receptor n | 2 | | | | | o | | o | |
| protein unc-119 homolog b | 2 | | | | | | o | o | |
| protein unc-13 homolog a | 2 | | | | | o | o | | |
| protein vac14 homolog | 2 | | o | | | | | | |
| protein-glutamine gamma-glutamyltransferase k | 2 | o | | | | | | | |
| protein-l-isoaspartate o-methyltransferase domain-containing protein 1 | 2 | o | | o | | | | | |
| protein-l-isoaspartate(d-aspartate) o-methyltransferase-like | 2 | o | | | | | | | |
| protein-tyrosine sulfotransferase 1-like | 2 | | | | o | | | | |
| protocadherin 11 x-linked | 2 | | | | | | o | o | |
| protocadherin 2 alpha b 10 | 2 | o | | | | o | | | |
| protocadherin 2a14 | 2 | | | | | o | o | | |
| protocadherin 2a5-like | 2 | o | | | | | | o | |
| protocadherin 2g25 | 2 | o | | | | | o | | |
| protocadherin 2g8 | 2 | o | | | | | | | |
| proto-oncogene protein c-fos | 2 | o | | | | | o | | |
| proto-oncogene serine threonine-protein kinase pim-1 | 2 | o | | | | | | | |
| pt repeat family protein | 2 | | | | o | | | | |
| ptb domain-containing engulfment adapter protein 1-like | 2 | | | | | | | | o |
| pterin-4-alpha-carbinolamine dehydratase 2 | 2 | o | | | | o | | | |
| ptk2b protein tyrosine kinase 2 beta | 2 | o | | | | o | | | |
| ptprd protein | 2 | o | | | | | | o | |
| pumilio 2 | 2 | | | | | o | o | | |
| purine rich element binding protein b | 2 | | o | o | | | | | |
| purinergic receptor g-protein 5 | 2 | o | o | | | | | | |
| purine-rich element binding protein a | 2 | o | | | | | | | |
| purkinje cell protein 4 variant 4 | 2 | | | o | | o | | | |
| pyridoxal phosphate phosphatase phospho2 | 2 | o | | | | o | | | |
| pyroglutamyl-peptidase 1 | 2 | o | o | | | | | | |
| pyruvate dehydrogenase | 2 | | o | | | | | | o |
| pyruvate dehydrogenase isozyme 2 | 2 | | o | | | | | | |
| pyruvate dehydrogenase kinase 2 | 2 | o | | | | | o | | |
| quinone oxidoreductase-like protein 1 | 2 | o | | o | | | | | |

| | | | | | | | | | |
|---|---|---|---|---|---|---|---|---|---|
| rab proteins geranylgeranyltransferase component a 2 | 2 | | | | | | | | o |
| rab3a interacting protein | 2 | o | | | | | | o | |
| rab-3a-interacting protein | 2 | | | | | | | | o |
| rab6 interacting protein 1 | 2 | o | | | | | o | | |
| rab-like protein 5 | 2 | o | | | o | | | | |
| rabphilin 3a homolog | 2 | | | | | | o | o | |
| rac-beta serine threonine-protein kinase | 2 | | o | | | | | | |
| rad51-associated protein 1 | 2 | o | | | | | | | |
| ral gtpase-activating protein subunit beta-like | 2 | o | | | | | | | |
| ran | 2 | | | | o | | | | |
| ran_gtp binding protein 5 | 2 | o | | | | | | | |
| ranbp-type and c3hc4-type zinc finger-containing protein 1-like | 2 | | | | o | | | | |
| rap1 gtpase-activating protein 1 | 2 | | | | | o | | | o |
| rapunzel 4 | 2 | o | | | | | | | o |
| rapunzel 5 | 2 | o | | | | | | | |
| ras and rab interactor 2 | 2 | o | | | | | | | |
| ras association domain family 1 | 2 | o | | o | | | | | |
| ras association domain-containing protein 2 | 2 | | | o | | | | o | |
| ras gtpase-activating protein | 2 | | | | | | o | o | |
| ras homolog gene member b | 2 | o | | o | | | | | |
| ras homolog gene member t1 | 2 | o | o | | | | | | |
| ras homolog gene member t1a | 2 | o | | | | | o | | |
| ras p21 protein activator 1 | 2 | o | | | | | | | |
| ras protein-specific guanine nucleotide-releasing factor 1 | 2 | | | | | | o | o | |
| ras protein-specific guanine nucleotide-releasing factor 2 | 2 | | | | | | o | o | |
| ras-interacting protein 1 | 2 | o | | | | | | | |
| ras-related c3 botulinum toxin substrate 2 precursor | 2 | o | | o | | | | | |
| ras-related c3 botulinum toxin substrate 3 (rho small gtp binding protein rac3) | 2 | | o | | | | o | | |
| ras-related gtp binding d | 2 | o | | | | | | | |
| ras-related protein rab-33b-like | 2 | | | | o | | | | o |
| ras-related protein rab-38-like | 2 | | | | | | | | o |
| ras-related protein rab-6a | 2 | | o | o | | | | | |
| rbm25 protein | 2 | o | o | | | | | | |
| rearranged l-myc fusion | 2 | o | | | | | | | |
| receptor accessory protein 1 | 2 | o | | | | | | | |
| receptor accessory protein 3 | 2 | o | o | | | | | | |
| receptor-binding cancer antigen expressed on o cells | 2 | | | o | | | | o | |
| receptor-interacting serine threonine-protein kinase 1 | 2 | o | o | | | | | | |
| receptor-type tyrosine-protein phosphatase o | 2 | | | | | | o | | |
| receptor-type tyrosine-protein phosphatase-like n-like | 2 | | | | | | | | o |
| recombination activating gene 1 activating protein 1 | 2 | o | | o | | | | | |
| regucalcin | 2 | o | | | | | | | |
| regulating synaptic membrane exocytosis 1 | 2 | | | | | | o | o | |
| regulation of nuclear pre-mrna domain-containing protein 1a | 2 | o | | | | | | | |
| regulator of chromosome condensation and btb domain containing protein 2 | 2 | o | | | | | | o | |
| regulator of g-protein signaling 12-like | 2 | | | | | | | | o |
| regulator of g-protein signaling 14-like | 2 | | | | o | | | | |
| regulator of g-protein signaling 5 | 2 | o | o | | | | | | |

| | | | | | | | | | |
|---|---|---|---|---|---|---|---|---|---|
| regulator of g-protein signaling 7 | 2 | | | | | | | | o |
| regulator of g-protein signaling 9-binding | 2 | | | | | | | | o |
| regulator of g-protein signalling 3 | 2 | o | | | | | o | | |
| regulatory factor 7 | 2 | o | | | | | | | |
| -related lipid transfer domain containing 13 | 2 | | o | | | | | | |
| relaxin insulin-like family peptide receptor 2 | 2 | o | | | | | | | |
| remodeling and spacing factor 1 | 2 | o | | | | | | | |
| replication factor c subunit 3 | 2 | o | | | o | | | | |
| replication factor c subunit 4 | 2 | | | | o | | | | |
| replication protein 32kda | 2 | o | o | | | | | | |
| reticulocalbin 2 | 2 | o | | | | | | | |
| reticulon 4 | 2 | o | o | | | | | | |
| reticulon 4 receptor | 2 | o | | | | | | | o |
| reticulon 4-l2 | 2 | o | | | | | | o | |
| reticulon 6-a1(2023 2173) | 2 | | | | | | o | o | |
| retinal rod rhodopsin-sensitive cgmp 3 -cyclic phosphodiesterase subunit delta | 2 | o | | | | | o | | |
| retinoid x receptor gamma | 2 | o | | | | | | | |
| retinol dehydrogenase 10 | 2 | | | o | | | | | o |
| retinol dehydrogenase 8 | 2 | | o | o | | | | | |
| retinol saturase (all-trans-retinol -reductase) | 2 | o | o | | | | | | |
| retinol-binding protein 2 | 2 | | o | o | | | | | |
| retroelement pol polyprotein | 2 | o | | | | | | | |
| retrograde golgi transport protein rgp1 homolog | 2 | | o | | o | | | | |
| reverse ribonuclease integrase | 2 | | | | | o | o | | |
| reverse transcriptase and rnase h-like protein | 2 | o | | | | | | | |
| reverse transcriptase-like partial | 2 | o | | | | o | | | |
| rgd1564379 protein | 2 | | | o | | | | o | |
| rgm domain member a | 2 | o | | | | | | | |
| rho family gtpase 2 | 2 | o | | | | o | | | |
| rho family gtpase 3 | 2 | o | | | | | o | | |
| rho gtpase activating protein 18 | 2 | o | o | | | | | | |
| rho gtpase activating protein 4 | 2 | o | | | | o | | | |
| rho gtpase-activating protein 21 | 2 | | | | | | o | | |
| rho guanine nucleotide exchange factor 17 | 2 | | o | | | | | | o |
| rhotekin | 2 | | | | | o | o | | |
| ribonuclease inhibitor-like | 2 | o | | | | | | | |
| ribonuclease p mrp 30kda subunit | 2 | | | o | | | o | | |
| ribonucleoside-diphosphate reductase large subunit-like | 2 | | | | o | | | | |
| ribonucleotide reductase m2 b (tp53 inducible) | 2 | o | | | | | | o | |
| ribonucleotide reductase m2 polypeptide | 2 | o | | | | | | | |
| ribose-phosphate pyrophosphokinase 2 | 2 | | o | | | o | | | |
| ribosomal l1 domain-containing protein 1-like | 2 | | | | o | | | | |
| ribosomal protein l17 | 2 | | o | | | | o | | |
| ribosomal protein s6 kinase beta-1 | 2 | o | | | o | | | | |
| ribosomal protein s6 polypeptide 2 | 2 | o | | o | | | | | |
| ribosomal rna methyltransferase nop2 | 2 | | o | | | | | | o |
| ribosome biogenesis protein bms1 homolog | 2 | | | | o | | | | |
| ribosome-binding protein 1 | 2 | | o | | o | | | | |
| riken cdna 4832428d23 | 2 | o | | | | | | | |
| riken cdna 4931428f04 gene | 2 | | | | | o | | o | |
| rilp-like protein 1-like | 2 | | o | | o | | | | |
| ring finger protein 122-like | 2 | | | | | | | | o |
| ring finger protein 126 | 2 | o | | | | | | | o |
| ring finger protein 13 | 2 | | o | | | | | | o |
| ring finger protein 146 | 2 | o | | | | | o | | |
| ring finger protein 2 | 2 | o | o | | | | | | |

| | | | | | | | | | |
|---|---|---|---|---|---|---|---|---|---|
| ring finger protein 38 | 2 | o | | | | | | o | |
| ring finger protein 40 | 2 | o | | | | | o | | |
| rna (guanine-9-) methyltransferase domain containing 1 | 2 | o | o | | | | | | |
| rna binding homolog 1 | 2 | | o | | | | | o | |
| rna binding motif protein 12b | 2 | o | | | | | | | |
| rna binding motif protein 19 | 2 | o | | | | | | | |
| rna binding motif protein 22 | 2 | o | | o | | | | | |
| rna binding motif protein 26 | 2 | o | o | | | | | | |
| rna binding motif protein 28 | 2 | | o | | | | | | |
| rna binding motif x-linked 2 | 2 | o | | | | o | | | |
| rna exonuclease 1 homolog | 2 | o | | o | | | | | |
| rna exonuclease 4 | 2 | | | | o | | | | |
| rna polymerase ii largest subunit | 2 | o | | | | | o | | |
| rna pseudouridylate synthase domain containing 2 | 2 | | | | o | | | | o |
| rna terminal phosphate cyclase domain 1 | 2 | o | o | | | | | | |
| rna-binding protein 26 | 2 | o | | | o | | | | |
| rna-binding protein 4b | 2 | o | o | | | | | | |
| rna-binding protein 6 | 2 | | | | | | o | | |
| rna-binding protein 7 | 2 | | | | | | | o | o |
| rna-binding single-stranded-interacting protein 3 | 2 | | | | | o | | | o |
| rrna promoter binding protein | 2 | | | | | o | o | | |
| rrna-processing protein utp23 homolog | 2 | o | | | o | | | | |
| rrp12-like protein | 2 | | | | o | | | | |
| run and sh3 domain containing 1 | 2 | o | | | | | o | | |
| run domain-containing protein 3b | 2 | o | | | | | | | o |
| ryanodine receptor 2 | 2 | o | | | | | o | | |
| S100-A6 [Anoplopoma fimbria] | 2 | o | | | | | | o | |
| s-adenosylmethionine synthase isoform type-1 | 2 | | o | | o | | | | |
| safb- transcription modulator | 2 | | | | o | | | o | |
| safb-like transcription modulator | 2 | | o | | o | | | | |
| sam and sh3 domain containing 1 | 2 | o | | | | | | | |
| sam domain and hd 1 | 2 | o | | | | | | | |
| sam domain and hd domain-containing protein 1 | 2 | o | | | | | | | |
| sarcoplasmic endoplasmic reticulum calcium atpase 2-like | 2 | | | o | | | o | | |
| sarcospan | 2 | o | | o | | | | | |
| sbf1 protein | 2 | | | | | o | o | | |
| scavenger receptor class f member 2-like | 2 | o | | | | o | | | |
| scavenger receptor cysteine-rich protein type 12 precursor | 2 | | o | | | | | | |
| scg10 protein | 2 | | | | | o | o | | |
| sclerostin domain-containing protein 1-like | 2 | | | | | | | | o |
| scm-like with four mbt domains protein 2 | 2 | | | | o | | | | |
| sco cytochrome oxidase deficient homolog 1 | 2 | o | | | | | | | |
| sda1 domain containing 1 | 2 | o | | | | | | | |
| sec1 family domain containing 2 | 2 | | o | o | | | | | |
| sec14-like protein 2 | 2 | | o | | | | | | |
| sec61 alpha 1 subunit ( cerevisiae) | 2 | o | | | | | | | |
| sec61-alpha | 2 | | | o | | | | | |
| secreted phosphoprotein 24 precursor | 2 | | o | o | | | | | |
| secretogranin v | 2 | | | | | o | | o | |
| secretory carrier membrane protein 2 | 2 | | o | | | o | | | |
| secretory carrier-associated membrane protein 5 | 2 | | | | | | o | o | |
| securin | 2 | | | | o | | | | |

| | | | | | | | | | |
|---|---|---|---|---|---|---|---|---|---|
| sel1 repeat-containing protein 1 | 2 | | | | o | | | | |
| selenophosphate synthetase 2 | 2 | | o | | | | | | o |
| selenoprotein j | 2 | | | | o | | | | o |
| selenoprotein l | 2 | | | o | | | | | |
| selenoprotein o | 2 | | o | | | | | | |
| selenoprotein p | 2 | | | | o | | | | |
| selenoprotein t precursor | 2 | | | | | | | | o |
| sema immunoglobulin domain transmembrane domain and short cytoplasmic 4g | 2 | | | | | | o | | |
| semaphorin 3fa | 2 | o | | | | | | o | |
| seminal plasma glycoprotein 120 | 2 | o | | | | | o | | |
| sept8a protein | 2 | o | | | | o | | | |
| septin 3 | 2 | | o | | | | o | | |
| septin 5 | 2 | | | | | | | | o |
| serine arginine repetitive matrix protein 1 | 2 | | o | | | | o | | |
| serine arginine-rich splicing factor 11-like | 2 | | | | | | o | | o |
| serine carboxypeptidase 1 | 2 | o | | | o | | | | |
| serine long chain base subunit 2 | 2 | o | o | | | | | | |
| serine peptidase 1 | 2 | o | | | | | | | |
| serine peptidase kazal type 1 | 2 | | o | | | | | o | |
| serine threonine kinase 11 | 2 | o | | | | | o | | |
| serine threonine kinase 25 (ste20 yeast) | 2 | o | o | | | | | | |
| serine threonine kinase 38 | 2 | o | | | | | | | |
| serine threonine kinase 40 | 2 | o | | o | | | | | |
| serine threonine protein kinase raf1 | 2 | o | o | | | | | | |
| serine threonine-protein kinase 12 | 2 | | o | | o | | | | |
| serine threonine-protein kinase nek6 | 2 | o | | | | | | | o |
| serine threonine-protein kinase plk2 | 2 | o | | | | | o | | |
| serine threonine-protein kinase rio3 | 2 | o | | | | o | | | |
| serine threonine-protein kinase sbk2-like | 2 | o | | | | | | | |
| serine threonine-protein kinase wnk1 isoform 3 | 2 | | | | | | o | | |
| serine threonine-protein phosphatase 2a 55 kda regulatory subunit b delta isoform-like | 2 | | | | o | | | | |
| serine threonine-protein phosphatase 2a 56 kda regulatory subunit epsilon isoform | 2 | o | | | | | | | |
| serine threonine-protein phosphatase 2a activator-like | 2 | | | | o | | | | o |
| serine threonine-protein phosphatase 2a catalytic subunit alpha isoform-like partial | 2 | | | | o | | | | |
| serine threonine-protein phosphatase mitochondrial-like | 2 | | | | o | | | | |
| serine threonine-protein phosphatase pp1-beta | 2 | | o | o | | | | | |
| serine--pyruvate mitochondrial precursor | 2 | | o | | | | | | |
| serologically defined colon cancer antigen 1 | 2 | o | | | | o | | | |
| serum amyloid a | 2 | | | | o | | | | |
| serum deprivation response | 2 | o | | o | | | | | |
| serum response factor | 2 | | o | | | | | | |
| seryl-trna synthetase 2 | 2 | | | | o | | | | |
| sestrin 2 | 2 | | | | o | | | | |
| set and mynd domain containing 2 | 2 | o | | | | | | | |
| set bifurcated 1 | 2 | o | o | | | | | | |
| set binding factor 2 | 2 | o | | | | | o | | |
| set translocation | 2 | o | | o | | | | | |
| set translocation (myeloid leukemia-associated) | 2 | o | | | | | | o | |
| setd8b protein | 2 | | o | | o | | | | |
| sfrs12-interacting protein 1 | 2 | o | | o | | | | | |
| sft2 domain containing 1 | 2 | o | | | | | | o | |

| | | | | | | | | | |
|---|---|---|---|---|---|---|---|---|---|
| sh2 domain containing 5 | 2 | | o | | | | | | |
| sh3 and multiple ankyrin repeat domains protein 3 | 2 | o | | | | | o | | |
| sh3 and px domain-containing protein 2a | 2 | o | | | | | | | |
| sh3 and px domains 2a | 2 | o | | | | | | | |
| sh3 and px domains 2b | 2 | o | | | | | | | |
| sh3 domain and tetratricopeptide repeats-containing protein 2 | 2 | | o | | | | | | o |
| sh3 domain-binding glutamic acid-rich protein | 2 | o | | | | | | | |
| sh3-domain grb2-like endophilin b1 | 2 | o | o | | | | | | |
| short branched chain specific acyl- mitochondrial | 2 | | | | | | o | | o |
| short-chain dehydrogenase reductase sdr | 2 | | | | | | | | o |
| short-chain specific acyl- mitochondrial | 2 | o | o | | | | | | |
| sialidase-1 precursor | 2 | o | | o | | | | | |
| sideroflexin 1 | 2 | | o | | | | o | | |
| signal recognition particle 14 kda protein | 2 | o | | o | | | | | |
| signal recognition particle 68 | 2 | o | | | | | | | |
| signal transducer and activator of transcription 5 | 2 | o | | | | | | | |
| signal transducer and activator of transcription 5b | 2 | o | | o | | | | | |
| signal transducing adaptor molecule (sh3 domain and itam motif) 1 | 2 | o | | | | o | | | |
| signal-induced proliferation-associated protein 1 | 2 | | | | | | | | o |
| silva protein | 2 | o | | | | o | | | |
| sjchgc09003 protein | 2 | | | o | | o | | | |
| slit and ntrk-like member 1 | 2 | | | | | | o | | |
| slit and ntrk-like member 6 | 2 | | | | | | | | o |
| slit and ntrk-like protein 3-like | 2 | | | | | | o | | |
| slit-robo rho gtpase activating protein 2 | 2 | o | | | | | o | | |
| small edrk-rich factor 2 | 2 | | o | | | | o | | |
| small glutamine-rich tetratricopeptide repeat -beta | 2 | o | | | | o | | | |
| small gtpase ras-dva-3 | 2 | | | | | | o | | |
| small subunit processome component 20 homolog | 2 | o | | | | | | | o |
| smc4 protein | 2 | o | | | | | o | | |
| smoothened homolog | 2 | o | | | | | | | |
| snare protein ykt6 | 2 | o | | o | | | | | |
| snrna-activating protein complex subunit 1-like | 2 | | | | o | | | | |
| snrna-activating protein complex subunit 5-like | 2 | | | | | | | | o |
| sodium- and chloride-dependent gaba transporter 2-like | 2 | | | | o | | | | o |
| sodium bicarbonate cotransporter | 2 | | | | | | | o | |
| sodium channel voltage-gated type ii alpha 1 | 2 | | | | | | o | o | |
| sodium potassium-transporting atpase alpha-1 subunit | 2 | o | o | | | | | | |
| sodium potassium-transporting atpase subunit beta-2 | 2 | | | | | | o | o | |
| sodium potassium-transporting atpase subunit beta-3-like | 2 | | | | | | | | o |
| sodium-coupled neutral amino acid transporter 3 | 2 | | o | | | | | | |
| sodium-dependent phosphate transporter 2 | 2 | | | | | | o | | |
| solute carrier family 12 member 2 isoform 2 | 2 | | | | | | o | | |
| solute carrier family 13 member 4 | 2 | | | | | | o | | o |

| | | | | | | | | | |
|---|---|---|---|---|---|---|---|---|---|
| solute carrier family 16 (monocarboxylic acid transporters) member 12 | 2 | o | | o | | | | | |
| solute carrier family 20 (phosphate transporter) member 1 | 2 | o | | | | | | | |
| solute carrier family 22 member 13-like | 2 | | o | | | | | | |
| solute carrier family 22 member 16-like | 2 | | o | | | | | | |
| solute carrier family 22 member 18 | 2 | | o | o | | | | | |
| solute carrier family 22 member 6-a | 2 | | | | | o | o | | |
| solute carrier family 22 member 7 | 2 | o | | | | o | | | |
| solute carrier family 25 (mitochondrial carrier citrate transporter) member 1 | 2 | | o | | | | | | |
| solute carrier family 25 (mitochondrial carrier dicarboxylate transporter) member 10 | 2 | | o | | | | | o | |
| solute carrier family 25 (mitochondrial carrier phosphate carrier) member 24 | 2 | o | | | | | | | |
| solute carrier family 25 member 34-like | 2 | | | | o | | | | |
| solute carrier family 25 member 48 | 2 | | o | | | | | | |
| solute carrier family 26 member 6-like | 2 | | | | | | | | o |
| solute carrier family 28 (sodium-coupled nucleoside transporter) member 1 | 2 | o | | | | | | | |
| solute carrier family 28 (sodium-coupled nucleoside transporter) member 2 | 2 | o | | o | | | | | |
| solute carrier family 30 (zinc transporter) member 5 | 2 | | o | | | | | | |
| solute carrier family 31 (copper transporters) member 1 | 2 | | o | o | | | | | |
| solute carrier family 37 (glycerol-3-phosphate transporter) member 1 | 2 | o | o | | | | | | |
| solute carrier family 39 (zinc transporter) member 7 | 2 | o | | | | | | | |
| solute carrier family 40 (iron-regulated transporter) member 1 | 2 | o | | o | | | | | |
| solute carrier family 6 (neurotransmitter glycine) member 9 | 2 | | | | | | o | o | |
| solute carrier family 7 (cationic amino acid y+ system) member isoform cra_a | 2 | o | | | | | o | | |
| solute carrier family anion member 2 (erythrocyte membrane protein band 3-like 1) | 2 | o | | | | | o | | |
| solute carrier family facilitated glucose transporter member 2 | 2 | | | | o | | | | |
| solute carrier family member 36 | 2 | o | | o | | | | | |
| solute carrier family member b3 | 2 | o | | | | | | | |
| solute carrier family member c1 | 2 | | | o | | | | o | |
| solute carrier family member e3 | 2 | o | | | | | | | |
| solute carrier family member e4 | 2 | o | | | | | o | | |
| solute carrier family member f4 | 2 | o | | | | | o | | |
| solute carrier family sodium bicarbonate transporter- member 10-like | 2 | | | | | | | o | |
| solute carrier organic anion transporter family member 1c1 | 2 | | o | | o | | | | |
| sorbin and sh3 domain containing 3 | 2 | o | | | | | | | |
| sorbitol dehydrogenase | 2 | | o | o | | | | | |
| sorcs receptor 1 | 2 | | | | | | o | o | |
| sortilin-related vps10 domain containing receptor 2 | 2 | | | | | | o | | |
| sorting nexin 10 | 2 | | | | o | | | | |
| sorting nexin 13 | 2 | o | | | | | | | |
| sorting nexin 17 | 2 | o | | | | | | | |
| sorting nexin 22 | 2 | o | o | | | | | | |
| sorting nexin 25 | 2 | | | | | o | | o | |

| | | | | | | | | | |
|---|---|---|---|---|---|---|---|---|---|
| sorting nexin 9 | 2 | o | o | | | | | | |
| sorting nexin-10-like | 2 | | | | o | | | | |
| soss complex subunit c | 2 | o | | o | | | | | |
| sparc cwcv and kazal-like domains proteoglycan 2 | 2 | | | | | | o | | o |
| sparc cwcv and kazal-like domains proteoglycan 3 | 2 | o | | o | | | | | |
| spastic paraplegia 21 homolog | 2 | o | | o | | | | | |
| spermatid perinuclear rna-binding | 2 | o | | | | | o | | |
| spermine synthase | 2 | o | | | | | | | o |
| sphingolipid delta -desaturase des1 | 2 | o | | | | | | o | |
| sphingomyelin phosphodiesterase-like | 2 | | | | o | | | | |
| spindle and kinetochore-associated protein 1 | 2 | | | | o | | | o | |
| spindlin 1 | 2 | | o | | | | o | | |
| splicing arginine serine-rich 12 | 2 | o | | | | | | | |
| splicing arginine serine-rich 15 | 2 | o | | | | | o | | |
| splicing factor 3b subunit 2 | 2 | | o | | | | | | |
| spna2 protein | 2 | | | | | | | | o |
| squamous cell carcinoma antigen recognized by t-cells 1 | 2 | | o | | | o | | | |
| squamous cell carcinoma antigen recognized by t-cells 3 | 2 | | | | o | | o | | |
| src family associated phosphoprotein 1 | 2 | o | | | | | | | |
| src homology 2 domain containing transforming protein d | 2 | o | | | | | | | |
| srp40 domain-containing protein | 2 | o | | | | | | | |
| srrm2 protein | 2 | o | | | | | | | |
| st3 beta-galactoside alpha- -sialyltransferase 5 | 2 | | o | | | | | | |
| st8 alpha-n-acetyl-neuraminide alpha- -sialyltransferase 3 | 2 | | | | | | o | | |
| stabilin 1 | 2 | o | | | | | | o | |
| stat-like protein | 2 | | | | | o | o | | |
| steroid 5 alpha-reductase 3 | 2 | o | | | | o | | | |
| sterol o-acyltransferase 2 | 2 | | o | | | | | | |
| steroleosin slo2-2 | 2 | o | | | | | | | |
| stress-associated endoplasmic reticulum protein 1 | 2 | | o | o | | | | | |
| stromal cell derived factor 4 | 2 | o | | | | | | | |
| stromal cell-derived factor 2 | 2 | | o | o | | | | | |
| stromal cell-derived factor 2-like protein 1-like | 2 | | | | o | | | | |
| structural maintenance of chromosomes flexible hinge domain containing 1 | 2 | | o | | | | | | |
| structural maintenance of chromosomes flexible hinge domain-containing protein 1 | 2 | | o | o | | | | | |
| structure specific recognition protein 1 | 2 | o | | o | | | | | |
| subfamily member 18 | 2 | o | | | | | | | |
| subfamily member 6 | 2 | | | | | o | | | o |
| subfamily member partial | 2 | | o | | | | | | |
| succinylglutamate desuccinylase aspartoacylase family protein | 2 | | | | o | | | | |
| sulfatase 2 | 2 | | | | | | o | o | |
| sulfotransferase 1c1 | 2 | | o | o | | | | | |
| sulfotransferase family cytosolic 2b member 1 | 2 | o | | | | | | o | |
| sulfotransferase member 1 | 2 | o | | o | | | | | |
| sun domain-containing protein 1 | 2 | | | | | | o | | o |
| superfast myosin heavy chain | 2 | o | | | | | | | |
| supervillin isoform 2 | 2 | o | | | | | | | |
| suppressor of 3-like 1 ( cerevisiae) | 2 | | o | | | | | | o |
| suppressor of cytokine signaling 3 | 2 | o | | | | | | | |

| | | | | | | | | | |
|---|---|---|---|---|---|---|---|---|---|
| surface antigen -like | 2 | o | | | | | | | o |
| surfeit 2 | 2 | o | | o | | | | | |
| surfeit locus protein 2 | 2 | | | | o | | | | |
| surp and g-patch domain-containing protein 1-like | 2 | | | | | | | | o |
| survival motor neuron protein 1 | 2 | o | | o | | | | | |
| sushi domain containing 1 | 2 | o | | | | | | | o |
| swi5-dependent recombination dna repair protein 1 homolog | 2 | | | | o | | | | |
| switch-associated protein 70 | 2 | o | | | | o | | | |
| symplekin | 2 | | | | | | | o | o |
| synapsin isoform cra_a | 2 | | | | | | | o | o |
| synaptojanin 1 | 2 | o | | | | | o | | |
| synaptonemal complex protein sc65 | 2 | o | | | | o | | | |
| synaptophysin | 2 | | | | | | o | o | |
| synaptopodin | 2 | o | | | | | | | |
| synaptoporin precursor | 2 | | | | | | | | o |
| synaptosomal-associated protein | 2 | | | | | | | o | o |
| synaptotagmin ix | 2 | o | | | | | o | | |
| syndecan 4 | 2 | | o | o | | | | | |
| synergin gamma-like | 2 | | o | | | | | | |
| synovial sarcoma chromosome 18 | 2 | o | | | | | o | | |
| syntabulin isoform a | 2 | | | | | o | | o | |
| syntaxin 4 | 2 | o | o | | | | | | |
| syntaxin 6 | 2 | o | | o | | | | | |
| syntaxin 7 | 2 | o | o | | | | | | |
| syntaxin binding protein 3 | 2 | o | o | | | | | | |
| syntaxin-1a (synaptotagmin-associated 35 kda protein) (neuron-specific antigen hpc-1) | 2 | o | o | | | | | | |
| t cell receptor beta variable 29 | 2 | o | | | | | | | |
| tacc2 protein | 2 | o | | | | | o | | |
| tapasin | 2 | o | | o | | | | | |
| tar dna binding protein | 2 | o | | | | | | | |
| target of myb protein 1-like | 2 | | | | o | | | | o |
| target of myb1 | 2 | o | o | | | | | | |
| tbc1 domain family member 30-like | 2 | | | | | | | | o |
| tbc1 domain family member 8 | 2 | | | | o | | | | |
| tbc1 domain member 17 | 2 | | o | o | | | | | |
| tbc1 domain member 30 | 2 | | | o | | o | | | |
| tbc1 domain member 8b | 2 | o | | | | | o | | |
| tbc1 domain member 9 (with gram domain) | 2 | o | | | | | o | | |
| t-box 15 | 2 | o | | | | | | | |
| tc1-like transposase | 2 | o | | | | o | | | |
| t-cell leukemia translocation-altered gene protein homolog | 2 | o | | o | | | | | |
| t-cell receptor alpha | 2 | o | | | | | | | |
| t-complex 11 like 1 | 2 | o | | o | | | | | |
| t-complex protein 1 subunit alpha | 2 | | | | | o | | | o |
| t-complex protein 1 subunit eta | 2 | | | | | | | | o |
| tea domain family member 3 | 2 | o | | | | | | | |
| teashirt homolog 3 | 2 | o | | | | | o | | |
| telethonin | 2 | o | | | | | | | |
| tenascin-x | 2 | o | | | | | | | |
| tenomodulin | 2 | o | | | | | | | |
| testis-expressed sequence 2 protein | 2 | | | | | | o | | o |
| tetraodon protein product cag00085 | 2 | o | o | | | | | | |
| tetraspanin 33 | 2 | o | | | | | | | |
| tetraspanin 5 | 2 | o | | | | | | o | |
| tetraspanin 6 | 2 | | | | o | | | | o |
| tetraspanin-33-like | 2 | | | | | | | | o |

| | | | | | | | | | |
|---|---|---|---|---|---|---|---|---|---|
| tetratricopeptide repeat domain 1 | 2 | o | | o | | | | | |
| tetratricopeptide repeat domain 36 | 2 | | o | | | | | | |
| tetratricopeptide repeat domain partial | 2 | o | | | | | | o | |
| tetratricopeptide repeat protein 17-like | 2 | | | o | | | o | | |
| tetratricopeptide repeat protein 27 | 2 | | | | o | | | | o |
| tetratricopeptide repeat protein 32 | 2 | o | o | | | | | | |
| tetratricopeptide repeat protein 35-like | 2 | | | | o | | | | |
| tgf-beta-activated kinase like | 2 | o | | | | | | | |
| thap domain-containing protein 1 | 2 | | | | o | o | | | |
| thap domain-containing protein 2 | 2 | | | o | | | | | |
| thiamin pyrophosphokinase 1 | 2 | | o | | o | | | | |
| thiamine triphosphatase | 2 | o | | o | | | | | |
| thioesterase superfamily member 4 | 2 | o | | | | | | | |
| thioredoxin 2 | 2 | o | o | | | | | | |
| thioredoxin domain containing 12 (endoplasmic reticulum) | 2 | o | o | | | | | | |
| thioredoxin domain-containing protein 15 precursor | 2 | | | | | | | | o |
| thioredoxin interacting protein | 2 | o | o | | | | | | |
| thioredoxin reductase cytoplasmic | 2 | | o | | | | | | |
| tho complex 1 | 2 | o | | o | | | | | |
| tho complex 5 | 2 | o | | | | | o | | |
| threonine synthase-like 2 | 2 | o | | | | o | | | |
| thrombin | 2 | | | | o | | | | o |
| thump domain containing 1 | 2 | o | | | | | o | | |
| thymidine kinase soluble | 2 | o | | | o | | | | |
| thymocyte nuclear protein 1 | 2 | | | | o | o | | | |
| thymosin beta-10 | 2 | | | | o | | | o | |
| thyroid hormone receptor interactor 12 | 2 | | | | | o | | o | |
| thyroid hormone receptor interactor 4 | 2 | o | o | | | | | | |
| tight junction protein zo-1 | 2 | o | | | | | o | | |
| tim21-like mitochondrial precursor | 2 | o | | | | | | o | |
| tim21-like mitochondrial-like | 2 | | | | o | | | | |
| tissue factor pathway inhibitor | 2 | | | o | | | | | |
| titin a | 2 | o | | | | | | | |
| tlc domain-containing protein 2 | 2 | o | | o | | | | | |
| tn3 protein | 2 | o | | | | | | | |
| tnfaip3 interacting protein 1 | 2 | | o | | | | | | o |
| tolloid-like 1 | 2 | | | | o | | | | |
| tousled-like kinase 1 | 2 | o | | | | | | | |
| toxin-1 precursor | 2 | | | | o | | | | |
| tpa: lim-domain-binding protein 4a | 2 | | | o | | | | o | |
| tpa_exp: polyprotein | 2 | | | o | | | | | |
| tpa_exp: transposase | 2 | | | o | | | | o | |
| tpm1 protein | 2 | o | | | | | | | |
| traf and tnf receptor associated protein | 2 | o | | o | | | | | |
| trafficking kinesin binding 2 | 2 | o | | | | | o | | |
| trafficking protein particle complex subunit 4 | 2 | | o | | | | | | o |
| trafficking protein particle complex subunit 5 | 2 | | o | | | | | | o |
| transcobalamin ii precursor | 2 | o | | o | | | | | |
| transcription activator brg1 | 2 | | | | | | o | | |
| transcription elongation factor b polypeptide 3 | 2 | o | o | | | | | | |
| transcription factor 7-like 1-a | 2 | o | | | | | | o | |
| transcription factor 7-like 2 | 2 | | | | | | o | | |
| transcription factor adf-1 | 2 | o | | | o | | | | |
| transcription factor btf3-like | 2 | | | | o | o | | | |
| transcription factor cp2-like 1 | 2 | | o | | | | | | |
| transcription factor iiia-like | 2 | | | | o | | | | |
| transcription factor rfx4-like | 2 | | | | | | | | o |

| | | | | | | | | | |
|---|---|---|---|---|---|---|---|---|---|
| transcription factor sp2 | 2 | o | | | o | | | | |
| transcription initiation factor iie subunit beta | 2 | | | | o | | | | o |
| transcription termination factor 1 | 2 | | | o | | o | | | |
| transcriptional regulator | 2 | o | | | o | | | | |
| transcriptional regulator atrx | 2 | | | | | | o | o | |
| transducin -like 1 x-linked | 2 | o | | | | | | | |
| transferrin receptor protein 1 | 2 | o | | o | | | | | |
| transforming growth beta 3 | 2 | o | | | | | | | |
| transgelin-3 | 2 | | | | | | | | o |
| translation initiation factor 5a | 2 | | | | o | | | o | |
| translation initiation factor if- mitochondrial precursor | 2 | o | | | | o | | | |
| translocase of inner mitochondrial membrane domain-containing protein 1 | 2 | | | | | | | | o |
| translocating chain-associated membrane protein 1 | 2 | o | | o | | | | | |
| translocon-associated protein subunit beta precursor | 2 | | o | o | | | | | |
| transmembrane 4 superfamily member 5 | 2 | | o | o | | | | | |
| transmembrane 4 superfamily member 8 isoform 1 | 2 | | o | | | o | | | |
| transmembrane 9 superfamily member 2-like | 2 | | o | | | | o | | |
| transmembrane and tpr repeat-containing protein 3-like | 2 | o | | | | | o | | |
| transmembrane and ubiquitin-like domain-containing protein 2-like | 2 | o | | o | | | | | |
| transmembrane bax inhibitor motif containing 1b | 2 | | o | o | | | | | |
| transmembrane bax inhibitor motif-containing protein 4-like | 2 | | | | o | | | | |
| transmembrane emp24 protein transport domain containing 7 | 2 | o | | o | | | | | |
| transmembrane prolyl 4-hydroxylase-like | 2 | | | | | | | o | o |
| transmembrane protein 104 | 2 | o | | | | | | | |
| transmembrane protein 126a | 2 | o | | o | | | | | |
| transmembrane protein 145 | 2 | | | | | | o | o | |
| transmembrane protein 159 | 2 | o | | | | o | | | |
| transmembrane protein 161a | 2 | o | | | | | | | |
| transmembrane protein 161b | 2 | o | | | | | o | | |
| transmembrane protein 163 | 2 | | | | | | o | o | |
| transmembrane protein 16c | 2 | | | | | | o | o | |
| transmembrane protein 17 | 2 | o | | | | o | | | |
| transmembrane protein 186 | 2 | o | | o | | | | | |
| transmembrane protein 203 | 2 | o | o | | | | | | |
| transmembrane protein 22 | 2 | | | | | o | | | o |
| transmembrane protein 63c | 2 | | | | | | o | o | |
| transmembrane protein 86a | 2 | | o | | | | | | |
| transmembrane protein adipocyte-associated 1 homolog | 2 | | o | | | | | | o |
| transmembrane protein c20orf108-like | 2 | | o | o | | | | | |
| transmembrane protein c6orf191-like | 2 | | | | o | o | | | |
| transmembrane protein c9orf46 | 2 | o | | o | | | | | |
| transportin 1 | 2 | o | | | | | | | |
| transposable element tcb2 transposase | 2 | | o | o | | | | | |
| transposase [Aedes aegypti] | 2 | o | | | | | | | |
| transposase element l1md-a101 l1md-a102 l1md-a2-like | 2 | | | | o | o | | | |
| transposase homolog | 2 | | | o | | | | o | |
| transposase yabusame-1 | 2 | o | | o | | | | | |
| treacle protein | 2 | o | | | | o | | | |

| | | | | | | | | | |
|---|---|---|---|---|---|---|---|---|---|
| tributyltin binding protein type 2-like protein | 2 | | o | | | | | | |
| trifunctional enzyme subunit mitochondrial-like | 2 | | | | o | | | | o |
| trinucleotide repeat containing 18 | 2 | | | | | | o | o | |
| trinucleotide repeat containing 6a | 2 | o | | | | | | | |
| trinucleotide repeat containing 6c | 2 | o | | | | | | | |
| tripartite motif containing 35-27 | 2 | | | | | | | | o |
| tripartite motif protein trim29-like | 2 | o | | | | | | | |
| tripartite motif-containing 2 | 2 | | o | | | o | | | |
| tripartite motif-containing 39 | 2 | | o | | | o | | | |
| tripartite motif-containing protein 13 | 2 | o | | o | | | | | |
| tripartite motif-containing protein 16-like | 2 | | | | | | | | o |
| tripartite motif-containing protein 2 | 2 | | | | | | o | | o |
| tripartite motif-containing protein 3-like | 2 | o | | | | | | | |
| tripartite motif-containing protein 60-like | 2 | o | | | | | | o | |
| trm1-like protein | 2 | o | | | | | | | o |
| trna methyltransferase 112 homolog | 2 | | | | o | | | | o |
| trna mitochondrial | 2 | | | | | | | | o |
| trna-dihydrouridine synthase 3-like | 2 | o | | | | | | | |
| trna-dihydrouridine synthase 4-like | 2 | o | | | o | | | | |
| trna-specific adenosine deaminase-like protein 3 | 2 | | | | | | | | o |
| troponin fast skeletal muscle | 2 | o | | | | | | | |
| troponin skeletal muscle | 2 | o | | | | | | | |
| troponin slow skeletal and cardiac muscles | 2 | o | | | | | | | |
| troponin slow skeletal muscle | 2 | o | | | | | | | |
| trypsinogen 1a | 2 | | o | | | | | | |
| trypsinogen 2 | 2 | | o | | | | | | |
| tsukushin precursor | 2 | o | o | | | | | | |
| tubulin beta | 2 | | | | | | | | o |
| tubulin beta-2a chain | 2 | | | | | | | | o |
| tuftelin-interacting protein 11 | 2 | o | | | o | | | | |
| tumor necrosis factor receptor member 14 (herpesvirus entry mediator) | 2 | | | | | o | | o | |
| tumor necrosis factor receptor superfamily member 14-like | 2 | | | | o | | | | |
| tumor necrosis factor receptor-2 | 2 | | | | | | | | o |
| tumor necrosis factor-inducible gene 6 | 2 | | | | | | | | o |
| tumor susceptibility gene 101 | 2 | o | o | | | | | | |
| tweety homolog 1 | 2 | | | | | | o | o | |
| two pore channel 3 | 2 | o | | | o | | | | |
| type i | 2 | o | | | | | o | | |
| type ii antifreeze protein | 2 | o | | | | | | | |
| type ii cytoskeletal 8 | 2 | o | o | | | | | | |
| type ii inositol- -bisphosphate 4-phosphatase | 2 | | | | | | o | o | |
| tyrosine 3-monooxygenase tryptophan 5-monooxygenase activation beta polypeptide like | 2 | | | | | o | | | o |
| tyrosine 3-monooxygenase tryptophan 5-monooxygenase activation theta polypeptide | 2 | | | | | | | | o |
| tyrosine kinase 2 | 2 | o | | | | | | o | |
| tyrosine kinase with immunoglobulin-like and egf-like domains 1 | 2 | o | | | | | | | |
| tyrosine-protein kinase 269 | 2 | o | | | | | | | |
| tyrosine-protein kinase jak1 | 2 | | o | | | | o | | |
| tyrosine-protein kinase receptor tie-1 precursor | 2 | o | o | | | | | | |
| tyrosine-protein kinase ryk | 2 | o | o | | | | | | |
| u11 u12 small nuclear ribonucleoprotein 25 kda protein | 2 | o | | | | | | | |

| | | | | | | | | | |
|---|---|---|---|---|---|---|---|---|---|
| u2 small nuclear rna auxiliary factor 2 | 2 | | | | | | o | | |
| u3 small nucleolar ribonucleoprotein protein mpp10 | 2 | o | o | | | | | | |
| u4 tri-snrnp-associated protein 2 | 2 | | o | | o | | | | |
| u4 u6 small nuclear ribonucleoprotein prp31 | 2 | | o | | | | | | |
| u5 small nuclear ribonucleoprotein 200 kda helicase | 2 | o | | | | | | | o |
| u6 snrna-associated sm-like protein lsm3 | 2 | o | | o | | | | | |
| u6 snrna-associated sm-like protein lsm7 | 2 | o | o | | | | | | |
| ubiquinone biosynthesis methyltransferase mitochondrial precursor | 2 | | o | | | | | | |
| ubiquinone biosynthesis monooxygenase coq6 | 2 | | o | o | | | | | |
| ubiquitin associated protein 2-like isoform 3 | 2 | o | | | | | o | | |
| ubiquitin carboxyl-terminal hydrolase 14 | 2 | o | | | o | | | | |
| ubiquitin carboxyl-terminal hydrolase 28 | 2 | o | | | | | | | |
| ubiquitin carboxyl-terminal hydrolase 4 | 2 | o | | | | | | | |
| ubiquitin carboxyl-terminal hydrolase 47 | 2 | | o | | | | o | | |
| ubiquitin carboxyl-terminal hydrolase 47 (ubiquitin thioesterase 47) (ubiquitin-specific-processing protease 47) (deubiquitinating enzyme 47) | 2 | o | | | | | o | | |
| ubiquitin carboxyl-terminal hydrolase 48 | 2 | o | | | | | | | |
| ubiquitin- containing phd and ring finger 1 | 2 | o | | | | | | | |
| ubiquitin-associated protein 2-like | 2 | | o | | | | | | o |
| ubiquitin-conjugating enzyme e2 c | 2 | o | o | | | | | | |
| ubiquitin-conjugating enzyme e2 t-like | 2 | | | | o | | | | o |
| ubiquitin-conjugating enzyme e2d 3-like | 2 | | | o | | | | o | |
| ubiquitin-conjugating enzyme e2h | 2 | o | | o | | | | | |
| ubiquitin-like modifier activating enzyme 3 | 2 | o | | | | | | | |
| ubiquitin-like protein fubi | 2 | o | o | | | | | | |
| ubiquitin-specific protease isoform a | 2 | o | | | | | | | |
| ubr3 protein | 2 | o | | | o | | | | |
| ubr4 protein | 2 | o | | | | | | | o |
| ubr5 protein | 2 | | | | | | | | o |
| ubx domain containing 2 | 2 | | | o | | | o | | |
| ubx domain containing 8 | 2 | o | | o | | | | | |
| ubx domain-containing protein 4 | 2 | | | | | | | o | o |
| udp-glucose ceramide glucosyltransferase-like 1 | 2 | o | | | | | o | | |
| udp-n-acetyl-alpha-d-galactosamine:polypeptide n-acetylgalactosaminyltransferase 8 ( c-t8) | 2 | | | o | | | | o | |
| udp-n-acteylglucosamine pyrophosphorylase 1 | 2 | | o | o | | | | | |
| unc-119 homolog ( elegans) | 2 | | | | | o | | o | |
| unc-13 homolog b ( elegans) | 2 | o | | | | | o | | |
| unc-93 homolog b1 ( elegans) | 2 | o | | o | | | | | |
| unc93-like protein mfsd11 isoform 1 | 2 | | | | o | | | | |
| uncharacterized aarf domain-containing protein kinase 2-like | 2 | | | | o | | | | |
| uncharacterized methyltransferase wbscr22-like | 2 | | | | o | | | | |
| uncharacterized protein c10orf18-like | 2 | | | | o | | | | |
| uncharacterized protein c15orf29-like | 2 | | o | | o | | | | |
| uncharacterized protein c19orf12 homolog | 2 | | | | | | | | o |
| uncharacterized protein c1orf109-like | 2 | | | | o | | | | |
| uncharacterized protein c1orf21 homolog | 2 | | | | | | | | o |
| uncharacterized protein c2orf24 homolog | 2 | o | | o | | | | | |
| uncharacterized protein c7orf44-like isoform 1 | 2 | o | | | | o | | | |

| | | | | | | | | | |
|---|---|---|---|---|---|---|---|---|---|
| uncharacterized protein c9orf114-like | 2 | o | | | | | | | |
| uncharacterized protein conserved in bacteria | 2 | o | | | | o | | | |
| uncharacterized protein iii | 2 | | | | o | | | | |
| uncharacterized protein kiaa0090-like isoform 2 | 2 | | | | | | o | o | |
| uncharacterized protein kiaa1467 homolog | 2 | | | | | | o | | o |
| uncharacterized protein kiaa1614-like | 2 | | | | | | | | o |
| uncharacterized protein kiaa1958-like | 2 | | | | o | | | | o |
| unconventional snare in the er 1 homolog | 2 | o | | o | | | | | |
| uncoupling protein 2 | 2 | o | o | | | | | | |
| uncoupling protein 4 | 2 | | o | | | | | | |
| upf0364 protein c6orf211 homolog | 2 | o | | | | | | o | |
| upf0428 protein cxorf56 homolog isoform 1 | 2 | | | | o | | | | |
| upf0436 protein c9orf6 homolog | 2 | | | | o | | | | |
| upf0436 protein c9orf6-like | 2 | | | | o | | | | o |
| upf0485 protein c1orf144 homolog | 2 | | | | | | | | o |
| upf0500 protein c1orf216 homolog | 2 | | | | | | | | o |
| upf0533 protein c5orf44-like protein | 2 | o | o | | | | | | |
| upf0545 protein c22orf39-like | 2 | o | | | | o | | | |
| upf0554 protein c2orf43 homolog | 2 | | o | | | o | | | |
| upf0577 protein kiaa1324-like | 2 | | | | | | | | o |
| upf0583 protein c15orf59 homolog | 2 | | | | | | | | o |
| upf0629 protein c17orf42 homolog | 2 | o | | o | | | | | |
| upf0631 protein c17orf108-like | 2 | | o | | | | o | | |
| upf0669 protein c6orf120 homolog | 2 | | | | o | | | | |
| upstream stimulatory factor 2 | 2 | o | o | | | | | | |
| uroporphyrinogen decarboxylase | 2 | o | | | o | | | | |
| uroporphyrinogen-iii synthase | 2 | | | o | | o | | | |
| usick-kaufman isoform cra_a | 2 | | | o | | o | | | |
| usp6 n-terminal like | 2 | o | | | | | | | |
| utrophin | 2 | o | | | o | | | | |
| vacuolar atp synthase catalytic subunit a | 2 | | | | o | | | | o |
| vacuolar protein sorting 13 homolog b | 2 | o | | | | | o | | |
| vacuolar protein sorting 13b isoform 1 | 2 | o | | | | | o | | |
| vacuolar protein sorting 16 | 2 | o | | o | | | | | |
| vacuolar protein sorting 26 homolog b | 2 | | o | | | | | | |
| vacuolar protein sorting-associated protein 26b-like | 2 | | | | | | o | | o |
| vacuolar protein sorting-associated protein 8 homolog | 2 | o | o | | | | | | |
| valacyclovir hydrolase-like | 2 | | | | | o | | | o |
| variable lymphocyte receptor a | 2 | | | o | | | | | |
| vascular endothelial growth factor a | 2 | o | | o | | | | | |
| vav 3 guanine nucleotide exchange factor | 2 | o | o | | | | | | |
| v-crk sarcoma virus ct10 oncogene homolog | 2 | o | | | | | o | | |
| vesicle transport protein got1a | 2 | | o | o | | | | | |
| vesicle transport protein got1b | 2 | o | | o | | | | | |
| vesicle transport protein sec20 | 2 | o | | o | | | | | |
| vesicle transport-related protein | 2 | o | | | | | | | |
| vesicle-associated membrane protein 2 | 2 | | | | | | o | o | |
| vesicle-associated membrane protein 8 | 2 | o | | o | | | | | |
| vesicle-trafficking protein sec22b | 2 | o | | o | | | | | |
| vesicular acetylcholine transporter | 2 | | | | | | | | o |
| v-ha-ras harvey rat sarcoma viral oncogene homolog | 2 | o | | | | | | o | |
| vhsv-induced protein | 2 | o | o | | | | | | |
| vitellogenin [Trematomus bernacchii] | 2 | | | o | | | | | |
| voltage-dependent anion-selective channel protein 1-like | 2 | | | | o | | | | |

| | | | | | | | | | |
|---|---|---|---|---|---|---|---|---|---|
| voltage-dependent calcium channel beta1 subunit | 2 | o | | | | | | | |
| voltage-dependent calcium channel subunit alpha-2 delta-2-like | 2 | | | | | | o | | |
| voltage-dependent p q-type calcium channel subunit alpha-1a | 2 | | | | | | o | | |
| von hippel-lindau disease tumor suppressor | 2 | o | o | | | | | | |
| von hippel-lindau disease tumor suppressor-like | 2 | | | | o | | | | |
| von willebrand factor c domain containing 2 | 2 | o | | | | | o | | |
| vps10 domain-containing receptor 1 | 2 | | | | | | o | | |
| vps13a protein | 2 | o | | | | | | | o |
| v-set and transmembrane domain-containing protein 2a | 2 | | | | | o | | o | |
| v-set domain containing t cell activation inhibitor 1 | 2 | o | | | | | | | |
| v-type proton atpase 116 kda subunit a isoform 1 | 2 | o | o | | | | | | |
| v-type proton atpase subunit f-like | 2 | | | | | | | | o |
| warm temperature acclimation-related 65 kda protein | 2 | | | o | o | | | | |
| wd repeat and socs box-containing protein 2 | 2 | | | | | o | o | | |
| wd repeat domain 17 | 2 | | | | | | o | | |
| wd repeat domain 48 | 2 | | o | | | | | | |
| wd repeat domain 54 | 2 | | o | | | o | | | |
| wd repeat domain 57 (u5 snrnp specific) | 2 | o | | | | | | | |
| wd repeat domain 68 | 2 | o | | | | | | | |
| wd repeat domain 75 | 2 | o | | | | | o | | |
| wd repeat-containing protein 26 | 2 | o | o | | | | | | |
| wd repeat-containing protein 27 | 2 | | | | o | | | | |
| wd repeat-containing protein 41 | 2 | o | | o | | | | | |
| wd repeat-containing protein 5 | 2 | | | | o | | | | o |
| wd repeat-containing protein 85 | 2 | o | o | | | | | | |
| wd repeat-containing protein 92 | 2 | o | | | | | | | |
| williams beuren syndrome chromosome region 22 | 2 | o | | o | | | | | |
| wilms tumor 1 associated protein | 2 | o | | | | | o | | |
| within bgcn homolog | 2 | o | | | | | | o | |
| wolfram syndrome 1 | 2 | o | | | | | | | |
| wsb1 protein | 2 | o | | | | | o | | |
| wu:fb59a11 protein | 2 | o | o | | | | | | |
| xaa-pro dipeptidase | 2 | | | | | | | | o |
| xin actin-binding repeat containing 1 | 2 | o | | | | | | | |
| xin actin-binding repeat-containing protein 1 | 2 | o | | | | | | | |
| xk-related protein 5 | 2 | | | | | | o | | |
| yes-associated protein 1 | 2 | o | | | | | | o | |
| ylp motif containing 1 | 2 | o | | | | o | | | |
| yrdc protein | 2 | o | o | | | | | | |
| yth domain containing 2 | 2 | | o | | | o | | | |
| zgc:103421 protein | 2 | o | | | | | o | | |
| zgc:165600 protein | 2 | | | | | o | | | o |
| zgc:171428 protein | 2 | o | | | | | | | |
| zgc:174877 protein | 2 | | | o | | | o | | |
| zgc:174888 protein | 2 | o | | o | | | | | |
| zgc:55448 protein | 2 | | | o | | | | o | |
| zinc cchc domain containing 24 | 2 | o | | | | | | | |
| zinc cchc domain containing 6 | 2 | o | | | | | | o | |
| zinc dhhc-type containing 18 | 2 | | o | | | | | o | |
| zinc finger 1 | 2 | o | | o | | | | | |
| zinc finger and btb domain containing 46 | 2 | o | | | | | | o | |

| | | | | | | | | | |
|---|---|---|---|---|---|---|---|---|---|
| zinc finger and btb domain-containing protein 2 | 2 | | | | | | | | o |
| zinc finger ccch domain-containing protein 7a | 2 | o | | | | o | | | |
| zinc finger ccch-type containing 7b | 2 | | o | o | | | | | |
| zinc finger cchc domain-containing protein 8 | 2 | | o | | | | | | o |
| zinc finger cchc-type and rna-binding motif-containing protein 1 | 2 | | | | | o | o | | |
| zinc finger e-box binding homeobox 1 | 2 | o | | | | | o | | |
| zinc finger mynd domain-containing protein 17 | 2 | o | | | | | | | |
| zinc finger partial | 2 | o | | | | | | | |
| zinc finger protein 106 | 2 | | | | | | o | o | |
| zinc finger protein 262 | 2 | | | o | | | | o | |
| zinc finger protein 271-like | 2 | | | | o | | | | o |
| zinc finger protein 300-like | 2 | o | | | | | | | |
| zinc finger protein 335 | 2 | | | | | | o | o | |
| zinc finger protein 347-like | 2 | o | | | | | | | |
| zinc finger protein 391 | 2 | | | | o | | | | |
| zinc finger protein 420-like | 2 | | o | | o | | | | |
| zinc finger protein 443 | 2 | o | | | | | | | |
| zinc finger protein 503 | 2 | o | o | | | | | | |
| zinc finger protein 507 | 2 | o | | | | | o | | |
| zinc finger protein 638 | 2 | o | | | | | | o | |
| zinc finger protein 670 | 2 | | o | | | o | | | |
| zinc finger protein 830 | 2 | o | | o | | | | | |
| zinc finger protein pegasus | 2 | | o | | | | | | |
| zinc finger protein zic 2 | 2 | | | | | o | | | o |
| zinc finger x-linked | 2 | o | | | | | | | |
| zinc fingers and homeoboxes protein 2 | 2 | | | | | | o | | o |
| zinc h2c2 domain containing | 2 | o | | | | | | | o |
| zinc hit type 1 | 2 | o | | | | | | | |
| zinc hit type 6 | 2 | o | | | | | | | |
| zinc knuckle protein | 2 | | | | | | o | | |
| zinc nfx1-type containing 1 | 2 | o | | | | o | | | |
| zinc phosphodiesterase elac protein 1 | 2 | | | | o | | | | |
| zinc transporter 9 | 2 | | o | | | | | | |
| zinc transporter zip8 | 2 | | o | o | | | | | |
| zona pellucida protein d precursor | 2 | | | | o | | | | |
| zpc domain containing protein 3 precursor | 2 | | | | o | | | | |
| zpc domain containing protein 5 | 2 | o | | | | | | | |
| zwilling a | 2 | o | | | | | | o | |
| zyxin | 2 | o | | | | | | o | |
| dna helicase q1-like | 1 | | | | | | | o | |
| 1110020g09rik protein | 1 | o | | | | | | | |
| 115 kda protein in type-1 retrotransposable element r1dm ( 115 kda protein in type i retrotransposable element r1dm) (orf 2) | 1 | o | | | | | | | |
| 116 kda u5 small nuclear ribonucleoprotein component isoform 2 | 1 | | | | o | | | | |
| 14-3-3 zeta | 1 | | | | o | | | | |
| 14kda- partial | 1 | | | | o | | | | |
| 1700123o20rik protein | 1 | o | | | | | | | |
| 1-acyl-sn-glycerol-3-phosphate acyltransferase delta | 1 | | o | | | | | | |
| 1-acyl-sn-glycerol-3-phosphate acyltransferase zeta precursor | 1 | | | | | | o | | |
| 1-o-acylceramide synthase precursor | 1 | o | | | | | | | |
| 1-phosphatidylinositol- -bisphosphate phosphodiesterase beta-4-like | 1 | | | | | | o | | |
| 2310042d19rik protein | 1 | o | | | | | | | |

| | | | | | | | | | |
|---|---|---|---|---|---|---|---|---|---|
| 26s protease regulatory subunit 8 | 1 | | | | | o | | | |
| 26s protease regulatory subunit s10b | 1 | | | | | | | o | |
| 26s proteasome non-atpase regulatory subunit 1 | 1 | | | | | | | | o |
| 26s proteasome non-atpase regulatory subunit 11 | 1 | | | | | | | | o |
| 26s proteasome non-atpase regulatory subunit 5 | 1 | | | | | | | | o |
| 27-kda protein | 1 | | | | | | | | o |
| 28 kda heat- and acid-stable phospho | 1 | | | | | | | | o |
| 2810459m11rik protein | 1 | | | o | | | | | |
| 2-aminoethanethiol dioxygenase | 1 | | | o | | | | | |
| 2-hydroxyacylsphingosine 1-beta-galactosyltransferase-like | 1 | | | | | | o | | |
| 2-oxoisovalerate dehydrogenase subunit mitochondrial | 1 | | | | | o | | | |
| 3 (2 ) -bisphosphate nucleotidase 1-like | 1 | | | | o | | | | |
| 3 -5 exonuclease with to crispr-associated protein | 1 | o | | | | | | | |
| 3 beta-hydroxysteroid dehydrogenase type 7-like | 1 | | o | | | | | | |
| 300 kda antigen | 1 | | | | | | | o | |
| 3-hydroxy-3-methylglutaryl-coenzyme a synthase 1 | 1 | | o | | | | | | |
| 3-hydroxyacyl- dehydratase 1 | 1 | o | | | | | | | |
| 3-hydroxyisobutyryl- mitochondrial precursor | 1 | | | | o | | | | |
| 3-hydroxyisobutyryl-coenzyme a hydrolase | 1 | | o | | | | | | |
| 3-hydroxymethyl-3-methylglutaryl-coenzyme a lyase | 1 | o | | | | | | | |
| 3-ketodihydrosphingosine reductase | 1 | | o | | | | | | |
| 3-keto-steroid reductase | 1 | | o | | | | | | |
| 3-mercaptopyruvate sulfurtransferase-like | 1 | | | | | | | | o |
| 3-oxo-5-alpha-steroid 4-dehydrogenase 1 | 1 | o | | | | | | | |
| 3-oxoacyl-acyl-carrier-protein reductase | 1 | | | o | | | | | |
| 3-phosphoinositide-dependent protein kinase 1 | 1 | o | | | | | | | |
| 4 repeat voltage-gated ion channel | 1 | | | | | | | o | |
| 40s ribosomal protein s10 | 1 | | | | | | | o | |
| 40s ribosomal protein s12 | 1 | | | | o | | | | |
| 40s ribosomal protein s18 | 1 | | | | | | | | o |
| 40s ribosomal protein s27-like | 1 | | | | o | | | | |
| 4631422o05rik protein | 1 | o | | | | | | | |
| 4-aminobutyrate mitochondrial | 1 | | | | | | | o | |
| 4-hydroxyphenylpyruvate dioxygenase-like | 1 | o | | | | | | | |
| 5 - cytosolic ia | 1 | o | | | | | | | |
| 5 -3 exoribonuclease 1 | 1 | o | | | | | | | |
| 5 -3 exoribonuclease 2 | 1 | o | | | | | | | |
| 5 -amp-activated protein kinase catalytic subunit alpha-2 | 1 | | | | | | o | | |
| 5 -amp-activated protein kinase subunit beta-1 | 1 | | | | | | | o | |
| 5 -amp-activated protein kinase subunit beta-2 | 1 | | | | | | | | o |
| 5 -amp-activated protein kinase subunit gamma-1 | 1 | | o | | | | | | |
| 5 -amp-activated protein kinase subunit gamma-3-like | 1 | | | | o | | | | |
| 5 -nucleotidase domain containing 1 | 1 | | | | | | o | | |
| 5 -nucleotidase precursor | 1 | o | | | | | | | |

| | | | | | | | | | |
|---|---|---|---|---|---|---|---|---|---|
| 52 kd repressor of inhibitor of protein | 1 | | | | | o | | | |
| 52 kda repressor of the inhibitor of the protein | 1 | o | | | | | | | |
| 52 kda repressor of the inhibitor of the protein kinase | 1 | o | | | | | | | |
| 52 kda repressor of the inhibitor of the protein kinase-like | 1 | | | | o | | | | |
| 55 kda erythrocyte membrane isoform 2 | 1 | | | | o | | | | |
| 5-azacytidine-induced protein 1-like | 1 | | | | | | o | | |
| 5-hydroxyisourate hydrolase-like | 1 | | o | | | | | | |
| 5-hydroxytryptamine receptor 2b | 1 | o | | | | | | | |
| 6-phosphofructokinase liver type | 1 | | o | | | | | | |
| 6-phosphofructokinase liver type-like | 1 | | o | | | | | | |
| 60 kda heat shock mitochondrial precursor | 1 | | o | | | | | | |
| 60 kda heat shock mitochondrial-like | 1 | | | | | | o | | |
| 60s ribosomal protein l12-like | 1 | | | | o | | | | |
| 60s ribosomal protein l23 | 1 | | | | o | | | | |
| 60s ribosomal protein l29 | 1 | | | | o | | | | |
| 60s ribosomal protein l29-like | 1 | o | | | | | | | |
| 60s ribosomal protein l31-like | 1 | | o | | | | | | |
| 60s ribosomal protein l37-like | 1 | | | | o | | | | |
| 60s ribosomal protein l7-like 1-like | 1 | | | | o | | | | |
| 60s ribosomal protein l9-like | 1 | | | | o | | | | |
| 6-phosphogluconate decarboxylating | 1 | | | o | | | | | |
| 6-phosphogluconolactonase | 1 | o | | | | | | | |
| 7 chain molecular models of averaged rigor crossbridges from tomograms of insect flight muscle | 1 | | | | o | | | | |
| 72 kda type iv collagenase | 1 | | o | | | | | | |
| 78 kda glucose-regulated protein | 1 | | | o | | | | | |
| 7-alpha-hydroxycholest-4-en-3-one 12-alpha-hydroxylase-like | 1 | | o | | | | | | |
| a chain crystal structures of hsc70bag1 in complex with small molecule inhibitors | 1 | | | | | | | | o |
| a chain crystal structures of human pyruvate dehydrogenase kinase 2 containing physiological and synthetic ligands | 1 | | | | | | o | | |
| a chain discovery of cell-active phenyl-imidazole pin1 inhibitors by structure-guided fragment evolution | 1 | | o | | | | | | |
| a chain matrix metalloproteinase-13 complexed with non-zinc binding inhibitor | 1 | | | | o | | | | |
| a chain tricyclic series of hsp90 inhibitors | 1 | | | | | | | | o |
| a chain trypsin specificity as elucidated by lie x- ray structures and association constant measurements | 1 | | o | | | | | | |
| a disintegrin and metalloproteinase domain 8 | 1 | o | | | | | | | |
| a kinase anchor protein 9 | 1 | | | | | o | | | |
| a1 adenosine receptor | 1 | o | | | | | | | |
| aarf domain containing kinase 5 | 1 | | o | | | | | | |
| abc transporter moat-c | 1 | o | | | | | | | |
| abhydrolase domain containing 3 | 1 | | | | | | | o | |
| abhydrolase domain-containing protein 12b-like | 1 | | | | o | | | | |
| abhydrolase domain-containing protein 14a-like | 1 | | | | | | | | o |
| abhydrolase domain-containing protein 8 | 1 | | | | o | | | | |
| abhydrolase domain-containing protein mitochondrial | 1 | | o | | | | | | |

| | | | | | | | | | |
|---|---|---|---|---|---|---|---|---|---|
| abhydrolase domain-containing protein mitochondrial-like | 1 | | | | o | | | | |
| abi gene member 3 binding protein isoform 4 | 1 | o | | | | | | | |
| abi member 3 binding protein | 1 | o | | | | | | | |
| abl interactor 2 isoform 12 | 1 | | | | | | o | | |
| abl-interactor 1 isoform 5 | 1 | o | | | | | | | |
| abl-interactor isoform cra_a | 1 | | | o | | | | | |
| absent in melanoma 1 protein | 1 | | | | o | | | | |
| absent in melanoma 1-like | 1 | o | | | | | | | |
| ac006551_24very retrotransposon reverse transcriptase | 1 | | | | | | | o | |
| ac087333_3high choriolytic enzyme 1 precursor | 1 | o | | | | | | | |
| acadvl protein | 1 | | o | | | | | | |
| accs protein | 1 | o | | | | | | | |
| acetyl- acetyltransferase 2 | 1 | | o | | | | | | |
| acetylcholine receptor beta subunit | 1 | o | | | | | | | |
| acetylcholine receptor subunit delta precursor | 1 | o | | | | | | | |
| acetyl-coenzyme a acyltransferase 2 | 1 | | | | | o | | | |
| acid alpha glucosidase | 1 | o | | | | | | | |
| acid phosphatase lysophosphatidic | 1 | o | | | | | | | |
| acid phosphatase soluble | 1 | | | | | | o | | |
| acid phosphatase-like 2 | 1 | | | | | o | | | |
| acid trehalase-like 1 | 1 | o | | | | | | | |
| acidic coiled-coil containing protein 2-like | 1 | o | | | | | | | |
| acidic fibroblast growth factor intracellular-binding | 1 | | | | o | | | | |
| acidic leucine-rich nuclear phosphoprotein 32 family member a | 1 | o | | | | | | | |
| acidic leucine-rich nuclear phosphoprotein 32 family member a-like | 1 | | | | o | | | | |
| acidic repeat-containing protein | 1 | | | | o | | | | |
| actin binding lim protein family member 2 | 1 | o | | | | | | | |
| actin binding protein (scraps drosophila) | 1 | o | | | | | | | |
| actin binding protein 1c | 1 | o | | | | | | | |
| actin binding protein isoform cra_a | 1 | | | | | | | o | |
| actin related protein 2 3 complex subunit 2 | 1 | | o | | | | | | |
| actin-binding lim protein 1 long isoform isoform cra_a | 1 | | | | | | | o | |
| actinin alpha 3 | 1 | o | | | | | | | |
| actinin alpha 4 | 1 | o | | | | | | | |
| actin-like 6a | 1 | | | o | | | | | |
| actin-related protein 2 3 complex subunit 1a | 1 | | | | | o | | | |
| actin-related protein 2 3 complex subunit 5 | 1 | o | | | | | | | |
| actin-related protein 8-like | 1 | | | | o | | | | |
| activated cdc42 kinase 1-like | 1 | | | | | | | | o |
| activated rna polymerase ii transcriptional coactivator p15-like | 1 | | | | o | | | | |
| activating signal cointegrator 1 complex subunit 2 | 1 | o | | | | | | | |
| activating transcription factor 1 | 1 | | o | | | | | | |
| activating transcription factor 7 interacting protein 2 | 1 | o | | | | | | | |
| activator of heat shock 90kda protein atpase homolog 1 | 1 | | | | | o | | | |
| activator of s phase kinase | 1 | | o | | | | | | |
| active breakpoint cluster region-related protein | 1 | | | | | | o | | |
| active regulator of sirt1-like | 1 | | o | | | | | | |
| activin a type i | 1 | o | | | | | | | |

| | | | | | | | | | |
|---|---|---|---|---|---|---|---|---|---|
| activin a type ib | 1 | | o | | | | | | |
| activin receptor type-1 precursor | 1 | | o | | | | | | |
| activity-dependent neuroprotector | 1 | o | | | | | | | |
| actp1_sagro ame: full=cytolysin src-1 ame: full=src i flags: precursor | 1 | o | | | | | | | |
| acyl- -binding domain-containing protein 5-like | 1 | | o | | | | | | |
| acyl -desaturase | 1 | | | | | | | | o |
| acyl- desaturase-like | 1 | | | | o | | | | |
| acyl- oxidase palmitoyl | 1 | | | | | | | o | |
| acyl- synthetase bubblegum family member 1 | 1 | | | | | | | o | |
| acyl- synthetase family member 2 | 1 | | o | | | | | | |
| acyl- synthetase partial | 1 | | | | | | | o | |
| acyl- synthetase short-chain family member 1 | 1 | o | | | | | | | |
| acyl- synthetase short-chain family member 3 | 1 | o | | | | | | | |
| acyl- thioesterase | 1 | | o | | | | | | |
| acyl- thioesterase 7 | 1 | | | | | o | | | |
| acylamino-acid-releasing enzyme-like | 1 | | | | | | | | o |
| acyl-coenzyme a binding domain containing 5 | 1 | | o | | | | | | |
| acylphosphatase erythrocyte type | 1 | | | | | o | | | |
| acylphosphatase-2 | 1 | o | | | | | | | |
| acyl-protein thioesterase 2 | 1 | o | | | | | | | |
| ad012w | 1 | | | | | | | | o |
| adam metallopeptidase domain 10 | 1 | o | | | | | | | |
| adam metallopeptidase domain 17 | 1 | | | | | | | o | |
| adam metallopeptidase domain 22 | 1 | | | | | | | o | |
| adam metallopeptidase with thrombospondin type 1 12 | 1 | o | | | | | | | |
| adaptin ear-binding coat-associated protein 1 | 1 | | | | | | | | o |
| adaptor-related protein complex alpha 2 subunit | 1 | o | | | | | | | |
| adaptor-related protein complex sigma 2 subunit | 1 | o | | | | | | | |
| add2 protein | 1 | | | | | | o | | |
| additional sex combs like 2 | 1 | o | | | | | | | |
| adenomatous polyposis coli protein 2 | 1 | | | | | | o | | |
| adenosine a1 receptor | 1 | o | | | | | | | |
| adenosine deaminase cecr1-a-like | 1 | | | | o | | | | |
| adenosine deaminase domain-containing protein 1-like isoform 2 | 1 | | | | o | | | | |
| adenosine deaminase-like | 1 | | | | | | | | o |
| adenosine monophosphate-protein transferase ficd | 1 | | | | o | | | | |
| adenosine receptor a1 | 1 | | | | o | | | | |
| adenosine rna-specific | 1 | o | | | | | | | |
| adenosylhomocysteinase a-like | 1 | | o | | | | | | |
| adenylate cyclase 6 | 1 | o | | | | | | | |
| adenylate cyclase 9 | 1 | o | | | | | | | |
| adenylate cyclase type 1 | 1 | | | | | | o | | |
| adenylate cyclase type 6 | 1 | o | | | | | | | |
| adenylate kinase 3 | 1 | | | | | | | o | |
| adenylate kinase 3-like 1 | 1 | o | | | | | | | |
| adenylate kinase 7 | 1 | | | | | o | | | |
| adenylate kinase isoenzyme 5 | 1 | | | | o | | | | |
| adenylate kinase isoenzyme 6 | 1 | | o | | | | | | |
| adenylate kinase isoenzyme 6-like | 1 | | | | | | | | o |
| adenylate kinase isoenzyme mitochondrial | 1 | | o | | | | | | |
| adenylosuccinate synthase like 1 | 1 | | | | | | | | o |
| adenylyl cyclase 35c-like | 1 | | | | | | | o | |
| adenylyl cyclase-associated protein 2 | 1 | | | | | o | | | |

| | | | | | | | | | |
|---|---|---|---|---|---|---|---|---|---|
| adf-1 l-orf | 1 | o | | | | | | | |
| adhesion molecule with ig-like domain 1 | 1 | | | | | | | o | |
| adhesion regulating molecule 1 | 1 | | | | | o | | | |
| adhesive plaque matrix partial | 1 | | | | o | | | | |
| adiponectin receptor protein 1 | 1 | | | | | | | | o |
| adipose differentiation-related protein | 1 | | o | | | | | | |
| adp atp translocase | 1 | | | | | | | | o |
| adp atp translocase 2-like | 1 | | | | | | | | o |
| adp-ribosyl cyclase 1-like | 1 | o | | | | | | | |
| adp-ribosylarginine hydrolase | 1 | o | | | | | | | |
| adp-ribosylation factor gtpase activating protein 2 | 1 | | o | | | | | | |
| adp-ribosylation factor interacting protein 1 (arfaptin 1) | 1 | | | | | | | o | |
| adp-ribosylation factor interacting protein 2b | 1 | | | | | | | o | |
| adp-ribosylation factor related protein 1 | 1 | o | | | | | | | |
| adp-ribosylation factor-binding protein gga3 | 1 | | | | | o | | | |
| adp-ribosylation factor-like 4 | 1 | | o | | | | | | |
| adp-ribosylation factor-like 6 interacting protein 2 | 1 | o | | | | | | | |
| adp-ribosylation factor-like 7 | 1 | o | | | | | | | |
| adp-ribosylation factor-like protein 5a | 1 | | | o | | | | | |
| adp-ribosylation factor-like protein 6 | 1 | | | | | | o | | |
| adp-ribosylation factor-like protein 6-interacting protein 1 | 1 | | o | | | | | | |
| adp-ribosylation factor-like protein 6-interacting protein 4-like | 1 | | o | | | | | | |
| adp-ribosylation factor-like protein 8a | 1 | | | | | | o | | |
| adp-ribosylhydrolase like 1 | 1 | o | | | | | | | |
| adrenocortical alacrimia | 1 | | o | | | | | | |
| adrenodoxin-like mitochondrial precursor | 1 | o | | | | | | | |
| adrenomedullin 2 | 1 | o | | | | | | | |
| adrenomedullin 3 | 1 | | o | | | | | | |
| af128810_1zpc domain containing protein 2 | 1 | o | | | | | | | |
| af255564_1alpha tubulin | 1 | | | | | | | o | |
| af255955_1beta tubulin | 1 | | | | | | | | o |
| af303557_1immunoglobulin heavy chain | 1 | | | o | | | | | |
| af303563_1immunoglobulin heavy chain | 1 | | | o | | | | | |
| AF318317_1unknown [Homo sapiens] | 1 | | | | | | o | | |
| af4 fmr2 family member 1-like | 1 | o | | | | | | | |
| af4 fmr2 family member 4 | 1 | | | | o | | | | |
| af4 fmr2 member 1 isoform 2 | 1 | o | | | | | | | |
| af465280_1coagulation factor v precursor | 1 | | o | | | | | | |
| af503912_2envelope protein | 1 | | | | | | o | | |
| afadin- and alpha-actinin-binding | 1 | | | | o | | | | |
| afadin- and alpha-actinin-binding protein | 1 | | | | o | | | | |
| agap002775 protein | 1 | | | o | | | | | |
| AGAP013147-PA [Anopheles gambiae str. PEST] | 1 | | | | o | | | | |
| agmatine ureohydrolase isoform cra_c | 1 | | o | | | | | | |
| agouti related protein 2 | 1 | | | | | | o | | |
| agtpbp1 protein | 1 | | | | | | o | | |
| a-kinase anchor protein 13 | 1 | o | | | | | | | |
| a-kinase anchor protein 2 isoform 2 | 1 | o | | | | | | | |
| akirin 1 | 1 | o | | | | | | | |
| alanine aminotransferase 2 | 1 | | | o | | | | | |
| alanine and arginine-rich domain-containing | 1 | | | | | | | | o |
| alanine n-terminal | 1 | | o | | | | | | |
| alanine--glyoxylate aminotransferase | 1 | | o | | | | | | |
| alanine-glyoxylate aminotransferase 2-like 1 | 1 | | o | | | | | | |

| | | | | | | | | | |
|---|---|---|---|---|---|---|---|---|---|
| alanine--glyoxylate aminotransferase 2-like 1-like | 1 | | | | o | | | | |
| alanyl-trna cytoplasmic-like | 1 | | | | | | | | o |
| alcohol dehydrogenase 1-like | 1 | | | | o | | | | |
| alcohol dehydrogenase family-1 | 1 | o | | | | | | | |
| alcohol iron 1 | 1 | | o | | | | | | |
| aldehyde dehydrogenase 1 member b1 | 1 | | | | o | | | | |
| aldehyde dehydrogenase 1 member l1 | 1 | o | | | | | | | |
| aldehyde dehydrogenase 8 member a1 | 1 | | o | | | | | | |
| aldehyde mitochondrial | 1 | | | | o | | | | |
| aldehyde oxidase | 1 | | o | | | | | | |
| aldolase c | 1 | | | | | | | o | |
| aldolase fructose-bisphosphate | 1 | | | | | | | | o |
| aldose 1-epimerase | 1 | | o | | | | | | |
| alg3 protein | 1 | o | | | | | | | |
| alkyl hydroperoxide reductase thiol specific antioxidant mal allergen | 1 | | | | | | | | o |
| alkylated dna repair protein alkb homolog 1-like | 1 | | | | o | | | | |
| alkylated dna repair protein alkb homolog 7 | 1 | | | | | o | | | |
| alkylation repair homolog 5 ( coli) | 1 | o | | | | | | | |
| alkylglycerone phosphate synthase | 1 | o | | | | | | | |
| alpha (50kda dystrophin-associated glycoprotein) | 1 | o | | | | | | | |
| alpha 1 (dystrophin-associated protein acidic component) | 1 | o | | | | | | | |
| alpha actin | 1 | o | | | | | | | |
| alpha beta hydrolase fold | 1 | | | o | | | | | |
| alpha enolase | 1 | | | | | | | | o |
| alpha globin regulatory element containing protein | 1 | o | | | | | | | |
| alpha-mannosyl-glycoprotein 4-beta-n-acetylglucosaminyltransferase a | 1 | | | | | | | | o |
| alpha-mannosyltransferase alg9-like | 1 | | | o | | | | | |
| alpha-sialyltransferase | 1 | | | o | | | | | |
| alpha-sialyltransferase st3gal ii | 1 | o | | | | | | | |
| alpha-sialyltransferase st3gal v | 1 | | | o | | | | | |
| alpha-sialyltransferase st8sia v | 1 | | | | | | o | | |
| alpha thalassaemia mental retardation x-linked protein | 1 | o | | | | | | | |
| alpha thalassemia mental retardation syndrome x-linked | 1 | | | | | | o | | |
| alpha thalassemia mental retardation syndrome x-linked (rad54 cerevisiae) | 1 | o | | | | | | | |
| alpha thalassemia mental retardation syndrome x-linked homolog | 1 | o | | | | | | | |
| alpha-fucosyltransferase 11 precursor | 1 | | | | | | | | o |
| alpha-1-antitrypsin [Epinephelus coioides] | 1 | | o | | | | | | |
| alpha1a-voltage-dependent calcium channel | 1 | | | | | | | o | |
| alpha-2a- receptor | 1 | | | | o | | | | |
| alpha-2-antiplasmin-like isoform 1 | 1 | | o | | | | | | |
| alpha-2-macroglobulin receptor | 1 | | | o | | | | | |
| alpha-2-macroglobulin-like isoform 2 | 1 | | o | | | | | | |
| alpha3-fucosyltransferase | 1 | o | | | | | | | |
| alpha-actinin-1-like isoform 1 | 1 | | | | | | o | | |
| alpha-actinin-4-like isoform 1 | 1 | | | | | | o | | |
| alpha-aminoadipate aminotransferase | 1 | | o | | | | | | |
| alpha-crystallin b chain | 1 | o | | | | | | | |
| alpha-dystroglycan | 1 | o | | | | | | | |
| alpha-galactosidase a | 1 | | | | | o | | | |

| | | | | | | | | | |
|---|---|---|---|---|---|---|---|---|---|
| alpha-n-acetylgalactosaminidase precursor | 1 | | | o | | | | | |
| alpha-synuclein | 1 | | | | | | | o | |
| alpha-tocopherol transfer protein | 1 | | o | | | | | | |
| alphatub84b-prov protein | 1 | | | | | | | | o |
| alpha-tubulin | 1 | | | o | | | | | |
| alport mental midface hypoplasia and elliptocytosis chromosomal gene 1 | 1 | o | | | | | | | |
| alternative oxidase | 1 | o | | | | | | | |
| alveolar soft part sarcoma chromosome candidate 1 | 1 | | o | | | | | | |
| amidohydrolase domain containing 2 | 1 | o | | | | | | | |
| amiloride-sensitive amine oxidase | 1 | | o | | | | | | |
| amiloride-sensitive cation channel neuronal | 1 | | | | | | o | | |
| amiloride-sensitive cation channel pituitary | 1 | | | | | | o | | |
| amine copper containing 3 | 1 | o | | | | | | | |
| amine oxidase | 1 | | o | | | | | | |
| amino acid permease | 1 | | o | | | | | | |
| aminoacyl trna synthetase complex-interacting multifunctional protein 1 | 1 | o | | | | | | | |
| aminoadipate-semialdehyde dehydrogenase-phosphopantetheinyl transferase | 1 | o | | | | | | | |
| aminopeptidase puromycin sensitive | 1 | o | | | | | | | |
| aminophospholipid transporter class type member 1 | 1 | | | | | o | | | |
| ammonium transporter rh type a | 1 | o | | | | | | | |
| amp deaminase 3 | 1 | o | | | | | | | |
| ampa selective glutamate receptor | 1 | | | | | | | o | |
| amp-dependent synthetase and ligase | 1 | | o | | | | | | |
| amphiphysin isoform 2 | 1 | | | | | | | o | |
| amyloid beta a4 partial | 1 | | | | | | | | o |
| amyloid beta a4 precursor protein-binding family a member 1 | 1 | | | | o | | | | |
| amyloid beta a4 precursor protein-binding family a member 2-like | 1 | | | | | o | | | |
| amyloid beta a4 precursor protein-binding family a member 2-like isoform 2 | 1 | | | | | | | | o |
| amyloid beta a4 precursor protein-binding family a member 3 | 1 | | | | | | | | o |
| amyloid beta a4 precursor protein-binding family b member 1 | 1 | o | | | | | | | |
| amyloid beta a4 precursor protein-binding family b member 2 | 1 | | | | | | | o | |
| amyloid beta a4 precursor protein-binding family b member 2-like | 1 | | | | | | o | | |
| amyloid beta precursor protein- family member 1 interacting protein | 1 | | o | | | | | | |
| amyloid beta precursor protein- family member 2-like | 1 | o | | | | | | | |
| amyotrophic lateral sclerosis 2 chromosome candidate 2 | 1 | o | | | | | | | |
| an1-type zinc finger protein 3 | 1 | o | | | | | | | |
| an1-type zinc finger protein 3-like | 1 | | | | | | | | o |
| anaphase promoting complex subunit 1 | 1 | | | | | | | o | |
| anaphase promoting complex subunit 4 | 1 | | o | | | | | | |
| anaphase-promoting complex subunit 1 | 1 | | | | | | | | o |
| anaphase-promoting complex subunit 2 | 1 | | | | o | | | | |
| anaphase-promoting complex subunit 7 | 1 | | o | | | | | | |
| and ph domain containing 1 | 1 | o | | | | | | | |
| and ph domain containing 5 | 1 | | o | | | | | | |
| and ph domain-containing protein 3-like | 1 | | | | o | | | | |

| | | | | | | | | | |
|---|---|---|---|---|---|---|---|---|---|
| and ph domain-containing protein 4 | 1 | | o | | | | | | |
| and ph domain-containing protein 5 (zinc finger fyve domain-containing protein 23) | 1 | o | | | | | | | |
| and pleckstrin domain protein 2 | 1 | o | | | | | | | |
| and pleckstrin domain-containing protein 1 | 1 | | | | | | o | | |
| androgen receptor alpha | 1 | o | | | | | | | |
| androgen receptor beta | 1 | o | | | | | | | |
| angiogenin 4 | 1 | o | | | | | | | |
| angiomotin-like protein 2 | 1 | | | | o | | | | |
| angiomotin-like protein 2-like | 1 | | | | | | | | o |
| angiopoietin-like 5 | 1 | o | | | | | | | |
| angiopoietin-like 7 | 1 | o | | | | | | | |
| angiopoietin-related protein 4 precursor | 1 | | o | | | | | | |
| angiotensin converting partial | 1 | | | o | | | | | |
| angiotensin i converting enzyme (peptidyl-dipeptidase a) 1 | 1 | | o | | | | | | |
| ank accession number ab073376 non-ltr retrotransposable element partially supported by genscan in oryzias latipes | 1 | o | | | | | | | |
| ank1 protein | 1 | | | | | | | o | |
| ankyrin 3 | 1 | | | | | | o | | |
| ankyrin isoform cra_a | 1 | o | | | | | | | |
| ankyrin isoform cra_b | 1 | | | | | | | o | |
| ankyrin isoform cra_e | 1 | | | | | | o | | |
| ankyrin isoform cra_i | 1 | | | | | | o | | |
| ankyrin repeat and btb poz domain-containing protein btbd11-like | 1 | o | | | | | | | |
| ankyrin repeat and death domain containing 1a | 1 | o | | | | | | | |
| ankyrin repeat and kinase domain containing 1 | 1 | o | | | | | | | |
| ankyrin repeat and socs box protein 12 | 1 | | | | | | | | o |
| ankyrin repeat and socs box protein 2 | 1 | o | | | | | | | |
| ankyrin repeat and socs box protein 8 | 1 | o | | | | | | | |
| ankyrin repeat and socs box-containing 14 | 1 | o | | | | | | | |
| ankyrin repeat and socs box-containing 4 | 1 | o | | | | | | | |
| ankyrin repeat and socs box-containing 6 | 1 | o | | | | | | | |
| ankyrin repeat and sterile alpha motif domain containing 6 | 1 | o | | | | | | | |
| ankyrin repeat domain 13b | 1 | | | | | | o | | |
| ankyrin repeat domain 2 (stretch responsive muscle) | 1 | o | | | | | | | |
| ankyrin repeat domain 28 | 1 | | | | | | | o | |
| ankyrin repeat domain-containing protein 1 | 1 | o | | | | | | | |
| ankyrin repeat domain-containing protein 11-like | 1 | | | | | | o | | |
| ankyrin repeat domain-containing protein 13d | 1 | | o | | | | | | |
| ankyrin repeat domain-containing protein 27 | 1 | | | | | | o | | |
| ankyrin repeat domain-containing protein 39 | 1 | o | | | | | | | |
| ankyrin repeat domain-containing protein 40-like | 1 | | o | | | | | | |
| ankyrin repeat domain-containing protein 57 | 1 | o | | | | | | | |
| ankyrin repeat family a protein 2 | 1 | | | | | | | | o |
| anoctamin-10 isoform 3 | 1 | | | | | | | | o |
| anterior gradient protein 3 homolog precursor | 1 | o | | | | | | | |
| anterior pharynx defective 1 homolog a ( elegans) | 1 | | | | o | | | | |
| anti-apoptotic protein nr13 | 1 | o | | | | | | | |
| antigen cd18 | 1 | o | | | | | | | |
| antigen identified by monoclonal antibody ki-67 | 1 | o | | | | | | | |

| | | | | | | | | | |
|---|---|---|---|---|---|---|---|---|---|
| ap-1 complex subunit beta-1 isoform 3 | 1 | | | | | | o | | |
| ap-1 complex subunit gamma-1-like isoform 1 | 1 | | | | o | | | | |
| ap-1 complex subunit mu-1-like isoform 2 | 1 | | o | | | | | | |
| ap1 endonuclease | 1 | o | | | | | | | |
| ap1 gamma subunit binding protein 1 | 1 | | | | | | o | | |
| ap-2 complex subunit alpha-2 | 1 | o | | | | | | | |
| ap-2 complex subunit sigma | 1 | | | | | | | | o |
| ap-3 complex subunit beta-1 | 1 | o | | | | | | | |
| ap-4 complex subunit sigma-1 | 1 | | | | | | | | o |
| apaf1-interacting protein homolog | 1 | | | | | | o | | |
| apelin receptor a | 1 | o | | | | | | | |
| apex nuclease (apurinic apyrimidinic endonuclease) 2 | 1 | o | | | | | | | |
| apical protein | 1 | o | | | | | | | |
| ap-like endonuclease reverse transcriptase | 1 | o | | | | | | | |
| apolipoprotein ai | 1 | | | | o | | | | |
| apolipoprotein a-i binding protein | 1 | o | | | | | | | |
| apolipoprotein a-iv precursor | 1 | | | | o | | | | |
| apolipoprotein a-iv3 | 1 | | | o | | | | | |
| apolipoprotein b 100 | 1 | | o | | | | | | |
| apolipoprotein b precursor | 1 | | o | | | | | | |
| apolipoprotein b-100 precursor | 1 | | | | | | o | | |
| apolipoprotein f precursor | 1 | | | | | | | | o |
| Apolipoprotein-L3 [Salmo salar] | 1 | o | | | | | | | |
| apoptosis antagonizing transcription factor | 1 | | | | o | | | | |
| apoptosis regulator bax | 1 | | o | | | | | | |
| apoptosis-inducing factor 2 | 1 | | o | | | | | | |
| apoptosis-inducing mitochondrion- 3 | 1 | | | | | | | | o |
| apoptosis-inducing mitochondrion-associated 1 | 1 | | | o | | | | | |
| aprataxin | 1 | o | | | | | | | |
| aprataxin-like isoform 1 | 1 | | | | o | | | | |
| aquaporin 11 | 1 | | o | | | | | | |
| aquaporin 7 | 1 | | o | | | | | | |
| arachidonate lipoxygenase 3 | 1 | | | o | | | | | |
| arfaptin-1 isoform 2 | 1 | | | | | o | | | |
| arfaptin-1-like isoform 2 | 1 | | | | o | | | | |
| arfaptin-2-like isoform 2 | 1 | | | | | | | | o |
| arf-gap domain and fg repeats-containing protein 1-like | 1 | o | | | | | | | |
| arf-gap with coiled- ank repeat and ph domain-containing protein 2 | 1 | | o | | | | | | |
| arf-gap with rho-gap ank repeat and ph domain-containing protein 1-like | 1 | o | | | | | | | |
| arginine glutamic acid dipeptide repeats | 1 | o | | | | | | | |
| arginine methyltransferase 3-like | 1 | o | | | | | | | |
| arginine vasopressin-induced protein 1 | 1 | | o | | | | | | |
| arginine-glutamic acid dipeptide repeats | 1 | | | | | | | o | |
| arkadia-like 1 isoform 1 | 1 | o | | | | | | | |
| arl13b protein | 1 | o | | | | | | | |
| armadillo repeat containing 3 | 1 | | | | | | o | | |
| armadillo repeat gene deletes in velo-cardio-facial syndrome | 1 | o | | | | | | | |
| armadillo repeat protein deleted in velo-cardio-facial syndrome | 1 | | | | | | o | | |
| arp1 actin-related protein 1 homolog b | 1 | | | | | | o | | |
| arp6 actin-related protein 6 homolog | 1 | o | | | | | | | |
| arrestin domain containing 2 | 1 | | o | | | | | | |
| arrestin domain-containing protein 2 | 1 | | | | | | o | | |
| aryl hydrocarbon receptor 2b | 1 | o | | | | | | | |

| | | | | | | | | | |
|---|---|---|---|---|---|---|---|---|---|
| aryl hydrocarbon receptor nuclear translocator 2 | 1 | | | | | | o | | |
| arylacetamide deacetylase | 1 | o | | | | | | | |
| arylformamidase | 1 | | | | | o | | | |
| arylsulfatase k precursor | 1 | o | | | | | | | |
| asialoglycoprotein receptor isoform cra_b | 1 | | o | | | | | | |
| asparagine-linked glycosylation 13 homolog ( cerevisiae) | 1 | | | | | o | | | |
| asparaginyl-trna synthetase | 1 | | o | | | | | | |
| aspartate mitochondrial | 1 | | o | | | | | | |
| aspartate-beta-hydroxylase | 1 | | o | | | | | | |
| aspartic retroviral-like 1 | 1 | o | | | | | | | |
| aspartoacylase 2 | 1 | | | | | | | | o |
| aspartyl aminopeptidase | 1 | | | | o | | | | |
| aspartyl asparaginyl beta-hydroxylase | 1 | o | | | | | | | |
| aspartyl-trna mitochondrial | 1 | | | | o | | | | |
| aspartyl-trna synthetase 2 | 1 | | o | | | | | | |
| asph protein | 1 | | | | | | o | | |
| asporin precursor | 1 | o | | | | | | | |
| astacin like metalloprotease | 1 | | o | | | | | | |
| astrocytic phosphoprotein pea-15-like | 1 | o | | | | | | | |
| astrotactin 2 | 1 | | | | | | o | | |
| at dna binding containing 1 | 1 | | | | | | o | | |
| at hook motif family protein | 1 | o | | | | | | | |
| ataxin 3 variant ref | 1 | | | | | | | | o |
| ataxin-1-like | 1 | o | | | | | | | |
| ataxin-2-like protein | 1 | | o | | | | | | |
| ataxin-7-like protein 3-like isoform 2 | 1 | o | | | | | | | |
| atlastin 3 | 1 | o | | | | | | | |
| atp dependent rna | 1 | | | | | | o | | |
| atp gtp binding 5 | 1 | | | | | | | o | |
| atp h+ mitochondrial f0 subunit d | 1 | | | | | | | o | |
| atp h+ mitochondrial f1 gamma polypeptide 1 | 1 | | | | o | | | | |
| atp synthase subunit b | 1 | | o | | | | | | |
| atp5a1 protein | 1 | | | | | | | | o |
| atpase aaa domain containing 1 | 1 | | o | | | | | | |
| atpase family aaa domain-containing protein 2 | 1 | | o | | | | | | |
| atpase family aaa domain-containing protein 3 | 1 | | | | | | | | o |
| atpase h+ transporting v0 subunit | 1 | | | | | | o | | |
| atpase type 13a3 | 1 | o | | | | | | | |
| atp-binding cassette sub-family a member 1 | 1 | | | | | | o | | |
| atp-binding cassette sub-family b member 9 | 1 | | | | | | | | o |
| atp-binding cassette sub-family d member 3 | 1 | | o | | | | | | |
| atp-binding cassette sub-family g member 5 | 1 | | o | | | | | | |
| atp-binding sub-family a member 3 | 1 | | | | | o | | | |
| atp-binding sub-family b (mdr tap) member 10 | 1 | | o | | | | | | |
| atp-binding sub-family b (mdr tap) member 3 like 1 | 1 | o | | | | | | | |
| atp-binding sub-family c (cftr mrp) member 3 | 1 | | | | | | | o | |
| atp-binding sub-family c (cftr mrp) member 4 | 1 | o | | | | | | | |
| atp-binding sub-family d member 1 | 1 | | o | | | | | | |
| atp-dependent chaperone protein | 1 | | | o | | | | | |
| atp-dependent clp protease atp-binding subunit clpx- mitochondrial | 1 | | | | | | | | o |
| atp-dependent metalloprotease yme1l isoform 2 | 1 | | | | | o | | | |
| atp-dependent metalloprotease yme1l1-like | 1 | o | | | | | | | |
| atp-dependent rna helicase | 1 | o | | | | | | | |
| atp-dependent rna helicase ddx18 | 1 | | | | | | | | o |

| | | | | | | | | | |
|---|---|---|---|---|---|---|---|---|---|
| atp-dependent rna helicase ddx19b-like | 1 | | | | o | | | | |
| atp-dependent rna helicase ddx1-like | 1 | | | | o | | | | |
| atp-dependent rna helicase ddx24-like | 1 | | o | | | | | | |
| atp-dependent rna helicase ddx3x | 1 | | o | | | | | | |
| atp-dependent zinc metalloprotease yme1l1 | 1 | | | | o | | | | |
| atp-sensitive inward rectifier potassium channel 10-like | 1 | | | | | | | | o |
| atrial natriuretic peptide-converting enzyme | 1 | | | | | | o | | |
| at-rich interactive domain-containing protein 3a | 1 | | | | o | | | | |
| at-rich interactive domain-containing protein 4b-like | 1 | | o | | | | | | |
| atr-interacting protein | 1 | | | | o | | | | |
| atrophin 1 | 1 | | | | | | o | | |
| atrophin-1 | 1 | | | | | | | | o |
| attractin | 1 | | | | | | | o | |
| attractin-like 1 | 1 | o | | | | | | | |
| autophagy protein 9 | 1 | | | | | | | | o |
| autophagy-related protein 2 homolog b-like | 1 | | o | | | | | | |
| autophagy-related protein 9a | 1 | | | | | | o | | |
| autosomal recessive 1a | 1 | o | | | | | | | |
| axonemal dynein light chain domain-containing protein 1 | 1 | | | | o | | | | |
| b cell rag associated protein | 1 | o | | | | | | | |
| b chain alternative splicing of rac1 generates a self- activating gtpase | 1 | | | | | | o | | |
| b chain crystal structure of the ectodomain complex of the cgrp a class-b reveals the site of drug antagonism | 1 | o | | | | | | | |
| b chain histone h3 recognition and presentation by the wdr5 module of the mll1 complex | 1 | | | | o | | | | |
| b chain snapshots of the rna processing factor scaf8 bound to different phosphorylated forms of the carboxy-terminal domain of rna-polymerase ii | 1 | o | | | | | | | |
| b double prime subunit of rna polymerase iii transcription initiation factor iiib | 1 | | | | | | | o | |
| bactericidal permeability-increasing protein lipopolysaccharide-binding protein | 1 | o | | | | | | | |
| baculoviral iap repeat-containing 5 | 1 | | | | o | | | | |
| baculoviral iap repeat-containing isoform cra_a | 1 | o | | | | | | | |
| baculoviral iap repeat-containing protein 4 | 1 | o | | | | | | | |
| baculoviral iap repeat-containing protein 5-partial | 1 | | | | o | | | | |
| baculoviral iap repeat-containing protein 6-like | 1 | | | | | | o | | |
| bag family molecular chaperone regulator 2 | 1 | | o | | | | | | |
| band -like protein 3 ( ) (differentially expressed in adenocarcinoma of the lung protein 1) (dal-1) | 1 | | | | | | | o | |
| bardet-biedl syndrome 1 | 1 | | o | | | | | | |
| bardet-biedl syndrome 5 | 1 | o | | | | | | | |
| bardet-biedl syndrome 5 protein homolog | 1 | | | | | | | | o |
| bardet-biedl syndrome 7 | 1 | o | | | | | | | |
| basement membrane-specific heparan sulfate proteoglycan core protein | 1 | | | | | | o | | |
| basic helix-loop-helix domain-containing protein kiaa2018 | 1 | | | | | | | | o |

| | | | | | | | | | |
|---|---|---|---|---|---|---|---|---|---|
| basic helix-loop-helix domain-containing protein kiaa2018-like | 1 | | | | | | | | o |
| basic leucine zipper and w2 domain-containing protein 1 | 1 | | | | | | | | o |
| basic leucine zipper nuclear factor 1 | 1 | o | | | | | | | |
| basonuclin 2 | 1 | | | | | | o | | |
| bbsome-interacting protein 1-like | 1 | | | | | | | | o |
| bc002230 protein | 1 | o | | | | | | | |
| b-cell antigen receptor complex-associated protein alpha chain | 1 | o | | | | | | | |
| b-cell antigen receptor complex-associated protein beta chain-like | 1 | o | | | | | | | |
| b-cell cll lymphoma 2 | 1 | o | | | | | | | |
| b-cell cll lymphoma 6 | 1 | o | | | | | | | |
| b-cell cll lymphoma member b | 1 | o | | | | | | | |
| b-cell leukemia lymphoma 2 | 1 | o | | | | | | | |
| b-cell lymphoma 6 protein isoform 2 | 1 | o | | | | | | | |
| bchain crystal structure of an integrin beta3-talin chimera | 1 | | | | | | o | | |
| bcl10-interacting card protein | 1 | o | | | | | | | |
| bcl2 adenovirus e1b 19 kda protein-interacting protein 2-like | 1 | | o | | | | | | |
| bcl2 adenovirus e1b 19kda interacting protein 1 | 1 | | o | | | | | | |
| bcl2 adenovirus e1b 19kda interacting protein 3-like | 1 | | | | | | o | | |
| bcl2-antagonist of cell death | 1 | | | o | | | | | |
| bcl2-associated athanogene | 1 | o | | | | | | | |
| bcl2-associated athanogene 2 | 1 | o | | | | | | | |
| bcl2-associated athanogene 3 | 1 | o | | | | | | | |
| bcl2-like 12 (proline rich) | 1 | | o | | | | | | |
| bcl2-like 13 (apoptosis facilitator) | 1 | | o | | | | | | |
| bcl2-related ovarian killer | 1 | | | | | | | | o |
| bcl6 co-repressor | 1 | | | | | | o | | |
| bcl-6 interacting corepressor-like | 1 | o | | | | | | | |
| bel12_ag transposon polyprotein | 1 | | | | | | o | | |
| benzodiazapine receptor associated protein 1 | 1 | o | | | | | | | |
| bestrophin 3 | 1 | o | | | | | | | |
| bet1-like protein | 1 | o | | | | | | | |
| beta 1 (fibronectin beta antigen cd29 includes msk12) isoform cra_a | 1 | o | | | | | | | |
| beta 2 (laminin s) | 1 | o | | | | | | | |
| beta fibrinogen | 1 | | | | | | | | o |
| beta-galactosyl-o-glycosyl-glycoprotein beta- -n-acetylglucosaminyltransferase 7 | 1 | | o | | | | | | |
| beta-galactosyltransferase 3 | 1 | | o | | | | | | |
| beta-galactosyltransferase 6 | 1 | | | | | | o | | |
| beta-galactosyltransferase-like | 1 | o | | | | | | | |
| beta-n-acetyl-galactosaminyl transferase 1 | 1 | | | | | | o | | |
| beta-n-acetyl-galactosaminyl transferase 2 | 1 | o | | | | | | | |
| beta-n-acetylgalactosaminyltransferase 1 | 1 | o | | | | | | | |
| beta-n-acetylgalactosaminyltransferase 2 | 1 | | o | | | | | | |
| beta subunit of rna polymerase | 1 | | | | | | | o | |
| beta-2-glycoprotein 1 | 1 | | o | | | | | | |
| beta-galactosamide alpha- -sialyltransferase ii | 1 | | | o | | | | | |
| beta-galactoside alpha -sialyltransferase st6gal ii | 1 | | | o | | | | | |

| | | | | | | | | | |
|---|---|---|---|---|---|---|---|---|---|
| beta-hexosaminidase beta chain precursor (n-acetyl-beta-glucosaminidase) (beta-n-acetylhexosaminidase) (hexosaminidase b) (cervical cancer proto-oncogene 7) (hcc-7) | 1 | | | o | | | | | |
| beta-hexosaminidase subunit beta | 1 | | | | o | | | | |
| beta-ketoacyl synthase | 1 | | o | | | | | | |
| beta-lactamase domain protein | 1 | | | o | | | | | |
| beta-lactamase-like protein | 1 | o | | | | | | | |
| beta-soluble nsf attachment | 1 | | | | | | | | o |
| Beta-synuclein [Heterocephalus glaber] | 1 | | | | | | | | o |
| beta-tubulin | 1 | | | | | | | | o |
| beta-tubulin at isoform a | 1 | | | | | | | o | |
| bhlh protein dec1a | 1 | o | | | | | | | |
| bhlh protein dec1b | 1 | o | | | | | | | |
| bicaudal c homolog 1 | 1 | | | | | o | | | |
| bicaudal d homolog 2 | 1 | | | | | o | | | |
| bifunctional atp-dependent dihydroxyacetone kinase fad-amp lyase | 1 | | o | | | | | | |
| bifunctional methylenetetrahydrofolate dehydrogenase mitochondrial-like | 1 | | | | o | | | | |
| bifunctional protein ncoat | 1 | o | | | | | | | |
| biglycan | 1 | | | | | | | | o |
| biglycan preproprotein variant | 1 | | o | | | | | | |
| biogenesis of lysosome-related organelles complex 1 subunit 3 | 1 | | | | | | | o | |
| biogenesis of lysosome-related organelles complex-1 subunit 2 | 1 | o | | | | | | | |
| biorientation of chromosomes in cell division protein 1 | 1 | | | | | | | | o |
| bis(5 -nucleosyl)-tetraphosphatase | 1 | | | o | | | | | |
| -bisphosphate nucleotidase 1 | 1 | | | | o | | | | |
| bladder cancer-associated protein | 1 | o | | | | | | | |
| blastula protease-10 | 1 | | | | o | | | | |
| blood vessel epicardial substance | 1 | o | | | | | | | |
| bloodthirsty | 1 | | | | | | | o | |
| bmp and activin membrane-bound inhibitor homolog | 1 | | o | | | | | | |
| bmp-2-inducible protein kinase isoform 1 | 1 | o | | | | | | | |
| bnr repeat domain protein | 1 | | | o | | | | | |
| bola-like protein 3 | 1 | | o | | | | | | |
| bone morphogenetic protein 1 precursor | 1 | | | | o | | | | |
| bone morphogenetic protein 7 | 1 | | o | | | | | | |
| bone specific cmf608 | 1 | o | | | | | | | |
| borealin | 1 | | | | o | | | | |
| br serine threonine kinase isoform cra_c | 1 | o | | | | | | | |
| brachyury and tbx related protein | 1 | | | | o | | | | |
| brain and acute cytoplasmic | 1 | | | | | | | o | |
| brain and muscle arnt-like 1 protein | 1 | | | o | | | | | |
| brain protein 16 | 1 | o | | | | | | | |
| brain protein i3 | 1 | | o | | | | | | |
| brain specific kinase 146 | 1 | o | | | | | | | |
| brain-enriched guanylate kinase-associated | 1 | | | | | | | | o |
| brain-enriched guanylate kinase-associated protein | 1 | | | | | | o | | |
| brain-specific angiogenesis inhibitor 1-associated protein 2 | 1 | | | | | | | o | |
| branched chain aminotransferase cytosolic | 1 | | o | | | | | | |
| branched-chain-amino-acid cytosolic | 1 | | o | | | | | | |
| brct domain protein | 1 | o | | | | | | | |
| breakpoint cluster region | 1 | | o | | | | | | |

| | | | | | | | | | |
|---|---|---|---|---|---|---|---|---|---|
| breakpoint cluster region isoform 1 | 1 | | | | | | o | | |
| breast cancer antiestrogen resistance 3 | 1 | | o | | | | | | |
| breast cancer anti-estrogen resistance protein 1 | 1 | | | | | | | | o |
| breast carcinoma amplified sequence 3 | 1 | o | | | | | | | |
| brefeldin a-inhibited guanine nucleotide-exchange protein 1-like | 1 | o | | | | | | | |
| brefeldin a-inhibited guanine nucleotide-exchange protein 3 | 1 | | | | | | o | | |
| brevican core | 1 | | | | | | | | o |
| brevican isoform 1 | 1 | | | | | | o | | |
| bridging integrator 2 | 1 | | | | o | | | | |
| brisc complex subunit abro1 | 1 | o | | | | | | | |
| bro1 domain-containing protein brox | 1 | | | | | | o | | |
| bromo adjacent homology domain-containing 1 | 1 | | | | | | o | | |
| bromodomain adjacent to zinc finger 1a | 1 | o | | | | | | | |
| bromodomain adjacent to zinc finger domain protein 2a | 1 | | | | | | o | | |
| bromodomain and phd finger 1 | 1 | o | | | | | | | |
| bromodomain and wd repeat domain containing 2 | 1 | o | | | | | | | |
| bromodomain and wd repeat-containing protein 1 | 1 | | | | | | o | | |
| bromodomain containing 8 isoform 1 | 1 | o | | | | | | | |
| bromodomain phd finger transcription factor | 1 | o | | | | | | | |
| bromodomain-containing protein | 1 | | | | o | | | | |
| bromodomain-containing protein 7 | 1 | | | | o | | | | |
| bromodomain-containing protein 8 | 1 | | | | | o | | | |
| bruno-3 transcript variant 4 | 1 | | | | | | | o | |
| bruno-like 4 protein | 1 | | | | | | | o | |
| bruno-like rna binding protein | 1 | | | | | | | o | |
| bruton agammaglobulinemia tyrosine kinase | 1 | o | | | | | | | |
| btb and cnc homology basic leucine zipper transcription factor 1 | 1 | o | | | | | | | |
| btb and cnc homology basic leucine zipper transcription factor 2 | 1 | o | | | | | | | |
| btb domain containing 14a | 1 | | | | | | o | | |
| btb domain containing 3 | 1 | | | | | | | | o |
| btb poz domain-containing protein 17-like | 1 | | | | | | o | | |
| btb poz domain-containing protein kctd4-like | 1 | | | | | | | | o |
| btb poz domain-containing protein kctd9-like | 1 | | | | | | | | o |
| bub3 budding uninhibited by benzimidazoles 3 homolog | 1 | o | | | | | | | |
| bud13 homolog | 1 | o | | | | | | | |
| budding uninhibited by benzimidazoles 3 homolog | 1 | o | | | | | | | |
| butyrobetaine 2-oxoglutarate dioxygenase (gamma-butyrobetaine hydroxylase) 1 | 1 | | | o | | | | | |
| butyrophilin subfamily 1 member a1 precursor | 1 | o | | | | | | | |
| butyrophilin-like protein 2-like | 1 | | o | | | | | | |
| butyrophilin-like protein 8-like | 1 | | | | | | | | o |
| c alpha -sialyltransferase | 1 | o | | | | | | | |
| c14orf179 homolog | 1 | o | | | | | | | |
| C19orf52 [Anoplopoma fimbria] | 1 | | | o | | | | | |
| c1orf131 homolog | 1 | o | | | | | | | |
| c1orf187 precursor | 1 | | | o | | | | | |
| c1orf43 homolog | 1 | | o | | | | | | |
| c1orf93 homolog | 1 | | o | | | | | | |

| | | | | | | | | | |
|---|---|---|---|---|---|---|---|---|---|
| c1q and tumor necrosis factor related protein 6 | 1 | o | | | | | | | |
| c2 domain-containing protein 2-like | 1 | | o | | | | | | |
| c2h2 finger domain | 1 | o | | | | | | | |
| c2h2 finger domain-containing protein | 1 | | | | | | o | | |
| c2h2 transcription factor | 1 | | | | | | | | o |
| c2orf25 mitochondrial precursor | 1 | | o | | | | | | |
| c2orf29 protein | 1 | o | | | | | | | |
| c3 and pzp- alpha-2-macroglobulin domain containing 8 | 1 | | o | | | | | | |
| c3orf54 homolog | 1 | o | | | | | | | |
| c4b-binding protein alpha chain precursor | 1 | | | | o | | | | |
| c4orf8 protein | 1 | o | | | | | | | |
| c5orf4 protein | 1 | | o | | | | | | |
| c6orf89 homolog | 1 | o | | | | | | | |
| c7orf10 protein | 1 | | o | | | | | | |
| c9orf123 homolog | 1 | | | | o | | | | |
| c9orf75 protein | 1 | | | | | | o | | |
| c9orf85 homolog | 1 | | o | | | | | | |
| ca++ cardiac fast twitch isoform cra_b | 1 | o | | | | | | | |
| ca++ cardiac slow twitch isoform cra_b | 1 | | o | | | | | | |
| ca++ cardiac slow twitch isoform cra_d | 1 | | o | | | | | | |
| ca++ plasma membrane 2 | 1 | | | | | | o | | |
| ca++ plasma membrane 3-like isoform 1 | 1 | | | | | | | o | |
| ca++ plasma membrane 4 | 1 | | | | | | | o | |
| ca++ ubiquitous | 1 | | | | | | o | | |
| cad protein | 1 | | | | o | | | | |
| cadherin 15 | 1 | o | | | | | | | |
| cadherin 22-like | 1 | o | | | | | | | |
| cadherin 4 | 1 | | | | | | o | | |
| cadherin egf lag seven-pass g-type receptor 3 | 1 | | | | | | o | | |
| cadherin h-cadherin | 1 | | | | | | o | | |
| cadherin osteoblast | 1 | | | | | | o | | |
| cadherin protein | 1 | | | | | | o | | |
| cadherin type 2 | 1 | | | | | | o | | |
| cadherin type 2 prepro partial | 1 | | | | | | | o | |
| cadherin-2 precursor | 1 | | | | | | o | | |
| cadherin-like 24 | 1 | o | | | | | | | |
| cadherin-related family member 1-like | 1 | | | | | o | | | |
| cadherin-related family member 2-like | 1 | | | | | | | | o |
| cadherin-related family member 5-like | 1 | | | | | | o | | |
| cadherin-related neuronal receptor variable 11 | 1 | | | | | | | o | |
| cadherin-related neuronal receptor variable 4 | 1 | | | | | | | o | |
| calbindin 1 | 1 | | | | | | | | o |
| calcineurin binding protein isoform cra_c | 1 | | | | | | | o | |
| calcitonin gene-related peptide precursor | 1 | | | | | | | | o |
| calcitonin receptor like receptor 3 spliced isoform | 1 | o | | | | | | | |
| calcium binding protein 5 | 1 | | | | | | o | | |
| calcium calmodulin-dependent protein kinase ( kinase) ii delta | 1 | | | o | | | | | |
| calcium calmodulin-dependent protein kinase ( kinase) ii delta 2 | 1 | | | | | | o | | |
| calcium calmodulin-dependent protein kinase beta | 1 | o | | | | | | | |
| calcium calmodulin-dependent protein kinase i | 1 | o | | | | | | | |
| calcium calmodulin-dependent protein kinase id | 1 | | | | | | o | | |
| calcium calmodulin-dependent protein kinase ii delta | 1 | | o | | | | | | |

| | | | | | | | | | |
|---|---|---|---|---|---|---|---|---|---|
| calcium calmodulin-dependent protein kinase iia | 1 | | | | | | o | | |
| calcium calmodulin-dependent protein kinase kinase alpha | 1 | | | | | | o | | |
| calcium calmodulin-dependent protein kinase type ii gamma chain | 1 | | | | | | o | | |
| calcium calmodulin-dependent protein kinase type ii subunit beta-like | 1 | | | | | | o | | |
| calcium calmodulin-dependent protein kinase type ii subunit gamma | 1 | o | | | | | | | |
| calcium channel alpha-1 subunit homolog | 1 | o | | | | | | | |
| calcium channel flower homolog | 1 | o | | | | | | | |
| calcium modulating ligand | 1 | | o | | | | | | |
| calcium polyvalent cation receptor 3 | 1 | o | | | | | | | |
| calcium signal-modulating cyclophilin ligand-like | 1 | | | | | | | | o |
| calcium uptake protein mitochondrial | 1 | | | | | | | | o |
| calcium voltage- alpha 1f subunit | 1 | | | o | | | | | |
| calcium voltage- alpha 2 delta subunit 3 | 1 | | | | | | o | | |
| calcium voltage- alpha2 delta subunit partial | 1 | o | | | | | | | |
| calcium voltage- beta 1 subunit | 1 | o | | | | | | | |
| calcium voltage- beta 2 subunit | 1 | | | | | | | o | |
| calcium voltage- beta 3a | 1 | | | | | | | o | |
| calcium voltage- t alpha 1g subunit | 1 | | | | | | | o | |
| calcium voltage- t alpha 1h subunit | 1 | | | | | | | o | |
| calcium voltage- t alpha 1i subunit | 1 | | | | | | o | | |
| calcium-binding and coiled-coil domain-containing protein 2 | 1 | | o | | | | | | |
| calcium-binding mitochondrial carrier protein s -2 | 1 | | | | | | o | | |
| calcium-binding mitochondrial carrier protein s -3-like | 1 | | | | | | | | o |
| calcium-binding protein 39-like | 1 | o | | | | | | | |
| calcium-dependent secretion activator 1 | 1 | | | | | | | | o |
| calcium-independent phospholipase a2-gamma | 1 | | o | | | | | | |
| calcium-regulated heat stable protein 1 | 1 | | | | | | | | o |
| calcium-transporting atpase sarcoplasmic endoplasmic reticulum type (calcium pump) | 1 | o | | | | | | | |
| calcyclin-binding protein | 1 | | o | | | | | | |
| caldesmon 1 | 1 | o | | | | | | | |
| calmodulin 2 | 1 | | | | | | o | | |
| calmodulin 2 (phosphorylase delta) isoform cra_a | 1 | | | | | | | | o |
| calmodulin 2-like | 1 | | | | o | | | | |
| calmodulin binding protein 4 | 1 | | | | | | | o | |
| calmodulin binding transcription activator 2 | 1 | | | | | | o | | |
| calmodulin-like protein 4 | 1 | | o | | | | | | |
| calmodulin-regulated spectrin-associated protein 3-like | 1 | | o | | | | | | |
| calnexin | 1 | | | | | | o | | |
| calpactin i heavy calcium ion binding protein | 1 | | | | o | | | | |
| calpain | 1 | | o | | | | | | |
| calpain (mu i) large subunit a | 1 | | | | | | o | | |
| calpain isoform cra_a | 1 | | | | o | | | | |
| calpain-3 | 1 | o | | | | | | | |
| calpain-3-like isoform 3 | 1 | o | | | | | | | |
| calsequestrin 1 (fast- skeletal muscle) | 1 | o | | | | | | | |
| calsequestrin 2 (cardiac muscle) | 1 | | | | | o | | | |
| calsequestrin 2b | 1 | | | | | | | o | |

| | | | | | | | | | |
|---|---|---|---|---|---|---|---|---|---|
| calsyntenin 2 | 1 | | | | | | o | | |
| calumenin | 1 | o | | | | | | | |
| camk family protein kinase | 1 | o | | | | | | | |
| camp and camp-inhibited cgmp -cyclic phosphodiesterase | 1 | | | | | | o | | |
| camp responsive element binding 2 | 1 | | | | | | | | o |
| camp-dependent protein kinase catalytic subunit beta | 1 | | | | | | o | | |
| camp-responsive element modulator | 1 | o | | | | | | | |
| camp-responsive element modulator-like isoform 1 | 1 | | o | | | | | | |
| canalicular multispecific organic anion transporter 1 | 1 | | | o | | | | | |
| cancer susceptibility candidate 4 isoform 1 | 1 | o | | | | | | | |
| cancer susceptibility candidate 5 | 1 | o | | | | | | | |
| canopy homolog 2 precursor | 1 | | | | o | | | | |
| cap-gly domain containing linker protein 1 | 1 | o | | | | | | | |
| cap-gly domain-containing linker protein 1-like isoform 2 | 1 | | | | | | | o | |
| cap-gly domain-containing linker protein 2-like | 1 | | | | | | o | | |
| capping protein (actin filament) gelsolin-like | 1 | o | | | | | | | |
| cappuccino homolog | 1 | | | | | | | | o |
| capsid maturational protease | 1 | | | | | | o | | |
| carbamoyl-phosphate synthetase aspartate and dihydroorotase | 1 | o | | | | | | | |
| carbohydrate kinase domain containing | 1 | o | | | | | | | |
| carbohydrate kinase domain containing-like | 1 | o | | | | | | | |
| carbohydrate kinase domain-containing protein | 1 | | | | | o | | | |
| carbohydrate sulfotransferase 12 | 1 | o | | | | | | | |
| carbohydrate sulfotransferase 15-like | 1 | | o | | | | | | |
| carbohydrate sulfotransferase 1-like | 1 | | | | | | | | o |
| carbonic anhydrase vi | 1 | o | | | | | | | |
| carbonic anhydrase vii | 1 | | | | o | | | | |
| carbonic anhydrase viii | 1 | | | | | | | o | |
| carbonic anhydrase xii | 1 | | | | | | | o | |
| carboxyl transferase | 1 | | o | | | | | | |
| carboxylesterase 1 (monocyte macrophage serine esterase 1) | 1 | | o | | | | | | |
| carboxylesterase 7 | 1 | | o | | | | | | |
| carboxypeptidase b2 precursor | 1 | | | | o | | | | |
| carboxypeptidase e precursor | 1 | | | | | | | | o |
| carboxypeptidase h | 1 | | | | | | o | | |
| carboxypeptidase n catalytic chain precursor | 1 | | | | o | | | | |
| carboxypeptidase o | 1 | o | | | | | | | |
| carboxypeptidase x 2 (m14 family) | 1 | o | | | | | | | |
| carboxypeptidase z-like | 1 | | | | | | | | o |
| cardiac calsequestrin | 1 | | | | | o | | | |
| cardiac troponin t | 1 | o | | | | | | | |
| carnitine o-acetyltransferase-like | 1 | | | | o | | | | |
| carnitine o-palmitoyltransferase liver isoform-like | 1 | | | | o | | | | |
| carnitine palmitoyltransferase i | 1 | | | o | | | | | |
| carnosine dipeptidase 1 (metallopeptidase m20 family) | 1 | | | | | | | o | |
| cartilage acidic protein 1 | 1 | o | | | | | | | |
| cartilage-associated protein precursor | 1 | | | | o | | | | |
| cas scaffolding protein family member 4 | 1 | o | | | | | | | |
| cas1 domain-containing protein 1-like | 1 | | | | | o | | | |

| | | | | | | | | | |
|---|---|---|---|---|---|---|---|---|---|
| cas-br-m ecotropic retroviral transforming sequence b | 1 | o | | | | | | | |
| cas-br-m ecotropic retroviral transforming sequence b isoform 1 | 1 | | | | | | o | | |
| casein kinase 1 epsilon | 1 | | | | | | o | | |
| casein kinase 1 gamma 2a | 1 | | o | | | | | | |
| casein kinase 2 alpha 1 | 1 | | | o | | | | | |
| cask interacting protein 1 | 1 | | | | | | o | | |
| caskin1 protein | 1 | o | | | | | | | |
| caspase 9 | 1 | o | | | | | | | |
| caspase a | 1 | o | | | | | | | |
| caspase recruitment domain-containing protein 8-like | 1 | | | | | | o | | |
| caspase xa | 1 | o | | | | | | | |
| caspase-1 [Sparus aurata] | 1 | | o | | | | | | |
| caspase-1 precursor | 1 | | | o | | | | | |
| cat eye syndrome chromosome candidate 1 | 1 | o | | | | | | | |
| cat eye syndrome chromosome candidate 5 | 1 | | o | | | | | | |
| cat eye syndrome critical region protein 2 | 1 | | | | | | o | | |
| catenin (cadherin-associated protein) alpha 102kda | 1 | | | | | | | o | |
| catenin alpha-2 | 1 | | | | | | | | o |
| cathepsin e | 1 | | | o | | | | | |
| cathepsin k precursor | 1 | | | | o | | | | |
| cathepsin l1 precursor | 1 | | | | | | | | o |
| cation diffusion facilitator family transporter containing protein | 1 | | | | | | o | | |
| cation h+ exchanger protein 1 | 1 | | | o | | | | | |
| catna1 protein | 1 | | o | | | | | | |
| caveolin 1 | 1 | o | | | | | | | |
| caveolin 3 | 1 | o | | | | | | | |
| cbf1 interacting corepressor cir | 1 | | | | | | o | | |
| cbsa protein | 1 | | o | | | | | | |
| cc chemokine ck- | 1 | o | | | | | | | |
| cc chemokine receptor 5 | 1 | o | | | | | | | |
| c-c chemokine receptor type 7-like | 1 | | | | o | | | | |
| c-c motif chemokine 19 precursor | 1 | | | o | | | | | |
| cc078_mouse ame: full=upf0640 protein c3orf78 homolog ame: full=small nucleolar rna host gene 8 | 1 | | | | | o | | | |
| ccaat enhancer-binding protein zeta | 1 | | o | | | | | | |
| ccch zinc finger domain protein | 1 | o | | | | | | | |
| ccctc-binding factor (zinc finger protein)-like | 1 | | o | | | | | | |
| ccnl1 protein | 1 | o | | | | | | | |
| ccr4 carbon catabolite repression 4-like | 1 | o | | | | | | | |
| ccr4 carbon catabolite repression 4-like ( cerevisiae) | 1 | | o | | | | | | |
| ccr4-not transcription complex subunit 8 | 1 | | | o | | | | | |
| ccr4-not transcription subunit 4 | 1 | | o | | | | | | |
| cd164 sialomucin | 1 | | o | | | | | | |
| cd200 molecule | 1 | o | | | | | | | |
| cd200 receptor 1 | 1 | o | | | | | | | |
| cd209 molecule | 1 | | | o | | | | | |
| cd276 antigen-like | 1 | o | | | | | | | |
| cd276 molecule | 1 | o | | | | | | | |
| cd2-associated protein | 1 | o | | | | | | | |
| cd3 epsilon | 1 | o | | | | | | | |
| cd3 zeta | 1 | o | | | | | | | |
| cd40 antigen | 1 | | | | | | | o | |
| cd4-2 protein | 1 | o | | | | | | | |

| | | | | | | | | | |
|---|---|---|---|---|---|---|---|---|---|
| cd48 antigen precursor | 1 | o | | | | | | | |
| CD63 antigen, putative [Pediculus humanus corporis] | 1 | | | | | o | | | |
| cd68 molecule | 1 | o | | | | | | | |
| cd80 86 molecule short secretory form | 1 | | | | o | | | | |
| cd82 antigen-like | 1 | | | | | | | | o |
| cd97 antigen precursor | 1 | o | | | | | | | |
| cd97 antigen-like | 1 | o | | | | | | | |
| cd99 antigen-like protein 2-like | 1 | | | | | | o | | |
| cd99 molecule-like 2 | 1 | | | | | | | o | |
| cdc2l1 protein | 1 | | | | o | | | | |
| cdc42 binding protein kinase alpha (dmpk-like) | 1 | o | | | | | | | |
| cdc42 effector protein (rho gtpase binding) 1 | 1 | o | | | | | | | |
| cdc42 effector protein 3 | 1 | o | | | | | | | |
| cdc42 gtpase-activating protein | 1 | o | | | | | | | |
| cdc42 small effector 2 | 1 | | o | | | | | | |
| cdc42 small effector protein 2-like | 1 | | | | o | | | | |
| cdc42-interacting protein 4 | 1 | | o | | | | | | |
| cdc-like kinase 1 | 1 | o | | | | | | | |
| cdgsh iron sulfur domain 1 | 1 | | | | o | | | | |
| cdgsh iron sulfur domain-containing protein 2 | 1 | o | | | | | | | |
| cdk5 regulatory subunit associated protein 1-like 1 | 1 | o | | | | | | | |
| cdk5 regulatory subunit associated protein 2 | 1 | o | | | | | | | |
| cdk-activating kinase assembly factor mat1 | 1 | o | | | | | | | |
| cdkn2a interacting protein | 1 | | | o | | | | | |
| cdkn2a-interacting protein | 1 | o | | | | | | | |
| cdkn2aip n-terminal-like protein | 1 | o | | | | | | | |
| cdna sequence bc052484-like | 1 | o | | | | | | | |
| cdna sequence isoform cra_a | 1 | | | | | | | o | |
| cdp-diacylglycerol--glycerol-3-phosphate 3-mitochondrial | 1 | | o | | | | | | |
| cell adhesion molecule 1 isoform 2 | 1 | | | | | | | o | |
| cell adhesion molecule jcam | 1 | | o | | | | | | |
| cell adhesion molecule with homology to l1cam | 1 | | | | | | | o | |
| cell cycle checkpoint protein rad17 | 1 | | | | | | | | o |
| cell cycle control protein 50c | 1 | | o | | | | | | |
| cell cycle progression protein 1 | 1 | | | o | | | | | |
| cell death-inducing dffa-like effector c | 1 | | | | o | | | | |
| cell division control protein 42 homolog | 1 | | | | | | o | | |
| cell division cycle 25a | 1 | o | | | | | | | |
| cell division cycle and apoptosis regulator protein 1 | 1 | | | | | | o | | |
| cell division cycle and apoptosis regulator protein 1-like | 1 | | o | | | | | | |
| cell division cycle associated 4 | 1 | o | | | | | | | |
| cell division cycle-associated protein 7 | 1 | | | | o | | | | |
| cell division protein | 1 | | | | | | o | | |
| cell division protein kinase 17 | 1 | | | | | | | o | |
| cell division protein kinase 9 | 1 | o | | | | | | | |
| cell growth regulator with ef-hand domain 1 | 1 | o | | | | | | | |
| cell surface fibronectin-binding protein | 1 | | | | | | | o | |
| cell surface protein | 1 | | | | o | | | | |
| cell wall adhesin | 1 | o | | | | | | | |
| cell wall-associated hydrolase | 1 | | | | o | | | | |
| cellular retinoic acid binding protein 2 | 1 | o | | | | | | | |
| centrin-1 | 1 | o | | | | | | | |
| centromere protein 312kda | 1 | o | | | | | | | |

| | | | | | | | | | |
|---|---|---|---|---|---|---|---|---|---|
| centromere protein c 1 | 1 | o | | | | | | | |
| centromere protein e | 1 | o | | | | | | | |
| centromere protein j | 1 | | | | | | | | o |
| centromere protein o | 1 | o | | | | | | | |
| centromere protein q-like | 1 | | | | o | | | | |
| centromere protein t | 1 | | | | o | | | | |
| centromere protein x | 1 | o | | | | | | | |
| centromere protein x-like | 1 | | | | | | o | | |
| centrosomal protein of 44 kda | 1 | | | | o | | | | |
| centrosomal protein poc5 | 1 | | | | o | | | | |
| centrosome and spindle pole associated protein 1 | 1 | | o | | | | | | |
| ceramide glucosyltransferase | 1 | | o | | | | | | |
| cerebellar degeneration-related protein 2 | 1 | | | | o | | | | |
| cerebellin-2 precursor | 1 | | | | | | | | o |
| cerebellin-2-like | 1 | | | | | | | | o |
| ceroid- neuronal 5 | 1 | o | | | | | | | |
| ceroid- neuronal isoform cra_a | 1 | o | | | | | | | |
| ceroid-lipofuscinosis neuronal protein 5-like | 1 | | | | | | | | o |
| ceruloplasmin [Chionodraco rastrospinosus] | 1 | | | o | | | | | |
| ceruloplasmin precursor | 1 | | | | | o | | | |
| cf191 protein | 1 | | | | | o | | | |
| cg31439 cg31439- partial | 1 | o | | | | | | | |
| cg33099 cg33099-pa | 1 | | | o | | | | | |
| cg41389 cg41389- partial | 1 | o | | | | | | | |
| cgmp-dependent protein kinase 1 | 1 | o | | | | | | | |
| cgmp-inhibited 3 -cyclic phosphodiesterase a | 1 | | | | | | o | | |
| cgmp-stimulated cyclic nucleotide phosphodiesterase | 1 | | | | | | o | | |
| ch076 protein | 1 | o | | | | | | | |
| chain a six finger zinc finger designed to recognize ann partial | 1 | | | | | o | | | |
| chaperonin containing tcp-1 delta | 1 | o | | | | | | | |
| chaperonin subunit 6a zeta | 1 | | | | | o | | | |
| charged multivesicular body protein 2b | 1 | | o | | | | | | |
| chd9 protein | 1 | o | | | | | | | |
| chemokine (c-c motif) ligand 19 | 1 | | | | | o | | | |
| chemokine (c-c motif) ligand 22 | 1 | | | | | o | | | |
| chemokine (c-c motif) ligand 3-like 1 | 1 | o | | | | | | | |
| chemokine (c-c motif) ligand 4 | 1 | o | | | | | | | |
| chemokine (c-x-c motif) ligand 14 | 1 | o | | | | | | | |
| chemokine (c-x-c motif) receptor 4 | 1 | | | | o | | | | |
| chemokine orphan receptor isoform cra_a | 1 | o | | | | | | | |
| chemokine receptor-like 1-like | 1 | o | | | | | | | |
| chemokine-like factor | 1 | | | | | | | | o |
| chibby homolog 1 | 1 | | | | | | o | | |
| chicken ovalbumin upstream promoter-transcription factor i | 1 | | | | | | o | | |
| chitinase domain containing 1 | 1 | o | | | | | | | |
| chitinase domain-containing protein 1 precursor | 1 | | | o | | | | | |
| chk1 checkpoint homolog ( pombe) | 1 | | | | | | | o | |
| chloramphenicol acetyltransferase | 1 | | o | | | | | | |
| chloride channel 2 | 1 | | | | | | o | | |
| chloride channel 3 isoform 1 | 1 | o | | | | | | | |
| chloride channel protein 2 | 1 | | | | | | o | | |
| chloride channel skeletal muscle | 1 | o | | | | | | | |
| chmadrin (short type) | 1 | o | | | | | | | |
| chn1 protein | 1 | | | | | | | o | |
| choline ethanolamine kinase | 1 | | | | | | | | o |

| | | | | | | | | | |
|---|---|---|---|---|---|---|---|---|---|
| choline kinase alpha-like | 1 | | | | | | | | o |
| choline o-acetyltransferase isoform 1 | 1 | o | | | | | | | |
| choline-phosphate cytidylyltransferase b | 1 | | o | | | | | | |
| cholinephosphotransferase 1 | 1 | o | | | | | | | |
| cholinergic muscarinic 2 | 1 | | | | | | o | | |
| chondroitin sulfate n-acetylgalactosaminyltransferase 1-like | 1 | o | | | | | | | |
| chondroitin sulfate proteoglycan 2 isoform cra_c | 1 | o | | | | | | | |
| chondroitin sulfate proteoglycan 5 (neuroglycan c) | 1 | | | | | | | o | |
| chorion protein | 1 | | | o | | | | | |
| choroideremia (rab escort protein 1) | 1 | o | | | | | | | |
| chromatin accessibility complex protein 1 | 1 | | o | | | | | | |
| chromatin assembly factor subunit a | 1 | o | | | | | | | |
| chromatin modifying protein 6 | 1 | | | | | o | | | |
| chromobox homolog 4 | 1 | o | | | | | | | |
| chromobox homolog 5 (hp1 alpha drosophila) | 1 | | o | | | | | | |
| chromobox homolog 8 | 1 | | | | | o | | | |
| chromobox homolog 8 (pc class drosophila) | 1 | o | | | | | | | |
| chromobox protein homolog 2 | 1 | | | | | | | | o |
| chromobox protein homolog 8 | 1 | o | | | | | | | |
| chromobox protein homolog 8-like | 1 | | | | | | | | o |
| chromodomain helicase dna binding protein 1 | 1 | o | | | | | | | |
| chromodomain helicase dna binding protein isoform cra_a | 1 | o | | | | | | | |
| chromodomain y-like | 1 | | o | | | | | | |
| chromodomain y-like 2 | 1 | o | | | | | | | |
| chromodomain-helicase-dna-binding protein 1-like | 1 | o | | | | | | | |
| chromodomain-helicase-dna-binding protein 2 | 1 | o | | | | | | | |
| chromogranin a | 1 | | | | | o | | | |
| chromosomal protein d1 | 1 | o | | | | | | | |
| chromosome 1 open reading frame 115 | 1 | o | | | | | | | |
| chromosome 1 open reading frame 131 | 1 | | | o | | | | | |
| chromosome 1 open reading frame 194 | 1 | | | | | o | | | |
| chromosome 1 open reading frame 2 | 1 | | | | | | o | | |
| chromosome 1 open reading frame 31 | 1 | | | o | | | | | |
| chromosome 1 open reading frame 51 | 1 | o | | | | | | | |
| chromosome 1 open reading frame 52 | 1 | | | | | | | | o |
| chromosome 1 open reading frame 71 | 1 | o | | | | | | | |
| chromosome 1 open reading frame 83 | 1 | | o | | | | | | |
| chromosome 1 open reading frame 93 | 1 | | | o | | | | | |
| chromosome 1 open reading frame 95 | 1 | | | | | o | | | |
| chromosome 1 open reading frame isoform cra_a | 1 | o | | | | | | | |
| chromosome 10 open reading frame 18 | 1 | | o | | | | | | |
| chromosome 10 open reading frame 26 | 1 | | | | | o | | | |
| chromosome 10 open reading frame 32 | 1 | o | | | | | | | |
| chromosome 10 open reading frame 58 | 1 | | o | | | | | | |
| chromosome 10 open reading frame 59 | 1 | o | | | | | | | |
| chromosome 10 open reading frame 72 | 1 | | | | | o | | | |
| chromosome 10 open reading frame 97 | 1 | o | | | | | | | |
| chromosome 12 open reading frame 43 | 1 | | | | | | o | | |
| chromosome 12 open reading frame 45 | 1 | o | | | | | | | |
| chromosome 14 open reading frame 152 | 1 | | | | | | | o | |
| chromosome 14 open reading frame 166b | 1 | o | | | | | | | |
| chromosome 14 open reading frame isoform cra_a | 1 | o | | | | | | | |
| chromosome 15 open reading frame 52 | 1 | o | | | | | | | |

| | | | | | | | | | |
|---|---|---|---|---|---|---|---|---|---|
| chromosome 16 open reading frame 62 protein | 1 | o | | | | | | | |
| chromosome 16 open reading frame 84 | 1 | o | | | | | | | |
| chromosome 17 open reading frame 28 | 1 | | | | | | | o | |
| chromosome 17 open reading frame 75 | 1 | | o | | | | | | |
| chromosome 17 open reading frame isoform cra_a | 1 | o | | | | | | | |
| chromosome 18 open reading frame isoform cra_a | 1 | o | | | | | | | |
| chromosome 19 open reading frame 26 | 1 | | | | | | | o | |
| chromosome 20 open reading frame 4 | 1 | o | | | | | | | |
| chromosome 20 open reading frame 43 | 1 | o | | | | | | | |
| chromosome 21 open reading frame 63 ortholog | 1 | o | | | | | | | |
| chromosome 22 open reading frame 28 | 1 | o | | | | | | | |
| chromosome 3 open reading frame 19 | 1 | o | | | | | | | |
| chromosome 3 open reading frame 23 | 1 | | o | | | | | | |
| chromosome 3 open reading frame 26 | 1 | | | o | | | | | |
| chromosome 3 open reading frame 70 | 1 | o | | | | | | | |
| chromosome 4 open reading frame 34 | 1 | | o | | | | | | |
| chromosome 5 open reading frame 22 | 1 | o | | | | | | | |
| chromosome 5 open reading frame 28 | 1 | o | | | | | | | |
| chromosome 5 open reading frame 35 | 1 | o | | | | | | | |
| chromosome 5 open reading frame isoform cra_a | 1 | | o | | | | | | |
| chromosome 6 open reading frame 150 | 1 | | | | o | | | | |
| chromosome 6 open reading frame 35 | 1 | | o | | | | | | |
| chromosome 7 open reading frame 25 | 1 | | o | | | | | | |
| chromosome 7 open reading frame 28b | 1 | | o | | | | | | |
| chromosome 7 open reading frame 36 | 1 | | | | | o | | | |
| chromosome 7 open reading frame 41 | 1 | o | | | | | | | |
| chromosome 7 open reading frame 64 | 1 | | | | | | | o | |
| chromosome 8 open reading frame 85-like | 1 | | | | | o | | | |
| chromosome 9 open reading frame 119 | 1 | | | | | o | | | |
| chromosome 9 open reading frame 125 | 1 | | | o | | | | | |
| chromosome 9 open reading frame 127 | 1 | | | | | | o | | |
| chromosome 9 open reading frame 61 | 1 | o | | | | | | | |
| chromosome 9 open reading frame 64 | 1 | o | | | | | | | |
| chromosome 9 open reading frame 89 | 1 | o | | | | | | | |
| chromosome segregation 1-like ( cerevisiae) | 1 | | | | | | | | o |
| chromosome segregation protein | 1 | o | | | | | | | |
| chromosome transmission fidelity protein 18 homolog | 1 | | | | | | | | o |
| chromosome x open reading frame 38 | 1 | o | | | | | | | |
| chromosome x open reading frame isoform cra_a | 1 | | | | | o | | | |
| churchill domain containing 1 | 1 | | | o | | | | | |
| chy zinc finger family protein | 1 | o | | | | | | | |
| chymotrypsin-like | 1 | | o | | | | | | |
| chymotrypsin-like elastase family member 1 precursor | 1 | | o | | | | | | |
| chymotrypsin-like elastase family member 2a | 1 | | o | | | | | | |
| chymotrypsin-like elastase member 3b-like | 1 | | o | | | | | | |
| ciliary neurotrophic factor receptor | 1 | | | | | | | o | |
| cingulin | 1 | | | | o | | | | |
| cingulin-like 1 | 1 | | | | | o | | | |
| citrate lyase beta like | 1 | o | | | | | | | |
| c-jun-amino-terminal kinase-interacting protein 1 | 1 | | | | | | | o | |

| | | | | | | | | | |
|---|---|---|---|---|---|---|---|---|---|
| c-jun-amino-terminal kinase-interacting protein 4-like | 1 | | o | | | | | | |
| cklf-like marvel transmembrane domain containing 3 | 1 | o | | | | | | | |
| cklf-like marvel transmembrane domain-containing protein 3 | 1 | | | | | | | | o |
| cl032 protein | 1 | | | | o | | | | |
| class i helical cytokine receptor number 16 | 1 | | | o | | | | | |
| class i helical cytokine receptor number 20 | 1 | o | | | | | | | |
| class i helical cytokine receptor number 26 | 1 | | | | | | o | | |
| class type 10a | 1 | | | o | | | | | |
| class type 11a | 1 | | | | | | o | | |
| class type 11a isoform cra_b | 1 | o | | | | | | | |
| class type 11b | 1 | o | | | | | | | |
| class vii unconventional myosin | 1 | | | | | o | | | |
| clathrin coat assembly protein ap180-like | 1 | | | | | | o | | |
| clathrin light chain a | 1 | | | | | | | | o |
| clathrin light chain a-like isoform 1 | 1 | | | | | | o | | |
| clathrin light chain a-like isoform 2 | 1 | | | | | | | | o |
| clathrin light chain b-like isoform 1 | 1 | | | | | | | o | |
| claudin 10 like | 1 | | | o | | | | | |
| claudin 11 | 1 | o | | | | | | | |
| claudin 12 | 1 | o | | | | | | | |
| claudin 5 | 1 | o | | | | | | | |
| claudin-4 | 1 | | | | o | | | | |
| cleavage and polyadenylation specificity factor subunit 3 | 1 | | | | | | | | o |
| cleavage and polyadenylation specificity factor subunit 3-like | 1 | | | | o | | | | |
| cleavage and polyadenylation specificity factor subunit 6 | 1 | | | | | | | | o |
| cleavage stimulation 3 pre- subunit 64kda | 1 | | o | | | | | | |
| cleavage stimulation 3 pre- subunit 77kda | 1 | | | | | | | o | |
| cleavage stimulation factor subunit 1-like | 1 | | | | | | | | o |
| clip-associating protein 1 | 1 | | | | | | o | | |
| clip-associating protein 2 | 1 | | | | | | o | | |
| c-maf-inducing protein | 1 | o | | | | | | | |
| c-mpl binding | 1 | | | o | | | | | |
| cmp-n-acetylneuraminate-beta-galactosamide-alpha- -sialyltransferase 1-like | 1 | | | | o | | | | |
| c-myc binding protein | 1 | o | | | | | | | |
| c-myc promoter-binding protein irlb | 1 | | | o | | | | | |
| c-myc promoter-binding protein isoform 2 | 1 | | | | | | | | o |
| cndp dipeptidase 2 (metallopeptidase m20 family) | 1 | o | | | | | | | |
| cntn5 protein | 1 | o | | | | | | | |
| coagulation factor b polypeptide | 1 | | o | | | | | | |
| coagulation factor ii receptor | 1 | o | | | | | | | |
| coagulation factor ii receptor-like 1 | 1 | | | | o | | | | |
| coagulation factor ii receptor-like 2 | 1 | | o | | | | | | |
| coagulation factor v ( labile factor) | 1 | | o | | | | | | |
| coagulation factor viii precursor | 1 | o | | | | | | | |
| coagulation factor vii-like | 1 | | | | o | | | | |
| coagulation factor xiii a chain precursor | 1 | | | | | | | o | |
| coagulation factor xiii b chain | 1 | | o | | | | | | |
| coagulation factor xiii b chain-like | 1 | o | | | | | | | |
| coatomer protein subunit alpha | 1 | | o | | | | | | |
| coatomer protein subunit beta 1 | 1 | | | o | | | | | |
| cobw domain containing protein | 1 | o | | | | | | | |

| | | | | | | | | | |
|---|---|---|---|---|---|---|---|---|---|
| cocaine- and amphetamine-regulated transcript | 1 | | | | | | | o | |
| cocaine- and amphetamine-regulated transcript ch9 | 1 | | | | | o | | | |
| co-chaperone protein mitochondrial precursor | 1 | o | | | | | | | |
| coenzyme q10 homolog a | 1 | o | | | | | | | |
| coenzyme q3 methyltransferase | 1 | | o | | | | | | |
| coenzyme q-binding protein coq10 homolog mitochondrial isoform 1 | 1 | | o | | | | | | |
| Cofilin-2 [Anoplopoma fimbria] | 1 | | | o | | | | | |
| cog4 protein | 1 | | | o | | | | | |
| coiled-coil and c2 domain containing 2a | 1 | o | | | | | | | |
| coiled-coil domain containing 109a | 1 | o | | | | | | | |
| coiled-coil domain containing 112 | 1 | o | | | | | | | |
| coiled-coil domain containing 117-like | 1 | | | | | | | o | |
| coiled-coil domain containing 126 | 1 | o | | | | | | | |
| coiled-coil domain containing 22 | 1 | o | | | | | | | |
| coiled-coil domain containing 3 | 1 | o | | | | | | | |
| coiled-coil domain containing 34 | 1 | o | | | | | | | |
| coiled-coil domain containing 49 | 1 | | o | | | | | | |
| coiled-coil domain containing 62 | 1 | o | | | | | | | |
| coiled-coil domain containing 84 | 1 | o | | | | | | | |
| coiled-coil domain containing 90b | 1 | | | o | | | | | |
| coiled-coil domain containing 96-like isoform 2 | 1 | | o | | | | | | |
| coiled-coil domain-containing protein 109a-like | 1 | o | | | | | | | |
| coiled-coil domain-containing protein 134 | 1 | | | | | | | o | |
| coiled-coil domain-containing protein 134-like | 1 | | o | | | | | | |
| coiled-coil domain-containing protein 135-like | 1 | | | | | | | | o |
| coiled-coil domain-containing protein 137-like | 1 | | | | o | | | | |
| coiled-coil domain-containing protein 25 | 1 | | | | | | | o | |
| coiled-coil domain-containing protein 30 | 1 | o | | | | | | | |
| coiled-coil domain-containing protein 47 | 1 | | | | o | | | | |
| coiled-coil domain-containing protein 55 | 1 | o | | | | | | | |
| coiled-coil domain-containing protein 84-like | 1 | o | | | | | | | |
| coiled-coil domain-containing protein 9 | 1 | o | | | | | | | |
| coiled-coil domain-containing protein 97 | 1 | o | | | | | | | |
| coiled-coil domain-containing protein 97-like | 1 | | | | | | | | o |
| coiled-coil-helix-coiled-coil-helix domain containing 4 | 1 | o | | | | | | | |
| col4a1 protein | 1 | o | | | | | | | |
| collagen a3 | 1 | o | | | | | | | |
| collagen family member (col-135) | 1 | o | | | | | | | |
| collagen prolyl 4-hydroxylase alpha iii subunit | 1 | o | | | | | | | |
| collagen triple helix repeat protein | 1 | | | | o | | | | |
| collagen triple helix repeat tail fiber protein from prophage | 1 | | | | o | | | | |
| collagen type iv alpha-3-binding protein | 1 | | o | | | | | | |
| collagen type vi alpha 4-like | 1 | o | | | | | | | |
| collagen type viii alpha 1 | 1 | o | | | | | | | |
| collagen type xi alpha2 | 1 | | | | | o | | | |
| collagen type xviii alpha 1 | 1 | o | | | | | | | |
| collagenase 3 | 1 | | | o | | | | | |
| collagenase 3-like | 1 | | | | o | | | | |
| collectin sub-family member 10 | 1 | | | o | | | | | |
| collectin sub-family member 11 | 1 | | o | | | | | | |
| colony stimulating factor 2 low-affinity (granulocyte-macrophage) | 1 | | | o | | | | | |

| | | | | | | | | | |
|---|---|---|---|---|---|---|---|---|---|
| colony-stimulating factor receptor 1a | 1 | | | | o | | | | |
| comm domain-containing protein 2 | 1 | o | | | | | | | |
| comm domain-containing protein 4 | 1 | | | | | | o | | |
| comm domain-containing protein 6-like | 1 | | | | | | | | o |
| comm domain-containing protein 7 | 1 | | o | | | | | | |
| complement 1 subcomponent q polypeptide beta | 1 | | o | | | | | | |
| complement 1 subcomponent q polypeptide gamma | 1 | o | | | | | | | |
| complement c1q subcomponent subunit a | 1 | o | | | | | | | |
| complement c1q-like adipose specific protein | 1 | | o | | | | | | |
| complement c1q-like protein 3-like | 1 | | | | | o | | | |
| complement c1r subcomponent precursor | 1 | | | | o | | | | |
| complement c1r subcomponent-like | 1 | | o | | | | | | |
| complement component 5 | 1 | | | o | | | | | |
| complement component c8 alpha chain | 1 | | | | o | | | | |
| complement component c8 beta chain precursor | 1 | | | | o | | | | |
| complement component c8 gamma chain precursor | 1 | | | | o | | | | |
| complement component q a chain | 1 | | | o | | | | | |
| complement component q c chain | 1 | | | o | | | | | |
| complement component q subcomponent binding protein | 1 | | | | o | | | | |
| complement component q subcomponent-like 2 | 1 | o | | | | | | | |
| complement component q subcomponent-like 4 like | 1 | | | o | | | | | |
| complement control-like protein | 1 | | | | o | | | | |
| complement receptor type 2 | 1 | | | | | | | o | |
| complement receptor-like | 1 | o | | | | | | | |
| complexin 2 | 1 | | | | | | o | | |
| component of oligomeric golgi complex isoform cra_d | 1 | | o | | | | | | |
| condensin complex subunit 2-like | 1 | | | | o | | | | |
| condensin-2 complex subunit g2 | 1 | | o | | | | | | |
| cone cgmp-specific 3 -cyclic phosphodiesterase subunit alpha | 1 | | | | o | | | | |
| connexin | 1 | o | | | | | | | |
| conserved hypothetical protein [Staphylococcus aureus A8796] | 1 | | | | o | | | | |
| conserved hypothetical secreted protein | 1 | o | | | | | | | |
| conserved oligomeric golgi complex subunit 3 | 1 | | | o | | | | | |
| conserved oligomeric golgi complex subunit partial | 1 | | o | | | | | | |
| conserved proline-rich protein | 1 | | | | o | | | | |
| conserved protein | 1 | | | | | o | | | |
| constitutive coactivator of ppar-gamma-like protein 2-like | 1 | | | | | | | o | |
| contactin associated protein 1 | 1 | | | | | | | o | |
| contactin-1a precursor | 1 | | | | | o | | | |
| contactin-associated 4-like | 1 | | | | | o | | | |
| copine family member isoform cra_c | 1 | | | | | | o | | |
| copine ii | 1 | | | | | | | | o |
| copine iv | 1 | | | | | | o | | |
| copine vii | 1 | | | | | | o | | |
| copine-4-like | 1 | | | | | | | | o |
| copper transporting atpase 2 | 1 | | o | | | | | | |
| copper-transporting atpase 2 | 1 | | o | | | | | | |
| cops7a protein | 1 | | | | | | o | | |

| | | | | | | | | | |
|---|---|---|---|---|---|---|---|---|---|
| core-binding beta subunit | 1 | o | | | | | | | |
| corin protein | 1 | o | | | | | | | |
| corin variant1 | 1 | o | | | | | | | |
| cornichon homolog 4 | 1 | | | | | | o | | |
| corticotropin releasing hormone binding protein | 1 | | | | | | | o | |
| corticotropin-releasing factor-binding protein precursor | 1 | | | | | | | | o |
| counting factor associated protein d-like | 1 | | | | | | | | o |
| coup transcription factor 2 | 1 | | | | | o | | | |
| cp059_bovin ame: full=uncharacterized protein c16orf59 homolog | 1 | o | | | | | | | |
| cpd photolyase-like | 1 | | | | | | o | | |
| cr032_anofi ame: full=upf0729 protein c18orf32 homolog | 1 | | | | | o | | | |
| cre-ama-1 protein | 1 | | | | | | o | | |
| creatine mitochondrial 1b | 1 | | | | | | | | o |
| creatine muscle | 1 | o | | | | | | | |
| creb binding protein | 1 | o | | | | | | | |
| creb regulated transcription coactivator 1 | 1 | | | | | | o | | |
| creb-regulated transcription coactivator 1 | 1 | | | | | | | | o |
| creb-regulated transcription coactivator 1-like | 1 | | | | | | o | | |
| cre-clec-180 protein | 1 | o | | | | | | | |
| cre-clp-4 protein | 1 | | | | | | o | | |
| crossover junction endonuclease eme1 | 1 | | | | o | | | | |
| crumbs homolog 3 isoform cra_a | 1 | | o | | | | | | |
| cryptochrome dash | 1 | | | | o | | | | |
| cst complex subunit ctc1 | 1 | | o | | | | | | |
| cst complex subunit stn1 | 1 | | | | | | | | o |
| ctd small phosphatase-like protein 2 | 1 | o | | | | | | | |
| ctd small phosphatase-like protein 2-a-like | 1 | | | | | | | | o |
| ctnna2 protein | 1 | | | | | | o | | |
| cttnbp2 n-terminal-like | 1 | o | | | | | | | |
| c-type lectin a | 1 | | | o | | | | | |
| c-type lectin domain family 10 member a-like isoform 2 | 1 | | o | | | | | | |
| c-type lectin domain family 4 member f | 1 | | o | | | | | | |
| c-type lectin receptor | 1 | o | | | | | | | |
| c-type lectin superfamily member g | 1 | | | o | | | | | |
| c-type mbl-2 protein | 1 | | | | | | o | | |
| cu zn superoxide dismutase | 1 | | | | o | | | | |
| cu++ alpha polypeptide | 1 | o | | | | | | | |
| cub and sushi domain-containing protein 1 | 1 | | | | | | | | o |
| cub and sushi domain-containing protein 3 | 1 | | | | | | o | | |
| cue domain containing 2 | 1 | o | | | | | | | |
| cugbp elav-like family member 2 isoform 1 | 1 | | | | | | o | | |
| cugbp elav-like family member 2-like | 1 | | | | | | o | | |
| cugbp elav-like family member 3 | 1 | | | | | o | | | |
| cullin-2 isoform 1 | 1 | | | | o | | | | |
| curculin domain protein (mannose-binding) lectin | 1 | o | | | | | | | |
| cutc copper transporter homolog | 1 | o | | | | | | | |
| cut-like 1 | 1 | | o | | | | | | |
| cut-like homeobox 1 | 1 | | o | | | | | | |
| cwf19-like protein 2 | 1 | o | | | | | | | |
| cxc chemokine d1 | 1 | o | | | | | | | |
| c-x-c chemokine receptor 4 | 1 | o | | | | | | | |
| cxxc-type zinc finger protein 5 | 1 | | o | | | | | | |
| cyc8_dicdi ame: full=general transcriptional corepressor trfa | 1 | o | | | | | | | |

| | | | | | | | | | |
|---|---|---|---|---|---|---|---|---|---|
| cyclic amp-dependent transcription factor atf-7 | 1 | o | | | | | | | |
| cyclic amp-responsive element-binding protein 3-like protein 3-a | 1 | | o | | | | | | |
| cyclic nucleotide gated channel alpha 2 | 1 | | | | o | | | | |
| -cyclic-nucleotide 3-phosphodiesterase | 1 | o | | | | | | | |
| cyclin b | 1 | | | | o | | | | |
| cyclin c | 1 | | | | | | o | | |
| cyclin d1 | 1 | o | | | | | | | |
| cyclin d2 | 1 | o | | | | | | | |
| cyclin g2 | 1 | o | | | | | | | |
| cyclin t2 isoform 1 | 1 | o | | | | | | | |
| cyclin-dependent kinase 12 | 1 | | | | | | | | o |
| cyclin-dependent kinase 2-interacting protein | 1 | | o | | | | | | |
| cyclin-dependent kinase inhibitor 1c | 1 | | | | | | | | o |
| cyclin-dependent kinase inhibitor 2d | 1 | o | | | | | | | |
| cyclin-dependent kinase-like 5 | 1 | | | | | | | o | |
| cyclin-related protein fam58a-like isoform 1 | 1 | | | | | | | | o |
| cyclin-t2 isoform 1 | 1 | | | | o | | | | |
| cyclin-y-like protein 1-like | 1 | | | | o | | | | |
| cyclophilin 1 | 1 | | | | | | o | | |
| cyclophilin-rna interacting protein | 1 | | | | | | o | | |
| cyclophilin-type peptidyl-prolyl cis-trans isomerase- bmcyp-2 | 1 | | | | | o | | | |
| cygb2_oryla ame: full=cytoglobin-2 | 1 | | | | | | | | o |
| cyp2d6-a-prov protein | 1 | | | o | | | | | |
| cystathionine beta-synthase-like | 1 | | | | o | | | | |
| cystathionine-beta-synthase | 1 | | | | | o | | | |
| cystatin f | 1 | | | | o | | | | |
| cysteine and glycine-rich protein 2 | 1 | | | | | o | | | |
| cysteine and histidine-rich domain-containing protein 1 | 1 | o | | | | | | | |
| cysteine protease atg4b-like isoform 1 | 1 | | o | | | | | | |
| cysteine protease atg4c | 1 | | | | | | o | | |
| cysteine type i | 1 | | o | | | | | | |
| cysteine-rich protein 2 | 1 | o | | | | | | | |
| cysteine-rich with egf-like domains 1 | 1 | o | | | | | | | |
| cystine glutamate transporter-like | 1 | | | | | | o | | |
| cytadherence-associated protein | 1 | | | | | | | | o |
| cytidine monophospho-n-acetylneuraminic acid synthetase | 1 | o | | | | | | | |
| cytochrome b ascorbate-dependent protein 3 | 1 | | | | o | | | | |
| cytochrome b5 reductase 3 | 1 | o | | | | | | | |
| cytochrome b5 reductase 4 | 1 | | | | | | | | o |
| cytochrome b5 type b | 1 | | | | o | | | | |
| cytochrome b-c1 complex subunit mitochondrial | 1 | | | | | | | | o |
| cytochrome c oxidase assembly protein cox15 homolog | 1 | o | | | | | | | |
| cytochrome c oxidase assembly protein cox16 mitochondrial-like | 1 | | | | o | | | | |
| cytochrome c oxidase subunit 7a-related mitochondrial-like | 1 | | | | o | | | | |
| cytochrome c oxidase subunit viia polypeptide 2 | 1 | | o | | | | | | |
| cytochrome c oxidase subunit viic | 1 | | | | o | | | | |
| cytochrome c-type heme lyase | 1 | o | | | | | | | |
| cytochrome family subfamily polypeptide 4 | 1 | | | o | | | | | |
| cytochrome family subfamily polypeptide 65 | 1 | | o | | | | | | |

| | | | | | | | | | |
|---|---|---|---|---|---|---|---|---|---|
| cytochrome family subfamily polypeptide isoform cra_a | 1 | o | | | | | | | |
| cytochrome p450 20a1 | 1 | o | | | | | | | |
| cytochrome p450 2g1-like | 1 | | o | | | | | | |
| cytochrome p450 2j6-like | 1 | | | | o | | | | |
| cytochrome p450 3a80 | 1 | | o | | | | | | |
| cytochrome p450 family 17 polypeptide 2 | 1 | | o | | | | | | |
| cytochrome p450 monooxygenase | 1 | | o | | | | | | |
| cytochrome p450 monooxygenase cyp2j2 | 1 | | | | | o | | | |
| cytochrome p4501c1 | 1 | o | | | | | | | |
| cytochrome partial | 1 | | | | | | | | o |
| cytohesin 1 interacting protein | 1 | o | | | | | | | |
| cytohesin 2-like | 1 | | | o | | | | | |
| cytohesin-1 | 1 | o | | | | | | | |
| cytokine receptor gamma chain | 1 | | | | | o | | | |
| cytokine-like protein 1 precursor | 1 | o | | | | | | | |
| cytoplasmatic actin | 1 | | o | | | | | | |
| cytoplasmic dynein 1 intermediate chain 2-like | 1 | | | | o | | | | |
| cytoplasmic dynein 1 light intermediate chain 2 | 1 | o | | | | | | | |
| cytoplasmic dynein 2 light intermediate chain 1-like | 1 | | | | | | | | o |
| cytoplasmic dynein intermediate chain 2 isoform | 1 | | | | | o | | | |
| cytoplasmic fmr1-interacting protein 1-like | 1 | | | | | | | | o |
| cytoplasmic linker associated protein 1 | 1 | | | | | | o | | |
| cytoplasmic phosphatidylinositol transfer protein 1 | 1 | | | | | | o | | |
| cytoplasmic trna 2-thiolation protein 1-like | 1 | | | | o | | | | |
| cytoskeletal associated protein | 1 | o | | | | | | | |
| cytoskeleton-associated protein 2-like | 1 | | | | o | | | | |
| cytosolic 5 -nucleotidase iii-like | 1 | | | | | | | | o |
| cytosolic carboxypeptidase 1 | 1 | | | | | | | | o |
| cytosolic carboxypeptidase-like protein 5 | 1 | | | | | | o | | |
| cytosolic fe-s cluster assembly factor nubp1 | 1 | o | | | | | | | |
| cytosolic phospholipase a2 | 1 | o | | | | | | | |
| cytosolic phospholipase a2 zeta-like | 1 | | | | | | | | o |
| cytosolic purine 5 -nucleotidase | 1 | | o | | | | | | |
| cytospin a | 1 | o | | | | | | | |
| d2hgdh protein | 1 | o | | | | | | | |
| d-2-hydroxyglutarate mitochondrial-like | 1 | | | | | | o | | |
| dcn1-like protein 4-like | 1 | o | | | | | | | |
| dcp2 decapping enzyme homolog ( cerevisiae) | 1 | o | | | | | | | |
| ddb1- and cul4-associated factor 12 | 1 | o | | | | | | | |
| ddb1- and cul4-associated factor 15 | 1 | | | | | o | | | |
| d-dopachrome decarboxylase | 1 | | o | | | | | | |
| ddrgk domain-containing protein 1 | 1 | | o | | | | | | |
| dead (asp-glu-ala-asp) box polypeptide 10 | 1 | | o | | | | | | |
| dead (asp-glu-ala-asp) box polypeptide 20 | 1 | o | | | | | | | |
| dead (asp-glu-ala-asp) box polypeptide 24 | 1 | o | | | | | | | |
| dead (asp-glu-ala-asp) box polypeptide 3 | 1 | o | | | | | | | |
| dead (asp-glu-ala-asp) box polypeptide 50 | 1 | | o | | | | | | |
| dead (asp-glu-ala-asp) box polypeptide 52 | 1 | o | | | | | | | |
| dead (asp-glu-ala-asp) box polypeptide 55 | 1 | | | o | | | | | |
| dead eye | 1 | o | | | | | | | |
| deah (asp-glu-ala-asp his) box polypeptide 57 | 1 | | o | | | | | | |
| deah (asp-glu-ala-his) box polypeptide 30 | 1 | o | | | | | | | |
| deah (asp-glu-ala-his) box polypeptide 30-like | 1 | | | | | o | | | |
| death effector domain- isoform cra_a | 1 | | o | | | | | | |

| | | | | | | | | | |
|---|---|---|---|---|---|---|---|---|---|
| death-associated protein | 1 | o | | | | | | | |
| death-associated protein kinase 3 | 1 | o | | | | | | | |
| decorin precursor | 1 | | | | | | | | o |
| dedicator of cytokinesis 1-like | 1 | o | | | | | | | |
| dedicator of cytokinesis 5 | 1 | o | | | | | | | |
| dedicator of cytokinesis 7 | 1 | | | | | | | o | |
| dedicator of cytokinesis protein 10 | 1 | o | | | | | | | |
| dedicator of cytokinesis protein 4 | 1 | | | | | | o | | |
| dedicator of cytokinesis protein 4-like | 1 | | | | | | o | | |
| dedicator of cytokinesis protein 6 | 1 | | o | | | | | | |
| defective in cullin neddylation domain containing 2 ( cerevisiae) | 1 | | | | | | o | | |
| deformed epidermal autoregulatory factor 1 homolog | 1 | o | | | | | | | |
| dehydrogenase reductase (sdr family) x-linked | 1 | | | o | | | | | |
| dehydrogenase reductase sdr family member 11 | 1 | | | o | | | | | |
| deleted in bladder cancer protein 1 | 1 | | | | | | | | o |
| deleted in malignant brain tumors 1 partial | 1 | | o | | | | | | |
| deleted in malignant brain tumors 1 protein | 1 | o | | | | | | | |
| deleted in malignant brain tumors 1-like | 1 | | | o | | | | | |
| delta( )-delta( )-dienoyl- mitochondrial-like | 1 | | | | o | | | | |
| delta-9-desaturase 2 | 1 | | o | | | | | | |
| delta-aminolevulinic acid dehydratase | 1 | o | | | | | | | |
| delta-like 1 | 1 | | | o | | | | | |
| delta-like 1-like protein | 1 | | | | | | o | | |
| delta-like protein 1 | 1 | | | | | | o | | |
| delta-like protein c precursor | 1 | | | o | | | | | |
| delta-sarcoglycan | 1 | o | | | | | | | |
| deltex 4 homolog | 1 | | | | | | o | | |
| deltex homolog 2 | 1 | | | | | | o | | |
| denn domain-containing protein 5a | 1 | | | | | | o | | |
| denn madd domain containing 1b | 1 | | | o | | | | | |
| denn madd domain containing 4a | 1 | | | o | | | | | |
| dentin matrix protein 4 | 1 | | | | | | o | | |
| dentin matrix protein 4-like | 1 | | | o | | | | | |
| deoxycytidine kinase | 1 | | | | o | | | | |
| deoxycytidylate deaminase | 1 | o | | | | | | | |
| deoxycytidylate deaminase isoform 2 | 1 | o | | | | | | | |
| deoxyguanosine mitochondrial | 1 | o | | | | | | | |
| deoxynucleotidyltransferase terminal-interacting protein 1 | 1 | o | | | | | | | |
| deoxynucleotidyltransferase terminal-interacting protein 2-like | 1 | | | | o | | | | |
| deoxyribonuclease i-like 1 | 1 | o | | | | | | | |
| deoxyribonuclease i-like 3 | 1 | o | | | | | | | |
| deoxyribonuclease tatdn2 | 1 | | | | o | | | | |
| dep domain-containing protein 7 | 1 | o | | | | | | | |
| dephospho- kinase domain-containing | 1 | | | | | | | | o |
| dermatan 4 sulfotransferase 1 | 1 | o | | | | | | | |
| dermatopontin | 1 | o | | | | | | | |
| desmoglein 2 | 1 | | o | | | | | | |
| desmoglein 4 | 1 | o | | | | | | | |
| desmoplakin | 1 | o | | | | | | | |
| desmoplakin a | 1 | o | | | | | | | |
| deubiquitinating protein vcip135 | 1 | | | | o | | | | |
| developmentally regulated rna-binding protein 1 | 1 | o | | | | | | | |
| developmentally-regulated gtp-binding protein 2 | 1 | | o | | | | | | |

| | | | | | | | | | |
|---|---|---|---|---|---|---|---|---|---|
| dexamethasone-induced ras-related protein 1-like | 1 | | | | | | | | o |
| dexamethasone-induced transcript | 1 | o | | | | | | | |
| dexh (asp-glu-x-his) box polypeptide 58 | 1 | | o | | | | | | |
| dgkb protein | 1 | | | | | | o | | |
| d-glucuronyl c5-epimerase | 1 | | | | | | o | | |
| diacylglycerol acyltransferase | 1 | o | | | | | | | |
| diacylglycerol beta 90kda | 1 | | | | | | o | | |
| diacylglycerol eta-like | 1 | | | | | | o | | |
| diacylglycerol kinase alpha | 1 | | | o | | | | | |
| diacylglycerol kinase zeta | 1 | | | | | | o | | |
| dickkopf-related protein 3 precursor | 1 | | | | | | | o | |
| diencephalon mesencephalon homeobox protein 1-b-like isoform 1 | 1 | | | | o | | | | |
| differentiation regulator | 1 | o | | | | | | | |
| dihydrodipicolinate synthase- mitochondrial precursor | 1 | | o | | | | | | |
| dihydrolipoamide s-acetyltransferase-like | 1 | | o | | | | | | |
| dihydrolipoamide s-succinyltransferase (e2 component of 2-oxo-glutarate complex) isoform cra_a | 1 | | | | | | | | o |
| dihydropteridine reductase | 1 | o | | | | | | | |
| dihydropyrimidinase | 1 | | o | | | | | | |
| dihydropyrimidinase-like 2 | 1 | | | | | | o | | |
| dihydrouridine synthase 4-like | 1 | o | | | | | | | |
| dihydroxyacetone phosphate acyltransferase | 1 | | o | | | | | | |
| dimethylarginine dimethylaminohydrolase 1 | 1 | o | | | | | | | |
| dimethylarginine dimethylaminohydrolase 2 | 1 | o | | | | | | | |
| dimethylglycine mitochondrial-like | 1 | | | o | | | | | |
| di-n-acetyl- | 1 | | | | | o | | | |
| dip2 disco-interacting protein 2 homolog b | 1 | | | | | | o | | |
| dipeptidyl aminopeptidase-like protein 6 | 1 | | | | | | o | | |
| dipeptidyl aminopeptidase-like protein 6-like | 1 | | | | | | o | | |
| dipeptidyl peptidase 4 | 1 | o | | | | | | | |
| dipeptidyl peptidase 9 | 1 | | o | | | | | | |
| dipeptidyl-peptidase 7 | 1 | | o | | | | | | |
| diphthamide biosynthesis | 1 | o | | | | | | | |
| diphthamide biosynthesis protein 1 | 1 | | | | | | | | o |
| diphthamide biosynthesis protein 1-like | 1 | o | | | | | | | |
| dis3 mitotic control homolog ( cerevisiae) | 1 | | | o | | | | | |
| dis3-like exonuclease 2 | 1 | o | | | | | | | |
| disabled homolog 2 | 1 | o | | | | | | | |
| disco-interacting protein 2 homolog b-a-like | 1 | | | | | | o | | |
| dishevelled 3 | 1 | | o | | | | | | |
| disintegrin and metalloproteinase domain-containing protein 10 | 1 | | | | | | | o | |
| disks large homolog 5 | 1 | | | | o | | | | |
| disks large-associated protein 1 | 1 | | | | | | o | | |
| disks large-associated protein 2-like | 1 | | | | | | o | | |
| disrupted in renal carcinoma 2 | 1 | | | o | | | | | |
| distal-less homeobox 6 | 1 | | | | | | o | | |
| disulfide-isomerase precursor | 1 | | | o | | | | | |
| dix domain containing 1 | 1 | | | | | o | | | |
| dlgh4 protein | 1 | | | | | o | | | |
| dna (cytosine-5)-methyltransferase 1 | 1 | | o | | | | | | |
| dna chr erato doi isoform cra_b | 1 | | | | | | | o | |
| dna chr wayne state university expressed | 1 | | | o | | | | | |
| dna cross-link repair 1b (pso2 cerevisiae) | 1 | | | | | | | o | |
| dna cross-link repair 1b protein | 1 | o | | | | | | | |
| dna damage-binding protein 2 | 1 | | | o | | | | | |

| | | | | | | | | | |
|---|---|---|---|---|---|---|---|---|---|
| dna damage-binding protein 2-like | 1 | | | | o | | | | |
| dna excision repair protein ercc-1 | 1 | o | | | | | | | |
| dna methyltransferase | 1 | | o | | | | | | |
| dna methyltransferase 1 associated protein 1 | 1 | o | | | | | | | |
| dna methyltransferase 3a | 1 | o | | | | | | | |
| dna methyltransferase 3b | 1 | | o | | | | | | |
| dna mismatch repair protein mlh3 | 1 | o | | | | | | | |
| dna mismatch repair protein msh3 | 1 | | | | | o | | | |
| dna polymerase alpha gram-positive type | 1 | | | o | | | | | |
| dna polymerase eta | 1 | | | | o | | | | |
| dna polymerase iii alpha subunit | 1 | o | | | | | | | |
| dna polymerase iii polc-type-like | 1 | | | | | | | | o |
| dna polymerase kappa | 1 | o | | | | | | | |
| dna polymerase zeta catalytic subunit | 1 | o | | | | | | | |
| dna primase large subunit | 1 | o | | | | | | | |
| dna primase small subunit | 1 | | | | o | | | | |
| dna repair protein rad51 homolog 2 | 1 | | | | o | | | | |
| dna repair protein rev1-like | 1 | o | | | | | | | |
| dna replication complex gins protein psf1 | 1 | | o | | | | | | |
| dna replication complex gins protein psf2-like | 1 | | | | o | | | | |
| dna replication complex gins protein sld5 | 1 | o | | | | | | | |
| dna topoisomerase 1-like | 1 | | | | o | | | | |
| dna topoisomerase 2-alpha | 1 | | o | | | | | | |
| dna topoisomerase 2-beta | 1 | | | | | o | | | |
| dna topoisomerase 2-binding protein 1 | 1 | o | | | | | | | |
| dna topoisomerase 3-beta-1 | 1 | | | | | | | | o |
| dna-binding death effector domain-containing protein 2 | 1 | o | | | | | | | |
| dna-binding protein inhibitor id-4-like | 1 | | | | | | | | o |
| dna-binding protein smubp-2 | 1 | | | | | o | | | |
| dna-dependent dna polymerase | 1 | | | | | | o | | |
| dna-dependent protein kinase catalytic subunit isoform 2 | 1 | o | | | | | | | |
| dna-directed rna polymerase i subunit rpa1 | 1 | o | | | | | | | |
| dna-directed rna polymerase i subunit rpa1-like | 1 | o | | | | | | | |
| dna-directed rna polymerase ii subunit rpb4 | 1 | | o | | | | | | |
| dna-directed rna polymerase iii subunit rpc1 | 1 | | | | o | | | | |
| dna-directed rna polymerase iii subunit rpc2 | 1 | | | | o | | | | |
| dna-directed rna polymerase iii subunit rpc3 | 1 | | | | o | | | | |
| dna-directed rna polymerase iii subunit rpc4-like | 1 | | | | | | | | o |
| dna-directed rna polymerase iii subunit rpc8-like | 1 | | | | o | | | | |
| dna-directed rna polymerases and iii subunit rpabc3-like | 1 | | | | o | | | | |
| dna-directed rna polymerases and iii subunit rpabc5 | 1 | | | | | o | | | |
| dna-directed rna polymerases n 8 kda subunit superfamily | 1 | o | | | | | | | |
| dnaj homolog subfamily a member 1-like | 1 | | | | o | | | | |
| dnaj homolog subfamily a member 4-like | 1 | | | | o | | | | |
| dnaj homolog subfamily b member 14 | 1 | | o | | | | | | |
| dnaj homolog subfamily b member 4 | 1 | | | | | | | | o |
| dnaj homolog subfamily b member 9-like | 1 | | | | o | | | | |
| dnaj homolog subfamily c member 17 | 1 | o | | | | | | | |
| dnaj homolog subfamily c member 22 | 1 | | | o | | | | | |
| dnaj homolog subfamily c member 25 | 1 | o | | | | | | | |
| dnaj homolog subfamily c member 25-like | 1 | | | o | | | | | |
| dnaj homolog subfamily c member 30 | 1 | o | | | | | | | |

| | | | | | | | | | |
|---|---|---|---|---|---|---|---|---|---|
| dnaj homolog subfamily c member 30-like | 1 | | | | | | | | o |
| dnaj homolog subfamily c member 4 | 1 | | | o | | | | | |
| dnaj homolog subfamily c member 7 | 1 | | | | | | | | o |
| dnajc13 protein | 1 | | | | | | | | o |
| dock8 protein | 1 | | o | | | | | | |
| dohh_drops ame: full=deoxyhypusine hydroxylase short=dohh ame: full=deoxyhypusine dioxygenase ame: full=deoxyhypusine monooxygenase | 1 | o | | | | | | | |
| dolichyl pyrophosphate phosphatase 1 | 1 | o | | | | | | | |
| dolichyl-diphosphooligosaccharide--protein glycosyltransferase subunit 1 | 1 | | | o | | | | | |
| dolichyl-diphosphooligosaccharide--protein glycosyltransferase subunit dad1 | 1 | | | | | | | | o |
| dolichyl-diphosphooligosaccharide--protein glycosyltransferase subunit stt3b-like | 1 | | | | | | o | | |
| dolichyl-phosphate beta-glucosyltransferase | 1 | | | o | | | | | |
| dolichyl-phosphate beta-glucosyltransferase isoform 1 | 1 | o | | | | | | | |
| domain and heat repeat-containing protein kiaa1468 homolog | 1 | | o | | | | | | |
| domain member 1b | 1 | | | | | | | o | |
| dopamine receptor d2 | 1 | | | | | | o | | |
| dopamine receptor d5 | 1 | | | | | o | | | |
| dopey1 protein | 1 | | o | | | | | | |
| dorsal root ganglia homeobox | 1 | o | | | | | | | |
| dot1- histone h3 methyltransferase ( cerevisiae) isoform cra_b | 1 | | | | | | o | | |
| doublecortin | 1 | | | | | | | o | |
| doublecortin domain containing 2 | 1 | o | | | | | | | |
| doublecortin kinase-2 | 1 | | | | | | o | | |
| doublecortin-like kinase 2 | 1 | | | o | | | | | |
| double-stranded rna activated protein kinase 1 | 1 | | o | | | | | | |
| double-stranded rna-dependent protein kinase | 1 | | | o | | | | | |
| down syndrome cell adhesion molecule-like protein 1-like | 1 | | | | | | o | | |
| down syndrome critical region gene 1-like 1 | 1 | | | | | | o | | |
| down syndrome critical region gene 1-like 2 | 1 | | | | | o | | | |
| down syndrome critical region protein 3-like | 1 | | o | | | | | | |
| downstream of isoform cra_a | 1 | | | | | | o | | |
| dph3 homolog | 1 | o | | | | | | | |
| dpy-19-like 1 ( elegans) | 1 | o | | | | | | | |
| drebrin-like isoform 2 | 1 | | | | | | | | o |
| drtp1 [Larimichthys crocea] | 1 | | o | | | | | | |
| dsrna-activated protein kinase r | 1 | | | o | | | | | |
| dtw domain containing 2 | 1 | | o | | | | | | |
| dual specificity phosphatase 10 | 1 | o | | | | | | | |
| dual specificity phosphatase 14 | 1 | | | | | | o | | |
| dual specificity phosphatase 22 | 1 | o | | | | | | | |
| dual specificity phosphatase 4 | 1 | | | | o | | | | |
| dual specificity phosphatase 6 | 1 | o | | | | | | | |
| dual specificity phosphatase 7 | 1 | o | | | | | | | |
| dual specificity phosphatase dupd1 | 1 | o | | | | | | | |
| dual specificity protein kinase clk2 | 1 | | | | | | | | o |
| dual specificity protein phosphatase 26-like | 1 | o | | | | | | | |
| dual specificity protein phosphatase 8 | 1 | | | | | | o | | |
| dual specificity protein phosphatase cdc14b | 1 | | | | | o | | | |
| dual specificity tyrosine-phosphorylation-regulated kinase 1a | 1 | | | | | | o | | |

| | | | | | | | | | |
|---|---|---|---|---|---|---|---|---|---|
| dual specificity tyrosine-phosphorylation-regulated kinase 1a-like | 1 | o | | | | | | | |
| dual-specificity tyrosine- -phosphorylation regulated kinase 3 | 1 | o | | | | | | | |
| duf307 family protein | 1 | o | | | | | | | |
| dullard homolog (xenopus laevis) | 1 | | o | | | | | | |
| dupd1 protein | 1 | o | | | | | | | |
| dusty protein kinase | 1 | | | | o | | | | |
| dynactin 1a | 1 | o | | | | | | | |
| dynactin 4 | 1 | o | | | | | | | |
| dynamin 2 isoform 2 isoform 16 | 1 | | | | | o | | | |
| dynamin 3 | 1 | o | | | | | | | |
| dynamin binding protein | 1 | | o | | | | | | |
| dynamin isoform cra_b | 1 | | o | | | | | | |
| dynamin-1-like protein | 1 | o | | | | | | | |
| dynein cytoplasmic 2 heavy chain 1 | 1 | | | | | | o | | |
| dynein heavy | 1 | | | | o | | | | |
| dynein heavy chain axonemal | 1 | | | | | | | | o |
| dynein light chain cytoplasmic-like | 1 | | | | o | | | | |
| dynein light chain tctex-type 1 | 1 | | | | | | o | | |
| dysbindin (dystrobrevin binding protein 1) domain containing 1 | 1 | o | | | | | | | |
| dysbindin domain-containing protein 1-like | 1 | | | | | | o | | |
| dysferlin interacting protein 1 | 1 | o | | | | | | | |
| dystonin isoform 1 | 1 | | | | | | o | | |
| dystrobrevin alpha | 1 | o | | | | | | | |
| dystrobrevin beta | 1 | | | | | | | o | |
| dystrobrevin beta- partial | 1 | | o | | | | | | |
| dystrophin | 1 | | | | | | o | | |
| dzip1 protein | 1 | o | | | | | | | |
| e1a binding protein isoform cra_b | 1 | | | | | | | o | |
| e1a binding protein p400 | 1 | | | | | | | o | |
| e1a-binding protein p400-like | 1 | | | | o | | | | |
| e2-induced gene 5 protein homolog | 1 | | | | o | | | | |
| e3 sumo-protein ligase 2-like | 1 | o | | | | | | | |
| e3 sumo-protein ligase pias2 | 1 | o | | | | | | | |
| e3 ubiquitin-protein ligase dtx3l | 1 | | | | | | | | o |
| e3 ubiquitin-protein ligase dtx3l-like | 1 | | | | o | | | | |
| e3 ubiquitin-protein ligase hectd1 | 1 | | | | | | o | | |
| e3 ubiquitin-protein ligase herc2-like | 1 | | | | | | | | o |
| e3 ubiquitin-protein ligase listerin-like | 1 | | | | | | | | o |
| e3 ubiquitin-protein ligase march4-like | 1 | | | | | | | | o |
| e3 ubiquitin-protein ligase march9-like | 1 | | | o | | | | | |
| e3 ubiquitin-protein ligase mdm2 | 1 | | | o | | | | | |
| e3 ubiquitin-protein ligase mgrn1 | 1 | | | | | | o | | |
| e3 ubiquitin-protein ligase nedd4-like | 1 | | | | | | | | o |
| e3 ubiquitin-protein ligase neurl1b-like | 1 | o | | | | | | | |
| e3 ubiquitin-protein ligase pdzrn3 isoform 1 | 1 | | | | | | | | o |
| e3 ubiquitin-protein ligase rnf167-like | 1 | | | | | | o | | |
| e3 ubiquitin-protein ligase rnf19a | 1 | | | o | | | | | |
| e3 ubiquitin-protein ligase rnf19b | 1 | | | | | | o | | |
| e3 ubiquitin-protein ligase rnf8 | 1 | | | | | | | | o |
| e3 ubiquitin-protein ligase trim33 isoform 2 | 1 | | | | o | | | | |
| e3 ubiquitin-protein ligase trim33-like | 1 | | | | | | | | o |
| e3 ubiquitin-protein ligase trim33-like isoform 2 | 1 | o | | | | | | | |
| e3 ubiquitin-protein ligase ubr3 | 1 | | | | | | o | | |
| e3 ubiquitin-protein ligase ubr4 | 1 | | o | | | | | | |
| e3 ubiquitin-protein ligase uhrf2 | 1 | | | | | | o | | |
| e4 binding protein 4-6 | 1 | | | | | | | | o |

| | | | | | | | | | |
|---|---|---|---|---|---|---|---|---|---|
| e74-like factor 1 (ets domain transcription factor) | 1 | o | | | | | | | |
| e74-like factor 2 (ets domain transcription factor) | 1 | | o | | | | | | |
| ea85_lambd ame: full=protein | 1 | | | | | | | | o |
| early estrogen-induced gene 1 protein | 1 | o | | | | | | | |
| early growth response 1-like | 1 | | | | | | | o | |
| early growth response protein 1 | 1 | | | | | | o | | |
| ebf3 partial | 1 | o | | | | | | | |
| ecdysoneless homolog | 1 | | | | | | o | | |
| echinoderm microtubule associated protein like 1 | 1 | o | | | | | | | |
| echinoderm microtubule associated protein like 2 | 1 | | | | | | o | | |
| echinoderm microtubule associated protein like 3 | 1 | o | | | | | | | |
| echinoderm microtubule associated protein like 5 | 1 | o | | | | | | | |
| echinoderm microtubule-associated 6-like | 1 | | | | | | o | | |
| ecotropic viral integration site 5 | 1 | | o | | | | | | |
| ecotropic viral integration site 5-like | 1 | | | | | | o | | |
| ecto-nox disulfide-thiol exchanger 2 | 1 | o | | | | | | | |
| ectonucleoside triphosphate diphosphohydrolase 3 | 1 | o | | | | | | | |
| ectonucleoside triphosphate diphosphohydrolase 5-like | 1 | | | | o | | | | |
| ectonucleotide pyrophosphatase phosphodiesterase 6 | 1 | o | | | | | | | |
| ectonucleotide pyrophosphatase phosphodiesterase family member 2 | 1 | | | | | | o | | |
| ectonucleotide pyrophosphatase phosphodiesterase family member 7-like | 1 | | | | o | | | | |
| edem3 protein | 1 | | o | | | | | | |
| eef1a2 binding protein | 1 | o | | | | | | | |
| ef-hand 1 | 1 | | | | | | | | o |
| ef-hand domain-containing family member a1-like | 1 | | | | o | | | | |
| eg214403 protein | 1 | | o | | | | | | |
| egf lag seven-pass g-type receptor 3 | 1 | | | | | | | o | |
| egf-containing fibulin-like extracellular matrix protein 2 | 1 | o | | | | | | | |
| egf-containing fibulin-like extracellular matrix protein isoform cra_a | 1 | o | | | | | | | |
| egf-like domain-containing protein 7 precursor | 1 | o | | | | | | | |
| egf-like- multiple 6 | 1 | o | | | | | | | |
| egf-like- multiple 7 | 1 | o | | | | | | | |
| egf-like repeats and discoidin i-like domains 3 | 1 | | | | | | o | | |
| egg envelope component zpc | 1 | | | | o | | | | |
| egg envelope glycoprotein | 1 | | | | o | | | | |
| eggshell protein | 1 | | | o | | | | | |
| eh domain binding protein 1 -like | 1 | o | | | | | | | |
| eh domain binding protein isoform 2 | 1 | | | | | | o | | |
| eh domain-binding protein 1-like isoform 3 | 1 | | o | | | | | | |
| eh-domain containing 1 | 1 | | o | | | | | | |
| eh-domain containing 3 | 1 | o | | | | | | | |
| eh-domain containing 4 | 1 | | | o | | | | | |
| eif4ba protein | 1 | o | | | | | | | |
| eif4g1 protein | 1 | | | o | | | | | |
| eif5 protein | 1 | | | | | | | | o |
| elastin microfibril interfacer 1 | 1 | | o | | | | | | |

| | | | | | | | | | |
|---|---|---|---|---|---|---|---|---|---|
| elastin microfibril interfacer 1b | 1 | | o | | | | | | |
| elastin microfibril interfacer 3a | 1 | o | | | | | | | |
| elav (embryonic abnormal drosophila)-like 2 (hu antigen b) | 1 | | | | | | o | | |
| elav (embryonic abnormal drosophila)-like 4 (hu antigen d) | 1 | | | | | | o | | |
| elav-like protein 4 | 1 | | | | | | | o | |
| elfa-a-prov protein | 1 | o | | | | | | | |
| elks rab6-interacting cast family member 1 isoform 1 | 1 | o | | | | | | | |
| elks rab6-interacting cast family member 1-like | 1 | | o | | | | | | |
| elmo domain containing 2 | 1 | | | | | | o | | |
| elongation factor mitochondrial precursor | 1 | | o | | | | | | |
| elongation of very long chain fatty acids (fen1 sur4 yeast)-like 4 | 1 | | | | | | o | | |
| elongation of very long chain fatty acids protein 1-like | 1 | | | | o | | | | |
| elongation of very long chain fatty acids protein 7-like | 1 | | | | o | | | | |
| elongator complex protein 3 | 1 | | | | | | | o | |
| embigin [Danio rerio] | 1 | | | | | | | o | |
| embigin precursor | 1 | | | | | o | | | |
| embryonic ectoderm development | 1 | o | | | | | | | |
| emopamil binding | 1 | | o | | | | | | |
| emopamil binding protein (sterol isomerase) | 1 | | | o | | | | | |
| endonuclease domain-containing 1 protein precursor | 1 | | | o | | | | | |
| endonuclease viii-like 1 | 1 | | | | o | | | | |
| endoplasmic reticulum lectin 1 isoform 1 | 1 | o | | | | | | | |
| endoplasmic reticulum lectin 1-like | 1 | | | | o | | | | |
| endoplasmic reticulum metallopeptidase 1 | 1 | o | | | | | | | |
| endoplasmic reticulum resident protein 27-like | 1 | | o | | | | | | |
| endoplasmic reticulum to nucleus signalling 1 | 1 | o | | | | | | | |
| endoplasmic reticulum-golgi intermediate compartment protein 2-like | 1 | | | | o | | | | |
| endothelial cell-specific molecule 1 | 1 | | | | | | | o | |
| endothelin converting enzyme 1 | 1 | | o | | | | | | |
| endothelin converting enzyme 2 | 1 | o | | | | | | | |
| endothelin-converting enzyme 1-like | 1 | | | | o | | | | |
| endothelin-converting enzyme 2 | 1 | | | | | | o | | |
| endothelin-converting enzyme-like 1-like | 1 | | | | | | | | o |
| engulfment adaptor ptb domain containing 1 | 1 | | | | | | o | | |
| engulfment and cell motility 2 | 1 | | | | | | | o | |
| engulfment and cell motility 3 | 1 | | o | | | | | | |
| engulfment and cell motility protein 2 | 1 | | | | | | o | | |
| engulfment and cell motility protein 2-like | 1 | | | | | o | | | |
| engulfment and cell motility protein 3-like | 1 | | o | | | | | | |
| enhancer of polycomb homolog 1 | 1 | | o | | | | | | |
| enhancer of zeste homolog 1 | 1 | o | | | | | | | |
| enolase a | 1 | | | | | | | o | |
| enolase-phosphatase e1-like | 1 | | | | o | | | | |
| enoyl- delta isomerase mitochondrial-like | 1 | | | | | | | | o |
| enoyl- hydratase | 1 | | | | o | | | | |
| enoyl- hydratase domain-containing protein mitochondrial | 1 | | o | | | | | | |
| enoyl- hydratase domain-containing protein mitochondrial-like | 1 | | | | | | | | o |
| enoyl- hydratase isomerase | 1 | | | | o | | | | |

| | | | | | | | | | |
|---|---|---|---|---|---|---|---|---|---|
| enoyl-coenzyme hydratase 3-hydroxyacyl coenzyme a dehydrogenase | 1 | | o | | | | | | |
| enterin neuropeptide | 1 | | | | | | o | | |
| envelope protein | 1 | | | o | | | | | |
| envoplakin | 1 | | | | | | | | o |
| enzymatic polyprotein endonuclease reverse | 1 | o | | | | | | | |
| eomesodermin homolog | 1 | | o | | | | | | |
| eph receptor a2 | 1 | o | | | | | | | |
| eph receptor a7 | 1 | o | | | | | | | |
| eph receptor b1 | 1 | o | | | | | | | |
| eph receptor b4a | 1 | | | o | | | | | |
| ephrin type-a receptor 3 | 1 | o | | | | | | | |
| ephrin type-b receptor 3 | 1 | | | | | | o | | |
| ephrin type-b receptor 4-like | 1 | | | | | | o | | |
| ephrin-b2 | 1 | | | | | | | o | |
| epidermal growth factor receptor pathway substrate 8 | 1 | | | | | | | o | |
| epidermal growth factor receptor substrate 15-like 1-like | 1 | | | | | | o | | |
| epimerase family protein sdr39u1 | 1 | | | | o | | | | |
| epiplakin 1 | 1 | | | | | | | o | |
| epiplakin-like protein | 1 | o | | | | | | | |
| epithelial protein lost in neoplasm beta | 1 | | o | | | | | | |
| epithelial splicing regulatory protein 2 | 1 | | o | | | | | | |
| epoxide hydrolase 2 | 1 | | | o | | | | | |
| epoxide hydrolase cytoplasmic | 1 | | | o | | | | | |
| eps8l2 protein | 1 | | | | | | | o | |
| eps8-like 3 | 1 | | o | | | | | | |
| epsilon | 1 | o | | | | | | | |
| epsilon-sarcoglycan precursor | 1 | | | | | | | o | |
| epsin 1 | 1 | | | | | | o | | |
| equilibrative nucleoside transporter 2 | 1 | | | | | | o | | |
| er degradation mannosidase alpha-like 1 | 1 | | | o | | | | | |
| er degradation mannosidase alpha-like 3 | 1 | | | | | | | o | |
| er degradation-enhancing alpha-mannosidase-like 1 | 1 | | o | | | | | | |
| er lumen protein retaining receptor 3 | 1 | o | | | | | | | |
| erbb receptor feedback inhibitor 1 | 1 | | | o | | | | | |
| erbb2ip protein | 1 | o | | | | | | | |
| erc protein 2-like | 1 | | | | | | o | | |
| erm-like protein | 1 | | | | | o | | | |
| erythrocyte membrane protein band | 1 | | | | | | o | | |
| erythrocyte membrane protein band (elliptocytosis rh-linked) | 1 | | o | | | | | | |
| erythrocyte membrane protein band -like 1 | 1 | | | | | | | o | |
| erythrocyte membrane protein band like 5 | 1 | | o | | | | | | |
| erythrocyte protein band | 1 | | | | | | | o | |
| erythrocyte protein band -like 1 | 1 | | | | | | | o | |
| erythrocyte protein band -like 3 | 1 | | | | | | o | | |
| erythrocyte protein band -like 3-like | 1 | o | | | | | | | |
| e-selectin | 1 | o | | | | | | | |
| esf1 homolog | 1 | | o | | | | | | |
| esophageal cancer related gene 4 protein | 1 | o | | | | | | | |
| esterase lipase thioesterase family protein | 1 | | o | | | | | | |
| estrogen receptor beta | 1 | o | | | | | | | |
| estrogen receptor beta 2 | 1 | | | o | | | | | |
| ethanolamine-phosphate cytidylyltransferase | 1 | | | o | | | | | |
| ets domain-containing protein | 1 | o | | | | | | | |
| ets variant 6 | 1 | o | | | | | | | |
| ets2 repressor factor | 1 | | | | | | o | | |

| | | | | | | | | | |
|---|---|---|---|---|---|---|---|---|---|
| eukaryotic initiation factor 4a | 1 | o | | | | | | | |
| eukaryotic translation elongation factor 1 alpha 1 | 1 | | | | | | | | o |
| eukaryotic translation elongation factor 1 beta 2 | 1 | | | | o | | | | |
| eukaryotic translation initiation factor 2 gamma subunit | 1 | | | | | o | | | |
| eukaryotic translation initiation factor 2 subunit 1 | 1 | | o | | | | | | |
| eukaryotic translation initiation factor 3 subunit 7 | 1 | | | | | | o | | |
| eukaryotic translation initiation factor 3 subunit a-like | 1 | | | | o | | | | |
| eukaryotic translation initiation factor 3 subunit f-like | 1 | | | | | | | | o |
| eukaryotic translation initiation factor 4 gamma 1 isoform 6 | 1 | o | | | | | | | |
| eukaryotic translation initiation factor 4 gamma 3 | 1 | | | | | | | | o |
| eukaryotic translation initiation factor 4 isoform cra_c | 1 | o | | | | | | | |
| eukaryotic translation initiation factor 4e family member 2 | 1 | o | | | | | | | |
| eukaryotic translation initiation factor 4e-binding protein 3 | 1 | | o | | | | | | |
| eukaryotic translation initiation factor 4h | 1 | | o | | | | | | |
| eukaryotic translation initiation factor subunit 2 38kda | 1 | | o | | | | | | |
| eukaryotic translation initiation factor subunit 2 39kda | 1 | o | | | | | | | |
| eukaryotic translation initiation factor subunit 4 delta | 1 | | | o | | | | | |
| eukaryotic translation initiation factor subunit 6 interacting protein | 1 | | o | | | | | | |
| eukaryotic translation initiation factor subunit 7 66 67kda | 1 | | | o | | | | | |
| eukaryotic translation initiation factor subunit a | 1 | o | | | | | | | |
| eukaryotic translation initiation factor subunit d | 1 | | | | o | | | | |
| eukaryotic translation initiation factor subunit e | 1 | o | | | | | | | |
| ewing sarcoma breakpoint region 1 | 1 | | | | | | | | o |
| ewsr1a protein | 1 | o | | | | | | | |
| excision repair cross-complementing rodent repair complementation group 5 | 1 | o | | | | | | | |
| excitatory amino acid transporter 3 | 1 | | | | | | | o | |
| excitatory amino acid transporter 4 | 1 | | | | | | o | | |
| exocyst complex component 1 | 1 | | | | | o | | | |
| exocyst complex component 1 isoform 2 | 1 | | | | | | | | o |
| exocyst complex component 6 | 1 | | | | | | o | | |
| exodeoxyribonuclease iii family protein | 1 | | | | | | o | | |
| exonuclease 3 -5 domain-containing protein 1-like | 1 | | | | o | | | | |
| exonuclease 3 -5 domain-containing protein 2 | 1 | | o | | | | | | |
| exosomal core protein csl4 | 1 | | | | o | | | | |
| exosome complex exonuclease rrp40 | 1 | o | | | | | | | |
| exosome component 4 | 1 | | | | o | | | | |
| exosome component 9 | 1 | | o | | | | | | |
| exostoses 1c | 1 | | | | | | | | o |

| | | | | | | | | | |
|---|---|---|---|---|---|---|---|---|---|
| exostoses -like 3 | 1 | o | | | | | | | |
| exostosin 2 | 1 | o | | | | | | | |
| exportin 7 | 1 | | | | | | o | | |
| extended synaptotagmin-like protein 1a | 1 | | | | | | | o | |
| extracellular calcium-sensing receptor-like | 1 | | | | o | | | | |
| extracellular leucine-rich repeat and fibronectin type-iii domain-containing protein 1-like | 1 | | | | | | o | | |
| extracellular matrix protein female organ and adipocyte specific | 1 | o | | | | | | | |
| eyes absent 4 | 1 | o | | | | | | | |
| eyes absent homolog 3 | 1 | o | | | | | | | |
| eyes absent homolog 4 | 1 | o | | | | | | | |
| ezrin | 1 | | | | o | | | | |
| ezrin like | 1 | | o | | | | | | |
| ezrin-like isoform 2 | 1 | | o | | | | | | |
| f11 receptor | 1 | | o | | | | | | |
| f110b_danre ame: full=protein fam110b | 1 | | | | | | o | | |
| f1712_xenla ame: full=protein fam171a2 flags: precursor | 1 | | | | | | | o | |
| fa83h_danre ame: full=protein fam83h | 1 | o | | | | | | | |
| fact complex subunit spt16 | 1 | o | | | | | | | |
| f-actin-capping protein subunit beta | 1 | o | | | | | | | |
| factor b c2b | 1 | | | | o | | | | |
| fad binding domain protein | 1 | o | | | | | | | |
| fad dependent oxidoreductase | 1 | o | | | | | | | |
| fad synthase | 1 | | | | | | | | o |
| fad1 flavin adenine dinucleotide synthetase homolog ( cerevisiae) | 1 | | o | | | | | | |
| fad-dependent oxidoreductase domain-containing protein 1-like | 1 | | | | | | o | | |
| fad-linked sulfhydryl oxidase alr | 1 | o | | | | | | | |
| fam107b protein | 1 | o | | | | | | | |
| fam179b protein | 1 | | | | | | o | | |
| fam21c protein | 1 | o | | | | | | | |
| fam83d protein | 1 | o | | | | | | | |
| fam83h protein | 1 | o | | | | | | | |
| family member 5 | 1 | | | | | | | o | |
| family protein | 1 | o | | | | | | | |
| family with sequence similarity 122a | 1 | o | | | | | | | |
| family with sequence similarity 19 (chemokine (c-c motif)-like) member a2 | 1 | | | | | o | | | |
| family with sequence similarity 19 (chemokine (c-c motif)-like) member a3 | 1 | | | | | o | | | |
| family with sequence similarity member a2 | 1 | | o | | | | | | |
| family with sequence similarity member e | 1 | o | | | | | | | |
| family with sequence similarity member h | 1 | | | o | | | | | |
| fanconi complementation group d2 | 1 | o | | | | | | | |
| fanconi complementation group e | 1 | | | | o | | | | |
| far upstream element-binding protein 2-like | 1 | | | | | | | | o |
| far upstream element-binding protein 3 | 1 | o | | | | | | | |
| farnesyl diphosphate synthase | 1 | o | | | | | | | |
| fas -associated via death domain | 1 | | o | | | | | | |
| fas-activated serine threonine kinase | 1 | | o | | | | | | |
| fasciculation and elongation protein zeta 2 (zygin ii) | 1 | | | | | | o | | |
| fascin | 1 | o | | | | | | | |
| fascin homolog actin-bundling protein (strongylocentrotus purpuratus) | 1 | o | | | | | | | |
| fast kinase domain-containing protein 1 | 1 | | | | | o | | | |

| | | | | | | | | | |
|---|---|---|---|---|---|---|---|---|---|
| fast kinase domain-containing protein 3-like | 1 | | | | o | | | | |
| fast kinase domains 5 | 1 | | | | | o | | | |
| fast myosin heavy chain hcii | 1 | o | | | | | | | |
| fast myotomal muscle tropomyosin | 1 | | | | o | | | | |
| fast myotomal muscle troponin-t-2 | 1 | o | | | | | | | |
| fat- partial | 1 | o | | | | | | | |
| fat storage-inducing transmembrane protein 2 | 1 | o | | | | | | | |
| fat tumor suppressor 2-like | 1 | | | | | | | o | |
| fatty acid amide hydrolase 2 | 1 | | o | | | | | | |
| fatty acid binding protein h6-isoform | 1 | | | | o | | | | |
| fatty acid delta-6 desaturase | 1 | | | | | o | | | |
| fatty oxidation complex alpha subunit | 1 | o | | | | | | | |
| f-box and leucine-rich repeat protein 18 | 1 | | | | | | | o | |
| f-box and leucine-rich repeat protein 22 | 1 | o | | | | | | | |
| f-box lrr-repeat protein 14-like | 1 | | | | o | | | | |
| f-box lrr-repeat protein 18 | 1 | | | | | | | | o |
| f-box lrr-repeat protein 20 | 1 | | | | | | o | | |
| f-box lrr-repeat protein 20-like | 1 | o | | | | | | | |
| f-box only protein 11-like | 1 | | | | | | o | | |
| f-box only protein 18 | 1 | o | | | | | | | |
| f-box only protein 22 | 1 | | o | | | | | | |
| f-box only protein 25 | 1 | o | | | | | | | |
| f-box only protein 2-like | 1 | | | | | | | | o |
| f-box only protein 32 | 1 | o | | | | | | | |
| f-box only protein 39-like | 1 | o | | | | | | | |
| f-box only protein 43 | 1 | | | | o | | | | |
| f-box only protein 47 | 1 | | | | o | | | | |
| f-box only protein 9 | 1 | | | o | | | | | |
| f-box protein 11 | 1 | o | | | | | | | |
| f-box protein 21 | 1 | o | | | | | | | |
| f-box protein 40 | 1 | o | | | | | | | |
| f-box protein 41 | 1 | | | | | | o | | |
| f-box protein 42 | 1 | | o | | | | | | |
| f-box protein 44 | 1 | | o | | | | | | |
| f-box spry domain-containing protein 1 | 1 | | | | | | | | o |
| f-box wd repeat-containing protein 12-like | 1 | | o | | | | | | |
| f-box wd repeat-containing protein 9-like | 1 | | | | o | | | | |
| fbp32 precursor | 1 | | | | | | | o | |
| fc fragment of binding protein | 1 | | | | o | | | | |
| fc receptor-like 2-like | 1 | o | | | | | | | |
| fc receptor-like 3 | 1 | o | | | | | | | |
| fc receptor-like 5 | 1 | o | | | | | | | |
| fc receptor-like protein 5-like | 1 | | o | | | | | | |
| fcgbp protein | 1 | | | | o | | | | |
| fch domain only protein 2 | 1 | o | | | | | | | |
| fdps protein | 1 | o | | | | | | | |
| feline leukemia virus subgroup c cellular receptor member 2 | 1 | | | o | | | | | |
| feline leukemia virus subgroup c receptor-related protein 2 | 1 | | o | | | | | | |
| fem-1 homolog a ( elegans) | 1 | o | | | | | | | |
| fer1l3 protein | 1 | o | | | | | | | |
| fer-1-like myoferlin ( elegans) | 1 | o | | | | | | | |
| ferm and pdz domain containing 4 | 1 | o | | | | | | | |
| ferm and pdz domain-containing protein 3-like | 1 | | | | | | o | | |
| ferm and pdz domain-containing protein 4-like | 1 | | | | o | | | | |
| fermitin family homolog 3 | 1 | o | | | | | | | |

| | | | | | | | | | |
|---|---|---|---|---|---|---|---|---|---|
| ferredoxin-fold anticodon-binding domain-containing protein 1-like | 1 | | | | o | | | | |
| ferritin heavy chain | 1 | | | | | | | | o |
| fetuin-b-like | 1 | | | | o | | | | |
| fggy carbohydrate kinase domain containing | 1 | | o | | | | | | |
| fibrinogen-like protein 1-like | 1 | o | | | | | | | |
| fibrinolytic enzyme | 1 | o | | | | | | | |
| fibroblast growth factor 1 | 1 | o | | | | | | | |
| fibroblast growth factor 16 | 1 | o | | | | | | | |
| fibroblast growth factor 19 | 1 | | o | | | | | | |
| fibroblast growth factor 6 | 1 | o | | | | | | | |
| fibroblast growth factor binding protein 1 | 1 | | | | | o | | | |
| fibroblast growth factor-binding protein 2 precursor | 1 | | | | | o | | | |
| fibronectin | 1 | | | | o | | | | |
| fibronectin isoform cra_d | 1 | | o | | | | | | |
| fibronectin leucine rich transmembrane protein 2 | 1 | | | | | | | o | |
| fibronectin type iii and spry domain-containing protein 2-like | 1 | | | | o | | | | |
| fibronectin type iii domain containing 3a | 1 | | | o | | | | | |
| fibronectin type iii domain containing 4 | 1 | | | | | | | o | |
| fibronectin type iii domain protein | 1 | o | | | | | | | |
| fibronectin type-iii domain-containing protein c4orf31 homolog precursor | 1 | | | | | | | o | |
| fibronectin type-iii domain-containing protein c4orf31-like | 1 | | | | | o | | | |
| fibulin 1 | 1 | | | | | | o | | |
| fibulin 5-like | 1 | o | | | | | | | |
| fidgetin | 1 | | | | | | o | | |
| filamin gamma | 1 | o | | | | | | | |
| filamin-c-like isoform 4 | 1 | o | | | | | | | |
| fizzy cell division cycle 20 related 1 | 1 | | o | | | | | | |
| fk506 binding | 1 | o | | | | | | | |
| fk506 binding protein 38kda | 1 | | o | | | | | | |
| fk506 binding protein 63 kda | 1 | o | | | | | | | |
| fk506 binding protein 7 | 1 | o | | | | | | | |
| fk506 binding protein 8 | 1 | | o | | | | | | |
| fk506 binding protein like | 1 | | | | | o | | | |
| fKIAA0680 [Takifugu rubripes] | 1 | o | | | | | | | |
| flap endonuclease gen homolog 1-like | 1 | | | | o | | | | |
| flap structure-specific endonuclease 1 | 1 | o | | | | | | | |
| flavin-containing monooxygenase fmo1 | 1 | | | | | | o | | |
| flavoprotein oxidoreductase-like | 1 | o | | | | | | | |
| flj25076 protein | 1 | | | | | | o | | |
| flj46154 protein | 1 | | | | | | | o | |
| flnc protein | 1 | o | | | | | | | |
| fmnl1 protein | 1 | o | | | | | | | |
| fms-like tyrosine kinase 4 | 1 | | o | | | | | | |
| fms-related tyrosine kinase 1 (vascular endothelial growth factor vascular permeability factor receptor) | 1 | o | | | | | | | |
| fms-related tyrosine kinase 3 | 1 | o | | | | | | | |
| folate hydrolase | 1 | o | | | | | | | |
| follistatin-like 5 isoform 1 | 1 | | | | | | o | | |
| follistatin-like 5 isoform 2 | 1 | | | | | | o | | |
| follistatin-related protein 5 isoform 2 | 1 | | | | | | o | | |
| folylpolyglutamate mitochondrial | 1 | o | | | | | | | |
| forkhead box d2 | 1 | | | | o | | | | |
| forkhead box f1 | 1 | o | | | | | | | |

| | | | | | | | | | |
|---|---|---|---|---|---|---|---|---|---|
| forkhead box h1 | 1 | | | | o | | | | |
| forkhead box j1b | 1 | | | | o | | | | |
| forkhead box o5 | 1 | o | | | | | | | |
| forkhead box p1 | 1 | o | | | | | | | |
| forkhead box p4 | 1 | o | | | | | | | |
| forkhead box protein j2 | 1 | o | | | | | | | |
| forkhead box protein j2-like | 1 | | | | | | | | o |
| forkhead box protein k1-like | 1 | | | | | | | | o |
| forkhead box protein p1-b | 1 | | | | | | | o | |
| formation of mitochondrial complexes 1 homolog | 1 | | o | | | | | | |
| formiminotransferase cyclodeaminase-like | 1 | | o | | | | | | |
| formin homology 2 domain containing 3 | 1 | o | | | | | | | |
| formin-like 1 | 1 | o | | | | | | | |
| formin-like 2 | 1 | o | | | | | | | |
| formin-like protein 2-like | 1 | o | | | | | | | |
| forty-two-three domain containing 1 | 1 | o | | | | | | | |
| four jointed box 1 | 1 | | | | | | | | o |
| four-jointed box protein 1-like | 1 | | | | | | | | o |
| fox-1 homolog c-like | 1 | | o | | | | | | |
| fra10ac1 protein | 1 | o | | | | | | | |
| fragile x mental autosomal homolog 2 | 1 | o | | | | | | | |
| fragile x mental retardation syndrome-related protein 1 | 1 | o | | | | | | | |
| fras1-related extracellular matrix protein 3 | 1 | o | | | | | | | |
| friend leukemia integration 1 | 1 | o | | | | | | | |
| frizzled homolog 2 | 1 | o | | | | | | | |
| frizzled homolog 6 | 1 | o | | | | | | | |
| frizzled homolog 8 | 1 | | | | | | o | | |
| fructosamine 3 kinase related protein | 1 | o | | | | | | | |
| fructosamine-3-kinase-related protein | 1 | | | | | o | | | |
| fructose- -bisphosphatase 2 | 1 | o | | | | | | | |
| fructose-bisphosphate aldolase a | 1 | o | | | | | | | |
| fumarylacetoacetase | 1 | | o | | | | | | |
| fun14 domain-containing protein 1-like | 1 | | | | | | | | o |
| fungal transcriptional regulatory n-terminal domain-containing protein | 1 | | | | | | | o | |
| furin endoprotease | 1 | | | o | | | | | |
| furin precursor | 1 | | | o | | | | | |
| fus interacting protein (serine arginine-rich) 1 | 1 | | o | | | | | | |
| fuse-binding protein-interacting repressor | 1 | o | | | | | | | |
| fxyd domain containing ion transport regulator 5a-like | 1 | o | | | | | | | |
| fxyd domain containing ion transport regulator 6 | 1 | | | | | o | | | |
| fxyd domain containing ion transport regulator 7 | 1 | o | | | | | | | |
| fxyd domain-containing ion transport regulator 6 | 1 | | | | | | o | | |
| fxyd domain-containing ion transport regulator 7 | 1 | | | | | | | o | |
| fyve and coiled-coil domain containing 1 | 1 | o | | | | | | | |
| g alpha s olf-2 protein | 1 | | | | | | o | | |
| g patch domain-containing protein 4 | 1 | o | | | | | | | |
| g protein alpha subunit | 1 | | | | o | | | | |
| g protein-activated inward rectifier potassium channel 2 | 1 | | | | | | | | o |
| g protein-coupled family group member b | 1 | | | | | | | o | |
| g protein-coupled family group member c | 1 | | | | | | o | | |
| g protein-coupled receptor 1 | 1 | o | | | | | | | |

| | | | | | | | | | |
|---|---|---|---|---|---|---|---|---|---|
| g protein-coupled receptor 107 | 1 | o | | | | | | | |
| g protein-coupled receptor 109a | 1 | o | | | | | | | |
| g protein-coupled receptor 151 | 1 | | | | | o | | | |
| g protein-coupled receptor 175 | 1 | | | o | | | | | |
| g protein-coupled receptor 31 | 1 | | | | | | | o | |
| g protein-coupled receptor 37 like 1 | 1 | | | | | | o | | |
| g protein-coupled receptor 6 | 1 | | | | | | o | | |
| g protein-coupled receptor 75 | 1 | | | | | o | | | |
| g protein-coupled receptor kinase 4 | 1 | o | | | | | | | |
| g t mismatch-specific thymine dna glycosylase | 1 | o | | | | | | | |
| g2 m phase-specific e3 ubiquitin-protein ligase-like | 1 | | | | o | | | | |
| g2 mitotic-specific cyclin-b2-like | 1 | | | | o | | | | |
| g2 m-phase specific e3 ubiquitin ligase | 1 | | o | | | | | | |
| ga binding protein transcription alpha subunit 60kda | 1 | o | | | | | | | |
| gabaa receptor gamma 3 subunit | 1 | | | | | | o | | |
| gag protein | 1 | | | | | o | | | |
| galactokinase 1 | 1 | | o | | | | | | |
| galactokinase 2 | 1 | | | | | | | | o |
| galactosylgalactosylxylosylprotein 3-beta-glucuronosyltransferase 2 | 1 | | | | | | | | o |
| galectin 8 | 1 | | | | | | | | o |
| galectin-3 | 1 | o | | | | | | | |
| galectin-8 | 1 | | o | | | | | | |
| gametogenetin-binding protein 2-like | 1 | | | | o | | | | |
| gamma 3 | 1 | | | | | | | o | |
| gamma complex associated protein 2 | 1 | o | | | | | | | |
| gamma complex associated protein 4 | 1 | | | | | | | | o |
| gamma fibrinogen | 1 | | o | | | | | | |
| gamma-aminobutyric acid (gaba-a) subunit alpha 3 | 1 | | | | | | | o | |
| gamma-aminobutyric acid (gaba-a) subunit beta 2 | 1 | | | | | | | | o |
| gamma-aminobutyric acid a alpha 1 | 1 | | | | | o | | | |
| gamma-aminobutyric acid a alpha 4 | 1 | | | | | | o | | |
| gamma-aminobutyric acid a alpha 6 | 1 | | | | | | | o | |
| gamma-aminobutyric acid a beta 1 | 1 | | | | | | o | | |
| gamma-aminobutyric acid a gamma 2 | 1 | | | | | | o | | |
| gamma-aminobutyric acid a pi | 1 | | | | | | o | | |
| gamma-aminobutyric acid receptor subunit alpha-6 | 1 | | | | | | | | o |
| gamma-aminobutyric acid receptor subunit delta precursor (gaba receptor subunit delta) | 1 | | | | | o | | | |
| gamma-aminobutyric acid receptor subunit gamma-2 precursor | 1 | | | | | | | o | |
| gamma-crystallin m1-1 | 1 | o | | | | | | | |
| gamma-glutamyl hydrolase precursor | 1 | o | | | | | | | |
| gamma-glutamylcyclotransferase precursor | 1 | | | | | | | | o |
| gamma-glutamyltransferase ywrd-like | 1 | | o | | | | | | |
| gamma-sarcoglycan | 1 | o | | | | | | | |
| gamma-tubulin complex component 5 | 1 | | | | o | | | | |
| ganglioside-induced differentiation-associated protein 2 | 1 | | | | | | | o | |
| gap junction alpha 46kda | 1 | o | | | | | | | |
| gap junction delta-2 | 1 | | | | | | o | | |
| gap junction protein beta 2 | 1 | o | | | | | | | |
| garnl1 protein | 1 | o | | | | | | | |
| gastric intrinsic factor-like | 1 | | | | | o | | | |

| | | | | | | | | | |
|---|---|---|---|---|---|---|---|---|---|
| gastrulation brain homeobox 1 | 1 | o | | | | | | | |
| gata-binding factor 2 | 1 | o | | | | | | | |
| gcat protein | 1 | | | o | | | | | |
| gc-rich sequence dna-binding factor | 1 | | o | | | | | | |
| gc-rich sequence dna-binding factor homolog | 1 | o | | | | | | | |
| gdnf family receptor alpha-4-like | 1 | | | | | | o | | |
| gdnf receptor alpha | 1 | | | | | | o | | |
| gdp dissociation inhibitor 1 | 1 | | o | | | | | | |
| gdp-l-fucose synthetase | 1 | | | | | | o | | |
| gdp-mannose dehydratase | 1 | | | | | o | | | |
| gem-associated protein 5 | 1 | | | | | | | | o |
| general transcription factor 3c polypeptide 4 | 1 | | | | o | | | | |
| general transcription factor iib | 1 | o | | | | | | | |
| general transcription factor iie subunit 1 | 1 | | | | o | | | | |
| general transcription factor iih subunit 1 | 1 | o | | | | | | | |
| general transcription factor polypeptide 34kda | 1 | o | | | | | | | |
| general transcription factor polypeptide 52kda | 1 | | | | | | | | o |
| general transcription factor polypeptide alpha 56kda | 1 | o | | | | | | | |
| general vesicular transport factor p115 | 1 | | o | | | | | | |
| genetic suppressor element 1-like | 1 | | | | | | o | | |
| gephyrin1 isoform | 1 | | | | | | o | | |
| geranylgeranyl diphosphate synthase 1 | 1 | | | o | | | | | |
| germinal histone h4 gene | 1 | | | o | | | | | |
| GF10854 [Drosophila ananassae] | 1 | | | | | | | o | |
| gfm2 protein | 1 | | o | | | | | | |
| GG24640 [Drosophila erecta] | 1 | | | | o | | | | |
| ghmp kinases atp-binding expressed | 1 | | o | | | | | | |
| ghrh pacap precursor | 1 | | | | | | | o | |
| GI14934 [Drosophila mojavensis] | 1 | o | | | | | | | |
| GI15861 [Drosophila mojavensis] | 1 | | | | | | | | o |
| gipc pdz domain containing member 1 | 1 | o | | | | | | | |
| GL13991 [Drosophila persimilis] | 1 | | | | | o | | | |
| glia maturation gamma | 1 | o | | | | | | | |
| glioma tumor suppressor candidate region gene 1 | 1 | | | | | | o | | |
| glis family zinc finger 2 | 1 | o | | | | | | | |
| globoside alpha- -n-acetylgalactosaminyltransferase 1 | 1 | | | o | | | | | |
| glomulin | 1 | | | | o | | | | |
| glomulin isoform 1 | 1 | | | | o | | | | |
| glucagon receptor precursor | 1 | | o | | | | | | |
| glucan ( -alpha-) branching enzyme 1 | 1 | | | o | | | | | |
| glucocorticoid modulatory element binding protein 1 | 1 | | | | | | o | | |
| glucocorticoid receptor dna binding factor 1 | 1 | o | | | | | | | |
| glucosamine-6-phosphate isomerase 2 | 1 | o | | | | | | | |
| glucosamine--fructose-6-phosphate aminotransferase | 1 | | o | | | | | | |
| glucose phosphate isomerase a | 1 | | | | | | | | o |
| glucose-6- 2 | 1 | | o | | | | | | |
| glucose-6-phosphatase 3 | 1 | | | o | | | | | |
| glucose-6-phosphate 1-dehydrogenase | 1 | | o | | | | | | |
| glucose-6-phosphate dehydrogenase | 1 | | | | | | o | | |
| glucoside xylosyltransferase 1 | 1 | o | | | | | | | |
| glucosylceramidase precursor | 1 | | o | | | | | | |
| glutamate decarboxylase 1 | 1 | | | | | | o | | |
| glutamate decarboxylase 2 | 1 | | | | | | | o | |

| | | | | | | | | | |
|---|---|---|---|---|---|---|---|---|---|
| glutamate delta 2 | 1 | | | | | | o | | |
| glutamate ionotropic kainate 2-like isoform 2 | 1 | | | | | | o | | |
| glutamate kainate 2 | 1 | | | | | | o | | |
| glutamate metabotropic 4 | 1 | o | | | | | | | |
| glutamate metabotropic 7 | 1 | o | | | | | | | |
| glutamate metabotropic 7-like | 1 | | | | | | | o | |
| glutamate metabotropic 8 precursor | 1 | | | | | o | | | |
| glutamate n-methyl d-asparate-associated protein 1 (glutamate binding) isoform cra_a | 1 | | | | | | | o | |
| glutamate n-methyl d-aspartate 1 | 1 | | | | | | o | | |
| glutamate n-methyl d-aspartate 2a | 1 | o | | | | | | | |
| glutamate receptor 2 isoform 2 precursor | 1 | | | | | | | o | |
| glutamate receptor 4 isoform 1 precursor | 1 | | | | | | o | | |
| glutamate receptor interacting protein 2 | 1 | o | | | | | | | |
| glutamate receptor ionotropic n-methyl d-aspartate 2a | 1 | | | | | | o | | |
| glutamate receptor u1-like | 1 | | | | | | | | o |
| glutamate--cysteine ligase catalytic subunit | 1 | | | | | | | | o |
| glutamic pyruvate transaminase (alanine aminotransferase) 2 | 1 | o | | | | | | | |
| glutaminase kidney mitochondrial-like | 1 | | | | | | | | o |
| glutamine and serine-rich protein 1 | 1 | | | | | | | o | |
| glutamine-asparagine rich protein | 1 | | | | | o | | | |
| glutamine-dependent nad(+) synthetase | 1 | | | | o | | | | |
| glutamine-dependent nad(+) synthetase-like | 1 | | o | | | | | | |
| glutamine-rich 1 | 1 | o | | | | | | | |
| glutaminyl cyclase | 1 | | | o | | | | | |
| glutaminyl-peptide cyclotransferase-like | 1 | | | | o | | | | |
| glutamyl-prolyl trna synthetase isoform 2 | 1 | o | | | | | | | |
| glutamyl-trna amidotransferase subunit a homolog | 1 | | o | | | | | | |
| glutamyl-trna amidotransferase-like | 1 | | | | | | o | | |
| glutaredoxin | 1 | | | o | | | | | |
| glutaredoxin 5 | 1 | | | | | o | | | |
| glutathione peroxidase 6 | 1 | | o | | | | | | |
| glutathione s-transferase c-terminal domain-containing protein | 1 | | | | | | | o | |
| glutathione synthetase | 1 | o | | | | | | | |
| glutathione transferase zeta 1 | 1 | | | | | o | | | |
| glyceraldehyde 3-phosphate dehydrogenase | 1 | | o | | | | | | |
| glycerol-3-phosphate acyltransferase 6 | 1 | o | | | | | | | |
| glycerol-3-phosphate dehydrogenase 1 | 1 | o | | | | | | | |
| glycerol-3-phosphate dehydrogenase 1-like protein | 1 | o | | | | | | | |
| glycerol-3-phosphate isoform cra_a | 1 | o | | | | | | | |
| glycerol-3-phosphate mitochondrial | 1 | o | | | | | | | |
| glycerophosphodiester phosphodiesterase 1 | 1 | o | | | | | | | |
| glycerophosphodiester phosphodiesterase domain containing 2 | 1 | | | | | o | | | |
| glycine amidinotransferase (l-arginine:glycine amidinotransferase) | 1 | o | | | | | | | |
| glycine- glutamate-thienylcyclohexylpiperidine-binding | 1 | | | | | | | o | |
| glycine n-acyltransferase | 1 | o | | | | | | | |
| glycine n-acyltransferase-like protein 3-like | 1 | | | | o | | | | |
| glycine receptor subunit alpha-2-like isoform 2 | 1 | | | | | | o | | |
| glycine receptor subunit beta isoform 1 | 1 | | | | | | o | | |
| glycine-rich repeat-containing protein | 1 | | o | | | | | | |
| glycogen liver form | 1 | | o | | | | | | |

| | | | | | | | | | |
|---|---|---|---|---|---|---|---|---|---|
| glycogen synthase 1 | 1 | o | | | | | | | |
| glycolipid transfer protein domain-containing protein 1 | 1 | | | o | | | | | |
| glycoprotein | 1 | | | o | | | | | |
| glycoprotein 350 220 | 1 | | | o | | | | | |
| glycoprotein 9 | 1 | o | | | | | | | |
| glycoprotein a repetitions predominant precursor | 1 | o | | | | | | | |
| glycoprotein v-like | 1 | | | o | | | | | |
| glycoside hydrolase family protein | 1 | | | | | | o | | |
| glycosylphosphatidylinositol anchor attachment 1 | 1 | | | | | | | | o |
| glycosyltransferase 1 domain containing 1 | 1 | o | | | | | | | |
| glycosyltransferase 25 domain containing 1 | 1 | o | | | | | | | |
| glycosyltransferase 25 domain containing 2 | 1 | o | | | | | | | |
| glycosyltransferase 8 domain-containing protein 1 | 1 | o | | | | | | | |
| glyoxylate reductase-like | 1 | | | | | | | | o |
| glypican 1 | 1 | o | | | | | | | |
| glypican 3 | 1 | | | | | | o | | |
| glypican 5 | 1 | o | | | | | | | |
| glypican 6 | 1 | o | | | | | | | |
| gm2 ganglioside activator protein | 1 | | | o | | | | | |
| gmp reductase | 1 | o | | | | | | | |
| golgi golgin subfamily 1 | 1 | | o | | | | | | |
| golgi golgin subfamily macrogolgin (with transmembrane signal) 1 | 1 | o | | | | | | | |
| golgi golgin subfamily macrogolgin 1-like | 1 | o | | | | | | | |
| golgi reassembly stacking protein 2 | 1 | o | | | | | | | |
| golgi-associated plant pathogenesis-related protein 1 | 1 | | | | | | | | o |
| gon-4-like ( elegans) | 1 | | | | | | o | | |
| gpbp-interacting protein 130b | 1 | | | | | | | o | |
| gpi ethanolamine phosphate transferase 2-like | 1 | | o | | | | | | |
| gpi inositol-deacylase | 1 | | | | | | o | | |
| gpi mannosyltransferase 2 | 1 | | | | o | | | | |
| gpi transamidase component pig-s-like | 1 | | | | | | | | o |
| gpi-anchor transamidase-like | 1 | | | | | | | | o |
| g-protein coupled receptor 183 | 1 | o | | | | | | | |
| g-protein coupled receptor 98 | 1 | | | | | | | | o |
| g-protein coupled receptor 98 precursor | 1 | | | | | | | | o |
| g-protein coupled receptor 98-like | 1 | o | | | | | | | |
| g-protein coupled receptor family c group 5 member c-like | 1 | | | | o | | | | |
| g-protein coupled receptor-associated sorting protein 2 | 1 | | | | o | | | | |
| g-protein signalling modulator 1 (ags3-elegans) isoform cra_c | 1 | o | | | | | | | |
| g-protein signalling modulator 1 (ags3-like elegans) | 1 | | | o | | | | | |
| g-protein-signaling modulator 2-like | 1 | | o | | | | | | |
| gpx5 protein | 1 | o | | | | | | | |
| grainyhead-like 1 | 1 | | | o | | | | | |
| gram domain containing 2 | 1 | | o | | | | | | |
| gram domain containing isoform cra_e | 1 | o | | | | | | | |
| gram domain-containing protein 3 isoform 1 | 1 | | | | | | | | o |
| gramd2 protein | 1 | | | | | o | | | |
| granulin 1 | 1 | | | | | | | | o |
| granulocyte colony-stimulating factor receptor precursor | 1 | o | | | | | | | |

| | | | | | | | | | |
|---|---|---|---|---|---|---|---|---|---|
| grb2-related adaptor protein | 1 | o | | | | | | | |
| grb2-related adaptor protein 2 | 1 | | | o | | | | | |
| grn protein | 1 | o | | | | | | | |
| growth arrest and dna-damage- gamma interacting protein 1 | 1 | o | | | | | | | |
| growth arrest and dna-damage-inducible protein gadd45 alpha | 1 | | | | | | | | o |
| growth arrest-specific protein 2 | 1 | o | | | | | | | |
| growth differentiation factor 15 | 1 | | | | | | | o | |
| growth factor receptor bound protein 2-associated | 1 | o | | | | | | | |
| growth factor receptor-bound protein 10 | 1 | | | o | | | | | |
| growth hormone-releasing hormone pitutary adenylate cyclase-activating protein | 1 | | | | | | | o | |
| growth hormone-releasing hormone receptor | 1 | | | | | | o | | |
| growth-hormone releasing hormone-like peptide receptor | 1 | o | | | | | | | |
| gsk-3-binding protein | 1 | | | | | | | | o |
| gtp binding protein 2 | 1 | | | o | | | | | |
| gtp cyclohydrolase 2 | 1 | | o | | | | | | |
| gtpase activating protein (sh3 domain) binding protein 1 | 1 | o | | | | | | | |
| gtpase imap family member partial | 1 | | | | o | | | | |
| gtpase kras-like | 1 | | o | | | | | | |
| gtpase regulator associated with the focal adhesion kinase pp125 | 1 | o | | | | | | | |
| gtpase-like protein | 1 | | | o | | | | | |
| gtp-binding protein gem-like | 1 | | | | | | | | o |
| gtp-binding protein rheb-like | 1 | | | | | | | o | |
| gtp-binding protein rhes-like | 1 | | | | o | | | | |
| guanine monphosphate synthetase | 1 | o | | | | | | | |
| guanine nucleotide binding 1 | 1 | | | | o | | | | |
| guanine nucleotide binding 3 -like | 1 | | | | | | | o | |
| guanine nucleotide binding alpha transducing [illegible] | 1 | | | | o | | | | |
| guanine nucleotide binding protein (g protein) alpha inhibiting activity polypeptide 1 | 1 | | | o | | | | | |
| guanine nucleotide binding protein (g protein) beta 5 | 1 | | | o | | | | | |
| guanine nucleotide binding protein (g protein) beta polypeptide like | 1 | | | | | | | o | |
| guanine nucleotide binding protein (g protein) gamma 2 | 1 | o | | | | | | | |
| guanine nucleotide binding protein (g protein) gamma 3 | 1 | | | | | | o | | |
| guanine nucleotide exchange | 1 | | | | | | | o | |
| guanine nucleotide-binding protein g g g subunit beta-1 | 1 | o | | | | | | | |
| guanylate cyclase alpha 2 | 1 | | | | | | | o | |
| guanylate kinase 1 | 1 | | | | | | | o | |
| h aca ribonucleoprotein complex subunit 1 | 1 | | | | | | | | o |
| h aca ribonucleoprotein complex subunit 4 | 1 | | | | | | o | | |
| h+ lysosomal v0 subunit a1 isoform 1 | 1 | | | | | | o | | |
| h+ lysosomal v0 subunit isoform cra_c | 1 | | | | | | | o | |
| hairy and enhancer of split 6 | 1 | | | | | | | | o |
| hairy and enhancer of split gene 2 | 1 | | | | | o | | | |
| hairy enhancer-of-split related with yrpw motif 1 | 1 | | | | | | o | | |
| haloacid dehalogenase-like hydrolase domain containing 1a | 1 | o | | | | | | | |

| | | | | | | | | | |
|---|---|---|---|---|---|---|---|---|---|
| haloacid dehalogenase-like hydrolase domain-containing protein 2-like | 1 | o | | | | | | | |
| haptoglobin | 1 | | | | o | | | | |
| haptoglobin fragment 1 | 1 | | | | o | | | | |
| haptoglobin-related protein | 1 | | o | | | | | | |
| hat family dimerisation domain containing expressed | 1 | o | | | | | | | |
| hat family dimerisation domain containing partial | 1 | | | | | | | o | |
| hat family dimerization domain protein | 1 | o | | | | | | | |
| haus augmin-like complex subunit 2-like | 1 | | | | | | | | o |
| haus augmin-like complex subunit 4 | 1 | o | | | | | | | |
| haus augmin-like complex subunit 6 | 1 | | | | o | | | | |
| hbs1-like protein isoform 3 | 1 | o | | | | | | | |
| hcg30195-like isoform 2 | 1 | | | | | | o | | |
| hcls1-associated protein x-1 | 1 | | o | | | | | | |
| hcp beta-lactamase-like protein c1orf163 homolog | 1 | o | | | | | | | |
| hd domain-containing protein 2 | 1 | o | | | | | | | |
| hd_fugru ame: full=huntingtin ame: full=huntington disease protein homolog short=hd protein homolog | 1 | | | | | | o | | |
| hdgr2_danre ame: full=hepatoma-derived growth factor-related protein 2 short=hrp-2 | 1 | | | | | | o | | |
| hdlbp protein | 1 | o | | | | | | | |
| headcase protein homolog | 1 | | | | | | | | o |
| heat repeat containing 6 | 1 | | | o | | | | | |
| heat repeat-containing protein 5b | 1 | | | | | | | o | |
| heat repeat-containing protein 7a | 1 | | o | | | | | | |
| heat shock 22kda protein 8 | 1 | o | | | | | | | |
| heat shock 27kda protein member 7 | 1 | o | | | | | | | |
| heat shock 70 kda protein 14-like | 1 | | | | o | | | | |
| heat shock cognate 71 kda protein | 1 | | | | | | o | | |
| heat shock cognate protein 70 | 1 | | o | | | | | | |
| heat shock protein | 1 | | | | o | | | | |
| heat shock protein 30 | 1 | o | | | | | | | |
| heat shock protein 40 | 1 | | o | | | | | | |
| heat shock protein 8 | 1 | | | | | | o | | |
| heat shock protein 90kda alpha class a member 1 | 1 | o | | | | | | | |
| heat shock protein hsp 90-alpha-like | 1 | | | | | | | | o |
| heat shock protein hsp 90-beta | 1 | | | | | | | o | |
| heat shock protein isoform cra_b | 1 | o | | | | | | | |
| heat shock transcription factor 1 | 1 | o | | | | | | | |
| heat-shock protein 70 | 1 | | | | o | | | | |
| heavy chain 11 | 1 | o | | | | | | | |
| heavy chain 2 | 1 | o | | | | | | | |
| heavy chain skeletal perinatal | 1 | o | | | | | | | |
| heavy chain smooth muscle | 1 | | o | | | | | | |
| heavy neurofilament protein | 1 | | | | | | o | | |
| heavy polypeptide 5 | 1 | o | | | | | | | |
| heavy polypeptide cardiac alpha-like | 1 | o | | | | | | | |
| heavy polypeptide isoform cra_a | 1 | | o | | | | | | |
| hect domain and rld 4 | 1 | | | o | | | | | |
| hect domain and rld 5 | 1 | | | o | | | | | |
| hedgehog acyltransferase | 1 | | | | | o | | | |
| hedgehog acyltransferase-like | 1 | o | | | | | | | |
| hedgehog interacting protein | 1 | | | | | o | | | |
| hedgehog-interacting protein | 1 | | o | | | | | | |

| | | | | | | | | | |
|---|---|---|---|---|---|---|---|---|---|
| helentron 5 helitron-like transposon replicase helicase endonuclease | 1 | | | | | | | o | |
| helicase mov-10- | 1 | | o | | | | | | |
| helicase mov-10-like | 1 | | | o | | | | | |
| helicase ski2w | 1 | o | | | | | | | |
| hells protein | 1 | o | | | | | | | |
| hemagluttinin family protein | 1 | | | | o | | | | |
| hematopoietic cell-specific lyn substrate 1 | 1 | o | | | | | | | |
| hematopoietic signal peptide-containing | 1 | o | | | | | | | |
| hematopoietic signal peptide-containing secreted 1 | 1 | | | | | o | | | |
| hematopoietically expressed homeobox | 1 | | o | | | | | | |
| heme binding protein 2 | 1 | | o | | | | | | |
| heme oxygenase | 1 | o | | | | | | | |
| heme-binding protein 2 | 1 | | o | | | | | | |
| hemicentin 1 | 1 | o | | | | | | | |
| hemoglobin subunit alpha-d | 1 | o | | | | | | | |
| hemolytic complement | 1 | | o | | | | | | |
| hemolytic complement-like | 1 | o | | | | | | | |
| heparan sulfate 2-o-sulfotransferase 1 | 1 | | | | o | | | | |
| heparan sulfate d-glucosaminyl 3-o-sulfotransferase 4 | 1 | | o | | | | | | |
| heparan sulfate glucosamine 3-o-sulfotransferase 3b1 | 1 | | | o | | | | | |
| heparan sulfate glucosamine 3-o-sulfotransferase 3b1-like | 1 | | | o | | | | | |
| heparanase 2 | 1 | | o | | | | | | |
| heparin-binding egf-like growth factor | 1 | o | | | | | | | |
| hepatocellular carcinoma-associated antigen 127 | 1 | | | | | | | | o |
| hepatocellular carcinoma-associated gene td26 | 1 | | | | o | | | | |
| hepatocyte cell adhesion molecule | 1 | | | | | o | | | |
| hepatoma-derived growth factor | 1 | o | | | | | | | |
| hepatoma-derived growth factor (high-mobility group protein 1-like) | 1 | | | o | | | | | |
| hephaestin isoform 1 | 1 | | | o | | | | | |
| hephaestin-like protein 1 | 1 | | | | | | o | | |
| hermansky-pudlak syndrome 1 | 1 | o | | | | | | | |
| herv-h ltr-associating 2 | 1 | | | o | | | | | |
| heterochromatin protein 1-beta-like | 1 | | | o | | | | | |
| heterochromatin protein binding protein 3 | 1 | | | | | | o | | |
| heterogeneous nuclear ribonucleoprotein | 1 | | | | | | o | | |
| heterogeneous nuclear ribonucleoprotein l-like isoform 2 | 1 | | | | | o | | | |
| heterogeneous nuclear ribonucleoprotein u-like protein 1-like | 1 | | o | | | | | | |
| heterogeneous nuclear ribonucleoproteins c1 c2 | 1 | | | | o | | | | |
| heterogeneous nuclear ribonucleoproteins c1 c2-like | 1 | | | | o | | | | |
| hexamethylene bis-acetamide inducible 1 | 1 | | o | | | | | | |
| hexaprenyldihydroxybenzoate mitochondrial-like | 1 | | | | o | | | | |
| hexosaminidase a (alpha polypeptide) | 1 | o | | | | | | | |
| hexosaminidase b (beta polypeptide) | 1 | | o | | | | | | |
| high affinity camp-specific and ibmx-insensitive 3 -cyclic phosphodiesterase 8b-like | 1 | o | | | | | | | |
| high affinity cgmp-specific 3 -cyclic phosphodiesterase 9a-like | 1 | | o | | | | | | |

| | | | | | | | | | |
|---|---|---|---|---|---|---|---|---|---|
| high affinity choline transporter 1 | 1 | o | | | | | | | |
| high density lipoprotein binding isoform cra_a | 1 | o | | | | | | | |
| high density lipoprotein binding isoform cra_c | 1 | | | | | | o | | |
| high molecular weight subunit pw212 precursor-related protein | 1 | | | | | | | | o |
| highly divergent homeobox | 1 | | o | | | | | | |
| hippocampus abundant transcript 1 | 1 | o | | | | | | | |
| hira | 1 | o | | | | | | | |
| hira interacting protein 3 | 1 | o | | | | | | | |
| histamine h1 receptor | 1 | | | | | | | | o |
| histidine kinase- dna gyrase b- and hsp90-like domain containing partial | 1 | | | o | | | | | |
| histidine triad hit-5 | 1 | o | | | | | | | |
| histidine triad nucleotide binding protein 3 | 1 | o | | | | | | | |
| histidine-rich glycoprotein precursor | 1 | | | | | | | o | |
| histone acetyltransferase myst4-like | 1 | o | | | | | | | |
| histone deacetylase | 1 | | | | o | | | | |
| histone deacetylase 10 | 1 | | o | | | | | | |
| histone deacetylase complex subunit sap30l-like isoform 2 | 1 | | o | | | | | | |
| histone h2b 5-like | 1 | | | | | | o | | |
| histone h3 type 2 | 1 | | o | | | | | | |
| histone h3-like centromeric protein a | 1 | | o | | | | | | |
| histone h4-like | 1 | | o | | | | | | |
| histone rna hairpin-binding | 1 | | | | o | | | | |
| histone stem-loop binding protein | 1 | | | | | o | | | |
| histone-lysine n-methyltransferase mll | 1 | o | | | | | | | |
| histone-lysine n-methyltransferase nsd3 | 1 | | | | | | o | | |
| histone-lysine n-methyltransferase setd1b | 1 | | | | | | | | o |
| histone-lysine n-methyltransferase setd2 | 1 | | o | | | | | | |
| histone-lysine n-methyltransferase setd2-like | 1 | | | | | | o | | |
| histone-lysine n-methyltransferase setdb1-b-like | 1 | | | | | | | | o |
| hiv tat specific factor 1 | 1 | | | | | | o | | |
| hiv-1 rev binding | 1 | | | | | | o | | |
| hla-b associated transcript 2 | 1 | | | | | | o | | |
| hla-b associated transcript 5 | 1 | o | | | | | | | |
| hmg box protein sox3 | 1 | | | | | | | o | |
| hmg box-containing protein 1 | 1 | o | | | | | | | |
| hmha1 protein | 1 | | o | | | | | | |
| hmp19 protein | 1 | | | | | | | | o |
| hnrnpd protein | 1 | | o | | | | | | |
| hnrnpl protein | 1 | | o | | | | | | |
| homeo box c5 | 1 | o | | | | | | | |
| homeobox and leucine zipper encoding | 1 | | | | o | | | | |
| homeobox c8 | 1 | o | | | | | | | |
| homeobox d9-like | 1 | o | | | | | | | |
| homeobox domain-containing protein | 1 | | | | | | o | | |
| homeobox protein hmx2 | 1 | | | | | | o | | |
| homeobox protein meis1 | 1 | o | | | | | | | |
| homeobox protein six1 | 1 | | | | | | | | o |
| homeobox prox 1 | 1 | | o | | | | | | |
| homeobox-containing protein 1-like | 1 | | | | | | o | | |
| homeodomain interacting protein kinase 3 isoform 1 | 1 | | | o | | | | | |
| homeodomain-interacting protein kinase 1 | 1 | | | | | | o | | |
| homeodomain-interacting protein kinase 2 isoform 2 | 1 | o | | | | | | | |
| homer homolog 2 | 1 | o | | | | | | | |
| homer protein homolog 1-like | 1 | | | | | | o | | |

| | | | | | | | | | |
|---|---|---|---|---|---|---|---|---|---|
| homer protein homolog 2 | 1 | o | | | | | | | |
| homo sapiens c21orf63 isoform b | 1 | o | | | | | | | |
| homocysteine s-methyltransferase | 1 | | o | | | | | | |
| homocysteine-responsive endoplasmic reticulum-resident ubiquitin-like domain member 2 protein | 1 | o | | | | | | | |
| homolog dmc1 | 1 | o | | | | | | | |
| homolog precursor | 1 | o | | | | | | | |
| homolog subfamily a member 4 | 1 | | | | | o | | | |
| homologous-pairing protein 2 homolog | 1 | o | | | | | | | |
| hook homolog 2 | 1 | o | | | | | | | |
| horma domain containing 1 | 1 | | | | | | | | o |
| horma domain-containing protein 1 | 1 | | | | o | | | | |
| hormone sensitive lipase | 1 | | o | | | | | | |
| hras-like suppressor 2 | 1 | | | o | | | | | |
| hs1-associating protein x-1 | 1 | | | o | | | | | |
| hsc70-interacting protein | 1 | | o | | | | | | |
| hsp90 co-chaperone cdc37-like 1 | 1 | o | | | | | | | |
| hspb (heat shock 27kda) associated protein 1 | 1 | o | | | | | | | |
| human homologue | 1 | | o | | | | | | |
| human immunodeficiency virus type i enhancer binding protein 1 | 1 | o | | | | | | | |
| human immunodeficiency virus type i enhancer-binding protein 2 homolog | 1 | | | o | | | | | |
| huwe1 protein | 1 | | | | | | | | o |
| hyaluronan and proteoglycan link protein 4 | 1 | | | | | | | | o |
| hyaluronan-binding protein 2-like | 1 | | | | o | | | | |
| hyaluronidase-2 precursor | 1 | | | | | | | | o |
| hyaluronoglucosaminidase 2 | 1 | o | | | | | | | |
| hydrocephalus-inducing protein | 1 | o | | | | | | | |
| hydroxyacid oxidase 2 | 1 | | o | | | | | | |
| hydroxyacid oxidase 2 (long chain) | 1 | | | o | | | | | |
| hydroxyacyl glutathione hydrolase | 1 | | | o | | | | | |
| hydroxyacyl-coenzyme a dehydrogenase 3-ketoacyl-coenzyme a thiolase enoyl-coenzyme a hydratase (trifunctional protein) beta subunit | 1 | | o | | | | | | |
| hydroxymethylglutaryl- cytoplasmic | 1 | | o | | | | | | |
| hydroxymethylglutaryl- synthase 1 | 1 | | o | | | | | | |
| hydroxyproline-rich glycoprotein dz-hrgp | 1 | | | | | o | | | |
| hyou1 protein | 1 | | | o | | | | | |
| hypermethylated in cancer 1 protein | 1 | o | | | | | | | |
| hypermethylated in cancer 2 | 1 | | | | | | o | | |
| hyperpolarization activated cyclic nucleotide-gated potassium channel 2 | 1 | | | | | | o | | |
| hypothetical isoform cra_a | 1 | | o | | | | | | |
| hypothetical loc298077 | 1 | | | o | | | | | |
| hypothetical loc539475 | 1 | | o | | | | | | |
| hypothetical loc565051 | 1 | o | | | | | | | |
| hypothetical loc570390 | 1 | | | o | | | | | |
| hypothetical malaria antigen | 1 | | | | o | | | | |
| hypoxia up-regulated protein 1 precursor | 1 | | | o | | | | | |
| hypoxia-inducible factor 1 alpha | 1 | o | | | | | | | |
| hypoxia-inducible factor prolyl 4-hydroxylase | 1 | o | | | | | | | |
| ice recrystallisation inhibition protein | 1 | | | | | | | | o |
| ig kappa chain v-iii region mopc 63 precursor | 1 | o | | | | | | | |
| igf binding protein 2 | 1 | | | | | o | | | |
| ik cytokine | 1 | | | | | o | | | |
| im:7137941 protein | 1 | o | | | | | | | |
| im:7145112 protein | 1 | o | | | | | | | |

| | | | | | | | | | |
|---|---|---|---|---|---|---|---|---|---|
| im:7151680 protein | 1 | | | | | o | | | |
| imap family member 7 | 1 | | | o | | | | | |
| immature colon carcinoma transcript 1 | 1 | o | | | | | | | |
| immune-responsive gene 1 protein homolog | 1 | o | | | | | | | |
| immunoglobulin light chain isotype l3 | 1 | o | | | | | | | |
| immunoglobulin superfamily containing leucine-rich repeat protein 2 isoform b | 1 | | | | | o | | | |
| immunoglobulin superfamily containing leucine-rich repeat protein 2-like | 1 | | | | | | | | o |
| immunoglobulin superfamily member 10-like | 1 | o | | | | | | | |
| immunoglobulin superfamily member 6 precursor | 1 | o | | | | | | | |
| imp (inosine monophosphate) dehydrogenase 2 | 1 | o | | | | | | | |
| importin 13 | 1 | o | | | | | | | |
| importin 8 | 1 | | o | | | | | | |
| importin partial | 1 | o | | | | | | | |
| importin subunit alpha-1 | 1 | | o | | | | | | |
| Importin-4 [Dicentrarchus labrax] | 1 | | | | | | o | | |
| in family member (ttn-1) | 1 | o | | | | | | | |
| inactive phospholipase c-like protein 1 | 1 | | | | | | o | | |
| inclusion membrane protein | 1 | | | o | | | | | |
| induced myeloid leukemia cell differentiation protein mcl-1 homolog | 1 | | | | o | | | | |
| ing (mammalian inhibitor of growth) homolog family member (ing-3)-like | 1 | | | | | | | o | |
| inhibin alpha chain precursor | 1 | | | o | | | | | |
| inhibitor kappa b alpha | 1 | | o | | | | | | |
| inhibitor of growth member 5 | 1 | o | | | | | | | |
| inhibitor of growth protein 1 | 1 | | | | o | | | | |
| inhibitor of growth protein 3 | 1 | | | | | | | o | |
| inhibitor of kappa light polypeptide gene enhancer in b- kinase epsilon | 1 | | o | | | | | | |
| inhibitor of kappa light polypeptide gene enhancer in b- kinase gamma | 1 | | | | | | | o | |
| inhibitor of nuclear factor kappa-b kinase subunit alpha | 1 | | o | | | | | | |
| inhibitor of nuclear factor kappa-b kinase-interacting protein isoform 1 | 1 | | o | | | | | | |
| inner membrane protein | 1 | | o | | | | | | |
| inorganic pyrophosphatase mitochondrial precursor | 1 | | | | | o | | | |
| inosine monophosphate dehydrogenase 1 | 1 | | | | | | | o | |
| inosine monophosphate dehydrogenase 2 | 1 | | | | | o | | | |
| inositol hexakisphosphate kinase 2 | 1 | o | | | | | | | |
| inositol monophosphatase 1 | 1 | o | | | | | | | |
| inositol polyphosphate 5-phosphatase ocrl-1-like | 1 | | | | | | o | | |
| inositol polyphosphate-5- 75kda | 1 | o | | | | | | | |
| inositol -triphosphate receptor 2 | 1 | | o | | | | | | |
| inositol -trisphosphate receptor type 1 | 1 | o | | | | | | | |
| inositol -trisphosphate receptor type 1-like | 1 | o | | | | | | | |
| inositol-tetrakisphosphate 1-kinase | 1 | | | | | | o | | |
| inositol-trisphosphate 3-kinase a | 1 | o | | | | | | | |
| inscuteable homolog | 1 | | | o | | | | | |
| insect-derived growth factor-a-like protein | 1 | | | o | | | | | |
| insulin gene enhancer protein isl-2 | 1 | | | | | | | | o |
| insulin receptor | 1 | | | | | | o | | |
| insulin receptor b | 1 | | | | | | o | | |
| insulin receptor substrate 4 | 1 | o | | | | | | | |

| | | | | | | | | | |
|---|---|---|---|---|---|---|---|---|---|
| insulin-like growth factor 1 receptor | 1 | | | | | | o | | |
| insulin-like growth factor 2 receptor | 1 | | | | | | | | o |
| insulin-like growth factor binding protein complex acid-labile subunit | 1 | | | | o | | | | |
| insulin-like growth factor binding protein-2 | 1 | | o | | | | | | |
| integral membrane protein | 1 | | | | | | | o | |
| integrase core domain protein | 1 | | | | | | o | | |
| integrator complex subunit 10 | 1 | o | | | | | | | |
| integrator complex subunit 11-like | 1 | | | | o | | | | |
| integrator complex subunit 5 | 1 | | | | o | | | | |
| integrin alpha fg-gap repeat containing 3 | 1 | o | | | | | | | |
| integrin alpha m | 1 | o | | | | | | | |
| integrin alpha-7 partial | 1 | o | | | | | | | |
| integrin alpha-9 | 1 | | | | | | o | | |
| integrin beta-1 subunit isoform 2 | 1 | o | | | | | | | |
| interaction protein for cytohesin exchange factors 1 | 1 | | | | | | | | o |
| interactor protein for cytohesin exchange factors 1 | 1 | o | | | | | | | |
| inter-alpha inhibitor h2 | 1 | | o | | | | | | |
| inter-alpha inhibitor h5 | 1 | o | | | | | | | |
| inter-alpha-trypsin inhibitor heavy chain h1 | 1 | o | | | | | | | |
| inter-alpha-trypsin inhibitor heavy chain h2-like | 1 | | | | o | | | | |
| inter-alpha-trypsin inhibitor heavy chain h3 precursor | 1 | o | | | | | | | |
| inter-alpha-trypsin inhibitor heavy chain h4 | 1 | | | | | | | o | |
| interferon ( beta and omega) receptor 1 | 1 | | | o | | | | | |
| interferon- double-stranded rna-activated protein kinase | 1 | | | | | | o | | |
| interferon- double-stranded rna-activated protein kinase-like | 1 | | | | o | | | | |
| interferon gamma receptor 2 | 1 | o | | | | | | | |
| interferon induced protein 2 | 1 | o | | | | | | | |
| interferon induced transmembrane protein 5 | 1 | | | o | | | | | |
| interferon inducible protein isg12 | 1 | | | | | | | | o |
| interferon regulatory factor 2 | 1 | | | | | | o | | |
| interferon regulatory factor 2 binding protein 2 | 1 | | | | | | o | | |
| interferon regulatory factor 2-binding protein 1 | 1 | | | | | | | | o |
| interferon regulatory factor 4 | 1 | o | | | | | | | |
| interferon-induced 35 kda protein homolog | 1 | | | | | | | | o |
| interferon-induced guanylate-binding protein 2-like | 1 | o | | | | | | | |
| interferon-induced protein with tetratricopeptide repeats 5-like | 1 | | o | | | | | | |
| interferon-induced transmembrane protein 3 | 1 | o | | | | | | | |
| interferon-inducible protein gig1 | 1 | | o | | | | | | |
| interferon-inducible protein gig2 | 1 | o | | | | | | | |
| interferon-inducible transmembrane protein 1 | 1 | | o | | | | | | |
| interferon-related developmental regulator 2 | 1 | o | | | | | | | |
| interleukin 1 receptor accessory 1 | 1 | | | | | | o | | |
| interleukin 1 receptor-like 2 | 1 | o | | | | | | | |
| interleukin 15 | 1 | o | | | | | | | |
| interleukin 15-like | 1 | o | | | | | | | |
| interleukin 17 isoform d | 1 | | | | o | | | | |
| interleukin 6 signal transducer ( oncostatin m receptor)-like | 1 | o | | | | | | | |
| interleukin enhancer binding factor 2 | 1 | | o | | | | | | |

| | | | | | | | | | |
|---|---|---|---|---|---|---|---|---|---|
| interleukin enhancer-binding factor 3 homolog | 1 | o | | | | | | | |
| interleukin-1 receptor antagonist | 1 | o | | | | | | | |
| interleukin-1 receptor type 1-like | 1 | | o | | | | | | |
| interleukin-1 receptor type 2-like | 1 | | o | | | | | | |
| interleukin-1 receptor-associated kinase 4 | 1 | | o | | | | | | |
| interleukin-10 receptor beta chain precursor | 1 | o | | | | | | | |
| interleukin-15 receptor alpha chain | 1 | o | | | | | | | |
| Interleukin-7 [Dicentrarchus labrax] | 1 | | | | o | | | | |
| interleukin-8 | 1 | o | | | | | | | |
| interleukin-8-like protein | 1 | o | | | | | | | |
| intermediate chain 2 | 1 | | o | | | | | | |
| intermediate filament 1 | 1 | o | | | | | | | |
| intermediate filament family orphan 2 | 1 | o | | | | | | | |
| intermediate filament protein | 1 | o | | | | | | | |
| interphotoreceptor matrix proteoglycan 2 | 1 | | | | | | o | | |
| intersectin 1 isoform itsn-l | 1 | | o | | | | | | |
| intersectin 2 | 1 | | | | | | o | | |
| intraflagellar transport protein 122 homolog | 1 | o | | | | | | | |
| intraflagellar transport protein 172 homolog | 1 | | | | | | o | | |
| intraflagellar transport protein 52 homolog | 1 | o | | | | | | | |
| ipo7 protein | 1 | | o | | | | | | |
| iq domain-containing protein k-like | 1 | | | | | | | | o |
| iq motif and sec7 domain 1 | 1 | | o | | | | | | |
| iq motif and sec7 domain 2 | 1 | | | | | | o | | |
| iq motif and sec7 domain-containing protein 2-like | 1 | | | | o | | | | |
| iq motif containing c | 1 | o | | | | | | | |
| iron zinc purple acid phosphatase-like protein | 1 | | | o | | | | | |
| iron zinc purple acid phosphatase-like protein precursor | 1 | o | | | | | | | |
| iron-sulfur cluster assembly enzyme mitochondrial | 1 | | o | | | | | | |
| iron-sulfur cluster assembly enzyme mitochondrial-like isoform 2 | 1 | | | | o | | | | |
| iroquois-class homeodomain protein irx-1 | 1 | | | | | | | | o |
| iroquois-class homeodomain protein irx-3 | 1 | o | | | | | | | |
| isoleucyl-trna cytoplasmic | 1 | | | | | | | o | |
| isoleucyl-trna mitochondrial | 1 | | | | | | | | o |
| isoleucyl-trna mitochondrial-like | 1 | | | | | | | | o |
| isopropylmalate isomerase large subunit | 1 | o | | | | | | | |
| itchy e3 ubiquitin protein ligase homolog | 1 | | o | | | | | | |
| itsn2 protein | 1 | | o | | | | | | |
| jagged 2 | 1 | o | | | | | | | |
| janus kinase 1 | 1 | | o | | | | | | |
| janus kinase and microtubule interacting protein 1 | 1 | | o | | | | | | |
| jazf zinc finger 1 | 1 | o | | | | | | | |
| jemma protein | 1 | o | | | | | | | |
| jerky homolog-like | 1 | o | | | | | | | |
| jumonji domain containing 3 | 1 | | | | | | o | | |
| jumonji domain containing 7 | 1 | o | | | | | | | |
| jumonji domain-containing 3 | 1 | | | | | | | | o |
| jun dimerization protein 2 | 1 | | | | | | | | o |
| junctional adhesion molecule 2a | 1 | o | | | | | | | |
| junctophilin 2 | 1 | o | | | | | | | |
| k acetyltransferase 2b | 1 | o | | | | | | | |
| k1468_danre ame: full= domain and heat repeat-containing protein kiaa1468 homolog | 1 | o | | | | | | | |
| kallmann syndrome 1 sequence | 1 | o | | | | | | | |
| karyopherin alpha 3 (importin alpha 4) | 1 | | | | | | o | | |

| | | | | | | | | | |
|---|---|---|---|---|---|---|---|---|---|
| karyopherin alpha 4 (importin alpha 3) | 1 | o | | | | | | | |
| karyopherin alpha 6 | 1 | o | | | | | | | |
| kdel (lys-asp-glu-leu) containing 1 | 1 | | | | | | | | o |
| ke (drosophila actin-binding) homolog family member (ketn-1) | 1 | o | | | | | | | |
| kelch domain containing 10 | 1 | o | | | | | | | |
| kelch domain containing 2 | 1 | o | | | | | | | |
| kelch domain containing 6 | 1 | o | | | | | | | |
| kelch domain-containing protein 10 | 1 | | o | | | | | | |
| kelch repeat | 1 | o | | | | | | | |
| kelch repeat and btb domain-containing protein 12 | 1 | o | | | | | | | |
| kelch repeat and btb domain-containing protein 13 | 1 | o | | | | | | | |
| kelch repeat and btb domain-containing protein 2 | 1 | | | | | | o | | |
| kelch repeat and btb domain-containing protein 3 | 1 | | | | | | | | o |
| kelch repeat and btb domain-containing protein 4 | 1 | | | | o | | | | |
| kelch repeat-containing protein | 1 | | | | o | | | | |
| kelch-like 31 | 1 | o | | | | | | | |
| kelch-like 33 | 1 | o | | | | | | | |
| kelch-like 5 | 1 | | | | | | o | | |
| kelch-like 9 | 1 | o | | | | | | | |
| kelch-like ech-associated protein 1-like | 1 | | | | o | | | | |
| kelch-like protein 10 | 1 | | | | o | | | | |
| kelch-like protein 17-like | 1 | | | | | o | | | |
| kelch-like protein 29-like | 1 | | | | | | o | | |
| keratin 15 | 1 | o | | | | | | | |
| keratin 7-like | 1 | | | o | | | | | |
| keratin-associated protein 10-4-like | 1 | | | | o | | | | |
| keratin-associated protein 10-8-like | 1 | | | | | | | | o |
| keratin-associated protein 10-like ensp00000375147 homolog | 1 | | | | | | | | o |
| keratocan precursor | 1 | | | | | o | | | |
| kh domain- rna- signal transduction-associated protein 1-like | 1 | | | | | | | | o |
| kiaa0020 protein | 1 | | o | | | | | | |
| kiaa0090 isoform 1 | 1 | o | | | | | | | |
| kiaa0100 protein | 1 | o | | | | | | | |
| kiaa0256 protein | 1 | | | | | | o | | |
| kiaa0298 protein | 1 | | | | | | | | o |
| kiaa0494 protein | 1 | | | | | o | | | |
| kiaa0799 protein | 1 | | o | | | | | | |
| kiaa0920 protein | 1 | o | | | | | | | |
| kiaa0954 protein | 1 | | | | | | | o | |
| kiaa0975 protein | 1 | o | | | | | | | |
| kiaa0979 protein | 1 | | | | | | | o | |
| kiaa0999 protein | 1 | o | | | | | | | |
| kiaa1026 protein | 1 | | | | | | o | | |
| kiaa1033 protein | 1 | o | | | | | | | |
| kiaa1095 protein | 1 | o | | | | | | | |
| kiaa1310 protein | 1 | o | | | | | | | |
| kiaa1378 protein | 1 | | | | | | o | | |
| kiaa1417 protein | 1 | o | | | | | | | |
| kiaa1589 protein | 1 | | | o | | | | | |
| kiaa1727 protein | 1 | o | | | | | | | |
| kiaa1822 protein | 1 | | | o | | | | | |
| kiaa1826 protein | 1 | | o | | | | | | |

| | | | | | | | | | |
|---|---|---|---|---|---|---|---|---|---|
| kiaa1882 protein | 1 | | | | | | o | | |
| kiaa1909 protein | 1 | o | | | | | | | |
| kiaa2010 protein | 1 | | o | | | | | | |
| kif21a protein | 1 | o | | | | | | | |
| kif9 protein | 1 | | o | | | | | | |
| kin of irre like 2 | 1 | o | | | | | | | |
| kin of irre like 3 | 1 | | | | | | o | | |
| kin of irre-like protein 3-like | 1 | | | | | | | | o |
| kinase c-binding protein 1 | 1 | o | | | | | | | |
| kinase suppressor of ras 1 | 1 | | o | | | | | | |
| kinase suppressor of ras 2 | 1 | | | | | | o | | |
| kinase suppressor of ras cg2899- partial | 1 | | | o | | | | | |
| kinase-like vesicle-associated | 1 | | | | | | o | | |
| kinesin family member 14-like | 1 | o | | | | | | | |
| kinesin family member 1c | 1 | o | | | | | | | |
| kinesin family member 20a | 1 | | | | o | | | | |
| kinesin family member 23 | 1 | | o | | | | | | |
| kinesin family member 26b | 1 | | | | | | o | | |
| kinesin family member 3c | 1 | | | | | o | | | |
| kinesin family member 4a | 1 | o | | | | | | | |
| kinesin family member 5a | 1 | | | | | | o | | |
| kinesin family member c1 | 1 | o | | | | | | | |
| kinesin family member c3 | 1 | | o | | | | | | |
| kinesin heavy chain isoform 5c-like | 1 | | | | | | o | | |
| kinesin light chain 2 | 1 | | | | | | | | o |
| kinesin-like protein kif13b-like | 1 | | | | | | | | o |
| kinesin-like protein kif1b-like isoform 3 | 1 | | | | | | | o | |
| kinesin-like protein kif20a | 1 | | | | o | | | | |
| kinesin-like protein kif2c | 1 | | o | | | | | | |
| kinetochore protein ndc80 homolog | 1 | | o | | | | | | |
| kinetochore protein nuf2 | 1 | o | | | | | | | |
| kinetochore protein spc25 | 1 | o | | | | | | | |
| kinetochore-associated protein dsn1 homolog | 1 | | | | o | | | | |
| kinetoplast-associated protein | 1 | o | | | | | | | |
| kininogen-1 isoform 1 | 1 | | | | o | | | | |
| kit ligand | 1 | o | | | | | | | |
| klf534-like protein | 1 | o | | | | | | | |
| klraq motif-containing protein 1-like | 1 | | o | | | | | | |
| kn motif and ankyrin repeat domains 1 | 1 | | | o | | | | | |
| krev interaction trapped protein 1 | 1 | o | | | | | | | |
| krueppel c2h2-type zinc finger | 1 | o | | | | | | | |
| krueppel-like factor 6-like isoform 1 | 1 | | | | | | | | o |
| kruppel-like factor 10 | 1 | | | | o | | | | |
| kruppel-like factor 4 | 1 | o | | | | | | | |
| ktel (lys-tyr-glu-leu) containing 1 | 1 | o | | | | | | | |
| kunitz-type protease inhibitor 1 precursor | 1 | | | | o | | | | |
| kv channel interacting protein like | 1 | | | o | | | | | |
| l-2-hydroxyglutarate mitochondrial | 1 | | o | | | | | | |
| la ribonucleoprotein domain member 1b | 1 | | o | | | | | | |
| lactate dehydrogenase d | 1 | | o | | | | | | |
| lactose-binding lectin l-2 | 1 | | o | | | | | | |
| lactosylceramide alpha- -sialyltransferase | 1 | | o | | | | | | |
| lag1 longevity assurance homolog 5 | 1 | o | | | | | | | |
| lag1 longevity assurance-like protein 1 | 1 | | | | | | o | | |
| lamin a | 1 | o | | | | | | | |
| lamin b1 | 1 | | | | o | | | | |
| lamin b3 | 1 | o | | | | | | | |
| lamina-associated polypeptide 1b | 1 | o | | | | | | | |
| lamina-associated polypeptide isoforms beta gamma-like | 1 | o | | | | | | | |

| | | | | | | | | | |
|---|---|---|---|---|---|---|---|---|---|
| laminin alpha 3 splice variant b1 | 1 | o | | | | | | | |
| laminin alpha 3b chain | 1 | o | | | | | | | |
| laminin alpha 4 | 1 | | | o | | | | | |
| laminin subunit beta-1-like | 1 | | | | | | | | o |
| laminin subunit beta-2 | 1 | | | | | | o | | |
| laminin subunit gamma-1 precursor | 1 | o | | | | | | | |
| l-amino acid oxidase | 1 | | | o | | | | | |
| lamp family protein c20orf103 homolog precursor | 1 | | | | | | | | o |
| lanosterol 14-alpha demethylase | 1 | | | | | o | | | |
| lantibiotic synthetase component c-like 2 | 1 | o | | | | | | | |
| la-related protein 7-like | 1 | | | | | | o | | |
| la-related protein partial | 1 | | | | | | o | | |
| large ala glu-rich protein | 1 | | | | o | | | | |
| large neutral amino acids transporter small subunit 1 | 1 | | | | | | o | | |
| large neutral amino acids transporter small subunit 2 | 1 | | | | o | | | | |
| large tegument protein | 1 | o | | | | | | | |
| latrophilin and seven transmembrane domain containing 1 | 1 | o | | | | | | | |
| latrophilin-1 precursor | 1 | | | | | | o | | |
| latrophilin-2 isoform 1 | 1 | | | | | | o | | |
| lats homolog 1-like | 1 | | | | | | | o | |
| layilin b | 1 | o | | | | | | | |
| lbr protein | 1 | | | | | o | | | |
| lecithin cholesterol acyl transferase | 1 | | | o | | | | | |
| lecithin retinol acyltransferase (phosphatidylcholine--retinol o-acyltransferase) | 1 | | | | | | | | o |
| lectin precursor | 1 | | o | | | | | | |
| lectin type 2 | 1 | | o | | | | | | |
| leiomodin 1 (smooth muscle) | 1 | o | | | | | | | |
| lens fiber major intrinsic protein | 1 | o | | | | | | | |
| leprecan-like 2 | 1 | o | | | | | | | |
| leucine rich repeat (in flii) interacting protein 2 | 1 | o | | | | | | | |
| leucine rich repeat and fibronectin type iii domain containing 2 | 1 | | | | | | | o | |
| leucine rich repeat containing 20 | 1 | o | | | | | | | |
| leucine rich repeat containing 28 | 1 | | o | | | | | | |
| leucine rich repeat containing 32 | 1 | o | | | | | | | |
| leucine rich repeat containing 33 | 1 | o | | | | | | | |
| leucine rich repeat containing 42 | 1 | o | | | | | | | |
| leucine rich repeat containing 47 | 1 | | | | o | | | | |
| leucine rich repeat neuronal 2 | 1 | | | | | | o | | |
| leucine rich repeat transmembrane neuronal 2 | 1 | | | | | | o | | |
| leucine tumor suppressor 2 | 1 | o | | | | | | | |
| leucine zipper protein 2 isoform 1 | 1 | | | | | o | | | |
| leucine-rich glioma-inactivated protein 1 | 1 | | | | | | | | o |
| leucine-rich glioma-inactivated protein 1-like | 1 | | | | | o | | | |
| leucine-rich repeat and coiled-coil domain-containing protein 1 | 1 | | o | | | | | | |
| leucine-rich repeat and fibronectin type iii domain-containing protein 1-like | 1 | | | | | | o | | |
| leucine-rich repeat and fibronectin type-iii domain-containing protein 6-like | 1 | | | | | | o | | |
| leucine-rich repeat and immunoglobulin-like domain-containing nogo receptor-interacting protein 1-like | 1 | | | | | | o | | |

| | | | | | | | | | |
|---|---|---|---|---|---|---|---|---|---|
| leucine-rich repeat and immunoglobulin-like domain-containing nogo receptor-interacting protein 2-like | 1 | | | | | | o | | |
| leucine-rich repeat domain protein | 1 | o | | | | | | | |
| leucine-rich repeat flightless-interacting protein 2 | 1 | o | | | | | | | |
| leucine-rich repeat flightless-interacting protein 2 isoform 1 | 1 | | | | | | | | o |
| leucine-rich repeat lgi family member 2 precursor | 1 | | | | | | | | o |
| leucine-rich repeat neuronal protein 3-like | 1 | | | | | | | | o |
| leucine-rich repeat protein shoc-2 | 1 | o | | | | | | | |
| leucine-rich repeat-containing g protein-coupled receptor 4 | 1 | | | o | | | | | |
| leucine-rich repeat-containing protein 32-like | 1 | | o | | | | | | |
| leucine-rich repeat-containing protein 42 | 1 | | | | o | | | | |
| leucine-rich repeat-containing protein 49 | 1 | | | | | | | | o |
| leucine-rich repeat-containing protein 59 | 1 | | | | | | | | o |
| leucine-rich repeat-containing protein 9-like | 1 | | | | o | | | | |
| leucine-rich repeats and calponin homology domain containing 2 | 1 | | | | | | o | | |
| leucine-rich repeats and transmembrane domains 1 | 1 | o | | | | | | | |
| leukocyte common antigen precursor | 1 | o | | | | | | | |
| leukocyte membrane antigen-like | 1 | o | | | | | | | |
| leukocyte receptor cluster member 4 | 1 | | | | | | o | | |
| leukocyte receptor cluster member 9 | 1 | | o | | | | | | |
| leukotriene b4 receptor | 1 | o | | | | | | | |
| leukotriene b4 receptor 1 | 1 | | o | | | | | | |
| leukotriene c4 synthase | 1 | o | | | | | | | |
| leydig cell tumor 10 kda protein | 1 | o | | | | | | | |
| ligand of numb protein x 2-like | 1 | | | | | | o | | |
| ligand of numb-protein x 2 | 1 | | | | | | o | | |
| ligase atp-dependent | 1 | o | | | | | | | |
| light ear protein | 1 | o | | | | | | | |
| light intermediate polypeptide 1 | 1 | | | | | | | o | |
| light polypeptide | 1 | | | | | | o | | |
| light polypeptide kinase | 1 | | o | | | | | | |
| light tctex-type 1 | 1 | o | | | | | | | |
| lim and calponin homology domains 1 isoform 4 | 1 | | | | | | o | | |
| lim and senescent cell antigen-like domains 1 isoform 2 | 1 | o | | | | | | | |
| lim domain binding 1 | 1 | | | | | | | | o |
| lim domain containing preferred translocation partner in lipoma | 1 | | o | | | | | | |
| lim domain kinase 2 | 1 | | | | | | | | o |
| lim domain only 1 | 1 | | | | | o | | | |
| lim domain only 1 (rhombotin 1) | 1 | | | | | | | o | |
| lim domain only 2 | 1 | o | | | | | | | |
| lim domain transcription factor lmo4 | 1 | | | | | | | | o |
| lim domain transcription factor lmo4-like | 1 | o | | | | | | | |
| lim domain-binding protein 1 | 1 | o | | | | | | | |
| lim domain-binding protein 3-like | 1 | | o | | | | | | |
| lim homeobox 6 | 1 | o | | | | | | | |
| limb region 1 homolog | 1 | o | | | | | | | |
| lin-10 homolog ( elegans) isoform cra_b | 1 | o | | | | | | | |
| lin-7 homolog b | 1 | | | | | o | | | |
| lingo2 protein | 1 | | | | | | | o | |

| | | | | | | | | | |
|---|---|---|---|---|---|---|---|---|---|
| lipid phosphate phosphatase-related protein type 1 | 1 | | | | | | o | | |
| lipid phosphate phosphatase-related protein type 2 | 1 | | | | | | o | | |
| lipid phosphate phosphatase-related protein type 5-like | 1 | | | | | | o | | |
| lipid phosphate phosphohydrolase 2 | 1 | o | | | | | | | |
| lipid phosphate phosphohydrolase 3 isoform 2 | 1 | | | | | | o | | |
| lipolysis-stimulated lipoprotein receptor precursor | 1 | | | | o | | | | |
| liprin-beta-1 isoform 1 | 1 | | | | | | | | o |
| lish domain-containing protein armc9 | 1 | | | | | | | | o |
| lish domain-containing protein c16orf63 homolog | 1 | | o | | | | | | |
| lissencephaly-1 homolog b | 1 | o | | | | | | | |
| litaf-like protein | 1 | | o | | | | | | |
| liver angiotensinogen | 1 | | o | | | | | | |
| liver basic fatty acid binding protein | 1 | | o | | | | | | |
| liver glycogen phosphorylase | 1 | o | | | | | | | |
| liver x receptor alpha | 1 | | | | | | o | | |
| lmbr1 domain-containing protein 2 | 1 | | | | o | | | | |
| loc100006898 protein | 1 | o | | | | | | | |
| loc100037905 protein | 1 | o | | | | | | | |
| loc100127300 protein | 1 | | | o | | | | | |
| loc100127334 protein | 1 | o | | | | | | | |
| loc100145034 protein | 1 | | | | | o | | | |
| loc100145131 protein | 1 | o | | | | | | | |
| loc100145420 protein | 1 | o | | | | | | | |
| loc100145470 protein | 1 | | | | | | | o | |
| loc100151157 protein | 1 | | | | | o | | | |
| loc100158280 protein | 1 | o | | | | | | | |
| loc100158319 protein | 1 | | | o | | | | | |
| loc100158343 protein | 1 | | o | | | | | | |
| loc100170538 protein | 1 | | | o | | | | | |
| loc397853 protein | 1 | o | | | | | | | |
| loc398480 protein | 1 | o | | | | | | | |
| loc398501 protein | 1 | | | | | | | | o |
| loc431836 protein | 1 | | o | | | | | | |
| loc443593 protein | 1 | o | | | | | | | |
| loc443703 protein | 1 | | | | | | | o | |
| loc443706 protein | 1 | | | o | | | | | |
| loc446285 protein | 1 | o | | | | | | | |
| loc446936 protein | 1 | | o | | | | | | |
| loc495229 protein | 1 | o | | | | | | | |
| loc497000 protein | 1 | | | o | | | | | |
| loc511108 protein | 1 | o | | | | | | | |
| loc553339 partial | 1 | | o | | | | | | |
| loc553343 protein | 1 | o | | | | | | | |
| loc553490 protein | 1 | o | | | | | | | |
| loc553495 protein | 1 | o | | | | | | | |
| loc556466 protein | 1 | | o | | | | | | |
| loc561593 protein | 1 | | | | | | | | o |
| loc563520 protein | 1 | | | | | | o | | |
| loc563523 protein | 1 | o | | | | | | | |
| loc565404 protein | 1 | o | | | | | | | |
| loc569044 protein | 1 | | o | | | | | | |
| loc569234 protein | 1 | | | | | | o | | |
| loc569631 protein | 1 | | | | | o | | | |
| loc570454 protein | 1 | o | | | | | | | |

| | | | | | | | | | |
|---|---|---|---|---|---|---|---|---|---|
| loc572412 protein | 1 | o | | | | | | | |
| loc733162 protein | 1 | | | | | | o | | |
| loc733202 protein | 1 | o | | | | | | | |
| loc733379 protein | 1 | | | o | | | | | |
| loc733382 protein | 1 | | | | | o | | | |
| loc733419 protein | 1 | o | | | | | | | |
| loc779081 protein | 1 | o | | | | | | | |
| loc794500 protein | 1 | | | | | | o | | |
| loc798746 protein | 1 | o | | | | | | | |
| loc799552 protein | 1 | o | | | | | | | |
| lon peptidase n-terminal domain and ring finger 3 | 1 | | | | | | o | | |
| lon peptidase n-terminal domain and ring finger protein 1 | 1 | | | | o | | | | |
| lon peptidase peroxisomal | 1 | | o | | | | | | |
| lon protease mitochondrial | 1 | | | | o | | | | |
| lon protease mitochondrial precursor | 1 | o | | | | | | | |
| long-chain fatty acid transport protein 1-like | 1 | | | | | | | | o |
| long-chain specific acyl- mitochondrial | 1 | | o | | | | | | |
| long-chain-fatty-acid-- ligase 4 | 1 | | o | | | | | | |
| long-chain-fatty-acid-- ligase 4-like | 1 | | | | | | o | | |
| long-chain-fatty-acid-- ligase acsbg1 | 1 | | | | | o | | | |
| low affinity cationic amino acid transporter 2 | 1 | | o | | | | | | |
| low density lipoprotein receptor adapter protein 1 | 1 | | | | | | | o | |
| low density lipoprotein receptor-related protein 10-like | 1 | o | | | | | | | |
| low quality protein: axin-2-like | 1 | | o | | | | | | |
| low quality protein: calmodulin-binding transcription activator 2-like | 1 | o | | | | | | | |
| low quality protein: ccaat enhancer-binding protein zeta-like | 1 | | | | o | | | | |
| low quality protein: chromodomain-helicase-dna-binding protein 9-like | 1 | | | | | | o | | |
| low quality protein: clip-associating protein 1-like | 1 | | | | | | o | | |
| low quality protein: cytoskeleton-associated protein 5-like | 1 | | | | | | o | | |
| low quality protein: disintegrin and metalloproteinase domain-containing protein 23-like | 1 | | | | | o | | | |
| low quality protein: dnaj homolog subfamily c member 13-like | 1 | | | | o | | | | |
| low quality protein: e3 sumo-protein ligase 2-like | 1 | | | o | | | | | |
| low quality protein: ecotropic viral integration site 5 protein homolog | 1 | o | | | | | | | |
| low quality protein: eukaryotic translation initiation factor 3 subunit a-like | 1 | | | | o | | | | |
| low quality protein: glyceraldehyde-3-phosphate dehydrogenase-like | 1 | | | | o | | | | |
| low quality protein: hephaestin-like | 1 | | | o | | | | | |
| low quality protein: intersectin-2-like | 1 | | | | | | o | | |
| low quality protein: mam and ldl-receptor class a domain-containing protein c10orf112-like | 1 | o | | | | | | | |
| low quality protein: myopalladin-like | 1 | o | | | | | | | |
| low quality protein: myosin-xv | 1 | | | | | | o | | |
| low quality protein: phosphatidate phosphatase lpin1-like | 1 | o | | | | | | | |

| | | | | | | | | | |
|---|---|---|---|---|---|---|---|---|---|
| low quality protein: prostaglandin g h synthase 1-like | 1 | o | | | | | | | |
| low quality protein: protein bat2-like 1-like | 1 | o | | | | | | | |
| low quality protein: protein piccolo-like | 1 | | | | | | | o | |
| low quality protein: protein sgt1-like | 1 | | | | o | | | | |
| low quality protein: sodium leak channel non-selective | 1 | | | | | | o | | |
| low quality protein: spectrin beta brain 2-like | 1 | | o | | | | | | |
| low quality protein: uncharacterized protein kiaa0467-like | 1 | | | | | | o | | |
| low quality protein: upf0378 protein kiaa0100-like | 1 | | | | | | o | | |
| low quality protein: von willebrand factor type egf and pentraxin domain-containing protein 1-like | 1 | | | | o | | | | |
| low quality protein: zinc finger and btb domain-containing protein 4-like | 1 | | | | | | | o | |
| low quality protein: zinc finger protein rlf-like | 1 | o | | | | | | | |
| low-density lipoprotein receptor-related protein 12 | 1 | | | | | | o | | |
| low-density lipoprotein receptor-related protein 1b | 1 | | | | o | | | | |
| low-density lipoprotein receptor-related protein 1b precursor (low-density lipoprotein receptor-related protein-deleted in tumor) (lrp-dit) | 1 | | | | | | o | | |
| lphn1 protein | 1 | | | | | | | | o |
| lps-responsive vesicle beach and anchor containing | 1 | | | | | | o | | |
| lpxtg cell wall surface anchor family protein | 1 | | o | | | | | | |
| lrp1 protein | 1 | o | | | | | | | |
| lrr and pyd domains-containing protein 12 | 1 | o | | | | | | | |
| lrr and pyd domains-containing protein 4 | 1 | o | | | | | | | |
| LRRG00134 [Rattus norvegicus] | 1 | o | | | | | | | |
| l-threonine 3- mitochondrial-like | 1 | | o | | | | | | |
| luc7-like isoform b | 1 | | | | | | o | | |
| lunapark a | 1 | | | | o | | | | |
| lwa_antel ame: full=lwamide neuropeptides contains: ame: full=lwamide i contains: ame: full=metamorphosin a short=mma ame: full=lwamide ii contains: ame: full=lwamide iii contains: ame: full=lwamide iv contains: ame: full=lwamide v contains: ame: full=lwamide vi contains: ame: full=lwamide vii contains: ame: full=lwamide viii contains: ame: full=lwamide ix flags: precursor | 1 | o | o | o | o | o | o | o | o |
| l-xylulose reductase | 1 | o | | | | | | | |
| lymph node homing receptor | 1 | o | | | | | | | |
| lymphocyte cytosolic protein 1 (l-plastin) | 1 | o | | | | | | | |
| lymphocyte-specific protein 1 | 1 | o | | | | | | | |
| lymphocyte-specific protein tyrosine kinase | 1 | o | | | | | | | |
| lymphoid enhancer-binding factor 1 | 1 | | | | | | | o | |
| lymphoid specific | 1 | o | | | | | | | |
| lyr motif-containing protein 1 | 1 | | | | | o | | | |
| lyr motif-containing protein 5a | 1 | | o | | | | | | |
| lysine -specific demethylase a | 1 | o | | | | | | | |
| lysine -specific demethylase b | 1 | o | | | | | | | |
| lysine-specific demethylase 2b-like | 1 | | | | o | | | | |
| lysine-specific demethylase 5c | 1 | o | | | | | | | |
| lysine-specific histone demethylase 1a-like | 1 | | | | | | o | | |

| | | | | | | | | | |
|---|---|---|---|---|---|---|---|---|---|
| lysm and peptidoglycan-binding domain-containing protein 4 | 1 | o | | | | | | | |
| lysocardiolipin acyltransferase 1 | 1 | o | | | | | | | |
| lysophosphatidic acid receptor 1 | 1 | | | o | | | | | |
| lysophosphatidic acid receptor 2 | 1 | | | o | | | | | |
| lysophosphatidic acid receptor 6 | 1 | | | | | | | | o |
| lysophospholipase i | 1 | | | | | o | | | |
| lysophospholipid acyltransferase 5 | 1 | | o | | | | | | |
| lysophospholipid acyltransferase lpcat4 | 1 | | o | | | | | | |
| lysosomal acid phosphatase precursor | 1 | | | | | | | | o |
| lysosomal protein ncu-g1-like | 1 | | | | o | | | | |
| lysosomal thioesterase ppt2-like | 1 | | | | o | | | | |
| lysosomal trafficking regulator-like | 1 | o | | | | | | | |
| lysosome-associated membrane glycoprotein 1 precursor | 1 | | | | | | | | o |
| lysozyme c-like | 1 | | o | | | | | | |
| lysyl hydroxylase | 1 | o | | | | | | | |
| macrophage colony-stimulating factor 1 | 1 | o | | | | | | | |
| macrophage colony-stimulating factor receptor | 1 | o | | | | | | | |
| macrophage stimulating 1 receptor (c-met-related tyrosine kinase) | 1 | | o | | | | | | |
| macrophage-stimulating protein receptor-like | 1 | | o | | | | | | |
| macrosialin precursor | 1 | | o | | | | | | |
| mad homolog 2 | 1 | o | | | | | | | |
| magnesium chelatase subunit h | 1 | | | | | o | | | |
| magnesium transporter mrs2 mitochondrial-like | 1 | o | | | | | | | |
| magnesium transporter nipa2 | 1 | o | | | | | | | |
| magnesium transporter nipa4-like | 1 | | o | | | | | | |
| mago nashi homolog | 1 | | | | | | o | | |
| maguk p55 subfamily member 2 | 1 | | | | | | | | o |
| maguk p55 subfamily member 5 | 1 | | | | o | | | | |
| major facilitator superfamily domain containing 3-like | 1 | o | | | | | | | |
| major facilitator superfamily domain-containing protein 8 | 1 | | o | | | | | | |
| major vault protein | 1 | | o | | | | | | |
| mak10 amino-acid n-acetyltransferase subunit ( cerevisiae) | 1 | | | | o | | | | |
| male-specific lethal 1 homolog | 1 | | | | | | o | | |
| mam domain-containing glycosylphosphatidylinositol anchor protein 1 | 1 | | | | | | | | o |
| manganese-dependent adp-ribose cdp-alcohol diphosphatase | 1 | | | o | | | | | |
| mannose c type 1b | 1 | | | | | | | o | |
| mannose c type 1-like 1 | 1 | o | | | | | | | |
| mannose receptor c type 1-like | 1 | | | | | | | o | |
| mannose-6-phosphate receptor (cation dependent) | 1 | o | | | | | | | |
| mannose-binding 2 | 1 | | | | | | o | | |
| mannose-binding 2-like | 1 | | | | | | | | o |
| mannose-binding lectin-associated serine protease | 1 | | | | o | | | | |
| mannoside acetylglucosaminyltransferase isoform cra_a | 1 | | o | | | | | | |
| mannosyl-oligosaccharide -alpha-mannosidase ia | 1 | o | | | | | | | |

| | | | | | | | | | |
|---|---|---|---|---|---|---|---|---|---|
| map kinase interacting serine threonine kinase 1 | 1 | | | | | | o | | |
| map kinase-activated protein kinase 2-like | 1 | | o | | | | | | |
| map kinase-activating death domain isoform 4 | 1 | | | | | | o | | |
| map kinase-interacting serine threonine-protein kinase 1 | 1 | | | | | | o | | |
| map kinase-interacting serine threonine-protein kinase 2 | 1 | | o | | | | | | |
| map microtubule affinity-regulating kinase 3 | 1 | o | | | | | | | |
| map microtubule affinity-regulating kinase 3-like isoform 1 | 1 | | | | | | | | o |
| map microtubule affinity-regulating kinase 3-like isoform 2 | 1 | | | | | | | | o |
| map3k12-binding inhibitory protein 1 | 1 | o | | | | | | | |
| map-kinase activating death domain-containing isoform 3 | 1 | | | o | | | | | |
| marvel domain-containing protein 2 | 1 | | o | | | | | | |
| mast cell proteinase-3-like | 1 | o | | | | | | | |
| mastermind-like 2 | 1 | o | | | | | | | |
| mastigoneme-like protein | 1 | | o | | | | | | |
| maternal embryonic leucine zipper kinase | 1 | | | | o | | | | |
| mating-type mat1-2 protein | 1 | | | | | | o | | |
| matrin-3 isoform 1 | 1 | | | | o | | | | |
| matrin-3 isoform 2 | 1 | | o | | | | | | |
| matrin-3-like isoform 1 | 1 | o | | | | | | | |
| matrix metallopeptidase 13 (collagenase 3) | 1 | | o | | | | | | |
| matrix metallopeptidase 13 (collagenase 3) precursor | 1 | | | | o | | | | |
| matrix metallopeptidase 16 (membrane-inserted) | 1 | o | | | | | | | |
| matrix metallopeptidase 3 (stromelysin progelatinase) | 1 | | o | | | | | | |
| matrix metalloproteinase 13 | 1 | | o | | | | | | |
| matrix metalloproteinase 19 | 1 | o | | | | | | | |
| matrix metalloproteinase 9 | 1 | | | | o | | | | |
| matrix metalloproteinase-16 | 1 | | | | | | o | | |
| matrix metalloproteinase-18-like | 1 | | o | | | | | | |
| matrix metalloproteinase-24 | 1 | | | | | | | o | |
| matrix-remodelling associated 5 | 1 | o | | | | | | | |
| matrix-remodelling associated 8 | 1 | o | | | | | | | |
| mature t-cell proliferation 1 | 1 | o | | | | | | | |
| max dimerization protein 4 | 1 | o | | | | | | | |
| max gene-associated protein | 1 | o | | | | | | | |
| max gene-associated protein isoform 2 | 1 | | | | | | o | | |
| max interactor 1 | 1 | o | | | | | | | |
| max-like protein x | 1 | o | | | | | | | |
| mbd1 protein | 1 | | | | | | | o | |
| mbt domain containing 1 | 1 | o | | | | | | | |
| mCG1031593 [Mus musculus] | 1 | | | | | o | | | |
| mCG145381 [Mus musculus] | 1 | | | | | | | | o |
| mdm4_danre ame: full=protein mdm4 ame: full=double minute 4 protein ame: full=mdm2-like p53-binding protein ame: full=protein mdmx ame: full=p53-binding protein mdm4 | 1 | | | o | | | | | |
| mechanosensitive ion channel | 1 | | o | | | | | | |
| mechanosensitive ion channel family protein | 1 | | o | | | | | | |
| mediator complex subunit 19 | 1 | | | | o | | | | |
| mediator complex subunit 27 | 1 | | | o | | | | | |

| | | | | | | | | | |
|---|---|---|---|---|---|---|---|---|---|
| mediator of rna polymerase ii transcription subunit 1 | 1 | | | | o | | | | |
| mediator of rna polymerase ii transcription subunit 12 | 1 | | o | | | | | | |
| mediator of rna polymerase ii transcription subunit 12 (thyroid hormone receptor-associated protein complex 230 kda component) (activator-recruited cofactor 240 kda component) (cag repeat protein 45) (opa-containing iso | 1 | o | | | | | | | |
| mediator of rna polymerase ii transcription subunit 19-b | 1 | o | | | | | | | |
| mediator of rna polymerase ii transcription subunit 19-like | 1 | | | | | | o | | |
| mediator of rna polymerase ii transcription subunit 24-like isoform 2 | 1 | | | | | | | o | |
| mediator of rna polymerase ii transcription subunit 30 | 1 | | | o | | | | | |
| mediator of rna polymerase ii transcription subunit 31 | 1 | | | | | | | | o |
| mediator of rna polymerase ii transcription subunit 4-like | 1 | | | | | | | | o |
| mediator of rna polymerase ii transcription subunit 8-like | 1 | | | | | | | | o |
| medium polypeptide | 1 | | | | | | o | | |
| megf10 protein | 1 | | | | | | o | | |
| megf8 partial | 1 | | o | | | | | | |
| melanocortin 2 receptor | 1 | o | | | | | | | |
| melanocyte proliferating gene 1 | 1 | | o | | | | | | |
| melanoma differentiation-associated protein 5 | 1 | | o | | | | | | |
| melanoma inhibitory activity protein 3 | 1 | | | o | | | | | |
| melanoma ubiquitous mutated protein | 1 | | | | | | | o | |
| melatonin receptor 1a | 1 | o | | | | | | | |
| member of ras oncogene family-like 2a | 1 | o | | | | | | | |
| member ras onocogene family | 1 | | | | | o | | | |
| membrane guanylyl cyclase | 1 | | | | | | o | | |
| membrane palmitoylated 55kda | 1 | | o | | | | | | |
| membrane protein mlc1 | 1 | | | | | o | | | |
| membrane-anchored cell surface protein | 1 | | | | | | | | o |
| membrane-associated guanylate ww and pdz domain-containing protein 1 | 1 | | | | | | o | | |
| membrane-associated ring finger 5 | 1 | o | | | | | | | |
| membrane-associated ring finger 8 | 1 | | | | | | o | | |
| membrane-bound transcription factor site 2 | 1 | o | | | | | | | |
| membrane-bound transcription factor site-1 protease | 1 | o | | | | | | | |
| membrane-spanning 4-domains subfamily a member 12 | 1 | | | o | | | | | |
| membrane-spanning 4-domains subfamily a member 4a | 1 | | | | | | o | | |
| membrane-spanning 4-domains subfamily a member 8a | 1 | | | | | | | o | |
| meningioma expressed antigen 5 isoform 1 | 1 | | o | | | | | | |
| meningioma expressed antigen 5 isoform 2 | 1 | o | | | | | | | |
| meprin 1 beta | 1 | | | | | | | o | |
| meprin alpha (paba peptide hydrolase) | 1 | | | o | | | | | |
| meprin beta | 1 | | | | | | | o | |
| merozoite surface antigen 2 | 1 | o | | | | | | | |
| mesenchyme homeobox 1 | 1 | o | | | | | | | |
| mesenchyme homeobox 2 | 1 | o | | | | | | | |

| | | | | | | | | | |
|---|---|---|---|---|---|---|---|---|---|
| mesothelin isoform 1 preproprotein | 1 | | | | | | | | o |
| metabotropic glutamate receptor 2 | 1 | | | | | | | | o |
| metabotropic glutamate receptor 5 splice isoform 2 | 1 | | | o | | | | | |
| metabotropic glutamate receptor 5-like | 1 | | | | | | o | | |
| metabotropic glutamate receptor 8 | 1 | | | | | | | | o |
| metal transporter cnnm2 | 1 | | | | | | o | | |
| metallo-beta-lactamase domain-containing protein 1 | 1 | o | | | | | | | |
| metallo-beta-lactamase family protein | 1 | | o | | | | | | |
| metalloendopeptidase homolog pex | 1 | | o | | | | | | |
| metallophosphoesterase domain containing 1 | 1 | | | | | | | o | |
| metalloreductase steap2 | 1 | | | | | | | | o |
| metalloreductase steap3-like | 1 | | | | | | | | o |
| metal-response element-binding transcription factor 2 | 1 | o | | | | | | | |
| metastasis associated 1 | 1 | | o | | | | | | |
| metastasis suppressor 1-like | 1 | | | | | o | | | |
| metastasis suppressor isoform cra_d | 1 | | | o | | | | | |
| metastasis-associated protein mta1-like | 1 | | o | | | | | | |
| metastasis-associated protein mta2 | 1 | | o | | | | | | |
| methionine aminopeptidase 1d | 1 | | | | | o | | | |
| methionine aminopeptidase 2-like | 1 | o | | | | | | | |
| methionine aminopeptidase mitochondrial precursor | 1 | o | | | | | | | |
| methionine-trna synthetase 2 | 1 | | | | | o | | | |
| methionyl-trna mitochondrial-like | 1 | | | | | | | | o |
| methyl- binding domain protein 1 | 1 | o | | | | | | | |
| methyl- binding domain protein 5 | 1 | o | | | | | | | |
| methylcrotonoyl- carboxylase beta mitochondrial | 1 | | | | o | | | | |
| methylcrotonoyl-coenzyme a carboxylase 1 | 1 | | o | | | | | | |
| methylenetetrahydrofolate reductase | 1 | | | | | | | | o |
| methylglutaconyl- mitochondrial-like | 1 | | | | | | | | o |
| methylmalonic aciduria (cobalamin deficiency) cblc with homocystinuria | 1 | o | | | | | | | |
| methylmalonic aciduria and homocystinuria type c protein homolog | 1 | o | | | | | | | |
| methylthioadenosine phosphorylase | 1 | | | | | | | o | |
| methyltransferase family member 1 | 1 | | | o | | | | | |
| methyltransferase like 2a | 1 | o | | | | | | | |
| methyltransferase mb3374 | 1 | | | | | o | | | |
| methyltransferase nsun6-like | 1 | | o | | | | | | |
| methyltransferase type 11 | 1 | o | | | | | | | |
| methyltransferase wbscr22 | 1 | | | | | | o | | |
| methyltransferase-like protein 1 | 1 | o | | | | | | | |
| methyltransferase-like protein 7a precursor | 1 | | | | o | | | | |
| methyltransferase-like protein 9-like | 1 | o | | | | | | | |
| methyltransferase-like protein mitochondrial-like | 1 | | | | o | | | | |
| metrn protein | 1 | | | | o | | | | |
| mevalonate decarboxylase | 1 | | | | | | o | | |
| mgc11257 protein | 1 | o | | | | | | | |
| mgc114910 protein | 1 | o | | | | | | | |
| mgc115312 protein | 1 | | | | | | | o | |
| mgc137510 protein | 1 | | o | | | | | | |
| mgc147117 protein | 1 | o | | | | | | | |
| mgc4836 protein | 1 | | | | | | o | | |
| mgc53818 protein | 1 | | o | | | | | | |
| mgc64596 protein | 1 | | | | | o | | | |

| | | | | | | | | | |
|---|---|---|---|---|---|---|---|---|---|
| mgc68650 protein | 1 | | | | o | | | | |
| mgc79469 protein | 1 | | | | | | | | o |
| mgc80389 protein | 1 | | | | | o | | | |
| mgc80500 protein | 1 | | | | | | o | | |
| mgc80644 protein | 1 | | | o | | | | | |
| mgc81615 protein | 1 | o | | | | | | | |
| mgc82365 protein | 1 | | | o | | | | | |
| mgc84302 protein | 1 | | | | | | o | | |
| mhc class ia antigen | 1 | | | | | | | o | |
| mhc class ii alpha antigen | 1 | | | | | | o | | |
| mhc class ii beta antigen | 1 | | | | | | | o | |
| mhc class iia antigen | 1 | o | | | | | | | |
| mib2 protein | 1 | | o | | | | | | |
| microfibrillar-associated protein 3-like precursor | 1 | | | | | | | o | |
| microphthalmia-associated transcription factor | 1 | | | | | o | | | |
| microtubule associated calponin and lim domain containing 1 | 1 | o | | | | | | | |
| microtubule associated calponin and lim domain containing 2 | 1 | o | | | | | | | |
| microtubule associated calponin and lim domain containing isoform cra_a | 1 | o | | | | | | | |
| microtubule associated serine threonine kinase 3 | 1 | | | | | | o | | |
| microtubule-associated protein 1 light chain 3 alpha | 1 | | | | | | o | | |
| microtubule-associated protein 1s | 1 | | | | | | | o | |
| microtubule-associated protein 2 isoform 1 isoform 4 | 1 | | | | | | o | | |
| microtubule-associated protein 2-like | 1 | | | | | | o | | |
| microtubule-associated protein 4 | 1 | | | | | | o | | |
| microtubule-associated protein 7 domain containing 1 | 1 | o | | | | | | | |
| microtubule-associated protein 7-like | 1 | | | | | | | o | |
| microtubule-associated protein 9 | 1 | | | | | | o | | |
| microtubule-associated protein homolog | 1 | | o | | | | | | |
| microtubule-associated protein tau-like | 1 | | | | | | | o | |
| microtubule-associated serine threonine-protein kinase 1 | 1 | | | | | | | o | |
| microtubule-associated serine threonine-protein kinase 2-like | 1 | | | | | | o | | |
| microtubule-associated serine threonine-protein kinase 3 | 1 | | | | | | | | o |
| microtubule-associated serine threonine-protein kinase 4 | 1 | | | | | | o | | |
| microtubule-associated serine threonine-protein kinase 4-like | 1 | | | | | | | o | |
| microtubule-associated tumor suppressor 1 homolog | 1 | | o | | | | | | |
| microtubule-associated tumor suppressor 1 homolog a-like | 1 | | | | | | | | o |
| midgut cysteine | 1 | | | o | | | | | |
| midkine | 1 | | | | | o | | | |
| midline-1 isoform 1 | 1 | o | | | | | | | |
| midnolin | 1 | | | o | | | | | |
| milk fat globule-egf factor 8 protein | 1 | o | | | | | | | |
| mindbomb homolog 1 | 1 | | o | | | | | | |
| mineralocorticoid receptor | 1 | | | | | o | | | |

| | | | | | | | | | |
|---|---|---|---|---|---|---|---|---|---|
| minichromosome maintenance complex component 3 associated protein | 1 | o | | | | | | | |
| minus agglutinin | 1 | o | | | | | | | |
| misshapen-like kinase 1 | 1 | | o | | | | | | |
| mit domain-containing protein 1 | 1 | o | | | | | | | |
| mitochondria antiviral signalling protein | 1 | | | o | | | | | |
| mitochondrial 28s ribosomal protein mitochondrial precursor | 1 | | o | | | | | | |
| mitochondrial 28s ribosomal protein s29 | 1 | | | | | | o | | |
| mitochondrial 39s ribosomal protein l33 | 1 | | o | | | | | | |
| mitochondrial aspartate aminotransferase | 1 | | | | | | | | o |
| mitochondrial atp synthase subunit gamma | 1 | | | | | | | | o |
| mitochondrial carnitine palmitoyltransferase i alpha1a | 1 | | | | | | | | o |
| mitochondrial carrier protein flj44862-like | 1 | | o | | | | | | |
| mitochondrial dimethyladenosine transferase 1 | 1 | o | | | | | | | |
| mitochondrial dynamin-like 120 kda protein | 1 | o | | | | | | | |
| mitochondrial fission factor | 1 | | | | | | o | | |
| mitochondrial glutamate carrier 1-like | 1 | | | | o | | | | |
| mitochondrial h+-transporting atp synthase f1 complex beta polypeptide | 1 | | | | | | o | | |
| mitochondrial import inner membrane translocase subunit tim10 | 1 | | | | o | | | | |
| mitochondrial import inner membrane translocase subunit tim14 | 1 | | | | | | | o | |
| mitochondrial import inner membrane translocase subunit tim22 | 1 | | | | | | o | | |
| mitochondrial import receptor subunit tom70 | 1 | | | | | | o | | |
| mitochondrial inner membrane protease subunit 1 | 1 | o | | | | | | | |
| mitochondrial inner membrane protease subunit 2 | 1 | | | | | o | | | |
| mitochondrial inner membrane protein | 1 | | o | | | | | | |
| mitochondrial inner membrane protein oxa1l | 1 | o | | | | | | | |
| mitochondrial methionyl-trna formyltransferase | 1 | o | | | | | | | |
| mitochondrial ornithine transporter 1 | 1 | | o | | | | | | |
| mitochondrial protein 18 kda | 1 | o | | | | | | | |
| mitochondrial rho gtpase 1 | 1 | | | | | | | o | |
| mitochondrial rho gtpase 1-like | 1 | o | | | | | | | |
| mitochondrial rho gtpase 2 | 1 | | o | | | | | | |
| mitochondrial ribosomal protein l23 | 1 | | | | | | | | o |
| mitochondrial ribosomal protein l27 isoform cra_b | 1 | | | | | | | | o |
| mitochondrial ribosomal protein l28-like | 1 | o | | | | | | | |
| mitochondrial ribosomal protein l33 | 1 | | | | | o | | | |
| mitochondrial ribosomal protein l34 | 1 | o | | | | | | | |
| mitochondrial ribosomal protein l36 | 1 | | o | | | | | | |
| mitochondrial ribosomal protein l39 | 1 | | | | | | o | | |
| mitochondrial ribosomal protein l4 | 1 | o | | | | | | | |
| mitochondrial ribosomal protein l43 | 1 | | | | o | | | | |
| mitochondrial ribosomal protein l9 | 1 | o | | | | | | | |
| mitochondrial ribosomal protein s11 | 1 | | o | | | | | | |
| mitochondrial ribosomal protein s12 | 1 | | o | | | | | | |
| mitochondrial ribosomal protein s30 | 1 | o | | | | | | | |
| mitochondrial ribosomal protein s34 | 1 | | o | | | | | | |
| mitochondrial ribosomal protein s35 | 1 | | o | | | | | | |
| mitochondrial ribosomal protein subunit rtc6 | 1 | o | | | | | | | |
| mitochondrial succinyl- ligase | 1 | o | | | | | | | |

| | | | | | | | | | |
|---|---|---|---|---|---|---|---|---|---|
| mitochondrial translational initiation factor 2 | 1 | o | | | | | | | |
| mitochondrial translational initiation factor isoform cra_a | 1 | | | | | | | o | |
| mitochondrial ubiquitin ligase activator of nfkb 1 | 1 | | | | | | | | o |
| mitochondrial ubiquitin ligase activator of nfkb 1-like | 1 | | o | | | | | | |
| mitochondrial-like | 1 | | | | o | | | | |
| mitochondrial-processing peptidase subunit alpha | 1 | | | | o | | | | |
| mitofusin 1 | 1 | o | | | | | | | |
| mitogen activated protein kinase 9 | 1 | o | | | | | | | |
| mitogen-activated protein kinase 10-like | 1 | | | | | | | o | |
| mitogen-activated protein kinase 11 | 1 | | | | | | | o | |
| mitogen-activated protein kinase 14 isoform 1 | 1 | | o | | | | | | |
| mitogen-activated protein kinase 15 | 1 | | | o | | | | | |
| mitogen-activated protein kinase 7 | 1 | | | | | o | | | |
| mitogen-activated protein kinase 8 interacting protein 3 | 1 | o | | | | | | | |
| mitogen-activated protein kinase 8 isoform 1 | 1 | | o | | | | | | |
| mitogen-activated protein kinase associated protein 1 | 1 | o | | | | | | | |
| mitogen-activated protein kinase isoform cra_a | 1 | | | | | | | o | |
| mitogen-activated protein kinase kinase 6 | 1 | o | | | | | | | |
| mitogen-activated protein kinase kinase kinase 10 | 1 | o | | | | | | | |
| mitogen-activated protein kinase kinase kinase 7 interacting protein 3 | 1 | | o | | | | | | |
| mitogen-activated protein kinase kinase kinase kinase 2 | 1 | o | | | | | | | |
| mitogen-activated protein kinase kinase kinase kinase 4-like | 1 | | | | | | | o | |
| mitogen-activated protein kinase-activated protein kinase 5 | 1 | o | | | | | | | |
| mitogen-activated protein kinase-binding protein 1 isoform 1 | 1 | | | | | | o | | |
| mitotic spindle-associated mmxd complex subunit mip18 | 1 | | | | | | | | o |
| mixed lineage kinase domain-like | 1 | o | | | | | | | |
| mj0042 family finger-like protein | 1 | | | | | | o | | |
| mkiaa0363 protein | 1 | | | | | | o | | |
| mkiaa0664 protein | 1 | | | | | o | | | |
| mkiaa1506 protein | 1 | | | | | | o | | |
| mlf1 interacting protein | 1 | o | | | | | | | |
| mln64 n-terminal domain homolog | 1 | | o | | | | | | |
| mlx interacting | 1 | | o | | | | | | |
| mms4 protein | 1 | | | | | | | o | |
| moloney leukemia virus homolog | 1 | o | | | | | | | |
| molybdenum cofactor sulfurase | 1 | o | | | | | | | |
| molybdenum cofactor synthesis 1 | 1 | | o | | | | | | |
| molybdenum cofactor synthesis 2 | 1 | o | | | | | | | |
| molybdenum cofactor synthesis 3 | 1 | | o | | | | | | |
| molybdenum cofactor synthesis protein 2 large subunit | 1 | | o | | | | | | |
| molybdopterin synthase small subunit mocs2a | 1 | | | o | | | | | |
| monoacylglycerol acyltransferase 1 | 1 | | o | | | | | | |
| monoacylglycerol o-acyltransferase 2 | 1 | | o | | | | | | |
| monoacylglycerol o-acyltransferase 3-like | 1 | | | o | | | | | |

| | | | | | | | | | |
|---|---|---|---|---|---|---|---|---|---|
| monocarboxylate transporter 1 | 1 | | o | | | | | | |
| monocarboxylate transporter 2 | 1 | | o | | | | | | |
| morc family cw-type zinc finger protein 2 | 1 | | | | o | | | | |
| morc family cw-type zinc finger protein 3 | 1 | | o | | | | | | |
| morc family cw-type zinc finger protein 4 | 1 | o | | | | | | | |
| motile sperm domain containing 1 | 1 | | | | | | | | o |
| m-phase inducer phosphatase 2-like | 1 | | | | o | | | | |
| m-phase phosphoprotein 1 | 1 | o | | | | | | | |
| mps one binder kinase activator-like 2 | 1 | o | | | | | | | |
| mrna turnover deadenylation component (pop2 homologue) | 1 | | | | | o | | | |
| mrna-capping enzyme | 1 | | | | | | | | o |
| mrna-decapping enzyme 1a | 1 | | | | o | | | | |
| msh2 protein | 1 | o | | | | | | | |
| mslc9a3r2 e3karp sip-1 tka-1 octs2 | 1 | | | | | | o | | |
| msug1 protein | 1 | | | o | | | | | |
| mterf domain containing 2 | 1 | o | | | | | | | |
| mterf domain-containing protein mitochondrial-like | 1 | | | | o | | | | |
| mucin 11a | 1 | | | | o | | | | |
| mucin 30e | 1 | o | | | | | | | |
| mucin apoprotein | 1 | | | | o | | | | |
| mucin-7 precursor | 1 | o | | | | | | | |
| mucin-associated surface protein | 1 | | | | o | | | | |
| mucolipin 1 | 1 | | | o | | | | | |
| mucosa associated lymphoid tissue lymphoma translocation gene 1-like | 1 | o | | | | | | | |
| mucosa associated lymphoid tissue lymphoma translocation gene isoform cra_b | 1 | | o | | | | | | |
| multidrug and toxin extrusion protein 2 | 1 | | o | | | | | | |
| multidrug and toxin extrusion protein 2-like | 1 | | o | | | | | | |
| multidrug resistance associated protein 2 | 1 | | o | | | | | | |
| multidrug resistance-associated protein 4 | 1 | | | | | | | | o |
| multifunctional chaperone | 1 | | | o | | | | | |
| multifunctional expression regulator | 1 | | o | | | | | | |
| multiple coagulation factor deficiency 2 | 1 | o | | | | | | | |
| multiple epidermal growth factor-like domains protein 8-like | 1 | o | | | | | | | |
| muscarinic acetylcholine receptor m4 | 1 | | | | | | o | | |
| muscle segment homeobox c | 1 | | | | | | o | | |
| muscleblind-like 1 isoform 4 | 1 | o | | | | | | | |
| muscleblind-like 1 isoform 5 | 1 | o | | | | | | | |
| muscle-restricted dual specificity phosphatase | 1 | o | | | | | | | |
| muscle-type creatine kinase ckm1 | 1 | o | | | | | | | |
| musculoskeletal embryonic nuclear protein 1 | 1 | o | | | | | | | |
| muts homolog 6 ( coli) | 1 | o | | | | | | | |
| myb and hsa domain-containing protein | 1 | o | | | | | | | |
| myb- swirm and mpn domains 1 | 1 | | o | | | | | | |
| myb-binding protein 1a-like | 1 | | | | o | | | | |
| myc box-dependent-interacting protein 1-like | 1 | | | | o | | | | |
| myc target protein 1 homolog | 1 | o | | | | | | | |
| myelin expression factor 2 | 1 | | o | | | | | | |
| myelin gene regulatory factor-like | 1 | | | | | | o | | |
| myelin protein zero-like 1 | 1 | | | o | | | | | |
| myelin protein zero-like 3 | 1 | | | o | | | | | |
| myelin transcription factor 1-like | 1 | | | | o | | | | |
| myeloid cell leukemia 1 | 1 | | o | | | | | | |
| myeloid differentiation factor 88 | 1 | o | | | | | | | |
| myeloid ecotropic viral integration 1 | 1 | o | | | | | | | |

| | | | | | | | | | |
|---|---|---|---|---|---|---|---|---|---|
| myeloid leukemia factor 2 | 1 | | o | | | | | | |
| myeloid lymphoid or mixed-lineage leukemia | 1 | o | | | | | | | |
| myeloid lymphoid or mixed-lineage leukemia 3 | 1 | | | | | | | o | |
| myocardin | 1 | o | | | | | | | |
| myocardin-like protein | 1 | o | | | | | | | |
| myocardin-related transcription factor b-like | 1 | | | | | | o | | |
| myocilin | 1 | o | | | | | | | |
| myof protein | 1 | o | | | | | | | |
| myogenic factor 1 | 1 | o | | | | | | | |
| myogenin | 1 | o | | | | | | | |
| myo-inositol-1 phosphate synthase-like | 1 | | | | | | | | o |
| myomesin-1 isoform 1 | 1 | | o | | | | | | |
| myosin binding protein fast-type | 1 | o | | | | | | | |
| myosin binding protein h-like | 1 | o | | | | | | | |
| myosin heavy skeletal adult | 1 | o | | | | | | | |
| myosin if | 1 | o | | | | | | | |
| myosin ixa | 1 | o | | | | | | | |
| myosin ixa-like 1 | 1 | o | | | | | | | |
| myosin ixb | 1 | | o | | | | | | |
| myosin light chain 1 | 1 | o | | | | | | | |
| myosin phosphatase rho-interacting protein | 1 | | | | | | o | | |
| myosin regulatory light chain mrcl3 | 1 | | | | | | | | o |
| myosin va (heavy chain myoxin) | 1 | o | | | | | | | |
| myosin xix | 1 | | | | o | | | | |
| myosin-9 (myosin heavy nonmuscle iia) (nonmuscle myosin heavy chain iia) (nmmhc ii-a) (nmmhc-iia) (cellular myosin heavy type a) (nonmuscle myosin heavy chain-a) (nmmhc-a) partial | 1 | | | | o | | | | |
| myosin-binding protein slow-type-like | 1 | o | | | | | | | |
| myotilin | 1 | o | | | | | | | |
| myotubularin | 1 | o | | | | | | | |
| myotubularin 1 | 1 | o | | | | | | | |
| myotubularin related protein 6 | 1 | o | | | | | | | |
| myotubularin-related protein 3 | 1 | o | | | | | | | |
| myotubularin-related protein 3-like | 1 | | | o | | | | | |
| myst histone acetyltransferase (monocytic leukemia) 4 | 1 | o | | | | | | | |
| n -acetyltransferase auxiliary subunit | 1 | | o | | | | | | |
| na+ k+ alpha polypeptide | 1 | | | o | | | | | |
| na+ k+ beta 2 polypeptide | 1 | | | | | o | | | |
| na+ k+ beta 2b polypeptide | 1 | | | | | | o | | |
| n-acetyl-d-glucosamine kinase | 1 | | | | | | | | o |
| n-acetylgalactosamine kinase | 1 | | | | | | | | o |
| n-acetylglucosamine-1-phosphotransferase subunit gamma precursor | 1 | o | | | | | | | |
| n-acetylglucosamine-6-sulfatase precursor | 1 | | | | | | o | | |
| n-acetylglucosaminyltransferase i | 1 | o | | | | | | | |
| n-acetylglutamate synthase | 1 | o | | | | | | | |
| n-acetylneuraminate pyruvate lyase- partial | 1 | o | | | | | | | |
| n-acetyltransferase 13 | 1 | | | | | | | o | |
| n-acetyltransferase 14 | 1 | | | | | | | | o |
| n-acetyltransferase 8-like protein | 1 | | | | | | | o | |
| n-acylsphingosine amidohydrolase | 1 | | o | | | | | | |
| nad -dependent steroid dehydrogenase-like | 1 | | | | | | o | | |
| nad h quinone 1 | 1 | o | | | | | | | |
| nad-dependent adp-ribosyltransferase sirtuin-4 | 1 | o | | | | | | | |

| | | | | | | | | | |
|---|---|---|---|---|---|---|---|---|---|
| nad-dependent adp-ribosyltransferase sirtuin-4-like | 1 | | | | o | | | | |
| nad-dependent deacetylase sirtuin-mitochondrial-like | 1 | | | | o | | | | |
| nad-dependent deacetylase sirtuin-2 | 1 | o | | | | | | | |
| nad-dependent deacetylase sirtuin-6 | 1 | | o | | | | | | |
| nadh dehydrogenase 1 alpha assembly factor 1 | 1 | o | | | | | | | |
| nadh dehydrogenase 1 alpha subcomplex subunit 2 | 1 | | o | | | | | | |
| nadh dehydrogenase fe-s protein 2 | 1 | | | | | | | o | |
| nadh dehydrogenase fe-s protein 49kda (nadh-coenzyme q reductase) | 1 | | | | | o | | | |
| nadh dehydrogenase fe-s protein isoform cra_a | 1 | o | | | | | | | |
| nadh dehydrogenase iron-sulfur protein 7 | 1 | | o | | | | | | |
| nadh-ubiquinone oxidoreductase 75 kda mitochondrial-like isoform 2 | 1 | | | | o | | | | |
| nadp-dependent malic mitochondrial precursor | 1 | | o | | | | | | |
| nadph:adrenodoxin mitochondrial-like | 1 | | o | | | | | | |
| n-alpha-acetyltransferase catalytic subunit-like | 1 | | | | | | o | | |
| nanos homolog 1 | 1 | o | | | | | | | |
| narg2 protein | 1 | | | | o | | | | |
| nascent polypeptide-associated complex subunit alpha isoform a | 1 | o | | | | | | | |
| nat12 protein | 1 | o | | | | | | | |
| natriuretic peptide receptor 2 | 1 | | | | o | | | | |
| ncl protein | 1 | | | | | | | | o |
| ncln protein | 1 | | | | | | | o | |
| ndrg family member 2 | 1 | o | | | | | | | |
| ndrg family member 3 | 1 | | o | | | | | | |
| ndrg family member 4 | 1 | | | | | | | o | |
| nebulin isoform 1 | 1 | o | | | | | | | |
| nebulin-related-anchoring protein | 1 | | o | | | | | | |
| necap endocytosis associated 2 | 1 | | o | | | | | | |
| nedd4-binding protein 1-like | 1 | o | | | | | | | |
| negative elongation factor b | 1 | | | | | | | | o |
| negative elongation factor d | 1 | | | | | | | | o |
| nel-like 2 | 1 | | | | | | o | | |
| nemo-like kinase | 1 | o | | | | | | | |
| neogenin isoform 1 | 1 | | | o | | | | | |
| neogenin- partial | 1 | | o | | | | | | |
| neoverrucotoxin subunit beta-like | 1 | | | | | | o | | |
| neprilysin | 1 | o | | | | | | | |
| n-ethylmaleimide-sensitive factor | 1 | | o | | | | | | |
| n-ethylmaleimide-sensitive factor attachment gamma | 1 | | o | | | | | | |
| netrin 1 | 1 | o | | | | | | | |
| netrin 1a | 1 | o | | | | | | | |
| netrin-4-like | 1 | | | | | | | | o |
| neudesin precursor | 1 | | | | | o | | | |
| neural adhesion molecule | 1 | | | | | | o | | |
| neural cell adhesion molecule 1 | 1 | | | | | | o | | |
| neural cell adhesion molecule 1 isoform 2 | 1 | | | | | | | o | |
| neural cell adhesion molecule 1 precursor | 1 | | | | | | | | o |
| neural differentiation and 1 | 1 | | | | | | o | | |
| neural wiskott-aldrich syndrome protein | 1 | | | | | o | | | |
| neural-cadherin-like | 1 | | | | | | o | | |

| | | | | | | | | | |
|---|---|---|---|---|---|---|---|---|---|
| neuregulin 1 variant iv-beta 1a | 1 | | | | | | o | | |
| neurexin 1 beta | 1 | | | | | | o | | |
| neurexin 3 | 1 | | | | | | | o | |
| neurexin 3a alpha | 1 | | | | | | o | | |
| neurexin 3a- partial | 1 | | | | | | | o | |
| neurexin iii-alpha | 1 | | | | | | o | | |
| neurexin-1-alpha isoform 2 | 1 | | | | | | o | | |
| neurexin-1-alpha-like isoform 2 | 1 | | | | | | o | | |
| neurexin-3-alpha isoform 2 | 1 | | | | | | | | o |
| neurexophilin 1 | 1 | | | | | | | o | |
| neurexophilin 2 | 1 | | | | | o | | | |
| neuritin 1-like | 1 | | | | | | | o | |
| neurobeachin isoform 2 | 1 | | | | | | o | | |
| neuroblastoma suppressor of tumorigenicity 1 precursor | 1 | | | | | o | | | |
| neuroepithelial cell transforming 1 | 1 | | | | | | o | | |
| neurofascin isoform 4 precursor | 1 | | | | | | o | | |
| neurofibromin 1 | 1 | | | o | | | | | |
| neurogenic differentiation factor 1 | 1 | | | | | | | | o |
| neurogenic locus notch homolog protein 2-like | 1 | | | | | | o | | |
| neuroligin 3a | 1 | | | | | | o | | |
| neuroligin 4a | 1 | | | | | | o | | |
| neurolin-like cell adhesion molecule | 1 | | | | | o | | | |
| neuromedin b | 1 | | | | | o | | | |
| neuronal acetylcholine receptor subunit alpha-2-like | 1 | | | | | | | | o |
| neuronal acetylcholine receptor subunit alpha-4 | 1 | o | | | | | | | |
| neuronal cell adhesion molecule | 1 | | | | | | o | | |
| neuronal cell adhesion molecule isoform 1 | 1 | | | | | o | | | |
| neuronal membrane glycoprotein m6-b isoform 1 | 1 | o | | | | | | | |
| neuronal nitric oxide synthase | 1 | | | | | | o | | |
| neuronal pentraxin ii precursor (np-ii) (neuronal activity-regulated pentraxin) | 1 | o | | | | | | | |
| neuronal pentraxin-like protein c16orf38-like | 1 | o | | | | | | | |
| neuro-oncological ventral antigen 1 | 1 | | | | | | o | | |
| neuropeptide y peptide yy receptor yb | 1 | | | | | | | o | |
| neuropeptide y receptor type 2-like | 1 | | | | | | o | | |
| neuropeptide y receptor type 6 (npy6-r) (pancreatic polypeptide receptor 2) | 1 | o | | | | | | | |
| neuropeptide-like protein c4orf48 homolog | 1 | | | | | | | | o |
| neuropilin and tolloid-like protein 2 | 1 | | | | | | | | o |
| neuropilin-2 precursor | 1 | | | | | | o | | |
| neuroserpin | 1 | | | | | | | o | |
| neurotrophic tyrosine type 3 isoform 1 | 1 | | | | | o | | | |
| neurotrophic tyrosine type 3 isoform 2 | 1 | | | | | | | o | |
| neutral sphingomyelinase (n-smase) activation associated factor | 1 | | | o | | | | | |
| nfatc2ip protein | 1 | | | | | | | | o |
| nf-kappa-b inhibitor-interacting ras-like protein 2 | 1 | | o | | | | | | |
| nf-kappab repressing factor | 1 | | o | | | | | | |
| nf-kappa-b-activating protein | 1 | | | | o | | | | |
| nfkb activating protein | 1 | | | o | | | | | |
| nfkb inhibitor interacting ras-like 2 | 1 | o | | | | | | | |
| nfx1-type zinc finger-containing protein 1 | 1 | | | o | | | | | |
| ngdn protein | 1 | | o | | | | | | |
| n-glycosylase dna lyase | 1 | o | | | | | | | |
| NHE8 [Takifugu obscurus] | 1 | | | | | | o | | |

| | | | | | | | | | |
|---|---|---|---|---|---|---|---|---|---|
| nibrin | 1 | o | | | | | | | |
| nicastrin | 1 | o | | | | | | | |
| nicotinamide nucleotide adenylyltransferase 3 | 1 | o | | | | | | | |
| nicotinamide riboside kinase 1 | 1 | | o | | | | | | |
| nicotinate phosphoribosyltransferase-like protein | 1 | | o | | | | | | |
| niemann-pick type c1 | 1 | | o | | | | | | |
| nilt1 leukocyte receptor-like | 1 | o | | | | | | | |
| nilt2 leucocyte receptor | 1 | o | | | | | | | |
| nima (never in mitosis gene a)-related kinase 3 | 1 | | | o | | | | | |
| nima (never in mitosis gene a)-related kinase 6 | 1 | o | | | | | | | |
| nima-related kinase 8 | 1 | | | | | | o | | |
| nima-related kinase 8-like | 1 | o | | | | | | | |
| nipa-like domain containing 2 | 1 | | | | | | | o | |
| nipa-like protein 3-like | 1 | | | | | | | | o |
| nipsnap homolog 3a ( elegans) | 1 | | o | | | | | | |
| nipsnap homolog 3b ( elegans) | 1 | | o | | | | | | |
| nitric oxide synthase 1 adaptor protein | 1 | o | | | | | | | |
| nitrilase 1 | 1 | | o | | | | | | |
| nitrogen fixation protein | 1 | | | | | | | | o |
| nk2 homeobox 1a | 1 | | | | | | o | | |
| nlr card domain containing 3 | 1 | o | | | | | | | |
| nlr pyrin domain containing 12 | 1 | | | o | | | | | |
| nlr pyrin domain containing 6-like | 1 | o | | | | | | | |
| n-lysine methyltransferase setd8-like | 1 | | | | o | | | | |
| nmda receptor nr2c subunit | 1 | | | | | o | | | |
| n-methyl-d-aspartate receptor | 1 | | | | | | o | | |
| n-methyl-d-aspartate receptor-associated isoform c | 1 | | | o | | | | | |
| n-myc downstream regulated gene 4 | 1 | | | | | | o | | |
| nodal modulator 1 | 1 | | | | | | o | | |
| noggin 4 | 1 | o | | | | | | | |
| nol1 nop2 sun domain member 4 | 1 | | | | o | | | | |
| nol1 nop2 sun domain member 6 | 1 | | o | | | | | | |
| non imprinted in prader-willi angelman syndrome 2 | 1 | | o | | | | | | |
| non-erythrocytic 4 | 1 | | | | | | o | | |
| non-metastatic cells protein expressed in (nucleoside-diphosphate kinase) | 1 | o | | | | | | | |
| non-muscle caldesmon-like | 1 | | | | o | | | | |
| non-smc condensin i subunit h | 1 | o | | | | | | | |
| non-smc condensin ii subunit d3 | 1 | | o | | | | | | |
| nonspecific cytotoxic cell receptor protein 1 | 1 | | | | | | | o | |
| non-structural maintenance of chromosomes element 4 homolog a | 1 | o | | | | | | | |
| nonstructural replicase | 1 | | | | o | | | | |
| nop14 nucleolar protein homolog | 1 | o | | | | | | | |
| nop16 nucleolar protein homolog | 1 | | | | | | o | | |
| notch 1 | 1 | o | | | | | | | |
| notch homolog 2 | 1 | o | | | | | | | |
| notch homolog translocation-associated | 1 | o | | | | | | | |
| notch homologue 3 | 1 | o | | | | | | | |
| notch signaling pathway homolog family member (nsh-1)-like | 1 | o | | | | | | | |
| notchless protein homolog 1 | 1 | | | | o | | | | |
| novel 7 transmembrane receptor (metabotropic glutamate family) protein | 1 | | | o | | | | | |
| novel calcium activated pottassium channel protein | 1 | | | | | | | o | |
| novel immune-type receptor 1 | 1 | | | | | | o | | |

| | | | | | | | | | |
|---|---|---|---|---|---|---|---|---|---|
| novel immune-type receptor 17 | 1 | o | | | | | | | |
| novel immune-type receptor 2 | 1 | o | | | | | | | |
| novel immune-type receptor 4 | 1 | o | | | | | | | |
| novel immune-type receptor allele 4 | 1 | o | | | | | | | |
| novel immunoglobulin i-set domain containing protein | 1 | o | | | | | | | |
| novel lectin c-type domain containing partial | 1 | o | | | | | | | |
| novel pentaxin family domain containing protein | 1 | | o | | | | | | |
| novel protein (wu:fd18f09) | 1 | o | | | | | | | |
| novel protein (zgc:111954) | 1 | o | | | | | | | |
| novel protein (zgc:113360) | 1 | | | o | | | | | |
| novel protein (zgc:152873) | 1 | | | | | | o | | |
| novel protein (zgc:153058) | 1 | | | | o | | | | |
| novel protein (zgc:55292) | 1 | o | | | | | | | |
| novel protein (zgc:55390) | 1 | o | | | | | | | |
| novel protein (zgc:55794) | 1 | o | | | | | | | |
| novel protein (zgc:63021) | 1 | | | | | o | | | |
| novel protein (zgc:65774) | 1 | | o | | | | | | |
| novel protein (zgc:86860) | 1 | | | o | | | | | |
| novel protein (zgc:92107) | 1 | o | | | | | | | |
| novel protein (zgc:92789) | 1 | | | | | o | | | |
| novel protein (zgc:92854) | 1 | | | | | | | | o |
| novel protein (zgc:92871) | 1 | | o | | | | | | |
| novel protein ankyrin repeat domain 47 | 1 | o | | | | | | | |
| novel protein beta-carotene oxygenase 2a | 1 | | o | | | | | | |
| novel protein choline kinase family protein | 1 | | | | | | o | | |
| novel protein chromosome 1 open reading frame 156 | 1 | o | | | | | | | |
| novel protein cmrf-35-like molecule 3 | 1 | o | | | | | | | |
| novel protein containing a b-box zinc finger domain | 1 | | | o | | | | | |
| novel protein containing a trypsin domain | 1 | o | | | | | | | |
| novel protein containing an inosine-uridine preferring nucleoside hydrolase domain | 1 | | o | | | | | | |
| novel protein containing immunoglobulin domains | 1 | o | | | | | | | |
| novel protein containing multiple sushi domains (scr repeat) | 1 | | | | | | | o | |
| novel protein containing six wd40 domains at c-terminus | 1 | | | | | | | o | |
| novel protein containing trypsin domains | 1 | o | | | | | | | |
| novel protein denn madd domain containing 2d ( zgc:153628) | 1 | | o | | | | | | |
| novel protein gamma family | 1 | | | | | | | o | |
| novel protein gpi-anchored membrane protein 1 ( zgc:55902) | 1 | | | | | o | | | |
| novel protein hairy-related ( ) | 1 | | | | | o | | | |
| novel protein hira interacting protein 5 ( zgc:110319) | 1 | | | | | | | o | |
| novel protein human and mouse btb domain containing 3 | 1 | | | | | | o | | |
| novel protein human and mouse caseinolytic atp- proteolytic subunit homolog ( coli) | 1 | | | | | | o | | |
| novel protein human and mouse par-3 partitioning defective 3 homolog b ( elegans) | 1 | o | | | | | | | |
| novel protein human and mouse sidekick homolog 1 | 1 | | | | | o | | | |
| novel protein human and mouse type domain containing 7b | 1 | o | | | | | | | |

| | | | | | | | | | |
|---|---|---|---|---|---|---|---|---|---|
| novel protein human ca2+ dependent activator protein for secretion | 1 | | | | | | | o | |
| novel protein human heterogeneous nuclear ribonucleoprotein c (c1 c2) | 1 | | | | o | | | | |
| novel protein human matrin 3 | 1 | | o | | | | | | |
| novel protein human solute carrier family sodium borate member 11 | 1 | o | | | | | | | |
| novel protein immunoglobulin member 10 | 1 | o | | | | | | | |
| novel protein lim domain 7 ( zgc:152922) | 1 | | | | | | o | | |
| novel protein opsin 5 | 1 | o | | | | | | | |
| novel protein patatin-like phospholipase domain containing 6 | 1 | | | | | o | | | |
| novel protein phosphatase and actin regulator 3 ( zgc:109967) | 1 | | | | | | | o | |
| novel protein solute carrier family member 5 | 1 | o | | | | | | | |
| novel protein tripartite motif-containing | 1 | o | | | | | | | |
| novel protein type i enveloping layer | 1 | o | | | | | | | |
| novel protein upstream binding transcription rna polymerase i ( zgc:63557) | 1 | o | | | | | | | |
| novel protein vertebrate abc1 activity of bc1 complex like ( pombe) | 1 | | o | | | | | | |
| novel protein vertebrate adenylate cyclase family | 1 | | | | | | | | o |
| novel protein vertebrate amyloid beta precursor-like protein 2 | 1 | | | | | | | o | |
| novel protein vertebrate ankyrin repeat domain 12 | 1 | o | | | | | | | |
| novel protein vertebrate ataxin 1 | 1 | | | | | | | | o |
| novel protein vertebrate atp-binding sub-family f member 1 | 1 | | o | | | | | | |
| novel protein vertebrate beta a1 | 1 | | | | | | | o | |
| novel protein vertebrate breast cancer anti-estrogen resistance 3 | 1 | o | | | | | | | |
| novel protein vertebrate cadherin h-cadherin | 1 | | | | | | | o | |
| novel protein vertebrate calcium voltage- beta 4 subunit ( zgc:136550) | 1 | | | | | | o | | |
| novel protein vertebrate capicua homolog | 1 | | | | | | o | | |
| novel protein vertebrate chondroitin sulfate proteoglycan family | 1 | | | | | | o | | |
| novel protein vertebrate cytoplasmic linker associated protein 1 | 1 | | | | | | o | | |
| novel protein vertebrate deltex 3 homolog | 1 | | o | | | | | | |
| novel protein vertebrate desmuslin | 1 | o | | | | | | | |
| novel protein vertebrate dipeptidyl-peptidase 6 | 1 | | | | | | o | | |
| novel protein vertebrate doublecortin and kinase-like 2 | 1 | | | | | | | o | |
| novel protein vertebrate egf-like repeats and discoidin i-like domains 3 | 1 | | | | | | o | | |
| novel protein vertebrate erythrocyte membrane protein band (elliptocytosis rh-linked) | 1 | o | | | | | | | |
| novel protein vertebrate fraser syndrome 1 homolog | 1 | o | | | | | | | |
| novel protein vertebrate fuse-binding protein-interacting repressor | 1 | | | | | | o | | |
| novel protein vertebrate gliacolin | 1 | | o | | | | | | |
| novel protein vertebrate imp (inosine monophosphate) dehydrogenase 1 | 1 | o | | | | | | | |

| | | | | | | | | | |
|---|---|---|---|---|---|---|---|---|---|
| novel protein vertebrate iq motif containing gtpase activating protein 2 | 1 | | o | | | | | | |
| novel protein vertebrate kinase | 1 | | | | | | | | o |
| novel protein vertebrate kinesin family member 1 family | 1 | | | | | | | o | |
| novel protein vertebrate kinesin family member 2c | 1 | o | | | | | | | |
| novel protein vertebrate myelin transcription factor 1 | 1 | | | | o | | | | |
| novel protein vertebrate myeloid lymphoid or mixed-lineage leukemia (trithorax drosophila) | 1 | o | | | | | | | |
| novel protein vertebrate myosin va (heavy polypeptide myoxin) | 1 | | | | | | o | | |
| novel protein vertebrate nephronectin | 1 | o | | | | | | | |
| novel protein vertebrate neuroepithelial cell transforming gene 1 | 1 | o | | | | | | | |
| novel protein vertebrate nuclear factor i c (ccaat-binding transcription factor) | 1 | o | | | | | | | |
| novel protein vertebrate odd oz ten-m homolog 2 | 1 | | | | | | o | | |
| novel protein vertebrate p66 alpha protein | 1 | | | | | o | | | |
| novel protein vertebrate pctaire protein kinase 2 | 1 | o | | | | | | | |
| novel protein vertebrate peroxisomal membrane protein 22kda | 1 | | o | | | | | | |
| novel protein vertebrate pest proteolytic signal containing nuclear protein ( zgc:103440) | 1 | | o | | | | | | |
| novel protein vertebrate phermone receptor protein | 1 | o | | | | | | | |
| novel protein vertebrate phosphatidylinositol binding clathrin assembly protein | 1 | o | | | | | | | |
| novel protein vertebrate phosphoinositide-3-class alpha polypeptide | 1 | | | | | | o | | |
| novel protein vertebrate pleckstrin homology domain family a (phosphoinositide binding specific) member 4 | 1 | | | | | | | | o |
| novel protein vertebrate poly binding protein 3 ( zgc:109966) | 1 | | | | | | | o | |
| novel protein vertebrate polymerase (dna directed) kappa | 1 | | o | | | | | | |
| novel protein vertebrate potassium voltage-gated kqt-like member 2 | 1 | | | | | | | o | |
| novel protein vertebrate protein tyrosine receptor d ( zgc:165626) | 1 | | | | | | o | | |
| novel protein vertebrate protein tyrosine receptor f | 1 | o | | | | | | | |
| novel protein vertebrate ptprf interacting binding protein 1 (liprin beta 1) | 1 | o | | | | | | | |
| novel protein vertebrate r3h domain containing 1 | 1 | o | | | | | | | |
| novel protein vertebrate rab6-interacting protein 2 | 1 | | | | | | o | | |
| novel protein vertebrate sh3 domain and tetratricopeptide repeats 1 | 1 | o | | | | | | | |
| novel protein vertebrate sialidase 1 (lysosomal sialidase) | 1 | o | | | | | | | |
| novel protein vertebrate solute (neurotransmitter betaine gaba) family 6 | 1 | o | | | | | | | |

| | | | | | | | | | |
|---|---|---|---|---|---|---|---|---|---|
| novel protein vertebrate solute carrier family 6 (neurotransmitter transporter) member 15 | 1 | o | | | | | | | |
| novel protein vertebrate solute carrier organic anion transporter family protein | 1 | | o | | | | | | |
| novel protein vertebrate ste20-like kinase | 1 | o | | | | | | | |
| novel protein vertebrate supervillin | 1 | o | | | | | | | |
| novel protein vertebrate titin | 1 | | | o | | | | | |
| novel protein vertebrate tumor necrosis factor receptor member 19-like | 1 | | | | | | o | | |
| novel protein vertebrate ubiquitin- homolog (xenopus laevis) | 1 | o | | | | | | | |
| novel protein vertebrate valosin-containing protein | 1 | o | | | | | | | |
| novel protein vertebrate valosin-containing protein ( zgc:136908) | 1 | o | | | | | | | |
| novel protein vertebrate wd repeat domain 44 | 1 | | | | | | o | | |
| novel protein vertebrate zinc finger protein 644 | 1 | | | | | | o | | |
| novel protein with immunoglobulin v-set andcd80-like c2-set immunoglobulin domains (zgc:172122) | 1 | | | | | | o | | |
| novel protein with lectin c-type domains | 1 | | | | | o | | | |
| novel sulfotransferase family protein | 1 | | o | | | | | | |
| novel transposon | 1 | o | | | | | | | |
| nsfl1 cofactor | 1 | | | | | | | | o |
| nsfl1 cofactor p47-like isoform 2 | 1 | | o | | | | | | |
| nsfl1c protein | 1 | o | | | | | | | |
| nt-3 growth factor receptor- partial | 1 | | | | | | o | | |
| n-terminal ef-hand calcium binding protein 1 | 1 | | | | | o | | | |
| n-terminal ef-hand calcium-binding protein 2 | 1 | | | | | | o | | |
| n-terminal ef-hand calcium-binding protein 2-like | 1 | | | | | | o | | |
| nuclear body protein sp140-like | 1 | | | | | | | | o |
| nuclear cap binding protein subunit 20kda | 1 | | | o | | | | | |
| nuclear cap-binding protein subunit 1 | 1 | | o | | | | | | |
| nuclear cap-binding protein subunit 2 | 1 | | | | o | | | | |
| nuclear distribution protein nude homolog 1 | 1 | o | | | | | | | |
| nuclear distribution protein nude-like 1 | 1 | o | | | | | | | |
| nuclear distribution protein nude-like 1-b-like | 1 | | | | o | | | | |
| nuclear export mediator factor nemf-like | 1 | | | | o | | | | |
| nuclear factor 1 b-type-like | 1 | | | | | | o | | |
| nuclear factor i a | 1 | | | | | | | o | |
| nuclear factor i b | 1 | o | | | | | | | |
| nuclear factor i c | 1 | o | | | | | | | |
| nuclear factor i x (ccaat-binding transcription factor) | 1 | | | | | o | | | |
| nuclear factor nf-kappa-b p100 subunit | 1 | o | | | | | | | |
| nuclear factor of activated t- calcineurin-dependent 1 | 1 | o | | | | | | | |
| nuclear factor of activated t- calcineurin-dependent 2 interacting protein | 1 | o | | | | | | | |
| nuclear factor of activated t-cells 5-like | 1 | | | | | | o | | |
| nuclear factor of activated t-cells tonicity-responsive | 1 | o | | | | | | | |
| nuclear factor of kappa light polypeptide gene enhancer in b-cells inhibitor-like 1 | 1 | o | | | | | | | |
| nuclear factor of kappa light polypeptide gene enhancer in b-cells inhibitor-like 2 | 1 | o | | | | | | | |
| nuclear factor ovary-like | 1 | | | | o | | | | |

| | | | | | | | | | |
|---|---|---|---|---|---|---|---|---|---|
| nuclear factor related to kappa b binding protein | 1 | | o | | | | | | |
| nuclear factor related to kappab binding protein | 1 | | o | | | | | | |
| nuclear fragile x mental retardation protein interacting protein 2 | 1 | o | | | | | | | |
| nuclear interleukin 3 regulated | 1 | o | | | | | | | |
| nuclear migration protein nudc-like | 1 | | | | | | | | o |
| nuclear mitotic apparatus protein | 1 | o | | | | | | | |
| nuclear oncoprotein skia | 1 | o | | | | | | | |
| nuclear pore complex protein nup155 | 1 | | o | | | | | | |
| nuclear pore complex protein nup85 | 1 | o | | | | | | | |
| nuclear pore complex protein nup88 | 1 | | | | | | | | o |
| nuclear protein localization 4 homolog ( cerevisiae) | 1 | o | | | | | | | |
| nuclear protein localization protein 4 homolog | 1 | | | | | | | | o |
| nuclear receptor 2c2-associated protein | 1 | | | | o | | | | |
| nuclear receptor binding protein 1 | 1 | o | | | | | | | |
| nuclear receptor binding set domain protein 1a | 1 | o | | | | | | | |
| nuclear receptor coactivator 2 | 1 | o | | | | | | | |
| nuclear receptor coactivator 6-like | 1 | | | | | | | | o |
| nuclear receptor coactivator 7 | 1 | | | | | o | | | |
| nuclear receptor co-repressor 1 isoform 1 | 1 | o | | | | | | | |
| nuclear receptor co-repressor 2 | 1 | o | | | | | | | |
| nuclear receptor co-repressor isoform cra_b | 1 | o | | | | | | | |
| nuclear receptor interacting protein 1 | 1 | o | | | | | | | |
| nuclear receptor subfamily 1 group d member 1 | 1 | | o | | | | | | |
| nuclear receptor subfamily 1 group d member 2 | 1 | | | | | | o | | |
| nuclear receptor subfamily 2 group c member 1 | 1 | o | | | | | | | |
| nuclear receptor subfamily group member 1b | 1 | | o | | | | | | |
| nuclear receptor subfamily group member 3 | 1 | | o | | | | | | |
| nuclear receptor subfamily group member 4 | 1 | | o | | | | | | |
| nuclear receptor subfamily group member 5 | 1 | | o | | | | | | |
| nuclear transcription factor gamma | 1 | o | | | | | | | |
| nuclear transcription x-box binding-like 1 | 1 | | | | | | o | | |
| nuclear transport factor 2-like export factor 2 | 1 | o | | | | | | | |
| nuclear undecaprenyl pyrophosphate synthase 1 homolog ( cerevisiae) | 1 | | o | | | | | | |
| nuclear-interacting partner of alk | 1 | o | | | | | | | |
| nucleobindin 1 | 1 | | o | | | | | | |
| nucleolar and spindle-associated protein 1 | 1 | | | | o | | | | |
| nucleolar complex associated 3 homolog | 1 | | | | | o | | | |
| nucleolar complex protein 3 homolog | 1 | | | | | | | o | |
| nucleolar protein 27kda | 1 | | | o | | | | | |
| nucleolar protein 5 | 1 | | | | | | o | | |
| nucleoporin 107kda | 1 | | o | | | | | | |
| nucleoporin 133kda | 1 | | | | | | | | o |
| nucleoporin 155 | 1 | o | | | | | | | |
| nucleoporin 160kda | 1 | | o | | | | | | |
| nucleoporin 214kda | 1 | | o | | | | | | |
| nucleoporin 88kda | 1 | o | | | | | | | |
| nucleoporin gle1 | 1 | o | | | | | | | |
| nucleoporin nup188 homolog | 1 | | | | | | | | o |
| nucleoporin p58 p45 | 1 | | | | | | o | | |
| nucleoside-triphosphatase c1orf57 | 1 | | | | | o | | | |
| nucleosomal binding protein 1 | 1 | o | | | | | | | |

| | | | | | | | | | |
|---|---|---|---|---|---|---|---|---|---|
| nucleosome-remodeling factor subunit bptf-like | 1 | o | | | | | | | |
| nucleotide binding | 1 | o | | | | | | | |
| nucleotide exchange factor sil1 precursor | 1 | | | o | | | | | |
| nucleotidyl transferase | 1 | | o | | | | | | |
| nudix (nucleoside diphosphate linked moiety x)-type motif 12 | 1 | | o | | | | | | |
| nudix (nucleoside diphosphate linked moiety x)-type motif 19 | 1 | o | | | | | | | |
| nudix-type motif 13 | 1 | | o | | | | | | |
| nur77 downstream protein 2 variant 1 | 1 | | o | | | | | | |
| nusap1 protein | 1 | o | | | | | | | |
| o-acyltransferase (membrane bound) domain containing 2 | 1 | o | | | | | | | |
| occludin | 1 | | o | | | | | | |
| octopamine receptor oamb | 1 | | o | | | | | | |
| odd oz ten-m homolog 4 | 1 | | | | | | o | | |
| odd-skipped related 1 | 1 | | | | | o | | | |
| odorant receptor | 1 | | | | o | | | | |
| ogdh protein | 1 | o | | | | | | | |
| olf-1 ebf-like-2 transcription factor | 1 | o | | | | | | | |
| olfactomedin 1 | 1 | o | | | | | | | |
| olfactomedin related er localized | 1 | o | | | | | | | |
| olfactomedin-like 1 | 1 | o | | | | | | | |
| olfactomedin-like 2a | 1 | o | | | | | | | |
| olfactomedin-like 3 | 1 | o | | | | | | | |
| oligonucleotide oligosaccharide-binding fold containing 1 | 1 | o | | | | | | | |
| oligophrenin 1 | 1 | | | | | | | o | |
| o-linked n-acetyl glucosamine transferase | 1 | o | | | | | | | |
| oncoprotein-induced transcript 3 | 1 | | o | | | | | | |
| oogenesis-related protein | 1 | | | | o | | | | |
| o-phosphoseryl-trna selenium transferase | 1 | | | o | | | | | |
| orf msv234 hypthetical protein | 1 | | | | | | o | | |
| orf61 protein | 1 | | o | | | | | | |
| organic anion transporting polypeptide 1c1 | 1 | | o | | | | | | |
| organic solute transporter beta isoform 2 | 1 | | o | | | | | | |
| origin recognition complex subunit 3 | 1 | | | | | | | | o |
| origin recognition complex subunit 3 protein | 1 | | | | | | o | | |
| origin recognition complex subunit 3-like ( cerevisiae) | 1 | | | | | | o | | |
| origin recognition complex subunit 3-like isoform 1 | 1 | | | | | | o | | |
| origin recognition complex subunit 4 | 1 | | | | o | | | | |
| origin recognition complex subunit 5 | 1 | | o | | | | | | |
| origin recognition subunit 1-like | 1 | | o | | | | | | |
| origin recognition subunit 3-like | 1 | o | | | | | | | |
| orm1-like protein 3 | 1 | o | | | | | | | |
| ornithine decarboxylase 1 | 1 | | | | | | o | | |
| ornithine mitochondrial | 1 | | | | | | | | o |
| orphan sodium- and chloride-dependent neurotransmitter transporter ntt4 | 1 | | | | | | o | | |
| orphan transporter short splicing variant | 1 | | | o | | | | | |
| o-sialoglycoprotein endopeptidase | 1 | o | | | | | | | |
| otogelin-like | 1 | o | | | | | | | |
| otopetrin 2 | 1 | | | | | | | | o |
| otospiralin | 1 | o | | | | | | | |
| otu domain containing 1 | 1 | o | | | | | | | |
| otu domain containing 4 | 1 | o | | | | | | | |
| otu domain containing 7a | 1 | | | o | | | | | |

| | | | | | | | | | |
|---|---|---|---|---|---|---|---|---|---|
| otu domain-containing protein 3 | 1 | | | | | | | | o |
| otu domain-containing protein 7a-like | 1 | | | | | | o | | |
| otu ubiquitin aldehyde binding 2 | 1 | | | o | | | | | |
| otud7b protein | 1 | o | | | | | | | |
| outer membrane efflux protein | 1 | | | | | | | o | |
| ovary-specific c1q-like factor | 1 | | o | | | | | | |
| ovary-specific mob-like protein | 1 | o | | | | | | | |
| oxa1l protein | 1 | | | | | o | | | |
| oxidation resistance 1-like | 1 | | | | | | | o | |
| oxidation resistance protein 1-like | 1 | | | | | | o | | |
| oxidoreductase nad-binding domain-containing protein 1 | 1 | | | | | | o | | |
| oxr1 protein | 1 | | | | | | o | | |
| oxysterol binding 11 | 1 | o | | | | | | | |
| oxysterol binding protein 2 | 1 | | o | | | | | | |
| oxysterol-binding protein 3 isoform 4 | 1 | | | | | | | o | |
| p21 -activated kinase 7 | 1 | o | | | | | | | |
| p2x purinoceptor 3 | 1 | | | | | | | | o |
| p2y purinoceptor 14-like | 1 | | | | o | | | | |
| p65 transcription factor | 1 | | | o | | | | | |
| p67phox [Siniperca chuatsi] | 1 | | | | | | | o | |
| p76 protein | 1 | o | | | | | | | |
| paf acetylhydrolase 29 kda subunit | 1 | o | | | | | | | |
| paired amphipathic helix protein sin3b | 1 | | | | | | | | o |
| paired-like homeodomain transcription factor 2beta | 1 | o | | | | | | | |
| pak1 interacting protein 1 | 1 | o | | | | | | | |
| pak-interacting exchange factor beta-like | 1 | | o | | | | | | |
| palmitoyl-protein thioesterase 1 | 1 | | o | | | | | | |
| palmitoyltransferase zdhhc17 | 1 | | | | | | o | | |
| palmitoyltransferase zdhhc9 | 1 | | | | | | o | | |
| pancreatic lipase-related protein 1 | 1 | o | | | | | | | |
| pancreatic progenitor cell differentiation and proliferation factor-like | 1 | | | | o | | | | |
| pancreatic secretory granule membrane major glycoprotein gp2-like | 1 | o | | | | | | | |
| pap associated domain containing 1-like | 1 | | | | | | | o | |
| papain-like cysteine prorease | 1 | | o | | | | | | |
| papillary renal cell carcinoma (translocation-associated) | 1 | o | | | | | | | |
| par-3 partitioning defective 3 homolog b ( elegans) | 1 | o | | | | | | | |
| parafibromin | 1 | | | | o | | | | |
| para-hydroxybenzoate-- mitochondrial precursor | 1 | o | | | | | | | |
| parapinopsin | 1 | | o | | | | | | |
| paraplegin (spastic paraplegia protein 7) | 1 | | o | | | | | | |
| parkinson disease 7 domain-containing protein 1-like | 1 | o | | | | | | | |
| paroxysmal nonkinesiogenic dyskinesia | 1 | | | | | | | o | |
| partner of y14 and mago | 1 | | | | | | | o | |
| parva protein | 1 | | | o | | | | | |
| pas domain-containing serine threonine-protein kinase | 1 | | | | o | | | | |
| patatin-like phospholipase domain containing 7 | 1 | | o | | | | | | |
| paxillin | 1 | o | | | | | | | |
| paxip1-associated protein 1 | 1 | o | | | | | | | |
| pb1 protein | 1 | o | | | | | | | |
| pbx knotted 1 homeobox 2 | 1 | o | | | | | | | |

| | | | | | | | | | |
|---|---|---|---|---|---|---|---|---|---|
| pcdh12 protein | 1 | o | | | | | | | |
| pcdh1a3 protein | 1 | | | | | | o | | |
| pcdh2g16 protein | 1 | | | | | | o | | |
| pclo protein | 1 | o | | | | | | | |
| pcna-associated factor | 1 | | o | | | | | | |
| pctp-like protein | 1 | | o | | | | | | |
| pdgf associated protein | 1 | | | o | | | | | |
| pdz and lim domain 5 isoform 2 | 1 | o | | | | | | | |
| pdz and lim domain protein 1-like | 1 | | | | | | | | o |
| pdz and lim domain protein 2 | 1 | o | | | | | | | |
| pdz domain containing 11 | 1 | o | | | | | | | |
| pdz domain-containing protein 11 | 1 | | | | | | | | o |
| pdz domain-containing protein gipc1 | 1 | | | | o | | | | |
| pecanex-like 3 | 1 | | | | | | o | | |
| pecanex-like protein 1 | 1 | | | | | | o | | |
| pelota homolog | 1 | o | | | | | | | |
| pentatricopeptide repeat domain 1 | 1 | | | | | | o | | |
| pentraxin-related rapidly induced by il-1 beta | 1 | o | | | | | | | |
| pepsinogen a2 precursor | 1 | | o | | | | | | |
| peptidase m20 domain containing 1 | 1 | | | | | o | | | |
| peptidase s1 family protein | 1 | | | | o | | | | |
| peptide deformylase-like | 1 | o | | | | | | | |
| peptide-n4-n-acetyl-beta-glucosaminylasparagine amidase | 1 | o | | | | | | | |
| peptidoglycan- domain containing 3 | 1 | | | o | | | | | |
| peptidyl arginine type ii | 1 | o | | | | | | | |
| peptidyl prolyl isomerase h | 1 | | | | | o | | | |
| peptidylarginine deiminase type iv | 1 | o | | | | | | | |
| peptidylglycine alpha-amidating monooxygenase | 1 | | | o | | | | | |
| peptidyl-glycine alpha-amidating monooxygenase a precursor | 1 | o | | | | | | | |
| peptidyl-prolyl cis-trans isomerase c-like | 1 | | | | | | | | o |
| peptidyl-prolyl cis-trans isomerase fkbp10 | 1 | | | | | | | | o |
| peptidyl-prolyl cis-trans isomerase fkbp3-like | 1 | | | | | | | | o |
| peptidyl-prolyl cis-trans isomerase fkbp6-like | 1 | | | | o | | | | |
| peptidyl-prolyl cis-trans isomerase g | 1 | | | | | | o | | |
| peptidyl-prolyl cis-trans isomerase mitochondrial-like | 1 | | | | | | | | o |
| peptidyl-prolyl cis-trans isomerase-like 1 | 1 | | | | o | | | | |
| peptidylprolyl isomerase | 1 | | o | | | | | | |
| peptidylprolyl isomerase c (cyclophilin c) | 1 | o | | | | | | | |
| peptidylprolyl isomerase d | 1 | o | | | | | | | |
| peptidylprolyl isomerase h (cyclophilin h) | 1 | | | | | | | o | |
| peptidylprolyl isomerase -like 1 | 1 | o | | | | | | | |
| peptidylprolyl isomerase-like 2 | 1 | | o | | | | | | |
| peptidyl-trna hydrolase | 1 | o | | | | | | | |
| peptidyl-trna hydrolase mitochondrial | 1 | | o | | | | | | |
| perforin 1 | 1 | | | | | | | o | |
| perforin-1-like | 1 | o | | | | | | | |
| pericentrin 2 | 1 | | o | | | | | | |
| pericentriolar material 1 | 1 | | | | | | o | | |
| pericentriolar material 1 protein | 1 | | | | | | | | o |
| period 1 | 1 | o | | | | | | | |
| peroxidasin homolog | 1 | o | | | | | | | |
| peroxiredoxin 2 | 1 | | | | o | | | | |
| peroxiredoxin 3 | 1 | o | | | | | | | |
| peroxisomal 3-ketoacyl- thiolase a | 1 | | o | | | | | | |

| | | | | | | | | | |
|---|---|---|---|---|---|---|---|---|---|
| peroxisomal bifunctional enzyme | 1 | | o | | | | | | |
| peroxisomal biogenesis factor 11a | 1 | | o | | | | | | |
| peroxisomal biogenesis factor 16 | 1 | o | | | | | | | |
| peroxisomal biogenesis factor 3 | 1 | | | | | | | | o |
| peroxisomal biogenesis factor 6 | 1 | o | | | | | | | |
| peroxisomal carnitine o-octanoyltransferase | 1 | | o | | | | | | |
| peroxisomal coenzyme a diphosphatase nudt7 | 1 | | o | | | | | | |
| peroxisomal membrane protein 11c-like | 1 | | | | o | | | | |
| peroxisomal membrane protein 4-like | 1 | | | | | | | | o |
| peroxisomal -trans-enoyl- isomerase-like | 1 | | o | | | | | | |
| peroxisome assembly protein 12-like | 1 | | | | o | | | | |
| peroxisome proliferator-activated receptor alpha | 1 | o | | | | | | | |
| peroxisome proliferator-activated receptor coactivator-related 1 | 1 | | | o | | | | | |
| peroxisome proliferator-activated receptor gamma coactivator-related protein 1-like | 1 | | | | o | | | | |
| pest proteolytic signal-containing nuclear | 1 | | | | | | | | o |
| ph domain containing protein | 1 | o | | | | | | | |
| phage protein | 1 | | | | o | | | | |
| phd finger protein 10 | 1 | o | | | | | | | |
| phd finger protein 13 | 1 | | | | | | o | | |
| phd finger protein 2 | 1 | o | | | | | | | |
| phd finger protein 8 | 1 | o | | | | | | | |
| phd finger-like domain-containing protein 5a | 1 | | o | | | | | | |
| phenylalanyl-trna beta subunit | 1 | | | o | | | | | |
| phf3 protein | 1 | o | | | | | | | |
| phosducin-like | 1 | | | | | | | o | |
| phosphatase 1 regulatory subunit 14b | 1 | | | | | | | o | |
| phosphatase and actin regulator 1-like | 1 | | | | | | | | o |
| phosphatase and tensin homolog | 1 | o | | | | | | | |
| phosphatidate phosphatase lpin2 | 1 | | | | | | o | | |
| phosphatidate phosphatase ppapdc1b | 1 | | | o | | | | | |
| phosphatidic acid phosphatase type 2 domain containing 3 | 1 | o | | | | | | | |
| phosphatidic acid phosphatase type 2a | 1 | o | | | | | | | |
| phosphatidylcholine:ceramide cholinephosphotransferase 1 | 1 | | o | | | | | | |
| phosphatidylethanolamine-binding protein 1 | 1 | | | | | o | | | |
| phosphatidylinositide phosphatase sac1 | 1 | o | | | | | | | |
| phosphatidylinositol 3 regulatory polypeptide 3 | 1 | | | | | | o | | |
| phosphatidylinositol 3-kinase regulatory subunit beta | 1 | o | | | | | | | |
| phosphatidylinositol 4- alpha polypeptide | 1 | | o | | | | | | |
| phosphatidylinositol 4- beta | 1 | o | | | | | | | |
| phosphatidylinositol- -bisphosphate 3-kinase catalytic subunit beta isoform | 1 | | o | | | | | | |
| phosphatidylinositol glycan anchor class s | 1 | o | | | | | | | |
| phosphatidylinositol transfer alpha | 1 | | | | | | o | | |
| phosphatidylinositol transfer beta | 1 | | | | | | o | | |
| phosphatidylinositol transfer protein beta isoform-like | 1 | | | | o | | | | |
| phosphatidylinositol- -trisphosphate-dependent rac exchange factor 1 | 1 | | | | | | o | | |
| phosphatidylinositol -trisphosphate-dependent rac exchanger 1 | 1 | | | | | | o | | |
| phosphatidylinositol-3-phosphate phosphatidylinositol 5- type iii | 1 | | | | | | o | | |

| | | | | | | | | | |
|---|---|---|---|---|---|---|---|---|---|
| phosphatidylinositol-4-phosphate 3-kinase c2 domain-containing subunit alpha | 1 | | o | | | | | | |
| phosphatidylinositol-4-phosphate 5- type gamma | 1 | | | | | | o | | |
| phosphatidylinositol-5-phosphate 4-kinase type-2 beta | 1 | | | | | | | | o |
| phosphatidylinositol-5-phosphate 4-kinase type-2 gamma | 1 | | o | | | | | | |
| phosphatidylinositol-glycan biosynthesis class w protein | 1 | o | | | | | | | |
| phosphatidylserine synthase 1 | 1 | | | | | | | | o |
| phosphodiesterase camp-specific (phosphodiesterase e4 dunce drosophila) | 1 | | | | | | o | | |
| phosphoenolpyruvate cytosolic | 1 | | o | | | | | | |
| phosphofurin acidic cluster sorting protein 1 | 1 | | | | | o | | | |
| phosphoglucomutase-1-like isoform 2 | 1 | o | | | | | | | |
| phosphoinositide-3- regulatory polypeptide 3 ( gamma) | 1 | | o | | | | | | |
| phosphoinositide-3- regulatory subunit 1 (p85 alpha) | 1 | | | o | | | | | |
| phosphoinositide-3-kinase-interacting protein 1 precursor | 1 | o | | | | | | | |
| phospholamban | 1 | o | | | | | | | |
| phospholipase a2 precursor | 1 | | | | | | o | | |
| phospholipase beta 4 | 1 | | | | | | o | | |
| phospholipase d member 4 | 1 | o | | | | | | | |
| phospholipase d member 5 | 1 | | | | | | | o | |
| phospholipase d1b | 1 | o | | | | | | | |
| phospholipase delta 3 | 1 | o | | | | | | | |
| phospholipase group vii (platelet-activating factor plasma) | 1 | | o | | | | | | |
| phospholipid scramblase 3 | 1 | | | | | | | o | |
| phospholipid-hydroperoxide glutathione peroxidase | 1 | | | | o | | | | |
| phosphopantothenoylcysteine synthetase | 1 | | | o | | | | | |
| phosphoribosyl pyrophosphate synthetase 2 | 1 | | | o | | | | | |
| phosphoribosyl transferase domain containing 1 | 1 | | o | | | | | | |
| phosphorylase alpha 2 | 1 | | | | | | | o | |
| phosphorylase b kinase gamma catalytic testis liver isoform | 1 | | | | | | | | o |
| phosphorylase b kinase regulatory subunit beta | 1 | o | | | | | | | |
| phosphorylase gamma 1 | 1 | o | | | | | | | |
| phosphorylase gamma 2 | 1 | | | o | | | | | |
| phosphorylase kinase alpha 1 | 1 | o | | | | | | | |
| phosphoserine aminotransferase | 1 | | | | | | | | o |
| phosphoseryl-trna kinase | 1 | | | o | | | | | |
| photoreceptor cadherin | 1 | o | | | | | | | |
| phthioceranic hydroxyphthioceranic acid synthase-like | 1 | | | | | | | | o |
| phyhd1 protein | 1 | | | | | | | | o |
| phytanoyl- dioxygenase | 1 | | | | | o | | | |
| phytanoyl- dioxygenase domain containing 1 | 1 | | | | | | | | o |
| phytanoyl- dioxygenase domain-containing protein 1 | 1 | | o | | | | | | |
| phytanoyl- hydroxylase-interacting | 1 | o | | | | | | | |
| phytanoyl- peroxisomal precursor | 1 | | o | | | | | | |
| piggybac transposable element derived 1-like | 1 | | | | | | | o | |
| piggybac transposable element derived 5 | 1 | | | | | | | o | |

| | | | | | | | | | |
|---|---|---|---|---|---|---|---|---|---|
| piggybac transposable element-derived protein 2-like isoform 1 | 1 | | | | | | o | | |
| piggybac transposase | 1 | | | | | | o | | |
| piggybac transposase uribo2 | 1 | o | | | | | | | |
| pih1 domain-containing protein 1 | 1 | o | | | | | | | |
| pin2 terf1-interacting telomerase inhibitor 1-like | 1 | | | | o | | | | |
| piscidin-like peptide | 1 | | | o | | | | | |
| pith domain-containing protein 1 | 1 | | o | | | | | | |
| pith domain-containing protein 1-like | 1 | | | | o | | | | |
| pitrilysin metallopeptidase 1 | 1 | o | | | | | | | |
| piwi-like protein 2 | 1 | | | | | o | | | |
| pla2g12b protein | 1 | | o | | | | | | |
| plac8-like 1 | 1 | o | | | | | | | |
| plac8-like protein 1 | 1 | o | | | | | | | |
| placental growth factor-like | 1 | o | | | | | | | |
| placenta-specific gene 8 | 1 | | | | o | | | | |
| plakophilin 1 | 1 | o | | | | | | | |
| plakophilin 3 | 1 | | | o | | | | | |
| plakophilin 4 isoform 2 | 1 | | o | | | | | | |
| plakophilin isoform 1 | 1 | | | | | o | | | |
| plakophilin-4 isoform 1 | 1 | | | | | | | | o |
| plasma kallikrein precursor | 1 | | o | | | | | | |
| plasma membrane calcium atpase 1 | 1 | | | | | | | o | |
| plasma membrane calcium atpase 2 isoform 5 | 1 | | | | | | | o | |
| plasma membrane calcium-transporting atpase 2 isoform 2 | 1 | | | | | | | o | |
| plasminogen activator inhibitor 1 rna-binding | 1 | | | | | | | | o |
| plasminogen activator inhibitor 1 rna-binding protein | 1 | | o | | | | | | |
| plastin 1 (i isoform) | 1 | o | | | | | | | |
| plastin 3 (t-isoform) | 1 | | o | | | | | | |
| plastin-1 | 1 | o | | | | | | | |
| platelet endothelial cell adhesion molecule precursor | 1 | | | | | | | | o |
| platelet glycoprotein ib alpha polypeptide | 1 | o | | | | | | | |
| platelet glycoprotein v-like | 1 | o | | | | | | | |
| platelet receptor gi24 | 1 | o | | | | | | | |
| platelet-activating factor acetylhydrolase | 1 | o | | | | | | | |
| platelet-derived growth factor alpha polypeptide | 1 | o | | | | | | | |
| platelet-derived growth factor receptor alpha-like | 1 | o | | | | | | | |
| platelet-derived growth factor receptor-like | 1 | o | | | | | | | |
| pleckstrin and sec7 domain containing | 1 | | | | | | o | | |
| pleckstrin and sec7 domain containing 2 | 1 | | o | | | | | | |
| pleckstrin homology domain family a (phosphoinositide binding specific) member 1 | 1 | o | | | | | | | |
| pleckstrin homology domain family g (with ef domain) member 3 | 1 | | o | | | | | | |
| pleckstrin homology domain family g (with ef domain) member 5 | 1 | | o | | | | | | |
| pleckstrin homology domain family g (with ef domain) member 7 | 1 | | o | | | | | | |
| pleckstrin homology domain-containing family a member 7 | 1 | | o | | | | | | |
| pleckstrin homology domain-containing family g member 7 | 1 | | | | o | | | | |
| pleckstrin homology domain-containing family h member 2-like | 1 | | o | | | | | | |

| | | | | | | | | | |
|---|---|---|---|---|---|---|---|---|---|
| pleckstrin homology domain-containing family o member 2 | 1 | o | | | | | | | |
| pleckstrin homology sec7 and coiled-coil domains-binding protein | 1 | o | | | | | | | |
| pleckstrin homology-like domain family a member 2 | 1 | | | | | | o | | |
| pleckstrin homology-like domain family b member 1 | 1 | | | | | | o | | |
| pleckstrin homology-like domain family b member 1-like | 1 | | | | | | | | o |
| pleckstrin homology-like domain family b member 2 | 1 | | o | | | | | | |
| pleckstrin homology-like family member 1 isoform 1 | 1 | o | | | | | | | |
| plectin 1 | 1 | | | | | | | o | |
| plectin 11 isoform 3 | 1 | o | | | | | | | |
| plectin isoform 1f | 1 | o | | | | | | | |
| plenty of sh3s | 1 | | | | | o | | | |
| plexin a2 | 1 | | | | | | o | | |
| plexin domain-containing protein 1 | 1 | | | | o | | | | |
| pms1 protein homolog 1 | 1 | | | | | | | o | |
| pneumococcal surface protein a | 1 | o | | | | | | | |
| podocalyxin-like protein 1-like | 1 | o | | | | | | | |
| pol polyprotein precursor | 1 | | | o | | | | | |
| poliovirus receptor | 1 | o | | | | | | | |
| poliovirus receptor related 2 | 1 | | | o | | | | | |
| poliovirus receptor related-gene 1 | 1 | | | | | | | o | |
| poliovirus receptor-related 1 | 1 | | | o | | | | | |
| poliovirus receptor-related 1 (herpesvirus entry mediator c) | 1 | | | | | | | o | |
| poliovirus receptor-related protein 2-like | 1 | | | | | | o | | |
| poliovirus receptor-related protein 3-like | 1 | | | | o | | | | |
| poly (adp-ribose) polymerase member 11 | 1 | | | | | | | o | |
| poly (adp-ribose) polymerase member 14-like | 1 | o | | | | | | | |
| poly (adp-ribose) polymerase member 16 | 1 | | | o | | | | | |
| poly (adp-ribose) polymerase member 2 | 1 | o | | | | | | | |
| poly (adp-ribose) polymerase member 3 | 1 | o | | | | | | | |
| poly (adp-ribose) polymerase member 9 | 1 | | o | | | | | | |
| poly (adp-ribose) polymerase member isoform cra_a | 1 | | | | | | o | | |
| poly (adp-ribose) polymerase member isoform cra_b | 1 | | o | | | | | | |
| poly binding protein | 1 | | | | | | | | o |
| poly -binding protein 4 | 1 | | | | | | | | o |
| poly binding protein interacting protein 2b | 1 | | | o | | | | | |
| poly binding protein isoform cra_b | 1 | | | | | | | o | |
| poly polymerase 12 | 1 | | | o | | | | | |
| poly -specific ribonuclease parn | 1 | | | | | | | | o |
| poly(adp-ribose) glycohydrolase arh3 | 1 | | o | | | | | | |
| polyadenylate-binding protein 1-like | 1 | | | | o | | | | |
| polyadenylate-binding protein-interacting protein 1 | 1 | | | | o | | | | |
| polyadenylate-binding protein-interacting protein 1 isoform 2 | 1 | | | | o | | | | |
| polyadenylate-binding protein-interacting protein 2 | 1 | o | | | | | | | |
| polycomb group protein asxl2 | 1 | | o | | | | | | |
| polycomb group ring finger protein 2 | 1 | | | | | | | | o |
| polycystic kidney disease 1-like | 1 | | o | | | | | | |
| polycystin 1 | 1 | o | | | | | | | |

| | | | | | | | | | |
|---|---|---|---|---|---|---|---|---|---|
| polycystin-1- partial | 1 | | | | o | | | | |
| polyhomeotic 1-like isoform 1 | 1 | | o | | | | | | |
| polyhomeotic-like protein 3 | 1 | | | | | | | o | |
| polymerase (dna directed) epsilon 3 (p17 subunit) | 1 | o | | | | | | | |
| polymerase delta-interacting protein 3 | 1 | | | o | | | | | |
| polymerase delta-interacting protein 3-like | 1 | | | | | | | | o |
| polymerase ii (dna directed) polypeptide a isoform 1 | 1 | o | | | | | | | |
| polymerase ii (dna directed) polypeptide h | 1 | | | | | | | o | |
| polymerase iii (dna directed) polypeptide e | 1 | o | | | | | | | |
| polymerase polyprotein | 1 | o | | | | | | | |
| polymorphic epithelial mucin | 1 | | | | o | | | | |
| polypeptide n-acetylgalactosaminyltransferase 11 | 1 | | | | | | | | o |
| polypeptide n-acetylgalactosaminyltransferase 13 | 1 | | | | | | | o | |
| polypeptide n-acetylgalactosaminyltransferase 5 | 1 | | o | | | | | | |
| polypeptide n-acetylgalactosaminyltransferase 8-like | 1 | o | | | | | | | |
| polyphosphoinositide phosphatase | 1 | | | | | | | | o |
| polypyrimidine tract binding protein 1a | 1 | | | | | o | | | |
| polypyrimidine tract-binding protein 2 | 1 | | | | | | o | | |
| polyubiquitin precursor | 1 | | | | | | | | o |
| polyunsaturated fatty acid elongase | 1 | | o | | | | | | |
| possible sd repeat-containing cell surface protein precursor | 1 | o | | | | | | | |
| postreplication repair e3 ubiquitin-protein ligase rad18 | 1 | | o | | | | | | |
| potassium channel kiaa0027 | 1 | | | | | | | o | |
| potassium channel modulatory factor 1 | 1 | | o | | | | | | |
| potassium channel tetramerisation domain containing 10 | 1 | o | | | | | | | |
| potassium channel tetramerisation domain containing 12 | 1 | o | | | | | | | |
| potassium channel tetramerisation domain containing 4 | 1 | | | | | | o | | |
| potassium channel tetramerisation domain containing 6 | 1 | | | o | | | | | |
| potassium channel tetramerisation domain containing 7 | 1 | | o | | | | | | |
| potassium intermediate small conductance calcium-activated subfamily member 1 | 1 | | | | | | o | | |
| potassium inwardly-rectifying subfamily member 10 | 1 | | | | | | | o | |
| potassium inwardly-rectifying subfamily member 5 | 1 | | | | | | | o | |
| potassium inwardly-rectifying subfamily member 6 | 1 | | | | | | | o | |
| potassium subfamily member 5 | 1 | o | | | | | | | |
| potassium voltage-gated channel subfamily a member 1-like | 1 | | | | | | o | | |
| potassium voltage-gated channel subfamily a member 2-like | 1 | o | | | | | | | |
| potassium voltage-gated channel subfamily a member 4 | 1 | | | | | | o | | |
| potassium voltage-gated channel subfamily c member 1 | 1 | | | | | | o | | |

| | | | | | | | | | |
|---|---|---|---|---|---|---|---|---|---|
| potassium voltage-gated channel subfamily c member 1-like | 1 | | | | | | o | | |
| potassium voltage-gated channel subfamily d member 2 | 1 | | | | | | o | | |
| potassium voltage-gated channel subfamily e member 4 | 1 | o | | | | | | | |
| potassium voltage-gated channel subfamily g member 2-like | 1 | | | | | | | | o |
| potassium voltage-gated shaker-related member 7 | 1 | o | | | | | | | |
| potassium voltage-gated subfamily h (eag-related) member 4 | 1 | o | | | | | | | |
| potassium voltage-gated subfamily member 2-like | 1 | | | o | | | | | |
| potential cell surface flocculin | 1 | | | | | | o | | |
| potential nuclear localization sequence binding protein nsr1p | 1 | o | | | | | | | |
| potential trna (adenine-n -)-methyltransferase catalytic subunit trmt61b-like | 1 | | o | | | | | | |
| pou class transcription factor 2 | 1 | | | | | | o | | |
| pp2a b subunit pr74 | 1 | o | | | | | | | |
| ppar-alpha interacting complex protein 285 | 1 | o | | | | | | | |
| ppm1b protein | 1 | | | | | | | o | |
| ppp1r12a protein | 1 | | | | | | o | | |
| ppp1r13b protein | 1 | o | | | | | | | |
| ppp1r3a protein | 1 | o | | | | | | | |
| pppde peptidase domain containing 2 | 1 | o | | | | | | | |
| pq loop repeat containing 2 | 1 | | | o | | | | | |
| pq loop repeat-containing protein 3 precursor | 1 | | | | | o | | | |
| pq-loop repeat-containing protein 1-like isoform 1 | 1 | | | | | | o | | |
| pq-loop repeat-containing protein 2-like | 1 | | | | | | | | o |
| pr gag-pro-pol | 1 | | | | | | | o | |
| prdx-deacylase domain-containing protein 1 | 1 | | | o | | | | | |
| pre-b-cell leukemia homeobox 1 | 1 | | | | | o | | | |
| pre-b-cell leukemia transcription factor 1 | 1 | o | | | | | | | |
| pre-b-cell leukemia transcription factor 2 | 1 | o | | | | | | | |
| PREDICTED: im:7149048 [Danio rerio] | 1 | o | | | | | | | |
| PREDICTED: neurexin-2-alpha-like [Oreochromis niloticus] | 1 | | | | | | | | o |
| PREDICTED: nibrin-like [Oreochromis niloticus] | 1 | | | | o | | | | |
| PREDICTED: obscurin-like [Oreochromis niloticus] | 1 | | | | o | | | | |
| PREDICTED: pericentrin [Danio rerio] | 1 | | o | | | | | | |
| PREDICTED: phenylalanine-4-hydroxylase-like [Ailuropoda melanoleuca] | 1 | | o | | | | | | |
| PREDICTED: pyrin-like [Ailuropoda melanoleuca] | 1 | | | o | | | | | |
| PREDICTED: radixin-like [Danio rerio] | 1 | | | | | o | | | |
| PREDICTED: RW1 protein-like [Danio rerio] | 1 | o | | | | | | | |
| PREDICTED: sb:cb1045 [Danio rerio] | 1 | o | | | | | | | |
| PREDICTED: sc:d0383 [Danio rerio] | 1 | | | | | o | | | |
| PREDICTED: secretogranin-2-like [Oreochromis niloticus] | 1 | | | | | | | | o |
| PREDICTED: si:dkey-65j6.2 [Danio rerio] | 1 | o | | | | | | | |
| PREDICTED: si:rp71-1p14.5 [Danio rerio] | 1 | o | | | | | | | |
| PREDICTED: similar to CG2083-PA [Canis familiaris] | 1 | | | | | | | o | |

| | | | | | | | | | |
|---|---|---|---|---|---|---|---|---|---|
| PREDICTED: similar to chaperonin [Strongylocentrotus purpuratus] | 1 | o | | | | | | | |
| PREDICTED: similar to conserved hypothetical protein [Hydra magnipapillata] | 1 | o | | | | | | | |
| PREDICTED: similar to conserved hypothetical protein, partial [Nasonia vitripennis] | 1 | o | | | | | | | |
| PREDICTED: similar to ENSANGP00000024626 [Nasonia vitripennis] | 1 | | | | | | | o | |
| PREDICTED: similar to KIAA0404 [Pan troglodytes] | 1 | o | | | | | | | |
| PREDICTED: similar to paralemmin-3 [Ornithorhynchus anatinus] | 1 | | o | | | | | | |
| PREDICTED: similar to ReO_6 [Strongylocentrotus purpuratus] | 1 | | | o | | | | | |
| PREDICTED: STIM2 protein-like [Danio rerio] | 1 | o | | | | | | | |
| PREDICTED: syntaphilin-like [Danio rerio] | 1 | | | | | | | o | |
| PREDICTED: tetraspanin-5-like [Oreochromis niloticus] | 1 | | | | o | | | | |
| PREDICTED: thioredoxin-interacting protein-like [Oreochromis niloticus] | 1 | | | | o | | | | |
| PREDICTED: TTC3 protein-like [Danio rerio] | 1 | o | | | | | | | |
| PREDICTED: uncharacterized protein K02A2.6-like [Oreochromis niloticus] | 1 | | | | o | | | | |
| PREDICTED: uo:ion006 [Danio rerio] | 1 | | | | | | | o | |
| PREDICTED: wu:fc17b08 [Danio rerio] | 1 | o | | | | | | | |
| pregnancy zone | 1 | | o | | | | | | |
| pregnancy zone protein isoform 1 | 1 | | o | | | | | | |
| pregnancy-associated plasma protein a | 1 | o | | | | | | | |
| pregnancy-zone protein | 1 | o | | | | | | | |
| preli domain-containing protein mitochondrial-like | 1 | | | | o | | | | |
| pre-mrna cleavage complex 2 protein pcf11-like | 1 | o | | | | | | | |
| pre-mrna processing factor 8 | 1 | | o | | | | | | |
| pre-mrna splicing factor prp8 | 1 | o | | | | | | | |
| pre-mrna-processing factor 6-like | 1 | | | | o | | | | |
| pre-mrna-processing-splicing factor 8-like | 1 | | o | | | | | | |
| pre-mrna-splicing factor atp-dependent rna helicase dhx32 | 1 | | | | o | | | | |
| pre-mrna-splicing factor cwc25 homolog | 1 | | o | | | | | | |
| pre-mrna-splicing factor rbm22 | 1 | | | | o | | | | |
| pre-mrna-splicing regulator wtap | 1 | | | | | | o | | |
| prenylcysteine oxidase 1 | 1 | | o | | | | | | |
| prenyltransferase domain containing 1 | 1 | | | | | | | | o |
| prepromelanin concentrating hormone | 1 | | | | | | o | | |
| preprotein subunit | 1 | | | | | | o | | |
| presenilin-2 | 1 | | | | | | | | o |
| presequence mitochondrial precursor | 1 | | | | | | | | o |
| prickle homolog 1 | 1 | o | | | | | | | |
| prickle-like 2 | 1 | | | | | o | | | |
| prion protein | 1 | o | | | | | | | |
| prion-like-(q n-rich)-domain-bearing protein family member (pqn-44)-like | 1 | | | | | | | o | |
| prkc apoptosis wt1 regulator | 1 | o | | | | | | | |
| prkr interacting protein 1 (il11 inducible) | 1 | o | | | | | | | |
| prkr-interacting protein 1 homolog | 1 | | | | | | | | o |

| | | | | | | | | | |
|---|---|---|---|---|---|---|---|---|---|
| probable 2-ketogluconate reductase-like | 1 | | o | | | | | | |
| probable aminopeptidase npepl1 | 1 | | | | | | | | o |
| probable atp-dependent rna helicase ddx20-like | 1 | | | | o | | | | |
| probable atp-dependent rna helicase ddx23 | 1 | | | | o | | | | |
| probable atp-dependent rna helicase ddx41-like | 1 | | | | o | | | | |
| probable atp-dependent rna helicase ddx47-like | 1 | | | | | | | | o |
| probable atp-dependent rna helicase ddx59-like | 1 | | | | | | | | o |
| probable atp-dependent rna helicase ddx6 | 1 | | | | | | o | | |
| probable cation-transporting atpase 13a2 | 1 | | | | o | | | | |
| probable cell surface protein precursor | 1 | | | | o | | | | |
| probable cysteinyl-trna mitochondrial precursor | 1 | o | | | | | | | |
| probable cytosolic iron-sulfur protein assembly protein ciao1-like | 1 | | | | | | | | o |
| probable -dihydro-8-oxoguanine triphosphatase nudt15-like | 1 | | | | o | | | | |
| probable d-tyrosyl-trna deacylase 2 | 1 | o | | | | | | | |
| probable e3 ubiquitin-protein ligase herc4 | 1 | | | | | | o | | |
| probable e3 ubiquitin-protein ligase herc4 isoform 1 | 1 | | | o | | | | | |
| probable e3 ubiquitin-protein ligase herc4-like | 1 | | | o | | | | | |
| probable e3 ubiquitin-protein ligase herc4-like isoform 1 | 1 | o | | | | | | | |
| probable e3 ubiquitin-protein ligase herc4-like isoform 2 | 1 | | | | | o | | | |
| probable e3 ubiquitin-protein ligase mid2 isoform 1 | 1 | | | | | | o | | |
| probable e3 ubiquitin-protein ligase rnf144a-like | 1 | | | | | | | o | |
| probable e3 ubiquitin-protein ligase trip12 isoform 1 | 1 | | | | | | o | | |
| probable e3 ubiquitin-protein ligase trip12-like | 1 | | o | | | | | | |
| probable fructose- -bisphosphatase tigar-like | 1 | o | | | | | | | |
| probable glutathione peroxidase 8 | 1 | o | | | | | | | |
| probable g-protein coupled receptor 133 | 1 | | | | o | | | | |
| probable g-protein coupled receptor 144 | 1 | o | | | | | | | |
| probable g-protein coupled receptor 174 | 1 | | | o | | | | | |
| probable g-protein coupled receptor 45-like | 1 | | | | | | | | o |
| probable hydrolase pnkd isoform 1 | 1 | | o | | | | | | |
| probable imidazolonepropionase | 1 | | o | | | | | | |
| probable n-acetyltransferase camello | 1 | | | | o | | | | |
| probable palmitoyltransferase zdhhc16 | 1 | o | | | | | | | |
| probable palmitoyltransferase zdhhc4 | 1 | o | | | | | | | |
| probable peptide nitrate transporter at1g59740-like | 1 | | | | o | | | | |
| probable polypeptide n-acetylgalactosaminyltransferase 8-like | 1 | | o | | | | | | |
| probable polyprenol reductase | 1 | o | | | | | | | |
| probable prolyl-trna mitochondrial | 1 | | | | o | | | | |
| probable protein-cysteine n-palmitoyltransferase porcupine isoform 1 | 1 | | | | o | | | | |
| probable ribosome biogenesis protein rlp24-like | 1 | | | | | | | | o |
| probable rna polymerase ii nuclear localization protein slc7a6os | 1 | | | | o | | | | |

| | | | | | | | | | |
|---|---|---|---|---|---|---|---|---|---|
| probable rna-binding protein 20 | 1 | o | | | | | | | |
| probable rna-binding protein eif1ad | 1 | | | o | | | | | |
| probable serine carboxypeptidase cpvl-like | 1 | | | | o | | | | |
| probable ubiquitin carboxyl-terminal hydrolase faf-x isoform 1 | 1 | | | | o | | | | |
| probable ubiquitin carboxyl-terminal hydrolase faf-x-like | 1 | | | | | | o | | |
| probable ubiquitin carboxyl-terminal hydrolase faf-x-like isoform 2 | 1 | | | | | | o | | |
| processing of precursor ribonuclease p mrp subunit ( cerevisiae) | 1 | o | | | | | | | |
| procollagen galactosyltransferase 1-like | 1 | | | | | | | | o |
| procollagen- -oxoglutarate 5-dioxygenase 2 | 1 | | | | | | | | o |
| procollagen type xv | 1 | o | | | | | | | |
| profilin 2 | 1 | | o | | | | | | |
| progesterone receptor membrane component 1 | 1 | o | | | | | | | |
| progestin and adipoq receptor family member ix | 1 | | | | | | | o | |
| progestin and adipoq receptor family member vi | 1 | o | | | | | | | |
| progestin induced partial | 1 | o | | | | | | | |
| programmed cell death 11 | 1 | | o | | | | | | |
| programmed cell death 6 interacting protein | 1 | | | | | | o | | |
| programmed cell death 6-interacting | 1 | | | | | | o | | |
| prohormone convertase pace4 | 1 | o | | | | | | | |
| prolactin receptor | 1 | | | | | | | | o |
| proline arginine-rich end leucine-rich repeat protein | 1 | o | | | | | | | |
| proline rich 12 | 1 | | | | | | | o | |
| proline rich protein 2 | 1 | | | | | o | | | |
| proline synthase co-transcribed bacterial homolog protein | 1 | o | | | | | | | |
| proline-rich akt1 substrate 1 | 1 | o | | | | | | | |
| proline-rich coiled-coil 1 | 1 | o | | | | | | | |
| proline-rich protein bca3 | 1 | o | | | | | | | |
| proline-rich protein prcc | 1 | | | | | | | | o |
| proline-rich transmembrane protein 3 | 1 | o | | | | | | | |
| proline-serine-threonine phosphatase-interacting protein 1 | 1 | o | | | | | | | |
| prolyl 4-hydroxylase subunit alpha-1-like | 1 | | | | o | | | | |
| prolyl 4-hydroxylase subunit alpha-2 isoform 2 precursor | 1 | o | | | | | | | |
| prolyl 4-hydroxylase subunit alpha-2 precursor | 1 | o | | | | | | | |
| prolyl endopeptidase | 1 | o | | | | | | | |
| prolyl-trna synthetase | 1 | o | | | | | | | |
| pro-melanin concentrating hormone | 1 | | | | | o | | | |
| proopiomelanocortin b | 1 | o | | | | | | | |
| propionyl- carboxylase | 1 | o | | | | | | | |
| pro-pol-dutpase polyprotein r dutpase integrase protease reverse transcriptase | 1 | o | | | | | | | |
| proprotein convertase subtilisin kexin type 1 | 1 | | | | | | o | | |
| proprotein convertase subtilisin kexin type 6 | 1 | | | o | | | | | |
| proprotein convertase subtilisin kexin type 6-partial | 1 | | | | o | | | | |
| prospero-related homeobox 1 | 1 | | o | | | | | | |
| prostaglandin e synthase | 1 | o | | | | | | | |
| prostaglandin g h synthase 1 | 1 | o | | | | | | | |
| prostaglandin g h synthase 2 | 1 | o | | | | | | | |

| | | | | | | | | | |
|---|---|---|---|---|---|---|---|---|---|
| prostaglandin reductase 1 | 1 | o | | | | | | | |
| prostaglandin reductase 2 | 1 | o | | | | | | | |
| prostaglandin-endoperoxide synthase 1 | 1 | o | | | | | | | |
| prostate stem cell antigen | 1 | o | | | | | | | |
| protease-associated domain-containing protein of 21 kda-like | 1 | | | | o | | | | |
| proteasome ( macropain) 26s non- 1 | 1 | | o | | | | | | |
| proteasome ( macropain) 26s non- 4 | 1 | | | | | | | | o |
| proteasome ( macropain) 26s non- 7 (mov34 homolog) | 1 | | | | | o | | | |
| proteasome ( macropain) alpha 4 | 1 | | | | | | | o | |
| proteasome subunit alpha type 4 | 1 | | | | | | | | o |
| proteasome subunit beta type-4 | 1 | | | | | | | | o |
| proteasome subunit beta type-5-like | 1 | | | | | | | | o |
| protein acn9 mitochondrial-like | 1 | | o | | | | | | |
| protein amp- beta 2 non-catalytic subunit | 1 | o | | | | | | | |
| protein angel homolog 1-like | 1 | | o | | | | | | |
| protein arginine methyltransferase 2 | 1 | | o | | | | | | |
| protein arginine methyltransferase 3 | 1 | | | | o | | | | |
| protein arginine methyltransferase 5 | 1 | o | | | | | | | |
| protein arginine methyltransferase 7 | 1 | | | | | o | | | |
| protein arginine methyltransferase 8 | 1 | o | | | | | | | |
| protein arginine n-methyltransferase 10 | 1 | | | | o | | | | |
| protein arginine n-methyltransferase 3 | 1 | | | | o | | | | |
| protein arginine n-methyltransferase 5 | 1 | | | | | o | | | |
| protein arginine n-methyltransferase 6 | 1 | | | o | | | | | |
| protein argonaute-2-like | 1 | | | | | | o | | |
| protein associated with topoisomerase ii homolog 1 | 1 | | | | | | | | o |
| protein bat2-like 1-like | 1 | | | | | | o | | |
| protein bicaudal c homolog 1 | 1 | | | | o | | | | |
| protein bicaudal d homolog 1-like | 1 | | | | | | o | | |
| protein btg1 | 1 | | | o | | | | | |
| protein c | 1 | | o | | | | | | |
| protein c (inactivator of coagulation factors va and viiia) | 1 | | o | | | | | | |
| protein c16orf88-like | 1 | o | | | | | | | |
| protein c17orf37 homolog | 1 | | | | o | | | | |
| protein canopy 4 | 1 | | | | | o | | | |
| protein capicua homolog | 1 | | o | | | | | | |
| protein casc4-like isoform 1 | 1 | o | | | | | | | |
| protein cereblon-like | 1 | | | | | | o | | |
| protein creg2 | 1 | | o | | | | | | |
| protein dgcr14 | 1 | o | | | | | | | |
| protein disulfide isomerase family member 2 | 1 | | o | | | | | | |
| protein disulfide isomerase family member 5 | 1 | o | | | | | | | |
| protein disulfide-isomerase a3-like | 1 | | | | | | | | o |
| protein disulfide-isomerase a4 | 1 | | | | o | | | | |
| protein disulfide-isomerase a4-like | 1 | | o | | | | | | |
| protein disulfide-isomerase precursor | 1 | | o | | | | | | |
| protein disulfide-isomerase tmx3-like | 1 | | o | | | | | | |
| protein dom3z-like | 1 | | | | | | | | o |
| protein dopey-2-like | 1 | | | | | | o | | |
| protein dpy-19 homolog 1 | 1 | o | | | | | | | |
| protein dpy-19 homolog 1-like | 1 | | o | | | | | | |
| protein dpy-19 homolog 4 | 1 | | | | o | | | | |
| protein efr3 homolog a | 1 | | o | | | | | | |
| protein efr3 homolog b | 1 | | | | | | o | | |
| protein ejaculatory bulb | 1 | | | | | o | | | |
| protein ergic-53 | 1 | | | | | | o | | |

| | | | | | | | | | |
|---|---|---|---|---|---|---|---|---|---|
| protein fam105b-like | 1 | | o | | | | | | |
| protein fam107a-like | 1 | | o | | | | | | |
| protein fam113a | 1 | | | | o | | | | |
| protein fam135b-like | 1 | | o | | | | | | |
| protein fam160a1-like | 1 | | o | | | | | | |
| protein fam160b1-like | 1 | | o | | | | | | |
| protein fam171b-like | 1 | | | | | o | | | |
| protein fam173a-like | 1 | | | | o | | | | |
| protein fam177a1-like | 1 | | o | | | | | | |
| protein fam181b-like | 1 | | | | | | | | o |
| protein fam189a2-like | 1 | o | | | | | | | |
| protein fam18a-like | 1 | | | | | | o | | |
| protein fam18b1-like | 1 | | | | o | | | | |
| protein fam193b-like | 1 | | | | | | o | | |
| protein fam198b-like | 1 | o | | | | | | | |
| protein fam19a5-like | 1 | | | | | | | | o |
| protein fam200a | 1 | | | | o | | | | |
| protein fam20a-like | 1 | | | o | | | | | |
| protein fam3a-like isoform 1 | 1 | | | | | | | | o |
| protein fam46c-like | 1 | | | | | | o | | |
| protein fam50a | 1 | | | | o | | | | |
| protein fam53b | 1 | | | | | | | | o |
| protein fam53b-like | 1 | | | | o | | | | |
| protein fam54b-like | 1 | | o | | | | | | |
| protein fam57b | 1 | | | | | | o | | |
| protein fam57b-like | 1 | | | | | | o | | |
| protein fam59b-like | 1 | | | | | | o | | |
| protein fam65a-like | 1 | | | | o | | | | |
| protein fam78b-like | 1 | | | | | o | | | |
| protein fam8a1-like | 1 | | | | o | | | | |
| protein fam91a1-like | 1 | | | | | | o | | |
| protein farnesyltransferase subunit beta | 1 | | | | | | o | | |
| protein fem-1 homolog a | 1 | | | | | | | | o |
| protein gcap14 homolog isoform 2 | 1 | o | | | | | | | |
| protein geranylgeranyltransferase type beta subunit | 1 | | | | o | | | | |
| protein gpr108-like | 1 | | | | | | o | | |
| protein gtlf3b | 1 | o | | | | | | | |
| protein- interferon-inducible double stranded rna dependent repressor of (p58 repressor)-like | 1 | o | | | | | | | |
| protein iq-domain 14 | 1 | o | | | | | | | |
| protein khnyn | 1 | o | | | | | | | |
| protein kiaa1045-like | 1 | | | | | | o | | |
| protein kiaa1199-like | 1 | | | | | | o | | |
| protein kiaa1731-like | 1 | | | | o | | | | |
| protein kinase | 1 | o | | | | | | | |
| protein kinase (camp- catalytic) inhibitor alpha | 1 | o | | | | | | | |
| protein kinase c iota type | 1 | | | | | | o | | |
| protein kinase c-binding protein 1 | 1 | | | | o | | | | |
| protein kinase c-binding protein 1-like | 1 | | | | | | | | o |
| protein kinase c-binding protein nell1-like | 1 | | | | | | o | | |
| protein kinase delta | 1 | o | | | | | | | |
| protein kinase family protein with pasta domain | 1 | | | | | | | | o |
| protein kinase iota | 1 | o | | | | | | | |
| protein kinase isoform cra_a | 1 | o | | | | | | | |
| protein kinase n2 | 1 | | o | | | | | | |
| protein kinase theta | 1 | o | | | | | | | |

| | | | | | | | | | |
|---|---|---|---|---|---|---|---|---|---|
| protein kinase-like protein 196 | 1 | | | o | | | | | |
| protein kish-a-like | 1 | | | | | | | | o |
| protein kri1 homolog | 1 | o | | | | | | | |
| protein las1 homolog | 1 | | | | o | | | | |
| protein lbh | 1 | o | | | | | | | |
| protein lin-37 homolog | 1 | | | | | o | | | |
| protein lin-52 homolog | 1 | | | | | | | | o |
| protein lin-54 homolog | 1 | o | | | | | | | |
| protein lin-7 homolog c-like | 1 | | | | | | | | o |
| protein lsm14 homolog b | 1 | | | | o | | | | |
| protein max-like | 1 | | | | | | | | o |
| protein mb21d1 | 1 | | | | o | | | | |
| protein mcm10 homolog | 1 | o | | | | | | | |
| protein mef2bnb | 1 | | | | o | | | | |
| protein mical-2 isoform 2 | 1 | o | | | | | | | |
| protein mical-3 | 1 | | | | | | o | | |
| protein mis18-beta-like | 1 | | | | o | | | | |
| protein nlrc3 | 1 | | o | | | | | | |
| protein npat | 1 | | | | | | o | | |
| protein odr-4 homolog | 1 | | o | | | | | | |
| protein o-linked mannose -n-acetylglucosaminyltransferase | 1 | o | | | | | | | |
| protein o-mannosyl-transferase 2 | 1 | o | | | | | | | |
| protein osteopotentia homolog | 1 | | | | | | | | o |
| protein partial | 1 | | | | | | o | | |
| protein phosphatase 1 regulatory subunit 3c-b-like | 1 | | | | o | | | | |
| protein phosphatase 1e (pp2c domain containing) | 1 | | | | | | o | | |
| protein phosphatase 2 (formerly 2a) regulatory subunit b (pr 52) beta isoform | 1 | o | | | | | | | |
| protein phosphatase 2 (formerly 2a) regulatory subunit b (pr 52) beta isoform cra_c | 1 | | | | | | | o | |
| protein phosphatase catalytic alpha isozyme | 1 | | | | | | | o | |
| protein phosphatase catalytic beta isozyme | 1 | | | | | | o | | |
| protein phosphatase magnesium- catalytic subunit | 1 | | | | | | | o | |
| protein phosphatase regulatory subunit 1 | 1 | o | | | | | | | |
| protein phosphatase regulatory subunit 10 | 1 | o | | | | | | | |
| protein phosphatase regulatory subunit 13 like | 1 | | o | | | | | | |
| protein phosphatase regulatory subunit 14c | 1 | | | | | o | | | |
| protein phosphatase regulatory subunit 15b | 1 | | | | | | | | o |
| protein phosphatase regulatory subunit 1a | 1 | | | | | | | o | |
| protein phosphatase regulatory subunit 1b | 1 | | | o | | | | | |
| protein phosphatase regulatory subunit 8 | 1 | | | o | | | | | |
| protein phosphatase regulatory subunit b (pr 53) | 1 | o | | | | | | | |
| protein phosphatase regulatory subunit b epsilon isoform | 1 | | o | | | | | | |
| protein phosphatase regulatory subunit b gamma | 1 | | | o | | | | | |
| protein product of hmfn0672 | 1 | o | | | | | | | |
| protein prrc2b | 1 | | | | | | o | | |
| protein prrc2c | 1 | | | | | | o | | |
| protein quaking-a | 1 | | | | | | o | | |
| protein reprimo-like | 1 | | | | | | | | o |
| protein rer1-like | 1 | | | | o | | | | |
| protein saal1 | 1 | | | | o | | | | |

| | | | | | | | | | |
|---|---|---|---|---|---|---|---|---|---|
| protein scribble homolog | 1 | | | | | | o | | |
| protein scribble partial | 1 | | | | | | o | | |
| protein serine threonine kinase | 1 | o | | | | | | | |
| protein shisa-4-like | 1 | | | | | | o | | |
| protein shroom2-like | 1 | | o | | | | | | |
| protein slc7a6os | 1 | | o | | | | | | |
| protein smg5 | 1 | | o | | | | | | |
| protein spinster homolog 1-like | 1 | | o | | | | | | |
| protein strawberry notch homolog 2-like | 1 | | | | | | o | | |
| protein strawberry notch homolog partial | 1 | | | | | | o | | |
| protein tex261 | 1 | o | | | | | | | |
| protein timeless homolog | 1 | | | | | | | | o |
| protein tob1-like | 1 | | | | | | | | o |
| protein transport protein sec23b | 1 | | o | | | | | | |
| protein transport protein sec24a | 1 | o | | | | | | | |
| protein transport protein sec24b-like | 1 | | | | | | o | | |
| protein transport protein sec24d | 1 | | o | | | | | | |
| protein transport protein sec61 subunit alpha-like 1 | 1 | | o | | | | | | |
| protein tweety homolog 1 | 1 | | | | | | | o | |
| protein tweety homolog 3-like | 1 | | | | | | o | | |
| protein tyrosine non-receptor type 11 | 1 | o | | | | | | | |
| protein tyrosine non-receptor type 12 | 1 | | | | | | | o | |
| protein tyrosine non-receptor type 14 | 1 | o | | | | | | | |
| protein tyrosine non-receptor type 2 | 1 | | | | o | | | | |
| protein tyrosine non-receptor type 23 | 1 | o | | | | | | | |
| protein tyrosine non-receptor type 4 | 1 | | | | | | o | | |
| protein tyrosine non-receptor type 6 | 1 | | | | | o | | | |
| protein tyrosine phosphatase-like a domain containing 2 | 1 | o | | | | | | | |
| protein tyrosine receptor b | 1 | | | | o | | | | |
| protein tyrosine receptor g | 1 | | o | | | | | | |
| protein tyrosine receptor isoform cra_a | 1 | o | | | | | | | |
| protein tyrosine receptor k | 1 | | | | | | | o | |
| protein tyrosine receptor m | 1 | o | | | | | | | |
| protein tyrosine receptor o | 1 | | | | | o | | | |
| protein tyrosine receptor s | 1 | o | | | | | | | |
| protein tyrosine receptor t | 1 | | | | o | | | | |
| protein tyrosine receptor- z polypeptide 1 | 1 | | | | | | | o | |
| protein unc-13 homolog c-like | 1 | | | | | | | | o |
| protein unc-13 homolog d | 1 | | | | o | | | | |
| protein unc-13-like protein b | 1 | | | | | | o | | |
| protein uxt | 1 | o | | | | | | | |
| protein virilizer homolog | 1 | | | | | | o | | |
| protein with 5 -3 exonuclease domain | 1 | | | | o | | | | |
| protein x-linked | 1 | | | | | | o | | |
| protein yif1b-like | 1 | | o | | | | | | |
| protein yipf7 | 1 | o | | | | | | | |
| protein z-dependent protease inhibitor | 1 | | | | | | | | o |
| protein zer-1 homolog | 1 | | | | | | o | | |
| protein zwilch homolog | 1 | | | | o | | | | |
| protein zyg-11 homolog | 1 | o | | | | | | | |
| protein-arginine deiminase type-4-like | 1 | o | | | | | | | |
| protein-associating with the carboxyl-terminal domain of ezrin | 1 | | | | | | o | | |
| protein-glutamine gamma-glutamyltransferase 5-like | 1 | | | | | | | | o |
| protein-l-isoaspartated-aspartate o-methyltransferase | 1 | | | | | | | | o |
| protein-o-mannosyltransferase 1 | 1 | o | | | | | | | |

| | | | | | | | | | |
|---|---|---|---|---|---|---|---|---|---|
| protein-tyrosine sulfotransferase 2-like | 1 | | o | | | | | | |
| proteolipid protein 2 | 1 | o | | | | | | | |
| protocadherin 1 gamma 2 | 1 | | | | | | | o | |
| protocadherin 19 | 1 | | | | | | | o | |
| protocadherin 2 gamma 1 | 1 | o | | | | | | | |
| protocadherin 2a11 | 1 | | | | | | | | o |
| protocadherin 2a12 | 1 | | | | | | | o | |
| protocadherin 2g1 | 1 | | | | | | o | | |
| protocadherin 2g15 | 1 | | | | | | | o | |
| protocadherin 2g17 | 1 | | | | | | | o | |
| protocadherin 2g18 | 1 | | | | | | | | o |
| protocadherin 2g19 | 1 | | | | | | | o | |
| protocadherin 2g2 | 1 | | | | | | o | | |
| protocadherin 2g23 | 1 | o | | | | | | | |
| protocadherin 2g30-like | 1 | | | | | | | o | |
| protocadherin 2g32 | 1 | | | | | | | o | |
| protocadherin 2g5 | 1 | | | | | | o | | |
| protocadherin 7 | 1 | | | | | | o | | |
| protocadherin alpha-3-like | 1 | | | | | | o | | |
| protocadherin alpha-8-like | 1 | | | | | | o | | |
| protocadherin fat 1 | 1 | | | o | | | | | |
| protocadherin fat 2 | 1 | | | | | | | o | |
| protocadherin fat 3 | 1 | o | | | | | | | |
| protocadherin gamma a2 precursor (pcdh-gamma-a2) | 1 | | | | | | o | | |
| protocadherin gamma-a11-like | 1 | | | | | | o | | |
| protocadherin-11 x-linked- partial | 1 | | | | | | o | | |
| protocadherin-15 isoform 4 | 1 | | | | o | | | | |
| protocadherin-17 precursor | 1 | | | | | | | | o |
| protocadherin-9-like isoform 1 | 1 | | | | | | o | | |
| proton myo-inositol cotransporter | 1 | | | | o | | | | |
| proton-coupled amino acid transporter 1-like isoform 2 | 1 | | o | | | | | | |
| proto-oncogene c-rel-like | 1 | | | | o | | | | |
| proto-oncogene dbl-like | 1 | | | | | | o | | |
| proz protein | 1 | | | | | | | o | |
| prp31 pre-mrna processing factor 31 homolog | 1 | | | o | | | | | |
| prp40 pre-mrna processing factor 40 homolog a | 1 | | | | | o | | | |
| prp6 pre-mrna splicing factor 6 homolog | 1 | o | | | | | | | |
| prpf39 protein | 1 | o | | | | | | | |
| psap protein | 1 | | | o | | | | | |
| pseudouridine synthase 1 | 1 | | | | | | | | o |
| pseudouridine-metabolizing bifunctional | 1 | | o | | | | | | |
| pseudouridylate synthase 10 | 1 | o | | | | | | | |
| pseudouridylate synthase 7 homolog | 1 | | | | | | o | | |
| psme4 protein | 1 | | | | o | | | | |
| ptb domain-containing engulfment adapter protein 1 | 1 | | | | | | | o | |
| ptd016 protein | 1 | | o | | | | | | |
| ptgs1 protein | 1 | o | | | | | | | |
| ptk2 protein tyrosine kinase 2 | 1 | | | | | | | o | |
| ptk2 protein tyrosine kinase isoform cra_a | 1 | o | | | | | | | |
| ptk7 protein tyrosine kinase 7- partial | 1 | o | | | | | | | |
| ptprf interacting binding protein 2 (liprin beta 2) | 1 | | | | | | o | | |
| ptprf interacting protein binding protein 1 isoform 2 | 1 | | o | | | | | | |
| pumilio homolog 2 | 1 | | | | | | o | | |
| purine-rich element binding protein b | 1 | | o | | | | | | |

| | | | | | | | | | |
|---|---|---|---|---|---|---|---|---|---|
| puromycin-sensitive aminopeptidase | 1 | | o | | | | | | |
| purpurin | 1 | | | | | o | | | |
| putative utrophin [Takifugu rubripes] | 1 | o | | | | | | | |
| pvalb6 protein | 1 | | | | | | | | o |
| px domain-containing protein kinase-like protein | 1 | o | | | | | | | |
| pyocin tail formation | 1 | | | | o | | | | |
| pyridine nucleotide-disulfide oxidoreductase domain-containing protein 1 | 1 | o | | | | | | | |
| pyridine nucleotide-disulfide oxidoreductase domain-containing protein 2 | 1 | | o | | | | | | |
| pyridoxal kinase | 1 | | | | | | | | o |
| pyridoxine 5 -phosphate oxidase | 1 | o | | | | | | | |
| pyridoxine-5 -phosphate oxidase-like | 1 | | | | o | | | | |
| pyrophosphatase phosphodiesterase 4 | 1 | | | | | | o | | |
| pyrroline-5-carboxylate reductase 2 | 1 | | o | | | | | | |
| pyrroline-5-carboxylate reductase family member 2 variant 1 | 1 | | | o | | | | | |
| pyrroline-5-carboxylate reductase-like protein c14orf148-like | 1 | | | | o | | | | |
| pyruvate dehydrogenase e1 alpha 1 | 1 | | o | | | | | | |
| pyruvate dehydrogenase isoenzyme 3 | 1 | o | | | | | | | |
| pyruvate dehydrogenase isozyme 3 | 1 | | | | | | o | | |
| pyruvate dehydrogenase isozyme 4 | 1 | | o | | | | | | |
| pyruvate dehydrogenase phosphatase isoenzyme 2 | 1 | o | | | | | | | |
| pyruvate dehydrogenase phosphatase regulatory subunit | 1 | o | | | | | | | |
| pyruvate dehydrogenase protein x mitochondrial | 1 | | | | | | | | o |
| pyruvate kinase muscle isozyme | 1 | | | | | | | | o |
| pyruvate liver and rbc | 1 | | o | | | | | | |
| pyruvate muscle | 1 | | o | | | | | | |
| pzp protein | 1 | | | | | | | | o |
| questionable orf | 1 | o | | | | | | | |
| queuine trna-ribosyltransferase subunit qtrtd1 | 1 | | | o | | | | | |
| quiescin q6 sulfhydryl oxidase 1 | 1 | | o | | | | | | |
| quinone oxidoreductase | 1 | | o | | | | | | |
| r3h domain-containing protein c19orf22 homolog | 1 | o | | | | | | | |
| r3hdm2 protein | 1 | | | | | | | | o |
| rab alpha subunit | 1 | o | | | | | | | |
| rab gtpase-binding effector protein 2 | 1 | o | | | | | | | |
| rab11 family interacting protein 2 (class i) | 1 | o | | | | | | | |
| rab11 family interacting protein 4 (class ii) | 1 | | | | | o | | | |
| rab11 family-interacting protein 1 isoform 2 | 1 | | | | o | | | | |
| rab3a interacting 1 | 1 | o | | | | | | | |
| rab5 gdp gtp exchange factor | 1 | o | | | | | | | |
| rab9 effector protein with kelch motifs | 1 | o | | | | | | | |
| rab-like protein 2a-like | 1 | | | | o | | | | |
| rac cdc42 guanine nucleotide exchange factor 2 | 1 | o | | | | | | | |
| rac gtpase activating protein 1 | 1 | | | | | | o | | |
| rad23 homolog b ( cerevisiae) | 1 | o | | | | | | | |
| rad23b protein | 1 | | | o | | | | | |
| rad50 interactor 1 | 1 | | o | | | | | | |
| rad51-like 1 | 1 | o | | | | | | | |
| radixin isoform d | 1 | | | | o | | | | |
| raf proto-oncogene serine threonine-protein kinase-like | 1 | o | | | | | | | |
| ragulator complex protein lamtor1 | 1 | | | | | | o | | |

| | | | | | | | | | |
|---|---|---|---|---|---|---|---|---|---|
| ral gtpase-activating protein alpha subunit 2-like | 1 | o | | | | | | | |
| ral gtpase-activating protein subunit beta | 1 | o | | | | | | | |
| ralbp1 associated eps domain containing 1 | 1 | o | | | | | | | |
| ralgps2 protein | 1 | o | | | | | | | |
| ran binding protein 10 | 1 | o | | | | | | | |
| ran binding protein 5 | 1 | | o | | | | | | |
| ran gtpase-activating protein 1 | 1 | | o | | | | | | |
| ran guanine nucleotide release factor | 1 | o | | | | | | | |
| ranbp2-like and grip domain-containing protein 5 6 isoform 1 | 1 | | | | | | o | | |
| rangap1 protein | 1 | o | | | | | | | |
| ran-specific gtpase-activating protein | 1 | | | | | o | | | |
| rap guanine nucleotide exchange factor 1 | 1 | | o | | | | | | |
| rap guanine nucleotide exchange factor 3 | 1 | | | | | o | | | |
| rap guanine nucleotide exchange factor 5-like | 1 | | | | | | o | | |
| rap1 gtpase-gdp dissociation stimulator 1-like | 1 | | | | | o | | | |
| rapamycin-insensitive companion of mtor | 1 | | | | | | o | | |
| ras association ( af-6) and pleckstrin homology domains 1 | 1 | | | | | | o | | |
| ras association domain-containing protein 8 | 1 | | | | o | | | | |
| ras gtpase | 1 | | | | | o | | | |
| ras gtpase-activating protein ngap | 1 | o | | | | | | | |
| ras gtpase-activating protein-binding protein 2 | 1 | | o | | | | | | |
| ras homolog gene member g (rho g) | 1 | o | | | | | | | |
| ras homolog gene member q | 1 | o | | | | | | | |
| ras p21 protein activator 2 | 1 | | | | | | | o | |
| ras related protein 1b | 1 | o | | | | | | | |
| ras-association domain family 7 | 1 | | | | | | | o | |
| ras-like gtp-binding protein rho1 | 1 | | | | | | o | | |
| ras-like protein family member 11a-like | 1 | o | | | | | | | |
| ras-related and estrogen-regulated growth inhibitor-like | 1 | | | | o | | | | |
| ras-related associated with diabetes | 1 | o | | | | | | | |
| ras-related c3 botulinum toxin substrate 1 precursor | 1 | | | | | | o | | |
| ras-related gtp binding c | 1 | | o | | | | | | |
| ras-related gtp-binding protein c | 1 | | | | o | | | | |
| ras-related protein orab-1 | 1 | | | | | o | | | |
| ras-related protein rab-11a | 1 | o | | | | | | | |
| ras-related protein rab-1a | 1 | | | o | | | | | |
| ras-related protein rab-26-like | 1 | | | | o | | | | |
| ras-related protein rab-34 | 1 | o | | | | | | | |
| ras-related protein rab-35 | 1 | | | | o | | | | |
| ras-related protein rab-6a-like | 1 | | | | | | o | | |
| ras-related protein ral-b | 1 | | | o | | | | | |
| rb1-inducible coiled coil protein 1 | 1 | | | o | | | | | |
| rb1-inducible coiled-coil protein 1 | 1 | | | | | o | | | |
| rcc1 and btb domain-containing protein 2 | 1 | | o | | | | | | |
| rcc1 domain-containing protein 1 | 1 | | | | | | | | o |
| rcc2 homolog | 1 | o | | | | | | | |
| receptor activity modifying protein 2 | 1 | o | | | | | | | |
| receptor activity modifying protein 3 | 1 | o | | | | | | | |
| receptor activity-modifying protein 1 precursor | 1 | | | o | | | | | |
| receptor activity-modifying protein 2 | 1 | o | | | | | | | |
| receptor activity-modifying protein 3 | 1 | | | | | | o | | |
| receptor tyrosine kinase flk-1 vegfr-2 | 1 | o | | | | | | | |
| receptor tyrosine-protein kinase erbb-3-like | 1 | | | | | | | | o |

| | | | | | | | | | |
|---|---|---|---|---|---|---|---|---|---|
| receptor-type tyrosine-protein phosphatase beta | 1 | | | | o | | | | |
| receptor-type tyrosine-protein phosphatase c isoform 2 | 1 | o | | | | | | | |
| receptor-type tyrosine-protein phosphatase delta-like | 1 | | | | | | o | | |
| receptor-type tyrosine-protein phosphatase f precursor | 1 | | | | | | | o | |
| receptor-type tyrosine-protein phosphatase kappa-like | 1 | | | | | | o | | |
| recq-mediated genome instability protein 2-like | 1 | | | | o | | | | |
| regulating synaptic membrane exocytosis protein 2 | 1 | | | | | | | | o |
| regulating synaptic membrane exocytosis protein 2-like | 1 | | | | | | o | | |
| regulator of calcineurin 1 | 1 | | | | | | | | o |
| regulator of differentiation 1 | 1 | | | | | | o | | |
| regulator of g-protein signaling 1 | 1 | | | | | | | | o |
| regulator of g-protein signaling 12 | 1 | | o | | | | | | |
| regulator of g-protein signaling 16 | 1 | | | | | | o | | |
| regulator of g-protein signaling 4 | 1 | | o | | | | | | |
| regulator of g-protein signaling 8 | 1 | | | | | | | | o |
| regulator of nonsense transcripts 1-like | 1 | | | o | | | | | |
| regulatory associated protein of complex 1 isoform 1 | 1 | o | | | | | | | |
| regulatory factor 1 (influences hla class ii expression) | 1 | | o | | | | | | |
| regulatory factor 3 (influences hla class ii expression) | 1 | | | | | | o | | |
| -related lipid transfer domain containing 7 | 1 | o | | | | | | | |
| related ras viral (r-ras) oncogene homolog | 1 | o | | | | | | | |
| relaxin 3a | 1 | | | | | o | | | |
| relaxin receptor 1-like | 1 | | | | | | o | | |
| relaxin-3 receptor 1 | 1 | | | | | | | | o |
| relaxin-3 receptor 1-like | 1 | | | | | | | | o |
| relt-like protein 2 | 1 | | | | | | o | | |
| renin binding protein | 1 | | o | | | | | | |
| renin receptor-like | 1 | | | | | | | | o |
| ReO_6 [Oryzias latipes] | 1 | | | | | | o | | |
| replication factor c subunit 5 | 1 | o | | | | | | | |
| replication protein a 14 kda subunit | 1 | | | | o | | | | |
| replication protein a 32 kda subunit | 1 | | | | o | | | | |
| replication protein a 32 kda subunit-like | 1 | | | | o | | | | |
| repulsive guidance molecule a | 1 | o | | | | | | | |
| required for meiotic nuclear division 5 homolog a | 1 | o | | | | | | | |
| required for meiotic nuclear division 5 homolog b ( cerevisiae) | 1 | o | | | | | | | |
| resistance to inhibitors of cholinesterase 8 homolog b ( elegans) | 1 | o | | | | | | | |
| restin isoform 2 | 1 | o | | | | | | | |
| ret proto-oncogene | 1 | | o | | | | | | |
| reticulon 1-a1 | 1 | | | | | | o | | |
| reticulon 4 receptor-like 1 | 1 | | | | | | | o | |
| reticulon 4 receptor-like 2 a | 1 | | | | | | | o | |
| retinitis pigmentosa 9 (autosomal dominant) | 1 | | o | | | | | | |
| retinoblastoma binding protein isoform cra_f | 1 | o | | | | | | | |
| retinoblastoma-associated protein | 1 | o | | | | | | | |

| | | | | | | | | | |
|---|---|---|---|---|---|---|---|---|---|
| retinoblastoma-associated protein 140 isoform 3 | 1 | o | | | | | | | |
| retinoblastoma-binding protein 6 isoform 1 | 1 | | | | | o | | | |
| retinoblastoma-binding protein 6 isoform 2 | 1 | | | o | | | | | |
| retinoblastoma-binding protein 8 | 1 | o | | | | | | | |
| retinoblastoma-like protein 1 | 1 | | | | | | o | | |
| retinoic acid induced 14 | 1 | o | | | | | | | |
| retinoic acid induced 17 | 1 | | | o | | | | | |
| retinoic acid receptor alpha | 1 | | o | | | | | | |
| retinoic acid receptor beta | 1 | | | | o | | | | |
| retinoic acid receptor beta-like | 1 | | | | o | | | | |
| retinoid x beta | 1 | | | | | o | | | |
| retinoid x gamma | 1 | | | | | o | | | |
| retinol dehydrogenase 13 | 1 | | o | | | | | | |
| retinol dehydrogenase 5 (11-cis 9-cis) | 1 | | o | | | | | | |
| retinol-binding protein cellular | 1 | | | | | | o | | |
| retrotransposon ty1-copia subclass | 1 | | o | | | | | | |
| retrotransposon-like family member (retr-1)- partial | 1 | | | | | | | o | |
| retrotransposon-like protein 1- partial | 1 | | | | | | | o | |
| retrovirus polyprotein | 1 | | | | | | o | | |
| rev3- catalytic subunit of dna polymerase zeta | 1 | o | | | | | | | |
| reverse transcriptase homolog | 1 | o | | | | | | | |
| rhamnose-binding lectin | 1 | | | o | | | | | |
| rho gdp-dissociation inhibitor 2 | 1 | | | | | | | o | |
| rho gtpase activating protein 11a | 1 | o | | | | | | | |
| rho gtpase activating protein 20 | 1 | | | | | | | o | |
| rho gtpase activating protein 25-like | 1 | o | | | | | | | |
| rho gtpase binding protein 2 | 1 | | o | | | | | | |
| rho gtpase-activating protein 1 | 1 | | | | | | o | | |
| rho gtpase-activating protein 11a isoform 1 | 1 | o | | | | | | | |
| rho gtpase-activating protein 12-like | 1 | | | | | | o | | |
| rho gtpase-activating protein 15 | 1 | | | | | o | | | |
| rho gtpase-activating protein 20-like | 1 | | | | | | | | o |
| rho gtpase-activating protein 21-like | 1 | | | | | | o | | |
| rho gtpase-activating protein 24-like | 1 | o | | | | | | | |
| rho gtpase-activating protein 27 | 1 | | | | | | | | o |
| rho gtpase-activating protein 29 | 1 | | o | | | | | | |
| rho gtpase-activating protein 32 isoform 1 | 1 | | | | | | | o | |
| rho gtpase-activating protein 39-like | 1 | | | | | | | | o |
| rho gtpase-activating protein 42-like | 1 | | | | | | | | o |
| rho gtpase-activating protein 44-like | 1 | | | | | | | | o |
| rho gtpase-activating protein 7-like | 1 | | | | o | | | | |
| rho gtpase-activating protein rich2 | 1 | | | | | | o | | |
| rho guanine exchange factor 16 | 1 | | | | o | | | | |
| rho guanine nucleotide exchange factor 10 | 1 | | | | | | o | | |
| rho guanine nucleotide exchange factor 12-like | 1 | | o | | | | | | |
| rho guanine nucleotide exchange factor 16 | 1 | | | | o | | | | |
| rho guanine nucleotide exchange factor 3 | 1 | o | | | | | | | |
| rho guanine nucleotide exchange factor 4-like | 1 | o | | | | | | | |
| rho-associated protein kinase 1 | 1 | o | | | | | | | |
| rho-associated protein kinase 2 | 1 | | | | | | o | | |
| rho-guanine nucleotide exchange factor | 1 | | | | | | o | | |
| rhomboid family 1 | 1 | o | | | | | | | |
| rho-related btb domain containing 2 | 1 | | | | | o | | | |
| rho-related gtp-binding protein | 1 | o | | | | | | | |
| rho-related gtp-binding protein precursor | 1 | | | | | | o | | |
| rho-type gtpase-activating protein flj32810-like isoform 2 | 1 | o | | | | | | | |

| | | | | | | | | | |
|---|---|---|---|---|---|---|---|---|---|
| riboflavin kinase-like | 1 | | | | | | | o | |
| riboflavin transporter 2 | 1 | | o | | | | | | |
| riboflavin transporter 2-like | 1 | | | | | | | | o |
| riboflavin-binding protein | 1 | | | | | | o | | |
| ribonuclease h1-like isoform 1 | 1 | | | | o | | | | |
| ribonuclease nuclear | 1 | o | | | | | | | |
| ribonuclease p mrp protein subunit pop5 | 1 | o | | | | | | | |
| ribonuclease p protein subunit p20 | 1 | | | | | | | | o |
| ribonuclease p protein subunit p29 | 1 | | o | | | | | | |
| ribonuclease p protein subunit p30 | 1 | | | | | | | o | |
| ribonuclease p protein subunit p38 | 1 | o | | | | | | | |
| ribonuclease subunit b | 1 | o | | | | | | | |
| ribonuclease zc3h12a-like | 1 | o | | | | | | | |
| ribose-5-phosphate isomerase | 1 | | o | | | | | | |
| ribosomal l24 domain containing 1 | 1 | | o | | | | | | |
| ribosomal protein l14 | 1 | | o | | | | | | |
| ribosomal protein l23a-like | 1 | | | | | | o | | |
| ribosomal protein l29 | 1 | | o | | | | | | |
| ribosomal protein l31 | 1 | | o | | | | | | |
| ribosomal protein l40 | 1 | | | o | | | | | |
| ribosomal protein s29 | 1 | | | | o | | | | |
| ribosomal protein s6 kinase alpha-3 | 1 | | | | | | o | | |
| ribosomal protein s6 kinase beta-1 isoform 3 | 1 | | | | | | o | | |
| ribosomal protein s6 kinase beta-2 | 1 | | | | o | | | | |
| ribosomal protein s6 polypeptide 3 | 1 | o | | | | | | | |
| ribosomal protein s6 polypeptide 5 | 1 | o | | | | | | | |
| ribosomal protein s6 polypeptide 6 | 1 | | | | | | | | o |
| ribosomal rna methyltransferase 2-like | 1 | | | | o | | | | |
| ribosomal rna processing 1 homolog b | 1 | o | | | | | | | |
| ribosomal rna processing protein 1 homolog a | 1 | o | | | | | | | |
| ribosomal rna processing protein 1 homolog b | 1 | | | | | | o | | |
| rieske (2fe-2s) domain-containing protein | 1 | | o | | | | | | |
| rieske domain-containing protein | 1 | o | | | | | | | |
| riken cdna 1300002k09 gene | 1 | | | o | | | | | |
| riken cdna 1700019e19 | 1 | | | | | o | | | |
| riken cdna 1810009a15 gene | 1 | | o | | | | | | |
| riken cdna 2310046k01 | 1 | | | | | o | | | |
| riken cdna 2610528e23 | 1 | o | | | | | | | |
| riken cdna 2810426n06 | 1 | | | | o | | | | |
| riken cdna 4930452b06 gene | 1 | | | | | | | o | |
| riken cdna 4932409i22 gene | 1 | | | o | | | | | |
| riken cdna 4933439f18 gene | 1 | o | | | | | | | |
| riken cdna 6430571l13 gene g20 protein isoform cra_a | 1 | o | | | | | | | |
| riken cdna 8430426h19 gene | 1 | | | | o | | | | |
| riken cdna e130309d02 gene | 1 | o | | | | | | | |
| rilp-like protein 2 | 1 | o | | | | | | | |
| rim-binding protein 2 | 1 | | | | | | o | | |
| ring finger and ccch-type zinc finger domain-containing protein 2-like | 1 | | | | | | o | | |
| ring finger and wd repeat domain 2 | 1 | | o | | | | | | |
| ring finger protein | 1 | | | | | | | | o |
| ring finger protein 11 | 1 | | | | | | o | | |
| ring finger protein 123 | 1 | o | | | | | | | |
| ring finger protein 128 | 1 | | o | | | | | | |
| ring finger protein 14 | 1 | | o | | | | | | |
| ring finger protein 157 | 1 | | | | | | o | | |
| ring finger protein 160 | 1 | | | o | | | | | |
| ring finger protein 20 | 1 | o | | | | | | | |
| ring finger protein 219 | 1 | | | | | | | | o |

| | | | | | | | | | |
|---|---|---|---|---|---|---|---|---|---|
| ring finger protein 29 | 1 | o | | | | | | | |
| ring finger protein 31- partial | 1 | | | | | | o | | |
| ring finger protein 34 | 1 | | | | | | o | | |
| ring finger protein 44 | 1 | | | | | | o | | |
| ring finger protein 6 | 1 | o | | | | | | | |
| ring hydroxylating alpha-subunit | 1 | | o | | | | | | |
| rio kinase 1 | 1 | o | | | | | | | |
| rio kinase 2 | 1 | o | | | | | | | |
| rna binding homolog 2 | 1 | o | | | | | | | |
| rna binding motif protein | 1 | | | | | o | | | |
| rna binding motif protein 12 | 1 | o | | | | | | | |
| rna binding motif protein 14 | 1 | | | | | | o | | |
| rna binding motif protein 17 isoform 2 | 1 | | | o | | | | | |
| rna binding motif protein 28 isoform 1 | 1 | o | | | | | | | |
| rna binding motif protein 28 isoform 2 | 1 | o | | | | | | | |
| rna binding motif protein 34 | 1 | | | | o | | | | |
| rna binding motif protein 39b | 1 | o | | | | | | | |
| rna binding motif protein 4 | 1 | | o | | | | | | |
| rna binding motif protein 47 | 1 | | o | | | | | | |
| rna binding motif protein 4b | 1 | o | | | | | | | |
| rna binding protein fox-1 homolog 3-like | 1 | | | | | | o | | |
| rna guanylyltransferase and 5 -phosphatase | 1 | o | | | | | | | |
| rna helicase lgp2 | 1 | o | | | | | | | |
| rna methyltransferase nol1 | 1 | | | | o | | | | |
| rna polymerase ii associated protein 1 | 1 | o | | | | | | | |
| rna polymerase ii associated protein 2 | 1 | | o | | | | | | |
| rna polymerase-associated protein rtf1 homolog | 1 | | o | | | | | | |
| rna pseudouridine synthase domain containing protein | 1 | | | | | | o | | |
| rna pseudouridylate synthase domain containing 4 | 1 | o | | | | | | | |
| rna pseudouridylate synthase domain-containing protein 2 | 1 | | | | o | | | | |
| rna recognition motif domain containing protein | 1 | o | | | | | | | |
| rna-binding protein 28 | 1 | | | | o | | | | |
| rna-binding protein 33-like | 1 | o | | | | | | | |
| rna-binding protein 40 | 1 | | | | | | o | | |
| rna-binding protein with serine-rich domain 1 | 1 | | o | | | | | | |
| rna-binding raly-like protein isoform 2 | 1 | | | | | | | o | |
| rna-binding single-stranded-interacting protein 2-like | 1 | | o | | | | | | |
| rna-dependent dna polymerase | 1 | | | | | | | o | |
| rna-directed dna partial | 1 | | | | | o | | | |
| rnase h and integrase-like protein | 1 | | | | | | o | | |
| rnf213 | 1 | o | | | | | | | |
| roundabout homolog 2 | 1 | | | | | | o | | |
| roundabout homolog 2-like | 1 | | | | | | o | | |
| rp42 homolog | 1 | o | | | | | | | |
| rpa-interacting protein a | 1 | | | | o | | | | |
| rpe-retinal g protein-coupled receptor | 1 | | | | | | | o | |
| rrna 2 -o-methyltransferase fibrillarin-like | 1 | | | | | | | | o |
| rrp15-like protein | 1 | o | | | | | | | |
| run and fyve domain containing 1 | 1 | o | | | | | | | |
| run and fyve domain containing 2 | 1 | | | | | | | | o |
| run and fyve domain-containing protein 1 isoform b | 1 | | | | | o | | | |
| run and fyve domain-containing protein 1-like | 1 | | o | | | | | | |
| run and fyve domain-containing protein 2 | 1 | | | | | | | | o |

| | | | | | | | | |
|---|---|---|---|---|---|---|---|---|
| run and sh3 domain containing 2 | 1 | | | | | o | | |
| run domain-containing protein 3a | 1 | | | | o | | | |
| run domain-containing protein 3b-like | 1 | | | | | o | | |
| runt-related transcription factor 1 translocated 1 (cyclin d-related) | 1 | o | | | | | | |
| ruvb-like 2 | 1 | o | | | | | | |
| rwd domain containing 1 | 1 | | | | o | | | |
| rwd domain containing 2b | 1 | o | | | | | | |
| rwd domain-containing protein 2b-like | 1 | | | o | | | | |
| rwd domain-containing protein 3 | 1 | | o | | | | | |
| s phase cyclin a-associated protein in the endoplasmic reticulum | 1 | | | | | | | o |
| s phase cyclin a-associated protein in the er | 1 | o | | | | | | |
| s100 calcium binding protein v2-like | 1 | | | | | | o | |
| sac3 domain-containing protein 1-like | 1 | | | o | | | | |
| s-acyl fatty acid synthase medium chain | 1 | | o | | | | | |
| safb- transcription isoform cra_a | 1 | o | | | | | | |
| safb-like transcription modulator-like | 1 | | | o | | | | |
| sal-like 1 | 1 | | o | | | | | |
| sal-like 2 | 1 | | | | | o | | |
| sal-like protein 2-like | 1 | | | | | | o | |
| sal-like protein 3-like | 1 | | | | | o | | |
| salt-inducible kinase 2 | 1 | o | | | | | | |
| sam domain-containing protein samsn-1 | 1 | o | | | | | | |
| samd9l protein | 1 | | o | | | | | |
| sap_chick ame: full=proactivator polypeptide contains: ame: full=saposin-a contains: ame: full=saposin-b contains: ame: full=saposin-c contains: ame: full=saposin-d flags: precursor | 1 | | o | | | | | |
| sap30 binding protein | 1 | o | | | | | | |
| saps domain family member 3 | 1 | | | | | | o | |
| saps3 protein | 1 | | | | | o | | |
| sarcalumenin | 1 | o | | | | | | |
| sarcoendoplasmic reticulum calcium atpase | 1 | o | | | | | | |
| sarcoglycan zeta | 1 | o | | | | | | |
| sarcoplasmic endoplasmic reticulum calcium atpase 1 | 1 | | | | | | o | |
| sarcoplasmic endoplasmic reticulum calcium atpase 3 | 1 | | | | | o | | |
| sarcosine dehydrogenase | 1 | | o | | | | | |
| saxitoxin and tetrodotoxin-binding protein 2-like | 1 | | o | | | | | |
| sc5a7_torma ame: full=high-affinity choline transporter 1 | 1 | o | | | | | | |
| scaffold attachment factor b | 1 | | | | | o | | |
| scaper protein | 1 | | o | | | | | |
| scavenger receptor class a member 5-like | 1 | | | | | | o | |
| scavenger receptor class member 3 | 1 | o | | | | | | |
| scavenger receptor class member 5 | 1 | o | | | | | | |
| scavenger receptor cysteine rich domain group b (4 domains)-like | 1 | o | | | | | | |
| scavenger receptor cysteine-rich type 1 protein cd163c-alpha | 1 | | o | | | | | |
| scavenger receptor cysteine-rich type 1 protein m130 | 1 | o | | | | | | |
| schwannomin interacting protein 1 | 1 | | | | | | o | |
| scinderin like a | 1 | | | | | | | o |
| scm-like with four mbt domains 1 | 1 | | | | | | | o |
| scm-like with four mbt domains 2 | 1 | | | | o | | | |
| scratch homolog zinc finger protein | 1 | | | | | o | | |

| | | | | | | | | | |
|---|---|---|---|---|---|---|---|---|---|
| scribbled homolog isoform 4 | 1 | | | | | | o | | |
| scy1-like 3 ( cerevisiae) | 1 | | | | o | | | | |
| seabream-type gonadotropin-releasing hormone precursor | 1 | | | | | | o | | |
| sec14 and spectrin domains 1 | 1 | | o | | | | | | |
| sec14l1 protein | 1 | o | | | | | | | |
| sec14-like protein 1 isoform a | 1 | | o | | | | | | |
| sec16 homolog a | 1 | | | | | | o | | |
| sec16 homolog a ( cerevisiae) | 1 | o | | | | | | | |
| sec23 homolog b ( cerevisiae) | 1 | | | o | | | | | |
| sec31 homolog b ( cerevisiae) | 1 | o | | | | | | | |
| sec61 alpha | 1 | | | o | | | | | |
| sec6-like 1 ( cerevisiae) | 1 | | | | | | o | | |
| secreted frizzled-related protein 1 | 1 | o | | | | | | | |
| secretion regulating guanine nucleotide exchange factor | 1 | | o | | | | | | |
| secretion-regulating guanine nucleotide exchange factor | 1 | | | | o | | | | |
| secretogranin ii | 1 | | | | | | | o | |
| secretory carrier membrane protein 3 | 1 | | | | | | o | | |
| secretory carrier-associated membrane protein 3 | 1 | | | | | | | | o |
| secretory phospholipase a2 receptor | 1 | o | | | | | | | |
| secretory phospholipase a2 receptor-like | 1 | | | | o | | | | |
| segment polarity protein dishevelled homolog dvl-1 | 1 | | | | | | o | | |
| seizure related 6 homolog | 1 | | | | | | | o | |
| selenium-binding protein | 1 | o | | | | | | | |
| selenium-binding protein 1 | 1 | | | | o | | | | |
| selenophosphate synthetase 1 | 1 | o | | | | | | | |
| selenoprotein ja | 1 | | o | | | | | | |
| selenoprotein m | 1 | | o | | | | | | |
| sema immunoglobulin domain short basic 3c | 1 | o | | | | | | | |
| sema immunoglobulin domain transmembrane domain and short cytoplasmic 4c | 1 | o | | | | | | | |
| semaphorin 3a precursor (semaphorin iii) (sema iii) isoform 3 | 1 | o | | | | | | | |
| semaphorin 4d | 1 | | o | | | | | | |
| semaphorin 6b | 1 | o | | | | | | | |
| semaphorin-4a precursor | 1 | o | | | | | | | |
| semaphorin-5a | 1 | | | | | | | | o |
| senp3b protein | 1 | | o | | | | | | |
| sentrin-specific protease 2 | 1 | | o | | | | | | |
| sentrin-specific protease 3 | 1 | | | | o | | | | |
| sepiapterin reductase | 1 | | | o | | | | | |
| sepm protein | 1 | | | | | | | | o |
| sepp1b protein | 1 | | | | o | | | | |
| sept7a protein | 1 | | | | | | | | o |
| sept8 protein | 1 | | | | | | | | o |
| septin 10 | 1 | | o | | | | | | |
| septin 6 | 1 | o | | | | | | | |
| ser thr-rich protein t10 in dgcr region | 1 | o | | | | | | | |
| serca (sarco-endoplasmic reticulum calcium atpase) family member (sca-1) | 1 | o | | | | | | | |
| serine (or cysteine) peptidase clade a (alpha-1 antitrypsin) member 10 | 1 | | o | | | | | | |
| serine (or cysteine) peptidase clade member 1a | 1 | | o | | | | | | |
| serine (or cysteine) proteinase clade a (alpha-1 antitrypsin) member 1 | 1 | | o | | | | | | |

| | | | | | | | | | |
|---|---|---|---|---|---|---|---|---|---|
| serine 27 | 1 | | | o | | | | | |
| serine active site containing 1 | 1 | o | | | | | | | |
| serine arginine repetitive matrix isoform 3 | 1 | | | | | | o | | |
| serine arginine-rich splicing factor 10-like | 1 | | o | | | | | | |
| serine arginine-rich splicing factor 6a | 1 | | | | | | | o | |
| serine arginine-rich splicing factor 7-like isoform 2 | 1 | | | | | | | | o |
| serine dehydratase-like | 1 | o | | | | | | | |
| serine hydrolase-like protein | 1 | | o | | | | | | |
| serine long chain base subunit 1 | 1 | | o | | | | | | |
| serine mitochondrial-like | 1 | | o | | | | | | |
| serine or cysteine proteinase inhibitor clade e member 1 | 1 | o | | | | | | | |
| serine peptidase 3 | 1 | o | | | | | | | |
| serine peptidase kunitz type 1 b | 1 | | | o | | | | | |
| serine protease | 1 | o | | | | | | | |
| serine protease 27-like | 1 | | | | o | | | | |
| serine protease inhibitor-like spi-1 protein | 1 | | | | | | o | | |
| serine protease mitochondrial-like | 1 | | | | | | | | o |
| serine protease precursor | 1 | o | | | | | | | |
| serine threonine kinase 3 (ste20 yeast) | 1 | | o | | | | | | |
| serine threonine kinase 32b | 1 | | | | | | | o | |
| serine threonine kinase 38 like | 1 | o | | | | | | | |
| serine threonine protein kinase araf | 1 | o | | | | | | | |
| serine threonine rich antigen | 1 | o | | | | | | | |
| serine threonine rich low complexity protein | 1 | o | | | | | | | |
| serine threonine-protein kinase 19-like | 1 | | | | o | | | | |
| serine threonine-protein kinase 25 | 1 | o | | | | | | | |
| serine threonine-protein kinase h1 | 1 | | | | o | | | | |
| serine threonine-protein kinase ick | 1 | | | | | | o | | |
| serine threonine-protein kinase mark1-like | 1 | | | | | | | | o |
| serine threonine-protein kinase n2 isoform 1 | 1 | o | | | | | | | |
| serine threonine-protein kinase nek10-like | 1 | | | | | o | | | |
| serine threonine-protein kinase nek2 | 1 | | | | o | | | | |
| serine threonine-protein kinase nek4 | 1 | o | | | | | | | |
| serine threonine-protein kinase nek7 | 1 | | | | o | | | | |
| serine threonine-protein kinase nek7-like | 1 | | | | o | | | | |
| serine threonine-protein kinase pak 2-like | 1 | | o | | | | | | |
| serine threonine-protein kinase pak 6 | 1 | | | | | | o | | |
| serine threonine-protein kinase pak 7 | 1 | | | | | | | o | |
| serine threonine-protein kinase pctaire-2 | 1 | | | | | | o | | |
| serine threonine-protein kinase pdik1l | 1 | | | | o | | | | |
| serine threonine-protein kinase prp4 homolog | 1 | o | | | | | | | |
| serine threonine-protein kinase sbk1-like | 1 | | | | | | | o | |
| serine threonine-protein kinase sgk1-like isoform 1 | 1 | o | | | | | | | |
| serine threonine-protein kinase sgk2 | 1 | | o | | | | | | |
| serine threonine-protein kinase srpk1 | 1 | | o | | | | | | |
| serine threonine-protein kinase srpk3 | 1 | o | | | | | | | |
| serine threonine-protein kinase srpk3-like | 1 | o | | | | | | | |
| serine threonine-protein kinase wnk1 | 1 | | | | | | o | | |
| serine threonine-protein kinase wnk1 isoform 2 | 1 | | | | | | | o | |
| serine threonine-protein kinase wnk4 | 1 | | | | | | | | o |
| serine threonine-protein phosphatase 2a 55 kda regulatory subunit b alpha isoform | 1 | | | | o | | | | |
| serine threonine-protein phosphatase 2a 55 kda regulatory subunit b delta isoform-like isoform 5 | 1 | | | | | o | | | |

| | | | | | | | | | |
|---|---|---|---|---|---|---|---|---|---|
| serine threonine-protein phosphatase 2a 55 kda regulatory subunit b gamma isoform | 1 | | | | | | o | | |
| serine threonine-protein phosphatase 2a 55 kda regulatory subunit b gamma isoform-like | 1 | | | | | | | o | |
| serine threonine-protein phosphatase 2a 65 kda regulatory subunit a beta isoform | 1 | | | | | | | o | |
| serine threonine-protein phosphatase 2a catalytic subunit alpha isoform-like protein | 1 | | | | | | | | o |
| serine threonine-protein phosphatase 2a regulatory subunit b subunit gamma | 1 | o | | | | | | | |
| serine threonine-protein phosphatase 2b catalytic subunit alpha isoform-like | 1 | | | | | | o | | |
| serine threonine-protein phosphatase 2b catalytic subunit gamma isoform | 1 | | | | | | o | | |
| serine threonine-protein phosphatase 4 regulatory subunit 1-like | 1 | | | | | | o | | |
| serine threonine-protein phosphatase 5 | 1 | o | | | | | | | |
| serine threonine-protein phosphatase 6 regulatory ankyrin repeat subunit a | 1 | | | | | | o | | |
| serine threonine-protein phosphatase 6 regulatory ankyrin repeat subunit a-like | 1 | | o | | | | | | |
| serine threonine-protein phosphatase 6 regulatory subunit 3 isoform 1 | 1 | o | | | | | | | |
| serine threonine-protein phosphatase with ef-hands 2 | 1 | | | | | | | o | |
| serotonin transporter | 1 | | o | | | | | | |
| serpin h1 precursor | 1 | o | | | | | | | |
| serpin peptidase clade a (alpha-1 antitrypsin) member 3 | 1 | | o | | | | | | |
| serpin peptidase clade b like | 1 | | | o | | | | | |
| serpin peptidase clade member 2b | 1 | | | | o | | | | |
| serrate rna effector molecule homolog isoform 1 | 1 | | o | | | | | | |
| serum paraoxonase arylesterase 2-like | 1 | | | | o | | | | |
| serum response factor binding protein 1 | 1 | o | | | | | | | |
| seryl-trna cytoplasmic | 1 | o | | | | | | | |
| sestrin 3 | 1 | | o | | | | | | |
| set and mynd domain containing 3 | 1 | o | | | | | | | |
| set domain containing 1a | 1 | | | | | | o | | |
| set1 ash2 histone methyltransferase complex subunit ash2-like | 1 | | o | | | | | | |
| sex hormone binding globulin | 1 | | | o | | | | | |
| sfrs protein kinase 3 | 1 | o | | | | | | | |
| sh2 domain containing 3c | 1 | | | | | | o | | |
| sh2 domain-containing adapter protein b | 1 | | | o | | | | | |
| sh2 domain-containing protein 3c isoform c | 1 | o | | | | | | | |
| sh3 and multiple ankyrin repeat domains 2 isoform 1 | 1 | | | | | | o | | |
| sh3 and multiple ankyrin repeat domains protein 1 | 1 | | | | | | o | | |
| sh3 domain and tetratricopeptide repeats 1 | 1 | | o | | | | | | |
| sh3 domain binding glutamic acid-rich protein like 2 | 1 | o | | | | | | | |
| sh3 domain binding glutamic acid-rich protein like 3 | 1 | o | | | | | | | |
| sh3 domain-binding glutamic acid-rich-like protein 3 | 1 | | o | | | | | | |
| sh3 domain-containing ring finger protein 3-like | 1 | o | | | | | | | |
| sh3-binding kinase 1-like | 1 | o | | | | | | | |

| | | | | | | | | | |
|---|---|---|---|---|---|---|---|---|---|
| sh3-domain grb2-like 2 | 1 | | | | | | | o | |
| sh3-domain grb2-like interacting protein 1 | 1 | | | | | | o | | |
| sh3-domain grb2-like interacting protein 1-like | 1 | | | | | | | o | |
| sh3kbp1-binding protein 1-like | 1 | | o | | | | | | |
| shc (src homology 2 domain containing) transforming protein 3 | 1 | | | | | | | o | |
| shc sh2 domain-binding protein 1 | 1 | | | | o | | | | |
| shc-transforming protein 1 | 1 | | | | | | o | | |
| shisa homolog 4 (xenopus laevis) | 1 | | | | | | | o | |
| shmt2 protein | 1 | | | | | o | | | |
| short transient receptor potential channel 5-like | 1 | | | | | | o | | |
| short transient receptor potential channel 7 isoform 3 | 1 | o | | | | | | | |
| short wavelength sensitive 2a | 1 | o | | | | | | | |
| short-chain dehydrogenase reductase family protei | 1 | | | | o | | | | |
| shroom family member 4 | 1 | o | | | | | | | |
| shugoshin-like 1 | 1 | o | | | | | | | |
| sialic acid binding ig-like lectin sialoadhesin | 1 | | | o | | | | | |
| sialin | 1 | | o | | | | | | |
| sid1 transmembrane family member 2-like | 1 | | | o | | | | | |
| sid1 transmembrane member 2 | 1 | o | | | | | | | |
| sidekick homolog 2 | 1 | | | | | | o | | |
| sideroflexin 3 | 1 | | | | | | o | | |
| sideroflexin 5 | 1 | | | | | | | | o |
| sidt2 protein | 1 | | | | | | | | o |
| sigma non-opioid intracellular receptor 1 | 1 | o | | | | | | | |
| signal cub and egf-like domain-containing protein 2-like | 1 | | o | | | | | | |
| signal cub egf-like 3 | 1 | | | | | | | o | |
| signal peptide peptidase-like 3-like | 1 | | | | | | o | | |
| signal recognition particle 54 | 1 | | | o | | | | | |
| signal recognition particle 68 kda protein | 1 | | o | | | | | | |
| signal recognition particle 72 kda protein | 1 | | | | o | | | | |
| signal sequence alpha | 1 | | | o | | | | | |
| signal transducer and activator of transcription 2 | 1 | | | | | | | | o |
| signal transducer and activator of transcription 4 | 1 | o | | | | | | | |
| signal transducer and activator of transcription 6 isoform 2 | 1 | o | | | | | | | |
| signal transducing adaptor family member 2 | 1 | | | | | | o | | |
| signal-induced proliferation-associated 1 like 3 | 1 | | | | | | o | | |
| signal-transducing adaptor protein 2-like | 1 | | | | | | | | o |
| similarity to ribosomal protein l5 | 1 | o | | | | | | | |
| sin3 homolog transcription regulator | 1 | | o | | | | | | |
| sin3a-associated 18kda | 1 | | | | | o | | | |
| sine oculis binding protein homolog | 1 | o | | | | | | | |
| sine oculis homeobox homolog | 1 | o | | | | | | | |
| sirtuin (silent mating type information regulation 2 homolog) 3 ( cerevisiae) | 1 | | | | o | | | | |
| sirtuin (silent mating type information regulation 2 homolog) 7 ( cerevisiae) | 1 | | | | | o | | | |
| sirtuin 1 | 1 | o | | | | | | | |
| sister chromatid cohesion protein dcc1 | 1 | | | | o | | | | |
| six homeobox 1 | 1 | o | | | | | | | |
| six homeobox 2 | 1 | o | | | | | | | |
| sjchgc03012 protein | 1 | o | | | | | | | |
| sjchgc03018 protein | 1 | | | o | | | | | |

| | | | | | | | | | |
|---|---|---|---|---|---|---|---|---|---|
| sjchgc03504 protein | 1 | | | | | | o | | |
| sjchgc07762 protein | 1 | | | | | o | | | |
| sjchgc07801 protein | 1 | | | | | | o | | |
| sjoegren syndrome scleroderma autoantigen 1 homolog | 1 | | | | | | o | | |
| sjogren syndrome nuclear autoantigen 1 | 1 | | | | o | | | | |
| skeletal muscle and kidney-enriched inositol phosphatase | 1 | o | | | | | | | |
| ski interacting protein | 1 | | o | | | | | | |
| skin mucus lectin | 1 | | o | | | | | | |
| slam family member 7-like | 1 | | | | | | | o | |
| s-layer domain protein | 1 | | | | | | o | | |
| slc24a4 protein | 1 | | | | | | | o | |
| slc9a3r2 protein | 1 | | | | | | o | | |
| slingshot homolog 1 | 1 | | | | | | o | | |
| slit and ntrk-like protein 3 | 1 | | | | | | o | | |
| slit and trk like 3 protein | 1 | | | | | | | o | |
| slit homolog 2 isoform 1 | 1 | | | | | | | o | |
| slit homolog 3 | 1 | | | o | | | | | |
| slit-robo rho gtpase-activating protein 1 | 1 | | | | | | o | | |
| slit-robo rho gtpase-activating protein 3-like | 1 | | | | | | | | o |
| smad nuclear interacting protein 1 | 1 | o | | | | | | | |
| smad4 protein | 1 | | | | o | | | | |
| small g protein signaling modulator 2 | 1 | o | | | | | | | |
| small glutamine-rich tetratricopeptide repeat-containing protein a | 1 | | o | | | | | | |
| small glutamine-rich tetratricopeptide repeat-containing protein alpha | 1 | | o | | | | | | |
| small glutamine-rich tetratricopeptide repeat-containing protein beta-like | 1 | | | | | | | | o |
| small gtpase ras-dva | 1 | | | | | | | o | |
| small inducible cytokine subfamily member 1 (endothelial monocyte-activating) | 1 | o | | | | | | | |
| small nuclear ribonucleoprotein polypeptide b | 1 | | | | | | | o | |
| small nuclear ribonucleoprotein polypeptide c | 1 | | | | | | | | o |
| small nuclear ribonucleoprotein sm d1 | 1 | | o | | | | | | |
| small nuclear ribonucleoprotein sm d3-like | 1 | | | | o | | | | |
| small nuclear rna activating polypeptide 3 | 1 | | | | | | | o | |
| small proline-rich protein 2h | 1 | | | | | | | o | |
| small serum protein 2-like | 1 | | o | | | | | | |
| small subunit of serine palmitoyltransferase a | 1 | o | | | | | | | |
| small subunit of serine palmitoyltransferase a-like | 1 | | | | o | | | | |
| small ubiquitin-related modifier 1 precursor | 1 | o | | | | | | | |
| smap1 protein | 1 | o | | | | | | | |
| smarca2 protein | 1 | o | | | | | | | |
| smarcc1 protein | 1 | | | | | | o | | |
| smarcc2 protein | 1 | | | | | | | | o |
| smc3 protein | 1 | o | | | | | | | |
| smc5 protein | 1 | o | | | | | | | |
| smek homolog suppressor of mek1 | 1 | o | | | | | | | |
| smfn protein | 1 | | | o | | | | | |
| smith-magenis syndrome chromosomal region candidate gene 7 | 1 | | | | o | | | | |
| smith-magenis syndrome chromosome candidate 7 | 1 | o | | | | | | | |
| smooth muscle myosin heavy chain 11 isoform 2 | 1 | o | | | | | | | |
| smoothelin | 1 | o | | | | | | | |
| smoothelin isoform 1 | 1 | | | | | | o | | |

| | | | | | | | | | |
|---|---|---|---|---|---|---|---|---|---|
| smyd family member 5 | 1 | o | | | | | | | |
| sn1-specific diacylglycerol lipase beta | 1 | o | | | | | | | |
| sn1-specific diacylglycerol lipase beta-like | 1 | o | | | | | | | |
| snail homolog 2 | 1 | o | | | | | | | |
| snap25-interacting protein | 1 | | | | | | o | | |
| snap47 protein | 1 | o | | | | | | | |
| snap-associated protein | 1 | | | | | | | | o |
| snf1-like kinase | 1 | o | | | | | | | |
| snf2-related cbp activator protein | 1 | | | | | | o | | |
| snph protein | 1 | | | | | | o | | |
| snrna-activating protein complex subunit 3 | 1 | | | | o | | | | |
| snrna-activating protein complex subunit 5 | 1 | | o | | | | | | |
| snrnp70 protein | 1 | o | | | | | | | |
| snurportin 1 | 1 | | | | o | | | | |
| snw domain-containing protein 1 | 1 | | o | | | | | | |
| socs box-containing wd protein s -2 | 1 | | | | | | | o | |
| sodium- and chloride-dependent gaba transporter 2 | 1 | | | | | | o | | |
| sodium- and chloride-dependent gaba transporter 3 | 1 | | | | | | | | o |
| sodium- and chloride-dependent glycine transporter 1 | 1 | | o | | | | | | |
| sodium channel and clathrin linker 1 | 1 | | | | o | | | | |
| sodium channel modifier 1 | 1 | o | | | | | | | |
| sodium channel protein type 2 subunit alpha | 1 | | | | | | | o | |
| sodium channel protein type 8 subunit alpha isoform 1 | 1 | | | | | | | o | |
| sodium channel protein type 8 subunit alpha-like isoform 3 | 1 | | | | | | o | | |
| sodium channel subunit beta-4-like | 1 | | | | | | | | o |
| sodium glucose cotransporter 1 | 1 | | o | | | | | | |
| sodium hydrogen exchanger 1 | 1 | o | | | | | | | |
| sodium hydrogen exchanger isoform 3 | 1 | o | | | | | | | |
| sodium leak channel non-selective protein | 1 | | | | | | o | | |
| sodium potassium atpase alpha subunit | 1 | | o | | | | | | |
| sodium potassium calcium exchanger 4 | 1 | | | | | | o | | |
| sodium potassium-transporting atpase alpha-1 | 1 | | | | | | o | | |
| sodium potassium-transporting atpase subunit beta-3 | 1 | | | | | o | | | |
| sodium voltage type alpha subunit | 1 | | | | | | o | | |
| sodium-coupled neutral amino acid transporter 4 | 1 | | | | | | o | | |
| sodium-coupled neutral amino acid transporter 7 | 1 | | | o | | | | | |
| sodium-dependent phosphate transporter 1-a | 1 | | | | | | o | | |
| sodium-driven chloride bicarbonate exchanger-like | 1 | | | | | | | | o |
| soluble calcium-activated nucleotidase 1 | 1 | | | | | | | | o |
| solute carrier family 1 (high affinity aspartate glutamate transporter) member 6 | 1 | | | | | | | | o |
| solute carrier family 10 (sodium bile acid cotransporter family) member 1 | 1 | | | | | | | o | |
| solute carrier family 12 (potassium chloride transporters) member 7 | 1 | | | | | | o | | |
| solute carrier family 12 (potassium-chloride transporter) member 5-like | 1 | | | | | | | o | |
| solute carrier family 12 member 2 | 1 | | | | | | o | | |
| solute carrier family 12 member 4-like | 1 | o | | | | | | | |
| solute carrier family 12 member 5 | 1 | | | | o | | | | |

| | | | | | | | | | |
|---|---|---|---|---|---|---|---|---|---|
| solute carrier family 12 member 9 | 1 | | o | | | | | | |
| solute carrier family 13 member 5-like | 1 | | o | | | | | | |
| solute carrier family 16 (monocarboxylic acid transporters) member 6 | 1 | | | | | | | o | |
| solute carrier family 17 (anion sugar transporter) member 5 | 1 | | | o | | | | | |
| solute carrier family 17 (sodium-dependent inorganic phosphate cotransporter) member 6 | 1 | | | | | | | | o |
| solute carrier family 2 (facilitated glucose transporter) member 2 | 1 | | | o | | | | | |
| solute carrier family 2 (facilitated glucose transporter) member 4 | 1 | | o | | | | | | |
| solute carrier family 2 (facilitated glucose transporter) member 9 | 1 | | o | | | | | | |
| solute carrier family 22 (extraneuronal monoamine transporter) member 3 | 1 | o | | | | | | | |
| solute carrier family 22 member 15 | 1 | o | | | | | | | |
| solute carrier family 22 member 17-like | 1 | | o | | | | | | |
| solute carrier family 22 member 23 | 1 | | | | | | | o | |
| solute carrier family 22 member 23-like | 1 | o | | | | | | | |
| solute carrier family 22 member 5 | 1 | o | | | | | | | |
| solute carrier family 22 member 5-like | 1 | | | | | | o | | |
| solute carrier family 22 member 6 | 1 | | o | | | | | | |
| solute carrier family 22 member ensg00000182157-like | 1 | | | | o | | | | |
| solute carrier family 23 member 2 | 1 | | o | | | | | | |
| solute carrier family 25 (carnitine acylcarnitine translocase) member 20 | 1 | | o | | | | | | |
| solute carrier family 25 (mitochondrial carrier adenine nucleotide translocator) member 6 | 1 | | o | | | | | | |
| solute carrier family 25 (mitochondrial carrier ornithine transporter) member 15 | 1 | o | | | | | | | |
| solute carrier family 25 (mitochondrial carrier phosphate carrier) member 25 | 1 | | o | | | | | | |
| solute carrier family 25 (mitochondrial carrier phosphate carrier) member 3 | 1 | | o | | | | | | |
| solute carrier family 25 member 33-like | 1 | | | | o | | | | |
| solute carrier family 25 member 44 | 1 | | o | | | | | | |
| solute carrier family 25 member 5 | 1 | o | | | | | | | |
| solute carrier family 27 (fatty acid transporter) member 2 | 1 | | o | | | | | | |
| solute carrier family 27 (fatty acid transporter) member 6 | 1 | | o | | | | | | |
| solute carrier family 30 (zinc transporter) member 7 | 1 | o | | | | | | | |
| solute carrier family 30 (zinc transporter) member 9 | 1 | | o | | | | | | |
| solute carrier family 33 (acetyl- transporter) member 1 | 1 | | o | | | | | | |
| solute carrier family 37 (glycerol-3-phosphate transporter) member 3 | 1 | | o | | | | | | |
| solute carrier family 39 (zinc transporter) member 12 | 1 | | | | | | | o | |
| solute carrier family 39 (zinc transporter) member 13 | 1 | o | | | | | | | |
| solute carrier family 4 (anion exchanger) member adaptor protein | 1 | o | | | | | | | |
| solute carrier family 4 sodium bicarbonate cotransporter member 7 | 1 | o | | | | | | | |

| | | | | | | | | | |
|---|---|---|---|---|---|---|---|---|---|
| solute carrier family 5 (sodium glucose cotransporter) member 2-like | 1 | o | | | | | | | |
| solute carrier family 5 (sodium iodide symporter) member 5 | 1 | | | | | o | | | |
| solute carrier family 6 (neurotransmitter betaine gaba) member isoform cra_a | 1 | | o | | | | | | |
| solute carrier family 6 (neurotransmitter transporter) member 19 | 1 | | o | | | | | | |
| solute carrier family 6 (neurotransmitter transporter) member isoform cra_a | 1 | | | | | | o | | |
| solute carrier family 6 (neutral amino acid transporter) member 19 | 1 | | o | | | | | | |
| solute carrier family 7 (cationic amino acid y+ system) member 1 | 1 | o | | | | | | | |
| solute carrier family 7 (cationic amino acid y+ system) member 2 | 1 | | o | | | | | | |
| solute carrier family 7 (cationic amino acid y+ system) member 3 | 1 | | | | | | o | | |
| solute carrier family 7 (cationic amino acid y+ system) member 5 | 1 | | | | | | o | | |
| solute carrier family 8 (sodium calcium exchanger) member 2 | 1 | o | | | | | | | |
| solute carrier family 8 (sodium calcium exchanger) member 4a | 1 | | | | | | o | | |
| solute carrier family 9 (sodium hydrogen exchanger) member 3 regulator 1 | 1 | o | | | | | | | |
| solute carrier family 9 (sodium hydrogen exchanger) member 6 | 1 | | | o | | | | | |
| solute carrier family anion member 1 (erythrocyte membrane protein band diego blood group) | 1 | o | | | | | | | |
| solute carrier family facilitated glucose transporter member 1 | 1 | | | | | | o | | |
| solute carrier family facilitated glucose transporter member 11-like | 1 | | o | | | | | | |
| solute carrier family facilitated glucose transporter member 3 | 1 | | | | | | o | | |
| solute carrier family facilitated glucose transporter member 4 | 1 | o | | | | | | | |
| solute carrier family facilitated glucose transporter member 5 | 1 | | o | | | | | | |
| solute carrier family facilitated glucose transporter member 8-like | 1 | | | | | | | | o |
| solute carrier family member 13 | 1 | o | | | | | | | |
| solute carrier family member 15 | 1 | o | | | | | | | |
| solute carrier family member 28 | 1 | | o | | | | | | |
| solute carrier family member 30 | 1 | o | | | | | | | |
| solute carrier family member 38 | 1 | | o | | | | | | |
| solute carrier family member 44 | 1 | o | | | | | | | |
| solute carrier family member a4 | 1 | | o | | | | | | |
| solute carrier family member b1 | 1 | | | o | | | | | |
| solute carrier family member b2 | 1 | | o | | | | | | |
| solute carrier family member c2 | 1 | | o | | | | | | |
| solute carrier family member f5 | 1 | | | | | | o | | |
| solute carrier family sodium bicarbonate transporter- member 11 | 1 | | o | | | | | | |
| solute carrier organic anion transporter family member 2b1-like | 1 | | | | | o | | | |
| solute carrier organic anion transporter family member 3a1 | 1 | | | | | | | | o |

| | | | | | | | | | |
|---|---|---|---|---|---|---|---|---|---|
| solute carrier organic anion transporter family member 5a1 | 1 | | | | | | o | | |
| solute carrier organic anion transporter member 1c1 isoform 1 | 1 | | o | | | | | | |
| solute carrier organic anion transporter member 2a1 | 1 | | o | | | | | | |
| solute carrier organic anion transporter member 2b1 | 1 | o | | | | | | | |
| solute carrier organic anion transporter member isoform cra_e | 1 | | | | | | | | o |
| somatolactin precursor | 1 | | | | o | | | | |
| somatostatin receptor type 5 | 1 | | o | | | | | | |
| somatostatin receptor type 5-like | 1 | | | | | | | | o |
| something about silencing protein 10-like | 1 | | | | o | | | | |
| son of sevenless homolog 1 | 1 | | | | | | o | | |
| sorbin and sh3 domain containing 1 | 1 | | | | | | o | | |
| sorbin and sh3 domain containing 2 | 1 | | | | | | | o | |
| sorbin and sh3 domain containing 2 protein | 1 | o | | | | | | | |
| sorbin and sh3 domain-containing protein 1 | 1 | | | | | | o | | |
| sortilin 1 | 1 | | o | | | | | | |
| sorting nexin 19a-like | 1 | o | | | | | | | |
| sorting nexin 4 | 1 | o | | | | | | | |
| sorting nexin 6 | 1 | o | | | | | | | |
| sorting nexin family member 27 | 1 | | o | | | | | | |
| sorting nexin-18 | 1 | | o | | | | | | |
| sorting nexin-19-like | 1 | | | | | | o | | |
| sorting nexin-22-like | 1 | | | | o | | | | |
| sorting nexin-24 | 1 | o | | | | | | | |
| sorting nexin-9 | 1 | | | | o | | | | |
| sp5 transcription factor | 1 | | | | | | o | | |
| spag9 protein | 1 | | | | | | | o | |
| spaghetti squash | 1 | | | | | o | | | |
| sparc related modular calcium binding 2 | 1 | o | | | | | | | |
| spastic paraplegia 21 (autosomal mast syndrome) | 1 | o | | | | | | | |
| spastic paraplegia paraplegin (pure and complicated autosomal recessive) | 1 | o | | | | | | | |
| spatacsin isoform 2 | 1 | | | | | o | | | |
| specifically androgen-regulated gene protein | 1 | | o | | | | | | |
| speckle-type poz | 1 | o | | | | | | | |
| spectrin alpha brain-like isoform 2 | 1 | | | | | | o | | |
| spectrin beta brain 1 | 1 | | o | | | | | | |
| spectrin beta brain 3-like | 1 | | | | | | | o | |
| spectrin beta -like | 1 | o | | | | | | | |
| spectrin repeat nuclear envelope 1 | 1 | o | | | | | | | |
| spectrin repeat nuclear envelope 2 | 1 | o | | | | | | | |
| spectrin sh3 domain binding protein 1 | 1 | | | | | | | o | |
| sperm associated antigen 1 | 1 | | | o | | | | | |
| sperm-associated antigen 7-like | 1 | | | | o | | | | |
| sperm-associated antigen 8-like | 1 | | | | | | | | o |
| spermatogenesis associated 2 | 1 | | | | o | | | | |
| spermatogenesis associated 5-like 1 | 1 | o | | | | | | | |
| spermatogenesis-associated protein 13 | 1 | | o | | | | | | |
| spermatogenesis-associated protein 7-like | 1 | | | | o | | | | |
| sphingomyelin phosphodiesterase 3 | 1 | | | | | | | | o |
| sphingomyelin phosphodiesterase 3-like | 1 | | | o | | | | | |
| sphingomyelin phosphodiesterase 4 | 1 | o | | | | | | | |
| sphingosine-1-phosphate receptor 1 | 1 | o | | | | | | | |
| spindle and kinetochore associated complex subunit 3 | 1 | | | | | o | | | |

| | | | | | | | | | |
|---|---|---|---|---|---|---|---|---|---|
| spindlin | 1 | | | | | | | o | |
| spire homolog 1 | 1 | | | | | | | o | |
| splicing arginine serine-rich 10 (transformer 2 drosophila) isoform cra_a | 1 | | | | | | | | o |
| splicing arginine serine-rich 13a | 1 | o | | | | | | | |
| splicing arginine serine-rich 16 | 1 | | | | | o | | | |
| splicing arginine serine-rich 18 | 1 | o | | | | | | | |
| splicing factor | 1 | o | | | | | | | |
| splicing factor 3a subunit 1 isoform 2 | 1 | | | | o | | | | |
| splicing factor 3a subunit 2 | 1 | | o | | | | | | |
| splicing factor subunit 1 | 1 | | o | | | | | | |
| splicing factor subunit 120kda | 1 | | | o | | | | | |
| splicing factor subunit 3 | 1 | o | | | | | | | |
| splicing factor subunit 66kda | 1 | o | | | | | | | |
| splicing factor u2af 35 kda subunit | 1 | | o | | | | | | |
| splicing factor u2af 65 kda subunit-like | 1 | | | | | | o | | |
| splicing proline- and glutamine-rich | 1 | | | | o | | | | |
| spondin-2 precursor | 1 | o | | | | | | | |
| sprouty 2-like | 1 | o | | | | | | | |
| sprouty homolog 2 | 1 | | | | o | | | | |
| sprouty homolog 4 | 1 | | | | | | o | | |
| spry domain-containing protein 3-like | 1 | o | | | | | | | |
| spry domain-containing protein 4-like | 1 | | | | o | | | | |
| spry domain-containing protein 7 | 1 | | | | o | | | | |
| spry domain-containing socs box protein 1-like | 1 | | | | | | o | | |
| spry domain-containing socs box protein 3-like | 1 | | | | | | o | | |
| squamous cell carcinoma antigen recognized by t cells | 1 | o | | | | | | | |
| squamous-cell carcinoma t-cell-recognized antigen | 1 | | | | | | | o | |
| src homology 2 domain containing f | 1 | | | o | | | | | |
| src homology 2 domain-containing transforming protein c3 | 1 | | | | | | o | | |
| src-family tyrosine kinase sck-like | 1 | o | | | | | | | |
| src-like isoform cra_b | 1 | o | | | | | | | |
| srcr membrane form partial | 1 | | o | | | | | | |
| srebp cleavage-activating protein | 1 | o | | | | | | | |
| srr1-like protein | 1 | o | | | | | | | |
| sr-related ctd-associated factor 1 | 1 | o | | | | | | | |
| sry (sex determining region y)-box 4 | 1 | o | | | | | | | |
| sry-box containing gene 11a | 1 | o | | | | | | | |
| sry-box containing gene 1a | 1 | | | | | o | | | |
| sry-box containing gene 4 | 1 | | | | | | o | | |
| sry-box containing gene 6b | 1 | o | | | | | | | |
| sry-box containing gene 7 | 1 | o | | | | | | | |
| ss18-like protein 2 | 1 | o | | | | | | | |
| st14a protein | 1 | o | | | | | | | |
| st3 beta-galactoside alpha- -sialyltransferase 3 | 1 | | o | | | | | | |
| st3 beta-galactoside alpha- -sialyltransferase isoform cra_c | 1 | | o | | | | | | |
| st6 (alpha-n-acetyl-neuraminyl- -beta-galactosyl- )-n-acetylgalactosaminide alpha- -sialyltransferase 2 | 1 | o | | | | | | | |
| st8 alpha-n-acetyl-neuraminide alpha- -sialyltransferase 1 | 1 | | | | | | o | | |
| st8 alpha-n-acetyl-neuraminide alpha- -sialyltransferase 5 | 1 | | | | | | | o | |
| stabilin 2 | 1 | | | | | o | | | |

| | | | | | | | | | |
|---|---|---|---|---|---|---|---|---|---|
| stanniocalcin 2 | 1 | | o | | | | | | |
| staphylococcal nuclease domain-containing protein 1 | 1 | | o | | | | | | |
| starch branching enzyme iib | 1 | | | | | | | | o |
| star-related lipid transfer protein 13 | 1 | | o | | | | | | |
| start domain containing 10 | 1 | | o | | | | | | |
| start domain containing 3 | 1 | | | o | | | | | |
| stathmin-4-like isoform 2 | 1 | | | | | | o | | |
| stathmin-like 4 | 1 | | | | | | o | | |
| ste20-like serine threonine-protein kinase-like isoform 2 | 1 | | | | | | | o | |
| ste20-related kinase adapter protein alpha | 1 | | | | | | o | | |
| steap family member 4 | 1 | | o | | | | | | |
| stem-loop binding protein | 1 | o | | | | | | | |
| sterile alpha and tir motif-containing protein 1 | 1 | | | | | | | | o |
| sterile alpha motif domain containing 8 | 1 | | o | | | | | | |
| sterile alpha motif domain containing 9 | 1 | | o | | | | | | |
| sterile alpha motif domain-containing protein 13-like | 1 | | | | | | o | | |
| steroid sulfatase | 1 | | o | | | | | | |
| sterol 26- mitochondrial-like | 1 | | | | o | | | | |
| sterol carrier protein x-related thiolase | 1 | | | o | | | | | |
| sterol regulatory element binding transcription factor 2 | 1 | | o | | | | | | |
| sterol regulatory element-binding protein 1 | 1 | | o | | | | | | |
| sterol regulatory element-binding protein 2 | 1 | | | | | | o | | |
| stim2 protein | 1 | o | | | | | | | |
| stk35 protein | 1 | | | | o | | | | |
| stomatin -like 2 | 1 | o | | | | | | | |
| stomatin-like protein 1 | 1 | | | | o | | | | |
| stonin-1 | 1 | | o | | | | | | |
| stonustoxin alpha-subunit | 1 | o | | | | | | | |
| stonustoxin subunit alpha-like | 1 | | | | | | o | | |
| stress-70 mitochondrial | 1 | | o | | | | | | |
| stress-induced phosphoprotein 1 | 1 | | o | | | | | | |
| stretchin- isoform d | 1 | o | | | | | | | |
| stromal cell-derived factor 1 precursor | 1 | o | | | | | | | |
| stromal cell-derived factor 1a | 1 | o | | | | | | | |
| stromal membrane-associated protein 1 | 1 | | | | | | o | | |
| strongly-conserved zn-finger binding protein | 1 | o | | | | | | | |
| structural maintenance of | 1 | o | | | | | | | |
| subfamily member 14 | 1 | | | | | o | | | |
| subfamily member 16 | 1 | | | | | | o | | |
| subfamily member 17 | 1 | | | | o | | | | |
| subfamily member a1 | 1 | | | o | | | | | |
| subfamily member a3-like | 1 | | | | | | | o | |
| succinate dehydrogenase assembly factor mitochondrial | 1 | | | | | o | | | |
| succinate dehydrogenase subunit flavoprotein | 1 | o | | | | | | | |
| succinate dehydrogenase subunit integral membrane protein | 1 | | | | | o | | | |
| sugar phosphate exchanger 2 | 1 | | | o | | | | | |
| sugar phosphate exchanger 3 | 1 | | | | | | | | o |
| sugar phosphate exchanger 3-like | 1 | | | | | | | | o |
| sugar transporter sweet1-like | 1 | | | | | | | | o |
| sulfatase isoform cra_a | 1 | o | | | | | | | |
| sulfatase modifying factor 2 | 1 | o | | | | | | | |
| sulfatase-modifying factor 1 precursor | 1 | o | | | | | | | |
| sulfated glycoprotein 1 isoform c preproprotein | 1 | | o | | | | | | |

| | | | | | | | | | |
|---|---|---|---|---|---|---|---|---|---|
| sulfite oxidase | 1 | | o | | | | | | |
| sulfotransferase 4a1 | 1 | | | | | | | o | |
| sumo1 sentrin specific peptidase 1 | 1 | | o | | | | | | |
| sumo1 sentrin specific peptidase 6 | 1 | | o | | | | | | |
| sumo-activating enzyme subunit 2 | 1 | o | | | | | | | |
| superkiller viralicidic activity 2-like ( cerevisiae) | 1 | | | | | | | o | |
| suppression of tumorigenicity 1 | 1 | o | | | | | | | |
| suppression of tumorigenicity 5 | 1 | | | o | | | | | |
| suppressor of cytokine signaling 2 | 1 | | | o | | | | | |
| suppressor of cytokine signaling 9 | 1 | o | | | | | | | |
| suppressor of g2 allele of skp1 | 1 | | o | | | | | | |
| suppressor of sable-like | 1 | o | | | | | | | |
| suppressor of tumorigenicity 7 protein homolog | 1 | | | | | | | | o |
| suppressor of variegation 4-20 homolog 1 | 1 | o | | | | | | | |
| suppressor of variegation 4-20 homolog 2 | 1 | o | | | | | | | |
| suppressor of ypt1 | 1 | | o | | | | | | |
| surf6_fugru ame: full=surfeit locus protein 6 homolog | 1 | | | | | o | | | |
| surface antigen protein 2 | 1 | | | | o | | | | |
| surface-anchored protein | 1 | o | | | | | | | |
| surfeit 4 | 1 | | o | | | | | | |
| surfeit locus protein 1 | 1 | | | | o | | | | |
| survival motor neuron protein | 1 | | | | | o | | | |
| survival of motor neuron protein-interacting protein 1 | 1 | o | | | | | | | |
| sushi repeat-containing protein srpx2 precursor | 1 | o | | | | | | | |
| sushi-domain containing secreted protein with a signal low complexity region followed by a sushi domain | 1 | o | | | | | | | |
| sushi-repeat-containing protein | 1 | | | o | | | | | |
| sushi-repeat-containing x-linked | 1 | o | | | | | | | |
| suv420h2 protein | 1 | o | | | | | | | |
| swi snf-related matrix-associated actin-dependent regulator of chromatin subfamily a-like protein 1-like | 1 | | | | o | | | | |
| swi snf-related matrix-associated actin-dependent regulator of chromatin subfamily b member 1 | 1 | | o | | | | | | |
| switch-associated protein 70-like | 1 | | | | | | | | o |
| sy63_disom ame: full=synaptotagmin-c ame: full=synaptic vesicle protein o-p65-c | 1 | o | | | | | | | |
| synapse associated protein sap47 homolog | 1 | | o | | | | | | |
| synapse-associated protein 1 | 1 | | o | | | | | | |
| synapsin i | 1 | | | | | | o | | |
| synapsin ii | 1 | | | | | | o | | |
| synapsin iii | 1 | o | | | | | | | |
| synapsin iii isoform 1 | 1 | | | | | | o | | |
| synapsin-3 variant | 1 | | | | | | | o | |
| synaptic ras gtpase activating protein 1 homolog | 1 | | | | | | | o | |
| synaptic vesicle 2-related protein | 1 | | | | | | o | | |
| synaptic vesicle glycoprotein 2a-like | 1 | | | | | | o | | |
| synaptic vesicle glycoprotein 2c | 1 | | | | | | o | | |
| synaptic vesicle membrane protein vat-1 homolog | 1 | o | | | | | | | |
| synaptic vesicle protein 2b homolog | 1 | | | o | | | | | |
| synaptobrevin homolog ykt6 | 1 | | o | | | | | | |

| | | | | | | | | | |
|---|---|---|---|---|---|---|---|---|---|
| synaptojanin 1 isoform a | 1 | | | | | | o | | |
| synaptonemal complex protein 3 | 1 | | | o | | | | | |
| synaptosomal-associated 91kda homolog | 1 | | | | | | o | | |
| synaptotagmin 1 | 1 | | | | | | o | | |
| synaptotagmin cytoplasmic rna interacting protein | 1 | o | | | | | | | |
| synaptotagmin iia | 1 | | | | | o | | | |
| synaptotagmin isoform cra_c | 1 | | | | | | | | o |
| synaptotagmin iv | 1 | | | | | | o | | |
| synaptotagmin xvi | 1 | o | | | | | | | |
| syncrip protein | 1 | | o | | | | | | |
| syndecan 2 | 1 | o | | | | | | | |
| syndecan 3 | 1 | | | | | o | | | |
| syndecan 3-like | 1 | | | | | | | o | |
| synj1 protein | 1 | | | | | | o | | |
| synovial x breakpoint 2 interacting protein | 1 | | | | o | | | | |
| syntaxin 19 | 1 | o | | | | | | | |
| syntaxin 1a | 1 | | o | | | | | | |
| syntaxin binding protein 2 | 1 | o | | | | | | | |
| syntaxin binding protein 5 | 1 | | | o | | | | | |
| syntaxin binding protein 5 isoform 2 | 1 | | | | | | o | | |
| syntaxin binding protein 5-like | 1 | o | | | | | | | |
| syntaxin binding protein 6 | 1 | | | | | o | | | |
| syntaxin-binding protein 1-like | 1 | | | | | | | | o |
| syntaxin-binding protein 2 | 1 | | | | o | | | | |
| sys1 homolog | 1 | o | | | | | | | |
| syt7 partial | 1 | | | | | | | o | |
| t calcium channel alpha 1g subunit variant 88 | 1 | | | | | | o | | |
| t cell receptor alpha | 1 | o | | | | | | | |
| t- immune regulator h+ lysosomal v0 subunit a3 | 1 | o | | | | | | | |
| tachykinin 3 | 1 | | | | | o | | | |
| tachykinin receptor 1 | 1 | | | | | | o | | |
| taf10 rna polymerase tata box binding protein -associated 30kda | 1 | | | | | | | o | |
| tafazzin | 1 | o | | | | | | | |
| tail-specific thyroid hormone up-regulated (gene 5) | 1 | o | | | | | | | |
| talin 2 | 1 | o | | | | | | | |
| tao kinase 1 | 1 | o | | | | | | | |
| tapasin-related protein | 1 | o | | | | | | | |
| tap-binding protein | 1 | | o | | | | | | |
| target of rapamycin complex 2 subunit mapkap1 | 1 | o | | | | | | | |
| targeting protein for xklp2 | 1 | o | | | | | | | |
| tata box binding protein -associated rna polymerase 48kda | 1 | | o | | | | | | |
| tata box-binding protein 1 | 1 | | | | | | | | o |
| tata element modulatory factor 1 | 1 | o | | | | | | | |
| tau-tubulin kinase 1 | 1 | | | | | | | | o |
| tau-tubulin kinase 2 | 1 | | | | | | o | | |
| tax1 (human t-cell leukemia virus type i) binding protein 1 | 1 | o | | | | | | | |
| tax1-binding protein 1 homolog isoform 1 | 1 | | o | | | | | | |
| tbc (tre-2 bub2 cdc16) domain family member (tbc-2)- partial | 1 | | | o | | | | | |
| tbc1 domain family member 5 | 1 | o | | | | | | | |
| tbc1 domain family member 8b | 1 | | | o | | | | | |
| tbc1 domain family member 9 | 1 | | | | | | | | o |
| tbc1 domain member 10a | 1 | | o | | | | | | |

| | | | | | | | | | |
|---|---|---|---|---|---|---|---|---|---|
| tbc1 domain member 14 | 1 | | | | | o | | | |
| tbc1 domain member 25 | 1 | o | | | | | | | |
| tbc1 domain member 7 | 1 | o | | | | | | | |
| tbc1 domain member 8 | 1 | | o | | | | | | |
| tbc1 domain member 8 (with gram domain) | 1 | o | | | | | | | |
| t-box 1 | 1 | | | | o | | | | |
| t-box brain protein 1 | 1 | o | | | | | | | |
| tbp-associated factor 4-like | 1 | o | | | | | | | |
| tbp-like 1 | 1 | o | | | | | | | |
| tbt-binding protein | 1 | | | o | | | | | |
| tcb1 transposase | 1 | o | | | | | | | |
| tcdd-inducible poly(adp-ribose) polymerase | 1 | | | o | | | | | |
| t-cell activation rho gtpase-activating protein | 1 | | | | o | | | | |
| t-cell lymphoma invasion and metastasis 2 | 1 | o | | | | | | | |
| t-cell surface antigen cd2 precursor | 1 | | | | o | | | | |
| t-cell-specific surface glycoprotein cd28 precursor | 1 | | | o | | | | | |
| tcf3 protein | 1 | | | | | | o | | |
| tcf7l2 protein | 1 | o | | | | | | | |
| t-complex protein 1 subunit alpha-like | 1 | | | | | | | | o |
| t-complex protein 1 subunit eta-like | 1 | | | | | | | | o |
| t-complex protein 1 subunit gamma-like isoform 2 | 1 | | | | o | | | | |
| t-complex protein 1 subunit theta-like | 1 | | | | | | | | o |
| tdp-glucose -dehydratase | 1 | o | | | | | | | |
| tdrd9_danre ame: full= atp-dependent rna helicase tdrd9 ame: full=tudor domain-containing protein 9 | 1 | o | | | | | | | |
| tea domain family member 4 | 1 | o | | | | | | | |
| tead1 protein | 1 | o | | | | | | | |
| teashirt family zinc finger 1 | 1 | o | | | | | | | |
| teashirt homolog 3-like | 1 | | | | | | | | o |
| teashirt zinc finger homeobox 3 | 1 | o | | | | | | | |
| tec protein tyrosine isoform cra_a | 1 | | | | o | | | | |
| tec protein tyrosine kinase | 1 | o | | | | | | | |
| tectonin beta-propeller repeat-containing protein 1 | 1 | o | | | | | | | |
| telomerase protein component 1 | 1 | o | | | | | | | |
| telomerase protein component 1-like | 1 | | o | | | | | | |
| telomerase reverse transcriptase | 1 | | | | o | | | | |
| telomerase-associated protein 1 | 1 | o | | | | | | | |
| telomeric repeat binding factor (nima-interacting) 1 | 1 | | o | | | | | | |
| telomeric repeat binding factor 1 | 1 | | o | | | | | | |
| temporarily assigned gene name family member (tag-163)-like | 1 | o | | | | | | | |
| tenascin n | 1 | o | | | | | | | |
| teneurin-2 isoform 1 | 1 | | | | | | o | | |
| tensin | 1 | o | | | | | | | |
| tensin 1 | 1 | | | | | | o | | |
| tensin 3 | 1 | | o | | | | | | |
| terminal uridylyltransferase 4 | 1 | o | | | | | | | |
| testis derived transcript | 1 | o | | | | | | | |
| testis-expressed sequence 10 protein homolog | 1 | o | | | | | | | |
| testis-expressed sequence 10 protein isoform 2 | 1 | | | | o | | | | |
| testis-expressed sequence 12 protein | 1 | o | | | | | | | |
| tetracycline transporter-like protein | 1 | o | | | | | | | |
| tetraspanin 11 | 1 | | o | | | | | | |
| tetraspanin 12 | 1 | o | | | | | | | |

| | | | | | | | | | |
|---|---|---|---|---|---|---|---|---|---|
| tetraspanin 14 | 1 | | | o | | | | | |
| tetraspanin 18b | 1 | | | | | | | | o |
| tetraspanin 9 | 1 | o | | | | | | | |
| tetraspanin isoform cra_b | 1 | | | | | | o | | |
| tetraspanin-1 | 1 | | o | | | | | | |
| tetraspanin-13-like | 1 | | | | o | | | | |
| tetraspanin-3-like isoform 1 | 1 | | | | | | | | o |
| tetraspanin-4 | 1 | | | | | | | | o |
| tetratricopeptide ankyrin repeat and coiled-coil containing 2 | 1 | o | | | | | | | |
| tetratricopeptide repeat domain 8 | 1 | o | | | | | | | |
| tetratricopeptide repeat domain isoform cra_a | 1 | o | | | | | | | |
| tetratricopeptide repeat protein 17 | 1 | | | | o | | | | |
| tetratricopeptide repeat protein 39b | 1 | | | | o | | | | |
| tetratricopeptide repeat protein 4 | 1 | | | | o | | | | |
| tetratricopeptide repeat protein 5 | 1 | | | | | | | | o |
| tfiia p55 | 1 | o | | | | | | | |
| tfiih basal transcription factor complex helicase subunit | 1 | | | | | | | | o |
| tgf-beta inducible early protein | 1 | o | | | | | | | |
| tgf-beta receptor type-2 | 1 | o | | | | | | | |
| thap domain apoptosis associated protein 1 | 1 | o | | | | | | | |
| thap domain containing 11 | 1 | | | | o | | | | |
| thap domain containing 11-like | 1 | o | | | | | | | |
| thap domain containing 4 | 1 | o | | | | | | | |
| thap domain containing 6 | 1 | o | | | | | | | |
| thap domain-containing protein 11 | 1 | | | | | o | | | |
| thap domain-containing protein 5 | 1 | | | | o | | | | |
| thap domain-containing protein 9-like | 1 | | | | | o | | | |
| thiamine-triphosphatase | 1 | | | | | | | | o |
| thiazide sensitive na-cl co- solute carrier family member 3 | 1 | o | | | | | | | |
| thioesterase superfamily member 2 | 1 | o | | | | | | | |
| thiolester containing protein ii- partial | 1 | | | o | | | | | |
| thioredoxin domain containing 15 | 1 | | o | | | | | | |
| thioredoxin domain-containing protein 1 | 1 | | | | o | | | | |
| thioredoxin domain-containing protein 15-like | 1 | | | | | | | | o |
| thioredoxin reductase 3 | 1 | | o | | | | | | |
| thioredoxin-like 4b | 1 | o | | | | | | | |
| thioredoxin-like protein 1 | 1 | | o | | | | | | |
| tho complex 7 homolog | 1 | | o | | | | | | |
| tho complex subunit 2 | 1 | | | | | | o | | |
| tho complex subunit 2-like | 1 | | | | | | o | | |
| threonine aldolase 1 | 1 | | o | | | | | | |
| threonyl-trna synthetase-like 2 | 1 | | o | | | | | | |
| thrombin protein | 1 | | | | o | | | | |
| thrombospondin 4 | 1 | o | | | | | | | |
| thrombospondin type-1 domain-containing protein 7a-like | 1 | | | | | | | o | |
| thump domain containing 2 | 1 | o | | | | | | | |
| thump domain containing 3 | 1 | o | | | | | | | |
| thump domain-containing protein 1-like | 1 | | | | o | | | | |
| thymidine kinase mitochondrial | 1 | | | | | o | | | |
| thymidine phosphorylase | 1 | | o | | | | | | |
| thymidylate synthase | 1 | o | | | | | | | |
| thymine-dna glycosylase-like | 1 | o | | | | | | | |
| thymine-dna isoform cra_a | 1 | o | | | | | | | |
| thymocyte selection-associated high mobility group box protein tox | 1 | | | | | | o | | |
| thymosin beta-4 | 1 | | | | o | | | | |

| | | | | | | | | | |
|---|---|---|---|---|---|---|---|---|---|
| thyroid adenoma-associated protein homolog | 1 | | | | | | | | o |
| thyroid hormone receptor interactor 10 | 1 | | o | | | | | | |
| thyroid hormone receptor-associated protein 3 | 1 | o | | | | | | | |
| thyroid transcription factor 1-associated protein 26 | 1 | | | | | | | | o |
| thyrotroph embryonic factor | 1 | | | | | | o | | |
| thyrotrophic embryonic factor | 1 | | o | | | | | | |
| thyrotropin beta subunit | 1 | | | | o | | | | |
| thyrotropin-releasing hormone degrading enzyme | 1 | | | | | | | o | |
| tight junction protein 1 (zona occludens 1) | 1 | o | | | | | | | |
| tight junction protein zo-1-like | 1 | | | o | | | | | |
| timeless interacting protein | 1 | o | | | | | | | |
| tiny fragments locus 9c | 1 | o | | | | | | | |
| tip41-like protein | 1 | o | | | | | | | |
| tissue factor pathway inhibitor 2 | 1 | | | | o | | | | |
| titin b | 1 | o | | | | | | | |
| tlc domain containing 1 | 1 | o | | | | | | | |
| t-lymphoma invasion and metastasis-inducing protein 1-like | 1 | | | | o | | | | |
| tmem132d protein | 1 | o | | | | | | | |
| tmem16d protein | 1 | | o | | | | | | |
| tmem214 protein | 1 | | o | | | | | | |
| tmo-4c4 | 1 | o | | | | | | | |
| tnf receptor-associated factor 2 | 1 | | o | | | | | | |
| tnf receptor-associated factor 2-like | 1 | | o | | | | | | |
| tnf receptor-associated factor 3 | 1 | | o | | | | | | |
| tnf receptor-associated factor 3-like isoform 1 | 1 | | o | | | | | | |
| tnf superfamily member 14 | 1 | | | o | | | | | |
| tnf-alpha converting enzyme | 1 | | | | | | | o | |
| toll interacting protein | 1 | | o | | | | | | |
| toll-like receptor 22 | 1 | o | | | | | | | |
| toll-like receptor adaptor molecule | 1 | o | | | | | | | |
| toll-like receptor adaptor molecule 1 | 1 | o | | | | | | | |
| tolloid-like 2 | 1 | | o | | | | | | |
| tom1 protein | 1 | | o | | | | | | |
| tom1-like protein 2 | 1 | | | | | | o | | |
| top2a protein | 1 | | | | o | | | | |
| topoisomerase like | 1 | | | | | | | o | |
| torsin a interacting protein 2 | 1 | | | o | | | | | |
| torsin family member a | 1 | | o | | | | | | |
| torsin family member a (torsin a) | 1 | | | o | | | | | |
| torsin-1a-interacting protein 2-like | 1 | | | | | | | | o |
| tousled-like kinase 2 | 1 | | | | | | | o | |
| tox high mobility group box family member 4 | 1 | | | o | | | | | |
| tp53 apoptosis effector | 1 | o | | | | | | | |
| tp53 dependent g2 arrest mediator candidate | 1 | | | | | o | | | |
| tp53-regulated inhibitor of apoptosis 1 | 1 | o | | | | | | | |
| tp53rk-binding protein | 1 | o | | | | | | | |
| tpa: leukemia inhibitory factor | 1 | | o | | | | | | |
| tpa: lim-domain-binding protein 4b | 1 | | o | | | | | | |
| tpa: zinc finger protein | 1 | o | | | | | | | |
| tpa_exp: claudin 25b | 1 | | o | | | | | | |
| tpa_exp: claudin 3a | 1 | o | | | | | | | |
| tpa_exp: claudin 3b | 1 | | | o | | | | | |
| tpa_exp: fxyd7b | 1 | | | | | o | | | |
| tpa_exp: pol polyprotein | 1 | o | | | | | | | |
| tpa_exp: ubiquitin | 1 | | o | | | | | | |
| tpa_inf: aldehyde reductase | 1 | | | o | | | | | |

| | | | | | | | | | |
|---|---|---|---|---|---|---|---|---|---|
| tpa_inf: fam20a | 1 | o | | | | | | | |
| tpa_inf: fam20c4 | 1 | | | o | | | | | |
| tpa_inf: twist2 | 1 | | | | | | o | | |
| tpa-induced transmembrane protein homolog | 1 | o | | | | | | | |
| tpr domain protein | 1 | o | | | | | | | |
| trab domain-containing protein | 1 | | | o | | | | | |
| traf family member-associated nfkb activator | 1 | o | | | | | | | |
| traf2 and nck-interacting protein kinase-like | 1 | | | | | | o | | |
| trafficking protein particle complex 1 | 1 | | o | | | | | | |
| trafficking protein particle complex 9 | 1 | o | | | | | | | |
| trafficking protein particle complex subunit 10 | 1 | | | | | | o | | |
| traf-interacting protein | 1 | | | | o | | | | |
| traf-type zinc finger domain containing 1 | 1 | o | | | | | | | |
| traf-type zinc finger domain-containing protein 1 | 1 | | | o | | | | | |
| trans- -enoyl- reductase | 1 | | o | | | | | | |
| trans- -enoyl- reductase-like | 1 | | | | | | | | o |
| trans golgi network protease furin | 1 | | | o | | | | | |
| trans-2-enoyl- mitochondrial precursor | 1 | | o | | | | | | |
| transcription cofactor vestigial-like protein 2 | 1 | o | | | | | | | |
| transcription factor 12 | 1 | o | | | | | | | |
| transcription factor 7-like 1 (t-cell hmg-box) | 1 | | | | | | | o | |
| transcription factor 7-like 2 (t-cell hmg-box) | 1 | | | | | | o | | |
| transcription factor ap-2 beta | 1 | | | | | o | | | |
| transcription factor binding to ighm enhancer 3a | 1 | o | | | | | | | |
| transcription factor coe1-like | 1 | | | | | | o | | |
| transcription factor coe3 | 1 | | | | | | | o | |
| transcription factor coe3-like isoform 1 | 1 | o | | | | | | | |
| transcription factor cp2 | 1 | | o | | | | | | |
| transcription factor cp2-like protein 1-like | 1 | | o | | | | | | |
| transcription factor e2f6 | 1 | | | | o | | | | |
| transcription factor e3 | 1 | | | | | | o | | |
| transcription factor etv7-like | 1 | | o | | | | | | |
| transcription factor gata-6-like | 1 | | o | | | | | | |
| transcription factor hes-1 | 1 | | | | o | | | | |
| transcription factor jun-b | 1 | | o | | | | | | |
| transcription factor maf | 1 | o | | | | | | | |
| transcription factor mitochondrial precursor | 1 | o | | | | | | | |
| transcription factor sox-1 | 1 | | | | o | | | | |
| transcription factor sox-2 | 1 | | | | | | | | o |
| transcription factor sp2-like | 1 | | | | | | o | | |
| transcription factor sp9 | 1 | | | | | | o | | |
| transcription factor tfiiib component b homolog | 1 | o | | | | | | | |
| transcription initiation factor iia subunit 1 | 1 | o | | | | | | | |
| transcription initiation factor tfiid subunit 1-like | 1 | o | | | | | | | |
| transcription initiation factor tfiid subunit 8 | 1 | o | | | | | | | |
| transcription termination rna polymerase i | 1 | o | | | | | | | |
| transcriptional enhancer factor tef-1 | 1 | o | | | | | | | |
| transcriptional intermediary factor 1 gamma | 1 | | o | | | | | | |
| transcriptional regulator kaiso | 1 | | | | | | o | | |
| transcriptional repressor nf-x1-like | 1 | | | | | o | | | |
| transcriptional repressor protein yy1 | 1 | o | | | | | | | |
| transducin -like 3 | 1 | | | | o | | | | |
| transducin-like enhancer protein 1 | 1 | | | | o | | | | |
| transferrin receptor | 1 | o | | | | | | | |

| | | | | | | | | | |
|---|---|---|---|---|---|---|---|---|---|
| transformation related protein 53 binding protein 1 | 1 | | | | | | | o | |
| transformation related protein 63 regulated like | 1 | | | | | | | o | |
| transformer 2b isoform 2 | 1 | | | o | | | | | |
| transformer-2 protein homolog beta | 1 | | o | | | | | | |
| transforming acidic coiled coil 1a | 1 | o | | | | | | | |
| transforming acidic coiled coil 1b isoform a | 1 | | | | | | o | | |
| transforming acidic coiled-coil-containing protein 1 isoform 2 | 1 | | | | | | | | o |
| transforming growth factor beta 3 | 1 | | o | | | | | | |
| transforming growth factor beta regulator 1 | 1 | | | o | | | | | |
| transforming growth factor beta regulator 4 | 1 | | | | | | | o | |
| transforming growth factor-beta-induced protein ig-h3 | 1 | o | | | | | | | |
| transforming growth factor-beta-induced protein ig-h3 precursor | 1 | | | | | | | | o |
| transgelin 3 | 1 | | | | | o | | | |
| transglutaminase 1 (k polypeptide epidermal type protein-glutamine-gamma-glutamyltransferase) | 1 | o | | | | | | | |
| transglutaminase 2 (c protein-glutamine-gamma-glutamyltransferase) | 1 | o | | | | | | | |
| transglutaminase 5 | 1 | o | | | | | | | |
| transglycosylase slt domain protein | 1 | | | | | | | | o |
| transient receptor potential cation channel subfamily m member 5 | 1 | | | | | | | o | |
| transient receptor potential cation subfamily member 2 | 1 | | | | | o | | | |
| transient receptor potential cation subfamily member 3 | 1 | | | | | | o | | |
| transient receptor potential cation subfamily member 6 | 1 | o | | | | | | | |
| transitional endoplasmic reticulum atpase | 1 | o | | | | | | | |
| transitional endoplasmic reticulum atpase ter94 | 1 | | | | | | o | | |
| transketolase-like protein 2 | 1 | | | | | | | o | |
| translation elongation factor ef-2 | 1 | | | | | | | o | |
| translation initiation factor eif-2b subunit gamma | 1 | | | | | | | | o |
| translation initiation factor if- mitochondrial-like | 1 | | | | | | | | o |
| translation initiation factor if-2 | 1 | o | | | | | | | |
| translation repressor nat1 | 1 | | | | | | o | | |
| translin | 1 | | o | | | | | | |
| translin-associated factor x | 1 | | o | | | | | | |
| translocase of outer mitochondrial membrane 34 | 1 | | o | | | | | | |
| translocated promoter region (to activated met oncogene) | 1 | | o | | | | | | |
| translocating chain-associated membrane protein 1-like 1-like | 1 | | | | o | | | | |
| translocation associated membrane protein 1 | 1 | | o | | | | | | |
| translocon-associated protein subunit alpha | 1 | | | o | | | | | |
| transmembrane 4 l six family member 5 | 1 | | o | | | | | | |
| transmembrane 4 l6 family member 1 | 1 | o | | | | | | | |
| transmembrane 4 l6 family member 4-like | 1 | | | | o | | | | |
| transmembrane 4 l6 family member 5-like | 1 | | | | o | | | | |
| transmembrane 4 superfamily member 1 | 1 | | o | | | | | | |
| transmembrane 4 superfamily member 2 | 1 | | | | | | | | o |

| | | | | | | | | | |
|---|---|---|---|---|---|---|---|---|---|
| transmembrane 6 superfamily member 2 | 1 | | | o | | | | | |
| transmembrane 7 superfamily member 2 | 1 | | o | | | | | | |
| transmembrane 7 superfamily member 3 | 1 | | o | | | | | | |
| transmembrane 9 superfamily protein member 4 | 1 | o | | | | | | | |
| transmembrane and coiled-coil domain family 1 | 1 | | | | | | o | | |
| transmembrane and coiled-coil domain family 2 | 1 | | o | | | | | | |
| transmembrane and coiled-coil domain-containing protein 1 | 1 | | | | o | | | | |
| transmembrane and coiled-coil domain-containing protein 6 | 1 | o | | | | | | | |
| transmembrane and coiled-coil domains 4 | 1 | | | | o | | | | |
| transmembrane and tetratricopeptide repeat containing 2 | 1 | | | | | | o | | |
| transmembrane and tetratricopeptide repeat containing 3 | 1 | o | | | | | | | |
| transmembrane and tetratricopeptide repeat containing 4 | 1 | | | | | o | | | |
| transmembrane and ubiquitin-like domain-containing protein 2 | 1 | | o | | | | | | |
| transmembrane anterior posterior transformation 1 | 1 | | | | | | | o | |
| transmembrane bax inhibitor motif containing 1 | 1 | | | | | | | o | |
| transmembrane bax inhibitor motif containing 6 | 1 | | | | | o | | | |
| transmembrane channel-like 6 | 1 | | | | o | | | | |
| transmembrane channel-like protein 6 | 1 | | | | | | | | o |
| transmembrane emp24 domain-containing protein 1 precursor | 1 | o | | | | | | | |
| transmembrane emp24 domain-containing protein 7 | 1 | | o | | | | | | |
| transmembrane emp24 domain-containing protein 7 precursor | 1 | | | | | o | | | |
| transmembrane emp24 domain-containing protein 9-like | 1 | | | | | | | | o |
| transmembrane emp24 protein transport domain containing 6 | 1 | | o | | | | | | |
| transmembrane phosphoinositide 3-phosphatase and tensin homolog 2 | 1 | o | | | | | | | |
| transmembrane protein 101 | 1 | o | | | | | | | |
| transmembrane protein 107 | 1 | o | | | | | | | |
| transmembrane protein 110 | 1 | | o | | | | | | |
| transmembrane protein 119 | 1 | o | | | | | | | |
| transmembrane protein 127 | 1 | o | | | | | | | |
| transmembrane protein 131-like | 1 | | | | | | o | | |
| transmembrane protein 132e | 1 | | | | | | o | | |
| transmembrane protein 135 | 1 | o | | | | | | | |
| transmembrane protein 136 | 1 | | | | | | o | | |
| transmembrane protein 138 | 1 | | | o | | | | | |
| transmembrane protein 145-like | 1 | | | | | | o | | |
| transmembrane protein 14c-like | 1 | | | | | | | | o |
| transmembrane protein 151b | 1 | o | | | | | | | |
| transmembrane protein 164 | 1 | o | | | | | | | |
| transmembrane protein 167a | 1 | | o | | | | | | |
| transmembrane protein 16a | 1 | | | | | o | | | |
| transmembrane protein 16f | 1 | o | | | | | | | |
| transmembrane protein 16k | 1 | | o | | | | | | |

| | | | | | | | | | |
|---|---|---|---|---|---|---|---|---|---|
| transmembrane protein 175 | 1 | | | | | | o | | |
| transmembrane protein 178-like | 1 | | | | | | | o | |
| transmembrane protein 180 | 1 | | | o | | | | | |
| transmembrane protein 180-like | 1 | o | | | | | | | |
| transmembrane protein 182 precursor | 1 | o | | | | | | | |
| transmembrane protein 20 | 1 | | o | | | | | | |
| transmembrane protein 200a-like | 1 | | | | | | | o | |
| transmembrane protein 204 | 1 | o | | | | | | | |
| transmembrane protein 214 | 1 | o | | | | | | | |
| transmembrane protein 218 | 1 | o | | | | | | | |
| transmembrane protein 223 | 1 | o | | | | | | | |
| transmembrane protein 223-like | 1 | | | | | | | | o |
| transmembrane protein 229b | 1 | | o | | | | | | |
| transmembrane protein 231 | 1 | o | | | | | | | |
| transmembrane protein 233-like | 1 | o | | | | | | | |
| transmembrane protein 238 | 1 | | | | o | | | | |
| transmembrane protein 25 isoform 1 | 1 | | | | | | | | o |
| transmembrane protein 42-like | 1 | | | | | | o | | |
| transmembrane protein 43 | 1 | o | | | | | | | |
| transmembrane protein 48 | 1 | o | | | | | | | |
| transmembrane protein 5 | 1 | o | | | | | | | |
| transmembrane protein 55a | 1 | | | | | | | | o |
| transmembrane protein 56 | 1 | | | | | | | o | |
| transmembrane protein 57 | 1 | | | | | | o | | |
| transmembrane protein 59-like | 1 | | | | | | o | | |
| transmembrane protein 60 | 1 | o | | | | | | | |
| transmembrane protein 62 | 1 | | | | | | o | | |
| transmembrane protein 63a | 1 | | | | | | o | | |
| transmembrane protein 70 | 1 | | o | | | | | | |
| transmembrane protein 87a-like isoform 2 | 1 | o | | | | | | | |
| transmembrane protein 97 | 1 | | o | | | | | | |
| transmembrane protein 9b precursor | 1 | | | | o | | | | |
| transmembrane protein c15orf27-like | 1 | | | | o | | | | |
| transmembrane protein c18orf45 homolog | 1 | | o | | | | | | |
| transmembrane protein c1orf70-like | 1 | | | | | | o | | |
| transmembrane protein c20orf108 | 1 | o | | | | | | | |
| transmembrane protein c3orf1 homolog | 1 | | o | | | | | | |
| transmembrane protein c5orf28-like | 1 | | | | | | | | o |
| transmembrane protein c9orf123-like | 1 | | o | | | | | | |
| transmembrane protein c9orf91 homolog | 1 | o | | | | | | | |
| transmembrane protein isoform cra_b | 1 | o | | | | | | | |
| transposable element tc3 | 1 | | | | | o | | | |
| transposable element tc3 transposase | 1 | | | | | | o | | |
| transposase [Danio rerio] | 1 | | | | | o | | | |
| Transposase [Dicentrarchus labrax] | 1 | | o | | | | | | |
| transposase [Strongylocentrotus purpuratus] | 1 | | | | | | | o | |
| transposon-derived buster3 transposase-like | 1 | | | o | | | | | |
| trf1-interacting ankyrin-related adp-ribose polymerase | 1 | | | | | | o | | |
| tribbles homolog 2 | 1 | o | | | | | | | |
| tributyltin-binding protein type 1 | 1 | | o | | | | | | |
| trichorhinophalangeal syndrome i | 1 | o | | | | | | | |
| trifunctional enzyme subunit mitochondrial | 1 | | | | | | | | o |
| trimethylguanosine synthase | 1 | o | | | | | | | |
| trimethyllysine epsilon | 1 | o | | | | | | | |
| trinucleotide repeat containing 4 isoform cra_a | 1 | | | | | | | | o |
| triose phosphate isomerase | 1 | | | | o | | | | |
| tripartite motif protein 16 | 1 | | o | | | | | | |
| tripartite motif-containing 32 | 1 | o | | | | | | | |
| tripartite motif-containing 33 | 1 | | | o | | | | | |

| | | | | | | | | | |
|---|---|---|---|---|---|---|---|---|---|
| tripartite motif-containing 36 | 1 | | | | | | o | | |
| tripartite motif-containing 46 | 1 | | | | | | o | | |
| tripartite motif-containing 59 | 1 | o | | | | | | | |
| tripartite motif-containing 8 | 1 | o | | | | | | | |
| tripartite motif-containing protein 16 isoform 1 | 1 | | o | | | | | | |
| tripartite motif-containing protein 2 isoform 1 | 1 | | | | | | o | | |
| tripartite motif-containing protein 35-like | 1 | | o | | | | | | |
| tripartite motif-containing protein 39-like | 1 | | | | o | | | | |
| tripartite motif-containing protein 46-like | 1 | | | | | | | | o |
| tripartite motif-containing protein 72 | 1 | | o | | | | | | |
| tripeptidyl peptidase ii | 1 | o | | | | | | | |
| tripeptidyl peptidase ii isoform 1 | 1 | o | | | | | | | |
| tripeptidyl-peptidase 2 | 1 | | | | | | | | o |
| triple helix repeat-containing collagen | 1 | o | | | | | | | |
| trna (cytosine-5-)-methyltransferase | 1 | | | | | | | | o |
| trna (uracil-5-)-methyltransferase homolog a | 1 | | | | o | | | | |
| trna 2 -phosphotransferase 1 | 1 | o | | | | | | | |
| trna aspartic acid methyltransferase 1 | 1 | o | | | | | | | |
| trna guanosine-2 -o-methyltransferase trm11 homolog | 1 | o | | | | | | | |
| trna selenocysteine associated protein 1 | 1 | | | o | | | | | |
| trna selenocysteine-associated protein 1 secp43 | 1 | | | | o | | | | |
| trna splicing endonuclease 15 homolog ( cerevisiae) | 1 | o | | | | | | | |
| trna-dihydrouridine synthase 1-like | 1 | | | | o | | | | |
| trna-dihydrouridine synthase 2-like | 1 | | | | | o | | | |
| trna-specific adenosine deaminase 2 | 1 | o | | | | | | | |
| trophoblast glyco | 1 | | | | o | | | | |
| trophozoite antigen | 1 | | | | o | | | | |
| tropomodulin 1 | 1 | o | | | | | | | |
| tropomodulin 3 | 1 | | | | | | o | | |
| tropomodulin-2 isoform 2 | 1 | o | | | | | | | |
| tropomyosin 3 | 1 | o | | | | | | | |
| tropomyosin 3 isoform 2 isoform 2 | 1 | o | | | | | | | |
| tropomyosin alpha-1 chain | 1 | | | o | | | | | |
| tropomyosin alpha-4 chain isoform 1 | 1 | o | | | | | | | |
| troponin c | 1 | o | | | | | | | |
| troponin c-akin-1 protein | 1 | | | o | | | | | |
| troponin cardiac | 1 | o | | | | | | | |
| troponin i | 1 | o | | | | | | | |
| troponin i fast skeletal muscle | 1 | o | | | | | | | |
| truncated cell wall surface anchor family protein (fragment 2) | 1 | o | | | | | | | |
| truncated herc6 | 1 | o | | | | | | | |
| trypsin | 1 | | o | | | | | | |
| trypsinogen 1 | 1 | | o | | | | | | |
| trypsinogen 2 precursor | 1 | | o | | | | | | |
| trypsinogen i | 1 | | | | | | o | | |
| trypsinogen y | 1 | | | | o | | | | |
| tryptase-2 precursor | 1 | o | | | | | | | |
| tryptophan hydroxylase | 1 | | | | | | o | | |
| tryptophan-rich antigen 74 | 1 | | | | | | | | o |
| tsc22 domain family protein 1 isoform 1 | 1 | o | | | | | | | |
| tshz1 protein | 1 | | | | o | | | | |
| tso1-like transcription factor | 1 | | | o | | | | | |
| tspan7 protein | 1 | | | | | | | | o |
| tsukushin-like isoform 1 | 1 | | o | | | | | | |
| ttk protein kinase | 1 | | o | | | | | | |

| | | | | | | | | | |
|---|---|---|---|---|---|---|---|---|---|
| tuba1 protein | 1 | | | | | o | | | |
| tubb2a protein | 1 | | | | | | | | o |
| tubby like protein 4 | 1 | | | | | | o | | |
| tubby protein homolog | 1 | | | | | | o | | |
| tubby-related protein 4-like | 1 | | | | | | o | | |
| tuberin isoform 1 | 1 | | | | | | o | | |
| tuberin-like protein 1 | 1 | | | o | | | | | |
| tuberous sclerosis 1 | 1 | | | | | | o | | |
| tubulin a | 1 | | | | | | | | o |
| tubulin alpha 2 | 1 | | | | o | | | | |
| tubulin alpha-1a chain- partial | 1 | | | | o | | | | |
| tubulin alpha-1c chain isoform 1 | 1 | | | | | | | | o |
| tubulin alpha-8 chain- partial | 1 | | | | | | | | o |
| tubulin beta chain | 1 | | | | | | | o | |
| tubulin beta-2a chain-like | 1 | | | | | | | | o |
| tubulin beta-4 chain-like | 1 | | | o | | | | | |
| tubulin delta chain-like | 1 | | | | | | | | o |
| tubulin folding cofactor e-like | 1 | o | | | | | | | |
| tubulin gamma-1 chain-like | 1 | | | | | | | | o |
| tubulin polymerization-promoting protein | 1 | o | | | | | | | |
| tubulin polymerization-promoting protein family member 3 | 1 | | | | | | | | o |
| tubulin tyrosine ligase | 1 | o | | | | | | | |
| tubulin-folding cofactor b | 1 | | | | | | | | o |
| tubulointerstitial nephritis antigen-like 1 | 1 | o | | | | | | | |
| tudor domain containing 7 | 1 | | o | | | | | | |
| tudor domain-containing protein 1 | 1 | o | | | | | | | |
| tudor domain-containing protein 3 | 1 | o | | | | | | | |
| tuftelin-like | 1 | | | | | | | | o |
| tumor necrosis alpha-induced protein 2 | 1 | o | | | | | | | |
| tumor necrosis alpha-induced protein 6 | 1 | o | | | | | | | |
| tumor necrosis alpha-induced protein 8-like 3 | 1 | | | o | | | | | |
| tumor necrosis factor ligand superfamily member 13b | 1 | | | o | | | | | |
| tumor necrosis factor member 15 | 1 | | | o | | | | | |
| tumor necrosis factor receptor member 11b | 1 | o | | | | | | | |
| tumor necrosis factor receptor member 1b | 1 | o | | | | | | | |
| tumor necrosis factor receptor superfamily member 5 precursor | 1 | o | | | | | | | |
| tumor necrosis factor receptor superfamily member 6 | 1 | o | | | | | | | |
| tumor necrosis factor receptor superfamily member 6 precursor | 1 | | | | | | | | o |
| tumor protein d52-like 1 | 1 | | o | | | | | | |
| tumor protein p53 binding protein 1 | 1 | | | | | | o | | |
| tumor protein p53 inducible protein 5 | 1 | | | | | | o | | |
| tumor protein p53-inducible nuclear protein 2 | 1 | | o | | | | | | |
| tumor protein p53-inducible nuclear protein 2-like | 1 | o | | | | | | | |
| tumor protein p53-inducible protein 11 | 1 | o | | | | | | | |
| tumor protein p63 regulated 1-like | 1 | | | | | o | | | |
| tumor protein p63-regulated gene 1-like protein | 1 | o | | | | | | | |
| tumor suppressor candidate 4 | 1 | o | | | | | | | |
| tumor suppressor p53-binding protein 1 | 1 | | | | | | | | o |
| tumor suppressor p53-binding protein 1-like | 1 | | | | | | | | o |
| tweety homolog 2 | 1 | | | | | | o | | |
| tweety homolog 3 | 1 | | | | | | o | | |
| twist homolog 2 | 1 | o | | | | | | | |
| twisted gastrulation | 1 | o | | | | | | | |

| | | | | | | | | | |
|---|---|---|---|---|---|---|---|---|---|
| twisted gastrulation homolog 1 | 1 | o | | | | | | | |
| twisted gastrulation protein homolog 1-like | 1 | | | | o | | | | |
| type alpha 3 (goodpasture antigen) binding isoform cra_a | 1 | | o | | | | | | |
| type alpha 3-like isoform 4 | 1 | o | | | | | | | |
| -type and c3hc4-type zinc finger-containing protein 1 | 1 | | | | o | | | | |
| type i inositol- -bisphosphate 4-phosphatase-like | 1 | | | | o | | | | |
| type i iodothyronine deiodinase | 1 | | | | | o | | | |
| type i keratin e7 | 1 | | o | | | | | | |
| type i transmembrane receptor | 1 | | | | | | | o | |
| type ii cax cation proton partial | 1 | o | | | | | | | |
| type ii inositol- -trisphosphate 5-phosphatase-like | 1 | o | | | | | | | |
| type iia procollagen | 1 | o | | | | | | | |
| type iii iodothyronine deiodinase | 1 | | o | | | | | | |
| type xxviii | 1 | o | | | | | | | |
| type-1 angiotensin ii receptor-associated | 1 | | | | | o | | | |
| type-2 ice-structuring protein precursor | 1 | | | | | | | o | |
| type-4 ice-structuring protein precursor | 1 | | | | | | | o | |
| tyrosine 3-monooxygenase tryptophan 5-monooxygenase activation beta polypeptide | 1 | | | | | o | | | |
| tyrosine 3-monooxygenase tryptophan 5-monooxygenase activation theta polypeptide a | 1 | | | | | | | | o |
| tyrosine 3-monooxygenase tryptophan 5-monooxygenase activation zeta polypeptide | 1 | | | | | | o | | |
| tyrosine protein kinase | 1 | | | | | | o | | |
| tyrosine recombinase- partial | 1 | o | | | | | | | |
| tyrosine-protein kinase abl1-like | 1 | o | | | | | | | |
| tyrosine-protein kinase csk-like | 1 | | | | | | | | o |
| tyrosine-protein kinase frk-like | 1 | | | | o | | | | |
| tyrosine-protein kinase fyn-like isoform 2 | 1 | | | | | | | o | |
| tyrosine-protein kinase jak2 | 1 | | | | | o | | | |
| tyrosine-protein kinase lyn | 1 | | | | | | | | o |
| tyrosine-protein kinase syk | 1 | | | | | | | | o |
| tyrosine-protein phosphatase non-receptor type 13 | 1 | o | | | | | | | |
| tyrosine-protein phosphatase non-receptor type 13 isoform 1 | 1 | | | | o | | | | |
| tyrosine-protein phosphatase non-receptor type 13 isoform 2 | 1 | | | | o | | | | |
| tyrosine-protein phosphatase non-receptor type 13 isoform 3 | 1 | | | | | | | | o |
| tyrosine-protein phosphatase non-receptor type 21 | 1 | o | | | | | | | |
| tyrosine-protein phosphatase non-receptor type 6 | 1 | o | | | | | | | |
| tyrosyl-trna cytoplasmic | 1 | | | | | | | | o |
| u2 small nuclear rna auxiliary factor 1-like 2 | 1 | o | | | | | | | |
| u2 small nuclear rna auxiliary factor 2 isoform b | 1 | o | | | | | | | |
| u3 small nucleolar homolog a | 1 | o | | | | | | | |
| u3 small nucleolar rna-associated protein 15 homolog | 1 | o | | | | | | | |
| u4 tri-snrnp-associated protein 1-like | 1 | | | o | | | | | |
| u4 u6 small nuclear ribonucleoprotein prp3 | 1 | o | | | | | | | |
| u88_hhv6u ame: full=uncharacterized protein u88 | 1 | | | | o | | | | |

| | | | | | | | | | |
|---|---|---|---|---|---|---|---|---|---|
| ubc protein | 1 | | | | | | | | o |
| ube2g1 protein | 1 | | o | | | | | | |
| ubiquinol-cytochrome c reductase complex chaperone | 1 | o | | | | | | | |
| ubiquinol-cytochrome c reductase complex chaperone cbp3 homolog | 1 | | | | | | | | o |
| ubiquinol-cytochrome c reductase iron-sulfur subunit | 1 | | | | | o | | | |
| ubiquinone biosynthesis protein coq4 mitochondrial | 1 | | | | | | o | | |
| ubiquinone menaquinone biosynthesis methyltransferase | 1 | | o | | | | | | |
| ubiquitin associated protein 2 | 1 | o | | | | | | | |
| ubiquitin associated protein 2-like | 1 | o | | | | | | | |
| ubiquitin b | 1 | | | | | | | | o |
| ubiquitin carboxyl-terminal | 1 | | | | | | | o | |
| ubiquitin carboxyl-terminal hydrolase | 1 | o | | | | | | | |
| ubiquitin carboxyl-terminal hydrolase 12 | 1 | | o | | | | | | |
| ubiquitin carboxyl-terminal hydrolase 15-like isoform 1 | 1 | | | | | | o | | |
| ubiquitin carboxyl-terminal hydrolase 19 isoform 3 | 1 | o | | | | | | | |
| ubiquitin carboxyl-terminal hydrolase 25 | 1 | | | | | | | | o |
| ubiquitin carboxyl-terminal hydrolase 3 | 1 | | | | | | | | o |
| ubiquitin carboxyl-terminal hydrolase 30 | 1 | o | | | | | | | |
| ubiquitin carboxyl-terminal hydrolase 31 | 1 | | | | | | o | | |
| ubiquitin carboxyl-terminal hydrolase 33 | 1 | | | | | | | o | |
| ubiquitin carboxyl-terminal hydrolase 34 | 1 | | | | o | | | | |
| ubiquitin carboxyl-terminal hydrolase 36 | 1 | | | | o | | | | |
| ubiquitin carboxyl-terminal hydrolase 47-like | 1 | | | | | | o | | |
| ubiquitin carboxyl-terminal hydrolase 4-like | 1 | | o | | | | | | |
| ubiquitin carboxyl-terminal hydrolase 8 | 1 | | | | | | o | | |
| ubiquitin carboxyl-terminal hydrolase bap1 | 1 | o | | | | | | | |
| ubiquitin carboxyl-terminal hydrolase cyld-like | 1 | | o | | | | | | |
| ubiquitin carboxyl-terminal hydrolase isozyme l5 | 1 | | | o | | | | | |
| ubiquitin conjugating enzyme protein 13 | 1 | | | | | | o | | |
| ubiquitin conjugation factor e4 | 1 | | o | | | | | | |
| ubiquitin conjugation factor e4 a | 1 | | | | o | | | | |
| ubiquitin conjugation factor e4 b-like | 1 | | | | o | | | | |
| ubiquitin domain-containing protein | 1 | o | | | | | | | |
| ubiquitin domain-containing protein 1-like | 1 | | | | | | | | o |
| ubiquitin family protein | 1 | | | o | | | | | |
| ubiquitin processing protease | 1 | o | | | | | | | |
| ubiquitin protein ligase e3c | 1 | o | | | | | | | |
| ubiquitin specific peptidase 10 | 1 | o | | | | | | | |
| ubiquitin specific peptidase 13 (isopeptidase t-3) | 1 | o | | | | | | | |
| ubiquitin specific peptidase 14 (trna-guanine transglycosylase) | 1 | | o | | | | | | |
| ubiquitin specific peptidase 16 | 1 | o | | | | | | | |
| ubiquitin specific peptidase 19 | 1 | o | | | | | | | |
| ubiquitin specific peptidase 2 | 1 | o | | | | | | | |
| ubiquitin specific peptidase 31 | 1 | | | | | | o | | |
| ubiquitin specific peptidase 33 | 1 | o | | | | | | | |
| ubiquitin specific peptidase 45 | 1 | o | | | | | | | |
| ubiquitin specific peptidase 5 (isopeptidase t) | 1 | | o | | | | | | |
| ubiquitin specific protease 18 | 1 | | | o | | | | | |
| ubiquitin specific protease 25 | 1 | | | | | | o | | |

| | | | | | | | | | |
|---|---|---|---|---|---|---|---|---|---|
| ubiquitin specific protease 39 | 1 | | | | | | | o | |
| ubiquitin specific protease x-linked isoform 2 | 1 | | | | | | | o | |
| ubiquitin thioesterase otu1 | 1 | o | | | | | | | |
| ubiquitin-60s ribosomal protein l40 | 1 | | o | | | | | | |
| ubiquitin-conjugating enzyme e2 | 1 | o | | | | | | | |
| ubiquitin-conjugating enzyme e2 a-like | 1 | | | | o | | | | |
| ubiquitin-conjugating enzyme e2 d1 | 1 | | | | o | | | | |
| ubiquitin-conjugating enzyme e2 q2 | 1 | | | | | | | | o |
| ubiquitin-conjugating enzyme e2 q2-like | 1 | | o | | | | | | |
| ubiquitin-conjugating enzyme e2c-binding protein | 1 | | | | o | | | | |
| ubiquitin-conjugating enzyme e2d 3 (ubc4 5 yeast) isoform cra_a | 1 | | o | | | | | | |
| ubiquitin-conjugating enzyme e2g 2 | 1 | | | | | | | o | |
| ubiquitin-conjugating enzyme e2m | 1 | o | | | | | | | |
| ubiquitin-conjugating enzyme e2q 2 | 1 | | o | | | | | | |
| ubiquitin-conjugating enzyme e2q family member 1 | 1 | o | | | | | | | |
| ubiquitin-conjugating enzyme e2s | 1 | | o | | | | | | |
| ubiquitin-conjugating enzyme family protein | 1 | o | | | | | | | |
| ubiquitin-like 4a | 1 | | | | | | o | | |
| ubiquitin-like modifier activating enzyme 1 | 1 | | | o | | | | | |
| ubiquitin-like modifier activating enzyme 6 | 1 | o | | | | | | | |
| ubiquitin-like protein atg12 | 1 | | | | | | o | | |
| ubiquitin-like protein atg12-like | 1 | | | | o | | | | |
| ubiquitin-protein ligase e3c | 1 | | o | | | | | | |
| ubiquitin-ribosomal protein fusion s27a | 1 | | o | | | | | | |
| ubiquitin-specific peptidase 46 (c19 family) | 1 | o | | | | | | | |
| u-box domain containing protein | 1 | | | | o | | | | |
| ubx domain protein 2a | 1 | | | | | | o | | |
| ubx domain-containing protein 1 | 1 | | o | | | | | | |
| ubx domain-containing protein 2a | 1 | o | | | | | | | |
| ubx domain-containing protein 2a-like | 1 | | | | | | o | | |
| ubxn6 protein | 1 | o | | | | | | | |
| uchl5 interacting protein | 1 | o | | | | | | | |
| udp- c:beta- -n-acetylgalactosaminyltransferase 1 | 1 | o | | | | | | | |
| udp- c:betagal beta- -n-acetylglucosaminyltransferase 7 | 1 | | | | | | | o | |
| udp glucuronosyltransferase 2b10-like isoform 2 | 1 | | | o | | | | | |
| udp glucuronosyltransferase 5 polypeptide f1 | 1 | | o | | | | | | |
| udp glucuronosyltransferase 5 polypeptide g2 precursor | 1 | | | | o | | | | |
| udp-gal:beta c beta - polypeptide 2 | 1 | o | | | | | | | |
| udp-gal:beta c beta - polypeptide 6 | 1 | o | | | | | | | |
| udp-gal:beta c beta -galactosyltransferase 2-like | 1 | o | | | | | | | |
| udp-glucuronate decarboxylase 1 | 1 | o | | | | | | | |
| udp-glucuronosyltransferase | 1 | | o | | | | | | |
| udp-glucuronosyltransferase 2a1-like isoform 2 | 1 | | o | | | | | | |
| udp-glucuronosyltransferase 2b22 | 1 | o | | | | | | | |
| udp-n-acetyl-alpha-d-galactosamine:polypeptide n-acetylgalactosaminyltransferase-like 1 | 1 | | | | | | | | o |
| udp-n-acetyl-alpha-d-galactosamine:polypeptide n-acetylgalactosaminyltransferase-like 2 | 1 | o | | | | | | | |

| | | | | | | | | | |
|---|---|---|---|---|---|---|---|---|---|
| udp-n-acetylglucosamine transferase subunit alg14 homolog | 1 | | | o | | | | | |
| udp-n-acetylglucosamine transporter | 1 | | o | | | | | | |
| udp-n-acetylglucosamine--peptide n-acetylglucosaminyltransferase 110 kda subunit-like isoform 2 | 1 | | | | | | o | | |
| ufm1-conjugating enzyme 1 | 1 | | o | | | | | | |
| ugt1ab protein | 1 | | o | | | | | | |
| ump-cmp kinase mitochondrial-like | 1 | o | | | | | | | |
| unc-13 homolog a ( elegans) | 1 | | | | | | o | | |
| unc-45 homolog a ( elegans) | 1 | | o | | | | | | |
| unc-5 homolog c ( elegans) | 1 | | | | | | o | | |
| unc-51-like kinase 1 | 1 | | o | | | | | | |
| unc-84 homolog a ( elegans) | 1 | | | | | | o | | |
| unc93-like protein mfsd11-like | 1 | | o | | | | | | |
| uncharacterized | 1 | | | | o | | | | |
| uncharacterized family 31 glucosidase kiaa1161-like | 1 | | | | o | | | | |
| uncharacterized oxidoreductase -like | 1 | | o | | | | | | |
| uncharacterized protein c10orf58-like | 1 | | | | | | | o | |
| uncharacterized protein c10orf78-like | 1 | o | | | | | | | |
| uncharacterized protein c10orf88 homolog | 1 | | | | | | | | o |
| uncharacterized protein c11orf46 homolog | 1 | | | | | | | | o |
| uncharacterized protein c11orf57-like isoform 2 | 1 | | o | | | | | | |
| uncharacterized protein c11orf65-like | 1 | o | | | | | | | |
| uncharacterized protein c12orf26-like | 1 | | | | o | | | | |
| uncharacterized protein c12orf4 homolog | 1 | o | | | | | | | |
| uncharacterized protein c12orf43 homolog | 1 | | | | o | | | | |
| uncharacterized protein c14orf138-like | 1 | o | | | | | | | |
| uncharacterized protein c14orf179 homolog | 1 | | | | | | o | | |
| uncharacterized protein c14orf28 homolog | 1 | | o | | | | | | |
| uncharacterized protein c15orf57 homolog | 1 | | | | | | | | o |
| uncharacterized protein c15orf61-like | 1 | | | o | | | | | |
| uncharacterized protein c16orf45 homolog | 1 | | | | | o | | | |
| uncharacterized protein c16orf68-like | 1 | o | | | | | | | |
| uncharacterized protein c17orf89-like | 1 | o | | | | | | | |
| uncharacterized protein c18orf25-like | 1 | | | | | | o | | |
| uncharacterized protein c18orf25-like isoform 2 | 1 | o | | | | | | | |
| uncharacterized protein c19orf29-like | 1 | o | | | | | | | |
| uncharacterized protein c19orf39 homolog | 1 | | | | | | | | o |
| uncharacterized protein c1orf172 homolog | 1 | | | | o | | | | |
| uncharacterized protein c1orf210-like isoform 1 | 1 | | | o | | | | | |
| uncharacterized protein c1orf31 homolog | 1 | | | | o | | | | |
| uncharacterized protein c20orf117-like | 1 | o | | | | | | | |
| uncharacterized protein c20orf160-like | 1 | o | | | | | | | |
| uncharacterized protein c20orf177-like | 1 | | | | | o | | | |
| uncharacterized protein c20orf72-like | 1 | | | | o | | | | |
| uncharacterized protein c21orf59-like | 1 | | | | o | | | | |
| uncharacterized protein c2orf64 homolog | 1 | | o | | | | | | |
| uncharacterized protein c2orf90- partial | 1 | o | | | | | | | |
| uncharacterized protein c3orf18-like | 1 | | | | | | | | o |
| uncharacterized protein c3orf26 homolog | 1 | | | | o | | | | |
| uncharacterized protein c4orf29 homolog | 1 | | o | | | | | | |
| uncharacterized protein c4orf44 homolog | 1 | | | | | | | | o |
| uncharacterized protein c4orf52-like | 1 | | | | | o | | | |
| uncharacterized protein c5orf34-like | 1 | o | | | | | | | |
| uncharacterized protein c5orf4-like | 1 | | | | | o | | | |

| | | | | | | | | | |
|---|---|---|---|---|---|---|---|---|---|
| uncharacterized protein c7orf30-like | 1 | | | | o | | | | |
| uncharacterized protein c7orf57 homolog isoform 2 | 1 | | | | | | o | | |
| uncharacterized protein c8orf4 homolog | 1 | | | | o | | | | |
| uncharacterized protein c8orf59 homolog | 1 | | | o | | | | | |
| uncharacterized protein c8orf76-like | 1 | | o | | | | | | |
| uncharacterized protein c9orf72-like | 1 | | | o | | | | | |
| uncharacterized protein c9orf78-like | 1 | | | | | | | | o |
| uncharacterized protein cxorf57 homolog | 1 | | | | o | | | | |
| uncharacterized protein cxorf57-like | 1 | | o | | | | | | |
| uncharacterized protein kiaa0090 homolog | 1 | | | | | | | | o |
| uncharacterized protein kiaa0146-like | 1 | | | | o | | | | |
| uncharacterized protein kiaa0408-like | 1 | | | | | | o | | |
| uncharacterized protein kiaa0528-like | 1 | | o | | | | | | |
| uncharacterized protein kiaa0564-like | 1 | | | | | | o | | |
| uncharacterized protein kiaa0895-like | 1 | o | | | | | | | |
| uncharacterized protein kiaa0895-like isoform 1 | 1 | | | | | | o | | |
| uncharacterized protein kiaa1310-like | 1 | | | | o | | | | |
| uncharacterized protein kiaa1539 homolog | 1 | | | | | | o | | |
| uncharacterized protein kiaa1797-like | 1 | | | | o | | | | |
| uncharacterized protein kiaa2026-like | 1 | | o | | | | | | |
| uncharacterized protein loc100000381 precursor | 1 | | | | o | | | | |
| uncharacterized protein loc100034574 | 1 | | | | o | | | | |
| uncharacterized protein loc100295268 | 1 | | | | o | | | | |
| uncharacterized protein loc100507341 | 1 | | | | | | | | o |
| uncharacterized protein loc100802441 | 1 | | | | o | | | | |
| uncharacterized protein loc285141-like | 1 | | | | | | o | | |
| uncharacterized protein loc336578 | 1 | | | | | | | | o |
| uncharacterized protein loc388588 homolog isoform 1 | 1 | | | o | | | | | |
| uncharacterized protein loc541387 | 1 | | | | o | | | | |
| uncharacterized protein loc768125 | 1 | | | | o | | | | |
| uncharacterized protein loc799220 | 1 | | | | o | | | | |
| uncoordinated family member (unc-89) | 1 | o | | | | | | | |
| unique cartilage matrix-associated protein | 1 | o | | | | | | | |
| unkempt homolog-like | 1 | o | | | | | | | |
| unknow protein | 1 | | | o | | | | | |
| unknown protein [Clostridium botulinum NCTC 2916] | 1 | | | | | | | o | |
| unknown protein [Siniperca chuatsi] | 1 | o | | | | | | o | |
| unknown protein [Streptococcus suis 98HAH33] | 1 | | | | | | | o | |
| unnamed protein product [Oikopleura dioica] | 1 | | | | o | | | | |
| unnamed protein product [Ostreococcus tauri] | 1 | | | | | | o | | |
| unnamed protein product [Trypanosoma congolense IL3000] | 1 | | | | o | | | | |
| unnamed protein product [Xenopus laevis] | 1 | | | o | | | | | |
| upf0452 protein c7orf41 homolog | 1 | | | | | | o | | |
| upf0454 protein c12orf49-like isoform 1 | 1 | o | | | | | | | |
| upf0454 protein c12orf49-like isoform 2 | 1 | o | | | | | | | |
| upf0465 protein c5orf33-like | 1 | | o | | | | | | |
| upf0471 protein c1orf63 homolog | 1 | | | | | | | | o |
| upf0480 protein c15orf24 homolog precursor | 1 | | | | o | | | | |
| upf0480 protein c15orf24-like | 1 | | | | o | | | | |
| upf0489 protein c5orf22-like | 1 | o | | | | | | | |
| upf0498 protein kiaa1191-like | 1 | | | | | | | o | |
| upf0527 membrane protein | 1 | | | | o | | | | |

| | | | | | | | | | |
|---|---|---|---|---|---|---|---|---|---|
| upf0534 protein c4orf43 homolog | 1 | | | | o | | | | |
| upf0538 protein c2orf76 homolog | 1 | | | | | | | | o |
| upf0538 protein c2orf76 homolog isoform 1 | 1 | | | | | | | | o |
| upf0542 protein c5orf43 homolog | 1 | | | | o | | | | |
| upf0547 protein c16orf87 homolog | 1 | | | | o | | | | |
| upf0549 protein c20orf43 homolog | 1 | | | | | | o | | |
| upf0550 protein c7orf28-like | 1 | | o | | | | | | |
| upf0551 protein mitochondrial-like | 1 | | o | | | | | | |
| upf0563 protein c17orf95 homolog | 1 | | | | o | | | | |
| upf0577 protein kiaa1324-like isoform 2 | 1 | | | | | | | | o |
| upf0598 protein c8orf82-like | 1 | o | | | | | | | |
| upf0606 protein c11orf41-like | 1 | | | | | | o | | |
| upf0628 protein c10orf96 homolog | 1 | | | | o | | | | |
| upf0638 protein b-like | 1 | | | o | | | | | |
| upf0640 protein c3orf78 homolog | 1 | | o | | | | | | |
| upf0663 transmembrane protein c17orf28 homolog | 1 | | | | o | | | | |
| upf0668 protein c10orf76 homolog | 1 | | | | | | | o | |
| upf0668 protein c10orf76-like | 1 | | o | | | | | | |
| upf0669 protein c6orf120 homolog precursor | 1 | | | | o | | | | |
| upf0670 protein c8orf55 homolog | 1 | | | | | | | | o |
| upf0683 protein c7orf47 homolog | 1 | | | | o | | | | |
| upf0690 protein c1orf52 homolog | 1 | | | | o | | | | |
| upf0692 protein c19orf54 homolog | 1 | o | | | | | | | |
| upf0705 protein c11orf49 homolog | 1 | o | | | | | | | |
| upf0712 protein c7orf64-like | 1 | | | | o | | | | |
| upf0723 protein c11orf83-like | 1 | | | | o | | | | |
| upf0764 protein c16orf89 homolog | 1 | | | | | | | | o |
| upf2 regulator of nonsense transcripts homolog | 1 | | o | | | | | | |
| upf3 regulator of nonsense transcripts homolog b | 1 | | o | | | | | | |
| upstream transcription factor 1 | 1 | o | | | | | | | |
| upstream transcription factor c-fos interacting | 1 | o | | | | | | | |
| urea erythrocyte | 1 | | | | o | | | | |
| uridine 5 -monophosphate synthase | 1 | | o | | | | | | |
| uridine diphosphate glucose pyrophosphatase | 1 | | | | | | | o | |
| urokinase-type plasminogen activator | 1 | o | | | | | | | |
| usher syndrome 2a (autosomal mild) | 1 | | | | | | o | | |
| usp22 protein | 1 | | o | | | | | | |
| usp4 protein | 1 | o | | | | | | | |
| usp43 protein | 1 | | | | | | | o | |
| utp--glucose-1-phosphate uridylyltransferase | 1 | | | | o | | | | |
| uv excision repair protein rad23 homolog a | 1 | o | | | | | | | |
| uv radiation resistance-associated gene protein | 1 | o | | | | | | | |
| uveal autoantigen with coiled-coil domains and ankyrin repeats | 1 | o | | | | | | | |
| vaccinia related kinase 3 | 1 | o | | | | | | | |
| vacuolar atp synthase subunit f | 1 | | | | | o | | | |
| vacuolar atpase assembly integral membrane protein vma21-like | 1 | | | | | | | | o |
| vacuolar protein sorting 13 homolog a | 1 | o | | | | | | | |
| vacuolar protein sorting 13 homolog b isoform 2 | 1 | o | | | | | | | |
| vacuolar protein sorting 13 homolog d ( cerevisiae) | 1 | o | | | | | | | |
| vacuolar protein sorting 13a-like | 1 | | | | o | | | | |
| vacuolar protein sorting 13c protein | 1 | | | | | | | o | |
| vacuolar protein sorting 13d | 1 | | | o | | | | | |
| vacuolar protein sorting 36 | 1 | o | | | | | | | |

| | | | | | | | | | |
|---|---|---|---|---|---|---|---|---|---|
| vacuolar protein sorting 39 | 1 | o | | | | | | | |
| vacuolar protein sorting-associated protein 13b isoform 1 | 1 | | | | | | o | | |
| vacuolar protein sorting-associated protein 13d-like | 1 | | | | | | o | | |
| vacuolar protein sorting-associated protein 16 homolog | 1 | | | | | | | | o |
| vacuolar protein sorting-associated protein 16 homolog isoform 1 | 1 | o | | | | | | | |
| vacuolar protein sorting-associated protein 33b | 1 | o | | | | | | | |
| vacuolar protein sorting-associated protein 45 | 1 | | | | | | | | o |
| vacuolar protein sorting-associated protein 45 isoform 2 | 1 | | | | | | o | | |
| vacuolar protein sorting-associated protein 4a-like | 1 | | o | | | | | | |
| vacuolar protein sorting-associated protein 53 homolog | 1 | | | | | | | o | |
| vacuolar protein sorting-associated protein vta1 homolog | 1 | | | | | | o | | |
| vacuolar proton atpases | 1 | | | | | | | | o |
| vacuolar proton pump subunit g 1 | 1 | | | | | | | | o |
| v-akt murine thymoma viral oncogene homolog 1 | 1 | o | | | | | | | |
| valacyclovir hydrolase | 1 | | | o | | | | | |
| valacyclovir hydrolase precursor | 1 | o | | | | | | | |
| vapb protein | 1 | | o | | | | | | |
| vasa | 1 | | | | o | | | | |
| vascular cell adhesion molecule 1 | 1 | o | | | | | | | |
| vascular endothelial growth factor d | 1 | | | | | | | | o |
| vascular endothelial growth factor precursor | 1 | | o | | | | | | |
| vascular endothelial zinc finger 1 | 1 | o | | | | | | | |
| vasoactive intestinal peptide receptor 1 | 1 | o | | | | | | | |
| vasodilator-stimulated phosphoprotein | 1 | o | | | | | | | |
| vasotocin type 2 receptor | 1 | | o | | | | | | |
| v-atpase subunit a | 1 | o | | | | | | | |
| vcp protein | 1 | o | | | | | | | |
| veinlet-like 2 | 1 | o | | | | | | | |
| venom dipeptidylpeptidase iv | 1 | o | | | | | | | |
| venom protein-2 | 1 | o | | | | | | | |
| v-erb-b2 erythroblastic leukemia viral oncogene homolog 3b | 1 | | | | | | | o | |
| versican core | 1 | | | | | | o | | |
| very low density lipoprotein receptor | 1 | | | | | | o | | |
| vesicle amine transport protein 1 | 1 | o | | | | | | | |
| vesicle transport protein sec20-like | 1 | | o | | | | | | |
| vesicle transport protein sft2a | 1 | | o | | | | | | |
| vesicle-associated membrane protein 5 | 1 | | | o | | | | | |
| vesicle-associated membrane protein-associated protein b | 1 | | | | o | | | | |
| vesicle-associated membrane protein-associated protein b c | 1 | | | | | | | | o |
| vesicle-associated membrane protein-associated protein b c variant 3 | 1 | o | | | | | | | |
| vesicle-trafficking protein sec22a | 1 | | | o | | | | | |
| vesicle-trafficking protein sec22b-like | 1 | | | | o | | | | |
| vesicular inhibitory amino acid transporter | 1 | | | | | | | o | |
| vestigial like 4 | 1 | | | | | | | o | |
| vg1 protein | 1 | | | | o | | | | |

| | | | | | | | | | |
|---|---|---|---|---|---|---|---|---|---|
| vg56_ichva ame: full=uncharacterized protein orf56 | 1 | | | | | | o | | |
| v-ha-ras harvey rat sarcoma viral oncogene-like | 1 | | | o | | | | | |
| vip peptides-like | 1 | | | | o | | | | |
| vip36-like protein | 1 | | | o | | | | | |
| viral a-type inclusion | 1 | o | | | | | | | |
| viral a-type inclusion protein repeat containing protein | 1 | | | | o | | | | |
| viral complement control protein | 1 | | | | o | | | | |
| viral protein tpx | 1 | | | | | | o | | |
| vitamin d receptor | 1 | | | o | | | | | |
| vitamin k epoxide reductase complex subunit 1 | 1 | | o | | | | | | |
| vitamin k epoxide reductase complex subunit 1-like protein 1 | 1 | | | | o | | | | |
| vitamin k-dependent gamma-carboxylase-like | 1 | | | | o | | | | |
| vitamin k-dependent protein c | 1 | | o | | | | | | |
| vitamin k-dependent protein z-like | 1 | | | | | | | | o |
| vitelline envelope protein beta | 1 | | | o | | | | | |
| vitelline membrane outer layer 1 homolog | 1 | o | | | | | | | |
| vitellogenin a | 1 | | | o | | | | | |
| vitellogenin receptor | 1 | | | | | | o | | |
| v-mos moloney murine sarcoma viral oncogene homolog | 1 | | | | o | | | | |
| v-myc myelocytomatosis viral oncogene homolog lung carcinoma derived | 1 | o | | | | | | | |
| voltage-dependent anion channel isoform cra_a | 1 | | | | | | | | o |
| voltage-dependent anion-selective channel protein 2-like | 1 | | | | | | | | o |
| voltage-dependent calcium channel gamma-7 subunit | 1 | | | | | | o | | |
| voltage-dependent p q-type calcium channel subunit alpha-1a-like | 1 | | | | | | | o | |
| voltage-dependent t-type calcium channel subunit alpha-1h-like | 1 | | | | | | o | | |
| voltage-gated potassium channel | 1 | | | | | | o | | |
| voltage-gated potassium channel subunit beta-1 | 1 | | | | | | o | | |
| vomeronasal type-2 receptor 1- partial | 1 | | | | | | o | | |
| vomeronasal type-2 receptor 1-like | 1 | | | | | | | | o |
| vomeronasal type-2 receptor 26-like | 1 | | | | | | o | | |
| vps20-associated 1 homolog ( cerevisiae) | 1 | o | | | | | | | |
| v-ral simian leukemia viral oncogene homolog b (ras related gtp binding protein) | 1 | o | | | | | | | |
| v-rel reticuloendotheliosis viral oncogene homolog | 1 | o | | | | | | | |
| v-set and immunoglobulin domain containing 10 like | 1 | | | | o | | | | |
| v-set and immunoglobulin domain-containing protein 10-like | 1 | | | | o | | | | |
| v-set and transmembrane domain containing 2 | 1 | | | | | | o | | |
| v-type proton atpase 116 kda subunit a isoform 1-like isoform 2 | 1 | | | | o | | | | |
| v-type proton atpase 116 kda subunit a isoform 4 | 1 | | | | | | | | o |
| v-type proton atpase catalytic subunit a | 1 | | | | | | | | o |
| v-type proton atpase subunit c 1-b-like | 1 | | | | o | | | | |
| v-type proton atpase subunit d 1 | 1 | | | | o | | | | |

| | | | | | | | | | |
|---|---|---|---|---|---|---|---|---|---|
| was protein homolog associated with golgi membranes and microtubules | 1 | o | | | | | | | |
| wash complex subunit 7-like | 1 | | | | o | | | | |
| wbscr14 isoform 2 | 1 | | o | | | | | | |
| wd repeat and fyve domain containing 2 | 1 | | o | | | | | | |
| wd repeat and fyve domain containing 3 isoform 1 | 1 | | | | | | | o | |
| wd repeat and fyve domain containing 3 isoform 2 | 1 | o | | | | | | | |
| wd repeat and fyve domain-containing protein 3 | 1 | | | | | | o | | |
| wd repeat and socs box-containing 1 | 1 | o | | | | | | | |
| wd repeat and socs box-containing protein 2-like | 1 | | | | | | | | o |
| wd repeat domain 12 | 1 | o | | | | | | | |
| wd repeat domain 18 | 1 | | o | | | | | | |
| wd repeat domain 19 | 1 | o | | | | | | | |
| wd repeat domain 20 | 1 | o | | | | | | | |
| wd repeat domain 26 | 1 | | | | | | o | | |
| wd repeat domain 4 | 1 | o | | | | | | | |
| wd repeat domain 41 | 1 | o | | | | | | | |
| wd repeat domain 47 | 1 | | | | | | o | | |
| wd repeat domain 59 | 1 | | o | | | | | | |
| wd repeat domain 7 | 1 | | | | | | o | | |
| wd repeat domain 77 | 1 | | | | | o | | | |
| wd repeat domain 81 | 1 | | o | | | | | | |
| wd repeat domain phosphoinositide-interacting protein 2-like | 1 | | | | | | | | o |
| wd repeat phosphoinositide interacting 2 | 1 | o | | | | | | | |
| wd repeat-containing protein 1 | 1 | | | | | | | | o |
| wd repeat-containing protein 11 | 1 | | | | | | | | o |
| wd repeat-containing protein 17 | 1 | | | | | | | | o |
| wd repeat-containing protein 3 | 1 | o | | | | | | | |
| wd repeat-containing protein 44 | 1 | o | | | | | | | |
| wd repeat-containing protein 59 | 1 | | | | | | | | o |
| wd repeat-containing protein 61 | 1 | o | | | | | | | |
| wd repeat-containing protein 7 isoform 1 | 1 | | | | | | | | o |
| wd repeat-containing protein 70 | 1 | o | | | | | | | |
| wd repeat-containing protein 73 | 1 | | | | o | | | | |
| wd repeat-containing protein c10orf79-like | 1 | | | | | | | o | |
| wd40 repeat-containing protein smu1 | 1 | | | | o | | | | |
| wdr5 protein | 1 | | | | o | | | | |
| wd-repeat protein | 1 | o | | | | | | | |
| wee1 homolog ( pombe) | 1 | | o | | | | | | |
| werner syndrome homolog | 1 | | o | | | | | | |
| wibg homolog | 1 | | | | | | o | | |
| williams beuren syndrome chromosome region 27 | 1 | o | | | | | | | |
| williams-beuren syndrome chromosomal region 18 protein homolog | 1 | o | | | | | | | |
| williams-beuren syndrome chromosomal region 27 protein | 1 | | | | o | | | | |
| wings apart-like homolog | 1 | | | | | | o | | |
| wiskott-aldrich syndrome protein | 1 | | | | | | o | | |
| wiskott-aldrich syndrome protein family member 2-like | 1 | | | | o | | | | |
| with ankyrin repeat and ph domain 2 | 1 | o | | | | | | | |
| with ankyrin repeat and ph domain 3 | 1 | o | | | | | | | |
| with gtpase ankyrin repeat and ph domain 1 | 1 | | | | | | o | | |
| wnk lysine deficient protein kinase 1 | 1 | o | | | | | | | |

| | | | | | | | | | |
|---|---|---|---|---|---|---|---|---|---|
| wnk lysine deficient protein kinase 3 | 1 | o | | | | | | | |
| wnk lysine deficient protein kinase 3-like | 1 | | | | | | o | | |
| wnt1 inducible signaling pathway protein 1 | 1 | o | | | | | | | |
| wolf-hirschhorn syndrome candidate 1-like 1 | 1 | o | | | | | | | |
| wolframin | 1 | o | | | | | | | |
| wsc domain-containing protein 2 | 1 | o | | | | | | | |
| wu: partial | 1 | o | | | | | | | |
| wu:fa99c08 protein | 1 | o | | | | | | | |
| wu:fb54a03 protein | 1 | o | | | | | | | |
| wu:fd12d03 protein | 1 | o | | | | | | | |
| wu:fi13g07 protein | 1 | | o | | | | | | |
| wu:fy63c09 protein | 1 | o | | | | | | | |
| ww domain-containing oxidoreductase | 1 | | | | | | o | | |
| xaa-pro aminopeptidase 1 | 1 | | | | | | o | | |
| xanthine dehydrogenase | 1 | | o | | | | | | |
| xanthine dehydrogenase oxidase | 1 | | | | o | | | | |
| xdrp1 protein | 1 | | | o | | | | | |
| xin actin-binding repeat containing 2 isoform 1 | 1 | o | | | | | | | |
| xk-related protein 2b | 1 | o | | | | | | | |
| xnf7 protein | 1 | | o | | | | | | |
| xnop56 protein | 1 | | | | o | | | | |
| xpg-complementing protein | 1 | | o | | | | | | |
| x-ray repair complementing defective repair in chinese hamster cells 1 | 1 | o | | | | | | | |
| x-ray repair complementing defective repair in chinese hamster cells 5 | 1 | o | | | | | | | |
| x-ray repair complementing defective repair in chinese hamster cells 5 (double-strand-break rejoining ku 80kda) | 1 | | | | | o | | | |
| x-ray repair complementing defective repair in chinese hamster cells isoform cra_b | 1 | | o | | | | | | |
| x-ray repair cross-complementing protein 5 | 1 | | | | | | | | o |
| xylulokinase homolog | 1 | o | | | | | | | |
| xylulokinase homolog ( influenzae) | 1 | | o | | | | | | |
| y chain e2~ubiquitin-hect | 1 | | | | | | | | |
| y+l amino acid transporter 2 | 1 | | | | | | | | o |
| yes-associated protein isoform cra_b | 1 | | | | | | o | | |
| yip1 domain member 2 | 1 | | | o | | | | | |
| yippee-like 3 | 1 | o | | | | | | | |
| yth domain family protein 1 | 1 | | | | | | o | | |
| yth domain family protein 3 | 1 | | | | | | o | | |
| yth domain member 1 | 1 | o | | | | | | | |
| yy1 associated factor 2 | 1 | o | | | | | | | |
| zan_mouse ame: full=zonadhesin flags: precursor | 1 | o | | | | | | | |
| zcchc8 protein | 1 | | | | | o | | | |
| zer-1 homolog | 1 | o | | | | | | | |
| zer-1 homolog ( elegans) | 1 | o | | | | | | | |
| zeta-chain associated protein kinase 70kda | 1 | | o | | | | | | |
| zgc:103559 protein | 1 | | o | | | | | | |
| Zgc:109744 [Danio rerio] | 1 | o | | | | | | | |
| zgc:112084 protein | 1 | o | | | | | | | |
| zgc:113346 protein | 1 | | o | | | | | | |
| zgc:113362 protein | 1 | o | | | | | | | |
| zgc:136396 protein | 1 | | | | o | | | | |
| zgc:152783 protein | 1 | | | | | | o | | |
| zgc:152785 protein | 1 | o | | | | | | | |
| zgc:152809 protein | 1 | | | o | | | | | |
| zgc:152984 protein | 1 | o | | | | | | | |
| zgc:153766 protein | 1 | | | o | | | | | |

| | | | | | | | | | |
|---|---|---|---|---|---|---|---|---|---|
| zgc:153955 protein | 1 | | o | | | | | | |
| zgc:154074 protein | 1 | | | | | | | o | |
| zgc:158151 protein | 1 | o | | | | | | | |
| zgc:158157 protein | 1 | o | | | | | | | |
| zgc:158748 protein | 1 | o | | | | | | | |
| zgc:162183 protein | 1 | | | | | | o | | |
| zgc:162331 protein | 1 | | | | | | o | | |
| zgc:162544 protein | 1 | | o | | | | | | |
| zgc:162608 protein | 1 | | o | | | | | | |
| zgc:162816 protein | 1 | | o | | | | | | |
| zgc:163083 protein | 1 | | | | | o | | | |
| zgc:165344 protein | 1 | o | | | | | | | |
| zgc:165461 protein | 1 | | | | | | | | o |
| zgc:165647 protein | 1 | | | | | o | | | |
| zgc:172067 protein | 1 | o | | | | | | | |
| zgc:172086 protein | 1 | o | | | | | | | |
| zgc:172122 protein | 1 | | | | | | | o | |
| zgc:172270 protein | 1 | o | | | | | | | |
| zgc:174698 protein | 1 | o | | | | | | | |
| zgc:175171 protein | 1 | o | | | | | | | |
| Zgc:56417 [Danio rerio] | 1 | o | | | | | | | |
| zgc:56719 protein | 1 | | o | | | | | | |
| zgc:63470 protein | 1 | o | | | | | | | |
| zgc:77056 protein | 1 | | | | | | | o | |
| zgc:77713 protein | 1 | | | o | | | | | |
| zic family member 1 | 1 | | | | | o | | | |
| zinc an1-type domain 1 | 1 | o | | | | | | | |
| zinc an1-type domain 5 | 1 | o | | | | | | | |
| zinc c3hc type 1 | 1 | o | | | | | | | |
| zinc c3hc-type containing isoform cra_e | 1 | o | | | | | | | |
| zinc cchc domain containing 11 | 1 | o | | | | | | | |
| zinc cchc domain containing 14 | 1 | o | | | | | | | |
| zinc dhhc-type containing 12 | 1 | o | | | | | | | |
| zinc dhhc-type containing 13 | 1 | o | | | | | | | |
| zinc dhhc-type containing 14 isoform 2 | 1 | o | | | | | | | |
| zinc dhhc-type containing 16 | 1 | o | | | | | | | |
| zinc finger (chy type) | 1 | | o | | | | | | |
| zinc finger and btb domain-containing protein 17 | 1 | o | | | | | | | |
| zinc finger and btb domain-containing protein 38 | 1 | | | | | | o | | |
| zinc finger and btb domain-containing protein 46 | 1 | | | | | | o | | |
| zinc finger and btb domain-containing protein 8b-like | 1 | | | | | | o | | |
| zinc finger and scan domain-containing protein 20 | 1 | o | | | | | | | |
| zinc finger and scan domain-containing protein 29 | 1 | | | | o | | | | |
| zinc finger ccch domain-containing protein 11a | 1 | o | | | | | | | |
| zinc finger ccch domain-containing protein 6-like | 1 | | | | | | o | | |
| zinc finger ccch domain-containing protein 7b | 1 | | | | | | o | | |
| zinc finger ccch type containing 11a-like | 1 | o | | | | | | | |
| zinc finger ccch-type containing 11a | 1 | | o | | | | | | |
| zinc finger cchc domain-containing protein 3-like | 1 | | | | o | | | | |
| zinc finger cchc domain-containing protein 8-like | 1 | | | | o | | | | |

| | | | | | | | | | |
|---|---|---|---|---|---|---|---|---|---|
| zinc finger fyve domain-containing protein 1 | 1 | o | | | | | | | |
| zinc finger hit domain-containing protein 1-like | 1 | | | | o | | | | |
| zinc finger homeobox 3 isoform 1 | 1 | | | | | | o | | |
| zinc finger multitype 2 | 1 | | | | | o | | | |
| zinc finger mym-type protein 3-like | 1 | | | | | | | | o |
| zinc finger mym-type protein partial | 1 | o | | | | | | | |
| zinc finger mynd domain-containing protein 17-like | 1 | o | | | | | | | |
| zinc finger protein 109 | 1 | | | | | o | | | |
| zinc finger protein 112 homolog isoform 2 | 1 | | | | | | o | | |
| zinc finger protein 135- partial | 1 | | | | | | | | o |
| zinc finger protein 143 | 1 | | | | | | | | o |
| zinc finger protein 16 | 1 | o | | | | | | | |
| zinc finger protein 160-like | 1 | o | | | | | | | |
| zinc finger protein 184 | 1 | o | | | | | | | |
| zinc finger protein 187 | 1 | o | | | | | | | |
| zinc finger protein 189 | 1 | o | | | | | | | |
| zinc finger protein 207 | 1 | | o | | | | | | |
| zinc finger protein 238 | 1 | o | | | | | | | |
| zinc finger protein 265 | 1 | | o | | | | | | |
| zinc finger protein 271 | 1 | | o | | | | | | |
| zinc finger protein 280c | 1 | | | | o | | | | |
| zinc finger protein 287 | 1 | o | | | | | | | |
| zinc finger protein 318 | 1 | o | | | | | | | |
| zinc finger protein 323 | 1 | o | | | | | | | |
| zinc finger protein 333 | 1 | | | | o | | | | |
| zinc finger protein 36 c3h type-like 1 | 1 | | | | | | | o | |
| zinc finger protein 384 isoform 2 | 1 | | | | | | | | o |
| zinc finger protein 385a | 1 | | | | | | o | | |
| zinc finger protein 385c | 1 | | | | | | | o | |
| zinc finger protein 395 | 1 | o | | | | | | | |
| zinc finger protein 395-like isoform 2 | 1 | | o | | | | | | |
| zinc finger protein 407 | 1 | | | | | | o | | |
| zinc finger protein 449 | 1 | | | | | | | o | |
| zinc finger protein 451 | 1 | o | | | | | | | |
| zinc finger protein 45-like | 1 | o | | | | | | | |
| zinc finger protein 510 | 1 | | | | | | | o | |
| zinc finger protein 511 | 1 | | | | o | | | | |
| zinc finger protein 512b | 1 | o | | | | | | | |
| zinc finger protein 518 | 1 | | | | | o | | | |
| zinc finger protein 518a | 1 | o | | | | | | | |
| zinc finger protein 518a-like | 1 | | | | o | | | | |
| zinc finger protein 518b | 1 | o | | | | | | | |
| zinc finger protein 532 | 1 | | | | | o | | | |
| zinc finger protein 536 | 1 | | | | | | o | | |
| zinc finger protein 536-like | 1 | o | | | | | | | |
| zinc finger protein 54 | 1 | o | | | | | | | |
| zinc finger protein 543 | 1 | | | | | | o | | |
| zinc finger protein 551-like | 1 | o | | | | | | | |
| zinc finger protein 560 | 1 | o | | | | | | | |
| zinc finger protein 567 | 1 | o | | | | | | | |
| zinc finger protein 570-like | 1 | o | | | | | | | |
| zinc finger protein 595 | 1 | | | | | o | | | |
| zinc finger protein 595-like | 1 | | o | | | | | | |
| zinc finger protein 598 | 1 | o | | | | | | | |
| zinc finger protein 599 | 1 | | | | | | o | | |
| zinc finger protein 600 | 1 | o | | | | | | | |
| zinc finger protein 61 | 1 | | | | | | o | | |
| zinc finger protein 613 | 1 | | | | | | o | | |

| | | | | | | | | | |
|---|---|---|---|---|---|---|---|---|---|
| zinc finger protein 618 | 1 | | | | | | o | | |
| zinc finger protein 629 | 1 | o | | | | | | | |
| zinc finger protein 630 | 1 | | | | | | o | | |
| zinc finger protein 638-like | 1 | o | | | | | | | |
| zinc finger protein 64 isoforms 1 and 2-like | 1 | | | | o | | | | |
| zinc finger protein 644 | 1 | | | o | | | | | |
| zinc finger protein 648 | 1 | o | | | | | | | |
| zinc finger protein 650 | 1 | | o | | | | | | |
| zinc finger protein 653-like | 1 | | | | | | | | o |
| zinc finger protein 654 | 1 | | o | | | | | | |
| zinc finger protein 654-like | 1 | | | | o | | | | |
| zinc finger protein 658 | 1 | o | | | | | | | |
| zinc finger protein 687 | 1 | o | | | | | | | |
| zinc finger protein 711 | 1 | | o | | | | | | |
| zinc finger protein 718 | 1 | o | | | | | | | |
| zinc finger protein 729-like | 1 | | o | | | | | | |
| zinc finger protein 93 | 1 | o | | | | | | | |
| zinc finger protein ap-zic | 1 | | | | | o | | | |
| zinc finger protein basonuclin-2 | 1 | | | | o | | | | |
| zinc finger protein c3h homolog | 1 | | | | | | o | | |
| zinc finger protein c3h type-like 2 | 1 | o | | | | | | | |
| zinc finger protein eos | 1 | o | | | | | | | |
| zinc finger protein kiaa0543 | 1 | o | | | | | | | |
| zinc finger protein of the cerebellum 5 | 1 | | | | | o | | | |
| zinc finger protein ozf | 1 | | | | o | | | | |
| zinc finger protein partial | 1 | o | | | | | | | |
| zinc finger protein pseudogene | 1 | | | | | | | o | |
| zinc finger protein rlf | 1 | | | | | | | | o |
| zinc finger protein with krab and scan domains 4 | 1 | | | | o | | | | |
| zinc finger protein y-linked | 1 | o | | | | | | | |
| zinc finger protein zfpm2 | 1 | | | | | | o | | |
| zinc finger protein zic 4 | 1 | | | | | | | | o |
| zinc finger swim domain-containing protein 5 | 1 | | | | | | o | | |
| zinc finger swim domain-containing protein 6 | 1 | o | | | | | | | |
| zinc finger zz-type and ef-hand domain-containing protein 1-like | 1 | o | | | | | | | |
| zinc fingers and homeoboxes 2 | 1 | o | | | | | | | |
| zinc fingers and homeoboxes 3 | 1 | o | | | | | | | |
| zinc fingers and homeoboxes protein 1 | 1 | | | | o | | | | |
| zinc fingers and homeoboxes protein 1-like | 1 | | | | | | o | | |
| zinc fyve domain containing 21 | 1 | | | | | | o | | |
| zinc fyve domain containing 9 | 1 | | | | | | | o | |
| zinc knuckle (cchc-type) family protein | 1 | | | | | o | | | |
| zinc matrin type 2 | 1 | o | | | | | | | |
| zinc mynd domain containing 12 | 1 | o | | | | | | | |
| zinc mynd domain containing isoform cra_a | 1 | o | | | | | | | |
| zinc mynd-type containing 17 | 1 | o | | | | | | | |
| zinc mynd-type containing 8 | 1 | | | o | | | | | |
| zinc phosphodiesterase elac protein 2 | 1 | | | | | o | | | |
| zinc ran-binding domain containing 1 | 1 | | | | | | o | | |
| zinc swim domain containing 5 | 1 | o | | | | | | | |
| zinc transporter 6 | 1 | o | | | | | | | |
| zinc transporter 8-like | 1 | | | | o | | | | |
| zinc transporter zip11 | 1 | | | | | | o | | |
| zinc transporter zip13 | 1 | | | o | | | | | |
| zinc ttf-type hat dimerisation nucleic acid- ob-fold | 1 | o | | | | | | | |
| zinc zz-type with ef hand domain 1 isoform 2 | 1 | o | | | | | | | |
| zinc-binding protein a33-like | 1 | | | | | | | | o |

| | | | | | | |
|---|---|---|---|---|---|---|
| zinedin isoform 2 | 1 | | o | | | |
| zmym1, putative [Perkinsus marinus ATCC 50983] | 1 | o | | | | |
| zmynd8 protein | 1 | o | | | | |
| zn-binding protein | 1 | o | | | | |
| znf460 protein | 1 | | | o | | |
| zona pellucida glycoprotein 1 | 1 | | | o | | |
| zona pellucida glycoprotein 2 | 1 | | | o | | |
| zona pellucida glycoprotein 3 | 1 | | | o | | |
| zona pellucida glycoprotein 3 b | 1 | | | o | | |
| zona pellucida protein x | 1 | | | o | | |
| zona pellucida sperm-binding protein 1 | 1 | | | o | | |
| zona pellucida sperm-binding protein 2 precursor | 1 | | | o | | |
| zona pellucida sperm-binding protein 3 | 1 | | | o | | |
| zpa domain containing protein | 1 | o | | | | |
| ZPC5 [Cynoglossus semilaevis] | 1 | | | o | | |
| zuotin related factor partial | 1 | o | | | | |
| zw10 protein | 1 | o | | | | |
| zyg-ll homolog b ( elegans) | 1 | | o | | | |
| zygotic dna replication licensing factor mcm6-b | 1 | | | | o | |
| zymogen granule membrane protein 16 precursor | 1 | | | | | o |
| zymogen granule membrane protein 16-like | 1 | | | | | o |

* NC, *N. coriiceps* ; PA, *P. antarcticum* ; CA, *C. aceratus* ; DM, *D. mawsoni.*
