# Supplementary material for: Transcriptomics and Comparative Analysis of Three Antarctic Notothenioid Fishes
Source: PLoS One. 2012 Aug 16;7(8):e43762. doi: 10.1371/journal.pone.0043762 (PMC3420891; doi:10.1371/journal.pone.0043762)
Supplement: Table S5 — Putatively identified globin genes in the three notothenioid fishes. (PDF) [file pone.0043762.s007.pdf]

Table S5. Putatively identified globin genes in the three notothenioid fishes

Contig ID	length (bp)	No. of Reads	Sequence Description	E-Value
NCL contig08253	631	156	alpha globin	5.49E-76
NCL contig09151	577	90	hemoglobin subunit beta-2	1.68E-75
NCL contig17881	540	70	alpha-type globin	7.6E-74
NCL contig08254	505	63	alpha globin	4.02E-57
NCL contig17037	620	52	hemoglobin beta chain	2.67E-80
NCL contig10578	843	41	hemoglobin subunit alpha-d	5.52E-58
NCB contig01248	635	18	alpha globin	5.55E-76
NCB contig01425	610	12	hemoglobin beta chain	2.61E-80
NCB contig02375	567	10	hemoglobin subunit beta-2	2.71E-75
NCB contig06672	497	8	alpha-type globin	1.55E-74
PAB contig07333	300	24	alpha-type globin	8.57E-37
PAB contig04673	472	9	hemoglobin beta chain	4.52E-62
PAL 196Contig1	634	78	alpha globin	2.1E-67
PAL 349Contig1	554	45	hemoglobin subunit beta-2	1.13E-67
PAL 385Contig1	525	38	alpha-type globin	8.36E-69
PAL 385Contig2	431	3	alpha-type globin	8.31E-61
CAB contig05756	575	4	cytoglobin	9.42E-39
CAL contig00930	812	14	cytoglobin	6.38E-69
